# A multimodal embedding model for sepsis data representation

**DOI:** 10.1038/s41746-026-02446-3

**Published:** 2026-02-23

**Authors:** Tuo Liu, Yonglin Li, Hongyi Chen, Naiqing Li, Yan Zhang, Xuanqi Huang, Jin Wang, Rui Chen, Yuping Zeng, Yuntao Liu, Danwen Zheng, Darong Wu, Changdong Wang, Tao Yu, Xiaotu Xi, Zhongde Zhang

**Affiliations:** 1 School of Computer Science and Engineering, Sun Yat-sen University, Guangzhou, China; 2 Guangzhou University of Chinese Medicine, Guangzhou, China; 3 The Second Affiliated Hospital of Guangzhou University of Chinese Medicine (Guangdong Provincial Hospital of Chinese Medicine), Guangzhou, China; 4 Guangdong Provincial Key Laboratory of Research on Emergency in TCM, Guangzhou, China; 5 Department of Emergency Medicine, Sun Yat-sen Memorial Hospital, Sun Yat-sen University, Guangzhou, China; 6 Institute of Cardiopulmonary Cerebral Resuscitation, Sun Yat-Sen University, Guangzhou, China; 7 Information Management Office, The Second Affiliated Hospital of Guangzhou University of Chinese Medicine (Guangdong Provincial Hospital of Chinese Medicine), Guangzhou, China; 8 State Key Laboratory of Traditional Chinese Medicine Syndrome, Guangzhou, China

**Keywords:** Biomarkers, Computational biology and bioinformatics, Diseases, Mathematics and computing, Medical research

## Abstract

Sepsis research has long been constrained by limited labeled data and models designed for specific tasks that primarily rely on tabular inputs, overlooking the valuable insights contained in clinical text. To address these limitations, we propose the Sepsis Data Representation Model (SepsisDRM), an embedding model that jointly processes tabular and textual data to capture comprehensive patient representations. Trained on a dataset comprising 19,526 sepsis patients, SepsisDRM demonstrates strong generalization across diverse sepsis-related tasks without task-specific tuning. It effectively stratifies patients into four clinically interpretable phenotypes and achieves robust performance in predicting 28-day outcomes, with AUC scores of 0.92, 0.94, and 0.78 on retrospective, prospective, and external datasets, respectively. As the first embedding model developed specifically for sepsis, SepsisDRM establishes a novel paradigm for sepsis research and offers a promising approach for studies in other fields that involve the integration of both tabular and textual data.

## Introduction

Sepsis is a life-threatening syndrome characterized by a dysregulated immune response to infection, leading to multi-organ dysfunction^1^. Despite extensive global efforts, including the launch of the Surviving Sepsis Campaign in 2002, sepsis and septic shock remain significant public health concerns^1,2^. In 2017, it was estimated that sepsis affected 49 million individuals worldwide, resulting in 11 million deaths, accounting for ~20% of global mortality^3^. The complexity of sepsis, which arises from interactions among various pathogens, infection sites, and host immune responses, presents substantial challenges in both diagnosis and treatment^4–6^. In addition, research in the sepsis domain spans a broad range of objectives, including phenotype identification^7,8^, outcome prediction^9,10^, and treatment optimization^11,12^. These tasks are highly diverse, often necessitating the design and training of specialized models for each task and scenario. This process is both time-consuming and resource-intensive, and the models may fail to perform adequately in cases where data are limited. Consequently, the development of an embedding model capable of addressing multiple sepsis-related tasks efficiently is imperative to enhance both efficacy and performance across the field.

Embedding models have shown success in extracting representations from medical images and textual information, which are effectively applied to various downstream tasks^13–17^. However, there has been no research on embedding models for sepsis data representation. Sepsis patient phenotyping and prognosis prediction are two critical tasks in sepsis research. Phenotyping aims to categorize sepsis patients into distinct subgroups based on their clinical presentations and physiological characteristics, thereby identifying groups with different pathological mechanisms and therapeutic needs. Previous approaches largely relied on clustering algorithms tailored to specific datasets, making it difficult to generalize across different scenarios and datasets^8^. In parallel, different data modalities have been leveraged for prognosis prediction. Tabular-only methods, based on demographics, laboratory results, or severity scores, have long been applied for risk stratification and outcome prediction in sepsis^7,18,19^. While interpretable and widely available, these approaches are limited by the scope of structured variables. Text-only studies have utilized clinical notes and radiology reports with machine learning and transformer-based models, demonstrating the value of unstructured information^20,21^. However, these methods often suffer from heterogeneity across institutions and limited generalizability. More recently, multimodal approaches have attempted to combine structured and unstructured inputs, showing performance gains over unimodal baselines^22,23^, though they typically remain dataset-specific and lack the scalability of a foundational embedding model. In addition, some studies have explored using highly accessible variables such as complete blood count (CBC) to build lightweight prognostic tools^24,25^. While pragmatic, such approaches cannot fully capture the complex heterogeneity of sepsis. Together, these lines of work highlight the promise of leveraging diverse modalities, while also underscoring the need for a generalizable multimodal embedding framework.

**Fig. 1:**
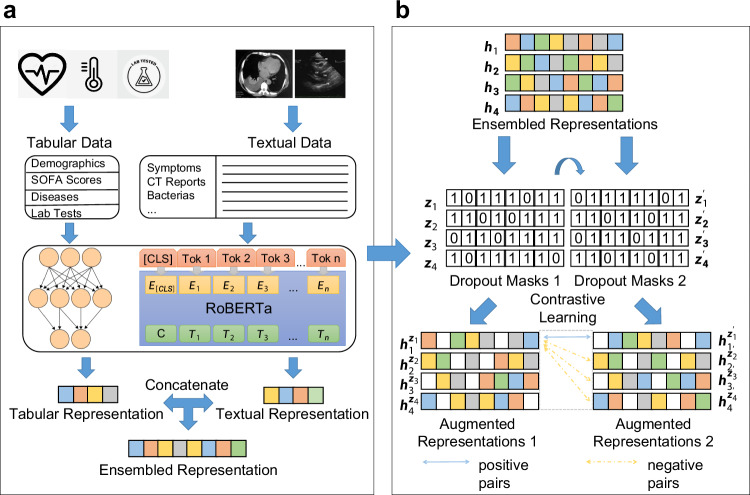
Illustration of the structure of our SepsisDRM. **a** The framework of SepsisDRM. The tabular data and textual data are processed through an MLP and RoBERTa, respectively, to obtain tabular representation and textual representation, which are ultimately concatenated to form the ensembled representation. **b** The training process of SepsisDRM. For a sample *x*_*i*_, its ensembled representation **h**_*i*_ is passed through the encoder twice with different dropout masks ${z}_{i},{z}_{i}^{{\prime} }$, resulting in two distinct representations ${{\bf{h}}}_{i}^{{z}_{i}},{{\bf{h}}}_{i}^{{z}_{i}^{{\prime} }}$. These representations are considered positive sample pairs, whereas representations derived from different samples, taking ${{\bf{h}}}_{i}^{{z}_{i}}$ and ${{\bf{h}}}_{j}^{{z}_{j}^{{\prime} }}$ as example, are regarded as negative sample pairs.

In this paper, we present Sepsis Data Representation Model (SepsisDRM), an embedding model tailored for sepsis research, trained on both tabular data and textual data from 19,526 sepsis patients from Guangdong Provincial Hospital of Chinese Medicine (GDHCM) and its four branch hospitals. SepsisDRM employs the multi-layer perceptron (MLP) and Robustly optimized BERT approach (RoBERTa)^26^ as its backbone (Fig. 1a) and is trained using a contrastive learning mechanism (Fig. 1b). Compared to most other embedding models applied in the medical domain, our SepsisDRM possesses unique characteristics. While many existing embedding models are primarily designed for image data^15^ or image-text pairs^16,17^, SepsisDRM is tailored for multi-modal data comprised of structured tabular data and textual information, which enhances representations by passing them through two different dropout layers to generate positive and negative sample pairs for contrastive learning. Through the process of contrastive learning, information is exchanged between the two modalities of tabular data and textual data. Furthermore, the architecture of SepsisDRM is extensible to other fields, as long as the input data includes tabular and textual data, offering a novel approach for exploring embedding models in diverse domains.

**Fig. 2:**
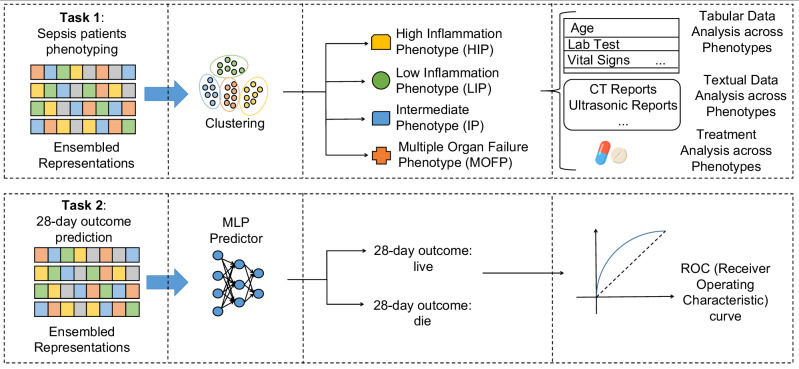
Application of the embedding model in the two downstream tasks. The embedding model takes as input the original sepsis data and outputs the representations of sepsis data, which can be applied to various downstream tasks such as clustering sepsis phenotypes and predicting 28-day outcomes for patients.

**Fig. 3:**
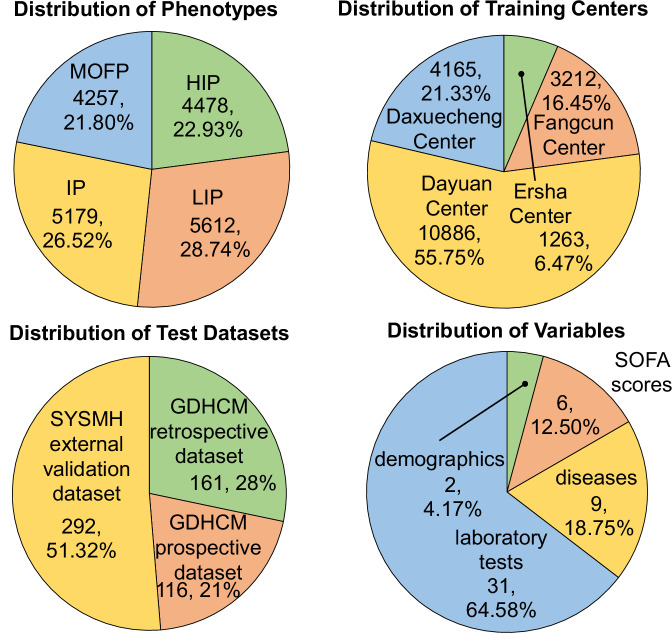
Distribution of four training centers, three validation datasets, 48 included variables and four phenotypes.

To evaluate the performance of SepsisDRM, we conducted two downstream tasks, including sepsis patient phenotyping and 28-day outcome prediction, as shown in Fig. 2, both of which yielded promising results. In the phenotyping task, we conducted clustering on a dataset comprising 19,526 patients from GDHCM, clustering all sepsis patients into four phenotypes: high inflammation phenotype (HIP), low inflammation phenotype (LIP), intermediate phenotype (IP), and multiple organ failure phenotype (MOFP). Subsequently, we analyzed the tabular data across different phenotypes and observed significant differences in variables such as laboratory test results, microbial cultures, and in-hospital mortality rates. Additionally, we performed an analysis of textual data, comparing the frequency of term occurrences across phenotypes, which corroborated the findings from the tabular data analysis. Furthermore, we compared the medication usage among different phenotypes, revealing that the drug Xuebijing (XBJ for short) was more effective for patients with the HIP phenotype. For the 28-day outcome prediction task, we conducted testing on the retrospective dataset from GDHCM, the prospective dataset from GDHCM, and the external validation set from Sun Yat-sen Memorial Hospital, Sun Yat-sen University (SYSMH), as shown in Fig. 3. And the proposed model achieved AUC scores of 0.92, 0.94, and 0.78, respectively. Besides, in our study, we also compared the performance of human medical experts and the SepsisDRM model in predicting 28-day outcomes for sepsis patients, demonstrating that the data-driven SepsisDRM significantly outperformed the experts in accuracy and consistency. To the best of our knowledge, SepsisDRM is the first embedding model for sepsis data representation that is capable of processing multimodal input data, including tabular data and textual data, thereby offering a novel approach for sepsis studies.

## Results

### Dataset introduction

In this study, we systematically collected and analyzed data from five centers across two hospitals, namely Guangdong Provincial Hospital of Chinese Medicine (GDHCM) and Sun Yat-sen Memorial Hospital, Sun Yat-sen University (SYSMH), to evaluate the effectiveness and generalizability of our model. Specifically, the training dataset was derived from four centers within GDHCM, namely Ersha Center, Fangcun Center, Dayuan Center, and Daxuecheng Center, providing rich representation information for embedding model training. These four centers include data from 1263, 3212, 10,886, and 4165 patients, respectively, as shown in Fig. 3. To ensure the model’s applicability and robustness across different settings, we designed a comprehensive validation including three datasets, namely the GDHCM retrospective dataset, GDHCM prospective dataset, and SYSMH external validation dataset. These three validation datasets include data from 161, 116, and 292 patients, respectively, as shown in Fig. 3.

**Fig. 4:**
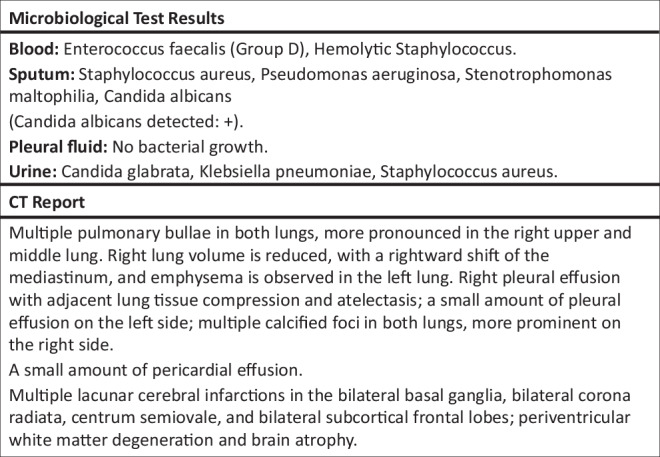
An example of microbiological test results and CT reports.

In terms of variable selection, the SepsisDRM model uses both tabular and textual variables for training and testing. The 48 tabular variables can be categorized into four groups: demographic information, Sequential Organ Failure Assessment (SOFA) scores, diseases, and laboratory tests (Lab tests), as shown in Fig. 3. Demographic information includes *Age* and *Sex*. SOFA scores include *SOFA Score, SOFA Respiratory System Score, SOFA Cardiovascular System Score, SOFA Liver Score, SOFA Coagulation Score*, and *SOFA Renal Function Score*. The SOFA score for each system is divided into five levels: 0, 1, 2, 3, and 4, with higher numbers indicating greater severity, and the *SOFA score* is the sum of the SOFA scores for each individual system. Diseases include *Hypertension, Coronary Artery Disease (CAD), Diabetes Mellitus (DM), Chronic Obstructive Pulmonary Disease (COPD), Chronic Kidney Disease (CKD), Cardiovascular Disease (CVD), Chronic Liver Disease, Hematological Malignancy*, and *Tumor*. All of these are binary variables, where “1” indicates that the patient has the condition, and “0” indicates he/she do not. Laboratory tests include *hsCRP, APTT, TT, INR, PTA, PT, FIB, WBC, HCT, RDW, LYM, Hb, PLT, PDW, NEUT, ALB, hs-cTnT, ALT, AST, Cr, K+, Na+, Urea, PA, DBIL, TBIL, TCO2, TC, LDL-C, non-HDL-C*, and *HDL-C*. The full names corresponding to the abbreviations for laboratory test results in the figure can be found in Supplementary Table 1. All of these laboratory tests are continuous variables. Detailed characteristics and statistical descriptions of these tabular variables can be found in Tables 1 and 2. Regarding textual variables, they are divided into two parts: the patient’s microbiological test results and CT report. The microbiological test results come from observing microorganisms cultured from the patient’s blood, sputum, pleural fluid, urine or other samples after a period of incubation, while the CT report is written by radiologists based on the patient’s CT images, as shown in Fig. 4. For specific data processing methods and inclusion criteria, refer to the section “Data curation”.

**Table 1:** Dataset information (Training Cohort)

Characteristic	Total	Ersha Center	Fangcun Center	Dayuan Center	Daxuecheng Center
No. of patients (%)	19,526 (100.0%)	1263 (6.5%)	3212 (16.5%)	10,886 (55.8%)	4165 (21.3%)
*Sex, No. (%)*					
Female	7668 (39.3%)	551 (43.6%)	1263 (39.3%)	4386 (40.3%)	1468 (35.2%)
Male	11,858 (60.7%)	712 (56.4%)	1949 (60.7%)	6500 (59.7%)	2697 (64.8%)
Age, mean (SD), yr	67.0 (17.1)	71.4 (15.6)	68.1 (15.7)	67.6 (17.0)	62.9 (18.0)
SOFA score, mean (SD)	5.05 (2.84)	4.40 (2.55)	4.74 (2.66)	5.17 (2.84)	5.17 (3.03)
Respiratory	2.13 (1.54)	1.70 (1.65)	2.22 (1.42)	2.24 (1.53)	1.93 (1.57)
Cardiovascular	0.49 (1.09)	0.50 (1.09)	0.48 (1.07)	0.48 (1.08)	0.54 (1.12)
Liver	0.46 (0.87)	0.42 (0.77)	0.34 (0.75)	0.46 (0.86)	0.55 (0.99)
Coagulation	0.94 (1.18)	0.87 (1.02)	0.75 (1.05)	0.99 (1.24)	0.97 (1.12)
Renal	0.85 (1.21)	0.92 (1.09)	0.80 (1.13)	0.83 (1.21)	0.94 (1.28)
*Hypertension, No. (%)*					
No	9703 (49.7%)	627 (49.6%)	1526 (47.5%)	5424 (49.8%)	2126 (51.0%)
Yes	9823 (50.3%)	636 (50.4%)	1686 (52.5%)	5462 (50.2%)	2039 (49.0%)
CAD, No. (%)					
No	15,619 (80.0%)	890 (70.5%)	2635 (82.0%)	8588 (78.9%)	3506 (84.2%)
Yes	3907 (20.0%)	373 (29.5%)	577 (18.0%)	2298 (21.1%)	659 (15.8%)
*DM, No. (%)*					
No	14,723 (75.4%)	866 (68.6%)	2403 (74.8%)	8268 (76.0%)	3186 (76.5%)
Yes	4803 (24.6%)	397 (31.4%)	809 (25.2%)	2618 (24.0%)	979 (23.5%)
*COPD, No. (%)*					
No	18,393 (94.2%)	1213 (96.0%)	3032 (94.4%)	10,203 (93.7%)	3945 (94.7%)
Yes	1133 (5.8%)	50 (4.0%)	180 (5.6%)	683 (6.3%)	220 (5.3%)
*CKD, No. (%)*					
No	17,169 (87.9%)	1106 (87.6%)	2893 (90.1%)	9640 (88.6%)	3530 (84.8%)
Yes	2357 (12.1%)	157 (12.4%)	319 (9.9%)	1246 (11.4%)	635 (15.2%)
*CVD, No. (%)*					
No	13,889 (71.1%)	990 (78.4%)	2056 (64.0%)	7862 (72.2%)	2981 (71.6%)
Yes	5637 (28.9%)	273 (21.6%)	1156 (36.0%)	3024 (27.8%)	1184 (28.4%)
Chronic liver dis., No. (%)					
No	18,324 (93.8%)	1224 (96.9%)	3058 (95.2%)	10346 (95.0%)	3696 (88.7%)
Yes	1202 (6.2%)	39 (3.1%)	154 (4.8%)	540 (5.0%)	469 (11.3%)
*Hema. malignancy, No. (%)*					
No	18,730 (95.9%)	1248 (98.8%)	3187 (99.2%)	10159 (93.3%)	4136 (99.3%)
Yes	796 (4.1%)	15 (1.2%)	25 (0.8%)	727 (6.7%)	29 (0.7%)
*Tumor, No. (%)*					
No	17,374 (89.0%)	1159 (91.8%)	2893 (90.1%)	9689 (89.0%)	3633 (87.2%)
Yes	2152 (11.0%)	104 (8.2%)	319 (9.9%)	1197 (11.0%)	532 (12.8%)
hsCRP, mg/L	66.8 [23.8, 137.0]	76.4 [25.7, 146.0]	62.1 [22.6, 128.0]	70.3 [26.3, 142.0]	59.0 [19.5, 126.0]
APTT, s	36.0 [29.7, 41.8]	28.7 [26.0, 33.0]	29.5 [26.4, 33.9]	39.7 [35.8, 44.4]	30.0 [26.5, 35.3]
TT, s	17.2 [16.1, 18.6]	17.4 [16.3, 18.6]	17.4 [16.3, 18.7]	17.2 [16.1, 18.6]	16.9 [15.7, 18.3]
INR, R	1.13 [1.04, 1.26]	1.15 [1.06, 1.25]	1.12 [1.04, 1.24]	1.14 [1.05, 1.27]	1.12 [1.02, 1.26]
PTA, %	78.3 [65.1, 91.0]	76.4 [64.2, 89.0]	74.0 [60.9, 89.0]	81.0 [69.0, 92.0]	75.2 [60.4, 89.0]
PT, s	14.0 [12.8, 15.4]	12.9 [11.9, 14.1]	13.0 [12.1, 14.3]	14.6 [13.7, 15.9]	12.9 [11.9, 14.4]
FIB, g/L	4.41 [3.19, 5.79]	4.42 [3.33, 5.91]	4.03 [2.81, 5.20]	4.66 [3.47, 6.05]	4.00 [2.82, 5.46]
WBC, 10^9^/L	10.20 [6.65, 14.70]	10.60 [6.97, 15.40]	11.00 [7.46, 15.00]	10.20 [6.51, 14.80]	9.59 [6.33, 13.80]
HCT, %	32.7 [26.8, 37.7]	33.7 [28.9, 38.5]	33.6 [27.9, 38.1]	32.3 [26.0, 37.4]	33.0 [27.2, 38.0]
RDW, %	13.8 [12.9, 15.3]	13.7 [12.9, 15.2]	13.8 [12.9, 15.3]	13.7 [12.8, 15.2]	14.0 [13.0, 15.7]
LYM, 10^9^/L	0.90 [0.57, 1.36]	0.86 [0.54, 1.30]	0.93 [0.59, 1.39]	0.89 [0.56, 1.38]	0.90 [0.58, 1.34]
Hb, g/L	108.0 [87.0, 125.0]	113.0 [95.0, 129.0]	111.0 [91.0, 127.0]	106.0 [84.0, 124.0]	110.0 [90.0, 127.0]
PLT, 10^9^/L	178 [114, 251]	173 [125, 240]	193 [131, 261]	177 [109, 250]	172 [109, 250]
PDW, fL	15.8 [13.2, 16.3]	12.7 [10.5, 16.0]	12.4 [10.8, 15.1]	16.1 [15.7, 16.4]	13.9 [11.0, 16.2]
NEUT, 10^9^/L	8.28 [4.88, 12.50]	8.77 [5.17, 13.00]	8.98 [5.69, 12.90]	8.24 [4.76, 12.60]	7.74 [4.60, 11.80]
ALB, g/L	33.5 [29.3, 37.8]	34.8 [30.7, 38.6]	33.2 [28.7, 37.5]	33.6 [29.5, 37.8]	33.2 [28.6, 37.8]
hs-cTnT, μg/L	0.03 [0.01, 0.07]	0.03 [0.01, 0.06]	0.03 [0.01, 0.07]	0.03 [0.01, 0.07]	0.03 [0.01, 0.07]
ALT, U/L	22.0 [13.0, 44.0]	20.0 [13.0, 40.0]	21.0 [12.4, 42.9]	23.0 [14.0, 45.0]	22.0 [13.0, 44.0]
AST, U/L	30.0 [20.0, 59.0]	28.0 [19.0, 53.5]	29.0 [19.4, 58.5]	31.0 [20.0, 59.0]	29.0 [18.0, 58.0]
Cr, μmol/L	91.0 [66.0, 147.0]	102.0 [74.0, 152.0]	94.0 [67.0, 143.0]	89.0 [65.6, 144.0]	93.0 [66.0, 157.0]
K+, mmol/L	3.93 [3.57, 4.34]	3.83 [3.50, 4.24]	3.92 [3.55, 4.31]	3.96 [3.58, 4.36]	3.93 [3.58, 4.33]
Na+, mmol/L	138.0 [135.0, 141.0]	138.0 [134.0, 140.0]	138.0 [134.0, 141.0]	138.0 [135.0, 142.0]	138.0 [134.0, 141.0]
Urea, mmol/L	6.86 [4.60, 11.70]	7.68 [5.25, 12.30]	6.91 [4.65, 11.20]	6.76 [4.58, 11.70]	6.80 [4.45, 12.00]
PA, mg/L	121.0 [68.9, 182.0]	128.0 [78.0, 187.0]	120.0 [68.0, 181.0]	120.0 [69.0, 180.0]	121.0 [64.0, 188.0]
DBIL, μmol/L	5.80 [3.60, 10.80]	5.80 [3.65, 10.90]	5.70 [3.60, 10.40]	5.70 [3.50, 10.50]	6.10 [3.70, 12.30]
TBIL, μmol/L	12.50 [8.00, 21.90]	12.60 [7.90, 21.60]	11.50 [7.40, 19.90]	13.00 [8.30, 22.20]	12.20 [7.40, 23.40]
TCO_2_, mmol/L	23.4 [20.4, 26.2]	22.7 [19.8, 25.0]	23.9 [21.0, 26.7]	23.7 [20.8, 26.6]	22.4 [19.5, 25.0]
TC, mmol/L	3.59 [2.85, 4.42]	3.64 [2.93, 4.40]	3.75 [3.07, 4.55]	3.54 [2.76, 4.38]	3.60 [2.85, 4.43]
LDL-C, mmol/L	2.13 [1.49, 2.83]	2.15 [1.51, 2.78]	2.26 [1.67, 2.96]	2.09 [1.43, 2.79]	2.12 [1.52, 2.84]
non-HDL-C, mmol/L	2.68 [2.05, 3.42]	2.72 [2.11, 3.43]	2.85 [2.26, 3.59]	2.60 [1.93, 3.34]	2.73 [2.12, 3.51]
HDL-C, mmol/L	0.86 [0.61, 1.13]	0.87 [0.65, 1.13]	0.85 [0.62, 1.09]	0.88 [0.61, 1.17]	0.81 [0.57, 1.05]

Retrospective data from four centers within GDHCM were used to train the model. For normally distributed continuous variables, data are presented as mean ± standard deviation (SD). For non-normally distributed continuous variables, data are presented as median with interquartile range [IQR]. Categorical variables are expressed as frequencies and percentages. Percentages may not sum to 100% due to rounding.

**Table 2:** Dataset information (Validation Cohort)

Characteristic	Total	GDHCM Retrospective Cohort	GDHCM Prospective Cohort	SYSMH External Validation Cohort
No. of patients (%)	569 (100.0%)	161 (28.3%)	116 (20.4%)	292 (51.3%)
*28-day outcome, No. (%)*				
Positive (dead)	99 (17.4%)	18 (11.2%)	18 (15.5%)	63 (21.6%)
Negative (alive)	470 (82.6%)	143 (88.8%)	98 (84.5%)	229 (78.4%)
*Sex, No. (%)*				
Female	226 (39.7%)	73 (45.3%)	33 (28.4%)	120 (41.1%)
Male	343 (60.3%)	88 (54.7%)	83 (71.6%)	172 (58.9%)
Age, mean (SD), yr	71.2 (15.8)	76.5 (14.4)	69.9 (14.4)	68.8 (16.5)
SOFA score, mean (SD)	4.42 (2.48)	5.72 (2.17)	4.69 (2.54)	3.60 (2.29)
Respiratory	1.77 (1.55)	3.32 (1.14)	1.59 (1.33)	0.99 (1.16)
Cardiovascular	0.21 (0.69)	0.19 (0.73)	0.54 (1.11)	0.10 (0.29)
Liver	0.52 (0.87)	0.39 (0.76)	0.50 (1.00)	0.60 (0.87)
Coagulation	0.65 (0.97)	0.70 (1.01)	0.92 (1.11)	0.51 (0.87)
Renal	0.95 (1.15)	1.12 (1.24)	0.97 (1.27)	0.84 (1.03)
*Hypertension, No. (%)*				
No	298 (52.4%)	75 (46.6%)	62 (53.4%)	161 (55.1%)
Yes	271 (47.6%)	86 (53.4%)	54 (46.6%)	131 (44.9%)
*CAD, No. (%)*				
No	436 (76.6%)	116 (72.0%)	78 (67.2%)	242 (82.9%)
Yes	133 (23.4%)	45 (28.0%)	38 (32.8%)	50 (17.1%)
*DM, No. (%)*				
No	410 (72.1%)	124 (77.0%)	79 (68.1%)	207 (70.9%)
Yes	159 (27.9%)	37 (23.0%)	37 (31.9%)	85 (29.1%)
*COPD, No. (%)*				
No	528 (92.8%)	152 (94.4%)	107 (92.2%)	269 (92.1%)
Yes	41 (7.2%)	9 (5.6%)	9 (7.8%)	23 (7.9%)
*CKD, No. (%)*				
No	496 (87.2%)	145 (90.1%)	83 (71.6%)	268 (91.8%)
Yes	73 (12.8%)	16 (9.9%)	33 (28.4%)	24 (8.2%)
*CVD, No. (%)*				
No	416 (73.1%)	105 (65.2%)	80 (69.0%)	231 (79.1%)
Yes	153 (26.9%)	56 (34.8%)	36 (31.0%)	61 (20.9%)
*Chronic liver dis., No. (%)*				
No	526 (92.4%)	159 (98.8%)	103 (88.8%)	264 (90.4%)
Yes	43 (7.6%)	2 (1.2%)	13 (11.2%)	28 (9.6%)
*Hema. malignancy, No. (%)*				
No	557 (97.9%)	160 (99.4%)	113 (97.4%)	284 (97.3%)
Yes	12 (2.1%)	1 (0.6%)	3 (2.6%)	8 (2.7%)
*Tumor, No. (%)*				
No	465 (81.7%)	141 (87.6%)	95 (81.9%)	229 (78.4%)
Yes	104 (18.3%)	20 (12.4%)	21 (18.1%)	63 (21.6%)
hsCRP, mg/L	97.5 [43.0, 161.0]	85.8 [40.5, 139.0]	83.5 [33.2, 136.0]	116.0 [55.3, 173.0]
APTT, s	33.8 [28.0, 40.6]	39.2 [34.7, 44.6]	36.5 [31.0, 43.0]	29.4 [26.3, 34.1]
TT, s	17.0 [16.0, 18.2]	17.0 [16.1, 18.2]	16.7 [15.7, 17.9]	17.0 [16.1, 18.3]
INR, R	1.16 [1.07, 1.29]	1.18 [1.10, 1.29]	1.19 [1.10, 1.30]	1.14 [1.05, 1.29]
PTA, %	75.0 [62.0, 85.6]	77.0 [66.0, 84.0]	75.4 [64.9, 85.5]	72.3 [59.1, 85.7]
PT, s	14.3 [13.1, 15.9]	14.7 [13.8, 15.6]	14.1 [13.2, 15.4]	13.9 [12.7, 16.5]
FIB, g/L	4.79 [3.27, 6.17]	5.11 [3.59, 6.43]	4.62 [3.30, 6.12]	4.64 [3.10, 5.92]
WBC, 10^9^/L	11.10 [7.14, 16.30]	10.20 [7.32, 15.40]	10.20 [6.13, 14.80]	12.10 [7.55, 17.60]
HCT, %	31.7 [26.0, 36.2]	29.8 [25.1, 35.0]	30.6 [24.3, 36.4]	32.0 [27.0, 36.0]
RDW, %	14.0 [13.0, 15.9]	13.7 [12.7, 15.3]	14.4 [13.1, 16.0]	14.0 [13.0, 16.0]
LYM, 10^9^/L	0.92 [0.55, 1.32]	1.00 [0.67, 1.36]	1.03 [0.60, 1.43]	0.85 [0.51, 1.24]
Hb, g/L	104.0 [83.0, 120.0]	98.0 [79.0, 116.0]	103.0 [79.0, 119.0]	107.0 [87.8, 123.0]
PLT, 10^9^/L	160 [104, 243]	198 [126, 270]	166 [115, 246]	146 [97, 217]
PDW, fL	15.0 [11.6, 16.2]	15.9 [15.3, 16.3]	16.1 [15.7, 16.4]	12.2 [10.5, 14.2]
NEUT, 10^9^/L	8.42 [5.18, 13.20]	8.03 [5.24, 12.00]	7.88 [4.64, 11.30]	8.80 [5.26, 14.40]
ALB, g/L	29.6 [25.9, 33.6]	30.7 [26.6, 34.9]	32.5 [28.2, 36.8]	28.0 [24.4, 31.6]
hs-cTnT, *μ*g/L	0.03 [0.02, 0.07]	0.05 [0.02, 0.11]	0.03 [0.01, 0.07]	0.03 [0.01, 0.06]
ALT, U/L	27.0 [15.0, 55.0]	32.0 [22.0, 60.0]	17.0 [10.8, 43.0]	27.5 [14.8, 56.5]
AST, U/L	35.0 [22.0, 69.5]	39.0 [25.0, 73.0]	26.0 [18.0, 54.5]	39.0 [23.8, 73.5]
Cr, μmol/L	106.0 [77.0, 167.0]	116.8 [76.0, 199.6]	109.0 [76.8, 175.0]	100.0 [77.0, 149.0]
K+, mmol/L	3.87 [3.49, 4.24]	3.92 [3.57, 4.33]	3.88 [3.59, 4.39]	3.82 [3.45, 4.15]
Na+, mmol/L	137.1 [134.0, 141.0]	138.1 [134.0, 142.2]	137.0 [133.0, 140.0]	137.0 [134.0, 141.0]
Urea, mmol/L	8.4 [5.5, 13.9]	8.8 [6.1, 16.0]	8.6 [5.9, 13.8]	8.0 [5.1, 13.2]
PA, mg/L	117.5 [68.0, 188.0]	80.0 [39.0, 137.4]	126.0 [77.8, 195.0]	90.0 [52.8, 141.0]
DBIL, μmol/L	5.00 [3.30, 9.30]	3.47 [2.80, 4.34]	5.55 [3.30, 11.00]	5.50 [3.40, 9.60]
TBIL, μmol/L	10.5 [6.7, 17.5]	8.8 [5.8, 14.3]	11.8 [6.9, 18.0]	11.2 [6.9, 18.7]
TCO2, mmol/L	23.6 [20.1, 26.7]	22.0 [19.0, 25.0]	24.3 [21.0, 27.2]	24.2 [20.9, 27.0]
TC, mmol/L	3.76 [3.02, 4.53]	3.59 [2.97, 4.34]	3.79 [3.04, 4.57]	3.85 [3.13, 4.63]
LDL-C, mmol/L	2.25 [1.60, 2.98]	2.15 [1.56, 2.89]	2.21 [1.56, 2.96]	2.34 [1.67, 3.02]
non-HDL-C, mmol/L	2.82 [2.21, 3.61]	2.61 [2.05, 3.40]	2.81 [2.22, 3.62]	3.01 [2.35, 3.77]
HDL-C, mmol/L	0.79 [0.52, 1.07]	0.84 [0.49, 1.20]	0.88 [0.67, 1.16]	0.72 [0.51, 0.99]

Three datasets were used for validation: a retrospective dataset from GDHCM, a prospective dataset from GDHCM, and an external validation dataset from SYSMH. Data presentation follows the same conventions as in Table 1.

### Clustering and identification of four diverse sepsis phenotypes

**Fig. 5:**
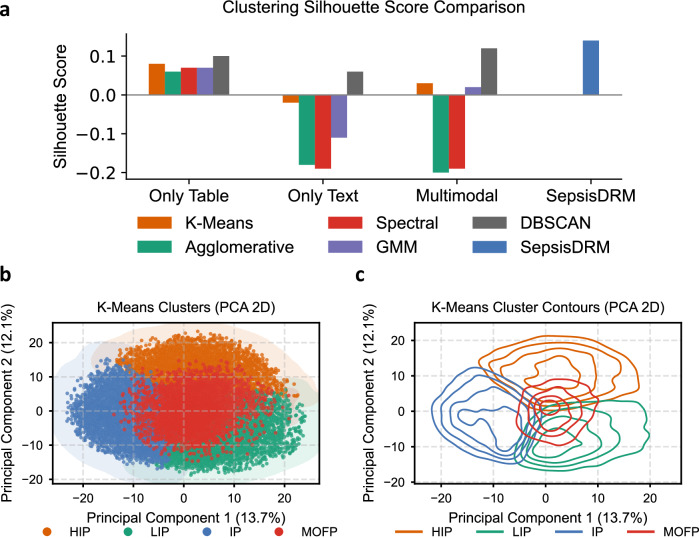
Clustering performance and visualization of sepsis phenotypes. **a** Silhouette score comparison across multiple clustering algorithms (*K*-Means, Agglomerative, Spectral, GMM, DBSCAN). **b** and **c** The scatter plot and contour plot of the clustering results for 4 clusters in a two-dimensional plane.

Unsupervised contrastive learning allows the model to directly extract high-quality representations from unlabeled sepsis data. Subsequently, clustering algorithms are applied to these representations to identify distinct sepsis phenotypes. After evaluating various clustering methods and the number of clusters, we selected *K*-means as the clustering algorithm. With the number of clusters set to four, this configuration yielded the clearest and most clinically relevant clustering results. We compared our proposed SepsisDRM against a variety of baseline algorithms across three input settings: tabular only, text only, and multimodal concatenation. As shown in Fig. 5a and Table 3, the baseline methods generally exhibited modest or even negative silhouette scores, indicating poor intra-cluster cohesion and inter-cluster separation. For example, text-only clustering yielded mostly negative silhouette values (e.g., −0.19 for Spectral), reflecting unstable or overlapping partitions, while the multimodal baselines did not substantially improve the separation (maximum silhouette of 0.12 for DBSCAN). In contrast, SepsisDRM achieved the highest silhouette score (0.14), suggesting more coherent and well-separated clusters. Beyond local cohesion, SepsisDRM substantially outperformed all baselines in the global Calinski–Harabasz score (2137.26 vs. ≤1059.09), as shown in Table 3, indicating that its clusters maximize between-class variance relative to within-class variance. Moreover, SepsisDRM obtained the lowest Davies-Bouldin score (1.97), as shown in Fig. 5a and Table 3, further confirming superior compactness and separability. Taken together, these results demonstrate that SepsisDRM yields more meaningful and discriminative structure in the latent space compared to both unimodal and multimodal baselines.

**Table 3:** Comparison between the proposed model (SepsisDRM + *K*-Means) and baseline methods on the clustering task

Category	Method	Silhouette score	Calinski–Harabasz score	Davies–Bouldin score
Only table	*K*-Means	0.08	1059.09	2.99
	Agglomerative	0.06	723.58	3.50
	Spectral	0.07	594.01	2.34
	GMM	0.07	612.58	4.08
	DBSCAN	0.10	468.19	2.54
Only text	*K*-Means	−0.02	78.40	11.69
	Agglomerative	−0.18	87.07	4.60
	Spectral	−0.19	44.90	2.37
	GMM	−0.11	62.96	8.69
	DBSCAN	0.06	350.62	3.16
Multimodal	*K*-Means	0.03	62.11	6.58
	Agglomerative	−0.20	69.87	4.54
	Spectral	−0.19	37.76	2.32
	GMM	0.02	58.47	10.23
	DBSCAN	0.12	560.82	2.27
Our model	SepsisDRM	**0.14**	**2137.26**	**1.97**

Reported metrics are Silhouette score, Calinski–Harabasz score, and Davies–Bouldin score. Bold values indicate the best performance across all methods, and underlined values denote the second-best performance.

To verify the robustness of the identified phenotypes, we performed a rigorous stability analysis. We evaluated the cluster stability using the adjusted Rand index (ARI) across 100 iterations. First, to assess sensitivity to initialization, we varied the random seed of the *K*-Means algorithm on the full dataset, yielding a mean ARI of 0.97 (±0.11), indicating that the clusters are invariant to initialization. Second, to assess robustness to data perturbations, we performed subsampling (using 80% of the dataset per iteration). The model achieved a mean ARI of 0.83 (±0.21), confirming that the identified phenotypes represent stable pathophysiological structures rather than artifacts of specific data samples.

We then conducted statistical analyses of patient characteristics and outcomes for these identified phenotypes. The scatter plot illustrates the distribution of data points across the two principal components, with distinct clusters represented by different colors, indicating the separation of groups (Fig. 5b). The contour plot visualizes the density and shape of each cluster’s distribution in the principal component space, using contour lines to highlight regions of higher density (Fig. 5c). This four-class model was determined to be the optimal solution, with the characteristics of each phenotype detailed in Table 4. For normally distributed continuous variables, data were presented as mean ± standard deviation (SD). For non-normally distributed continuous variables, data were presented as median with interquartile range (IQR). Categorical variables were expressed as frequencies and percentages.

**Table 4:** The characteristics of the four Phenotypes

Characteristic	Total	HIP	LIP	IP	MOFP	*P*
No. of patients (%)	19,526 (100.0%)	4478 (22.9%)	5612 (28.7%)	5179 (26.5%)	4257 (21.8%)	
*Sex, No. (%)*						<0.001
Female	7668 (39.3%)	1777 (39.7%)	2283 (40.7%)	2062 (39.8%)	1546 (36.3%)	
Male	11,858 (60.7%)	2701 (60.3%)	3329 (59.3%)	3117 (60.2%)	2711 (63.7%)	
Age, mean (SD), yr	67.00 (17.10)	67.70 (16.30)	65.40 (18.40)	69.30 (16.10)	65.50 (17.00)	<0.001
ICU Hosp. Days, day	3.43 (14.20)	2.74 (6.92)	2.87 (7.32)	4.41 (24.60)	3.71 (8.35)	<0.001
Hosp. Days, day	14.00 (26.10)	12.30 (12.50)	13.70 (14.50)	16.30 (44.80)	13.10 (15.00)	<0.001
*In-hospital mortality, No. (%)*						<0.001
No	17,652 (90.4%)	4052 (90.5%)	5256 (93.7%)	4694 (90.6%)	3650 (85.7%)	
Yes	1874 (9.6%)	426 (9.5%)	356 (6.3%)	485 (9.4%)	607 (14.3%)	
SOFA, mean (SD)	5.05 (2.84)	5.16 (2.89)	4.41 (2.34)	4.16 (1.99)	6.84 (3.39)	<0.001
Respiratory	2.13 (1.54)	2.05 (1.57)	1.93 (1.58)	2.60 (1.32)	1.92 (1.58)	<0.001
Cardiovascular	0.94 (1.18)	1.32 (1.25)	1.18 (1.18)	0.11 (0.41)	1.23 (1.23)	<0.001
Liver	0.46 (0.87)	0.54 (0.87)	0.28 (0.60)	0.32 (0.73)	0.78 (1.16)	<0.001
Coagulation	0.49 (1.09)	0.54 (1.12)	0.35 (0.93)	0.45 (1.04)	0.70 (1.25)	<0.001
Renal	0.85 (1.21)	0.58 (0.82)	0.45 (0.73)	0.50 (0.80)	2.10 (1.58)	<0.001
*Hypertension, No. (%)*						<0.001
No	9703 (49.7%)	2526 (56.4%)	2871 (51.2%)	2430 (46.9%)	1876 (44.1%)	
Yes	9823 (50.3%)	1952 (43.6%)	2741 (48.8%)	2749 (53.1%)	2381 (55.9%)	
*CAD, No. (%)*						<0.001
No	15,619 (80.0%)	3689 (82.4%)	4543 (81.0%)	4140 (79.9%)	3247 (76.3%)	
Yes	3907 (20.0%)	789 (17.6%)	1069 (19.0%)	1039 (20.1%)	1010 (23.7%)	
DM, No. (%)						<0.001
No	14,723 (75.4%)	3408 (76.1%)	4489 (80.0%)	3783 (73.0%)	3043 (71.5%)	
Yes	4803 (24.6%)	1070 (23.9%)	1123 (20.0%)	1396 (27.0%)	1214 (28.5%)	
*COPD, No. (%)*						<0.001
No	18,393 (94.2%)	4246 (94.8%)	5287 (94.2%)	4762 (91.9%)	4098 (96.3%)	
Yes	1133 (5.8%)	232 (5.2%)	325 (5.8%)	417 (8.1%)	159 (3.7%)	
*CKD, No. (%)*						<0.001
No	17,169 (87.9%)	4219 (94.2%)	5314 (94.7%)	4818 (93.0%)	2818 (66.2%)	
Yes	2357 (12.1%)	259 (5.8%)	298 (5.3%)	361 (7.0%)	1439 (33.8%)	
*CVD, No. (%)*						<0.001
No	13,889 (71.1%)	3378 (75.4%)	3777 (67.3%)	3483 (67.3%)	3251 (76.4%)	
Yes	5637 (28.9%)	1100 (24.6%)	1835 (32.7%)	1696 (32.7%)	1006 (23.6%)	
*Chronic liver dis., No. (%)*						<0.001
No	18,324 (93.8%)	4221 (94.3%)	5372 (95.7%)	5056 (97.6%)	3675 (86.3%)	
Yes	1202 (6.2%)	257 (5.7%)	240 (4.3%)	123 (2.4%)	582 (13.7%)	
*Hema. malignancy, No. (%)*						<0.001
No	18,730 (95.9%)	4227 (94.4%)	5201 (92.7%)	5132 (99.1%)	4170 (98.0%)	
Yes	796 (4.1%)	251 (5.6%)	411 (7.3%)	47 (0.9%)	87 (2.0%)	
*Tumor, No. (%)*						<0.001
No	17,374 (89.0%)	3910 (87.3%)	5201 (92.7%)	4450 (85.9%)	3813 (89.6%)	
Yes	2152 (11.0%)	568 (12.7%)	411 (7.3%)	729 (14.1%)	444 (10.4%)	
hsCRP, mg/L	66.8 [23.8, 137.0]	173.0 [127.0, 235.0]	27.6 [10.3, 58.0]	68.5 [31.3, 119.0]	50.0 [18.1, 104.0]	<0.001
APTT, s	36.0 [29.7, 41.8]	37.9 [31.5, 43.6]	34.3 [28.3, 39.5]	34.9 [28.9, 40.7]	38.0 [31.5, 45.0]	<0.001
TT, s	17.2 [16.1, 18.6]	16.9 [15.7, 18.1]	17.2 [16.1, 18.4]	17.1 [16.0, 18.3]	17.9 [16.6, 20.1]	<0.001
INR, R	1.13 [1.04, 1.26]	1.18 [1.10, 1.30]	1.08 [1.01, 1.17]	1.12 [1.04, 1.22]	1.19 [1.07, 1.42]	<0.001
PTA, %	78.3 [65.1, 91.0]	73.2 [62.0, 84.0]	86.3 [74.0, 97.0]	79.0 [68.0, 90.0]	72.0 [54.0, 87.0]	<0.001
PT, s	14.0 [12.8, 15.4]	14.5 [13.4, 15.9]	13.4 [12.3, 14.5]	13.9 [12.8, 15.0]	14.6 [13.2, 17.0]	<0.001
FIB, mg/L FEU	4.41 [3.19, 5.79]	5.50 [4.15, 6.88]	3.62 [2.76, 4.58]	4.95 [3.90, 6.22]	3.87 [2.56, 5.22]	<0.001
WBC, 10^9^/L	10.20 [6.65, 14.70]	10.60 [6.54, 15.50]	8.59 [5.32, 12.50]	11.80 [8.50, 16.40]	9.99 [6.57, 14.60]	<0.001
HCT, %	32.7 [26.8, 37.7]	32.4 [26.8, 37.0]	35.1 [29.3, 39.5]	32.3 [27.3, 36.8]	30.0 [24.0, 36.3]	<0.001
RDW, %	13.8 [12.9, 15.3]	13.7 [12.9, 15.2]	13.4 [12.8, 14.8]	13.8 [12.9, 15.4]	14.3 [13.2, 16.1]	<0.001
LYM, 10^9^/L	0.90 [0.57, 1.36]	0.76 [0.47, 1.15]	0.92 [0.60, 1.41]	1.05 [0.70, 1.52]	0.83 [0.52, 1.28]	<0.001
Hb, g/L	108.0 [87.0, 125.0]	108.0 [87.0, 124.0]	117.0 [96.0, 132.0]	105.0 [87.0, 122.0]	98.0 [77.0, 121.0]	<0.001
PLT, 10^9^/L	178 [114, 251]	137 [87, 187]	146 [98, 193]	295 [242, 364]	150 [92, 212]	<0.001
PDW, fL	15.8 [13.2, 16.3]	16.0 [14.6, 16.5]	15.9 [13.2, 16.4]	15.5 [11.6, 16.0]	16.0 [14.5, 16.4]	<0.001
NEUT, 10^9^/L	8.28 [4.88, 12.50]	8.77 [5.04, 13.30]	6.59 [3.52, 10.40]	9.82 [6.51, 14.10]	8.12 [4.88, 12.50]	<0.001
ALB, g/L	33.5 [29.3, 37.8]	31.7 [27.8, 35.6]	37.0 [33.2, 40.5]	32.9 [29.0, 36.8]	31.6 [27.4, 36.0]	<0.001
hs-cTnT, μg/L	0.03 [0.01, 0.07]	0.03 [0.01, 0.06]	0.02 [0.01, 0.04]	0.03 [0.01, 0.06]	0.06 [0.02, 0.18]	<0.001
ALT, U/L	22.0 [13.0, 44.0]	22.2 [14.0, 39.0]	19.0 [12.9, 32.0]	20.0 [12.0, 35.0]	45.0 [16.0, 160.0]	<0.001
AST, U/L	30.0 [20.0, 59.0]	31.0 [20.0, 52.0]	26.0 [18.0, 41.0]	27.0 [19.0, 45.0]	75.0 [24.0, 241.0]	<0.001
Cr, μmol/L	91.0 [66.0, 147.0]	89.0 [66.0, 125.0]	81.0 [64.0, 110.0]	79.0 [60.0, 114.0]	229.0 [101.0, 489.0]	<0.001
K+, mmol/L	3.93 [3.57, 4.34]	3.81 [3.47, 4.18]	3.90 [3.58, 4.23]	3.94 [3.57, 4.31]	4.19 [3.69, 4.79]	<0.001
Na+, mmol/L	138.0 [135.0, 141.0]	137.0 [134.0, 141.0]	139.0 [136.0, 142.0]	138.0 [134.0, 141.0]	138.0 [134.0, 142.0]	<0.001
Urea, μmol/L	6.86 [4.60, 11.70]	6.80 [4.70, 10.50]	5.74 [4.20, 8.19]	6.08 [4.20, 9.47]	14.30 [7.70, 23.90]	<0.001
PA, mg/L	121.0 [68.9, 182.0]	77.0 [44.0, 117.0]	186.0 [141.0, 233.0]	102.0 [60.0, 152.0]	111.0 [60.0, 178.0]	<0.001
DBIL, μmol/L	5.80 [3.60, 10.80]	7.60 [4.80, 14.00]	4.80 [3.20, 7.60]	5.00 [3.30, 8.50]	7.70 [3.60, 25.20]	<0.001
TBIL, μ>mol/L	12.50 [8.00, 21.90]	15.40 [10.00, 25.30]	11.50 [7.80, 18.10]	10.80 [7.15, 17.20]	14.40 [7.70, 38.80]	<0.001
TCO2, mmol/L	23.4 [20.4, 26.2]	23.1 [20.3, 25.8]	24.0 [21.6, 26.5]	24.2 [21.5, 27.1]	21.3 [17.5, 24.5]	<0.001
TC, mmol/L	3.59 [2.85, 4.42]	3.36 [2.63, 4.17]	3.83 [3.16, 4.67]	3.63 [2.88, 4.44]	3.45 [2.68, 4.31]	<0.001
LDL-C, mmol/L	2.13 [1.49, 2.83]	1.92 [1.27, 2.60]	2.32 [1.72, 3.01]	2.22 [1.60, 2.90]	1.97 [1.33, 2.70]	<0.001
non-LDL-C, mmol/L	2.68 [2.05, 3.42]	2.55 [1.91, 3.26]	2.79 [2.16, 3.52]	2.72 [2.08, 3.46]	2.64 [1.99, 3.41]	<0.001
HDL-C, mmol/L	0.86 [0.61, 1.13]	0.76 [0.49, 1.03]	1.00 [0.77, 1.27]	0.85 [0.62, 1.10]	0.77 [0.49, 1.03]	<0.001

Normally distributed variables are mean (SD); others are median [IQR]. *P* values: Chi-square/Fisher’s exact (categorical) or Kruskal–Wallis (continuous).

**Fig. 6:**
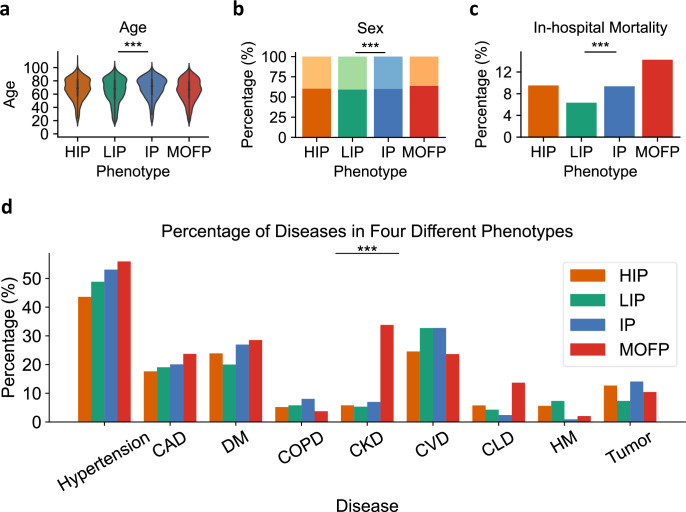
In-hospital mortality, disease incidence rate, and demographics in four phenotypes. **a** Distribution of age in four phenotypes. **b** Distribution of sex in four phenotypes. Darker bars represent the proportion of male patients, whereas lighter bars represent the proportion of female patients. **c** In-hospital mortality of four phenotypes. **d** Incidence rate of hypertension, coronary artery disease (CAD), diabetes mellitus (DM), chronic obstructive pulmonary disease (COPD), chronic kidney disease (CKD), cardiovascular disease (CVD), chronic liver disease (CLD), hematological malignancy (HM), and tumor in four phenotypes. Statistical analyses were performed using a Kruskal–Wallis *H* test, and *** means *P* ≤ 0.001. Pairwise adjusted *p*-values for comparisons of characteristics among the four phenotypes are shown in Table 5.

**Fig. 7:**
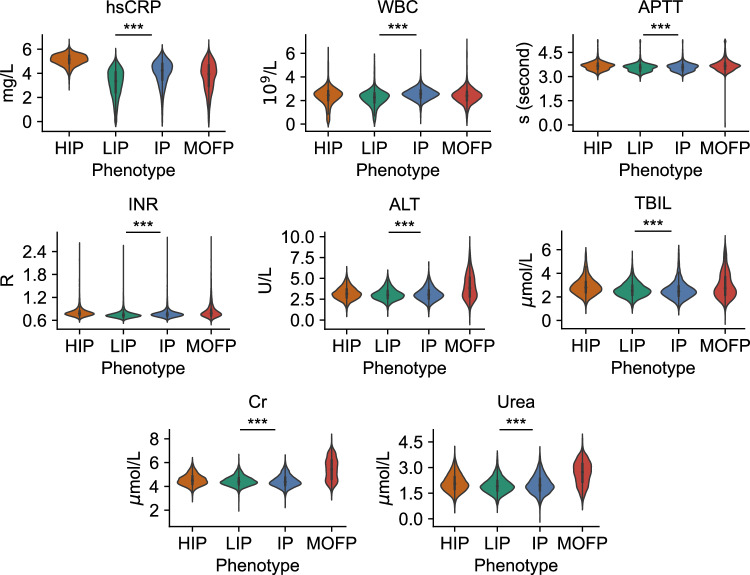
Violin plots of key laboratory test results in four phenotypes. Due to the significant variability in test results across patients, directly visualizing the data does not yield optimal results. Therefore, to better display the distributions, we applied a log transformation to the key blood test indices. Prior to this transformation, a constant of 1 was added to all values to avoid issues with the log transformation of zero values. The full names corresponding to the abbreviations for laboratory test results in the figure can be found in Supplementary Table 1. Statistical analyses were performed using a Kruskal–Wallis *H* test, and *** means *p* ≤ 0.001. Pairwise adjusted *p*-values for comparisons of characteristics among the four phenotypes are shown in Table 5.

**Fig. 8:**
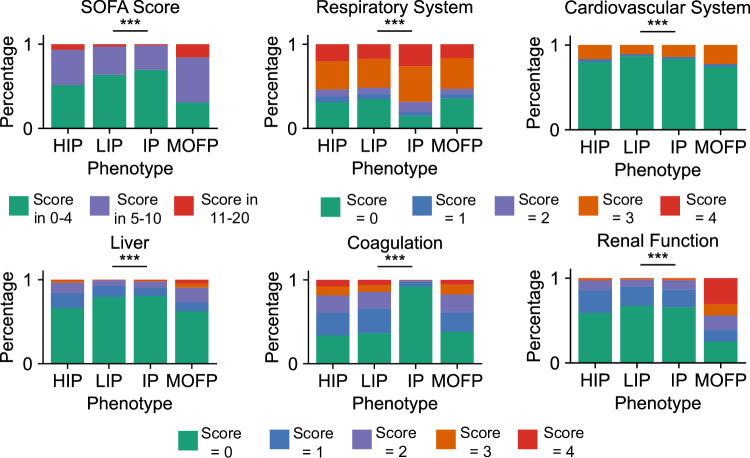
SOFA scores across four phenotypes. Distribution of SOFA scores in four phenotypes, with different colors representing the proportion of corresponding scores in that phenotype. Statistical analyses were performed using a Kruskal–Wallis *H* test, and *** means *P* ≤ 0.001. Pairwise adjusted *p*-values for comparisons of characteristics among the four phenotypes are shown in Table 5.

The distribution of age and sex across the four phenotypes is shown in Fig. 6a and b, indicating that demographic information is generally consistent across phenotypes. In-hospital mortality rates differed significantly across the four phenotypes (Chi-square test, *p* < 0.001), as visualized in Fig. 6c. Similarly, the incidence rates of nine comorbidities showed distinct patterns (Fig. 6d). The key laboratory test results across four phenotypes are shown in Fig. 7, and the remaining laboratory test results are shown in Supplementary Figs. 1–3. We performed significance testing for all variables to validate these observations. Continuous variables (e.g., age, laboratory indicators, SOFA scores) were analyzed using the Kruskal–Wallis *H* test, while categorical variables (e.g., mortality, sex, comorbidities) were analyzed using the Chi-square test (*χ*^2^), with the results presented in Figs. 6–8, as well as Table 4. Pairwise adjusted *p*-values for comparisons of characteristics among the four phenotypes (HIP, LIP, IP, and MOFP) are shown in Table 5.

**Table 5:** Pairwise adjusted *p*-values for comparisons of characteristics among the four phenotypes (HIP, LIP, IP, and MOFP)

Variable	HIP vs. IP	HIP vs. LIP	HIP vs. MOFP	IP vs. LIP	IP vs. MOFP	LIP vs. MOFP
Age	2.55 × 10^−6^	<1 × 10^−6^	<1 × 10^−6^	<1 × 10^−6^	0.152	<1 × 10^−6^
Sex	0.924	0.924	5.13 × 10^−3^	0.924	6.60 × 10^−5^	2.68 × 10^−3^
Mortality	<1 × 10^−6^	0.805	<1 × 10^−6^	<1 × 10^−6^	<1 × 10^−6^	<1 × 10^−6^
SOFA	<1 × 10^−6^	<1 × 10^−6^	<1 × 10^−6^	1.77 × 10^−4^	<1 × 10^−6^	<1 × 10^−6^
SOFA-Respiratory	1.96 × 10^−4^	<1 × 10^−6^	1.96 × 10^−4^	<1 × 10^−6^	0.796	<1 × 10^−6^
SOFA-Cardiovascular	<1 × 10^−6^	1.13 × 10^−5^	<1 × 10^−6^	1.13 × 10^−5^	<1 × 10^−6^	<1 × 10^−6^
SOFA-Liver	<1 × 10^−6^	<1 × 10^−6^	<1 × 10^−6^	0.832	<1 × 10^−6^	<1 × 10^−6^
SOFA-Coagulation	1.19 × 10^−5^	<1 × 10^−6^	1.79 × 10^−3^	<1 × 10^−6^	0.294	<1 × 10^−6^
SOFA-Renal	<1 × 10^−6^	<1 × 10^−6^	<1 × 10^−6^	0.018	<1 × 10^−6^	<1 × 10^−6^
Hypertension	<1 × 10^−6^	<1 × 10^−6^	<1 × 10^−6^	2.18 × 10^−5^	<1 × 10^−6^	5.84 × 10^−3^
CAD	0.149	8.33 × 10^−3^	< 1 × 10^−6^	0.189	< 1 × 10^−6^	3.83 × 10^−5^
DM	2.03 × 10^−5^	9.94 × 10^−4^	2.12 × 10^−6^	<1 × 10^−6^	<1 × 10^−6^	0.079
COPD	0.193	<1 × 10^−6^	7.73 × 10^−3^	2.09 × 10^−6^	4.53 × 10^−5^	<1 × 10^−6^
CKD	0.468	0.149	<1 × 10^−6^	0.025	<1 × 10^−6^	<1 × 10^−6^
CVD	<1 × 10^−6^	<1 × 10^−6^	0.672	0.955	<1 × 10^−6^	<1 × 10^−6^
Chronic liver disease	2.39 × 10^−3^	< 1 × 10^−6^	< 1 × 10^−6^	8.06 × 10^−5^	< 1 × 10^−6^	< 1 × 10^−6^
Hematological malignancy	2.89 × 10^−5^	<1 × 10^−6^	<1 × 10^−6^	<1 × 10^−6^	<1 × 10^−6^	5.48 × 10^−3^
Tumor	<1 × 10^−6^	0.029	1.54 × 10^−3^	< 1 × 10^−6^	3.18 × 10^−6^	<1 × 10^−6^
hsCRP	<1 × 10^−6^	< 1 × 10^−6^	<1 × 10^−6^	<1 × 10^−6^	<1 × 10^−6^	<1 × 10^−6^
APTT	<1 × 10^−6^	<1 × 10^−6^	0.134	1.08 × 10^−6^	<1 × 10^−6^	<1 × 10^−6^
TT	<1 × 10^−6^	<1 × 10^−6^	<1 × 10^−6^	6.89 × 10^−3^	<1 × 10^−6^	<1 × 10^−6^
INR	<1 × 10^−6^	<1 × 10^−6^	0.207	<1 × 10^−6^	<1 × 10^−6^	<1 × 10^−6^
PTA	<1 × 10^−6^	<1 × 10^−6^	0.084	<1 × 10^−6^	<1 × 10^−6^	<1 × 10^−6^
PT	<1 × 10^−6^	<1 × 10^−6^	0.012	<1 × 10^−6^	<1 × 10^−6^	<1 × 10^−6^
FIB	<1 × 10^−6^	<1 × 10^−6^	<1 × 10^−6^	<1 × 10^−6^	<1 × 10^−6^	<1 × 10^−6^
WBC	<1 × 10^−6^	<1 × 10^−6^	0.032	<1 × 10^−6^	<1 × 10^−6^	<1 × 10^−6^
HCT	<1 × 10^−6^	0.486	<1 × 10^−6^	<1 × 10^−6^	<1 × 10^−6^	<1 × 10^−6^
RDW	<1 × 10^−6^	2.51 × 10^−3^	<1 × 10^−6^	<1 × 10^−6^	<1 × 10^−6^	<1 × 10^−6^
LYM	<1 × 10^−6^	<1 × 10^−6^	<1 × 10^−6^	<1 × 10^−6^	<1 × 10^−6^	<1 × 10^−6^
Hb	<1 × 10^−6^	0.016	<1 × 10^−6^	<1 × 10^−6^	<1 × 10^−6^	<1 × 10^−6^
PLT	2.47 × 10^−4^	<1 × 10^−6^	<1 × 10^−6^	<1 × 10^−6^	<1 × 10^−6^	<1 × 10^−6^
PDW	<1 × 10^−6^	<1 × 10^−6^	0.229	<1 × 10^−6^	<1 × 10^−6^	<1 × 10^−6^
NEUT	<1 × 10^−6^	<1 × 10^−6^	0.011	<1 × 10^−6^	<1 × 10^−6^	<1 × 10^−6^
ALB	<1 × 10^−6^	<1 × 10^−6^	0.384	<1 × 10^−6^	<1 × 10^−6^	<1 × 10^−6^
hs-cTnT	<1 × 10^−6^	0.739	<1 × 10^−6^	<1 × 10^−6^	<1 × 10^−6^	<1 × 10^−6^
ALT	<1 × 10^−6^	<1 × 10^−6^	<1 × 10^−6^	0.046	<1 × 10^−6^	<1 × 10^−6^
AST	<1 × 10^−6^	<1 × 10^−6^	<1 × 10^−6^	1.53 × 10^−5^	<1 × 10^−6^	<1 × 10^−6^
Cr	<1 × 10^−6^	<1 × 10^−6^	<1 × 10^−6^	0.044	<1 × 10^−6^	<1 × 10^−6^
K+	<1 × 10^−6^	<1 × 10^−6^	<1 × 10^−6^	5.24 × 10^−3^	<1 × 10^−6^	<1 × 10^−6^
Na+	<1 × 10^−6^	1.07 × 10^−4^	< 1 × 10^−6^	< 1 × 10^−6^	< 1 × 10^−6^	5.28 × 10^−4^
Urea	<1 × 10^−6^	<1 × 10^−6^	<1 × 10^−6^	<1 × 10^−6^	<1 × 10^−6^	<1 × 10^−6^
PA	<1 × 10^−6^	<1 × 10^−6^	<1 × 10^−6^	<1 × 10^−6^	<1 × 10^−6^	<1 × 10^−6^
DBIL	<1 × 10^−6^	<1 × 10^−6^	1.65× 10^−3^	1.34 × 10^−5^	<1 × 10^−6^	<1 × 10^−6^
TBIL	<1 × 10^−6^	<1 × 10^−6^	1.99 × 10^−4^	1.99 × 10^−4^	<1 × 10^−6^	<1 × 10^−6^
TCO_2_	<1 × 10^−6^	<1 × 10^−6^	<1 × 10^−6^	0.022	<1 × 10^−6^	<1 × 10^−6^
TC	<1 × 10^−6^	<1 × 10^−6^	4.69 × 10^−5^	<1 × 10^−6^	<1 × 10^−6^	<1 × 10^−6^
LDL-C	<1 × 10^−6^	<1 × 10^−6^	6.99 × 10^−4^	<1 × 10^−6^	<1 × 10^−6^	<1 × 10^−6^
non-HDL-C	<1 × 10^−6^	< 1 × 10^−6^	2.08 × 10^−5^	2.81 × 10^−4^	< 1 × 10^−6^	2.81 × 10^−4^
HDL-C	<1 × 10^−6^	<1 × 10^−6^	0.723	<1 × 10^−6^	<1 × 10^−6^	<1 × 10^−6^

Values are adjusted for multiple testing using the Holm method.

Patients in Group I, which emerged from the unsupervised clustering, were characterized post hoc by the highest levels of inflammatory markers such as hsCRP, and had a moderate in-hospital mortality rate of 9.51%. This group was therefore designated the “High inflammation phenotype (HIP)”. In contrast, Group II showed the lowest levels of inflammatory markers (hsCRP), minimal organ dysfunction, and the lowest prevalence of comorbidities such as diabetes and chronic kidney disease. Consequently, this group had the lowest in-hospital mortality rate of 6.34% and was designated the “Low inflammation phenotype (LIP)”. Group III exhibited intermediate characteristics across the board, including age, inflammatory markers (hsCRP, WBC), coagulation parameters (APTT, INR), and comorbidities. Since these variables all fell into the middle range compared to the other phenotypes, this group was labeled the “Intermediate phenotype (IP)”, with an in-hospital mortality rate of 9.36%. Finally, Group IV displayed the most severe clinical profile, with the highest levels of inflammation (hsCRP, WBC), liver function markers (ALT, TBIL), renal function markers (Cr, urea), and coagulation abnormalities. This group also had the highest burden of comorbidities, such as hypertension, coronary artery disease, diabetes mellitus, chronic kidney disease, and chronic liver disease, as shown in Fig. 6d, resulting in the highest in-hospital mortality rate of 14.3%. Accordingly, this group was defined as the “Multiple Organ Failure Phenotype (MOFP)”. The analysis of SOFA scores across phenotypes (Fig. 8) further confirmed these observations: the MOFP phenotype contained the highest number of patients with a SOFA Liver Score and SOFA Renal Function Score of 4, and had the largest proportion of patients with overall SOFA scores between 11 and 20, consistent with the greatest severity among the four phenotypes.

### Statistical analysis of text data for patients with four phenotypes

In analyzing the textual data of the patients, we began by calculating the Term Significance (TS) for each medical term (details in the section “Methods of data analysis”). This metric allowed us to assess the relative importance of each term in the context of sepsis phenotypes. By calculating TS, we could identify which medical terms were more prominent in the textual data associated with specific phenotypes. This was particularly useful for distinguishing between the four sepsis phenotypes that we had identified, namely the high inflammation phenotype (HIP), low inflammation phenotype (LIP), intermediate phenotype (IP), and multiple organ failure phenotype (MOFP).

**Fig. 9:**
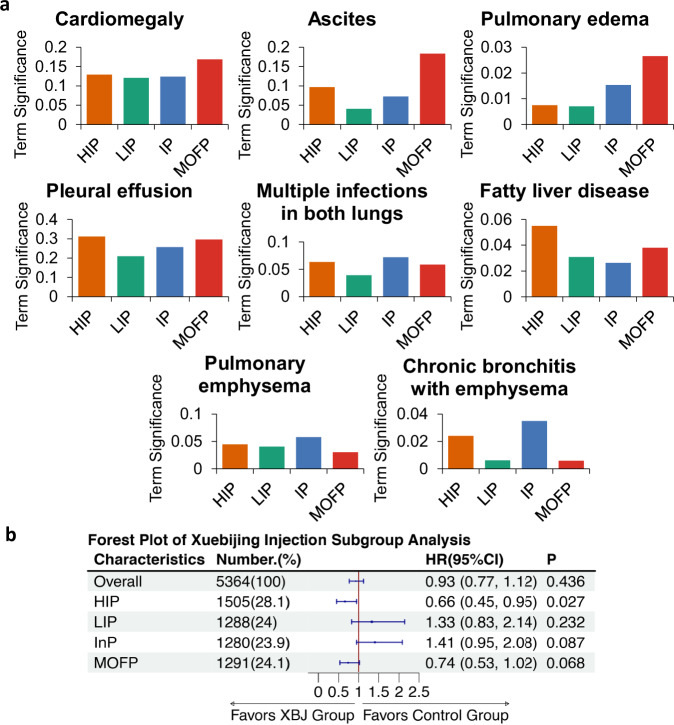
Term frequencies in textual data and drug intervention effects across phenotypes. **a** Frequencies of specific terms occurring in the textual data of patients with different phenotypes. **b** Forest plot of Xuebijing injection subgroup analysis.

Once the TS values were computed, we conducted a detailed analysis of the differences in specific medical terminology among these four phenotypes, focusing on the terms that occurred most frequently in each phenotype. This analysis aimed to uncover how the clinical representations of each sepsis phenotype were reflected in the patients’ medical records and documentation, thereby supporting the clinical relevance of the phenotypic distinctions. These analysis results are visually summarized in Fig. 9a, which illustrates the frequency distribution of key medical terms across the four phenotypes. The specific values of the TS of terms can be found in Supplementary Fig. 4, Supplementary Tables 2 and 3.

In the MOFP group, several terms exhibited notably high frequencies. These included “Cardiomegaly”, “Ascites”, and “Pulmonary edema”. These terms reflect the multi-system involvement and severe organ dysfunction that characterizes the MOFP phenotype, consistent with its name and the known clinical features of multiple organ failure. Notably, when we compared the MOFP group to the LIP group, we found that these same terms had much lower frequencies in the LIP phenotype, which is expected given that LIP is characterized by less severe inflammation and organ involvement.

The HIP showed a markedly different profile in terms of terminology frequency. The terms that appeared most frequently in this phenotype were “Pleural effusion”, “Multiple infections in both lungs”, and “Fatty liver disease”. These terms highlight the strong association between the HIP phenotype and severe, widespread inflammation, particularly within the lungs and respiratory system, as well as evidence of systemic inflammatory processes affecting the liver and cardiovascular system. The frequent mention of pulmonary infections further underscores the inflammation-centric nature of this phenotype.

In the case of the IP, certain terms also appeared at high frequencies, further distinguishing this group from the others. Among the most frequently occurring terms were “Multiple infections in both lungs”, “Pulmonary emphysema”, and “Chronic bronchitis with emphysema”. The elevated frequency of these terms is consistent with the respiratory and infection-related characteristics of the IP phenotype, which is predominantly marked by severe pulmonary infections and associated chronic respiratory conditions.

The results from the textual data analysis align closely with those obtained from the analysis of the tabular data, demonstrating consistency across different data modalities. This congruence provides additional validation for our phenotypic classification and the terminology used to differentiate the four groups.

### Baseline characteristics and impact of medicine treatment on four phenotypes

We conducted an exploratory study on the medications used for different phenotypes and found that Xuebijing injection (one of the most widely used adjunctive therapies for sepsis in China^27,28^) exhibits varying efficacy in different subgroups of sepsis patients. The baseline characteristics of patients prior to the study on Xuebijing treatment are detailed in Table 6, which presents results before propensity score matching (PSM). Compared to the control group, patients receiving Xuebijing had higher SOFA scores, older ages, and inconsistent comorbidities. After PSM, 2682 patients who did not receive Xuebijing were matched with 2682 patients who did. The baseline characteristics of both groups were reassessed following matching. Notably, there were no significant differences between the groups in baseline characteristics, except for the presence of chronic kidney disease, as shown in the matched results in Table 6. We conducted univariate logistic regression analysis to identify risk factors for in-hospital mortality. Factors such as age, SOFA score, sex, presence of ischemic heart disease (CAD), and cerebrovascular disease (CVD) were associated with in-hospital mortality, as detailed in Table 4. In the multivariate logistic regression analysis, age, sex, presence of diabetes, cerebrovascular disease, solid tumors, and the use of therapies including Albumin and human immunoglobulin, along with SOFA scores, were also found to be associated with in-hospital mortality, as indicated in Table 6. Additionally, we performed a subgroup analysis of phenotypes in relation to Xuebijing treatment and in-hospital mortality, with results depicted in Fig. 9b. No differences were observed in the overall sepsis population or in the Low Inflammation Phenotype (LIP), Intermediate Phenotype (IP), and Multiple Organ Failure Phenotype (MOFP) groups. However, a significant difference was noted in the High Inflammation Phenotype (HIP), with an odds ratio (OR) of 0.66 and a 95% confidence interval (CI) of (0.45, 0.95).

**Table 6:** The baseline characteristics of patients prior to the study on Xuebijing treatment before PSM and after PSM

Variable	Before PSM					
	Level	Total	Control group	Treatment group (XBJ)	*P*	SMD
No.		19,526	16,844	2682		
Age (mean (SD))		66.96 (17.11)	66.73 (17.17)	68.38 (16.64)	<0.001	0.097
Sex (%)	Female	7668 (39.3%)	6590 (39.1%)	1078 (40.2%)	0.302	0.022
	Male	11,858 (60.7%)	10,254 (60.9%)	1604 (59.8%)		
Hypertension, No. (%)	No	9703 (49.7%)	8383 (49.8%)	1320 (49.2%)	0.610	0.011
	Yes	9823 (50.3%)	8461 (50.2%)	1362 (50.8%)		
CAD, No. (%)	No	15,619 (80.0%)	13,473 (80.0%)	2146 (80.0%)	0.994	0.001
	Yes	3907 (20.0%)	3371 (20.0%)	536 (20.0%)		
DM, No. (%)	No	14,723 (75.4%)	12,804 (76.0%)	1919 (71.6%)	<0.001	0.102
	Yes	4803 (24.6%)	4040 (24.0%)	763 (28.4%)		
COPD, No. (%)	No	18,393 (94.2%)	15,865 (94.2%)	2528 (94.3%)	0.920	0.003
	Yes	1133 (5.8%)	979 (5.8%)	154 (5.7%)		
CKD, No. (%)	No	17,169 (87.9%)	14,803 (87.9%)	2366 (88.2%)	0.644	0.010
	Yes	2357 (12.1%)	2041 (12.1%)	316 (11.8%)		
CVD, No. (%)	No	13,889 (71.1%)	11,979 (71.1%)	1910 (71.2%)	0.935	0.002
	Yes	5637 (28.9%)	4865 (28.9%)	772 (28.8%)		
Chronic liver disease, No. (%)	No	18,324 (93.8%)	15,763 (93.6%)	2561 (95.5%)	<0.001	0.084
	Yes	1202 (6.2%)	1081 (6.4%)	121 (4.5%)		
Hematologic malignancy, No. (%)	No	18,730 (95.9%)	16,106 (95.6%)	2624 (97.8%)	<0.001	0.125
	Yes	796 (4.1%)	738 (4.4%)	58 (2.2%)		
Tumor, No. (%)	No	17,374 (89.0%)	14,917 (88.6%)	2457 (91.6%)	<0.001	0.102
	Yes	2152 (11.0%)	1927 (11.4%)	225 (8.4%)		
SOFA score (SD)		5.05 (2.84)	4.89 (2.73)	6.04 (3.30)	<0.001	0.378
**Variable**	**After PSM**					
	**Level**	**Total**	**Control group**	**Treatment group (XBJ)**	***P***	**SMD**
No.		5364	2682	2682		
Age (mean (SD))		68.44 (16.63)	68.50 (16.63)	68.38 (16.64)	0.783	0.008
Sex (%)	Female	2191 (40.8%)	1113 (41.5%)	1078 (40.2%)	0.345	0.027
	Male	3173 (59.2%)	1569 (58.5%)	1604 (59.8%)		
Hypertension, No. (%)	No	2607 (48.6%)	1287 (48.0%)	1320 (49.2%)	0.382	0.025
	Yes	2757 (51.4%)	1395 (52.0%)	1362 (50.8%)		
CAD, No. (%)	No	4309 (80.3%)	2163 (80.6%)	2146 (80.0%)	0.583	0.016
	Yes	1055 (19.7%)	519 (19.4%)	536 (20.0%)		
DM, No. (%)	No	3863 (72.0%)	1944 (72.5%)	1919 (71.6%)	0.465	0.021
	Yes	1501 (28.0%)	738 (27.5%)	763 (28.4%)		
COPD, No. (%)	No	5085 (94.8%)	2557 (95.3%)	2528 (94.3%)	0.085	0.049
	Yes	279 (5.2%)	125 (4.7%)	154 (5.7%)		
CKD, No. (%)	No	4778 (89.1%)	2412 (89.9%)	2366 (88.2%)	0.049	0.055
	Yes	586 (10.9%)	270 (10.1%)	316 (11.8%)		
CVD, No. (%)	No	3848 (71.7%)	1938 (72.3%)	1910 (71.2%)	0.413	0.023
	Yes	1516 (28.3%)	744 (27.7%)	772 (28.8%)		
Chronic liver disease, No. (%)	No	5150 (96.0%)	2589 (96.5%)	2561 (95.5%)	0.060	0.053
	Yes	214 (4.0%)	93 (3.5%)	121 (4.5%)		
Hematologic malignancy, No. (%)	No	5247 (97.8%)	2623 (97.8%)	2624 (97.8%)	1.000	0.003
	Yes	117 (2.2%)	59 (2.2%)	58 (2.2%)		
Tumor, No. (%)	No	4920 (91.7%)	2463 (91.8%)	2457 (91.6%)	0.804	0.008
	Yes	444 (8.3%)	219 (8.2%)	225 (8.4%)		
SOFA score (SD)		6.00 (3.34)	5.95 (3.38)	6.04 (3.30)	0.364	0.025

### Few-shot classification with task-specific supervised learning

Based on SepsisDRM, we only need a small amount of labeled data to predict the outcomes of sepsis patients with high accuracy. Specifically, we appended a three-layer multi-layer perceptron (MLP) as a classifier to the pre-trained SepsisDRM model. During training, the SepsisDRM parameters were frozen, and only the parameters of the MLP classifier were updated, allowing for a significantly faster training process. This binary classification task involved predicting whether the patient’s 28-day outcome would be survival or mortality. Model performance was evaluated using the area under the receiver operating characteristic curve (AUC), the area under the precision-recall curve (AUPRC), F1 Score, Balanced Accuracy, Matthews correlation coefficient (MCC), Specificity (true negative rate (TNR)), Sensitivity (Recall/true positive rate (TPR)), positive predictive value (PPV/Precision), negative predictive value (NPV), and out-of-fold (OOF) confusion matrix. To ensure robustness, we adopted five-fold cross-validation and present results as mean $[\min ,\max ]$ across folds. The Out-of-Fold (OOF) confusion matrices are presented as [[TN, FN], [FP, TP]] (true negative, false negative, false positive, true positive), aggregating per-fold predictions on held-out data across 5-fold cross-validation to approximate generalization performance. For transparency regarding class imbalance, the evaluation cohorts comprised GDHCM retrospective (*n* = 161, positive number = 18, negative number = 143), GDHCM prospective (*n* = 116, positive number = 18, negative number = 98), and SYSMH external (*n* = 292, positive number = 63, negative number = 229).

**Fig. 10:**
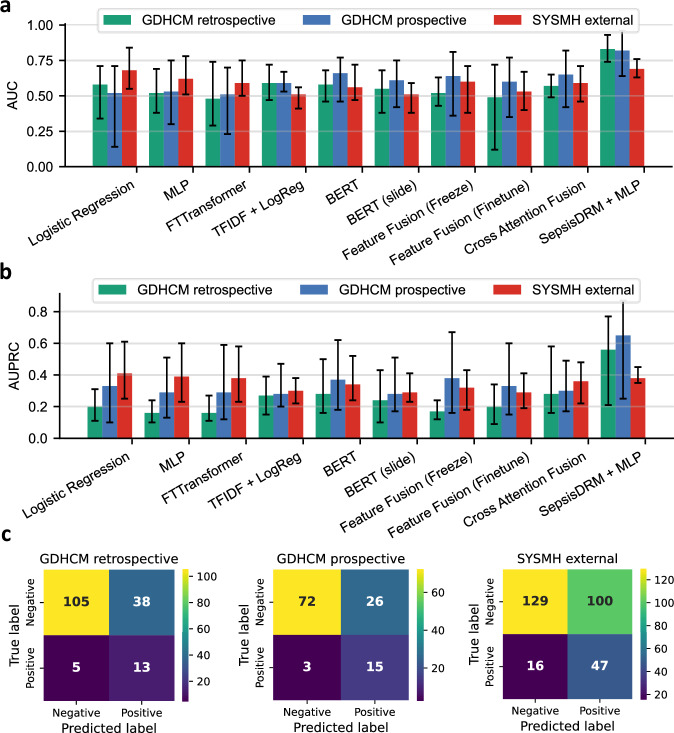
Performance comparison and classification results of SepsisDRM. **a** and **b** Comparison of SepsisDRM against baseline models in terms of area under the ROC curve (AUC) and area under the precision–recall curve (AUPRC), respectively. **c** Confusion matrix of SepsisDRM, illustrating the distribution of true/false positives and true/false negatives in classification outcomes. The confusion matrix is Out-of-Fold (OOF), aggregating per-fold predictions on held-out data across 5-fold cross-validation and reflecting TP/FP/TN/FN counts from out-of-sample evaluations to approximate generalization performance.

**Fig. 11:**
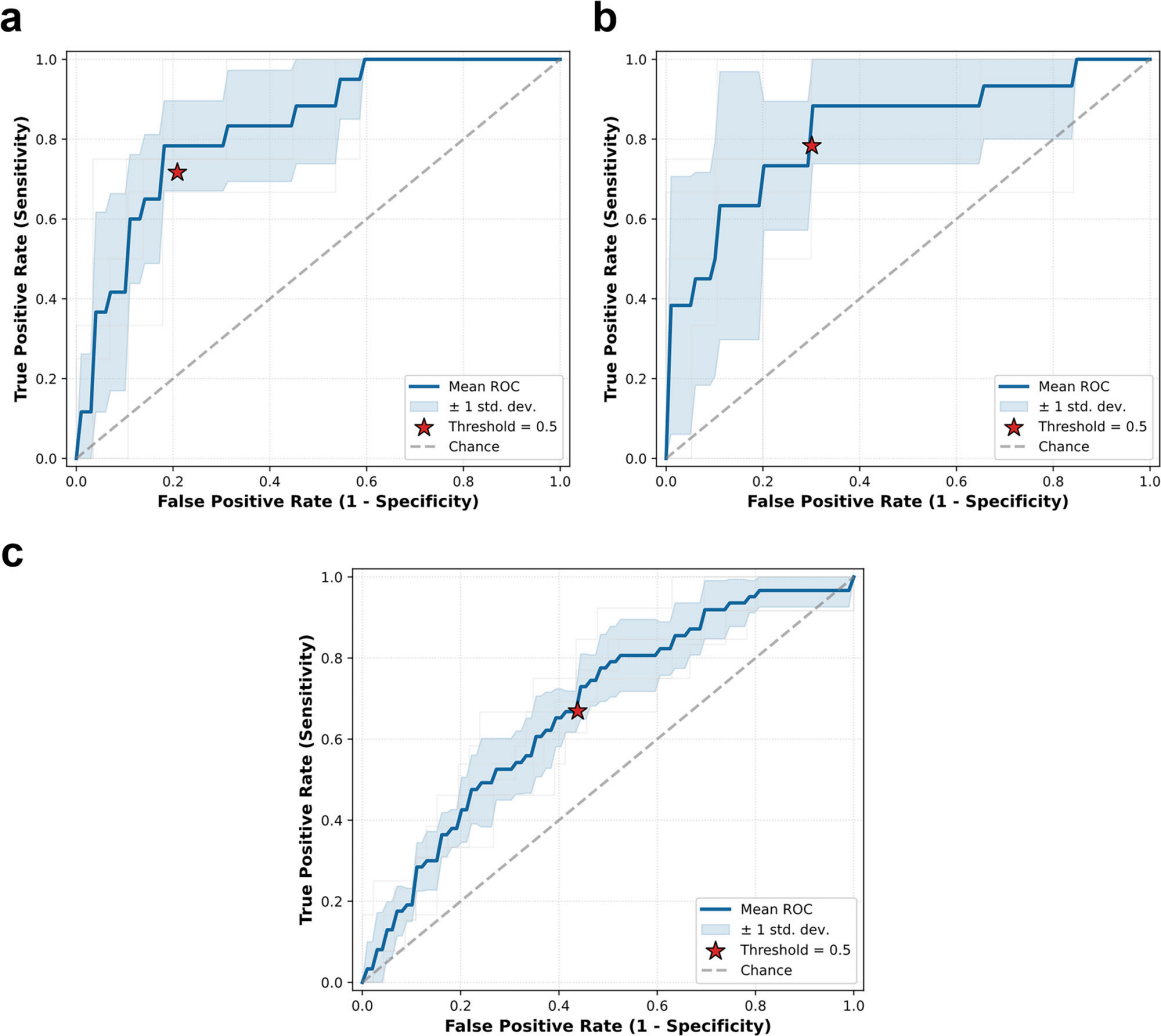
Empirical validation of the 0.5 decision threshold across three datasets. The receiver operating characteristic (ROC) curves are plotted for the test sets of **a** the GDHCM retrospective cohort, **b** the GDHCM prospective cohort, and **c** the SYSMH external validation cohort. The blue curves represent the mean ROC across 5-fold cross-validation, with shaded areas indicating the standard deviation. The red star (⋆) explicitly marks the operating point corresponding to the default decision threshold of 0.5. In all datasets, the 0.5 point is located near the top-left corner (optimal trade-off between Sensitivity and 1-Specificity), confirming the validity of using 0.5 as the decision boundary.

SepsisDRM demonstrated consistently strong performance across all datasets when directly compared with a broad panel of baseline models, as shown in Table 7. On the GDHCM retrospective dataset (*n* = 161, 18/143), SepsisDRM achieved an AUC of 0.83, [0.74, 0.93] and an AUPRC of 0.56, [0.21, 0.77], clearly surpassing all table-only, text-only, and multimodal baselines (best baseline AUC = 0.59, AUPRC = 0.28), as shown in Fig. 10a, b. Beyond threshold-free discrimination, SepsisDRM also showed superior threshold-dependent outcomes, attaining F1 = 0.37 vs. ≤0.29 for baselines, Balanced Accuracy = 0.73 vs. ≤0.61, and MCC = 0.33 vs. ≤0.18, while simultaneously achieving Specificity = 0.73, Sensitivity = 0.72, PPV = 0.30, and NPV = 0.96, as shown in Table 7. On the GDHCM prospective dataset (*n* = 116, 18/98), SepsisDRM reached AUC = 0.82, [0.64, 0.96] and AUPRC = 0.65, [0.25, 0.87], again outperforming all unimodal and multimodal baselines (best baseline AUC = 0.66, AUPRC = 0.37), as shown in Fig. 10a, b. It further improved clinically meaningful measures, including F1 = 0.54 vs. ≤0.35, Balanced Accuracy = 0.79 vs. ≤0.64, and MCC = 0.48 vs. ≤0.20, together with markedly higher Sensitivity (0.85) and NPV (0.97), as shown in Table 7. On the SYSMH external validation dataset (*n* = 292, 63/229), SepsisDRM maintained good generalizability with AUC = 0.69, [0.63, 0.76] and AUPRC = 0.38, [0.35, 0.45], competitive with the best baseline (logistic regression, AUC = 0.68, AUPRC = 0.41), as shown in Fig. 10a, b. This relatively weaker advantage on the external dataset can be largely explained by domain shift: differences in patient populations, clinical practice, and documentation style between hospitals introduce substantial distributional changes. Since our model was pre-trained exclusively on unlabeled data from GDHCM without any adaptation to SYSMH, metrics that are more sensitive to prevalence and thresholding, such as AUPRC, are particularly affected. While its AUPRC advantage narrowed under domain shift, SepsisDRM continued to deliver stronger F1 (0.47 vs. ≤0.41), Balanced Accuracy (0.65 vs. ≤0.63), and MCC (0.28 vs. ≤0.22), supported by higher Sensitivity (0.74) and NPV (0.91), as shown in Table 7. In addition to reporting the above metrics, we also present the out-of-fold (OOF) confusion matrices of SepsisDRM on the three datasets, as shown in Fig. 10c, in order to provide a more detailed view of its classification errors and predictive balance. Furthermore, to explicitly validate the decision threshold, we analyzed the ROC curves with the 0.5 operating point marked, as shown in Fig. 11. Across all three datasets, the 0.5 threshold (indicated by the red star) consistently lies near the optimal trade-off point (the top-left corner), confirming that the decision boundary learned via Focal Loss is well-aligned and that 0.5 is an empirically valid threshold for this task. Taken together, these results demonstrate that SepsisDRM consistently outperforms diverse baselines across three different datasets. Its advantage is particularly pronounced in internal and prospective datasets, while the stable AUC and robust threshold-dependent performance under external domain shift highlight the generalizability of the learned representations.

**Table 7:** Comparison between the proposed model (SepsisDRM + MLP) and baseline methods on the GDHCM retrospective dataset, GDHCM prospective dataset, and SYSMH external validation dataset

Dataset	Category	Method	AUC	AUPRC	F1	Balanced_Accuracy	MCC	Specificity	Sensitivity	PPV	NPV	Confusion matrix
GDHCM retrospective	Only Table	Logistic regression	0.58 [0.34, 0.71]	0.20 [0.11, 0.31]	0.19 [0.00, 0.40]	0.52 [0.34, 0.68]	0.04 [−0.23, 0.31]	0.71 [0.54, 0.86]	0.33 [0.00, 0.67]	0.14 [0.00, 0.33]	0.90 [0.83, 0.94]	[[102, 41], [12, 6]]
		MLP	0.52 [0.38, 0.69]	0.16 [0.10, 0.24]	0.20 [0.13, 0.31]	0.54 [0.47, 0.63]	0.05 [−0.04, 0.19]	0.62 [0.52, 0.76]	0.45 [0.25, 0.67]	0.13 [0.08, 0.22]	0.90 [0.87, 0.94]	[[89, 54], [10, 8]]
		FTTransformer	0.48 [0.29, 0.74]	0.16 [0.11, 0.27]	0.06 [0.00, 0.19]	0.42 [0.34, 0.47]	−0.12 [−0.23, −0.07]	0.63 [0.17, 0.93]	0.22 [0.00, 0.75]	0.03 [0.00, 0.11]	0.86 [0.83, 0.90]	[[90, 53], [14, 4]]
	Only Text	TFIDF + LogReg	0.59 [0.47,−0.72]	0.27 [0.15, 0.39]	0.19 [0.11, 0.29]	0.52 [0.42, 0.61]	0.04 [−0.10, 0.21]	0.70 [0.50, 0.93]	0.33 [0.25, 0.50]	0.16 [0.07, 0.33]	0.89 [0.87, 0.91]	[[100, 43], [12, 6]]
		BERT	0.58 [0.46, 0.68]	0.28 [0.16, 0.50]	0.29 [0.22, 0.40]	0.61 [0.56, 0.65]	0.18 [0.08, 0.36]	0.69 [0.52, 0.96]	0.53 [0.33, 0.75]	0.23 [0.15, 0.50]	0.93 [0.90, 0.94]	[[98, 45], [8, 10]]
		BERT (slide)	0.55 [0.38, 0.68]	0.24 [0.10, 0.43]	0.23 [0.12, 0.44]	0.55 [0.43, 0.70]	0.08 [−0.08, 0.36]	0.66 [0.45, 0.90]	0.43 [0.25, 0.75]	0.17 [0.07, 0.40]	0.90 [0.87, 0.93]	[[94, 49], [10, 8]]
	Multimodal	Feature fusion (freeze)	0.52 [0.43, 0.63]	0.17 [0.12, 0.24]	0.12 [0.00, 0.25]	0.50 [0.43, 0.58]	0.00 [−0.11, 0.11]	0.69 [0.41, 1.00]	0.32 [0.00, 0.75]	0.08 [0.00, 0.15]	0.90 [0.88, 0.92]	[[99, 44], [12, 6]]
		Feature fusion (finetune)	0.49 [0.12, 0.72]	0.20 [0.09, 0.34]	0.13 [0.00, 0.23]	0.51 [0.48, 0.55]	0.01 [−0.06, 0.07]	0.54 [0.00, 1.00]	0.48 [0.00, 1.00]	0.07 [0.00, 0.14]	0.72 [0.00, 0.92]	[[77, 66], [9, 9]]
		Cross attention fusion	0.57 [0.49, 0.65]	0.28 [0.16, 0.58]	0.24 [0.17, 0.33]	0.59 [0.49, 0.72]	0.13 [−0.01, 0.30]	0.57 [0.32, 0.93]	0.60 [0.25, 1.00]	0.18 [0.10, 0.33]	0.93 [0.90, 1.00]	[[82, 61], [7, 11]]
	Our Model	SepsisDRM + MLP	**0.83 [0.74, 0.93]**	**0.56 [0.21, 0.77]**	**0.37 [0.33, 0.50]**	**0.73 [0.61, 0.86]**	**0.33 [0.27, 0.49]**	**0.73 [0.48, 0.97]**	**0.72 [0.25, 1.00]**	**0.30 [0.21, 0.50]**	**0.96 [0.90, 1.00]**	**[[105, 38], [5, 13]]**
GDHCM prospective	Only Table	Logistic regression	0.52 [0.14, 0.71]	0.33 [0.10, 0.60]	0.20 [0.00, 0.31]	0.47 [0.21, 0.62]	−0.04 [−0.40, 0.17]	0.61 [0.42, 0.80]	0.33 [0.00, 0.67]	0.15 [0.00, 0.22]	0.83 [0.73, 0.92]	[[60, 38], [12, 6]]
		MLP	0.53 [0.30, 0.75]	0.29 [0.13, 0.51]	0.24 [0.14, 0.33]	0.51 [0.40, 0.65]	0.02 [−0.13, 0.21]	0.57 [0.40, 0.80]	0.45 [0.25, 0.67]	0.17 [0.09, 0.22]	0.85 [0.80, 0.92]	[[56, 42], [10, 8]]
		FT-Transformer	0.51 [0.23, 0.70]	0.29 [0.12, 0.59]	0.29 [0.00, 0.50]	0.57 [0.32, 0.82]	0.13 [−0.27, 0.44]	0.69 [0.40, 0.90]	0.45 [0.00, 1.00]	0.26 [0.00, 0.50]	0.87 [0.80, 1.00]	[[68, 30], [10, 8]]
	Only Text	TFIDF + LogReg	0.59 [0.53, 0.67]	0.28 [0.20, 0.47]	0.10 [0.00, 0.29]	0.48 [0.39, 0.59]	−0.06 [−0.19, 0.16]	**0.84 [0.75, 0.90]**	0.12 [0.00, 0.33]	0.08 [0.00, 0.25]	0.84 [0.82, 0.89]	[[82, 16], [16, 2]]
		BERT	0.66 [0.46, 0.77]	0.37 [0.18, 0.62]	0.35 [0.17, 0.50]	0.64 [0.45, 0.84]	0.20 [−0.08, 0.48]	0.69 [0.65, 0.75]	0.58 [0.25, 1.00]	0.25 [0.12, 0.33]	0.90 [0.81, 1.00]	[[68, 30], [8, 10]]
		BERT (slide)	0.61 [0.42, 0.75]	0.28 [0.17, 0.51]	0.24 [0.15, 0.35]	0.53 [0.42, 0.65]	0.04 [−0.12, 0.21]	0.61 [0.50, 0.68]	0.45 [0.25, 0.75]	0.17 [0.11, 0.23]	0.86 [0.80, 0.92]	[[60, 38], [10, 8]]
	Multimodal	Feature fusion (freeze)	0.64 [0.36, 0.81]	0.38 [0.16, 0.67]	0.18 [0.00, 0.33]	0.54 [0.47, 0.63]	0.07 [−0.05, 0.22]	0.53 [0.20, 1.00]	0.55 [0.00, 1.00]	0.11 [0.00, 0.20]	0.90 [0.80, 1.00]	[[52, 46], [8, 10]]
		Feature fusion (finetune)	0.60 [0.35, 0.77]	0.33 [0.15, 0.60]	0.28 [0.00, 0.50]	0.59 [0.47, 0.84]	0.13 [−0.09, 0.48]	0.45 [0.00, 0.95]	0.73 [0.00, 1.00]	0.17 [0.00, 0.33]	0.75 [0.00, 1.00]	[[44, 54], [5, 13]]
		Cross attention fusion	0.65 [0.42, 0.82]	0.30 [0.17, 0.49]	0.30 [0.13, 0.46]	0.58 [0.38, 0.82]	0.13 [−0.19, 0.44]	0.52 [0.05, 0.70]	0.65 [0.25, 1.00]	0.21 [0.09, 0.30]	0.90 [0.77, 1.00]	[[51, 47], [7, 11]]
	Our Model	SepsisDRM + MLP	**0.82 [0.64, 0.96]**	**0.65 [0.25, 0.87]**	**0.54 [0.42, 0.75]**	**0.79 [0.68, 0.92]**	**0.48 [0.32, 0.70]**	0.73 [0.45, 0.95]	**0.85 [0.50, 1.00]**	**0.44 [0.27, 0.75]**	**0.97 [0.89, 1.00]**	**[[72, 26], [3, 15]]**
SYSMH external	Only Table	Logistic regression	0.68 [0.55, 0.84]	**0.41 [0.25, 0.61]**	0.41 [0.23, 0.55]	0.63 [0.46, 0.75]	0.22 [−0.07, 0.41]	0.69 [0.61, 0.78]	0.58 [0.31, 0.85]	0.33 [0.18, 0.41]	0.86 [0.76, 0.94]	[[157, 72], [27, 36]]
		MLP	0.62 [0.51, 0.78]	0.39 [0.23, 0.60]	0.35 [0.20, 0.44]	0.59 [0.50, 0.66]	0.17 [0.00, 0.27]	0.62 [0.35, 0.89]	0.56 [0.15, 0.92]	0.29 [0.22, 0.38]	0.86 [0.78, 0.95]	[[143, 86], [28, 35]]
		FT-Transformer	0.59 [0.50, 0.75]	0.38 [0.23, 0.58]	0.37 [0.32, 0.42]	0.54 [0.46, 0.62]	0.03 [−0.26, 0.21]	0.32 [0.00, 0.74]	**0.76 [0.50, 0.92]**	0.25 [0.20, 0.33]	0.66 [0.00, 0.89]	[[74, 155], [15, 48]]
	Only Text	TFIDF + LogReg	0.51 [0.41, 0.56]	0.30 [0.22, 0.38]	0.25 [0.18, 0.34]	0.53 [0.50, 0.57]	0.05 [0.00, 0.14]	**0.80 [0.76, 0.85]**	0.25 [0.15, 0.38]	0.25 [0.22, 0.31]	0.80 [0.78, 0.81]	[[184, 45], [47, 16]]
		BERT	0.56 [0.47, 0.72]	0.34 [0.24, 0.52]	0.26 [0.15, 0.36]	0.52 [0.46, 0.60]	0.04 [−0.09, 0.23]	0.75 [0.62, 0.89]	0.29 [0.15, 0.42]	0.25 [0.15, 0.44]	0.79 [0.76, 0.83]	[[171, 58], [45, 18]]
		BERT (slide)	0.51 [0.38, 0.59]	0.29 [0.23, 0.41]	0.31 [0.20, 0.36]	0.54 [0.46, 0.59]	0.08 [−0.07, 0.16]	0.72 [0.61, 0.80]	0.37 [0.23, 0.46]	0.27 [0.18, 0.31]	0.80 [0.76, 0.83]	[[165, 64], [40, 23]]
	Multimodal	Feature fusion (freeze)	0.60 [0.38, 0.71]	0.32 [0.18, 0.43]	0.32 [0.21, 0.42]	0.54 [0.37, 0.63]	0.11 [−0.23, 0.33]	0.69 [0.35, 0.96]	0.39 [0.15, 0.77]	**0.36 [0.14, 0.57]**	0.80 [0.67, 0.87]	[[158, 71], [38, 25]]
		Feature fusion (finetune)	0.53 [0.40, 0.67]	0.29 [0.19, 0.41]	0.27 [0.00, 0.36]	0.49 [0.45, 0.50]	−0.02 [−0.09, 0.00]	0.25 [0.00, 1.00]	0.73 [0.00, 1.00]	0.17 [0.00, 0.22]	0.30 [0.00, 0.78]	[[57, 172], [17, 46]]
		Cross attention fusion	0.59 [0.46, 0.71]	0.36 [0.22, 0.48]	0.31 [0.12, 0.43]	0.53 [0.50, 0.62]	0.04 [−0.00, 0.21]	0.34 [0.00, 0.93]	0.71 [0.08, 1.00]	0.24 [0.21, 0.29]	0.49 [0.00, 0.88]	[[79, 150], [19, 44]]
	Our Model	SepsisDRM + MLP	**0.69 [0.63, 0.76]**	0.38 [0.35, 0.45]	**0.47 [0.37, 0.57]**	**0.65 [0.52, 0.75]**	**0.28 [0.10, 0.45]**	0.56 [0.04, 0.83]	0.74 [0.62, 1.00]	**0.36 [0.23, 0.50]**	**0.91 [0.85, 1.00]**	**[[129, 100], [16, 47]]**

Reported metrics are AUC, AUPRC, F1, Balanced Accuracy, MCC, Specificity, Sensitivity, PPV, NPV, and Out-of-Fold (OOF) confusion matrix. All values are mean [min, max] from five-fold cross-validation; the Out-of-Fold (OOF) confusion matrices are presented as [[TN, FN], [FP, TP]] (true negative, false negative, false positive, true positive), aggregating per-fold predictions on held-out data across 5-fold cross-validation to approximate generalization performance; bold indicates the best and underlining the second-best within each dataset-metric. Threshold-dependent metrics are computed at a fixed decision threshold of 0.5.

### Comparison of human experts and SepsisDRM in predicting 28-day outcomes for sepsis patients

In the experiment of predicting 28-day outcomes for sepsis patients, we invited 11 human experts, including 2 residents, 5 attending physicians, and 4 consulting physicians, to predict the 28-day outcome. The detailed experimental setup is described in the section “Methodology for comparing SepsisDRM and human expert performance”. This experiment was conducted on a prospective dataset from GDHCM, with human experts making predictions after reviewing patient data (with names and IDs removed). The variables presented to the experts were identical to those used in the SepsisDRM model for training and testing, and the task was to predict whether the patient would survive or die within 28 days of hospital admission. We presented F1, Balanced Accuracy, MCC, Specificity, Sensitivity, PPV, and NPV of 11 medical experts and SepsisDRM in Table 8, and the detailed information of human experts are shown in Supplementary Table 4.

**Table 8:** Performance comparison between individual experts and the proposed model (SepsisDRM) on the GDHCM prospective cohort

Expert/Method	F1	Balanced Acc	MCC	Specificity	Sensitivity	PPV	NPV	Confusion matrix
Resident 1	0.29	0.57	0.1	0.58	0.56	0.2	0.88	[[57, 41], [8, 10]]
Resident 2	0.47	0.74	0.37	0.76	0.72	0.35	0.94	[[74, 24], [5, 13]]
Attending 1	0.27	0.57	0.13	0.86	0.28	0.26	0.87	[[84, 14], [13, 5]]
Attending 2	0.36	0.61	0.29	0.95	0.28	0.5	0.88	[[93, 5], [13, 5]]
Attending 3	0.3	0.58	0.13	0.72	0.44	0.23	0.88	[[71, 27], [10, 8]]
Attending 4	0.38	0.67	0.24	0.61	0.72	0.25	0.92	[[60, 38], [5, 13]]
Attending 5	0.33	0.61	0.21	0.88	0.33	0.33	0.88	[[86, 12], [12, 6]]
Consulting 1	0.34	0.62	0.18	0.63	0.61	0.23	0.9	[[62, 36], [7, 11]]
Consulting 2	0.43	0.69	0.31	0.78	0.61	0.33	0.92	[[76, 22], [7, 11]]
Consulting 3	0.49	0.71	0.38	0.87	0.56	0.43	0.91	[[85, 13], [8, 10]]
Consulting 4	0.33	0.6	0.33	**0.98**	0.22	**0.67**	0.87	[[96, 2], [14, 4]]
SepsisDRM	**0.54 [0.42, 0.75]**	**0.79 [0.68, 0.92]**	**0.48 [0.32, 0.70]**	0.73 [0.45, 0.95]	**0.85 [0.50, 1.00]**	0.44 [0.27, 0.75]	**0.97 [0.89, 1.00]**	**[[72, 26], [3, 15]]**

Bold values indicate the best performance across all experts / methods, and underlined values denote the second-best performance. For SepsisDRM, results are reported as mean [min, max] based on five-fold cross-validation. The Out-of-Fold (OOF) confusion matrices are presented as [[TN, FN], [FP, TP]] (True Negative, False Negative, False Positive, True Positive), aggregating per-fold predictions on held-out data across 5-fold cross-validation to approximate generalization performance; bold indicates the best and underlining the second-best within each dataset-metric. Threshold-dependent metrics are computed at a fixed decision threshold of 0.5.

**Fig. 12:**
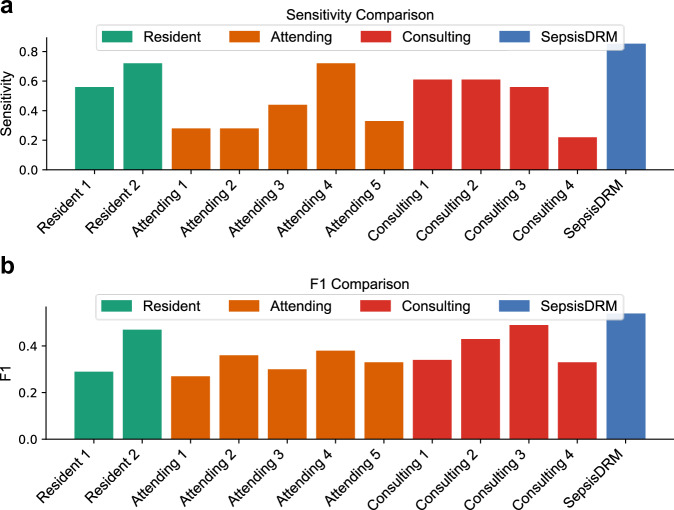
Performance comparison between medical experts and SepsisDRM on predicting 28-day outcomes in sepsis patients. **a** The sensitivity comparison between medical experts and SepsisDRM. **b** The F1 comparison between medical experts and SepsisDRM.

At a pre-specified and fixed decision threshold of 0.5, we report threshold-dependent metrics that directly reflect the clinical cost trade-off. Compared with 11 experts, SepsisDRM + MLP exhibits a more conservative, low-missed-death profile at this operating point: Sensitivity is 0.85 [0.50, 1.00], exceeding all experts (expert median 0.56, maximum 0.72), as shown in Fig. 12a. The wide range of expert performance (e.g., Sensitivity varying from 0.50 to 0.72) highlights significant inter-rater variability in human clinical judgment. In contrast, SepsisDRM provides a stable and consistent prediction. The Negative Predictive Value (NPV) is 0.97 [0.89, 1.00], also higher than all experts (median 0.88, maximum 0.94), supporting trustworthy rule-out decisions. The model’s F1 = 0.54 [0.42, 0.75], Balanced Accuracy = 0.79 [0.68, 0.92], and Matthews correlation coefficient (MCC) = 0.48 [0.32, 0.70] each exceed the best expert (expert maxima F1 = 0.49, BalAcc = 0.74, MCC = 0.38), as shown in Fig. 12b and Table 8, indicating that the gains are not achieved by a degenerate “predict-negative” strategy but by improved discrimination across classes. While prioritizing fewer missed deaths, the model’s specificity = 0.73 [0.45, 0.95] is slightly below the expert median (0.78); nevertheless, the positive predictive value (PPV) = 0.44 [0.27, 0.75] remains superior to 9/11 experts (median 0.33), showing that positive alerts are not indiscriminately inflated. Overall, the combination of high sensitivity and high NPV, together with competitive PPV, F1, Balanced Accuracy, and MCC at the fixed threshold of 0.5, reflects a clinically preferred setting where avoiding false negatives is prioritized over avoiding false positives. This indicates that the model operates in a conservative and reliable regime, rather than showing only a superficial gain in AUC.

From the comparison between human experts and SepsisDRM, we can observe that, unlike human doctors who rely on experience for judgment, predicting 28-day outcomes is better suited to data-driven approaches like SepsisDRM. Sepsis is a highly heterogeneous and rapidly evolving condition with complex pathophysiological processes, which makes it difficult for even experienced clinicians to predict outcomes accurately. Data-driven models like SepsisDRM can systematically integrate a vast range of clinical variables (e.g., demographic information, SOFA scores, lab tests, etc.) and detect subtle correlations that may not be apparent to human experts. This ability to extract richer, high-quality representations allows SepsisDRM to make more accurate predictions.

Interestingly, we also found that higher seniority among the doctors did not necessarily result in more accurate predictions. Experienced physicians may often rely on established heuristics or prior clinical experience, which might not align with the specific patterns present in the data. In contrast, less experienced doctors might approach the task in a more data-driven manner, aligning more closely with the structured nature of the dataset. This observation suggests that while clinical experience is invaluable, it may not always capture the nuanced and data-specific features that models like SepsisDRM can identify. Therefore, SepsisDRM can offer valuable insights to physicians in the diagnosis and management of sepsis patients.

### Ablation study

To systematically assess the contribution of different information sources, we conducted two types of ablation experiments on both unsupervised clustering and supervised classification tasks. First, we compared table-only, text-only, and multimodal variants of our model to evaluate the impact of individual modalities. Second, we further removed specific components of the input data (e.g., demographics, SOFA scores, laboratory tests, diseases, microbiological results, and CT reports) to quantify their respective contributions.

**Fig. 13:**
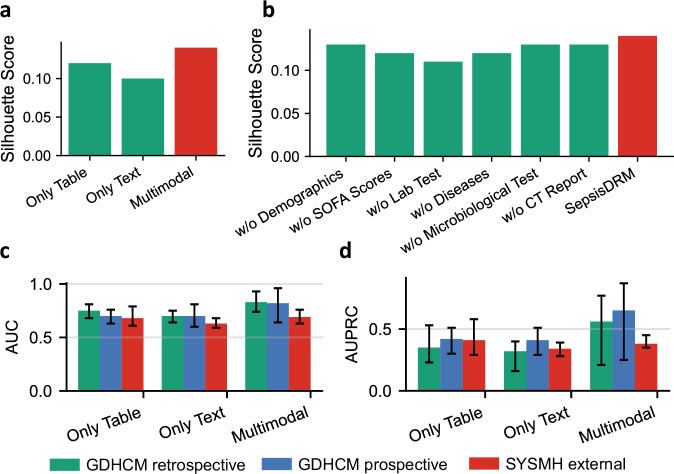
Ablation results on clustering and classification tasks. **a** and **b** Silhouette score of modality ablations (table-only, text-only, and multimodal) and component ablations (removing specific input types including demographics, SOFA scores, laboratory tests, diseases, microbiological tests, or CT reports), respectively. **c** and **d** AUC and AUPRC performance of modality ablations across the GDHCM retrospective, GDHCM prospective, and SYSMH external cohorts.

In the unsupervised setting, clustering quality was evaluated using Silhouette score (higher is better), Calinski–Harabasz score (higher is better), and Davies–Bouldin score (lower is better). As shown in Fig. 13a and Table 9, the multimodal model consistently yields the best clustering quality across all three metrics (Silhouette = 0.14, Calinski–Harabasz = 2137.26, Davies–Bouldin = 1.97). The table-only variant achieves moderate performance (0.12/1938.00/2.27), while the text-only variant performs worst (0.10/1175.72/2.86). These findings suggest that tabular variables capture more structured cluster information than textual data, but their integration produces more cohesive and better-separated phenotypic subgroups, highlighting the necessity of multimodal fusion for robust and clinically meaningful patient stratification.

**Table 9:** Comparison of ablation settings on clustering task

Modality	Silhouette score	Calinski–Harabasz score	Davies–Bouldin score
Only Table	0.12	1938.00	2.27
Only Text	0.10	1175.72	2.86
Multimodal	**0.14**	**2137.26**	**1.97**
**Ablation**	**Silhouette**	**Calinski_Harabasz**	**Davies_Bouldin**
w/o Demographics	0.13	2071.52	2.01
w/o SOFA Scores	0.12	1996.48	1.98
w/o Lab Test	0.11	1308.96	2.54
w/o Diseases	0.12	1412.41	2.34
w/o Microbiological test	0.13	1803.57	2.07
w/o CT Report	0.13	1942.55	2.21
SepsisDRM	**0.14**	**2137.26**	**1.97**

The upper part of the table presents performance when using different input modalities ("Only Table” means only input tabular variables, “Only Text” means only input textual variables, and “Multimodal” means input both tabular and textual variables), while the lower part shows model performance after removing different components of the input ("w/o” means “without”). Reported metrics are Silhouette score, Calinski–Harabasz score, and Davies–Bouldin score. Bold values indicate the best performance across all methods.

Beyond modality-level comparisons, ablations of specific input components further reveal their relative contributions, as shown in Fig. 13b and Table 9. Removing laboratory tests or disease information leads to a pronounced decline in clustering quality (e.g., Silhouette = 0.11 and 0.12, Davies–Bouldin = 2.54 and 2.34), indicating their central role in distinguishing patient subgroups. In contrast, removing demographics, SOFA scores, microbiological tests or CT reports only slightly reduces performance (e.g., Silhouette = 0.13, 0.12, 0.13 and 0.13, Davies–Bouldin = 2.01, 1.98, 2.07 and 2.21). These results suggest that while all components provide complementary information, certain clinical variables, particularly laboratory tests and disease profiles, are critical for capturing meaningful patient structure.

Building on these unsupervised findings, we next evaluated classification performance across the GDHCM retrospective, GDHCM prospective, and SYSMH external cohorts. Performance was assessed using both threshold-free discrimination metrics (AUC and AUPRC) and clinically relevant threshold-dependent metrics (F1, Balanced Accuracy, MCC, Sensitivity, Specificity, PPV, and NPV), reported as mean [min, max] across five-fold cross-validation. We also calculated the out-of-fold confusion matrices. All these results are presented in Table 10.

**Table 10:** Comparison of ablation settings on the GDHCM retrospective, GDHCM prospective, and SYSMH external datasets

Dataset	Modality	AUC	AUPRC	F1	Balanced_Accuracy	MCC	Specificity	Sensitivity	PPV	NPV	Confusion matrix
GDHCM retrospective	Only Table	0.75 [0.68, 0.81]	0.35 [0.23, 0.53]	0.20 [0.00, 0.29]	0.55 [0.50, 0.61]	0.09 [0.00, 0.20]	0.49 [0.00, 1.00]	0.62 [0.00, 1.00]	0.13 [0.00, 0.25]	0.75 [0.00, 1.00]	[[69, 74], [6, 12]]
	Only Text	0.70 [0.64, 0.75]	0.32 [0.16, 0.40]	0.23 [0.18, 0.27]	0.57 [0.53, 0.62]	0.09 [0.04, 0.15]	0.52 [0.39, 0.66]	0.62 [0.50, 0.75]	0.14 [0.11, 0.17]	0.92 [0.89, 0.93]	[[74, 69], [7, 11]]
	Multimodal	**0.83 [0.74, 0.93]**	**0.56 [0.21, 0.77]**	**0.37 [0.33, 0.50]**	**0.73 [0.61, 0.86]**	**0.33 [0.27, 0.49]**	**0.73 [0.48, 0.97]**	**0.72 [0.25, 1.00]**	**0.30 [0.21, 0.50]**	**0.96 [0.90, 1.00]**	**[[105, 38], [5, 13]]**
GDHCM prospective	Only Table	0.70 [0.63, 0.76]	0.42 [0.30, 0.51]	0.28 [0.00, 0.50]	0.61 [0.42, 0.80]	0.16 [−0.17, 0.45]	0.61 [0.35, 0.85]	0.62 [0.00, 1.00]	0.19 [0.00, 0.33]	0.91 [0.81, 1.00]	[[60, 38], [7, 11]]
	Only Text	0.70 [0.60, 0.81]	0.41 [0.29, 0.51]	0.26 [0.00, 0.40]	0.57 [0.45, 0.65]	0.11 [−0.13, 0.26]	0.61 [0.15, 0.89]	0.53 [0.00, 1.00]	0.19 [0.00, 0.33]	0.90 [0.85, 1.00]	[[59, 39], [8, 10]]
	Multimodal	**0.82 [0.64, 0.96]**	**0.65 [0.25, 0.87]**	**0.54 [0.42, 0.75]**	**0.79 [0.68, 0.92]**	**0.48 [0.32, 0.70]**	**0.73 [0.45, 0.95]**	**0.85 [0.50, 1.00]**	**0.44 [0.27, 0.75]**	**0.97 [0.89, 1.00]**	**[[72, 26], [3, 15]]**
SYSMH external	Only Table	0.68 [0.61, 0.79]	**0.41 [0.29, 0.58]**	0.38 [0.26, 0.49]	0.59 [0.49, 0.69]	0.16 [−0.01, 0.32]	0.52 [0.24, 0.85]	0.67 [0.23, 0.85]	0.28 [0.20, 0.34]	0.85 [0.79, 0.93]	[[118, 111], [21, 42]]
	Only Text	0.63 [0.59, 0.68]	0.34 [0.28, 0.39]	0.33 [0.25, 0.38]	0.54 [0.44, 0.61]	0.07 [−0.20, 0.23]	0.52 [0.02, 0.89]	0.55 [0.23, 1.00]	0.30 [0.19, 0.44]	0.79 [0.50, 1.00]	[[120, 109], [29, 34]]
	Multimodal	**0.69 [0.63, 0.76]**	0.38 [0.35, 0.45]	**0.47 [0.37, 0.57]**	**0.65 [0.52, 0.75]**	**0.28 [0.10, 0.45]**	**0.56 [0.04, 0.83]**	**0.74 [0.62, 1.00]**	**0.36 [0.23, 0.50]**	**0.91 [0.85, 1.00]**	**[[129, 100], [16, 47]]**
**Dataset**	**Ablation**	**AUC**	**AUPRC**	**F1**	**Balanced_Accuracy**	**MCC**	**Specificity**	**Sensitivity**	**PPV**	**NPV**	**Confusion Matrix**
GDHCM retrospective	w/o Demographics	0.75 [0.60,0.86]	0.44 [0.17,0.58]	0.30 [0.21,0.44]	0.63 [0.58,0.70]	0.20 [0.11,0.36]	0.62 [0.18,0.90]	0.63 [0.50,1.00]	0.22 [0.12,0.40]	0.94 [0.90,1.00]	[[89, 54], [7, 11]]
	w/o SOFA Scores	0.77 [0.63,0.85]	0.37 [0.18,0.54]	0.31 [0.13,0.44]	0.62 [0.45,0.70]	0.21 [−0.06,0.36]	0.63 [0.18,0.90]	0.62 [0.33,1.00]	0.24 [0.08,0.40]	0.94 [0.89,1.00]	[[90, 53], [7, 11]]
	w/o Lab Test	0.73 [0.68,0.79]	0.42 [0.18,0.51]	0.25 [0.18,0.29]	0.60 [0.53,0.70]	0.13 [0.04,0.24]	0.52 [0.39,0.69]	0.68 [0.50,1.00]	0.15 [0.11,0.18]	0.93 [0.90,1.00]	[[75, 68], [6, 12]]
	w/o Diseases	0.77 [0.65,0.88]	0.40 [0.17,0.62]	0.29 [0.19,0.44]	0.63 [0.55,0.70]	0.21 [0.11,0.36]	0.46 [0.11,0.90]	**0.80 [0.50,1.00]**	0.21 [0.11,0.40]	**0.97 [0.92,1.00]**	[[66, 77], [4, 14]]
	w/o Microbiological Test	0.71 [0.63,0.85]	0.31 [0.18,0.65]	0.21 [0.00,0.36]	0.60 [0.48,0.76]	0.12 [−0.06,0.34]	0.51 [0.21,0.96]	0.68 [0.00,1.00]	0.13 [0.00,0.22]	0.95 [0.90,1.00]	[[72, 71], [5, 13]]
	w/o CT Report	0.76 [0.62,0.88]	0.39 [0.18,0.54]	**0.38 [0.21,0.57]**	0.66 [0.59,0.73]	0.31 [0.14,0.53]	**0.76 [0.18,0.97]**	0.57 [0.33,1.00]	**0.36 [0.12,0.67]**	0.94 [0.93,1.00]	[[109, 34], [8, 10]]
	SepsisDRM	**0.83 [0.74, 0.93]**	**0.56 [0.21, 0.77]**	0.37 [0.33, 0.50]	**0.73 [0.61, 0.86]**	**0.33 [0.27, 0.49]**	0.73 [0.48, 0.97]	0.72 [0.25, 1.00]	0.30 [0.21, 0.50]	0.96 [0.90, 1.00]	**[[105, 38], [5, 13]]**
GDHCM prospective	w/o Demographics	0.76 [0.66,0.89]	0.56 [0.26,0.80]	0.42 [0.29,0.67]	0.68 [0.58,0.81]	0.31 [0.17,0.61]	0.69 [0.30,0.95]	0.67 [0.25,1.00]	0.35 [0.22,0.67]	0.93 [0.86,1.00]	[[67, 31], [6, 12]]
	w/o SOFA Scores	0.77 [0.71,0.84]	0.55 [0.31,0.73]	0.38 [0.25,0.55]	0.66 [0.55,0.78]	0.25 [0.10,0.45]	0.70 [0.50,0.85]	0.62 [0.25,0.75]	0.29 [0.20,0.43]	0.91 [0.85,0.94]	[[69, 29], [7, 11]]
	w/o Lab Test	0.75 [0.66,0.89]	0.46 [0.29,0.70]	0.42 [0.31,0.57]	0.68 [0.58,0.82]	0.32 [0.12,0.51]	0.63 [0.15,0.95]	0.73 [0.50,1.00]	0.34 [0.19,0.67]	0.94 [0.87,1.00]	[[62, 36], [5, 13]]
	w/o Diseases	0.80 [0.68,0.98]	0.62 [0.34,0.92]	0.37 [0.27,0.57]	0.63 [0.50,0.73]	0.24 [0.00,0.51]	0.63 [0.25,0.95]	0.63 [0.25,1.00]	0.32 [0.17,0.67]	0.90 [0.83,1.00]	[[62, 36], [7, 11]]
	w/o Microbiological Test	0.74 [0.68,0.84]	0.44 [0.28,0.81]	0.40 [0.29,0.60]	0.67 [0.59,0.80]	0.30 [0.16,0.52]	0.58 [0.21,0.85]	0.77 [0.33,1.00]	0.30 [0.17,0.50]	0.95 [0.89,1.00]	[[57, 41], [4, 14]]
	w/o CT Report	0.78 [0.60,0.98]	0.60 [0.23,0.92]	0.26 [0.00,0.40]	0.59 [0.43,0.76]	0.12 [−0.17,0.36]	0.65 [0.35,0.85]	0.53 [0.00,1.00]	0.19 [0.00,0.25]	0.89 [0.81,1.00]	[[64, 34], [9, 9]]
	SepsisDRM	**0.82 [0.64, 0.96]**	**0.65 [0.25, 0.87]**	**0.54 [0.42, 0.75]**	**0.79 [0.68, 0.92]**	**0.48 [0.32, 0.70]**	**0.73 [0.45, 0.95]**	**0.85 [0.50, 1.00]**	**0.44 [0.27, 0.75]**	**0.97 [0.89, 1.00]**	**[[72, 26], [3, 15]]**
SYSMH external	w/o Demographics	0.66 [0.60,0.77]	0.39 [0.31,0.50]	0.33 [0.25,0.40]	0.53 [0.50,0.62]	0.07 [0.00,0.21]	0.43 [0.00,0.83]	0.64 [0.23,1.00]	0.25 [0.21,0.33]	0.68 [0.00,1.00]	[[98, 131], [23, 40]]
	w/o SOFA Scores	0.67 [0.61,0.72]	0.39 [0.28,0.54]	0.38 [0.35,0.41]	0.58 [0.52,0.61]	0.14 [0.10,0.21]	0.45 [0.04,0.72]	0.70 [0.46,1.00]	0.27 [0.23,0.32]	0.89 [0.82,1.00]	[[103, 126], [19, 44]]
	w/o Lab Test	0.63 [0.60,0.67]	0.34 [0.31,0.37]	0.30 [0.19,0.42]	0.56 [0.51,0.61]	0.12 [0.03,0.19]	**0.74 [0.37,0.89]**	0.38 [0.15,0.85]	0.30 [0.25,0.38]	0.82 [0.78,0.89]	[[170, 59], [39, 24]]
	w/o Diseases	0.65 [0.60,0.69]	0.37 [0.32,0.43]	0.33 [0.19,0.41]	0.55 [0.51,0.59]	0.09 [0.03,0.21]	0.41 [0.17,0.87]	0.68 [0.15,1.00]	0.24 [0.23,0.25]	0.86 [0.78,1.00]	[[93, 136], [20, 43]]
	w/o Microbiological Test	0.68 [0.63,0.73]	**0.40 [0.35,0.44]**	0.40 [0.36,0.47]	0.59 [0.52,0.69]	0.17 [0.10,0.31]	0.41 [0.04,0.63]	**0.78 [0.54,1.00]**	0.28 [0.23,0.35]	0.90 [0.82,1.00]	[[79, 150], [19, 44]]
	w/o CT Report	0.65 [0.59,0.67]	0.35 [0.32,0.41]	0.36 [0.35,0.38]	0.56 [0.50,0.59]	0.11 [0.00,0.16]	0.48 [0.00,0.72]	0.64 [0.46,1.00]	0.27 [0.21,0.32]	0.66 [0.00,0.84]	[[111, 118], [23, 40]]
	SepsisDRM	**0.69 [0.63, 0.76]**	0.38 [0.35, 0.45]	**0.47 [0.37, 0.57]**	**0.65 [0.52, 0.75]**	**0.28 [0.10, 0.45]**	0.56 [0.04, 0.83]	0.74 [0.62, 1.00]	**0.36 [0.23, 0.50]**	**0.91 [0.85, 1.00]**	**[[129, 100], [16, 47]]**

The upper part of the table presents performance when using different input modalities ("Only Table” means only input tabular variables, “Only Text” means only input textual variables, and “Multimodal” means input both tabular and textual variables), while the lower part shows model performance after removing different components of the input ("w/o” means “without”). Metrics include AUC, AUPRC, F1, Balanced Accuracy, MCC, Specificity, Sensitivity, PPV, and NPV, reported as mean [min, max] from five-fold cross-validation. Bold values denote the best performance within each dataset-metric, and underlined values denote the second-best performance.

Across all datasets, the multimodal model consistently achieves superior or comparable discrimination while providing more favorable error-balanced performance. On the GDHCM retrospective dataset, multimodal attains the highest discrimination (AUC = 0.83, AUPRC = 0.56), outperforming the table-only (0.75/0.35) and text-only (0.70/0.32) baselines, as shown in Fig. 13c and d. It further improves threshold-dependent outcomes, including F1 (0.37 vs. 0.20/0.23), Balanced Accuracy (0.73 vs. 0.55/0.57), and MCC (0.33 vs. 0.09/0.09), while simultaneously enhancing Specificity (0.73), Sensitivity (0.72), PPV (0.30), and NPV (0.96), as shown in Table 10. On the GDHCM prospective dataset, multimodal again delivers the best discrimination (AUC = 0.82, AUPRC = 0.65), as shown in Fig. 13c and d, and substantially outperforms unimodal variants on threshold-dependent measures: F1 (0.54 vs. 0.28/0.26), Balanced Accuracy (0.79 vs. 0.61/0.57), MCC (0.48 vs. 0.16/0.11), along with markedly higher Sensitivity (0.85) and NPV (0.97), as shown in Table 10. On the SYSMH external validation dataset, multimodal achieves competitive discrimination (AUC = 0.69, AUPRC = 0.38), close to the table-only variant (0.68/0.41), as shown in Fig. 13c and d, but shows clear advantages in F1 (0.47 vs. 0.38/0.33), Balanced Accuracy (0.65 vs. 0.59/0.54), MCC (0.28 vs. 0.16/0.07), Sensitivity (0.74 vs. 0.67/0.55), and NPV (0.91 vs. 0.85/0.79), as shown in Table 10. These ablation results suggest that structured and unstructured modalities provide complementary information: while unimodal models capture partial aspects of patient state, their fusion substantially enhances both discrimination and clinically meaningful operating characteristics. The findings highlight the multimodal design as essential for robust and clinically reliable performance across heterogeneous patient cohorts.

A closer comparison between unimodal variants further reveals that table-only models consistently outperform text-only models across all three datasets. On the GDHCM retrospective dataset, table-only achieves higher discrimination (AUC = 0.75 vs. 0.70) and comparable or better threshold-dependent metrics, as shown in Table 10. On the GDHCM prospective dataset, the advantage of tabular inputs persists but is more modest, with table-only slightly exceeding text-only across most metrics. On the SYSMH external cohort, table-only again surpasses text-only, most notably in AUPRC (0.41 vs. 0.34), as shown in Fig. 13d, indicating that structured variables are more robust under domain shift. These findings suggest that tabular data provide a stronger and more stable foundation for prognosis prediction, while textual data alone are less informative but contribute complementary signals. The consistent superiority of the multimodal model underscores that leveraging both modalities is essential to maximize predictive performance and generalizability.

**Fig. 14:**
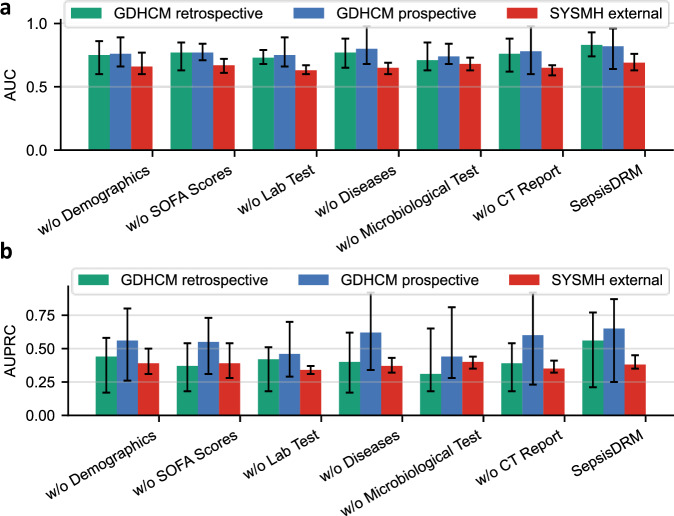
Component ablation results on supervised classification tasks. **a** and **b** AUC and AUPRC performance when removing specific input components (demographics, SOFA scores, laboratory tests, diseases, microbiological tests, or CT reports), compared with the full SepsisDRM model.

Beyond modality-level analysis, ablations of specific input components further highlight their relative contributions. In the GDHCM retrospective dataset, removing laboratory tests or microbiological tests markedly reduces performance (e.g., AUC = 0.73 and 0.71, AUPRC = 0.42 and 0.31), as shown in Figs. 14a and b, indicating their critical role in prediction. In the GDHCM prospective dataset, excluding laboratory tests, microbiological tests or demographics leads to noticeable drops in AUC (e.g., AUC = 0.75, 0.74 and 0.76, AUPRC = 0.46, 0.44 and 0.56), as shown in Fig. 14a and b, suggesting these features are especially valuable for real-time clinical assessments. In contrast, removing diseases produces more moderate effects (e.g., AUC = 0.80, AUPRC = 0.62), though still inferior to the full model. On the SYSMH external dataset, the impact of individual components varies, with the absence of laboratory tests leading to the largest decline in AUC (e.g., AUC = 0.63, AUPRC = 0.34), as shown in Fig. 14a and b, further emphasizing their robustness under domain shift. Taken together across the three datasets, laboratory tests consistently emerge as an influential component, whereas the contributions of other variables vary across datasets.

## Discussion

This study presents SepsisDRM, a multimodal embedding model that integrates tabular and textual data to characterize sepsis phenotypes and predict prognoses. We identified four clinically distinct phenotypes and developed a robust few-shot prognostic model. Notably, our analysis suggests that Xuebijing injection may specifically benefit the hyper-inflammatory phenotype. To our knowledge, this is the first application of multimodal embedding models for sepsis cohort characterization.

Data scarcity and incompleteness remain significant bottlenecks in sepsis research. Unlike previous studies restricted by small proprietary datasets^8^ or high missingness^7^, we utilized a large multi-center cohort for pre-training. To ensure data quality, we established a rigorous reliability threshold, excluding variables with excessive missingness. Furthermore, by leveraging unsupervised contrastive learning, SepsisDRM bypasses the need for large-scale labeled outcomes, enabling the utilization of vast amounts of unlabeled clinical data. Unlike approaches relying solely on tabular data^29^, our model extracts semantic features from clinical notes (e.g., CT reports), which we found crucial for distinguishing phenotypes (Fig. 5a).

Our clustering analysis revealed four phenotypes aligning with established clinical profiles. The MOFP (Multiple Organ Failure Phenotype) resembled the *δ* phenotype in Seymour et al.^7^, characterized by the highest organ dysfunction, comorbidity burden, and mortality. Biological analysis suggests this may involve the gut–liver axis and the Angiopoietin-2-HGF-C/EBP*β* pathway^30^, identifying potential therapeutic targets. In contrast, the LIP (Low Inflammation Phenotype) showed the best prognosis. Textual analysis corroborated these findings: MOFP records were enriched with terms like “liver cirrhosis” and “pulmonary edema,” whereas LIP records lacked organ-failure descriptors. These distinctions support the necessity of precision medicine-aggressive intervention for MOFP versus conservative management for LIP.

Regarding therapeutic implications, observational data suggested that Xuebijing Injection reduced mortality in the high inflammation phenotype (HIP). This aligns with its known mechanism of modulating immune responses^31^. Our findings reinforce that selecting appropriate sepsis subpopulations is critical for clinical trial success^7^, highlighting the value of SepsisDRM in patient stratification. However, as evidence for Xuebijing is primarily observational, prospective interventional studies are essential to establish causal efficacy and safety.

In classification tasks, SepsisDRM demonstrated strong few-shot learning capabilities. Across internal and prospective datasets, the model consistently outperformed baselines in both discrimination and calibration metrics. On the external validation dataset, while achieving the best ranking performance, the model showed a drop in precision-recall performance. This reflects significant domain shift arising from the inherent heterogeneity between institutions, including variations in clinical documentation styles, laboratory standards, and patient population characteristics. However, SepsisDRM remained competitive with baselines fully trained on the target domain, suggesting that the learned embeddings retain meaningful predictive signals even under such heterogeneity. Future work will mitigate this via domain adaptation (DA) strategies to align feature distributions across diverse environments.

Our comparative study showed SepsisDRM significantly outperforming the panel of human experts. These results underscore the potential of AI as a decision-support tool to augment clinical judgment, particularly where experience-based heuristics may fail. However, regarding the physician comparison, although all 11 experts reviewed the entire prospective cohort allowing for robust statistical comparison, the experts were recruited from limited institutions. Future studies involving a larger, multi-center cohort of clinicians would help to further evaluate the generalizability of the human baseline.

Despite these promising results, our study has several limitations. First, the reliance on observational data inherently introduces biases, such as selection bias and confounding variables. Second, the identified phenotypes derive primarily from a single-center cohort. Due to heterogeneity in EHR documentation and laboratory standards, direct application of these phenotypes to external datasets requires data harmonization. Thus, we deferred external phenotype validation to future multi-center studies. Third, the current model relies on admission data, limiting the capture of temporal disease evolution. Future work should incorporate longitudinal modeling. Fourth, regarding model interpretability, while we analyzed broad component contributions, future research will employ feature importance ranking (e.g., SHAP) to quantify the specific influence of critical biomarkers like CRP and PCT^32,33^.

Methodologically, we employed standard stratified cross-validation. While effective, we acknowledge that a nested cross-validation design (specifically with an outer Leave-One-Out loop) is often considered the gold standard for small datasets to strictly decouple model selection from evaluation and minimize selection bias^34^. However, given the high computational complexity of pre-training and fine-tuning our multimodal foundation model, implementing a nested approach was computationally prohibitive in this study. Consequently, our current performance estimates may carry some selection bias, and future work with greater computational resources should prioritize nested validation strategies. We also aim to validate clinical efficiency by measuring the ‘wall-clock time’ savings compared to manual expert review in future study. Moreover, we adopted RoBERTa rather than larger LLMs due to reproducibility and computational feasibility; future work could incorporate stronger backbones like Qwen. Additionally, the small size of our labeled datasets (due to the difficulty of obtaining 28-day outcomes) may limit statistical power. Finally, although SepsisDRM outperformed human experts, its specificity is not consistently high. Thus, we envision it as a decision-support tool to assist rather than replace clinicians, with ultimate responsibility remaining with medical professionals.

Deployment strategies regarding missing data also warrant attention. To ensure feature reliability, we empirically evaluated missingness thresholds of 40%, 50%, and 60%, ultimately establishing a strict 50% cut-off; our experiments confirmed that including variables exceeding this limit degraded performance due to the unreliability of reconstructing sparse feature spaces. For real-world deployment, we advocate for a local imputation strategy: end-users should apply standard imputation (e.g., Multiple Imputation for cohorts or population medians for single patients) on their local datasets before inference. This ensures data completion is tailored to the target population’s distribution.

In conclusion, SepsisDRM demonstrates the power of multimodal embedding models in characterizing sepsis heterogeneity and predicting outcomes, offering a robust tool for future precision medicine research.

## Methods

### Data curation

Our data were sourced from two hospitals (Guangdong Provincial Hospital of Chinese Medicine (GDHCM) and Sun Yat-sen Memorial Hospital, Sun Yat-sen University (SYSMH)) across five centers (Ersha Center, Fangcun Center, Dayuan Center, Daxuecheng Center from GDHCM, and Emergency Department of SYSMH), comprising a total of 19,934 cases. The inclusion criteria for the sepsis training and validation cohorts were as follows:

1. diagnosis consistent with Sepsis-3.0 criteria (According to Sepsis-3.0, sepsis is defined as life-threatening organ dysfunction caused by a dysregulated host response to infection, and sepsis is diagnosed when there is suspected or confirmed infection accompanied by an acute increase of ≥2 points in the SOFA score);
2. age ≥18 years old;
3. hospital stay exceeding 24 h;
4. blood culture performed;
5. hospitalization between January 1, 2014, and June 30, 2024;
6. first hospitalization due to sepsis.

**Fig. 15:**
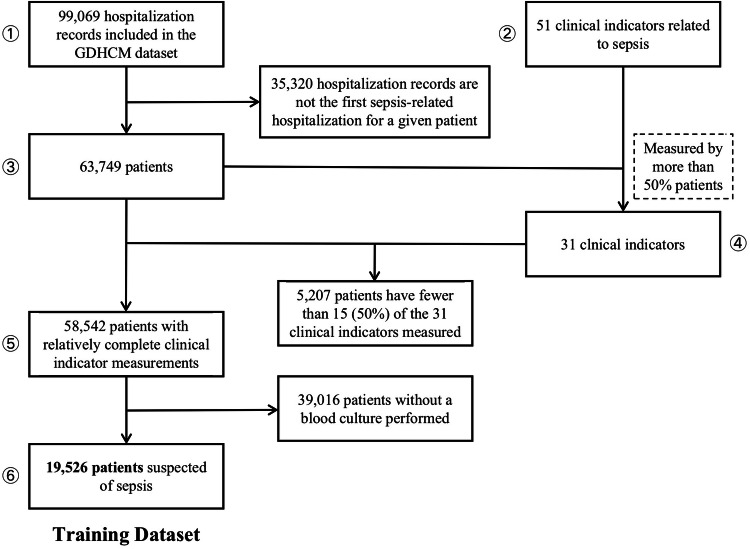
The complete data selection process of our training dataset. Among all hospitalized patients (99,069 admissions, including both sepsis-related and non-sepsis cases), those with sufficient data and a blood culture measurement were identified as suspected of sepsis, forming the final study cohort (19,526 patients).

For each patient, only the results from the first examination during hospitalization were included in the dataset. Through a series of rigorous including steps, we refined the initial coarse-grained dataset of 99,069 patients, ultimately narrowing it down to a high-quality cohort of 19,526 sepsis patients, which is our training dataset, as shown in Fig. 15. While the initial dataset was larger, it included many cases with lower data quality. After applying strict selection criteria, the resulting subset represents a more focused and reliable patient group for our analysis. Regarding the test sets, the GDHCM retrospective dataset comprises patients from the GDHCM database with 28-day outcome labels, totaling 161 cases. The GDHCM prospective dataset consists of prospective patients collected after the study’s initiation, specifically from July 1, 2024 to July 15, 2024, including 116 cases of sepsis patients. The SYSMH external validation dataset, collected from March 1, 2018 to October 30, 2022, contains a total of 292 sepsis patients. Both the GDHCM retrospective dataset and the SYSMH external validation dataset initially met our data inclusion criteria, while the GDHCM prospective dataset was collected in strict adherence to our inclusion criteria during the sampling process. Details of the training dataset baseline feature conditions for different centers are provided in Table 1, and validation dataset in Table 2.

We began by performing variable selection on our datasets. For the numerical variables, we considered four categories of tabular features: demographic information, sequential organ failure assessment (SOFA) scores, clinical laboratory indicators, and underlying diseases. Demographic information included sex and age at admission. SOFA scores consisted of the overall SOFA score and five organ-specific subscores (respiratory, cardiovascular, liver, coagulation, and renal). The selection of laboratory indicators was guided by their clinical availability, relevance to sepsis, and prior references from other sepsis phenotype studies. After excluding 35,320 hospitalization records that were not the first sepsis-related hospitalization for a given patient, we further removed laboratory indicators measured in fewer than 50% of patients from an initial set of 51 candidates, yielding 31 final laboratory indicators (see Supplementary Table 5). We also experimented with alternative thresholds (40% and 60%) and found that the 50% threshold achieved the best model performance (as shown in the Section “Hyperparameter tuning”). For the retained indicators, we employed multiple imputation^35^ to handle missing values. In addition, we incorporated 9 underlying diseases (e.g., hypertension, coronary artery disease, and diabetes), represented as binary variables. In total, the 48 tabular variables used for model development comprised 2 demographic variables, 6 SOFA-related variables, 31 laboratory indicators, and 9 binary disease indicators.

Additionally, we integrated two types of textual inputs: CT imaging reports and bacteriological examination results. For CT imaging, only the first report following hospital admission was used to prompt the model. In the case of bacteriological examinations, the prompt format was standardized as “sample: detected microorganisms”, where “sample” referred to the microbial culture samples analyzed. Both textual prompts, where available, were concatenated to form the final textual input for the model. Finally, the textual input including CT imaging reports and bacteriological examination results, and tabular input including 48 tabular variables, are fed together into SepsisDRM.

This study was conducted in accordance with the Declaration of Helsinki and received ethical approval from the ethics committees of Guangdong Provincial Hospital of Chinese Medicine (BE2024-196-01) and Sun Yat-sen Memorial Hospital, Sun Yat-sen University (SYSKY-2024-032-01). The sepsis cohort data from the five centers were retrospectively extracted from electronic health records (EHR) and underwent de-identification, with informed consent waivers obtained.

### Baselines

To evaluate the performance of SepsisDRM, we conducted comprehensive benchmarking against widely adopted algorithms for both clustering and classification tasks. Detailed architectures, hyperparameter settings, and implementation details for all baseline models are provided in Supplementary Information.

For the clustering task, we compared our approach against five standard paradigms evaluated on tabular, text, and fusion features. These included *K*-Means clustering^36^ (partition-based), agglomerative hierarchical clustering^37^ (Ward’s linkage), spectral clustering^38^ (graph-based), Gaussian mixture models (GMMs)^39^ (probabilistic), and DBSCAN^40^ (density-based). We reported the Silhouette Score^41^, Calinski–Harabasz Score^42^, and Davies–Bouldin Score^43^ as evaluation metrics.

For the classification task, we benchmarked SepsisDRM against baselines across three modalities. First, for *tabular-only* inputs, we implemented logistic regression (LR)^44^, multi-layer perceptron (MLP), and the state-of-the-art FT-Transformer^45^. Second, for *text-only* inputs, we utilized TF-IDF + logistic regression^44,46^ and transformer-based encoders, specifically BERT^47^ and RoBERTa^26^ (including sliding-window aggregation^48,49^ for long texts). Third, for *multimodal* comparisons, we evaluated Feature-Level Fusion architectures^50,51^ (with both frozen and fine-tuned text encoders) and Cross-Attention Fusion models^52–54^. All evaluations were performed using 5-fold cross-validation with identical folds. Transformer-based models use AdamW with linear learning-rate warmup^55^. To address class imbalance, we employed focal loss^56^ for neural models and class reweighting for tree-based or linear models.

### Model design and training

Our SepsisDRM employs a dual-stream encoding architecture to fuse multimodal clinical data. The framework consists of a tabular data encoder and a textual data encoder. For the tabular stream, given raw numerical inputs (48 variables), we utilize a multilayer perceptron (MLP) composed of three linear layers (mapping 48 → 768 → 2048 → 768) with ReLU activations. Crucially, the final layer of the MLP projects the tabular features into a ${{\bf{h}}}_{tab}\in {{\mathbb{R}}}^{d}$ vector where *d* = 768, strictly aligning with the output dimensionality of the textual encoder to ensure a balanced contribution from both modalities. For the textual stream, we utilize RoBERTa^26^ to process clinical notes, yielding a textual representation ${{\bf{h}}}_{text}\in {{\mathbb{R}}}^{d}$ (*d* = 768). The multimodal fusion is achieved by concatenating these two aligned representations to form the final ensembled embedding ${{\bf{h}}}_{ens}=[{{\bf{h}}}_{tab};{{\bf{h}}}_{text}]\in {{\mathbb{R}}}^{2d}$, resulting in a final dimensionality of 1536. These encoders are trained in an unsupervised manner using a contrastive learning mechanism inspired by SimCSE^57^. Regarding data augmentation, we rely on *standard dropout* (rate = 0.3) within the encoders as the sole augmentation strategy. Specifically, for a sample *x*_*i*_, the input is fed into the model twice with different random dropout masks, generating two distinct embeddings **h**_*i*_ and ${{\bf{h}}}_{i}^{{\prime} }$. These form a positive pair, while embeddings from other samples in the batch serve as negatives. The training objective is to minimize the InfoNCE loss calculated directly on these 1536-dimensional vectors:

1 $${\ell }_{i}=-\log \frac{{{\rm{e}}}^{{\rm{sim}}({{\bf{h}}}_{i},{{\bf{h}}}_{i}^{{\prime} })/\tau }}{{\sum }_{j=1}^{N}{{\rm{e}}}^{\mathrm{sim}({{\bf{h}}}_{i},{{\bf{h}}}_{j}^{{\prime} })/\tau }},$$

where ${\rm{sim}}\,(\cdot )$ denotes cosine similarity and *τ* is the temperature parameter (set to 0.05). This mechanism forces the model to learn spatially discriminative and semantically robust representations without requiring external data transformations.

Before feeding the training data into the model, we first normalize the numerical data for each sample, ensuring a mean of 0 and a standard deviation of 1. For the textual data in each sample, we tokenize the text and then input the resulting tokens into the model. We utilize the RoBERTa tokenizer with a truncation length set to the maximum of 512 tokens. Notably, 96.72% of the clinical notes fell below this threshold, so truncation affected only a very small proportion of unusually long records. During training, we minimize the contrastive learning loss in Eq. (1) over a single epoch. After experimenting with various parameter combinations, we set the learning rate to 1e−5, the batch size to 32, and the dropout rate to 0.3 (as shown in the section “Hyperparameter tuning”), training on an NVIDIA A800 80GB GPU. The representation dimension of the MLP is 768, with a hidden layer dimension set to 2048. The dimension of the representation obtained from RoBERTa is also 768. Finally, we concatenate the representations from the MLP and RoBERTa to obtain an ensembled representation with a dimension of 1536.

### Adapting SepsisDRM to clustering and classification task

Using the pre-trained embedding model as a representation extractor, we conducted clustering and classification tasks on our dataset. For the clustering task, we first utilized the embedding model to obtain representations for each sample, and then applied clustering methods. Given the large scale of our dataset, we selected *K*-Means clustering due to its excellent performance and high computational speed. After experimenting with various numbers of clusters, we ultimately selected four as the optimal number, as this choice yielded the most distinct separation across clinical variables. Clustering stability was quantified using the adjusted Rand index (ARI) over 100 runs with varying random seeds and 80% random subsampling.

For the classification task, we trained a classification head on top of the representations extracted by the embedding model. The labeled data was evaluated using a *stratified five-fold cross-validation protocol* to rigorously handle the class imbalance between sepsis and control cases, in which the dataset was partitioned into five folds with an 80:20 split at each iteration. In every run, the model was trained on four folds (80%) and evaluated on the held-out fold (20%), rotating the validation fold across the five runs. The classification head consisted of an MLP whose hidden dimension (*h*) and depth (*d*) were tuned per dataset. We set the batch size to 64 and trained the model for 50 epochs on an NVIDIA A800 80GB GPU. To explicitly address the class imbalance during optimization, we trained the classification head using *Focal Loss*^56^, which assigns higher weights to hard-to-classify examples (or the minority class), thereby preventing the model from being biased toward the majority class. Although Focal Loss improves class separation, it may affect probability calibration. Therefore, to ensure the validity of the default decision threshold (0.5), we performed an empirical post-hoc analysis using ROC curves. We visualized the operating point corresponding to the 0.5 threshold relative to the optimal trade-off position (maximizing sensitivity and specificity) to verify the alignment of the decision boundary. During this process, we froze all parameters of the embedding model and trained only the classification head. After training, we evaluated the model’s performance by comparing the predicted results with the ground truth, calculating evaluation metrics included AUC, AUPRC, F1, Balanced Accuracy, MCC, Specificity, Sensitivity, PPV, and NPV, and reporting the mean, minimum and maximum across the five folds. Besides, we also calculated The Out-of-Fold (OOF) confusion matrices by aggregating per-fold predictions on held-out data across five-fold cross-validation to approximate generalization performance.

### Methodology for comparing SepsisDRM and human expert performance

To conduct the comparative experiment between SepsisDRM and human experts, we invited 11 medical professionals to participate in our study (including 10 males and 1 female, with an average age of 36.45 years, as shown in Supplementary Table 4). All participants provided informed consent prior to the experiment. The group consisted of 2 residents (Resident 1, 2), 5 attending physicians (Attending 1–5), and 4 consulting physicians (Consulting 1–4), with Resident 1, Attending 1, Attending 2, Consulting 1, Consulting 3 holding MD degrees, and the remaining participants holding master’s degrees in medicine. Each participant had at least 5 years of experience in various medical fields.

During the experiment, the medical experts had access to all the variables used in the training and testing of the SepsisDRM model, as detailed in Tables 1 and 2. Crucially, to ensure a fair comparison, the experts reviewed the entire GDHCM Prospective Dataset (*n* = 116). All 11 physicians independently assessed the same set of patients without collaboration, allowing for a direct paired comparison. The information presented to the experts was strictly limited to the data available at the admission time point, ensuring an identical information window to the model. Future outcomes were redacted, and the physicians were blinded to the true 28-day mortality prevalence of the cohort. Based on these variables, they were asked to predict the 28-day outcome of each patient, specifically whether the patient would survive or die. After collecting the predictions from the human experts, we compared them against the ground truth and calculating F1, Balanced Accuracy, MCC, Specificity, Sensitivity, PPV, and NPV. Finally, we compared and analyzed the results of the human experts against the predictions made by the SepsisDRM model.

### Methodology for ablation study

To systematically examine the contribution of each modality and the robustness of our model design, we conducted ablation experiments during the training of the embedding model (encoder) under both clustering and classification settings. For modality-level ablations, we trained encoder variants using only tabular inputs, only textual inputs, or their full multimodal combination. For component-level ablations, we further trained models after removing specific subsets of variables, including demographics, SOFA scores, laboratory tests, diseases, microbiological tests, and CT reports. In the clustering task, the learned representations were partitioned into four subgroups using *K*-Means (*k* = 4), consistent with our study design, and evaluated with three standard metrics: silhouette coefficient, Calinski-Harabasz index, and Davies-Bouldin index. These analyses provide complementary perspectives on how different modalities and clinical components contribute to the formation of meaningful and separable patient structures.

In the classification task, the ablations were likewise incorporated during encoder training and classification head training. Separate models were trained for the tabular-only, text-only, and multimodal settings, as well as for each component-level removal. All models were evaluated using five-fold stratified cross-validation to ensure balanced distributions of positive and negative samples across folds. For every fold, the model was trained with the same optimization strategy as in the main experiments, and the checkpoint with the best validation AUC was retained. Out-of-fold (OOF) predictions were aggregated to compute overall confusion matrices, from which threshold-dependent metrics—including Sensitivity, Specificity, PPV, NPV, F1-score, Balanced Accuracy, and MCC-were derived, in addition to threshold-free metrics such as AUC and AUPRC.

### Methods of data analysis

Following the clustering process, we performed a statistical analysis of the clinical characteristics of samples within each cluster. First, we conduct Lilliefors test^58^ for each variable to determine which variables follow a normal distribution. For normally distributed continuous variables, data were presented as mean ± standard deviation (SD). For non-normally distributed continuous variables, data were presented as median with interquartile range (IQR). Categorical variables were expressed as frequencies and percentages. Then, we compared the group differences of each variable. For continuous variables that followed a normal distribution, one-way analysis of variance (ANOVA)^59^ was used to compare groups. If a significant difference was detected, the Benjamini and Hochberg^60^ correction was applied to adjust for multiple comparisons. For non-normally distributed data, the Kruskal–Wallis *H* test^61^ was used for group comparisons, and when significant differences were observed, the Dunn test^62^ was used for post-hoc multiple comparisons. For comparisons between categorical variables across multiple groups, the *χ*^2^-test was employed.

When analyzing the textual data of patients across different phenotypes, we calculate the significance of each term using the following method:

2 $$\,{\rm{TS}}(t,p)={\rm{TF}}(t,p)* {\rm{IPF}}\,(t,P),$$

where TS(*t*, *p*) stands for term significance, which is the metric we use during the term analysis process. TF(*t*, *p*) stands for term frequency, which measures how frequently term *t* appears in phenotype *p*, and can be represented as

3 $$\,{\mathrm{TF}}\,(t,p)=\frac{{n}_{t}^{p}}{{n}_{total}^{p}},$$

where ${n}_{t}^{p}$ is the number of times term *t* appears in phenotype *p*, and ${n}_{{\rm{total}}}^{p}$ is the total number of terms in phenotype *p*. IPF(*t*, *P*) stands for inverse phenotype frequency, which measures how important term *t* is across the entire dataset *P*, with more frequent terms being given less importance, and can be represented as

4 $$\,{\rm{IPF}}\,(t,P)=\log \left(\frac{{n}_{P}}{{n}_{P}^{t}}\right)$$

where *n*_*P*_ is the total number of phenotypes in corpus *P*, and ${n}_{P}^{t}$ is the number of phenotypes in *P* that contain term *t*. Afterward, we analyze the TS of terms across different phenotypes. This formulation is conceptually analogous to the classical TF–IDF metric, with phenotype frequency replacing document frequency, thereby tailoring the measure to phenotype-level text analysis.

In the analysis of drug use across different phenotypes, propensity score matching (PSM) was employed to reduce confounding and ensure comparability between the drug use and control groups. The propensity score was calculated using a logistic regression model, incorporating covariates such as age, sex, and comorbidities. Patients were matched 1:1 using the nearest neighbor matching algorithm with a caliper of 0.2. Covariate balance between matched groups was assessed using standardized mean differences (SMD), with values below 0.1 indicating adequate balance. After matching, logistic regression was performed to evaluate the association between drug use and in-hospital mortality.

Subgroup analyses were conducted based on phenotypes, and interaction terms were included in the regression models to assess heterogeneity across subgroups. A *p*-value < 0.05 was considered statistically significant. All statistical tests were two-sided. The statistical analyses were performed using R software (version 4.4.1), with the packages MatchIt, ggplot2, ggstatsplot and compareGroups.

### Hyperparameter tuning

To avoid potential bias from manual tuning, all hyperparameters in SepsisDRM were optimized using systematic grid search.

For the embedding model, we performed an extensive grid search over learning rate (1e−6, 5e−6, 1e−5, 5e−5, 1e−4), dropout rate (0.1, 0.2, 0.3, 0.4, 0.5), and training epochs (1–3). Table 11 lists the full validation loss results. The optimal configuration was identified as 1 epoch, learning rate = 1 × 10^−5^, dropout = 0.3, which yielded the lowest validation loss (0.0006). Although we also tested longer training (2–3 epochs) and broader parameter ranges, these consistently resulted in higher validation loss, suggesting overfitting or convergence issues. Thus, training for only 1 epoch effectively acted as an empirical early stopping strategy guided by validation performance.

**Table 11:** Full grid search results for the embedding model hyperparameters

Learning rate	Dropout	Validation Loss		
		Epoch Num = 1	Epoch Num = 2	Epoch Num = 3
1 × 10^−6^	0.1	0.0062	0.0055	0.0048
	0.2	0.0061	0.0054	0.0047
	0.3	0.0063	0.0056	0.0049
	0.4	0.0065	0.0058	0.0051
	0.5	0.0068	0.0060	0.0053
5 × 10^−6^	0.1	0.0042	0.0035	0.0030
	0.2	0.0040	0.0033	0.0029
	0.3	0.0041	0.0035	0.0031
	0.4	0.0043	0.0038	0.0034
	0.5	0.0046	0.0041	0.0037
1 × 10^−5^	0.1	0.0021	0.0028	0.0032
	0.2	0.0015	0.0021	0.0024
	0.3	**0.0006**	0.0030	0.0031
	0.4	0.0012	0.0022	0.0035
	0.5	0.0018	0.0025	0.0039
5 × 10^−5^	0.1	0.0013	0.0032	0.0027
	0.2	0.0011	0.0020	0.0022
	0.3	0.0016	0.0024	0.0030
	0.4	0.0021	0.0029	0.0036
	0.5	0.0026	0.0033	0.0040
1 × 10^−4^	0.1	0.0039	0.0125	0.0158
	0.2	0.0028	0.0098	0.0135
	0.3	0.0041	0.0107	0.0161
	0.4	0.0055	0.0135	0.0175
	0.5	0.0062	0.0142	0.0182

We evaluated combinations of Learning Rates, Dropout rates, and Training Epochs (1–3). Bold indicates the best performance (1 × 10^−5^, 0.3, Epoch Num = 1), and underline indicates the second-best (5 × 10^−5^, 0.2, Epoch Num = 1).

For clustering, we systematically compared five algorithms: *K*-Means, agglomerative clustering, Gaussian mixture models (GMM), Spectral Clustering, and DBSCAN. For *K*-means and GMM, the number of clusters was varied from 2 to 10; for agglomerative clustering, we evaluated *n*_clusters_ = 2−10 with Ward linkage; for spectral clustering, *n*_clusters_ = 2−10 was tested; and for DBSCAN, we searched eps = 2.0–5.0 and min-samples = 5 or 10. Internal metrics, including Silhouette score, Calinski-–Harabasz score, and Davies–Bouldin score were used for model selection. The best performance was obtained with *K*-Means, *n*_clusters_ = 4, giving the highest Silhouette score (0.14), with competitive Calinski–Harabasz score (2137.26) and Davies–Bouldin score (1.97) (Table 12).

**Table 12:** Grid search results on the clustering task

Method	Hyperparameters	Inertia	Silhouette score	Calinski–Harabasz score	Davies–Bouldin score
Agglomerative	n_clusters = 2	/	0.07	1215.16	3.27
Agglomerative	n_clusters = 3	/	0.07	1260.77	2.94
Agglomerative	n_clusters = 4	/	0.07	1251.99	2.41
Agglomerative	n_clusters = 5	/	0.08	1254.99	2.75
Agglomerative	n_clusters = 6	/	0.08	1242.61	2.58
Agglomerative	n_clusters = 7	/	0.09	1231.17	2.40
Agglomerative	n_clusters = 8	/	0.08	1142.19	2.64
Agglomerative	n_clusters = 9	/	0.07	1072.42	2.50
Agglomerative	n_clusters = 10	/	0.08	1017.04	2.43
GMM	n_clusters = 2	/	0.10	2102.02	3.03
GMM	n_clusters = 3	/	0.10	2120.87	2.53
GMM	n_clusters = 4	/	0.11	2057.50	2.24
GMM	n_clusters = 5	/	0.13	2095.13	2.00
GMM	n_clusters = 6	/	0.12	1766.92	2.02
GMM	n_clusters = 7	/	0.11	1604.91	2.22
GMM	n_clusters = 8	/	0.11	1471.57	2.15
GMM	n_clusters = 9	/	0.11	1362.35	2.28
GMM	n_clusters = 10	/	0.09	1250.11	2.48
Spectral	n_clusters = 2	/	0.05	1246.10	2.37
Spectral	n_clusters = 3	/	0.06	1034.86	2.60
Spectral	n_clusters = 4	/	0.05	901.71	2.63
Spectral	n_clusters = 5	/	0.02	742.38	2.34
Spectral	n_clusters = 6	/	0.00	640.22	2.15
Spectral	n_clusters = 7	/	0.01	650.05	2.40
Spectral	n_clusters = 8	/	−0.02	606.33	2.29
Spectral	n_clusters = 9	/	−0.04	561.27	2.18
Spectral	n_clusters = 10	/	−0.03	587.30	2.11
DBSCAN	eps = 2.0, min_samples = 5	/	−0.01	32.69	0.93
DBSCAN	eps = 2.0, min_samples = 10	/	0.07	15.18	**0.88**
DBSCAN	eps = 2.5, min_samples = 5	/	−0.03	18.51	0.97
DBSCAN	eps = 2.5, min_samples = 10	/	−0.01	29.99	0.92
DBSCAN	eps = 3.0, min_samples = 5	/	−0.14	34.50	1.01
DBSCAN	eps = 3.0, min_samples = 10	/	−0.02	36.99	0.95
DBSCAN	eps = 3.5, min_samples = 5	/	−0.21	24.06	1.00
DBSCAN	eps = 3.5, min_samples = 10	/	0.00	72.34	0.98
DBSCAN	eps = 4.0, min_samples = 5	/	−0.25	19.76	1.04
DBSCAN	eps = 4.0, min_samples = 10	/	−0.14	52.37	1.11
DBSCAN	eps = 4.5, min_samples = 5	/	−0.28	19.12	1.11
DBSCAN	eps = 4.5, min_samples = 10	/	−0.18	57.47	1.09
DBSCAN	eps = 5.0, min_samples = 5	/	−0.29	23.09	1.15
DBSCAN	eps = 5.0, min_samples = 10	/	−0.20	41.51	1.12
K-Means	n_clusters = 2	2.70 × 10^7^	0.1	2072.10	2.92
K-Means	n_clusters = 3	2.35 × 10^7^	0.12	2099.00	2.52
K-Means	n_clusters = 5	1.95 × 10^7^	0.13	**2252.42**	1.99
K-Means	n_clusters = 6	1.88 × 10^7^	0.12	1881.51	2.13
K-Means	n_clusters = 7	1.83 × 10^7^	0.11	1718.94	2.25
K-Means	n_clusters = 8	1.80 × 10^7^	0.12	1585.66	2.06
K-Means	n_clusters = 9	1.78 × 10^7^	0.11	1477.50	2.14
*K*-Means	n_clusters = 10	1.77 × 10^7^	0.1	1381.95	2.15
*K*-Means	n_clusters = 4	2.05 × 10^7^	**0.14**	2137.26	1.97

Reported metrics are Silhouette score, Calinski–Harabasz score, and Davies–Bouldin score. Bold values indicate the best performance across all settings, and underlined values denote the second-best performance. The last row of the table is the setting we finally chose as our hyperparameter setting.

For the MLP classification head, grid search was expanded to cover a wider range of hyperparameters: threshold for variables selection (40%, 50%, 60%), learning rate (1e−4, 3e−4, 1e−3, 3e−3, 1e−2), weight decay (0, 1e−4, 1e−3, 5e−3, 1e−2), hidden size (256, 400, 512, 1024, 2048), and depth (1−4). The threshold for variable selection refers to the criterion used when choosing laboratory test variables as model inputs: if the proportion of patients who underwent a given test was below this threshold, that laboratory test was excluded from the study. Models were evaluated with AUC, AUPRC, F1, Balanced Accuracy, MCC, Specificity, Sensitivity, PPV, and NPV on validation splits, and the final settings were selected separately for each dataset. The optimal configurations were: GDHCM retrospective dataset (threshold = 50%, lr = 0.001, weight decay = 0.001, hidden size = 512, depth = 2), GDHCM prospective dataset (threshold = 50%, lr = 0.001, weight decay = 0.001, hidden size = 512, depth = 1), and SYSMH external validation dataset (threshold = 50%, lr = 0.001, wd = 0.0, hidden size = 256, depth = 1). These achieved the best overall ranking across discrimination and threshold-dependent metrics (Table 13). Due to space constraints, we report in the main text only the results of the final chosen configuration, while the complete results of all hyperparameter candidates are provided in Supplementary Tables 6–41.

**Table 13:** Grid search results on the classification task

Dataset	Config	AUC	AUPRC	F1
GDHCM retrospective	th50_lr0.001_wd0.001_h512_d2	0.83 [0.74, 0.93]	**0.56 [0.21, 0.77]**	0.37 [0.33, 0.50]
GDHCM prospective	th50_lr0.001_wd0.001_h512_d1	0.82 [0.64, 0.96]	**0.65 [0.25, 0.87]**	**0.54 [0.42, 0.75]**
SYSMH external	th50_lr0.001_wd0.0_h256_d1	0.69 [0.63, 0.76]	0.38 [0.35, 0.45]	**0.47 [0.37, 0.57]**
**Dataset**	**Config**	**Balanced Acc**	**MCC**	**Specificity**
GDHCM retrospective	th50_lr0.001_wd0.001_h512_d2	0.73 [0.61, 0.86]	0.33 [0.27, 0.49]	0.73 [0.48, 0.97]
GDHCM prospective	th50_lr0.001_wd0.001_h512_d1	**0.79 [0.68, 0.92]**	**0.48 [0.32, 0.70]**	0.73 [0.45, 0.95]
SYSMH external	th50_lr0.001_wd0.0_h256_d1	**0.65 [0.52, 0.75]**	**0.28 [0.10, 0.45]**	0.56 [0.04, 0.83]
**Dataset**	**Config**	**Sensitivity**	**PPV**	**NPV**
GDHCM retrospective	th50_lr0.001_wd0.001_h512_d2	0.72 [0.25, 1.00]	0.30 [0.21, 0.50]	0.96 [0.90, 1.00]
GDHCM prospective	th50_lr0.001_wd0.001_h512_d1	0.85 [0.50, 1.00]	0.44 [0.27, 0.75]	0.97 [0.89, 1.00]
SYSMH external	th50_lr0.001_wd0.0_h256_d1	0.74 [0.62, 1.00]	**0.36 [0.23, 0.50]**	0.91 [0.85, 1.00]

Reported metrics are AUC, AUPRC, F1, Balanced Accuracy, MCC, Specificity, Sensitivity, PPV, and NPV. Bold values indicate the best performance across all settings, and underlined values denote the second-best performance. Due to space constraints, we report in the main text only the results of the final chosen configuration, while the complete results of all hyperparameter candidates are provided in Supplementary Tables 6-14.

### Implementation details

During the embedding model training phase, we utilized AdamW^55^ as the default optimizer. We set the learning rate to 1 × 10^−5^. The model underwent 1 epoch of training. The batch size was set to 8. The dropout rate was set to 0.3. The input text truncation length was set to 512. During the classifier training phase, we also utilized AdamW^55^ as the default optimizer. The labeled data was evaluated using a *stratified five-fold cross-validation* protocol, in which the dataset was partitioned into five folds with an 80:20 split at each iteration. In every run, the model was trained on four folds (80%) and evaluated on the held-out fold (20%), rotating the validation fold across the five runs. The classification head consisted of an MLP whose hidden dimension (*h*) and depth (*d*) were tuned per dataset. The batch size was set to 64. Details on the selection of other related hyperparameters are provided in the section “Hyperparameter tuning”. Our model implementation was carried out using PyTorch^63^.

## Supplementary information

Supplementary information

## Data Availability

GDHCM dataset used to train SepsisDRM, and GDHCM retrospective dataset, GDHCM prospective dataset, SYSMH external validation dataset used to test SepsisDRM, are not publicly available due to its potentially identifiable nature.

## Code availability

To ensure long-term accessibility and facilitate reproducibility, the complete source code of SepsisDRM, along with the synthetically generated toy datasets and pre-trained model weights, has been archived on *Zenodo* with the persistent identifier 10.5281/zenodo.17828465. The repository includes detailed documentation and environment configuration files.
