# Author Correction: A multimodal embedding model for sepsis data representation

**DOI:** 10.1038/s41746-026-03127-x

**Published:** 2026-08-13

**Authors:** Tuo Liu, Yonglin Li, Hongyi Chen, Naiqing Li, Yan Zhang, Xuanqi Huang, Jin Wang, Rui Chen, Yuping Zeng, Yuntao Liu, Danwen Zheng, Darong Wu, Changdong Wang, Tao Yu, Xiaotu Xi, Zhongde Zhang

**Affiliations:** 1 School of Computer Science and Engineering, Sun Yat-sen University, Guangzhou, China; 2 Guangzhou University of Chinese Medicine, Guangzhou, China; 3 The Second Affiliated Hospital of Guangzhou University of Chinese Medicine (Guangdong Provincial Hospital of Chinese Medicine), Guangzhou, China; 4 Guangdong Provincial Key Laboratory of Research on Emergency in TCM, Guangzhou, China; 5 Department of Emergency Medicine, Sun Yat-sen Memorial Hospital, Sun Yat-sen University, Guangzhou, China; 6 Institute of Cardiopulmonary Cerebral Resuscitation, Sun Yat-Sen University, Guangzhou, China; 7 Information Management Office, The Second Affiliated Hospital of Guangzhou University of Chinese Medicine (Guangdong Provincial Hospital of Chinese Medicine), Guangzhou, China; 8 State Key Laboratory of Traditional Chinese Medicine Syndrome, Guangzhou, China

**Keywords:** Biomarkers, Computational biology and bioinformatics, Diseases, Mathematics and computing, Medical research

Correction to: *npj Digital Medicine* 10.1038/s41746-026-02446-3, published online 23 February 2026

In the original version of this article, the order of variables in the statistical output did not correspond to the row order in Tables 1 and 2. In addition, several unit labels and variable names were incorrect.

