# Supplementary material for: A multimodal embedding model for sepsis data representation
Source: NPJ Digit Med. 2026 Feb 23;9:272. doi: 10.1038/s41746-026-02446-3 (PMC13039152; doi:10.1038/s41746-026-02446-3)
Supplement: Supplementary file 1 — Supplementary information [file 41746_2026_2446_MOESM1_ESM.pdf]

A Multimodal Embedding Model for Sepsis Data Representation

Tuo Liu[1], Yonglin Li[2,3,4], Hongyi Chen[1], Naiqing Li[1], Yan Zhang[5,6], Xuanqi Huang[1], Jin Wang[2,3,4], Rui Chen[3,4], Yuping Zeng[7], Yuntao Liu[3,8], Danwen Zheng[3,4], Darong Wu[3,8], Changdong Wang[1], Tao Yu[5], Xiaotu Xi[3,4], Zhongde Zhang[3,8]*

[1]School of Computer Science and Engineering, Sun Yat-sen University, Guangzhou, China.
[2]Guangzhou University of Chinese Medicine, Guangzhou, China.
[3]The Second Affiliated Hospital of Guangzhou University of Chinese Medicine (Guangdong Provincial Hospital of Chinese Medicine), Guangzhou, China.
[4]Guangdong Provincial Key Laboratory of Research on Emergency in TCM, Guangzhou, China.
[5]Department of Emergency Medicine, Sun Yat-sen Memorial Hospital, Sun Yat-sen University, Guangzhou, China.
[6]Institute of Cardiopulmonary Cerebral Resuscitation, Sun Yat-Sen University, Guangzhou, China.
[7]Information Management Office, The Second Affiliated Hospital of Guangzhou University of Chinese Medicine (Guangdong Provincial Hospital of Chinese Medicine), Guangzhou, China.
[8]State Key Laboratory of Traditional Chinese Medicine Syndrome, Guangzhou, China.

*Corresponding author(s). E-mail(s): doctorzzd99@gzucm.edu.cn;
Contributing authors: liut259@mail2.sysu.edu.cn; liyonglin@gzucm.edu.cn; chenhy359@mail2.sysu.edu.cn; adslnq@mail.sysu.edu.cn; zhangy585@mail2.sysu.edu.cn; huangxq83@mail2.sysu.edu.cn; wangjin9953@163.com; happydecr@outlook.com; zyp8365@163.com; liuyuntao@gzucm.edu.cn; zhengdanwen@gzucm.edu.cn; darongwu@gzucm.edu.cn;

wangchd3@mail.sysu.edu.cn; yut@mail.sysu.edu.cn;
xxttm@gzucm.edu.cn;

1 Baselines Model Implementation Details

To evaluate the clustering capability of SepsisDRM, we conducted a series of unsupervised learning experiments using widely adopted clustering algorithms as baselines. The following methods cover partition-based, hierarchical, graph-based, probabilistic, and density-based paradigms.

`K-Means clustering` [1]. We use the classical partitioning algorithm that minimizes within-cluster variance, fixing the number of clusters at four in accordance with our study design.

`Agglomerative hierarchical clustering (Ward)` [2]. This bottom–up procedure iteratively merges clusters to optimize Ward's variance-based objective, enabling the capture of nested data structure while maintaining $k = 4$ clusters.

`Spectral clustering` [3]. We construct a nearest-neighbor affinity graph and perform clustering in the graph Laplacian eigenspace, which is effective for non-convex cluster geometry; the number of clusters is set to four and assignments are produced via K-Means in the embedded space.

`Gaussian Mixture Models (GMMs)` [4]. We adopt a probabilistic mixture of multivariate Gaussians (full covariance), yielding soft assignments that can accommodate heterogeneous cluster shapes; the number of components is fixed at four.

`DBSCAN` (Density-Based Spatial Clustering of Applications with Noise) [5]. We perform a small grid over ε and *min_samples* to approximate four meaningful clusters while retaining robustness to outliers; when the exact four-cluster solution is not available, the best configuration is selected based on internal validity.

Each method is evaluated on three feature types: (i) *tabular features* from structured numerical vectors, standardized by z-score; (ii) *text features* obtained from clinical notes using a pretrained RoBERTa model, with the final hidden representations aggregated at the document level and subsequently standardized; and (iii) *fusion features* constructed by concatenating tabular and text embeddings, followed by PCA for dimensionality control. We report the *Silhouette Score* [6], *Calinski-Harabasz Score* [7], and *Davies-Bouldin Score* [8] as the evaluation metrics.

To contextualize the effectiveness of our approach on classification task, we benchmark against a comprehensive suite of baselines that operate on (i) tabular variables only, (ii) free-text clinical notes only, and (iii) multimodal models that combine both sources. For neural models, we address class imbalance with focal loss [9]. For tree-based models, we use positive-class reweighting (e.g., `scale_pos_weight`). All evaluations are performed in 5-fold cross-validation using identical folds across baselines.

`Logistic Regression (LR).` A linear classifier on standardized tabular features with ℓ_2 regularization and inverse-frequency class weighting

(class_weight=balanced) [10]. This constitutes a strong, well-calibrated linear reference.

Multi-Layer Perceptron (MLP). A feed-forward network over continuous features with ReLU activations and dropout regularization, trained with AdamW and focal loss [9].

FT-Transformer. A transformer-style architecture for tabular data that projects continuous features to a token embedding space and processes them with self-attention [11]. We use a compact configuration (e.g., $d_{\text{model}} \approx 128$, 2 encoder layers, 4 heads) and a linear classification head.

TF--IDF + Logistic Regression. A sparse bag-of-n-grams representation (unigrams and bigrams) with term frequency–inverse document frequency normalization [12]; a logistic regression classifier with ℓ_2 regularization is trained on the TF–IDF features [10].

BERT Encoder (RoBERTa family). A transformer encoder initialized from a RoBERTa-style pretrained checkpoint and fine-tuned end-to-end for binary classification using the [CLS] representation [13, 14]. For Chinese clinical text we employ a whole-word masking RoBERTa variant [15]. Optimization uses AdamW with a linear warmup schedule.

BERT with Sliding-Window Aggregation. To accommodate long notes beyond the encoder's maximum context, each document is partitioned into overlapping windows; window-level posterior probabilities are computed independently and then mean-pooled to obtain the document-level score [16, 17]. Encoder and optimization settings follow the BERT baseline above.

Feature-Level Fusion (Frozen Encoder). We concatenate the text [CLS] embedding (text encoder kept frozen) with the normalized tabular vector and train a shallow MLP classifier on the joint representation. Feature-level fusion of this form is a widely used baseline in multimodal classification [18, 19].

Feature-Level Fusion (Jointly Finetuned Encoder). Same as above, but the text encoder is updated jointly with the fusion head, allowing the textual representation to adapt to the tabular context [18, 19].

Cross-Attention Fusion. To enable richer cross-modal interactions, we map the tabular vector into a small set of learned "pseudo-tokens" and append them to the sequence of text tokens. A transformer encoder layer then performs self-attention over the concatenated sequence, enabling the tabular tokens to attend to text (and vice versa); the resulting [CLS]-like representation is passed to a classifier head. This design follows established cross-attention paradigms in multimodal learning [20–22].

For neural baselines (text-only and multimodal), we employ focal loss [9]. Transformer-based models use AdamW with linear learning-rate warmup [23]. Tree-based methods apply scale_pos_weight according to the class ratio. All methods use identical cross-validation folds, and we report AUC, AUPRC, F1, Balanced Accuracy, MCC, Sensitivity, Specificity, PPV, and NPV.

2 Supplementary Figures

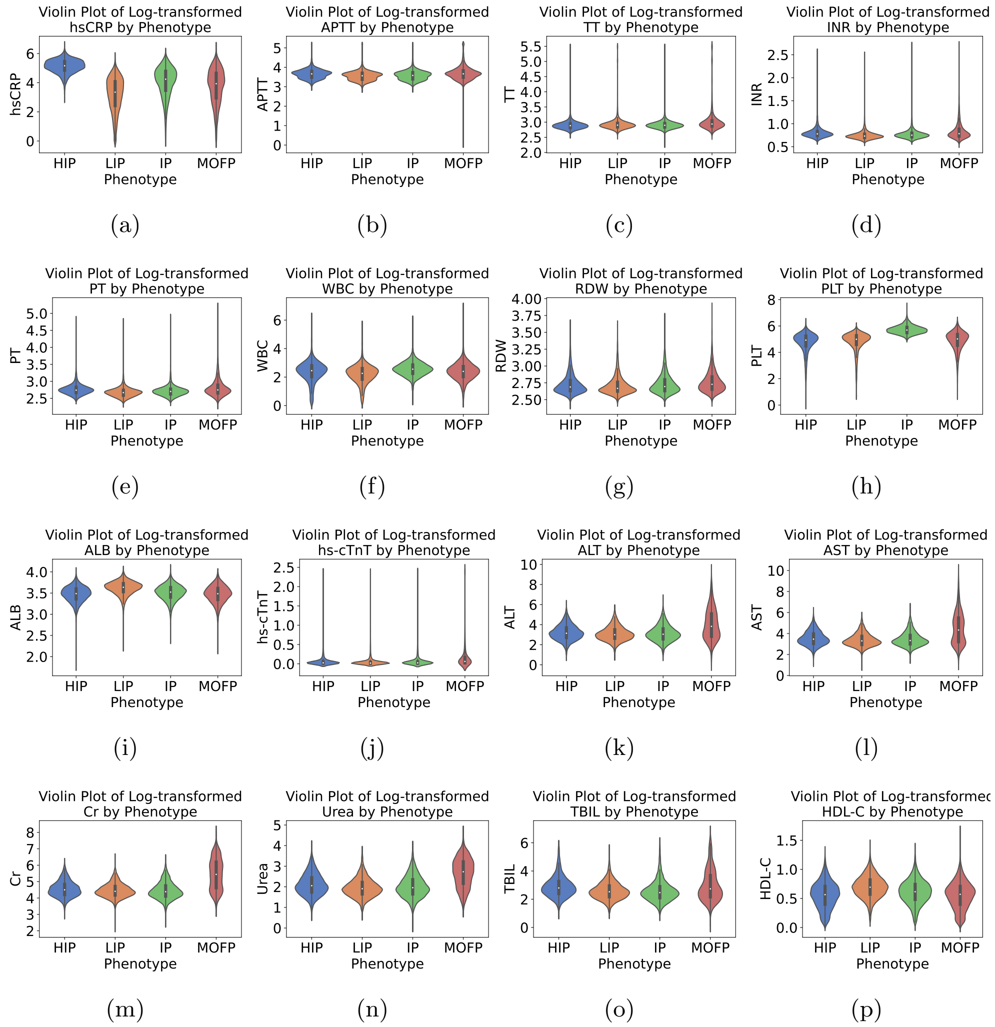

Supplementary Figure 1: This figure presents violin plots of key laboratory test results. Due to the significant variability in test results across patients, directly visualizing the data does not yield optimal results. Therefore, to better display the distributions, we applied a log transformation to the key blood test indices. Prior to this transformation, a constant of 1 was added to all values to avoid issues with log transformation of zero values. This approach reduces the range of the data, making the distribution more comparable and the visualization clearer.

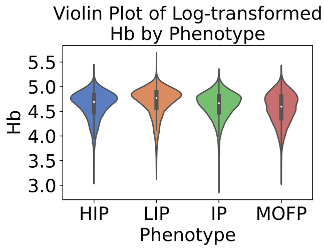

(a)

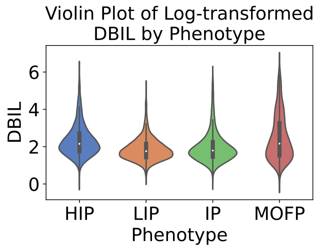

(b)

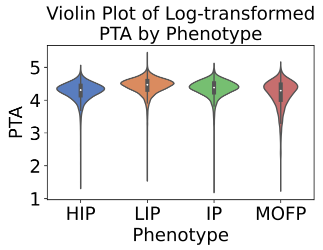

(c)

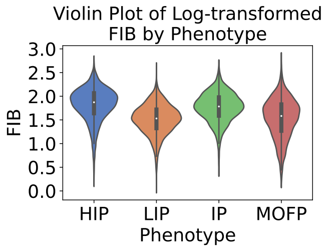

(d)

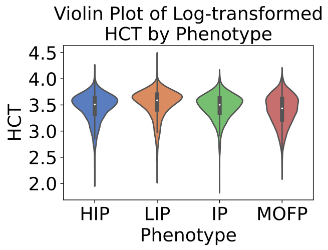

(e)

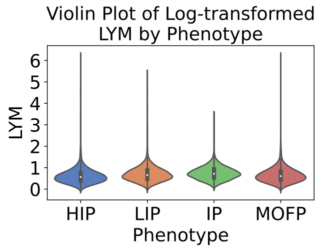

(f)

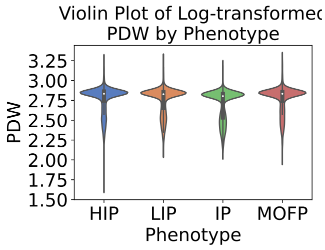

(g)

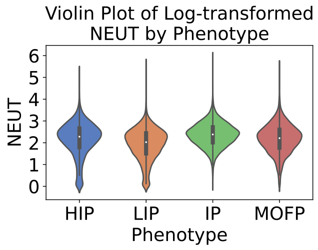

(h)

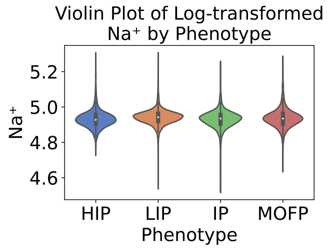

(i)

Supplementary Figure 2: This figure presents violin plots of remaining laboratory test results. Due to the significant variability in test results across patients, directly visualizing the data does not yield optimal results. Therefore, to better display the distributions, we applied a log transformation to the key blood test indices. Prior to this transformation, a constant of 1 was added to all values to avoid issues with log transformation of zero values. This approach reduces the range of the data, making the distribution more comparable and the visualization clearer.

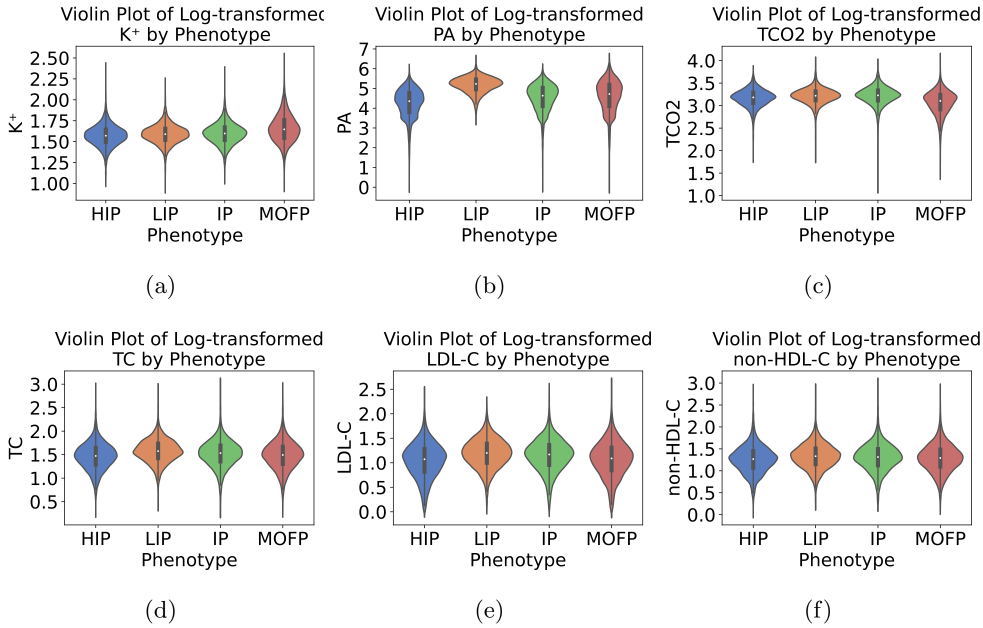

Supplementary Figure 3: This figure presents violin plots of remaining laboratory test results. Due to the significant variability in test results across patients, directly visualizing the data does not yield optimal results. Therefore, to better display the distributions, we applied a log transformation to the key blood test indices. Prior to this transformation, a constant of 1 was added to all values to avoid issues with log transformation of zero values. This approach reduces the range of the data, making the distribution more comparable and the visualization clearer.

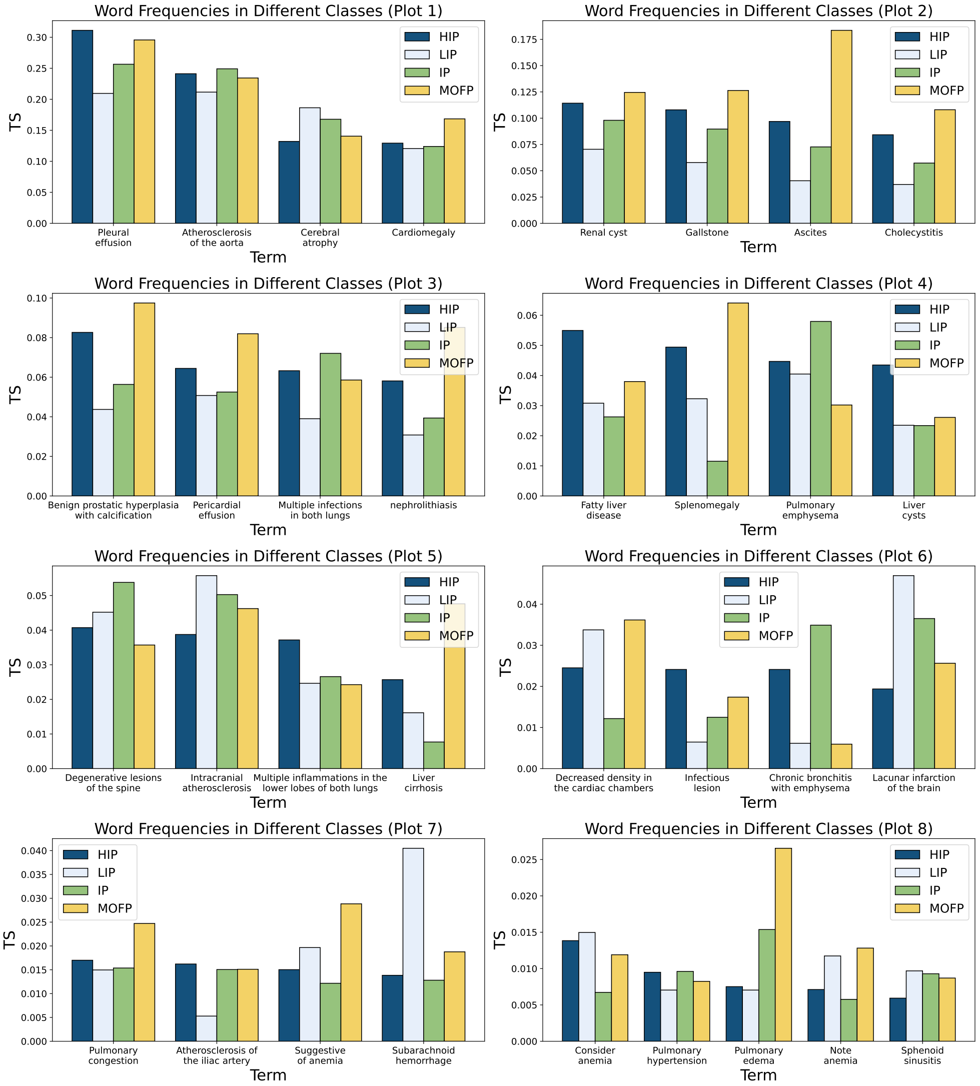

Supplementary Figure 4: A set of bar charts illustrating the frequencies of specific terms occurring in the textual data of patients with different phenotypes. These terms were frequently mentioned across the textual data of all patients and appeared in the textual data of patients from each of the four phenotypes.

3 Supplementary Tables

Abbreviation	Full Name
hsCRP	Hypersensitive C-reactive Protein
APTT	Activated Partial Thromboplastin Time
TT	Thrombin Time
INR	International Normalized Ratio
PTA	Prothrombin Activity
PT	Prothrombin Time
FIB	Fibrinogen
WBC	White Blood Cell Count
HCT	Hematocrit
RDW	Red Cell Distribution Width
LYM	Lymphocyte Count
Hb	Hemoglobin
PLT	Blood Platelet Count
hs-cTnT	Cardiac Troponin T
ALT	Alanine Aminotransferase
AST	Aspartate Aminotransferase
Cr	Creatinine
K+	Potassium Ion
Na+	Sodium Ion
Urea	Urea
PA	Prealbumin
DBIL	Direct Bilirubin
TBIL	Total Bilirubin
TCO2	Total Carbon Dioxide
TC	Total Cholesterol
LDL-C	Low-density Lipoprotein Cholesterol
non-HDL-C	Non-high-density Lipoprotein Cholesterol
HDL-C	High-density Lipoprotein Cholesterol

Supplementary Table 1: Comparison table between abbreviations and full names of laboratory test results.

Term Name	HIP	LIP	IP	MOFP
Aneurysmal dilation of the ascending aorta	0.0051	0.0053	0.0048	-
Ascites	0.0969	0.0405	0.0727	0.1835
Atherosclerosis of the aorta	0.2412	0.2116	0.2491	0.2344
Atherosclerosis of the iliac artery	0.0162	0.0053	0.0150	0.0151
Benign prostatic hyperplasia with calcification	0.0826	0.0437	0.0564	0.0975
Bilateral ethmoid sinusitis	-	0.0059	0.0048	-
Bilateral pleural thickening	0.0127	-	0.0141	0.0101
Bilateral small pleural effusions	-	-	0.0058	-
Biliary dilation	0.0067	-	0.0106	0.0069
Biliary stasis in the gallbladder	-	-	-	0.0064
Blood accumulation	0.0063	-	-	-
Blood accumulation in the ventricular system	-	0.0050	-	-
Brain swelling	-	0.0059	-	-
Cardiomegaly	0.1293	0.1206	0.1239	0.1684
Cerebral atrophy	0.1321	0.1863	0.1678	0.1405
Cholecystitis	0.0842	0.0370	0.0573	0.1080
Cholestasis	-	-	-	0.0096
Chronic bronchitis with emphysema	0.0241	0.0062	0.0349	0.0060
Consider anemia	0.0138	0.0150	0.0067	0.0119
Consider cyst	-	0.0091	-	0.0133
Consider inflammation	-	0.0173	0.0183	0.0156
Consider liver abscess	0.0087	-	-	-
Decreased density in the cardiac chambers	0.0245	0.0337	0.0122	0.0362
Degenerative lesions of the spine	0.0407	0.0452	0.0538	0.0357
Enlarged mediastinal lymph nodes	0.0059	-	0.0134	-
Ethmoid sinusitis	-	0.0079	-	-
Fatty liver disease	0.0550	0.0308	0.0263	0.0380
Gallbladder enlargement	0.0063	-	0.0051	0.0073
Gallstone	0.1079	0.0578	0.0897	0.1263
Gastric varices	-	-	-	0.0101
Heart failure	-	0.0076	-	0.0165
Hemorrhage in the corona radiata	-	0.0050	-	-
Hemorrhage into the ventricular system	-	0.0047	-	-
Hydrocephalus	-	-	0.0058	-
Incomplete aeration of both lower lungs	0.0087	-	0.0150	-
Incomplete expansion of the lower lobes of both lungs	0.0063	0.0053	-	-
Infectious lesion	0.0241	0.0065	0.0125	0.0174
Intestinal stasis	0.0055	-	-	-
Intracranial atherosclerosis	0.0387	0.0558	0.0503	0.0462

Supplementary Table 2: The specific term significance (TS) of high-frequency words in the patients' textual data.

Term Name	HIP	LIP	IP	MOFP
Lacunar infarction of the brain	0.0194	0.0469	0.0365	0.0256
Left basal ganglia hemorrhage	-	0.0062	-	-
Left pleural thickening	-	-	0.0070	-
Liver cirrhosis	0.0257	0.0161	0.0077	0.0476
Liver cysts	0.0435	0.0235	0.0234	0.0261
Metastases	0.0332	-	0.0359	0.0101
Mild atelectasis due to adjacent lung compression	-	-	0.0051	-
Multiple infections in both lungs	0.0633	0.0390	0.0720	0.0586
Multiple inflammations in the lower lobes of both lungs	0.0372	0.0246	0.0266	0.0243
Multiple lung metastases in both lungs	-	-	0.0061	-
Multiple mediastinal lymph nodes	0.0107	-	0.0070	-
Multiple pulmonary bullae in both lungs	0.0071	0.0053	0.0080	-
Multiple small mediastinal lymph nodes	0.0119	0.0103	0.0131	-
Multiple small retroperitoneal lymph nodes	-	-	-	0.0069
nephrolithiasis	0.0581	0.0308	0.0394	0.0851
Note anemia	0.0071	0.0117	0.0058	0.0128
Osteoporosis	0.0158	0.0053	0.0086	-
Partly chronic inflammation	-	-	-	0.0073
Pericardial effusion	0.0644	0.0508	0.0525	0.0819
Peritonitis	0.0055	-	-	-
Periventricular white matter degeneration	0.0712	-	-	-
Periventricular white matter rarefaction	0.0127	0.0194	-	-
Pleural effusion	0.3112	0.2095	0.2565	0.2957
Portal hypertension	0.0079	0.0067	-	0.0179
Pulmonary congestion	0.0170	0.0150	0.0154	0.0247
Pulmonary edema	0.0075	0.0070	0.0154	0.0265
Pulmonary emphysema	0.0447	0.0405	0.0580	0.0302
Pulmonary hypertension	0.0095	0.0070	0.0096	0.0082
Renal atrophy	-	-	-	0.0554
Renal cyst	0.1143	0.0704	0.0980	0.1245
Right inguinal hernia	0.0055	-	-	-
Sphenoid sinusitis	0.0059	0.0097	0.0093	0.0087
Splenomegaly	0.0494	0.0323	0.0115	0.0641
Subarachnoid hemorrhage	0.0138	0.0405	0.0128	0.0188
Subfalcine herniation	-	0.0050	-	-
Suggestive of anemia	0.0150	0.0197	0.0122	0.0288
Thickened gallbladder wall	-	-	-	0.0078
Thickening of both adrenal glands	0.0087	-	0.0096	-
Thickening of the left adrenal gland	0.0138	-	0.0058	0.0105
Thyroid changes	0.0063	0.0062	-	0.0114

Supplementary Table 3: The specific term significance (TS) of high-frequency words in the patients' textual data.

Physicians	Sex	Age	Degree	AUC Score
Resident 1	Male	28	MD	0.74
Resident 2	Male	30	MM	0.57
Attending 1	Male	36	MD	0.61
Attending 2	Female	35	MD	0.61
Attending 3	Male	41	MM	0.67
Attending 4	Male	32	MM	0.58
Attending 5	Male	33	MM	0.57
Consulting 1	Male	45	MD	0.62
Consulting 2	Male	40	MM	0.60
Consulting 3	Male	40	MD	0.69
Consulting 4	Male	41	MM	0.71

Supplementary Table 4: The detailed information about the human experts and their AUC scores. The *Physicians* column lists the identifiers of the different experts, along with their respective professional ranks. The *Degree* column indicates the highest academic degree of each expert, where *MM* stands for Master of Medicine and *MD* stands for Doctor of Medicine. The *AUC Score* column shows their performance in the task of predicting the 28-day outcomes for sepsis patients.

Lab Tests	Conut	Percentage (%)	Lab Tests	Conut	Percentage (%)
Hypersensitive C-reactive Protein (hsCRP)	32156	50.44	B-Type Natriuretic Peptide (BNP)	24496	38.43
Activated Partial Thromboplastin Time (APTT)	55165	86.53	C-Reactive Protein (CRP)	14845	23.29
Thrombin Time (TT)	40881	64.13	Thyroid-Stimulating Hormone (TSH)	20466	32.10
International Normalized Ratio (INR)	55150	86.51	Triglyceride (TG)	24518	38.46
Prothrombin Activity (PTA)	33643	52.77	Triiodothyronine (T3)	19577	30.71
Prothrombin Time (PT)	55168	86.54	Thyroxine (T4)	19016	29.83
Fibrinogen (FIB)	55162	86.53	Free Triiodothyronine (FT3)	16591	26.03
White Blood Cell Count (WBC)	60103	94.28	Free Thyroxine (FT4)	16102	25.26
Hematocrit (HCT)	60103	94.28	Cardiac Troponin I (cTnI)	2747	4.31
Red Cell Distribution Width (RDW)	60089	94.26	N-Terminal Pro B-Type Natriuretic Peptide (NT-ProBNP)	11717	18.38
Lymphocyte Count (LYM)	52969	83.09	D-Dimer (DDi)	29137	45.71
Hemoglobin (Hb)	60108	94.29	Procalcitonin (PCT)	23906	37.50
Blood Platelet Count (PLT)	60102	94.28	Chloride Ion (Cl+)	30951	48.55
Platelet Distribution Width (PDW)	59228	92.91	Lactate Dehydrogenase (LDH)	22458	35.23
Neutrophil Count (NEUT)	52969	83.09	Anion Gap (AG)	25453	39.93
Albumin (ALB)	54535	85.55	Erythrocyte Sedimentation Rate (ESR)	10484	16.45
High-sensitivity Cardiac Troponin T (hs-cTnT)	34120	53.52	Partial Pressure of Carbon Dioxide (PCO2)	30138	47.28
Alanine Aminotransferase (ALT)	54471	85.45	Blood Lactate (Lac)	29782	46.72
Aspartate Aminotransferase (AST)	56371	88.43	Partial Pressure of Oxygen (PO2)	30137	47.27
Creatinine (Cr)	59406	93.19	High-Sensitivity Cardiac Troponin I (hs-cTnI)	5172	8.11
Potassium Ion (K+)	58755	92.17			
Sodium Ion (Na+)	58709	92.09			
Urea	59377	93.14			
Prealbumin (PA)	32525	51.02			
Direct Bilirubin (DBIL)	45452	71.30			
Total Bilirubin (TBIL)	52958	83.07			
Total Carbon Dioxide (TCO2)	58626	91.96			
Total Cholesterol (TC)	37368	58.62			
Low-density Lipoprotein Cholesterol (LDL-C)	37103	58.20			
high-density Lipoprotein Cholesterol (non-HDL-C)	33058	51.86			
High-density Lipoprotein Cholesterol (HDL-C)	37331	58.56			

Supplementary Table 5: The availability rate of all laboratory test indicators including the 31 indicators used in the study and the remaining 21 indicators removed due to the serious missing records. *Count* refers to the number of patients who have this laboratory test indicator, and *Percentage* represents the proportion of these patients out of the total number of patients, which refers to the 64,157 patients after excluding 35,320 hospitalization records that are not the first sepsis-related hospitalization for a given patient.

Config	AUC	AUPRC	F1	Balanced_Accuracy	MCC	Specificity	Sensitivity	PPV	NPV
lr0.0001_wd0.0_h256_d1	0.77 [0.67, 0.88]	0.44 [0.17, 0.53]	0.34 [0.15, 0.67]	0.64 [0.51, 0.84]	0.23 [0.01, 0.62]	0.73 [0.54, 0.93]	0.55 [0.33, 0.75]	0.27 [0.10, 0.60]	0.93 [0.90, 0.96]
lr0.0001_wd0.0_h400_d1	0.80 [0.69, 0.89]	0.49 [0.38, 0.55]	0.30 [0.16, 0.50]	0.64 [0.48, 0.79]	0.20 [-0.03, 0.44]	0.61 [0.29, 0.86]	0.67 [0.25, 1.00]	0.21 [0.09, 0.38]	0.94 [0.89, 1.00]
lr0.0001_wd0.0_h512_d1	0.75 [0.62, 0.88]	0.43 [0.15, 0.55]	0.27 [0.14, 0.40]	0.60 [0.49, 0.74]	0.15 [-0.01, 0.33]	0.73 [0.64, 0.79]	0.48 [0.33, 0.75]	0.19 [0.09, 0.27]	0.92 [0.90, 0.95]
lr0.0001_wd0.0_h256_d2	0.76 [0.65, 0.90]	0.41 [0.19, 0.56]	0.31 [0.00, 0.50]	0.64 [0.39, 0.79]	0.21 [-0.16, 0.44]	0.79 [0.61, 0.86]	0.48 [0.00, 0.75]	0.24 [0.00, 0.38]	0.93 [0.88, 0.96]
lr0.0001_wd0.0_h400_d2	0.76 [0.63, 0.91]	0.45 [0.17, 0.60]	0.23 [0.00, 0.40]	0.55 [0.45, 0.68]	0.09 [-0.11, 0.31]	0.57 [0.00, 0.89]	0.53 [0.00, 1.00]	0.17 [0.00, 0.33]	0.73 [0.00, 0.93]
lr0.0001_wd0.0_h512_d2	0.76 [0.64, 0.87]	0.41 [0.19, 0.54]	0.36 [0.17, 0.67]	0.64 [0.52, 0.84]	0.26 [0.03, 0.62]	0.85 [0.71, 0.93]	0.43 [0.25, 0.75]	0.32 [0.11, 0.60]	0.92 [0.89, 0.96]
lr0.0001_wd0.0001_h256_d1	0.75 [0.61, 0.88]	0.42 [0.17, 0.53]	0.28 [0.00, 0.46]	0.63 [0.45, 0.77]	0.20 [-0.11, 0.40]	0.70 [0.17, 0.93]	0.57 [0.00, 1.00]	0.21 [0.00, 0.33]	0.95 [0.89, 1.00]
lr0.0001_wd0.0001_h400_d1	0.77 [0.63, 0.89]	0.45 [0.22, 0.55]	0.31 [0.18, 0.46]	0.63 [0.53, 0.77]	0.20 [0.04, 0.40]	0.72 [0.39, 0.86]	0.55 [0.33, 0.75]	0.22 [0.11, 0.33]	0.93 [0.91, 0.96]
lr0.0001_wd0.0001_h512_d1	0.76 [0.63, 0.86]	0.38 [0.17, 0.51]	0.35 [0.00, 0.60]	0.67 [0.41, 0.82]	0.27 [-0.14, 0.55]	0.82 [0.66, 0.93]	0.52 [0.00, 0.75]	0.28 [0.00, 0.50]	0.94 [0.88, 0.96]
lr0.0001_wd0.0001_h256_d2	0.78 [0.65, 0.90]	0.46 [0.16, 0.68]	0.28 [0.00, 0.55]	0.62 [0.46, 0.81]	0.19 [-0.09, 0.49]	0.87 [0.79, 0.93]	0.37 [0.00, 0.75]	0.24 [0.00, 0.43]	0.92 [0.90, 0.96]
lr0.0001_wd0.0001_h400_d2	0.78 [0.65, 0.89]	0.46 [0.21, 0.67]	0.31 [0.00, 0.55]	0.64 [0.45, 0.81]	0.21 [-0.11, 0.49]	0.85 [0.79, 0.89]	0.42 [0.00, 0.75]	0.24 [0.00, 0.43]	0.92 [0.89, 0.96]
lr0.0001_wd0.0001_h512_d2	0.78 [0.68, 0.88]	0.41 [0.21, 0.49]	0.31 [0.17, 0.57]	0.61 [0.50, 0.73]	0.18 [0.00, 0.53]	0.54 [0.00, 0.97]	0.67 [0.50, 1.00]	0.26 [0.10, 0.67]	0.74 [0.00, 0.95]
lr0.0001_wd0.001_h256_d1	0.78 [0.70, 0.88]	0.43 [0.27, 0.60]	0.34 [0.22, 0.50]	0.71 [0.58, 0.86]	0.28 [0.12, 0.49]	0.66 [0.46, 0.82]	0.77 [0.33, 1.00]	0.23 [0.17, 0.33]	0.96 [0.92, 1.00]
lr0.0001_wd0.001_h400_d1	0.76 [0.61, 0.88]	0.44 [0.15, 0.55]	0.33 [0.17, 0.57]	0.62 [0.52, 0.73]	0.23 [0.03, 0.53]	0.70 [0.41, 0.97]	0.55 [0.33, 0.75]	0.29 [0.11, 0.67]	0.93 [0.91, 0.93]
lr0.0001_wd0.001_h512_d1	0.82 [0.64, 0.88]	0.43 [0.33, 0.52]	0.30 [0.22, 0.38]	0.65 [0.57, 0.72]	0.22 [0.14, 0.30]	0.52 [0.14, 0.83]	0.78 [0.50, 1.00]	0.20 [0.12, 0.29]	**0.97 [0.92, 1.00]**
lr0.0001_wd0.001_h256_d2	0.79 [0.65, 0.87]	0.41 [0.18, 0.55]	0.25 [0.00, 0.46]	0.58 [0.41, 0.77]	0.12 [-0.14, 0.40]	0.65 [0.00, 0.83]	0.52 [0.00, 1.00]	0.18 [0.00, 0.33]	0.74 [0.00, 0.96]
lr0.0001_wd0.001_h400_d2	0.82 [0.69, 0.96]	0.53 [0.25, 0.79]	0.24 [0.00, 0.36]	0.60 [0.45, 0.71]	0.14 [-0.11, 0.27]	0.57 [0.00, 0.89]	0.63 [0.00, 1.00]	0.16 [0.00, 0.29]	0.75 [0.00, 1.00]
lr0.0001_wd0.001_h512_d2	0.77 [0.67, 0.89]	0.45 [0.18, 0.60]	0.28 [0.00, 0.57]	0.59 [0.39, 0.73]	0.16 [-0.16, 0.53]	0.80 [0.66, 0.97]	0.37 [0.00, 0.50]	0.25 [0.00, 0.67]	0.91 [0.88, 0.93]
lr0.0003_wd0.0_h256_d1	0.77 [0.65, 0.91]	0.45 [0.18, 0.61]	0.38 [0.25, 0.60]	0.70 [0.64, 0.82]	0.31 [0.16, 0.55]	0.72 [0.43, 0.90]	0.68 [0.50, 1.00]	0.29 [0.15, 0.50]	0.95 [0.92, 1.00]
lr0.0003_wd0.0_h400_d1	0.81 [0.74, 0.88]	0.47 [0.25, 0.73]	0.41 [0.27, 0.67]	0.72 [0.61, 0.84]	**0.35 [0.16, 0.62]**	0.76 [0.43, 0.93]	0.68 [0.50, 1.00]	0.32 [0.16, 0.60]	0.95 [0.91, 1.00]
lr0.0003_wd0.0_h512_d1	0.74 [0.61, 0.88]	0.39 [0.18, 0.52]	0.35 [0.13, 0.67]	0.65 [0.47, 0.84]	0.25 [-0.04, 0.62]	0.80 [0.61, 0.93]	0.50 [0.25, 0.75]	0.29 [0.08, 0.60]	0.93 [0.89, 0.96]
lr0.0003_wd0.0_h256_d2	0.80 [0.68, 0.91]	0.50 [0.23, 0.73]	0.31 [0.00, 0.50]	0.62 [0.45, 0.72]	0.22 [-0.11, 0.43]	**0.88 [0.82, 0.93]**	0.37 [0.00, 0.50]	0.28 [0.00, 0.50]	0.92 [0.89, 0.93]
lr0.0003_wd0.0_h400_d2	0.80 [0.68, 0.96]	0.50 [0.23, 0.86]	0.39 [0.25, 0.60]	0.70 [0.57, 0.82]	0.32 [0.15, 0.55]	0.84 [0.75, 0.90]	0.57 [0.25, 0.75]	0.31 [0.22, 0.50]	0.94 [0.90, 0.96]
lr0.0003_wd0.0_h512_d2	0.79 [0.68, 0.91]	0.44 [0.18, 0.58]	0.34 [0.00, 0.67]	0.64 [0.38, 0.84]	0.24 [-0.18, 0.62]	0.82 [0.75, 0.93]	0.47 [0.00, 0.75]	0.28 [0.00, 0.60]	0.93 [0.88, 0.96]
lr0.0003_wd0.0001_h256_d1	0.83 [0.73, 0.91]	0.48 [0.29, 0.58]	0.35 [0.27, 0.46]	0.72 [0.66, 0.77]	0.30 [0.26, 0.40]	0.65 [0.43, 0.83]	0.80 [0.50, 1.00]	0.24 [0.16, 0.33]	**0.97 [0.92, 1.00]**
lr0.0003_wd0.0001_h400_d1	0.78 [0.62, 0.89]	0.39 [0.16, 0.55]	0.32 [0.25, 0.43]	0.70 [0.57, 0.78]	0.27 [0.15, 0.36]	0.66 [0.46, 0.90]	0.73 [0.25, 1.00]	0.22 [0.17, 0.30]	0.96 [0.90, 1.00]
lr0.0003_wd0.0001_h512_d1	0.78 [0.62, 0.91]	0.39 [0.15, 0.56]	0.27 [0.00, 0.43]	0.61 [0.39, 0.75]	0.16 [-0.16, 0.36]	0.74 [0.57, 0.83]	0.48 [0.00, 0.75]	0.19 [0.00, 0.30]	0.92 [0.88, 0.96]
lr0.0003_wd0.0001_h256_d2	0.79 [0.69, 0.86]	0.44 [0.19, 0.58]	0.31 [0.16, 0.44]	0.60 [0.48, 0.70]	0.20 [-0.03, 0.36]	0.76 [0.29, 0.97]	0.45 [0.25, 0.67]	0.29 [0.09, 0.50]	0.91 [0.89, 0.93]
lr0.0003_wd0.0001_h400_d2	0.78 [0.65, 0.89]	0.51 [0.17, 0.75]	0.36 [0.25, 0.46]	0.69 [0.60, 0.77]	0.28 [0.15, 0.40]	0.78 [0.61, 0.86]	0.60 [0.33, 0.75]	0.26 [0.15, 0.33]	0.94 [0.92, 0.96]
lr0.0003_wd0.0001_h512_d2	0.78 [0.63, 0.91]	0.44 [0.23, 0.54]	0.36 [0.20, 0.67]	0.68 [0.56, 0.84]	0.28 [0.08, 0.62]	0.73 [0.38, 0.93]	0.63 [0.33, 1.00]	0.27 [0.14, 0.60]	0.95 [0.92, 1.00]
lr0.0003_wd0.001_h256_d1	0.78 [0.63, 0.90]	0.41 [0.16, 0.58]	0.30 [0.00, 0.55]	0.62 [0.39, 0.81]	0.19 [-0.16, 0.49]	0.76 [0.46, 0.86]	0.48 [0.00, 0.75]	0.23 [0.00, 0.43]	0.92 [0.88, 0.96]
lr0.0003_wd0.001_h400_d1	0.78 [0.65, 0.91]	0.42 [0.20, 0.57]	0.40 [0.18, 0.75]	0.69 [0.53, 0.86]	0.32 [0.04, 0.72]	0.70 [0.39, 0.97]	0.68 [0.50, 1.00]	0.33 [0.11, 0.75]	0.95 [0.92, 1.00]
lr0.0003_wd0.001_h512_d1	0.79 [0.62, 0.89]	0.49 [0.30, 0.64]	0.39 [0.20, 0.67]	0.69 [0.56, 0.84]	0.31 [0.08, 0.62]	0.79 [0.66, 0.93]	0.60 [0.33, 0.75]	0.31 [0.14, 0.60]	0.94 [0.92, 0.96]
lr0.0003_wd0.001_h256_d2	0.80 [0.73, 0.87]	0.43 [0.22, 0.54]	0.41 [0.25, 0.57]	0.70 [0.60, 0.74]	0.34 [0.15, 0.53]	0.85 [0.72, 0.97]	0.55 [0.33, 0.75]	**0.36 [0.20, 0.67]**	0.94 [0.92, 0.96]
lr0.0003_wd0.001_h400_d2	0.80 [0.68, 0.89]	0.41 [0.23, 0.55]	0.36 [0.18, 0.50]	0.70 [0.54, 0.79]	0.29 [0.06, 0.44]	0.71 [0.45, 0.83]	0.70 [0.33, 1.00]	0.24 [0.12, 0.38]	0.96 [0.91, 1.00]
lr0.0003_wd0.001_h512_d2	0.80 [0.67, 0.89]	0.44 [0.34, 0.53]	0.41 [0.24, 0.60]	0.73 [0.66, 0.82]	0.34 [0.21, 0.55]	0.73 [0.32, 0.90]	0.73 [0.50, 1.00]	0.30 [0.14, 0.50]	0.96 [0.92, 1.00]
lr0.001_wd0.0_h256_d1	0.78 [0.72, 0.87]	0.39 [0.24, 0.51]	0.33 [0.19, 0.55]	0.65 [0.54, 0.81]	0.24 [0.09, 0.49]	0.59 [0.07, 0.86]	0.72 [0.33, 1.00]	0.23 [0.10, 0.43]	0.96 [0.92, 1.00]
lr0.001_wd0.0_h400_d1	0.79 [0.68, 0.88]	0.34 [0.17, 0.48]	0.21 [0.00, 0.46]	0.59 [0.39, 0.77]	0.12 [-0.16, 0.40]	0.63 [0.21, 0.93]	0.55 [0.00, 1.00]	0.13 [0.00, 0.33]	0.95 [0.88, 1.00]
lr0.001_wd0.0_h512_d1	0.79 [0.74, 0.83]	0.42 [0.29, 0.60]	0.35 [0.29, 0.46]	0.70 [0.65, 0.77]	0.28 [0.21, 0.40]	0.73 [0.66, 0.79]	0.67 [0.50, 0.75]	0.24 [0.18, 0.33]	0.95 [0.92, 0.96]
lr0.001_wd0.0_h256_d2	0.77 [0.70, 0.86]	0.32 [0.20, 0.41]	0.33 [0.27, 0.43]	0.67 [0.59, 0.75]	0.25 [0.19, 0.36]	0.78 [0.64, 0.93]	0.57 [0.25, 0.75]	0.25 [0.17, 0.33]	0.94 [0.90, 0.96]
lr0.001_wd0.0_h400_d2	0.79 [0.67, 0.88]	0.34 [0.19, 0.51]	0.29 [0.00, 0.67]	0.64 [0.50, 0.84]	0.21 [0.00, 0.62]	0.65 [0.00, 1.00]	0.62 [0.00, 1.00]	0.22 [0.00, 0.60]	0.76 [0.00, 1.00]
lr0.001_wd0.0_h512_d2	**0.84 [0.74, 0.94]**	0.50 [0.30, 0.71]	0.32 [0.20, 0.42]	0.70 [0.57, 0.81]	0.27 [0.08, 0.41]	0.57 [0.43, 0.83]	**0.83 [0.50, 1.00]**	0.21 [0.12, 0.29]	**0.97 [0.92, 1.00]**
lr0.001_wd0.0001_h256_d1	0.78 [0.65, 0.91]	0.45 [0.33, 0.53]	0.37 [0.22, 0.60]	0.71 [0.58, 0.82]	0.32 [0.12, 0.55]	0.71 [0.48, 0.90]	0.72 [0.33, 1.00]	0.28 [0.17, 0.50]	0.96 [0.92, 1.00]
lr0.001_wd0.0001_h400_d1	0.83 [0.75, 0.90]	0.45 [0.23, 0.69]	0.38 [0.27, 0.50]	**0.74 [0.69, 0.79]**	0.33 [0.24, 0.44]	0.66 [0.43, 0.83]	**0.83 [0.67, 1.00]**	0.25 [0.16, 0.38]	**0.97 [0.95, 1.00]**
lr0.001_wd0.0001_h512_d1	0.83 [0.65, 0.92]	0.47 [0.35, 0.58]	0.33 [0.22, 0.46]	0.68 [0.58, 0.79]	0.26 [0.12, 0.40]	0.79 [0.57, 0.93]	0.57 [0.25, 1.00]	0.26 [0.17, 0.33]	0.94 [0.90, 1.00]
lr0.001_wd0.0001_h256_d2	0.81 [0.74, 0.90]	0.42 [0.21, 0.63]	0.34 [0.25, 0.46]	0.69 [0.59, 0.77]	0.27 [0.13, 0.40]	0.72 [0.50, 0.86]	0.67 [0.33, 1.00]	0.24 [0.18, 0.33]	0.95 [0.91, 1.00]
lr0.001_wd0.0001_h400_d2	0.81 [0.69, 0.91]	0.44 [0.25, 0.55]	0.25 [0.00, 0.53]	0.62 [0.50, 0.88]	0.18 [0.00, 0.53]	0.56 [0.03, 1.00]	0.67 [0.00, 1.00]	0.17 [0.00, 0.36]	**0.97 [0.90, 1.00]**
lr0.001_wd0.0001_h512_d2	0.82 [0.70, 0.89]	0.48 [0.37, 0.59]	0.35 [0.20, 0.50]	0.68 [0.57, 0.79]	0.26 [0.08, 0.44]	0.70 [0.46, 0.83]	0.67 [0.50, 0.75]	0.24 [0.12, 0.38]	0.94 [0.92, 0.96]
lr0.001_wd0.001_h256_d1	0.82 [0.68, 0.90]	0.42 [0.20, 0.57]	0.32 [0.00, 0.44]	0.66 [0.50, 0.75]	0.26 [0.00, 0.36]	0.86 [0.72, 1.00]	0.47 [0.00, 0.75]	0.26 [0.00, 0.40]	0.93 [0.90, 0.96]
lr0.001_wd0.001_h400_d1	0.83 [0.65, 0.90]	0.51 [0.32, 0.61]	0.38 [0.27, 0.55]	0.73 [0.68, 0.81]	0.33 [0.24, 0.49]	0.68 [0.43, 0.86]	0.78 [0.50, 1.00]	0.27 [0.16, 0.43]	**0.97 [0.93, 1.00]**
lr0.001_wd0.001_h512_d1	0.81 [0.70, 0.91]	0.49 [0.30, 0.76]	0.32 [0.17, 0.50]	0.66 [0.50, 0.79]	0.24 [0.01, 0.44]	0.75 [0.46, 0.90]	0.57 [0.25, 1.00]	0.25 [0.12, 0.40]	0.94 [0.88, 1.00]
lr0.001_wd0.001_h256_d2	0.81 [0.68, 0.91]	0.52 [0.19, 0.71]	**0.42 [0.20, 0.67]**	0.72 [0.56, 0.84]	**0.35 [0.08, 0.62]**	0.75 [0.38, 0.93]	0.70 [0.33, 1.00]	0.32 [0.14, 0.60]	0.96 [0.92, 1.00]
lr0.001_wd0.001_h400_d2	0.78 [0.64, 0.88]	0.38 [0.16, 0.48]	0.35 [0.20, 0.55]	0.65 [0.57, 0.81]	0.27 [0.13, 0.49]	0.74 [0.14, 0.97]	0.57 [0.25, 1.00]	0.31 [0.11, 0.50]	0.94 [0.90, 1.00]
**lr0.001_wd0.001_h512_d2**	0.83 [0.74, 0.93]	**0.56 [0.21, 0.77]**	0.37 [0.33, 0.50]	0.73 [0.61, 0.86]	0.33 [0.27, 0.49]	0.73 [0.48, 0.97]	0.72 [0.25, 1.00]	0.30 [0.21, 0.50]	0.96 [0.90, 1.00]
Rank	2	1	6	2	3	13	6	5	2

Supplementary Table 6: Grid search results on the classification task on GDHCM retrospective dataset (choosing 50% as the threshold for clinical variables selection). Reported metrics are AUC, AUPRC, F1, Balanced Accuracy, MCC, Specificity, Sensitivity, PPV, and NPV. Bold values indicate the best performance across all settings, and underlined values denote the second-best performance. The second-to-last row corresponds to the hyperparameter configuration we selected. The row labeled *Rank* indicates the relative position of the selected hyperparameter configuration among all candidate settings based on its validation performance across each metric.

Config	AUC	AUPRC	F1	Balanced_Accuracy	MCC	Specificity	Sensitivity	PPV	NPV
lr1e-05_wd0.005_h1024_d3	0.73 [0.63, 0.87]	0.33 [0.16, 0.49]	0.20 [0.00, 0.32]	0.57 [0.50, 0.77]	0.08 [0.00, 0.32]	0.44 [0.00, 1.00]	0.70 [0.00, 1.00]	0.12 [0.00, 0.19]	0.56 [0.00, 1.00]
lr1e-05_wd0.005_h2048_d3	0.74 [0.63, 0.84]	0.34 [0.22, 0.43]	0.22 [0.00, 0.43]	0.58 [0.50, 0.75]	0.11 [0.00, 0.36]	0.57 [0.00, 1.00]	0.58 [0.00, 1.00]	0.14 [0.00, 0.30]	0.74 [0.00, 0.96]
lr1e-05_wd0.005_h1024_d4	0.77 [0.67, 0.85]	0.31 [0.19, 0.39]	0.19 [0.00, 0.29]	0.55 [0.50, 0.62]	0.07 [0.00, 0.16]	0.52 [0.00, 1.00]	0.58 [0.00, 1.00]	0.12 [0.00, 0.20]	0.73 [0.00, 0.93]
lr1e-05_wd0.005_h2048_d4	0.74 [0.65, 0.81]	0.34 [0.18, 0.47]	0.20 [0.00, 0.33]	0.55 [0.50, 0.65]	0.08 [0.00, 0.22]	0.46 [0.00, 1.00]	0.65 [0.00, 1.00]	0.13 [0.00, 0.25]	0.55 [0.00, 0.93]
lr1e-05_wd0.01_h1024_d3	0.71 [0.61, 0.82]	0.36 [0.18, 0.45]	0.18 [0.00, 0.30]	0.54 [0.50, 0.65]	0.05 [0.00, 0.20]	0.39 [0.00, 1.00]	0.68 [0.00, 1.00]	0.11 [0.00, 0.19]	0.55 [0.00, 0.94]
lr1e-05_wd0.01_h2048_d3	0.76 [0.62, 0.85]	0.31 [0.15, 0.45]	0.16 [0.00, 0.36]	0.51 [0.41, 0.66]	0.02 [-0.14, 0.26]	0.53 [0.00, 1.00]	0.50 [0.00, 1.00]	0.11 [0.00, 0.29]	0.54 [0.00, 0.92]
lr1e-05_wd0.01_h1024_d4	0.76 [0.68, 0.84]	0.32 [0.21, 0.44]	0.14 [0.00, 0.25]	0.52 [0.50, 0.58]	0.02 [0.00, 0.11]	0.53 [0.00, 1.00]	0.50 [0.00, 1.00]	0.08 [0.00, 0.17]	0.54 [0.00, 0.90]
lr1e-05_wd0.01_h2048_d4	0.75 [0.65, 0.83]	0.41 [0.17, 0.53]	0.27 [0.17, 0.38]	0.61 [0.50, 0.72]	0.14 [0.00, 0.30]	0.56 [0.00, 0.79]	0.65 [0.33, 1.00]	0.18 [0.11, 0.25]	0.75 [0.00, 0.95]
lr3e-05_wd0.005_h1024_d3	0.76 [0.65, 0.87]	0.37 [0.19, 0.48]	0.19 [0.00, 0.40]	0.56 [0.39, 0.74]	0.08 [-0.16, 0.33]	0.66 [0.00, 1.00]	0.45 [0.00, 1.00]	0.13 [0.00, 0.27]	0.73 [0.00, 0.95]
lr3e-05_wd0.005_h2048_d3	0.77 [0.62, 0.90]	0.43 [0.18, 0.56]	0.25 [0.00, 0.67]	0.57 [0.45, 0.84]	0.13 [-0.06, 0.62]	0.67 [0.00, 1.00]	0.47 [0.00, 1.00]	0.20 [0.00, 0.60]	0.73 [0.00, 0.96]
lr3e-05_wd0.005_h1024_d4	0.77 [0.65, 0.86]	0.42 [0.22, 0.50]	0.26 [0.00, 0.43]	0.62 [0.50, 0.75]	0.17 [0.00, 0.36]	0.65 [0.00, 1.00]	0.58 [0.00, 1.00]	0.18 [0.00, 0.33]	0.75 [0.00, 0.96]
lr3e-05_wd0.005_h2048_d4	0.77 [0.67, 0.88]	0.35 [0.23, 0.55]	0.29 [0.00, 0.55]	0.63 [0.50, 0.81]	0.20 [0.00, 0.49]	0.63 [0.00, 1.00]	0.63 [0.00, 1.00]	0.21 [0.00, 0.43]	0.75 [0.00, 0.96]
lr3e-05_wd0.01_h1024_d3	0.76 [0.65, 0.84]	0.37 [0.18, 0.53]	0.29 [0.20, 0.43]	0.62 [0.50, 0.75]	0.17 [0.00, 0.36]	0.58 [0.00, 0.83]	0.65 [0.33, 1.00]	0.20 [0.12, 0.30]	0.75 [0.00, 0.96]
lr3e-05_wd0.01_h2048_d3	0.76 [0.63, 0.88]	0.37 [0.19, 0.54]	0.23 [0.00, 0.40]	0.56 [0.45, 0.68]	0.09 [-0.11, 0.31]	0.65 [0.00, 0.89]	0.47 [0.00, 1.00]	0.17 [0.00, 0.33]	0.73 [0.00, 0.93]
lr3e-05_wd0.01_h1024_d4	0.77 [0.69, 0.87]	0.40 [0.19, 0.49]	0.28 [0.18, 0.40]	0.62 [0.54, 0.72]	0.18 [0.06, 0.31]	0.75 [0.57, 0.86]	0.50 [0.25, 0.75]	0.21 [0.13, 0.33]	0.93 [0.89, 0.95]
lr3e-05_wd0.01_h2048_d4	0.77 [0.62, 0.88]	0.37 [0.15, 0.56]	0.21 [0.00, 0.43]	0.57 [0.41, 0.75]	0.10 [-0.14, 0.36]	0.69 [0.00, 1.00]	0.45 [0.00, 1.00]	0.15 [0.00, 0.33]	0.73 [0.00, 0.96]
lr1e-05_wd0.0_h256_d1	0.69 [0.56, 0.83]	0.35 [0.15, 0.42]	0.20 [0.00, 0.33]	0.55 [0.49, 0.66]	0.10 [-0.01, 0.29]	0.52 [0.00, 1.00]	0.58 [0.00, 1.00]	0.18 [0.00, 0.50]	0.74 [0.00, 1.00]
lr1e-05_wd0.0_h400_d1	0.71 [0.60, 0.83]	0.35 [0.15, 0.47]	0.18 [0.00, 0.30]	0.54 [0.48, 0.67]	0.07 [-0.03, 0.24]	0.35 [0.00, 1.00]	0.73 [0.00, 1.00]	0.10 [0.00, 0.17]	0.76 [0.00, 1.00]
lr1e-05_wd0.0_h512_d1	0.69 [0.60, 0.84]	0.41 [0.24, 0.70]	0.15 [0.00, 0.28]	0.54 [0.50, 0.64]	0.07 [0.00, 0.21]	0.48 [0.00, 1.00]	0.60 [0.00, 1.00]	0.08 [0.00, 0.16]	0.76 [0.00, 1.00]
lr1e-05_wd0.0_h256_d2	0.72 [0.58, 0.83]	0.34 [0.19, 0.62]	0.23 [0.00, 0.46]	0.59 [0.50, 0.88]	0.12 [0.00, 0.47]	0.48 [0.00, 1.00]	0.70 [0.00, 1.00]	0.14 [0.00, 0.30]	0.56 [0.00, 1.00]
lr1e-05_wd0.0_h400_d2	0.83 [0.71, 0.95]	0.51 [0.34, 0.70]	0.21 [0.00, 0.40]	0.53 [0.50, 0.65]	0.07 [0.00, 0.36]	0.39 [0.00, 1.00]	0.67 [0.00, 1.00]	0.17 [0.00, 0.50]	0.37 [0.00, 0.93]
lr1e-05_wd0.0_h512_d2	0.68 [0.58, 0.82]	0.31 [0.16, 0.43]	0.20 [0.00, 0.35]	0.58 [0.50, 0.74]	0.11 [0.00, 0.32]	0.35 [0.00, 1.00]	0.80 [0.00, 1.00]	0.12 [0.00, 0.21]	0.58 [0.00, 1.00]
lr1e-05_wd0.0001_h256_d1	0.70 [0.57, 0.80]	0.36 [0.25, 0.45]	0.16 [0.00, 0.33]	0.57 [0.50, 0.72]	0.10 [0.00, 0.30]	0.54 [0.00, 1.00]	0.60 [0.00, 1.00]	0.10 [0.00, 0.20]	0.76 [0.00, 1.00]
lr1e-05_wd0.0001_h400_d1	0.74 [0.65, 0.88]	0.43 [0.27, 0.70]	0.19 [0.00, 0.28]	0.56 [0.50, 0.64]	0.08 [0.00, 0.21]	0.31 [0.00, 1.00]	0.80 [0.00, 1.00]	0.11 [0.00, 0.16]	0.58 [0.00, 1.00]
lr1e-05_wd0.0001_h512_d1	0.70 [0.58, 0.82]	0.34 [0.17, 0.44]	0.24 [0.18, 0.26]	0.59 [0.52, 0.68]	0.14 [0.06, 0.23]	0.22 [0.04, 0.45]	0.95 [0.75, 1.00]	0.14 [0.10, 0.16]	0.99 [0.93, 1.00]
lr1e-05_wd0.0001_h256_d2	0.76 [0.61, 0.86]	0.32 [0.20, 0.44]	0.13 [0.00, 0.23]	0.50 [0.46, 0.53]	0.00 [-0.09, 0.09]	0.40 [0.00, 1.00]	0.60 [0.00, 1.00]	0.07 [0.00, 0.13]	0.56 [0.00, 1.00]
lr1e-05_wd0.0001_h400_d2	0.73 [0.65, 0.82]	0.33 [0.18, 0.45]	0.13 [0.00, 0.23]	0.51 [0.50, 0.53]	0.02 [0.00, 0.09]	0.41 [0.00, 1.00]	0.60 [0.00, 1.00]	0.07 [0.00, 0.13]	0.56 [0.00, 1.00]
lr1e-05_wd0.0001_h512_d2	0.73 [0.61, 0.84]	0.35 [0.17, 0.50]	0.14 [0.00, 0.27]	0.52 [0.48, 0.62]	0.03 [-0.06, 0.19]	0.44 [0.00, 1.00]	0.60 [0.00, 1.00]	0.08 [0.00, 0.15]	0.56 [0.00, 1.00]
lr1e-05_wd0.001_h256_d1	0.68 [0.60, 0.82]	0.35 [0.16, 0.48]	0.22 [0.18, 0.25]	0.56 [0.50, 0.63]	0.10 [0.00, 0.18]	0.11 [0.00, 0.25]	1.00 [1.00, 1.00]	0.12 [0.10, 0.14]	0.80 [0.00, 1.00]
lr1e-05_wd0.001_h400_d1	0.71 [0.55, 0.89]	0.38 [0.21, 0.60]	0.19 [0.00, 0.31]	0.53 [0.46, 0.63]	0.03 [-0.09, 0.19]	0.42 [0.00, 0.93]	0.63 [0.00, 1.00]	0.12 [0.00, 0.22]	0.55 [0.00, 0.92]
lr1e-05_wd0.001_h512_d1	0.74 [0.61, 0.84]	0.37 [0.21, 0.46]	0.20 [0.00, 0.35]	0.58 [0.50, 0.74]	0.10 [0.00, 0.32]	0.35 [0.00, 1.00]	0.80 [0.00, 1.00]	0.12 [0.00, 0.21]	0.58 [0.00, 1.00]
lr1e-05_wd0.001_h256_d2	0.72 [0.58, 0.82]	0.36 [0.16, 0.65]	0.13 [0.00, 0.23]	0.51 [0.50, 0.53]	0.02 [0.00, 0.09]	0.41 [0.00, 1.00]	0.60 [0.00, 1.00]	0.07 [0.00, 0.13]	0.56 [0.00, 1.00]
lr1e-05_wd0.001_h400_d2	0.74 [0.64, 0.91]	0.42 [0.26, 0.63]	0.09 [0.00, 0.22]	0.50 [0.50, 0.50]	0.00 [0.00, 0.00]	0.60 [0.00, 1.00]	0.40 [0.00, 1.00]	0.05 [0.00, 0.12]	0.54 [0.00, 0.90]
lr1e-05_wd0.001_h512_d2	0.70 [0.61, 0.83]	0.33 [0.17, 0.66]	0.17 [0.00, 0.25]	0.51 [0.44, 0.59]	0.01 [-0.08, 0.16]	0.28 [0.00, 1.00]	0.73 [0.00, 1.00]	0.09 [0.00, 0.14]	0.55 [0.00, 1.00]
lr3e-05_wd0.0_h256_d1	0.72 [0.62, 0.83]	0.31 [0.17, 0.41]	0.20 [0.17, 0.23]	0.52 [0.50, 0.53]	0.04 [0.00, 0.09]	0.17 [0.00, 0.39]	0.87 [0.67, 1.00]	0.12 [0.10, 0.13]	0.77 [0.00, 1.00]
lr3e-05_wd0.0_h400_d1	0.72 [0.62, 0.84]	0.31 [0.16, 0.42]	0.21 [0.00, 0.38]	0.57 [0.50, 0.72]	0.09 [0.00, 0.30]	0.55 [0.00, 1.00]	0.58 [0.00, 1.00]	0.13 [0.00, 0.25]	0.74 [0.00, 0.95]
lr3e-05_wd0.0_h512_d1	0.72 [0.64, 0.84]	0.38 [0.19, 0.51]	0.22 [0.00, 0.35]	0.58 [0.50, 0.70]	0.12 [0.00, 0.27]	0.61 [0.04, 1.00]	0.55 [0.00, 1.00]	0.14 [0.00, 0.23]	0.93 [0.90, 1.00]
lr3e-05_wd0.0_h256_d2	0.79 [0.74, 0.88]	0.34 [0.26, 0.44]	0.14 [0.00, 0.27]	0.52 [0.50, 0.59]	0.03 [0.00, 0.13]	0.54 [0.00, 1.00]	0.50 [0.00, 1.00]	0.08 [0.00, 0.18]	0.54 [0.00, 0.91]
lr3e-05_wd0.0_h400_d2	0.74 [0.67, 0.84]	0.39 [0.17, 0.49]	0.26 [0.14, 0.38]	0.59 [0.49, 0.72]	0.12 [-0.01, 0.30]	0.53 [0.00, 0.76]	0.65 [0.33, 1.00]	0.17 [0.09, 0.25]	0.74 [0.00, 0.95]
lr3e-05_wd0.0_h512_d2	0.77 [0.65, 0.85]	0.43 [0.20, 0.53]	0.19 [0.00, 0.29]	0.55 [0.50, 0.62]	0.06 [0.00, 0.16]	0.46 [0.00, 1.00]	0.63 [0.00, 1.00]	0.12 [0.00, 0.20]	0.55 [0.00, 0.94]
lr3e-05_wd0.0001_h256_d1	0.75 [0.65, 0.87]	0.44 [0.31, 0.67]	0.14 [0.00, 0.29]	0.53 [0.50, 0.63]	0.03 [0.00, 0.17]	0.50 [0.00, 1.00]	0.55 [0.00, 1.00]	0.08 [0.00, 0.18]	0.55 [0.00, 0.94]
lr3e-05_wd0.0001_h400_d1	0.76 [0.62, 0.84]	0.34 [0.20, 0.42]	0.19 [0.00, 0.40]	0.60 [0.50, 0.74]	0.13 [0.00, 0.33]	0.64 [0.00, 1.00]	0.55 [0.00, 1.00]	0.12 [0.00, 0.27]	0.75 [0.00, 1.00]
lr3e-05_wd0.0001_h512_d1	0.72 [0.60, 0.88]	0.38 [0.15, 0.55]	0.18 [0.00, 0.33]	0.53 [0.45, 0.69]	0.04 [-0.06, 0.24]	0.44 [0.00, 1.00]	0.62 [0.00, 1.00]	0.11 [0.00, 0.21]	0.55 [0.00, 0.95]
lr3e-05_wd0.0001_h256_d2	0.75 [0.64, 0.84]	0.38 [0.18, 0.64]	0.24 [0.22, 0.27]	0.57 [0.50, 0.65]	0.09 [0.00, 0.19]	0.37 [0.00, 0.69]	0.77 [0.50, 1.00]	0.14 [0.12, 0.18]	0.56 [0.00, 0.95]
lr3e-05_wd0.0001_h400_d2	0.74 [0.62, 0.85]	0.37 [0.15, 0.48]	0.23 [0.12, 0.38]	0.56 [0.43, 0.72]	0.08 [-0.08, 0.30]	0.47 [0.00, 0.69]	0.65 [0.33, 1.00]	0.14 [0.07, 0.25]	0.73 [0.00, 0.95]
lr3e-05_wd0.0001_h512_d2	0.72 [0.60, 0.84]	0.36 [0.14, 0.44]	0.25 [0.13, 0.35]	0.58 [0.47, 0.70]	0.11 [-0.04, 0.27]	0.57 [0.00, 0.82]	0.58 [0.33, 1.00]	0.16 [0.08, 0.23]	0.74 [0.00, 0.95]
lr3e-05_wd0.001_h256_d1	0.73 [0.65, 0.83]	0.31 [0.18, 0.41]	0.20 [0.00, 0.32]	0.58 [0.50, 0.71]	0.12 [0.00, 0.28]	0.48 [0.07, 1.00]	0.68 [0.00, 1.00]	0.12 [0.00, 0.19]	0.95 [0.90, 1.00]
lr3e-05_wd0.001_h400_d1	0.72 [0.63, 0.83]	0.29 [0.16, 0.37]	0.22 [0.00, 0.38]	0.57 [0.50, 0.72]	0.11 [0.00, 0.30]	0.50 [0.00, 1.00]	0.65 [0.00, 1.00]	0.14 [0.00, 0.25]	0.75 [0.00, 1.00]
lr3e-05_wd0.001_h512_d1	0.74 [0.62, 0.84]	0.34 [0.25, 0.42]	0.25 [0.19, 0.33]	0.59 [0.50, 0.72]	0.13 [0.00, 0.30]	0.36 [0.00, 0.66]	0.83 [0.50, 1.00]	0.15 [0.11, 0.20]	0.77 [0.00, 1.00]
lr3e-05_wd0.001_h256_d2	0.75 [0.68, 0.84]	0.36 [0.21, 0.54]	0.22 [0.15, 0.27]	0.54 [0.44, 0.71]	0.05 [-0.08, 0.26]	0.25 [0.00, 0.59]	0.83 [0.50, 1.00]	0.13 [0.08, 0.16]	0.55 [0.00, 1.00]
lr3e-05_wd0.001_h400_d2	0.82 [0.71, 0.96]	0.44 [0.20, 0.76]	0.23 [0.00, 0.40]	0.59 [0.50, 0.74]	0.12 [0.00, 0.33]	0.60 [0.00, 1.00]	0.58 [0.00, 1.00]	0.15 [0.00, 0.27]	0.74 [0.00, 0.95]
lr3e-05_wd0.001_h512_d2	0.76 [0.64, 0.86]	0.42 [0.30, 0.62]	0.21 [0.00, 0.38]	0.57 [0.50, 0.72]	0.10 [0.00, 0.30]	0.63 [0.00, 1.00]	0.52 [0.00, 1.00]	0.14 [0.00, 0.25]	0.74 [0.00, 0.95]
lr0.0001_wd0.005_h256_d1	0.76 [0.63, 0.87]	0.41 [0.19, 0.52]	0.33 [0.15, 0.55]	0.64 [0.51, 0.81]	0.23 [0.01, 0.49]	0.80 [0.68, 0.86]	0.48 [0.33, 0.75]	0.25 [0.10, 0.43]	0.93 [0.90, 0.96]
lr0.0001_wd0.005_h400_d1	0.76 [0.63, 0.87]	0.41 [0.16, 0.54]	0.31 [0.20, 0.46]	0.66 [0.56, 0.77]	0.23 [0.08, 0.40]	0.68 [0.41, 0.79]	0.65 [0.33, 1.00]	0.21 [0.14, 0.33]	0.95 [0.91, 1.00]
lr0.0001_wd0.005_h512_d1	0.77 [0.64, 0.87]	0.44 [0.35, 0.52]	0.27 [0.00, 0.55]	0.63 [0.50, 0.81]	0.22 [0.00, 0.49]	0.92 [0.86, 1.00]	0.35 [0.00, 0.75]	0.22 [0.00, 0.43]	0.92 [0.90, 0.96]
lr0.0001_wd0.005_h256_d2	0.78 [0.67, 0.86]	0.38 [0.21, 0.51]	0.31 [0.20, 0.55]	0.62 [0.50, 0.81]	0.19 [0.00, 0.49]	0.66 [0.00, 0.86]	0.58 [0.33, 1.00]	0.23 [0.12, 0.43]	0.74 [0.00, 0.96]
lr0.0001_wd0.005_h400_d2	0.78 [0.73, 0.87]	0.43 [0.29, 0.53]	0.30 [0.00, 0.50]	0.64 [0.50, 0.72]	0.24 [0.00, 0.43]	0.86 [0.62, 1.00]	0.42 [0.00, 0.75]	0.26 [0.00, 0.50]	0.93 [0.90, 0.95]
lr0.0001_wd0.005_h512_d2	0.76 [0.65, 0.86]	0.38 [0.22, 0.47]	0.28 [0.20, 0.44]	0.59 [0.52, 0.70]	0.18 [0.07, 0.36]	0.64 [0.03, 0.97]	0.55 [0.25, 1.00]	0.26 [0.13, 0.50]	0.94 [0.90, 1.00]
lr0.0001_wd0.01_h256_d1	0.77 [0.63, 0.87]	0.38 [0.19, 0.50]	0.25 [0.00, 0.43]	0.58 [0.41, 0.75]	0.13 [-0.14, 0.36]	0.65 [0.00, 0.86]	0.52 [0.00, 1.00]	0.18 [0.00, 0.33]	0.74 [0.00, 0.96]
lr0.0001_wd0.01_h400_d1	0.76 [0.64, 0.87]	0.42 [0.17, 0.52]	0.32 [0.13, 0.55]	0.65 [0.47, 0.81]	0.23 [-0.04, 0.49]	0.71 [0.41, 0.86]	0.58 [0.33, 1.00]	0.24 [0.08, 0.43]	0.94 [0.89, 1.00]

Supplementary Table 7: Grid search results on the classification task on GDHCM retrospective dataset (choosing 50% as the threshold for clinical variables selection). Reported metrics are AUC, AUPRC, F1, Balanced Accuracy, MCC, Specificity, Sensitivity, PPV, and NPV.

Config	AUC	AUPRC	F1	Balanced_Accuracy	MCC	Specificity	Sensitivity	PPV	NPV
lr0.0001_wd0.01_h512_d1	0.75 [0.62, 0.87]	0.42 [0.27, 0.50]	0.29 [0.00, 0.44]	0.65 [0.50, 0.74]	0.23 [0.00, 0.36]	0.75 [0.29, 1.00]	0.55 [0.00, 1.00]	0.22 [0.00, 0.40]	0.94 [0.90, 1.00]
lr0.0001_wd0.01_h256_d2	0.76 [0.68, 0.85]	0.38 [0.18, 0.48]	0.31 [0.00, 0.55]	0.63 [0.43, 0.81]	0.22 [-0.13, 0.49]	0.85 [0.83, 0.86]	0.42 [0.00, 0.75]	0.25 [0.00, 0.43]	0.92 [0.89, 0.96]
lr0.0001_wd0.01_h400_d2	0.78 [0.69, 0.88]	0.42 [0.20, 0.51]	0.30 [0.00, 0.46]	0.63 [0.45, 0.77]	0.20 [-0.11, 0.40]	0.85 [0.79, 0.89]	0.42 [0.00, 0.75]	0.23 [0.00, 0.33]	0.92 [0.89, 0.96]
lr0.0001_wd0.01_h512_d2	0.78 [0.68, 0.86]	0.38 [0.19, 0.50]	0.28 [0.18, 0.55]	0.60 [0.50, 0.81]	0.16 [0.00, 0.49]	0.61 [0.00, 0.90]	0.60 [0.25, 1.00]	0.21 [0.12, 0.43]	0.74 [0.00, 0.96]
lr0.0003_wd0.005_h256_d1	0.77 [0.64, 0.89]	0.41 [0.17, 0.52]	0.30 [0.22, 0.44]	0.62 [0.52, 0.70]	0.19 [0.07, 0.36]	0.64 [0.03, 0.90]	0.60 [0.33, 1.00]	0.23 [0.13, 0.40]	0.94 [0.92, 1.00]
lr0.0003_wd0.005_h400_d1	0.78 [0.67, 0.89]	0.46 [0.18, 0.59]	0.32 [0.15, 0.67]	0.62 [0.51, 0.84]	0.21 [0.01, 0.62]	0.74 [0.39, 0.93]	0.50 [0.25, 0.75]	0.26 [0.10, 0.60]	0.92 [0.89, 0.96]
lr0.0003_wd0.005_h512_d1	0.79 [0.67, 0.88]	0.46 [0.30, 0.53]	0.34 [0.20, 0.55]	0.66 [0.54, 0.81]	0.26 [0.07, 0.49]	0.70 [0.25, 0.90]	0.63 [0.25, 1.00]	0.26 [0.13, 0.43]	0.95 [0.89, 1.00]
lr0.0003_wd0.005_h256_d2	0.77 [0.65, 0.90]	0.43 [0.17, 0.54]	0.29 [0.00, 0.60]	0.61 [0.41, 0.82]	0.19 [-0.14, 0.55]	0.85 [0.76, 0.93]	0.37 [0.00, 0.75]	0.25 [0.00, 0.50]	0.92 [0.88, 0.96]
lr0.0003_wd0.005_h400_d2	0.81 [0.76, 0.89]	0.45 [0.23, 0.53]	0.33 [0.00, 0.67]	0.65 [0.45, 0.84]	0.25 [-0.11, 0.62]	0.74 [0.24, 0.93]	0.57 [0.00, 1.00]	0.26 [0.00, 0.60]	0.95 [0.89, 1.00]
lr0.0003_wd0.005_h512_d2	0.77 [0.68, 0.88]	0.37 [0.19, 0.51]	0.32 [0.00, 0.50]	0.63 [0.41, 0.76]	0.23 [-0.14, 0.43]	0.87 [0.82, 0.93]	0.38 [0.00, 0.67]	0.29 [0.00, 0.50]	0.92 [0.88, 0.96]
lr0.0003_wd0.01_h256_d1	0.77 [0.64, 0.91]	0.44 [0.20, 0.55]	0.33 [0.22, 0.43]	0.65 [0.58, 0.75]	0.23 [0.12, 0.36]	0.82 [0.76, 0.86]	0.48 [0.33, 0.75]	0.25 [0.17, 0.33]	0.93 [0.92, 0.96]
lr0.0003_wd0.01_h400_d1	0.78 [0.67, 0.89]	0.45 [0.24, 0.55]	0.35 [0.20, 0.57]	0.64 [0.57, 0.73]	0.26 [0.12, 0.53]	0.72 [0.14, 0.97]	0.57 [0.33, 1.00]	0.31 [0.11, 0.67]	0.94 [0.92, 1.00]
lr0.0003_wd0.01_h512_d1	0.76 [0.63, 0.89]	0.42 [0.16, 0.54]	0.35 [0.00, 0.67]	0.62 [0.36, 0.84]	0.24 [-0.19, 0.62]	0.77 [0.39, 0.93]	0.48 [0.00, 0.75]	0.31 [0.00, 0.60]	0.92 [0.87, 0.96]
lr0.0003_wd0.01_h256_d2	0.79 [0.69, 0.87]	0.44 [0.21, 0.58]	0.27 [0.00, 0.50]	0.58 [0.39, 0.72]	0.15 [-0.16, 0.43]	0.80 [0.62, 0.93]	0.37 [0.00, 0.50]	0.23 [0.00, 0.50]	0.91 [0.88, 0.93]
lr0.0003_wd0.01_h400_d2	0.79 [0.68, 0.89]	0.44 [0.22, 0.55]	0.23 [0.00, 0.36]	0.56 [0.41, 0.66]	0.13 [-0.14, 0.29]	0.86 [0.82, 0.97]	0.27 [0.00, 0.50]	0.23 [0.00, 0.50]	0.90 [0.88, 0.92]
lr0.0003_wd0.01_h512_d2	0.79 [0.69, 0.90]	0.44 [0.20, 0.53]	0.35 [0.00, 0.67]	0.64 [0.39, 0.84]	0.26 [-0.16, 0.62]	0.87 [0.79, 0.93]	0.42 [0.00, 0.75]	0.31 [0.00, 0.60]	0.92 [0.88, 0.96]
lr0.001_wd0.005_h256_d1	0.80 [0.71, 0.90]	0.42 [0.23, 0.53]	0.34 [0.25, 0.50]	0.67 [0.60, 0.79]	0.26 [0.15, 0.44]	0.69 [0.28, 0.86]	0.65 [0.33, 1.00]	0.24 [0.16, 0.38]	0.95 [0.92, 1.00]
lr0.001_wd0.005_h400_d1	0.76 [0.63, 0.88]	0.44 [0.22, 0.52]	0.33 [0.15, 0.60]	0.63 [0.44, 0.82]	0.21 [-0.08, 0.55]	0.69 [0.21, 0.90]	0.57 [0.25, 0.75]	0.26 [0.08, 0.50]	0.92 [0.86, 0.96]
lr0.001_wd0.005_h512_d1	0.78 [0.67, 0.90]	0.44 [0.19, 0.56]	0.31 [0.00, 0.57]	0.61 [0.39, 0.73]	0.21 [-0.16, 0.53]	0.85 [0.79, 0.97]	0.37 [0.00, 0.50]	0.29 [0.00, 0.67]	0.92 [0.88, 0.93]
lr0.001_wd0.005_h256_d2	0.83 [0.70, 0.90]	0.45 [0.28, 0.65]	0.42 [0.36, 0.60]	0.75 [0.66, 0.84]	0.36 [0.26, 0.55]	0.78 [0.68, 0.90]	0.73 [0.50, 1.00]	0.31 [0.25, 0.50]	0.96 [0.92, 1.00]
lr0.001_wd0.005_h400_d2	0.79 [0.63, 0.90]	0.49 [0.17, 0.72]	0.42 [0.20, 0.60]	0.72 [0.56, 0.82]	0.35 [0.08, 0.55]	0.83 [0.75, 0.93]	0.60 [0.33, 0.75]	0.34 [0.14, 0.50]	0.94 [0.92, 0.96]
lr0.001_wd0.005_h512_d2	0.79 [0.65, 0.88]	0.39 [0.27, 0.48]	0.38 [0.18, 0.60]	0.68 [0.50, 0.90]	0.30 [0.00, 0.56]	0.70 [0.00, 0.97]	0.67 [0.25, 1.00]	0.33 [0.10, 0.50]	0.76 [0.00, 1.00]
lr0.001_wd0.01_h256_d1	0.76 [0.65, 0.90]	0.37 [0.22, 0.53]	0.31 [0.18, 0.40]	0.63 [0.54, 0.68]	0.23 [0.06, 0.31]	0.74 [0.25, 0.97]	0.52 [0.25, 1.00]	0.28 [0.13, 0.50]	0.93 [0.90, 1.00]
lr0.001_wd0.01_h400_d1	0.77 [0.69, 0.86]	0.48 [0.18, 0.70]	0.28 [0.20, 0.40]	0.61 [0.57, 0.68]	0.19 [0.10, 0.31]	0.65 [0.14, 0.90]	0.58 [0.25, 1.00]	0.22 [0.11, 0.33]	0.94 [0.90, 1.00]
lr0.001_wd0.01_h512_d1	0.80 [0.65, 0.90]	0.41 [0.29, 0.62]	0.29 [0.00, 0.40]	0.65 [0.47, 0.75]	0.22 [-0.09, 0.33]	0.79 [0.50, 0.93]	0.52 [0.00, 1.00]	0.22 [0.00, 0.33]	0.94 [0.87, 1.00]
lr0.001_wd0.01_h256_d2	0.83 [0.77, 0.96]	0.47 [0.25, 0.65]	0.34 [0.22, 0.40]	0.68 [0.58, 0.74]	0.26 [0.12, 0.32]	0.76 [0.48, 0.86]	0.60 [0.33, 1.00]	0.25 [0.17, 0.33]	0.95 [0.92, 1.00]
lr0.001_wd0.01_h400_d2	0.80 [0.70, 0.91]	0.50 [0.30, 0.76]	0.36 [0.24, 0.46]	0.69 [0.55, 0.77]	0.28 [0.12, 0.40]	0.66 [0.10, 0.90]	0.72 [0.50, 1.00]	0.26 [0.13, 0.40]	0.96 [0.93, 1.00]
lr0.001_wd0.01_h512_d2	0.79 [0.69, 0.93]	0.44 [0.18, 0.74]	0.34 [0.22, 0.60]	0.67 [0.50, 0.82]	0.26 [0.00, 0.55]	0.61 [0.00, 0.90]	0.73 [0.25, 1.00]	0.26 [0.12, 0.50]	0.76 [0.00, 1.00]
lr0.0001_wd0.0_h1024_d1	0.76 [0.63, 0.87]	0.43 [0.17, 0.62]	0.31 [0.15, 0.55]	0.61 [0.50, 0.81]	0.18 [0.00, 0.49]	0.64 [0.00, 0.86]	0.58 [0.33, 1.00]	0.23 [0.10, 0.43]	0.74 [0.00, 0.96]
lr0.0001_wd0.0_h2048_d1	0.77 [0.65, 0.89]	0.45 [0.25, 0.55]	0.32 [0.20, 0.60]	0.64 [0.50, 0.82]	0.22 [0.00, 0.55]	0.50 [0.00, 0.90]	0.78 [0.50, 1.00]	0.23 [0.12, 0.50]	0.76 [0.00, 1.00]
lr0.0001_wd0.0_h1024_d2	0.78 [0.69, 0.90]	0.45 [0.21, 0.56]	0.33 [0.16, 0.57]	0.62 [0.48, 0.73]	0.22 [-0.03, 0.53]	0.72 [0.29, 0.97]	0.52 [0.25, 0.67]	0.30 [0.09, 0.67]	0.92 [0.89, 0.94]
lr0.0001_wd0.0_h2048_d2	0.79 [0.69, 0.91]	0.51 [0.18, 0.70]	0.37 [0.16, 0.67]	0.63 [0.48, 0.84]	0.29 [-0.03, 0.62]	0.77 [0.29, 1.00]	0.50 [0.25, 0.75]	0.44 [0.09, 1.00]	0.92 [0.89, 0.96]
lr0.0001_wd0.0001_h1024_d1	0.75 [0.62, 0.86]	0.39 [0.15, 0.52]	0.27 [0.00, 0.55]	0.59 [0.43, 0.81]	0.14 [-0.13, 0.49]	0.60 [0.00, 0.86]	0.58 [0.00, 1.00]	0.19 [0.00, 0.43]	0.74 [0.00, 0.96]
lr0.0001_wd0.0001_h2048_d1	0.78 [0.61, 0.89]	0.46 [0.32, 0.59]	0.34 [0.23, 0.55]	0.66 [0.59, 0.81]	0.25 [0.13, 0.49]	0.71 [0.29, 0.86]	0.62 [0.33, 1.00]	0.25 [0.13, 0.43]	0.94 [0.91, 1.00]
lr0.0001_wd0.0001_h1024_d2	0.79 [0.67, 0.90]	0.47 [0.19, 0.59]	0.25 [0.00, 0.44]	0.58 [0.45, 0.70]	0.14 [-0.11, 0.36]	0.77 [0.32, 0.93]	0.38 [0.00, 0.67]	0.22 [0.00, 0.40]	0.91 [0.89, 0.93]
lr0.0001_wd0.0001_h2048_d2	0.75 [0.63, 0.87]	0.44 [0.16, 0.61]	0.33 [0.00, 0.60]	0.62 [0.36, 0.82]	0.22 [-0.19, 0.55]	0.83 [0.71, 0.90]	0.42 [0.00, 0.75]	0.27 [0.00, 0.50]	0.92 [0.87, 0.96]
lr0.0001_wd0.001_h1024_d1	0.74 [0.61, 0.87]	0.41 [0.15, 0.52]	0.28 [0.00, 0.55]	0.61 [0.41, 0.81]	0.18 [-0.14, 0.49]	0.81 [0.76, 0.86]	0.42 [0.00, 0.75]	0.22 [0.00, 0.43]	0.92 [0.88, 0.96]
lr0.0001_wd0.001_h2048_d1	0.80 [0.68, 0.90]	0.47 [0.23, 0.58]	0.37 [0.24, 0.67]	0.69 [0.56, 0.84]	0.29 [0.09, 0.62]	0.71 [0.38, 0.93]	0.67 [0.33, 1.00]	0.29 [0.14, 0.60]	0.95 [0.92, 1.00]
lr0.0001_wd0.001_h1024_d2	0.79 [0.67, 0.88]	0.44 [0.23, 0.52]	0.42 [0.22, 0.60]	0.70 [0.58, 0.82]	0.34 [0.12, 0.55]	0.86 [0.79, 0.93]	0.55 [0.33, 0.75]	0.35 [0.17, 0.50]	0.94 [0.92, 0.96]
lr0.0001_wd0.001_h2048_d2	0.76 [0.62, 0.91]	0.40 [0.18, 0.57]	0.38 [0.15, 0.75]	0.64 [0.48, 0.86]	0.27 [-0.03, 0.72]	0.74 [0.29, 0.97]	0.55 [0.33, 0.75]	0.33 [0.09, 0.75]	0.92 [0.89, 0.97]
lr0.0003_wd0.0_h1024_d1	0.78 [0.63, 0.89]	0.36 [0.19, 0.48]	0.35 [0.00, 0.67]	0.67 [0.43, 0.84]	0.27 [-0.13, 0.62]	0.80 [0.64, 0.93]	0.53 [0.00, 0.75]	0.28 [0.00, 0.60]	0.94 [0.89, 0.96]
lr0.0003_wd0.0_h2048_d1	0.77 [0.64, 0.91]	0.47 [0.21, 0.59]	0.34 [0.21, 0.50]	0.65 [0.58, 0.75]	0.27 [0.10, 0.43]	0.80 [0.50, 0.97]	0.50 [0.25, 0.75]	0.33 [0.13, 0.50]	0.93 [0.90, 0.96]
lr0.0003_wd0.0_h1024_d2	0.78 [0.68, 0.90]	0.37 [0.22, 0.49]	0.31 [0.22, 0.43]	0.65 [0.56, 0.75]	0.24 [0.10, 0.36]	0.63 [0.17, 0.96]	0.67 [0.25, 1.00]	0.26 [0.14, 0.50]	0.96 [0.89, 1.00]
lr0.0003_wd0.0_h2048_d2	0.80 [0.63, 0.91]	0.46 [0.29, 0.55]	0.41 [0.20, 0.57]	0.74 [0.56, 0.88]	0.37 [0.08, 0.53]	0.77 [0.57, 0.97]	0.72 [0.33, 1.00]	0.33 [0.14, 0.67]	0.96 [0.92, 1.00]
lr0.0003_wd0.0001_h1024_d1	0.78 [0.68, 0.90]	0.42 [0.18, 0.55]	0.28 [0.00, 0.50]	0.61 [0.39, 0.72]	0.18 [-0.16, 0.43]	0.76 [0.45, 0.93]	0.47 [0.00, 1.00]	0.22 [0.00, 0.50]	0.93 [0.88, 1.00]
lr0.0003_wd0.0001_h2048_d1	0.78 [0.67, 0.90]	0.45 [0.18, 0.56]	0.38 [0.22, 0.55]	0.70 [0.58, 0.81]	0.31 [0.12, 0.49]	0.78 [0.46, 0.90]	0.62 [0.33, 1.00]	0.30 [0.17, 0.43]	0.95 [0.92, 1.00]
lr0.0003_wd0.0001_h1024_d2	0.81 [0.69, 0.89]	0.46 [0.25, 0.56]	0.42 [0.31, 0.50]	0.73 [0.66, 0.88]	0.35 [0.24, 0.47]	0.82 [0.71, 0.93]	0.63 [0.50, 1.00]	0.34 [0.20, 0.50]	0.95 [0.92, 1.00]
lr0.0003_wd0.0001_h2048_d2	0.81 [0.70, 0.90]	0.55 [0.26, 0.75]	0.40 [0.17, 0.60]	0.72 [0.51, 0.82]	0.33 [0.01, 0.55]	0.71 [0.36, 0.90]	0.73 [0.50, 1.00]	0.31 [0.10, 0.50]	0.95 [0.91, 1.00]
lr0.0003_wd0.001_h1024_d1	0.79 [0.64, 0.90]	0.46 [0.30, 0.56]	0.36 [0.20, 0.57]	0.68 [0.56, 0.73]	0.30 [0.08, 0.53]	0.70 [0.38, 0.97]	0.67 [0.33, 1.00]	0.31 [0.14, 0.67]	0.96 [0.92, 1.00]
lr0.0003_wd0.001_h2048_d1	0.78 [0.65, 0.91]	0.42 [0.18, 0.54]	0.29 [0.20, 0.40]	0.63 [0.56, 0.73]	0.22 [0.08, 0.31]	0.79 [0.46, 0.93]	0.47 [0.25, 1.00]	0.26 [0.14, 0.33]	0.93 [0.90, 1.00]
lr0.0003_wd0.001_h1024_d2	0.80 [0.71, 0.91]	0.45 [0.25, 0.56]	0.41 [0.22, 0.57]	0.67 [0.58, 0.73]	0.34 [0.12, 0.53]	0.90 [0.82, 0.97]	0.43 [0.33, 0.50]	0.42 [0.17, 0.67]	0.93 [0.92, 0.93]
lr0.0003_wd0.001_h2048_d2	0.80 [0.69, 0.91]	0.45 [0.24, 0.56]	0.39 [0.23, 0.67]	0.67 [0.57, 0.84]	0.34 [0.15, 0.62]	0.73 [0.29, 1.00]	0.62 [0.25, 1.00]	0.43 [0.13, 1.00]	0.95 [0.90, 1.00]
lr0.001_wd0.0_h1024_d1	0.77 [0.60, 0.92]	0.39 [0.19, 0.58]	0.27 [0.18, 0.44]	0.61 [0.54, 0.71]	0.18 [0.06, 0.36]	0.59 [0.14, 0.90]	0.63 [0.33, 1.00]	0.20 [0.13, 0.40]	0.95 [0.91, 1.00]
lr0.001_wd0.0_h2048_d1	0.82 [0.65, 0.91]	0.45 [0.20, 0.61]	0.35 [0.18, 0.57]	0.64 [0.54, 0.81]	0.26 [0.06, 0.53]	0.70 [0.10, 0.97]	0.58 [0.33, 1.00]	0.30 [0.13, 0.67]	0.95 [0.91, 1.00]
lr0.001_wd0.0_h1024_d2	0.78 [0.67, 0.85]	0.46 [0.32, 0.56]	0.36 [0.22, 0.55]	0.67 [0.50, 0.81]	0.26 [0.00, 0.49]	0.62 [0.00, 0.86]	0.72 [0.50, 1.00]	0.26 [0.12, 0.43]	0.76 [0.00, 0.96]
lr0.001_wd0.0_h2048_d2	0.81 [0.73, 0.89]	0.48 [0.27, 0.60]	0.39 [0.34, 0.45]	0.75 [0.72, 0.81]	0.33 [0.29, 0.39]	0.89 [0.86, 0.93]	0.62 [0.50, 0.75]	0.42 [0.33, 0.50]	0.95 [0.93, 0.96]
lr0.001_wd0.0001_h1024_d1	0.82 [0.75, 0.91]	0.46 [0.22, 0.57]	0.33 [0.00, 0.67]	0.64 [0.41, 0.84]	0.24 [-0.14, 0.62]	0.84 [0.61, 0.93]	0.43 [0.00, 0.75]	0.30 [0.00, 0.60]	0.92 [0.88, 0.96]
lr0.001_wd0.0001_h2048_d1	0.78 [0.63, 0.91]	0.45 [0.16, 0.59]	0.44 [0.27, 0.57]	0.72 [0.65, 0.79]	0.37 [0.19, 0.53]	0.83 [0.64, 0.97]	0.62 [0.50, 0.75]	0.38 [0.17, 0.67]	0.95 [0.93, 0.96]
lr0.001_wd0.0001_h1024_d2	0.80 [0.67, 0.90]	0.42 [0.25, 0.70]	0.24 [0.00, 0.50]	0.62 [0.48, 0.79]	0.16 [-0.07, 0.44]	0.77 [0.46, 0.97]	0.47 [0.00, 1.00]	0.17 [0.00, 0.38]	0.93 [0.88, 1.00]

Supplementary Table 8: Grid search results on the classification task on GDHCM retrospective dataset (choosing 50% as the threshold for clinical variables selection). Reported metrics are AUC, AUPRC, F1, Balanced Accuracy, MCC, Specificity, Sensitivity, PPV, and NPV.

Config	AUC	AUPRC	F1	Balanced_Accuracy	MCC	Specificity	Sensitivity	PPV	NPV
lr0.001_wd0.0001_h2048_d2	0.80 [0.70, 0.90]	0.47 [0.20, 0.61]	0.36 [0.22, 0.67]	0.68 [0.57, 0.84]	0.28 [0.12, 0.62]	0.81 [0.68, 0.93]	0.55 [0.25, 0.75]	0.29 [0.17, 0.60]	0.94 [0.90, 0.96]
lr0.001_wd0.001_h1024_d1	0.78 [0.65, 0.88]	0.46 [0.21, 0.64]	0.36 [0.20, 0.60]	0.67 [0.57, 0.82]	0.28 [0.13, 0.55]	0.72 [0.14, 0.90]	0.62 [0.33, 1.00]	0.29 [0.11, 0.50]	0.95 [0.92, 1.00]
lr0.001_wd0.001_h2048_d1	0.78 [0.65, 0.89]	0.37 [0.18, 0.50]	0.29 [0.00, 0.57]	0.61 [0.41, 0.73]	0.20 [-0.14, 0.53]	0.75 [0.31, 0.97]	0.47 [0.00, 1.00]	0.26 [0.00, 0.67]	0.93 [0.88, 1.00]
lr0.001_wd0.001_h1024_d2	0.81 [0.68, 0.90]	0.47 [0.28, 0.68]	0.35 [0.20, 0.50]	0.69 [0.57, 0.79]	0.27 [0.08, 0.44]	0.59 [0.14, 0.83]	0.78 [0.67, 1.00]	0.24 [0.11, 0.38]	0.96 [0.93, 1.00]
lr0.001_wd0.001_h2048_d2	0.81 [0.73, 0.85]	0.46 [0.28, 0.60]	0.39 [0.32, 0.55]	0.73 [0.66, 0.81]	0.33 [0.26, 0.49]	0.77 [0.54, 0.86]	0.68 [0.50, 1.00]	0.29 [0.19, 0.43]	0.95 [0.92, 1.00]
lr0.0001_wd0.0_h256_d3	0.77 [0.64, 0.85]	0.39 [0.19, 0.50]	0.30 [0.15, 0.50]	0.63 [0.50, 0.79]	0.18 [0.00, 0.44]	0.60 [0.00, 0.83]	0.65 [0.33, 1.00]	0.21 [0.10, 0.38]	0.75 [0.00, 0.96]
lr0.0001_wd0.0_h400_d3	0.76 [0.65, 0.86]	0.35 [0.18, 0.47]	0.20 [0.00, 0.44]	0.55 [0.38, 0.70]	0.09 [-0.18, 0.36]	0.69 [0.00, 1.00]	0.40 [0.00, 1.00]	0.16 [0.00, 0.40]	0.73 [0.00, 0.93]
lr0.0001_wd0.0_h512_d3	0.77 [0.69, 0.85]	0.42 [0.19, 0.60]	0.28 [0.20, 0.40]	0.64 [0.54, 0.77]	0.19 [0.07, 0.33]	0.66 [0.34, 0.86]	0.62 [0.25, 1.00]	0.19 [0.14, 0.27]	0.94 [0.89, 1.00]
lr0.0001_wd0.0_h256_d4	0.78 [0.67, 0.86]	0.40 [0.24, 0.50]	0.29 [0.18, 0.44]	0.60 [0.50, 0.75]	0.16 [0.00, 0.36]	0.49 [0.00, 0.90]	0.72 [0.33, 1.00]	0.21 [0.10, 0.40]	0.56 [0.00, 0.96]
lr0.0001_wd0.0_h400_d4	0.81 [0.71, 0.90]	0.44 [0.21, 0.53]	0.38 [0.22, 0.67]	0.67 [0.50, 0.84]	0.29 [0.00, 0.62]	0.63 [0.00, 0.93]	0.72 [0.33, 1.00]	0.31 [0.12, 0.60]	0.76 [0.00, 1.00]
lr0.0001_wd0.0_h512_d4	0.78 [0.65, 0.89]	0.42 [0.17, 0.55]	0.33 [0.15, 0.60]	0.64 [0.50, 0.82]	0.21 [0.00, 0.55]	0.64 [0.00, 0.90]	0.63 [0.33, 1.00]	0.24 [0.10, 0.50]	0.75 [0.00, 0.96]
lr0.0001_wd0.0001_h256_d3	0.77 [0.69, 0.88]	0.42 [0.19, 0.51]	0.28 [0.20, 0.40]	0.62 [0.50, 0.74]	0.16 [0.00, 0.33]	0.58 [0.00, 0.79]	0.65 [0.33, 1.00]	0.19 [0.12, 0.27]	0.75 [0.00, 0.95]
lr0.0001_wd0.0001_h400_d3	0.79 [0.71, 0.89]	0.42 [0.20, 0.58]	0.28 [0.00, 0.50]	0.63 [0.50, 0.79]	0.19 [0.00, 0.44]	0.67 [0.00, 1.00]	0.58 [0.00, 1.00]	0.20 [0.00, 0.38]	0.75 [0.00, 0.96]
lr0.0001_wd0.0001_h512_d3	0.78 [0.69, 0.88]	0.40 [0.19, 0.56]	0.24 [0.17, 0.40]	0.56 [0.50, 0.68]	0.09 [0.00, 0.31]	0.48 [0.00, 0.86]	0.63 [0.33, 1.00]	0.17 [0.11, 0.33]	0.55 [0.00, 0.93]
lr0.0001_wd0.0001_h256_d4	0.77 [0.70, 0.83]	0.38 [0.19, 0.48]	0.29 [0.20, 0.60]	0.60 [0.50, 0.82]	0.17 [0.00, 0.55]	0.67 [0.00, 0.90]	0.53 [0.25, 1.00]	0.23 [0.12, 0.50]	0.74 [0.00, 0.96]
lr0.0001_wd0.0001_h400_d4	0.79 [0.69, 0.87]	0.43 [0.20, 0.52]	0.33 [0.18, 0.67]	0.62 [0.50, 0.84]	0.21 [0.00, 0.62]	0.66 [0.00, 0.93]	0.58 [0.33, 1.00]	0.26 [0.12, 0.60]	0.74 [0.00, 0.96]
lr0.0001_wd0.0001_h512_d4	0.79 [0.67, 0.87]	0.40 [0.23, 0.53]	0.28 [0.00, 0.55]	0.60 [0.43, 0.81]	0.16 [-0.13, 0.49]	0.68 [0.00, 0.90]	0.52 [0.00, 1.00]	0.22 [0.00, 0.43]	0.74 [0.00, 0.96]
lr0.0001_wd0.001_h256_d3	0.78 [0.65, 0.86]	0.34 [0.19, 0.52]	0.17 [0.00, 0.33]	0.56 [0.48, 0.71]	0.08 [-0.07, 0.27]	0.69 [0.00, 1.00]	0.43 [0.00, 1.00]	0.11 [0.00, 0.22]	0.73 [0.00, 0.95]
lr0.0001_wd0.001_h400_d3	0.78 [0.68, 0.87]	0.43 [0.17, 0.55]	0.33 [0.18, 0.50]	0.63 [0.50, 0.79]	0.21 [0.00, 0.44]	0.67 [0.00, 0.90]	0.58 [0.33, 1.00]	0.25 [0.12, 0.40]	0.75 [0.00, 0.96]
lr0.0001_wd0.001_h512_d3	0.77 [0.69, 0.87]	0.34 [0.18, 0.48]	0.29 [0.00, 0.55]	0.61 [0.43, 0.81]	0.17 [-0.13, 0.49]	0.64 [0.00, 0.90]	0.58 [0.00, 1.00]	0.22 [0.00, 0.43]	0.74 [0.00, 0.96]
lr0.0001_wd0.001_h256_d4	0.79 [0.73, 0.87]	0.42 [0.20, 0.51]	0.20 [0.00, 0.36]	0.54 [0.50, 0.66]	0.07 [0.00, 0.26]	0.52 [0.00, 1.00]	0.57 [0.00, 1.00]	0.13 [0.00, 0.29]	0.55 [0.00, 0.92]
lr0.0001_wd0.001_h400_d4	0.78 [0.62, 0.90]	0.41 [0.15, 0.57]	0.29 [0.17, 0.55]	0.63 [0.50, 0.81]	0.18 [0.00, 0.49]	0.59 [0.00, 0.86]	0.67 [0.25, 1.00]	0.21 [0.11, 0.43]	0.75 [0.00, 1.00]
lr0.0001_wd0.001_h512_d4	0.80 [0.74, 0.88]	0.38 [0.21, 0.54]	0.30 [0.00, 0.67]	0.62 [0.50, 0.84]	0.21 [0.00, 0.62]	0.72 [0.00, 1.00]	0.52 [0.00, 1.00]	0.25 [0.00, 0.60]	0.74 [0.00, 0.96]
lr0.0003_wd0.0_h256_d3	0.78 [0.69, 0.85]	0.44 [0.21, 0.67]	0.24 [0.00, 0.44]	0.58 [0.46, 0.70]	0.12 [-0.09, 0.36]	0.69 [0.00, 0.93]	0.47 [0.00, 1.00]	0.19 [0.00, 0.40]	0.73 [0.00, 0.93]
lr0.0003_wd0.0_h400_d3	0.80 [0.71, 0.89]	0.41 [0.27, 0.55]	0.35 [0.20, 0.60]	0.67 [0.56, 0.82]	0.28 [0.08, 0.55]	0.83 [0.71, 0.97]	0.50 [0.25, 0.75]	0.32 [0.14, 0.50]	0.93 [0.90, 0.96]
lr0.0003_wd0.0_h512_d3	0.81 [0.68, 0.91]	0.46 [0.26, 0.69]	0.39 [0.31, 0.67]	0.70 [0.61, 0.84]	0.33 [0.22, 0.62]	0.83 [0.71, 0.97]	0.57 [0.25, 0.75]	0.35 [0.20, 0.60]	0.94 [0.90, 0.96]
lr0.0003_wd0.0_h256_d4	0.79 [0.70, 0.90]	0.43 [0.29, 0.54]	0.35 [0.18, 0.60]	0.67 [0.54, 0.82]	0.27 [0.06, 0.55]	0.83 [0.75, 0.90]	0.50 [0.25, 0.75]	0.29 [0.13, 0.50]	0.93 [0.90, 0.96]
lr0.0003_wd0.0_h400_d4	0.76 [0.64, 0.87]	0.36 [0.16, 0.51]	0.34 [0.00, 0.55]	0.66 [0.45, 0.81]	0.26 [-0.11, 0.49]	0.86 [0.83, 0.89]	0.47 [0.00, 0.75]	0.27 [0.00, 0.43]	0.93 [0.89, 0.96]
lr0.0003_wd0.0_h512_d4	0.79 [0.69, 0.88]	0.40 [0.25, 0.47]	0.33 [0.18, 0.46]	0.65 [0.54, 0.77]	0.23 [0.06, 0.40]	0.81 [0.75, 0.86]	0.48 [0.33, 0.75]	0.25 [0.13, 0.33]	0.93 [0.91, 0.96]
lr0.0003_wd0.0001_h256_d3	0.79 [0.68, 0.88]	0.41 [0.18, 0.55]	0.32 [0.00, 0.50]	0.62 [0.45, 0.79]	0.22 [-0.11, 0.44]	0.72 [0.00, 0.96]	0.52 [0.00, 1.00]	0.30 [0.00, 0.50]	0.74 [0.00, 0.96]
lr0.0003_wd0.0001_h400_d3	0.79 [0.69, 0.89]	0.39 [0.20, 0.51]	0.33 [0.14, 0.55]	0.62 [0.49, 0.81]	0.21 [-0.01, 0.49]	0.65 [0.00, 0.96]	0.58 [0.33, 1.00]	0.28 [0.09, 0.50]	0.74 [0.00, 0.96]
lr0.0003_wd0.0001_h512_d3	0.81 [0.65, 0.88]	0.51 [0.27, 0.73]	0.38 [0.18, 0.55]	0.69 [0.54, 0.81]	0.30 [0.06, 0.49]	0.84 [0.75, 0.89]	0.53 [0.33, 0.75]	0.30 [0.13, 0.43]	0.94 [0.91, 0.96]
lr0.0003_wd0.0001_h256_d4	0.77 [0.67, 0.84]	0.38 [0.20, 0.51]	0.30 [0.22, 0.40]	0.61 [0.50, 0.67]	0.19 [0.00, 0.36]	0.67 [0.00, 0.96]	0.55 [0.25, 1.00]	0.26 [0.12, 0.50]	0.74 [0.00, 0.95]
lr0.0003_wd0.0001_h400_d4	0.80 [0.73, 0.92]	0.44 [0.21, 0.62]	0.35 [0.22, 0.47]	0.72 [0.58, 0.84]	0.30 [0.12, 0.46]	0.68 [0.50, 0.82]	0.77 [0.33, 1.00]	0.23 [0.17, 0.31]	0.96 [0.92, 1.00]
lr0.0003_wd0.0001_h512_d4	0.80 [0.71, 0.84]	0.44 [0.29, 0.64]	0.28 [0.22, 0.36]	0.62 [0.50, 0.79]	0.18 [0.00, 0.34]	0.63 [0.00, 0.90]	0.62 [0.25, 1.00]	0.21 [0.12, 0.29]	0.75 [0.00, 1.00]
lr0.0003_wd0.001_h256_d3	0.81 [0.71, 0.88]	0.45 [0.23, 0.54]	0.44 [0.24, 0.55]	0.69 [0.62, 0.81]	0.42 [0.14, 0.56]	0.87 [0.57, 1.00]	0.50 [0.25, 0.75]	0.61 [0.14, 1.00]	0.93 [0.91, 0.96]
lr0.0003_wd0.001_h400_d3	0.75 [0.65, 0.87]	0.37 [0.16, 0.51]	0.38 [0.22, 0.60]	0.68 [0.58, 0.82]	0.30 [0.12, 0.55]	0.82 [0.64, 0.93]	0.55 [0.33, 0.75]	0.32 [0.17, 0.50]	0.94 [0.92, 0.96]
lr0.0003_wd0.001_h512_d3	0.79 [0.69, 0.91]	0.42 [0.25, 0.56]	0.36 [0.22, 0.67]	0.69 [0.58, 0.84]	0.30 [0.12, 0.62]	0.76 [0.54, 0.93]	0.62 [0.25, 1.00]	0.30 [0.17, 0.60]	0.95 [0.90, 1.00]
lr0.0003_wd0.001_h256_d4	0.79 [0.69, 0.89]	0.45 [0.19, 0.60]	0.39 [0.22, 0.57]	0.73 [0.58, 0.90]	0.33 [0.12, 0.56]	0.75 [0.46, 0.86]	0.72 [0.33, 1.00]	0.28 [0.17, 0.40]	0.96 [0.92, 1.00]
lr0.0003_wd0.001_h400_d4	0.78 [0.69, 0.90]	0.39 [0.21, 0.56]	0.36 [0.00, 0.67]	0.69 [0.43, 0.84]	0.28 [-0.13, 0.62]	0.79 [0.66, 0.93]	0.58 [0.00, 0.75]	0.27 [0.00, 0.60]	0.94 [0.89, 0.96]
lr0.0003_wd0.001_h512_d4	0.78 [0.69, 0.86]	0.46 [0.19, 0.65]	0.25 [0.18, 0.31]	0.57 [0.50, 0.65]	0.12 [0.00, 0.21]	0.47 [0.00, 0.93]	0.68 [0.25, 1.00]	0.19 [0.10, 0.33]	0.55 [0.00, 0.95]
lr0.001_wd0.0_h256_d3	0.79 [0.69, 0.87]	0.38 [0.25, 0.45]	0.40 [0.27, 0.60]	0.69 [0.61, 0.82]	0.33 [0.19, 0.55]	0.84 [0.64, 0.93]	0.55 [0.33, 0.75]	0.34 [0.17, 0.50]	0.94 [0.92, 0.96]
lr0.001_wd0.0_h400_d3	0.80 [0.74, 0.84]	0.38 [0.22, 0.52]	0.38 [0.21, 0.50]	0.72 [0.59, 0.79]	0.32 [0.14, 0.44]	0.65 [0.18, 0.83]	0.78 [0.67, 1.00]	0.26 [0.12, 0.38]	0.97 [0.95, 1.00]
lr0.001_wd0.0_h512_d3	0.82 [0.69, 0.87]	0.43 [0.33, 0.47]	0.30 [0.22, 0.40]	0.63 [0.50, 0.82]	0.18 [0.00, 0.39]	0.45 [0.00, 0.89]	0.82 [0.33, 1.00]	0.20 [0.12, 0.27]	0.58 [0.00, 1.00]
lr0.001_wd0.0_h256_d4	0.82 [0.73, 0.87]	0.38 [0.33, 0.46]	0.36 [0.22, 0.47]	0.74 [0.58, 0.84]	0.32 [0.12, 0.46]	0.67 [0.52, 0.82]	0.82 [0.33, 1.00]	0.24 [0.17, 0.31]	0.97 [0.92, 1.00]
lr0.001_wd0.0_h400_d4	0.83 [0.71, 0.96]	0.51 [0.46, 0.65]	0.35 [0.20, 0.62]	0.69 [0.56, 0.91]	0.28 [0.08, 0.61]	0.80 [0.66, 0.89]	0.58 [0.25, 1.00]	0.26 [0.14, 0.44]	0.95 [0.89, 1.00]
lr0.001_wd0.0_h512_d4	0.81 [0.68, 0.91]	0.46 [0.21, 0.70]	0.37 [0.25, 0.46]	0.75 [0.68, 0.77]	0.33 [0.23, 0.40]	0.59 [0.36, 0.79]	0.90 [0.75, 1.00]	0.24 [0.14, 0.33]	0.98 [0.96, 1.00]
lr0.001_wd0.0001_h256_d3	0.81 [0.76, 0.88]	0.51 [0.27, 0.71]	0.32 [0.20, 0.50]	0.65 [0.50, 0.78]	0.22 [0.00, 0.43]	0.60 [0.00, 0.93]	0.70 [0.33, 1.00]	0.24 [0.12, 0.50]	0.76 [0.00, 1.00]
lr0.001_wd0.0001_h400_d3	0.83 [0.74, 0.90]	0.45 [0.33, 0.64]	0.35 [0.22, 0.50]	0.68 [0.57, 0.80]	0.27 [0.12, 0.44]	0.80 [0.61, 0.90]	0.57 [0.25, 1.00]	0.27 [0.17, 0.38]	0.94 [0.90, 1.00]
lr0.001_wd0.0001_h512_d3	0.82 [0.71, 0.89]	0.45 [0.31, 0.60]	0.41 [0.35, 0.50]	0.74 [0.70, 0.84]	0.35 [0.27, 0.43]	0.76 [0.66, 0.93]	0.73 [0.50, 1.00]	0.30 [0.23, 0.50]	0.96 [0.93, 1.00]
lr0.001_wd0.0001_h256_d4	0.81 [0.70, 0.87]	0.38 [0.26, 0.45]	0.35 [0.24, 0.46]	0.71 [0.61, 0.79]	0.31 [0.14, 0.40]	0.69 [0.54, 0.97]	0.73 [0.25, 1.00]	0.28 [0.14, 0.50]	0.96 [0.90, 1.00]
lr0.001_wd0.0001_h400_d4	0.81 [0.67, 0.89]	0.39 [0.27, 0.48]	0.34 [0.18, 0.50]	0.70 [0.50, 0.82]	0.27 [0.00, 0.44]	0.59 [0.00, 0.90]	0.80 [0.25, 1.00]	0.24 [0.10, 0.38]	0.77 [0.00, 1.00]
lr0.001_wd0.0001_h512_d4	0.85 [0.65, 0.92]	0.48 [0.40, 0.58]	0.38 [0.22, 0.57]	0.76 [0.58, 0.90]	0.35 [0.12, 0.56]	0.65 [0.36, 0.82]	0.87 [0.33, 1.00]	0.25 [0.14, 0.40]	0.98 [0.92, 1.00]
lr0.001_wd0.001_h256_d3	0.82 [0.74, 0.87]	0.38 [0.29, 0.47]	0.34 [0.20, 0.40]	0.71 [0.56, 0.79]	0.28 [0.08, 0.38]	0.66 [0.50, 0.79]	0.77 [0.33, 1.00]	0.22 [0.14, 0.27]	0.96 [0.92, 1.00]
lr0.001_wd0.001_h400_d3	0.82 [0.75, 0.91]	0.44 [0.25, 0.63]	0.41 [0.29, 0.57]	0.79 [0.67, 0.90]	0.38 [0.21, 0.56]	0.69 [0.62, 0.79]	0.88 [0.67, 1.00]	0.27 [0.18, 0.40]	0.98 [0.95, 1.00]
lr0.001_wd0.001_h512_d3	0.81 [0.71, 0.86]	0.41 [0.32, 0.51]	0.35 [0.20, 0.44]	0.69 [0.57, 0.82]	0.28 [0.08, 0.39]	0.70 [0.46, 0.90]	0.68 [0.50, 1.00]	0.26 [0.12, 0.40]	0.95 [0.92, 1.00]
lr0.001_wd0.001_h256_d4	0.80 [0.65, 0.89]	0.40 [0.28, 0.57]	0.37 [0.19, 0.50]	0.69 [0.55, 0.77]	0.29 [0.06, 0.43]	0.71 [0.43, 0.93]	0.67 [0.50, 0.75]	0.28 [0.11, 0.50]	0.94 [0.92, 0.96]
lr0.001_wd0.001_h400_d4	0.83 [0.70, 0.92]	0.46 [0.32, 0.58]	0.34 [0.25, 0.46]	0.71 [0.67, 0.77]	0.28 [0.21, 0.40]	0.59 [0.36, 0.79]	0.83 [0.67, 1.00]	0.22 [0.14, 0.33]	0.97 [0.95, 1.00]
lr0.001_wd0.001_h512_d4	0.80 [0.64, 0.86]	0.46 [0.33, 0.80]	0.34 [0.20, 0.40]	0.70 [0.56, 0.82]	0.26 [0.08, 0.39]	0.69 [0.55, 0.79]	0.72 [0.33, 1.00]	0.22 [0.14, 0.27]	0.95 [0.92, 1.00]

Supplementary Table 9: Grid search results on the classification task on GDHCM retrospective dataset (choosing 50% as the threshold for clinical variables selection). Reported metrics are AUC, AUPRC, F1, Balanced Accuracy, MCC, Specificity, Sensitivity, PPV, and NPV.

Config	AUC	AUPRC	F1	Balanced_Accuracy	MCC	Specificity	Sensitivity	PPV	NPV
lr0.0001_wd0.0_h256_d1	0.77 [0.68, 0.88]	0.46 [0.32, 0.62]	0.32 [0.25, 0.44]	0.60 [0.55, 0.68]	0.18 [0.10, 0.32]	0.69 [0.26, 0.85]	0.52 [0.25, 1.00]	0.26 [0.18, 0.40]	0.90 [0.85, 1.00]
lr0.0001_wd0.0_h400_d1	0.77 [0.68, 0.88]	0.54 [0.26, 0.73]	0.40 [0.33, 0.55]	0.68 [0.60, 0.78]	0.30 [0.20, 0.45]	0.58 [0.20, 0.85]	0.78 [0.50, 1.00]	0.29 [0.20, 0.43]	0.95 [0.89, 1.00]
lr0.0001_wd0.0_h512_d1	0.77 [0.66, 0.93]	0.57 [0.33, 0.72]	0.45 [0.38, 0.60]	0.72 [0.65, 0.89]	0.35 [0.22, 0.58]	0.76 [0.55, 0.85]	0.68 [0.50, 1.00]	0.35 [0.25, 0.43]	0.93 [0.89, 1.00]
lr0.0001_wd0.0_h256_d2	0.81 [0.71, 0.95]	0.57 [0.39, 0.76]	0.41 [0.26, 0.50]	0.68 [0.55, 0.84]	0.31 [0.13, 0.48]	0.57 [0.11, 0.90]	0.80 [0.50, 1.00]	0.31 [0.15, 0.50]	0.96 [0.89, 1.00]
lr0.0001_wd0.0_h400_d2	0.77 [0.62, 0.91]	0.54 [0.24, 0.71]	0.35 [0.27, 0.44]	0.64 [0.57, 0.71]	0.22 [0.10, 0.32]	0.49 [0.20, 0.85]	0.78 [0.50, 1.00]	0.24 [0.17, 0.40]	0.94 [0.89, 1.00]
lr0.0001_wd0.0_h512_d2	0.78 [0.70, 0.86]	0.52 [0.29, 0.73]	0.37 [0.33, 0.44]	0.65 [0.61, 0.68]	0.25 [0.22, 0.32]	0.65 [0.25, 0.89]	0.65 [0.33, 1.00]	0.29 [0.21, 0.40]	0.93 [0.89, 1.00]
lr0.0001_wd0.0001_h256_d1	0.77 [0.65, 0.89]	0.49 [0.29, 0.69]	0.39 [0.22, 0.57]	0.67 [0.53, 0.82]	0.26 [0.05, 0.50]	0.66 [0.45, 0.89]	0.68 [0.25, 1.00]	0.29 [0.20, 0.50]	0.92 [0.84, 1.00]
lr0.0001_wd0.0001_h400_d1	0.78 [0.66, 0.96]	0.58 [0.30, 0.87]	0.39 [0.27, 0.44]	0.68 [0.57, 0.79]	0.28 [0.10, 0.40]	0.63 [0.45, 0.85]	0.73 [0.50, 1.00]	0.29 [0.17, 0.40]	0.94 [0.89, 1.00]
lr0.0001_wd0.0001_h512_d1	0.79 [0.57, 0.93]	0.59 [0.22, 0.74]	0.51 [0.36, 0.75]	0.75 [0.65, 0.95]	0.42 [0.22, 0.73]	0.68 [0.30, 0.90]	0.83 [0.67, 1.00]	0.39 [0.22, 0.60]	0.96 [0.92, 1.00]
lr0.0001_wd0.0001_h256_d2	0.79 [0.68, 0.93]	0.48 [0.27, 0.59]	0.35 [0.25, 0.46]	0.63 [0.53, 0.82]	0.21 [0.08, 0.44]	0.52 [0.05, 0.90]	0.75 [0.25, 1.00]	0.25 [0.14, 0.33]	0.93 [0.86, 1.00]
lr0.0001_wd0.0001_h400_d2	0.78 [0.70, 0.93]	0.52 [0.28, 0.61]	0.32 [0.00, 0.57]	0.60 [0.42, 0.78]	0.17 [-0.17, 0.50]	0.69 [0.32, 0.89]	0.52 [0.00, 0.75]	0.25 [0.00, 0.50]	0.88 [0.81, 0.94]
lr0.0001_wd0.0001_h512_d2	0.77 [0.68, 0.84]	0.50 [0.33, 0.61]	0.46 [0.38, 0.57]	0.74 [0.68, 0.85]	0.37 [0.30, 0.53]	0.70 [0.47, 0.85]	0.78 [0.50, 1.00]	0.34 [0.23, 0.40]	0.95 [0.89, 1.00]
lr0.0001_wd0.001_h256_d1	0.78 [0.62, 0.89]	0.45 [0.26, 0.62]	0.40 [0.30, 0.67]	0.69 [0.55, 0.90]	0.29 [0.08, 0.63]	0.53 [0.35, 0.80]	0.85 [0.50, 1.00]	0.27 [0.19, 0.50]	0.95 [0.87, 1.00]
lr0.0001_wd0.001_h400_d1	0.77 [0.68, 0.88]	0.54 [0.32, 0.71]	0.39 [0.29, 0.47]	0.68 [0.59, 0.78]	0.28 [0.16, 0.41]	0.71 [0.55, 0.84]	0.65 [0.33, 1.00]	0.29 [0.25, 0.33]	0.93 [0.89, 1.00]
lr0.0001_wd0.001_h512_d1	0.75 [0.69, 0.85]	0.44 [0.28, 0.57]	0.41 [0.36, 0.53]	0.71 [0.65, 0.82]	0.33 [0.26, 0.49]	0.59 [0.30, 0.80]	0.83 [0.50, 1.00]	0.29 [0.22, 0.36]	**0.96 [0.89, 1.00]**
lr0.0001_wd0.001_h256_d2	0.81 [0.72, 0.96]	0.58 [0.33, 0.81]	0.42 [0.31, 0.55]	0.69 [0.55, 0.87]	0.32 [0.13, 0.53]	0.55 [0.10, 0.90]	0.83 [0.50, 1.00]	0.31 [0.18, 0.50]	0.97 [0.90, 1.00]
lr0.0001_wd0.001_h400_d2	0.79 [0.74, 0.88]	0.55 [0.31, 0.73]	0.42 [0.27, 0.50]	0.70 [0.58, 0.80]	0.32 [0.16, 0.45]	0.61 [0.16, 0.84]	0.78 [0.50, 1.00]	0.30 [0.16, 0.40]	0.95 [0.89, 1.00]
lr0.0001_wd0.001_h512_d2	0.77 [0.68, 0.95]	0.51 [0.33, 0.71]	0.26 [0.00, 0.40]	0.58 [0.45, 0.70]	0.14 [-0.13, 0.34]	0.71 [0.40, 0.95]	0.45 [0.00, 1.00]	0.23 [0.00, 0.50]	0.89 [0.82, 1.00]
lr0.0003_wd0.0_h256_d1	0.77 [0.71, 0.89]	0.45 [0.32, 0.67]	0.30 [0.00, 0.50]	0.61 [0.45, 0.74]	0.17 [-0.13, 0.40]	0.64 [0.42, 0.90]	0.58 [0.00, 1.00]	0.22 [0.00, 0.50]	0.90 [0.82, 1.00]
lr0.0003_wd0.0_h400_d1	0.81 [0.72, 0.96]	0.52 [0.34, 0.81]	0.40 [0.36, 0.46]	0.70 [0.62, 0.82]	**0.31 [0.20, 0.44]**	0.57 [0.30, 0.75]	0.83 [0.50, 1.00]	0.27 [0.22, 0.30]	0.96 [0.88, 1.00]
lr0.0003_wd0.0_h512_d1	0.83 [0.69, 0.89]	0.56 [0.43, 0.77]	0.42 [0.29, 0.55]	0.68 [0.59, 0.78]	0.30 [0.16, 0.45]	0.81 [0.70, 0.85]	0.55 [0.33, 0.75]	0.35 [0.25, 0.43]	0.91 [0.88, 0.94]
lr0.0003_wd0.0_h256_d2	0.75 [0.70, 0.84]	0.46 [0.31, 0.72]	0.30 [0.22, 0.36]	0.60 [0.53, 0.65]	0.16 [0.05, 0.26]	**0.54 [0.30, 0.84]**	0.65 [0.25, 1.00]	0.21 [0.17, 0.25]	0.93 [0.84, 1.00]
lr0.0003_wd0.0_h400_d2	0.78 [0.69, 0.93]	0.57 [0.31, 0.72]	0.41 [0.27, 0.57]	0.67 [0.58, 0.78]	0.34 [0.16, 0.50]	0.61 [0.16, 1.00]	0.73 [0.25, 1.00]	0.43 [0.16, 1.00]	0.95 [0.87, 1.00]
lr0.0003_wd0.0_h512_d2	0.77 [0.57, 0.89]	0.51 [0.22, 0.74]	0.46 [0.33, 0.67]	0.71 [0.62, 0.92]	0.35 [0.19, 0.65]	0.74 [0.50, 0.90]	0.68 [0.50, 1.00]	0.37 [0.22, 0.50]	0.93 [0.89, 1.00]
lr0.0003_wd0.0001_h256_d1	0.77 [0.64, 0.88]	0.58 [0.42, 0.69]	0.39 [0.24, 0.46]	0.68 [0.52, 0.79]	0.28 [0.03, 0.40]	0.57 [0.35, 0.85]	0.78 [0.50, 1.00]	0.28 [0.14, 0.40]	**0.94 [0.88, 1.00]**
lr0.0003_wd0.0001_h400_d1	0.82 [0.71, 0.89]	0.54 [0.31, 0.66]	0.45 [0.33, 0.60]	0.72 [0.60, 0.89]	0.36 [0.25, 0.58]	0.76 [0.60, 0.95]	0.68 [0.25, 1.00]	0.38 [0.25, 0.50]	0.93 [0.86, 1.00]
lr0.0003_wd0.0001_h512_d1	0.78 [0.68, 0.96]	0.59 [0.34, 0.87]	0.34 [0.24, 0.44]	0.63 [0.52, 0.76]	0.20 [0.03, 0.36]	0.67 [0.37, 0.85]	0.58 [0.25, 1.00]	0.27 [0.14, 0.40]	0.90 [0.85, 1.00]
lr0.0003_wd0.0001_h256_d2	0.77 [0.69, 0.91]	0.50 [0.30, 0.72]	0.37 [0.00, 0.57]	0.68 [0.45, 0.82]	0.29 [-0.13, 0.51]	0.72 [0.32, 0.95]	0.63 [0.00, 1.00]	0.31 [0.00, 0.67]	0.93 [0.82, 1.00]
lr0.0003_wd0.0001_h400_d2	0.81 [0.71, 0.96]	0.61 [0.33, 0.81]	0.43 [0.21, 0.75]	0.68 [0.46, 0.95]	0.30 [-0.05, 0.73]	0.62 [0.26, 0.89]	0.73 [0.50, 1.00]	0.33 [0.12, 0.60]	0.92 [0.83, 1.00]
lr0.0003_wd0.0001_h512_d2	0.81 [0.71, 0.93]	0.50 [0.29, 0.77]	0.41 [0.33, 0.60]	0.69 [0.60, 0.89]	0.32 [0.16, 0.58]	0.70 [0.40, 0.95]	0.68 [0.25, 1.00]	0.34 [0.25, 0.50]	0.93 [0.86, 1.00]
lr0.0003_wd0.001_h256_d1	0.79 [0.65, 0.89]	0.52 [0.31, 0.72]	0.42 [0.35, 0.50]	0.68 [0.62, 0.75]	0.30 [0.23, 0.42]	0.70 [0.25, 0.85]	0.67 [0.50, 1.00]	0.33 [0.21, 0.40]	0.93 [0.89, 1.00]
lr0.0003_wd0.001_h400_d1	0.79 [0.70, 0.89]	0.44 [0.35, 0.58]	0.33 [0.00, 0.50]	0.63 [0.45, 0.76]	0.21 [-0.13, 0.40]	0.76 [0.53, 0.90]	0.50 [0.00, 1.00]	0.27 [0.00, 0.50]	0.90 [0.85, 1.00]
lr0.0003_wd0.001_h512_d1	0.78 [0.64, 0.96]	0.48 [0.24, 0.81]	0.42 [0.29, 0.50]	0.71 [0.60, 0.84]	0.33 [0.13, 0.48]	0.58 [0.40, 0.90]	0.83 [0.50, 1.00]	0.30 [0.18, 0.50]	0.96 [0.90, 1.00]
lr0.0003_wd0.001_h256_d2	0.77 [0.66, 0.85]	0.48 [0.30, 0.71]	0.31 [0.22, 0.36]	0.60 [0.53, 0.65]	0.18 [0.05, 0.26]	0.71 [0.30, 0.90]	0.50 [0.25, 1.00]	**0.26 [0.20, 0.33]**	0.90 [0.84, 1.00]
lr0.0003_wd0.001_h400_d2	0.80 [0.70, 0.93]	0.49 [0.28, 0.66]	0.35 [0.00, 0.55]	0.68 [0.47, 0.87]	0.27 [-0.09, 0.53]	0.73 [0.45, 0.95]	0.63 [0.00, 1.00]	0.25 [0.00, 0.38]	0.93 [0.83, 1.00]
lr0.0003_wd0.001_h512_d2	0.78 [0.70, 0.88]	0.49 [0.36, 0.62]	0.37 [0.26, 0.43]	0.64 [0.55, 0.70]	0.26 [0.13, 0.34]	0.56 [0.11, 0.95]	0.72 [0.33, 1.00]	0.30 [0.15, 0.50]	0.94 [0.89, 1.00]
lr0.001_wd0.0_h256_d1	0.75 [0.64, 0.86]	0.53 [0.25, 0.73]	0.43 [0.35, 0.50]	0.70 [0.62, 0.75]	0.33 [0.19, 0.42]	0.62 [0.42, 0.90]	0.78 [0.50, 1.00]	0.32 [0.21, 0.50]	0.95 [0.90, 1.00]
lr0.001_wd0.0_h400_d1	0.84 [0.65, 0.98]	0.63 [0.31, 0.92]	0.39 [0.38, 0.40]	0.69 [0.65, 0.74]	0.28 [0.22, 0.33]	0.59 [0.47, 0.74]	0.78 [0.67, 1.00]	0.26 [0.23, 0.29]	0.94 [0.92, 1.00]
lr0.001_wd0.0_h512_d1	0.74 [0.64, 0.89]	0.46 [0.25, 0.67]	0.44 [0.29, 0.67]	0.68 [0.61, 0.75]	0.35 [0.19, 0.67]	0.58 [0.21, 1.00]	0.78 [0.50, 1.00]	0.41 [0.17, 1.00]	0.95 [0.91, 1.00]
lr0.001_wd0.0_h256_d2	0.78 [0.67, 0.86]	0.53 [0.44, 0.65]	0.45 [0.33, 0.60]	0.69 [0.60, 0.80]	0.37 [0.20, 0.52]	**0.88 [0.75, 0.95]**	0.50 [0.25, 0.75]	**0.46 [0.29, 0.50]**	0.91 [0.86, 0.94]
lr0.001_wd0.0_h400_d2	0.76 [0.65, 0.88]	0.45 [0.27, 0.61]	0.31 [0.00, 0.55]	0.65 [0.50, 0.78]	0.23 [0.00, 0.45]	0.74 [0.47, 1.00]	0.55 [0.00, 1.00]	0.24 [0.00, 0.43]	0.92 [0.85, 1.00]
lr0.001_wd0.0_h512_d2	**0.81 [0.67, 0.88]**	0.54 [0.43, 0.70]	0.29 [0.00, 0.44]	0.61 [0.47, 0.68]	0.17 [-0.09, 0.32]	0.64 [0.11, 0.95]	**0.58 [0.00, 1.00]**	0.21 [0.00, 0.40]	**0.91 [0.83, 1.00]**
lr0.001_wd0.0001_h256_d1	0.80 [0.69, 0.95]	0.44 [0.28, 0.83]	0.42 [0.25, 0.55]	0.71 [0.56, 0.87]	0.32 [0.10, 0.53]	0.60 [0.15, 0.79]	0.82 [0.33, 1.00]	0.29 [0.19, 0.38]	0.96 [0.88, 1.00]
lr0.001_wd0.0001_h400_d1	0.80 [0.69, 0.96]	0.57 [0.37, 0.81]	0.41 [0.35, 0.50]	**0.71 [0.62, 0.82]**	0.32 [0.20, 0.44]	0.57 [0.30, 0.75]	**0.85 [0.50, 1.00]**	0.28 [0.21, 0.38]	**0.96 [0.88, 1.00]**
lr0.001_wd0.0001_h512_d1	0.79 [0.70, 0.89]	0.44 [0.28, 0.62]	0.49 [0.35, 0.67]	0.77 [0.70, 0.82]	0.43 [0.30, 0.60]	0.63 [0.40, 0.90]	**0.90 [0.75, 1.00]**	0.37 [0.21, 0.60]	**0.98 [0.94, 1.00]**
lr0.001_wd0.0001_h256_d2	0.78 [0.71, 0.85]	0.49 [0.33, 0.62]	0.39 [0.22, 0.67]	0.65 [0.53, 0.81]	0.27 [0.05, 0.61]	0.79 [0.58, 0.95]	0.52 [0.25, 0.75]	0.35 [0.20, 0.67]	0.90 [0.84, 0.95]
lr0.001_wd0.0001_h400_d2	0.80 [0.73, 0.84]	0.53 [0.40, 0.73]	0.44 [0.35, 0.53]	0.71 [0.62, 0.82]	0.34 [0.23, 0.49]	0.66 [0.25, 0.85]	0.77 [0.50, 1.00]	0.32 [0.21, 0.40]	**0.95 [0.89, 1.00]**
lr0.001_wd0.0001_h512_d2	0.77 [0.71, 0.86]	0.54 [0.42, 0.72]	0.44 [0.30, 0.57]	0.69 [0.57, 0.78]	0.33 [0.17, 0.50]	0.59 [0.15, 0.89]	0.78 [0.50, 1.00]	0.34 [0.18, 0.50]	0.96 [0.89, 1.00]
lr0.001_wd0.001_h256_d1	0.76 [0.63, 0.98]	0.51 [0.24, 0.92]	0.36 [0.00, 0.62]	0.66 [0.42, 0.88]	0.25 [-0.16, 0.58]	0.63 [0.25, 0.84]	0.68 [0.00, 1.00]	0.25 [0.00, 0.44]	0.94 [0.84, 1.00]
lr0.001_wd0.001_h400_d1	0.82 [0.65, 0.98]	0.58 [0.36, 0.92]	0.43 [0.27, 0.86]	0.67 [0.50, 0.97]	0.28 [0.00, 0.84]	0.50 [0.15, 0.95]	0.83 [0.67, 1.00]	0.32 [0.17, 0.75]	**0.93 [0.83, 1.00]**
lr0.001_wd0.001_h256_d2	0.82 [0.70, 0.88]	0.56 [0.39, 0.75]	0.42 [0.33, 0.57]	0.68 [0.61, 0.73]	0.33 [0.22, 0.51]	0.72 [0.40, 0.95]	0.65 [0.33, 1.00]	0.37 [0.25, 0.67]	0.93 [0.89, 1.00]
lr0.001_wd0.001_h400_d2	**0.85 [0.71, 0.96]**	0.53 [0.34, 0.81]	**0.34 [0.00, 0.67]**	0.64 [0.50, 0.92]	**0.28 [0.00, 0.65]**	0.68 [0.20, 1.00]	0.60 [0.00, 1.00]	0.38 [0.00, 1.00]	0.92 [0.86, 1.00]
lr0.001_wd0.001_h512_d2	0.79 [0.71, 0.88]	0.52 [0.33, 0.72]	0.46 [0.40, 0.50]	0.73 [0.70, 0.78]	0.37 [0.30, 0.42]	0.70 [0.45, 0.90]	0.77 [0.50, 1.00]	0.35 [0.27, 0.50]	0.95 [0.90, 1.00]
**lr0.001_wd0.001_h512_d1**	0.82 [0.64, 0.96]	**0.65 [0.25, 0.87]**	**0.54 [0.42, 0.75]**	**0.79 [0.68, 0.92]**	**0.48 [0.32, 0.70]**	0.73 [0.45, 0.95]	0.85 [0.50, 1.00]	0.44 [0.27, 0.75]	0.97 [0.89, 1.00]
Rank	4	1	1	1	1	6	2	2	2

Supplementary Table 10: Grid search results on the classification task on GDHCM prospective dataset (choosing 50% as the threshold for clinical variables selection). Reported metrics are AUC, AUPRC, F1, Balanced Accuracy, MCC, Specificity, Sensitivity, PPV, and NPV. Bold values indicate the best performance across all settings, and underlined values denote the second-best performance. The second-to-last row corresponds to the hyperparameter configuration we selected. The row labeled *Rank* indicates the relative position of the selected hyperparameter configuration among all candidate settings based on its validation performance across each metric.

Config	AUC	AUPRC	F1	Balanced_Accuracy	MCC	Specificity	Sensitivity	PPV	NPV
lr1e-05_wd0.005_h1024_d3	0.73 [0.65, 0.88]	0.47 [0.25, 0.61]	0.31 [0.20, 0.43]	0.59 [0.50, 0.74]	0.13 [0.00, 0.33]	0.37 [0.00, 0.75]	0.80 [0.25, 1.00]	0.20 [0.14, 0.30]	0.55 [0.00, 1.00]
lr1e-05_wd0.005_h2048_d3	0.78 [0.68, 0.93]	0.58 [0.32, 0.72]	0.26 [0.00, 0.40]	0.57 [0.43, 0.76]	0.08 [-0.17, 0.36]	0.40 [0.00, 0.85]	0.73 [0.00, 1.00]	0.16 [0.00, 0.25]	0.55 [0.00, 1.00]
lr1e-05_wd0.005_h1024_d4	0.78 [0.68, 0.91]	0.58 [0.32, 0.71]	0.34 [0.29, 0.43]	0.60 [0.50, 0.79]	0.14 [0.00, 0.40]	0.26 [0.00, 0.74]	0.93 [0.67, 1.00]	0.21 [0.17, 0.29]	0.39 [0.00, 1.00]
lr1e-05_wd0.005_h2048_d4	0.76 [0.67, 0.91]	0.52 [0.30, 0.71]	0.39 [0.25, 0.50]	0.69 [0.55, 0.82]	0.29 [0.10, 0.44]	0.64 [0.40, 0.85]	0.73 [0.25, 1.00]	0.29 [0.23, 0.40]	0.94 [0.85, 1.00]
lr1e-05_wd0.01_h1024_d3	0.74 [0.66, 0.84]	0.50 [0.26, 0.65]	0.28 [0.00, 0.43]	0.60 [0.50, 0.79]	0.14 [0.00, 0.40]	0.40 [0.00, 1.00]	0.80 [0.00, 1.00]	0.17 [0.00, 0.27]	0.57 [0.00, 1.00]
lr1e-05_wd0.01_h2048_d3	0.74 [0.64, 0.86]	0.56 [0.32, 0.71]	0.36 [0.27, 0.46]	0.64 [0.50, 0.74]	0.21 [0.00, 0.35]	0.49 [0.00, 0.80]	0.78 [0.50, 1.00]	0.25 [0.17, 0.33]	0.74 [0.00, 1.00]
lr1e-05_wd0.01_h1024_d4	0.73 [0.65, 0.91]	0.50 [0.25, 0.72]	0.28 [0.00, 0.43]	0.61 [0.50, 0.79]	0.15 [0.00, 0.40]	0.58 [0.00, 1.00]	0.63 [0.00, 1.00]	0.19 [0.00, 0.29]	0.73 [0.00, 1.00]
lr1e-05_wd0.01_h2048_d4	0.77 [0.66, 0.95]	0.49 [0.26, 0.64]	0.24 [0.00, 0.67]	0.58 [0.43, 0.92]	0.12 [-0.17, 0.65]	0.71 [0.00, 1.00]	0.45 [0.00, 1.00]	0.18 [0.00, 0.50]	0.70 [0.00, 1.00]
lr3e-05_wd0.005_h1024_d3	0.80 [0.67, 0.88]	0.52 [0.38, 0.60]	0.34 [0.00, 0.50]	0.65 [0.50, 0.75]	0.26 [0.00, 0.40]	0.79 [0.45, 1.00]	0.52 [0.00, 1.00]	0.28 [0.00, 0.40]	0.92 [0.86, 1.00]
lr3e-05_wd0.005_h2048_d3	0.79 [0.67, 0.93]	0.55 [0.37, 0.66]	0.37 [0.22, 0.55]	0.67 [0.53, 0.87]	0.25 [0.05, 0.53]	0.61 [0.47, 0.80]	0.73 [0.25, 1.00]	0.26 [0.17, 0.38]	0.93 [0.84, 1.00]
lr3e-05_wd0.005_h1024_d4	0.77 [0.70, 0.91]	0.44 [0.26, 0.58]	0.32 [0.00, 0.50]	0.63 [0.50, 0.78]	0.24 [0.00, 0.41]	0.70 [0.15, 1.00]	0.57 [0.00, 1.00]	0.27 [0.00, 0.50]	0.93 [0.86, 1.00]
lr3e-05_wd0.005_h2048_d4	0.79 [0.68, 0.95]	0.59 [0.36, 0.71]	0.40 [0.30, 0.57]	0.64 [0.53, 0.78]	0.30 [0.09, 0.50]	0.64 [0.05, 0.95]	0.65 [0.25, 1.00]	0.38 [0.17, 0.50]	0.94 [0.86, 1.00]
lr3e-05_wd0.01_h1024_d3	0.76 [0.67, 0.85]	0.57 [0.36, 0.71]	0.32 [0.23, 0.44]	0.60 [0.40, 0.75]	0.13 [-0.27, 0.38]	0.47 [0.05, 0.85]	0.73 [0.25, 1.00]	0.22 [0.14, 0.29]	0.86 [0.50, 1.00]
lr3e-05_wd0.01_h2048_d3	0.80 [0.77, 0.88]	0.52 [0.38, 0.65]	0.33 [0.24, 0.44]	0.59 [0.50, 0.68]	0.15 [0.00, 0.32]	0.41 [0.00, 0.85]	0.77 [0.33, 1.00]	0.24 [0.14, 0.40]	0.56 [0.00, 1.00]
lr3e-05_wd0.01_h1024_d4	0.74 [0.68, 0.89]	0.50 [0.29, 0.71]	0.36 [0.25, 0.46]	0.66 [0.55, 0.82]	0.24 [0.10, 0.44]	0.58 [0.15, 0.85]	0.73 [0.25, 1.00]	0.25 [0.19, 0.30]	0.94 [0.85, 1.00]
lr3e-05_wd0.01_h2048_d4	0.77 [0.70, 0.96]	0.56 [0.30, 0.87]	0.23 [0.00, 0.40]	0.56 [0.50, 0.76]	0.09 [0.00, 0.36]	0.63 [0.00, 1.00]	0.50 [0.00, 1.00]	0.17 [0.00, 0.25]	0.71 [0.00, 1.00]
lr1e-05_wd0.0_h256_d1	0.79 [0.63, 0.98]	0.56 [0.25, 0.92]	0.27 [0.21, 0.35]	0.55 [0.43, 0.63]	0.09 [-0.12, 0.23]	0.34 [0.05, 0.80]	0.75 [0.25, 1.00]	0.17 [0.13, 0.21]	0.92 [0.78, 1.00]
lr1e-05_wd0.0_h400_d1	0.72 [0.65, 0.89]	0.48 [0.25, 0.64]	0.19 [0.00, 0.35]	0.53 [0.38, 0.68]	0.03 [-0.23, 0.27]	0.56 [0.25, 0.95]	0.50 [0.00, 1.00]	0.12 [0.00, 0.23]	0.88 [0.79, 1.00]
lr1e-05_wd0.0_h512_d1	0.72 [0.66, 0.84]	0.49 [0.27, 0.71]	0.25 [0.00, 0.35]	0.56 [0.40, 0.71]	0.08 [-0.20, 0.30]	0.49 [0.30, 0.80]	0.63 [0.00, 1.00]	0.15 [0.00, 0.21]	0.89 [0.80, 1.00]
lr1e-05_wd0.0_h256_d2	0.80 [0.71, 0.93]	0.57 [0.29, 0.81]	0.30 [0.00, 0.46]	0.62 [0.43, 0.82]	0.19 [-0.17, 0.44]	0.45 [0.05, 0.85]	0.80 [0.00, 1.00]	0.19 [0.00, 0.30]	0.96 [0.81, 1.00]
lr1e-05_wd0.0_h400_d2	0.75 [0.69, 0.82]	0.47 [0.30, 0.61]	0.27 [0.00, 0.42]	0.60 [0.43, 0.73]	0.13 [-0.17, 0.35]	0.51 [0.32, 0.85]	0.68 [0.00, 1.00]	0.17 [0.00, 0.27]	0.92 [0.81, 1.00]
lr1e-05_wd0.0_h512_d2	0.82 [0.74, 0.88]	0.48 [0.33, 0.66]	0.26 [0.00, 0.40]	0.57 [0.48, 0.70]	0.13 [-0.09, 0.30]	0.56 [0.00, 0.95]	0.58 [0.00, 1.00]	0.22 [0.00, 0.50]	0.72 [0.00, 1.00]
lr1e-05_wd0.0001_h256_d1	0.70 [0.58, 0.82]	0.44 [0.19, 0.60]	0.30 [0.20, 0.33]	0.60 [0.50, 0.68]	0.15 [0.00, 0.27]	0.46 [0.35, 0.75]	0.75 [0.25, 1.00]	0.19 [0.17, 0.21]	0.92 [0.83, 1.00]
lr1e-05_wd0.0001_h400_d1	0.75 [0.65, 0.96]	0.50 [0.31, 0.87]	0.29 [0.24, 0.33]	0.57 [0.48, 0.63]	0.10 [-0.04, 0.22]	0.50 [0.26, 0.70]	0.63 [0.50, 1.00]	0.19 [0.15, 0.25]	0.89 [0.82, 1.00]
lr1e-05_wd0.0001_h512_d1	0.75 [0.65, 0.86]	0.53 [0.33, 0.66]	0.24 [0.00, 0.35]	0.55 [0.40, 0.71]	0.06 [-0.20, 0.30]	0.42 [0.00, 0.80]	0.68 [0.00, 1.00]	0.15 [0.00, 0.21]	0.72 [0.00, 1.00]
lr1e-05_wd0.0001_h256_d2	0.72 [0.64, 0.86]	0.46 [0.29, 0.60]	0.20 [0.00, 0.30]	0.47 [0.40, 0.53]	-0.08 [-0.27, 0.09]	0.18 [0.00, 0.80]	0.75 [0.00, 1.00]	0.12 [0.00, 0.17]	0.46 [0.00, 1.00]
lr1e-05_wd0.0001_h400_d2	0.73 [0.67, 0.80]	0.48 [0.34, 0.57]	0.22 [0.00, 0.30]	0.53 [0.50, 0.63]	0.04 [0.00, 0.22]	0.25 [0.00, 1.00]	0.80 [0.00, 1.00]	0.13 [0.00, 0.18]	0.37 [0.00, 1.00]
lr1e-05_wd0.0001_h512_d2	0.74 [0.71, 0.78]	0.38 [0.34, 0.50]	0.22 [0.00, 0.44]	0.57 [0.43, 0.68]	0.10 [-0.17, 0.32]	0.68 [0.35, 1.00]	0.45 [0.00, 1.00]	0.16 [0.00, 0.40]	0.89 [0.81, 1.00]
lr1e-05_wd0.001_h256_d1	0.76 [0.69, 0.86]	0.38 [0.30, 0.59]	0.21 [0.00, 0.40]	0.56 [0.40, 0.70]	0.08 [-0.20, 0.30]	0.59 [0.20, 0.85]	0.53 [0.00, 1.00]	0.14 [0.00, 0.29]	0.91 [0.80, 1.00]
lr1e-05_wd0.001_h400_d1	0.72 [0.63, 0.88]	0.48 [0.24, 0.77]	0.29 [0.27, 0.31]	0.58 [0.55, 0.63]	0.12 [0.08, 0.22]	0.42 [0.26, 0.65]	0.73 [0.50, 1.00]	0.19 [0.17, 0.22]	0.90 [0.87, 1.00]
lr1e-05_wd0.001_h512_d1	0.74 [0.67, 0.79]	0.55 [0.28, 0.71]	0.30 [0.18, 0.46]	0.59 [0.48, 0.73]	0.14 [-0.04, 0.35]	0.50 [0.32, 0.70]	0.68 [0.25, 1.00]	0.20 [0.13, 0.33]	0.90 [0.82, 1.00]
lr1e-05_wd0.001_h256_d2	0.71 [0.63, 0.77]	0.50 [0.32, 0.71]	0.21 [0.00, 0.32]	0.50 [0.45, 0.58]	0.00 [-0.13, 0.17]	0.25 [0.00, 0.90]	0.75 [0.00, 1.00]	0.12 [0.00, 0.19]	0.52 [0.00, 1.00]
lr1e-05_wd0.001_h400_d2	0.81 [0.73, 0.86]	0.51 [0.32, 0.59]	0.30 [0.00, 0.44]	0.62 [0.43, 0.73]	0.18 [-0.17, 0.35]	0.56 [0.21, 0.85]	0.68 [0.00, 1.00]	0.20 [0.00, 0.33]	0.93 [0.81, 1.00]
lr1e-05_wd0.001_h512_d2	0.81 [0.68, 0.89]	0.54 [0.38, 0.66]	0.32 [0.27, 0.40]	0.60 [0.50, 0.68]	0.16 [0.00, 0.27]	0.35 [0.00, 0.80]	0.85 [0.50, 1.00]	0.21 [0.16, 0.33]	0.76 [0.00, 1.00]
lr3e-05_wd0.0_h256_d1	0.77 [0.68, 0.95]	0.61 [0.45, 0.76]	0.31 [0.17, 0.43]	0.56 [0.36, 0.70]	0.08 [-0.34, 0.30]	0.34 [0.05, 0.65]	0.78 [0.67, 1.00]	0.20 [0.10, 0.30]	0.83 [0.50, 1.00]
lr3e-05_wd0.0_h400_d1	0.82 [0.68, 0.96]	0.59 [0.37, 0.87]	0.33 [0.22, 0.44]	0.62 [0.54, 0.75]	0.20 [0.05, 0.38]	0.43 [0.15, 0.74]	0.82 [0.33, 1.00]	0.21 [0.17, 0.29]	0.95 [0.88, 1.00]
lr3e-05_wd0.0_h512_d1	0.79 [0.70, 0.93]	0.47 [0.32, 0.70]	0.35 [0.00, 0.50]	0.68 [0.47, 0.80]	0.28 [-0.09, 0.45]	0.75 [0.53, 0.95]	0.60 [0.00, 1.00]	0.29 [0.00, 0.50]	0.93 [0.86, 1.00]
lr3e-05_wd0.0_h256_d2	0.73 [0.69, 0.82]	0.49 [0.28, 0.72]	0.31 [0.00, 0.43]	0.63 [0.40, 0.79]	0.18 [-0.20, 0.40]	0.63 [0.55, 0.80]	0.63 [0.00, 1.00]	0.20 [0.00, 0.30]	0.91 [0.80, 1.00]
lr3e-05_wd0.0_h400_d2	0.74 [0.68, 0.86]	0.51 [0.28, 0.63]	0.38 [0.24, 0.55]	0.66 [0.50, 0.82]	0.24 [0.00, 0.45]	0.47 [0.00, 0.80]	0.85 [0.75, 1.00]	0.26 [0.14, 0.43]	0.75 [0.00, 1.00]
lr3e-05_wd0.0_h512_d2	0.76 [0.65, 0.91]	0.51 [0.32, 0.60]	0.39 [0.20, 0.55]	0.68 [0.44, 0.87]	0.27 [-0.10, 0.53]	0.52 [0.21, 0.75]	0.83 [0.50, 1.00]	0.27 [0.12, 0.38]	0.94 [0.80, 1.00]
lr3e-05_wd0.0001_h256_d1	0.76 [0.64, 0.91]	0.55 [0.26, 0.79]	0.33 [0.00, 0.50]	0.64 [0.45, 0.71]	0.25 [-0.13, 0.55]	0.70 [0.42, 1.00]	0.57 [0.00, 1.00]	0.35 [0.00, 1.00]	0.91 [0.82, 1.00]
lr3e-05_wd0.0001_h400_d1	0.75 [0.67, 0.86]	0.45 [0.28, 0.71]	0.28 [0.00, 0.50]	0.59 [0.40, 0.84]	0.13 [-0.20, 0.48]	0.55 [0.15, 0.80]	0.63 [0.00, 1.00]	0.19 [0.00, 0.33]	0.91 [0.80, 1.00]
lr3e-05_wd0.0001_h512_d1	0.76 [0.70, 0.81]	0.44 [0.33, 0.62]	0.34 [0.00, 0.50]	0.65 [0.40, 0.84]	0.23 [-0.20, 0.48]	0.73 [0.55, 0.95]	0.57 [0.00, 1.00]	0.28 [0.00, 0.50]	0.91 [0.80, 1.00]
lr3e-05_wd0.0001_h256_d2	0.80 [0.73, 0.95]	0.53 [0.35, 0.64]	0.40 [0.25, 0.67]	0.68 [0.53, 0.92]	0.28 [0.04, 0.65]	0.66 [0.30, 0.85]	0.70 [0.25, 1.00]	0.30 [0.18, 0.50]	0.92 [0.85, 1.00]
lr3e-05_wd0.0001_h400_d2	0.74 [0.67, 0.84]	0.52 [0.29, 0.71]	0.32 [0.00, 0.50]	0.63 [0.43, 0.79]	0.18 [-0.17, 0.40]	0.62 [0.20, 0.85]	0.63 [0.00, 1.00]	0.22 [0.00, 0.38]	0.90 [0.80, 1.00]
lr3e-05_wd0.0001_h512_d2	0.83 [0.75, 0.93]	0.56 [0.33, 0.81]	0.37 [0.27, 0.55]	0.65 [0.50, 0.78]	0.23 [0.00, 0.45]	0.40 [0.00, 0.80]	0.90 [0.75, 1.00]	0.25 [0.16, 0.43]	0.77 [0.00, 1.00]
lr3e-05_wd0.001_h256_d1	0.76 [0.69, 0.91]	0.50 [0.28, 0.72]	0.29 [0.18, 0.40]	0.57 [0.39, 0.71]	0.09 [-0.23, 0.30]	0.46 [0.11, 0.70]	0.68 [0.25, 1.00]	0.19 [0.11, 0.27]	0.86 [0.67, 1.00]
lr3e-05_wd0.001_h400_d1	0.73 [0.66, 0.91]	0.50 [0.27, 0.63]	0.29 [0.00, 0.55]	0.59 [0.38, 0.87]	0.12 [-0.23, 0.53]	0.59 [0.37, 0.75]	0.58 [0.00, 1.00]	0.19 [0.00, 0.38]	0.89 [0.79, 1.00]
lr3e-05_wd0.001_h512_d1	0.78 [0.70, 0.93]	0.58 [0.31, 0.81]	0.28 [0.22, 0.36]	0.55 [0.50, 0.63]	0.09 [0.00, 0.20]	0.48 [0.16, 0.80]	0.63 [0.25, 1.00]	0.19 [0.15, 0.29]	0.89 [0.83, 1.00]
lr3e-05_wd0.001_h256_d2	0.74 [0.66, 0.86]	0.48 [0.29, 0.66]	0.35 [0.29, 0.43]	0.64 [0.53, 0.74]	0.22 [0.09, 0.33]	0.38 [0.05, 0.65]	0.90 [0.75, 1.00]	0.22 [0.17, 0.30]	0.97 [0.91, 1.00]
lr3e-05_wd0.001_h400_d2	0.78 [0.73, 0.93]	0.57 [0.31, 0.72]	0.36 [0.22, 0.46]	0.63 [0.38, 0.73]	0.13 [-0.47, 0.35]	0.47 [0.00, 0.70]	0.78 [0.67, 1.00]	0.24 [0.13, 0.33]	0.76 [0.00, 1.00]
lr3e-05_wd0.001_h512_d2	0.77 [0.67, 0.84]	0.51 [0.39, 0.71]	0.40 [0.33, 0.50]	0.70 [0.63, 0.84]	0.31 [0.20, 0.48]	0.57 [0.25, 0.75]	0.83 [0.50, 1.00]	0.27 [0.21, 0.33]	0.96 [0.88, 1.00]
lr0.0001_wd0.005_h256_d1	0.77 [0.60, 0.93]	0.51 [0.26, 0.71]	0.34 [0.22, 0.44]	0.63 [0.53, 0.74]	0.21 [0.05, 0.33]	0.58 [0.25, 0.85]	0.68 [0.25, 1.00]	0.24 [0.18, 0.40]	0.93 [0.84, 1.00]
lr0.0001_wd0.005_h400_d1	0.79 [0.71, 0.89]	0.55 [0.35, 0.64]	0.41 [0.31, 0.47]	0.70 [0.62, 0.82]	0.30 [0.17, 0.44]	0.62 [0.50, 0.85]	0.78 [0.50, 1.00]	0.29 [0.20, 0.40]	0.94 [0.89, 1.00]
lr0.0001_wd0.005_h512_d1	0.78 [0.67, 0.88]	0.54 [0.31, 0.71]	0.36 [0.31, 0.44]	0.64 [0.58, 0.68]	0.23 [0.12, 0.32]	0.60 [0.37, 0.85]	0.68 [0.50, 1.00]	0.27 [0.20, 0.40]	0.92 [0.89, 1.00]
lr0.0001_wd0.005_h256_d2	0.77 [0.67, 0.88]	0.48 [0.27, 0.65]	0.37 [0.24, 0.46]	0.65 [0.50, 0.73]	0.23 [0.00, 0.35]	0.49 [0.00, 0.85]	0.80 [0.50, 1.00]	0.27 [0.14, 0.40]	0.75 [0.00, 1.00]
lr0.0001_wd0.005_h400_d2	0.77 [0.67, 0.88]	0.48 [0.31, 0.71]	0.41 [0.29, 0.60]	0.68 [0.55, 0.89]	0.29 [0.13, 0.58]	0.57 [0.10, 0.85]	0.78 [0.50, 1.00]	0.29 [0.18, 0.43]	0.95 [0.89, 1.00]
lr0.0001_wd0.005_h512_d2	0.79 [0.67, 0.91]	0.55 [0.33, 0.71]	0.46 [0.40, 0.57]	0.74 [0.68, 0.79]	0.38 [0.26, 0.50]	0.69 [0.55, 0.89]	0.78 [0.50, 1.00]	0.35 [0.27, 0.50]	0.95 [0.89, 1.00]
lr0.0001_wd0.01_h256_d1	0.79 [0.67, 0.93]	0.61 [0.34, 0.81]	0.33 [0.22, 0.40]	0.63 [0.53, 0.71]	0.19 [0.05, 0.30]	0.62 [0.42, 0.80]	0.63 [0.25, 1.00]	0.24 [0.20, 0.33]	0.91 [0.84, 1.00]
lr0.0001_wd0.01_h400_d1	0.78 [0.69, 0.89]	0.51 [0.34, 0.72]	0.37 [0.25, 0.44]	0.65 [0.55, 0.75]	0.25 [0.10, 0.38]	0.69 [0.47, 0.85]	0.62 [0.25, 1.00]	0.29 [0.17, 0.40]	0.92 [0.85, 1.00]

Supplementary Table 11: Grid search results on the classification task on GDHCM prospective dataset (choosing 50% as the threshold for clinical variables selection). Reported metrics are AUC, AUPRC, F1, Balanced Accuracy, MCC, Specificity, Sensitivity, PPV, and NPV.

Config	AUC	AUPRC	F1	Balanced_Accuracy	MCC	Specificity	Sensitivity	PPV	NPV
lr0.0001_wd0.01_h512_d1	0.81 [0.67, 0.91]	0.58 [0.39, 0.71]	0.41 [0.29, 0.57]	0.70 [0.60, 0.85]	0.31 [0.13, 0.53]	0.62 [0.45, 0.85]	0.78 [0.50, 1.00]	0.29 [0.18, 0.40]	0.94 [0.89, 1.00]
lr0.0001_wd0.01_h256_d2	0.80 [0.70, 0.91]	0.55 [0.37, 0.70]	0.29 [0.24, 0.38]	0.57 [0.50, 0.68]	0.13 [0.00, 0.29]	0.34 [0.00, 0.90]	0.80 [0.25, 1.00]	0.21 [0.14, 0.33]	0.74 [0.00, 1.00]
lr0.0001_wd0.01_h400_d2	0.75 [0.66, 0.88]	0.50 [0.27, 0.60]	0.33 [0.22, 0.44]	0.58 [0.38, 0.73]	0.07 [-0.47, 0.35]	0.54 [0.00, 0.80]	0.62 [0.50, 0.75]	0.25 [0.13, 0.33]	0.72 [0.00, 0.94]
lr0.0001_wd0.01_h512_d2	0.78 [0.67, 0.93]	0.57 [0.31, 0.71]	0.30 [0.00, 0.55]	0.62 [0.48, 0.87]	0.19 [-0.09, 0.53]	0.71 [0.37, 0.95]	0.53 [0.00, 1.00]	0.25 [0.00, 0.50]	0.90 [0.83, 1.00]
lr0.0003_wd0.005_h256_d1	0.79 [0.72, 0.89]	0.55 [0.32, 0.72]	0.39 [0.27, 0.50]	0.67 [0.57, 0.76]	0.26 [0.10, 0.40]	0.60 [0.40, 0.90]	0.73 [0.50, 1.00]	0.29 [0.17, 0.50]	0.92 [0.89, 1.00]
lr0.0003_wd0.005_h400_d1	0.77 [0.67, 0.91]	0.54 [0.26, 0.71]	0.37 [0.27, 0.44]	0.65 [0.50, 0.75]	0.23 [0.00, 0.38]	0.51 [0.25, 0.85]	0.78 [0.50, 1.00]	0.26 [0.17, 0.40]	0.93 [0.83, 1.00]
lr0.0003_wd0.005_h512_d1	0.77 [0.67, 0.88]	0.52 [0.29, 0.71]	0.27 [0.00, 0.44]	0.56 [0.36, 0.74]	0.07 [-0.34, 0.33]	0.55 [0.05, 0.85]	0.58 [0.00, 1.00]	0.20 [0.00, 0.40]	0.82 [0.50, 1.00]
lr0.0003_wd0.005_h256_d2	0.76 [0.65, 0.91]	0.44 [0.25, 0.56]	0.21 [0.00, 0.40]	0.55 [0.45, 0.63]	0.13 [-0.13, 0.47]	0.79 [0.20, 1.00]	0.32 [0.00, 1.00]	0.31 [0.00, 1.00]	0.89 [0.82, 1.00]
lr0.0003_wd0.005_h400_d2	0.80 [0.72, 0.89]	0.59 [0.35, 0.72]	0.41 [0.29, 0.57]	0.66 [0.59, 0.73]	0.30 [0.16, 0.51]	0.78 [0.50, 0.95]	0.55 [0.33, 0.75]	0.38 [0.23, 0.67]	0.91 [0.89, 0.93]
lr0.0003_wd0.005_h512_d2	0.82 [0.73, 1.00]	0.58 [0.34, 1.00]	0.37 [0.25, 0.57]	0.66 [0.55, 0.78]	0.27 [0.10, 0.50]	0.68 [0.26, 0.89]	0.63 [0.25, 1.00]	0.30 [0.18, 0.50]	0.93 [0.85, 1.00]
lr0.0003_wd0.01_h256_d1	0.79 [0.67, 0.96]	0.53 [0.28, 0.87]	0.44 [0.38, 0.50]	0.71 [0.65, 0.80]	0.38 [0.26, 0.55]	0.74 [0.47, 1.00]	0.67 [0.33, 1.00]	0.46 [0.23, 1.00]	0.94 [0.89, 1.00]
lr0.0003_wd0.01_h400_d1	0.78 [0.67, 0.96]	0.63 [0.34, 0.87]	0.39 [0.30, 0.44]	0.67 [0.63, 0.73]	0.28 [0.19, 0.35]	0.61 [0.26, 0.85]	0.73 [0.50, 1.00]	0.29 [0.18, 0.40]	0.93 [0.89, 1.00]
lr0.0003_wd0.01_h512_d1	0.78 [0.67, 0.89]	0.47 [0.35, 0.58]	0.38 [0.21, 0.50]	0.65 [0.46, 0.76]	0.25 [-0.05, 0.40]	0.56 [0.25, 0.90]	0.73 [0.50, 1.00]	0.30 [0.13, 0.50]	0.93 [0.83, 1.00]
lr0.0003_wd0.01_h256_d2	0.78 [0.68, 0.88]	0.52 [0.33, 0.71]	0.41 [0.31, 0.62]	0.67 [0.58, 0.88]	0.29 [0.12, 0.58]	0.70 [0.40, 0.95]	0.65 [0.33, 1.00]	0.34 [0.20, 0.50]	0.92 [0.89, 1.00]
lr0.0003_wd0.01_h400_d2	0.76 [0.64, 0.89]	0.48 [0.25, 0.71]	0.38 [0.25, 0.50]	0.67 [0.54, 0.84]	0.25 [0.06, 0.48]	0.60 [0.42, 0.85]	0.73 [0.50, 1.00]	0.27 [0.15, 0.40]	0.92 [0.89, 1.00]
lr0.0003_wd0.01_h512_d2	0.76 [0.67, 0.93]	0.54 [0.28, 0.71]	0.39 [0.27, 0.67]	0.66 [0.55, 0.92]	0.26 [0.10, 0.65]	0.58 [0.10, 0.90]	0.73 [0.25, 1.00]	0.29 [0.17, 0.50]	0.94 [0.86, 1.00]
lr0.001_wd0.005_h256_d1	0.76 [0.67, 0.91]	0.53 [0.32, 0.71]	0.37 [0.25, 0.60]	0.66 [0.55, 0.89]	0.26 [0.10, 0.58]	0.75 [0.45, 0.90]	0.58 [0.25, 1.00]	0.30 [0.21, 0.43]	0.91 [0.85, 1.00]
lr0.001_wd0.005_h400_d1	0.78 [0.69, 0.93]	0.50 [0.31, 0.70]	0.35 [0.22, 0.46]	0.64 [0.53, 0.76]	0.21 [0.05, 0.36]	0.65 [0.42, 0.80]	0.63 [0.25, 1.00]	0.25 [0.15, 0.33]	0.91 [0.84, 1.00]
lr0.001_wd0.005_h512_d1	0.78 [0.65, 0.88]	0.46 [0.25, 0.63]	0.37 [0.00, 0.50]	0.69 [0.48, 0.76]	0.29 [-0.09, 0.42]	0.74 [0.50, 0.95]	0.63 [0.00, 1.00]	0.29 [0.00, 0.50]	0.93 [0.83, 1.00]
lr0.001_wd0.005_h256_d2	0.79 [0.69, 0.95]	0.55 [0.31, 0.73]	0.43 [0.31, 0.67]	0.70 [0.62, 0.92]	0.31 [0.17, 0.65]	0.62 [0.50, 0.84]	0.78 [0.67, 1.00]	0.30 [0.20, 0.50]	0.93 [0.91, 1.00]
lr0.001_wd0.005_h400_d2	0.82 [0.76, 0.88]	0.57 [0.39, 0.73]	0.47 [0.33, 0.67]	0.70 [0.61, 0.75]	0.39 [0.23, 0.67]	0.76 [0.35, 1.00]	0.65 [0.33, 1.00]	0.46 [0.24, 1.00]	0.94 [0.89, 1.00]
lr0.001_wd0.005_h512_d2	0.81 [0.75, 0.95]	0.54 [0.34, 0.76]	0.50 [0.26, 0.60]	0.76 [0.55, 0.89]	0.42 [0.13, 0.58]	0.63 [0.11, 0.85]	0.90 [0.75, 1.00]	0.37 [0.15, 0.50]	0.98 [0.94, 1.00]
lr0.001_wd0.01_h256_d1	0.77 [0.73, 0.84]	0.51 [0.32, 0.72]	0.35 [0.27, 0.44]	0.62 [0.57, 0.68]	0.20 [0.10, 0.32]	0.69 [0.47, 0.85]	0.55 [0.33, 0.75]	0.27 [0.17, 0.40]	0.90 [0.88, 0.92]
lr0.001_wd0.01_h400_d1	0.76 [0.67, 0.86]	0.49 [0.35, 0.71]	0.29 [0.00, 0.40]	0.63 [0.48, 0.70]	0.21 [-0.09, 0.32]	0.67 [0.32, 0.95]	0.58 [0.00, 1.00]	0.24 [0.00, 0.50]	0.92 [0.83, 1.00]
lr0.001_wd0.01_h512_d1	0.78 [0.68, 0.95]	0.48 [0.29, 0.76]	0.42 [0.29, 0.57]	0.69 [0.59, 0.78]	0.32 [0.16, 0.50]	0.64 [0.35, 0.89]	0.75 [0.33, 1.00]	0.31 [0.24, 0.50]	0.95 [0.89, 1.00]
lr0.001_wd0.01_h256_d2	0.81 [0.66, 0.91]	0.59 [0.28, 0.78]	0.37 [0.25, 0.50]	0.62 [0.53, 0.70]	0.25 [0.09, 0.40]	0.44 [0.05, 0.90]	0.80 [0.50, 1.00]	0.30 [0.14, 0.50]	0.96 [0.90, 1.00]
lr0.001_wd0.01_h400_d2	0.80 [0.68, 0.91]	0.61 [0.37, 0.80]	0.46 [0.29, 0.60]	0.72 [0.58, 0.89]	0.36 [0.17, 0.58]	0.77 [0.50, 0.90]	0.68 [0.25, 1.00]	0.36 [0.23, 0.43]	0.93 [0.86, 1.00]
lr0.001_wd0.01_h512_d2	0.77 [0.69, 0.95]	0.54 [0.38, 0.76]	0.43 [0.31, 0.67]	0.70 [0.62, 0.92]	0.31 [0.17, 0.65]	0.67 [0.50, 0.84]	0.73 [0.50, 1.00]	0.31 [0.20, 0.50]	0.93 [0.88, 1.00]
lr0.0001_wd0.0_h1024_d1	0.75 [0.67, 0.88]	0.52 [0.28, 0.71]	0.35 [0.27, 0.50]	0.64 [0.50, 0.80]	0.22 [0.00, 0.45]	0.54 [0.25, 0.95]	0.73 [0.25, 1.00]	0.28 [0.17, 0.50]	0.92 [0.83, 1.00]
lr0.0001_wd0.0_h2048_d1	0.83 [0.73, 0.95]	0.58 [0.40, 0.72]	0.46 [0.38, 0.57]	0.73 [0.68, 0.85]	0.38 [0.30, 0.53]	0.73 [0.47, 0.90]	0.73 [0.50, 1.00]	0.36 [0.23, 0.50]	0.95 [0.89, 1.00]
lr0.0001_wd0.0_h1024_d2	0.78 [0.70, 0.93]	0.53 [0.30, 0.72]	0.32 [0.24, 0.43]	0.61 [0.50, 0.79]	0.17 [0.00, 0.40]	0.42 [0.00, 0.85]	0.80 [0.25, 1.00]	0.22 [0.14, 0.27]	0.75 [0.00, 1.00]
lr0.0001_wd0.0_h2048_d2	0.80 [0.65, 0.93]	0.56 [0.25, 0.74]	0.44 [0.29, 0.57]	0.68 [0.50, 0.84]	0.33 [0.00, 0.51]	0.69 [0.00, 0.95]	0.68 [0.25, 1.00]	0.40 [0.17, 0.67]	0.74 [0.00, 1.00]
lr0.0001_wd0.0001_h1024_d1	0.78 [0.64, 0.89]	0.55 [0.24, 0.73]	0.40 [0.25, 0.60]	0.67 [0.54, 0.80]	0.29 [0.06, 0.52]	0.59 [0.25, 0.89]	0.75 [0.33, 1.00]	0.30 [0.15, 0.50]	0.95 [0.89, 1.00]
lr0.0001_wd0.0001_h2048_d1	0.77 [0.64, 1.00]	0.57 [0.25, 1.00]	0.31 [0.00, 0.44]	0.62 [0.40, 0.70]	0.18 [-0.20, 0.32]	0.70 [0.32, 0.85]	0.53 [0.00, 1.00]	0.24 [0.00, 0.40]	0.90 [0.80, 1.00]
lr0.0001_wd0.0001_h1024_d2	0.80 [0.73, 0.91]	0.56 [0.33, 0.73]	0.32 [0.00, 0.50]	0.61 [0.43, 0.70]	0.20 [-0.17, 0.40]	0.78 [0.55, 0.95]	0.45 [0.00, 0.75]	0.29 [0.00, 0.50]	0.89 [0.81, 0.92]
lr0.0001_wd0.0001_h2048_d2	0.78 [0.64, 0.95]	0.54 [0.24, 0.72]	0.37 [0.00, 0.67]	0.63 [0.45, 0.81]	0.24 [-0.13, 0.61]	0.75 [0.32, 0.95]	0.52 [0.00, 0.75]	0.33 [0.00, 0.67]	0.89 [0.82, 0.95]
lr0.0001_wd0.001_h1024_d1	0.80 [0.69, 0.93]	0.52 [0.32, 0.73]	0.39 [0.22, 0.57]	0.66 [0.53, 0.78]	0.27 [0.05, 0.50]	0.80 [0.63, 0.89]	0.52 [0.25, 0.67]	0.33 [0.20, 0.50]	0.90 [0.84, 0.94]
lr0.0001_wd0.001_h2048_d1	0.78 [0.67, 0.89]	0.57 [0.32, 0.71]	0.45 [0.36, 0.55]	0.73 [0.68, 0.78]	0.36 [0.25, 0.45]	0.72 [0.53, 0.85]	0.73 [0.50, 1.00]	0.34 [0.25, 0.43]	0.94 [0.89, 1.00]
lr0.0001_wd0.001_h1024_d2	0.77 [0.67, 0.91]	0.50 [0.29, 0.71]	0.45 [0.29, 0.75]	0.71 [0.53, 0.95]	0.35 [0.04, 0.73]	0.69 [0.30, 0.90]	0.73 [0.25, 1.00]	0.36 [0.18, 0.60]	0.93 [0.86, 1.00]
lr0.0001_wd0.001_h2048_d2	0.76 [0.65, 0.91]	0.52 [0.25, 0.71]	0.42 [0.33, 0.50]	0.68 [0.60, 0.80]	0.37 [0.27, 0.47]	0.86 [0.60, 1.00]	0.50 [0.25, 1.00]	0.53 [0.33, 1.00]	0.91 [0.86, 1.00]
lr0.0003_wd0.0_h1024_d1	0.79 [0.69, 0.89]	0.54 [0.31, 0.72]	0.37 [0.30, 0.44]	0.63 [0.53, 0.68]	0.23 [0.09, 0.32]	0.66 [0.05, 0.89]	0.60 [0.33, 1.00]	0.31 [0.17, 0.40]	0.92 [0.89, 1.00]
lr0.0003_wd0.0_h2048_d1	0.77 [0.68, 0.84]	0.51 [0.32, 0.65]	0.41 [0.25, 0.67]	0.65 [0.55, 0.81]	0.29 [0.10, 0.61]	0.85 [0.70, 0.95]	0.45 [0.25, 0.67]	0.38 [0.25, 0.67]	0.89 [0.85, 0.95]
lr0.0003_wd0.0_h1024_d2	0.80 [0.68, 0.89]	0.60 [0.26, 0.83]	0.48 [0.25, 0.75]	0.72 [0.53, 0.85]	0.39 [0.09, 0.70]	0.64 [0.05, 0.95]	0.80 [0.50, 1.00]	0.40 [0.14, 0.75]	0.95 [0.90, 1.00]
lr0.0003_wd0.0_h2048_d2	0.83 [0.74, 0.95]	0.63 [0.34, 0.76]	0.53 [0.33, 0.73]	0.77 [0.60, 0.93]	0.47 [0.26, 0.70]	0.86 [0.74, 0.95]	0.68 [0.25, 1.00]	0.49 [0.33, 0.67]	0.94 [0.86, 1.00]
lr0.0003_wd0.0001_h1024_d1	0.76 [0.63, 0.89]	0.56 [0.25, 0.73]	0.37 [0.29, 0.46]	0.65 [0.58, 0.73]	0.26 [0.17, 0.35]	0.71 [0.30, 0.90]	0.60 [0.25, 1.00]	0.30 [0.22, 0.33]	0.92 [0.86, 1.00]
lr0.0003_wd0.0001_h2048_d1	0.80 [0.71, 0.96]	0.58 [0.30, 0.81]	0.41 [0.29, 0.55]	0.68 [0.60, 0.87]	0.28 [0.13, 0.53]	0.68 [0.50, 0.85]	0.68 [0.50, 1.00]	0.30 [0.18, 0.40]	0.92 [0.89, 1.00]
lr0.0003_wd0.0001_h1024_d2	0.84 [0.73, 0.94]	0.59 [0.30, 0.79]	0.47 [0.35, 0.60]	0.73 [0.63, 0.80]	0.36 [0.19, 0.52]	0.74 [0.50, 0.85]	0.72 [0.67, 0.75]	0.36 [0.23, 0.50]	0.93 [0.91, 0.94]
lr0.0003_wd0.0001_h2048_d2	0.81 [0.70, 0.96]	0.58 [0.32, 0.87]	0.50 [0.33, 0.60]	0.76 [0.68, 0.84]	0.42 [0.27, 0.52]	0.72 [0.37, 0.90]	0.80 [0.50, 1.00]	0.39 [0.20, 0.50]	0.96 [0.90, 1.00]
lr0.0003_wd0.001_h1024_d1	0.78 [0.63, 0.93]	0.58 [0.28, 0.72]	0.34 [0.20, 0.50]	0.60 [0.44, 0.70]	0.19 [-0.10, 0.40]	0.63 [0.21, 0.90]	0.58 [0.25, 1.00]	0.28 [0.12, 0.50]	0.89 [0.80, 1.00]
lr0.0003_wd0.001_h2048_d1	0.77 [0.63, 0.86]	0.55 [0.25, 0.75]	0.40 [0.29, 0.57]	0.66 [0.60, 0.73]	0.29 [0.13, 0.51]	0.59 [0.26, 0.95]	0.73 [0.50, 1.00]	0.33 [0.18, 0.67]	0.94 [0.89, 1.00]
lr0.0003_wd0.001_h1024_d2	0.83 [0.74, 0.93]	0.58 [0.32, 0.77]	0.32 [0.00, 0.46]	0.65 [0.47, 0.82]	0.21 [-0.09, 0.44]	0.65 [0.40, 0.95]	0.65 [0.00, 1.00]	0.21 [0.00, 0.30]	0.93 [0.86, 1.00]
lr0.0003_wd0.001_h2048_d2	0.80 [0.65, 0.95]	0.54 [0.30, 0.73]	0.36 [0.00, 0.62]	0.68 [0.45, 0.88]	0.27 [-0.13, 0.58]	0.63 [0.30, 0.90]	0.73 [0.00, 1.00]	0.24 [0.00, 0.44]	0.95 [0.82, 1.00]
lr0.001_wd0.0_h1024_d1	0.75 [0.64, 0.88]	0.51 [0.26, 0.63]	0.43 [0.25, 0.62]	0.69 [0.54, 0.88]	0.30 [0.06, 0.58]	0.61 [0.42, 0.89]	0.77 [0.67, 1.00]	0.31 [0.15, 0.50]	0.93 [0.89, 1.00]
lr0.001_wd0.0_h2048_d1	0.78 [0.70, 0.91]	0.52 [0.31, 0.73]	0.30 [0.00, 0.44]	0.62 [0.45, 0.68]	0.19 [-0.13, 0.32]	0.61 [0.30, 0.90]	0.63 [0.00, 1.00]	0.21 [0.00, 0.40]	0.93 [0.82, 1.00]
lr0.001_wd0.0_h1024_d2	0.81 [0.72, 0.89]	0.55 [0.45, 0.72]	0.47 [0.36, 0.53]	0.74 [0.68, 0.83]	0.37 [0.25, 0.49]	0.76 [0.65, 0.85]	0.72 [0.50, 1.00]	0.36 [0.25, 0.40]	0.94 [0.89, 1.00]
lr0.001_wd0.0_h2048_d2	0.79 [0.69, 0.95]	0.63 [0.37, 0.83]	0.40 [0.29, 0.60]	0.66 [0.50, 0.89]	0.29 [0.00, 0.58]	0.70 [0.00, 0.95]	0.63 [0.25, 1.00]	0.37 [0.17, 0.50]	0.73 [0.00, 1.00]
lr0.001_wd0.0001_h1024_d1	0.80 [0.74, 0.88]	0.53 [0.31, 0.73]	0.32 [0.00, 0.53]	0.64 [0.48, 0.83]	0.22 [-0.09, 0.49]	0.69 [0.20, 0.95]	0.60 [0.00, 1.00]	0.24 [0.00, 0.36]	0.93 [0.83, 1.00]
lr0.001_wd0.0001_h2048_d1	0.76 [0.65, 0.86]	0.52 [0.27, 0.73]	0.36 [0.33, 0.44]	0.64 [0.60, 0.68]	0.26 [0.22, 0.32]	0.72 [0.37, 0.95]	0.57 [0.25, 1.00]	0.34 [0.20, 0.50]	0.91 [0.86, 1.00]
lr0.001_wd0.0001_h1024_d2	0.84 [0.73, 1.00]	0.60 [0.22, 0.90]	0.52 [0.29, 0.67]	0.79 [0.61, 0.92]	0.46 [0.19, 0.65]	0.62 [0.21, 0.85]	0.95 [0.75, 1.00]	0.38 [0.17, 0.50]	0.99 [0.94, 1.00]

Supplementary Table 12: Grid search results on the classification task on GDHCM prospective dataset (choosing 50% as the threshold for clinical variables selection). Reported metrics are AUC, AUPRC, F1, Balanced Accuracy, MCC, Specificity, Sensitivity, PPV, and NPV.

Config	AUC	AUPRC	F1	Balanced_Accuracy	MCC	Specificity	Sensitivity	PPV	NPV
lr0.001_wd0.0001_h2048_d2	0.84 [0.73, 0.93]	0.53 [0.31, 0.81]	0.39 [0.00, 0.57]	0.70 [0.50, 0.79]	0.32 [0.00, 0.50]	0.77 [0.58, 1.00]	0.63 [0.00, 1.00]	0.29 [0.00, 0.50]	0.93 [0.83, 1.00]
lr0.001_wd0.001_h1024_d1	0.76 [0.61, 0.84]	0.52 [0.23, 0.68]	0.41 [0.30, 0.50]	0.68 [0.63, 0.75]	0.30 [0.22, 0.42]	0.58 [0.26, 0.85]	0.78 [0.50, 1.00]	0.30 [0.18, 0.40]	0.95 [0.89, 1.00]
lr0.001_wd0.001_h2048_d1	0.76 [0.65, 0.93]	0.57 [0.26, 0.72]	0.36 [0.00, 0.57]	0.67 [0.50, 0.78]	0.28 [0.00, 0.50]	0.73 [0.35, 1.00]	0.62 [0.00, 1.00]	0.27 [0.00, 0.50]	0.93 [0.83, 1.00]
lr0.001_wd0.001_h1024_d2	0.80 [0.71, 0.92]	0.66 [0.32, 0.89]	0.53 [0.31, 0.86]	0.75 [0.62, 0.92]	0.45 [0.17, 0.85]	0.72 [0.35, 1.00]	0.78 [0.50, 1.00]	0.47 [0.20, 1.00]	0.95 [0.89, 1.00]
lr0.001_wd0.001_h2048_d2	0.83 [0.74, 0.93]	0.56 [0.34, 0.79]	0.52 [0.33, 0.67]	0.76 [0.60, 0.83]	0.44 [0.15, 0.61]	0.70 [0.45, 0.95]	0.83 [0.67, 1.00]	0.42 [0.21, 0.67]	0.96 [0.90, 1.00]
lr0.0001_wd0.0_h256_d3	0.80 [0.70, 0.95]	0.53 [0.29, 0.64]	0.32 [0.00, 0.50]	0.63 [0.43, 0.79]	0.19 [-0.17, 0.40]	0.61 [0.00, 0.85]	0.65 [0.00, 1.00]	0.24 [0.00, 0.40]	0.73 [0.00, 1.00]
lr0.0001_wd0.0_h400_d3	0.77 [0.64, 0.90]	0.57 [0.25, 0.73]	0.47 [0.33, 0.67]	0.69 [0.60, 0.81]	0.35 [0.15, 0.61]	0.72 [0.45, 0.95]	0.67 [0.50, 0.75]	0.40 [0.21, 0.67]	0.92 [0.90, 0.95]
lr0.0001_wd0.0_h512_d3	0.76 [0.69, 0.89]	0.55 [0.28, 0.72]	0.29 [0.00, 0.50]	0.60 [0.45, 0.75]	0.16 [-0.13, 0.42]	0.73 [0.25, 0.95]	0.47 [0.00, 0.75]	0.26 [0.00, 0.50]	0.88 [0.82, 0.94]
lr0.0001_wd0.0_h256_d4	0.79 [0.67, 0.95]	0.55 [0.34, 0.71]	0.35 [0.29, 0.38]	0.65 [0.58, 0.71]	0.24 [0.17, 0.30]	0.62 [0.35, 0.90]	0.68 [0.25, 1.00]	0.26 [0.21, 0.33]	0.93 [0.86, 1.00]
lr0.0001_wd0.0_h400_d4	0.78 [0.68, 0.93]	0.53 [0.35, 0.71]	0.43 [0.29, 0.75]	0.69 [0.58, 0.95]	0.33 [0.17, 0.73]	0.61 [0.20, 0.90]	0.78 [0.25, 1.00]	0.33 [0.20, 0.60]	0.96 [0.86, 1.00]
lr0.0001_wd0.0_h512_d4	0.77 [0.70, 0.89]	0.49 [0.30, 0.61]	0.25 [0.00, 0.50]	0.56 [0.50, 0.75]	0.09 [0.00, 0.42]	0.53 [0.00, 1.00]	0.58 [0.00, 1.00]	0.18 [0.00, 0.40]	0.52 [0.00, 0.94]
lr0.0001_wd0.0001_h256_d3	0.76 [0.68, 0.84]	0.51 [0.28, 0.71]	0.38 [0.19, 0.50]	0.65 [0.41, 0.84]	0.23 [-0.16, 0.48]	0.46 [0.15, 0.85]	0.83 [0.50, 1.00]	0.26 [0.11, 0.40]	0.93 [0.75, 1.00]
lr0.0001_wd0.0001_h400_d3	0.76 [0.63, 0.91]	0.53 [0.24, 0.65]	0.35 [0.22, 0.50]	0.61 [0.49, 0.79]	0.19 [-0.01, 0.40]	0.44 [0.15, 0.90]	0.78 [0.50, 1.00]	0.25 [0.13, 0.50]	0.92 [0.83, 1.00]
lr0.0001_wd0.0001_h512_d3	0.80 [0.70, 0.93]	0.54 [0.35, 0.72]	0.21 [0.00, 0.44]	0.55 [0.43, 0.68]	0.09 [-0.17, 0.32]	0.60 [0.10, 1.00]	0.50 [0.00, 1.00]	0.15 [0.00, 0.40]	0.91 [0.81, 1.00]
lr0.0001_wd0.0001_h256_d4	0.81 [0.70, 0.95]	0.55 [0.30, 0.72]	0.27 [0.00, 0.67]	0.61 [0.48, 0.81]	0.21 [-0.09, 0.61]	0.91 [0.68, 1.00]	0.32 [0.00, 0.67]	0.28 [0.00, 0.67]	0.88 [0.83, 0.95]
lr0.0001_wd0.0001_h400_d4	0.77 [0.68, 0.93]	0.52 [0.31, 0.71]	0.33 [0.00, 0.67]	0.65 [0.50, 0.92]	0.24 [0.00, 0.65]	0.82 [0.63, 1.00]	0.48 [0.00, 1.00]	0.26 [0.00, 0.50]	0.90 [0.83, 1.00]
lr0.0001_wd0.0001_h512_d4	0.81 [0.68, 0.96]	0.59 [0.27, 0.81]	0.30 [0.00, 0.67]	0.64 [0.50, 0.92]	0.22 [0.00, 0.65]	0.73 [0.00, 1.00]	0.55 [0.00, 1.00]	0.22 [0.00, 0.50]	0.73 [0.00, 1.00]
lr0.0001_wd0.001_h256_d3	0.80 [0.75, 0.89]	0.46 [0.33, 0.55]	0.26 [0.00, 0.44]	0.58 [0.50, 0.71]	0.13 [0.00, 0.32]	0.61 [0.00, 1.00]	0.55 [0.00, 1.00]	0.20 [0.00, 0.40]	0.72 [0.00, 1.00]
lr0.0001_wd0.001_h400_d3	0.80 [0.70, 0.93]	0.58 [0.36, 0.72]	0.38 [0.30, 0.50]	0.64 [0.55, 0.70]	0.24 [0.08, 0.40]	0.73 [0.35, 0.90]	0.55 [0.33, 0.75]	0.32 [0.19, 0.50]	0.90 [0.88, 0.93]
lr0.0001_wd0.001_h512_d3	0.78 [0.67, 0.91]	0.56 [0.30, 0.71]	0.33 [0.00, 0.44]	0.64 [0.45, 0.73]	0.22 [-0.13, 0.35]	0.70 [0.32, 0.90]	0.58 [0.00, 1.00]	0.24 [0.00, 0.40]	0.92 [0.82, 1.00]
lr0.0001_wd0.001_h256_d4	0.75 [0.67, 0.95]	0.53 [0.28, 0.71]	0.36 [0.29, 0.44]	0.66 [0.58, 0.76]	0.24 [0.12, 0.36]	0.63 [0.40, 0.90]	0.68 [0.25, 1.00]	0.27 [0.20, 0.33]	0.92 [0.86, 1.00]
lr0.0001_wd0.001_h400_d4	0.80 [0.70, 0.96]	0.56 [0.31, 0.87]	0.28 [0.00, 0.67]	0.61 [0.43, 0.92]	0.15 [-0.17, 0.65]	0.78 [0.53, 0.85]	0.43 [0.00, 1.00]	0.22 [0.00, 0.50]	0.88 [0.81, 1.00]
lr0.0001_wd0.001_h512_d4	0.79 [0.69, 0.95]	0.62 [0.28, 0.83]	0.34 [0.00, 0.50]	0.60 [0.43, 0.73]	0.23 [-0.17, 0.55]	0.71 [0.05, 1.00]	0.50 [0.00, 1.00]	0.38 [0.00, 1.00]	0.91 [0.81, 1.00]
lr0.0003_wd0.0_h256_d3	0.79 [0.70, 0.91]	0.57 [0.31, 0.72]	0.45 [0.38, 0.57]	0.72 [0.68, 0.78]	0.37 [0.29, 0.50]	0.67 [0.35, 0.90]	0.77 [0.50, 1.00]	0.36 [0.24, 0.50]	0.96 [0.90, 1.00]
lr0.0003_wd0.0_h400_d3	0.79 [0.70, 0.88]	0.54 [0.34, 0.72]	0.38 [0.00, 0.67]	0.64 [0.48, 0.81]	0.27 [-0.09, 0.61]	0.72 [0.00, 0.95]	0.57 [0.00, 1.00]	0.36 [0.00, 0.67]	0.72 [0.00, 0.95]
lr0.0003_wd0.0_h512_d3	0.80 [0.70, 0.91]	0.57 [0.38, 0.72]	0.49 [0.29, 0.67]	0.71 [0.50, 0.81]	0.38 [0.00, 0.61]	0.66 [0.00, 0.95]	0.77 [0.50, 1.00]	0.41 [0.17, 0.67]	0.76 [0.00, 1.00]
lr0.0003_wd0.0_h256_d4	0.79 [0.67, 0.95]	0.52 [0.31, 0.71]	0.43 [0.29, 0.60]	0.70 [0.50, 0.89]	0.32 [0.00, 0.58]	0.62 [0.00, 0.95]	0.78 [0.25, 1.00]	0.35 [0.17, 0.50]	0.76 [0.00, 1.00]
lr0.0003_wd0.0_h400_d4	0.81 [0.74, 0.89]	0.51 [0.34, 0.68]	0.33 [0.00, 0.57]	0.61 [0.50, 0.78]	0.21 [0.00, 0.50]	0.58 [0.00, 1.00]	0.63 [0.00, 1.00]	0.27 [0.00, 0.50]	0.74 [0.00, 1.00]
lr0.0003_wd0.0_h512_d4	0.81 [0.71, 0.91]	0.55 [0.34, 0.74]	0.43 [0.31, 0.55]	0.70 [0.55, 0.87]	0.33 [0.13, 0.53]	0.57 [0.10, 0.90]	0.83 [0.50, 1.00]	0.32 [0.18, 0.50]	0.97 [0.90, 1.00]
lr0.0003_wd0.0001_h256_d3	0.79 [0.66, 0.95]	0.55 [0.26, 0.68]	0.29 [0.00, 0.46]	0.59 [0.43, 0.82]	0.13 [-0.17, 0.44]	0.53 [0.00, 0.85]	0.65 [0.00, 1.00]	0.20 [0.00, 0.40]	0.71 [0.00, 1.00]
lr0.0003_wd0.0001_h400_d3	0.80 [0.68, 0.91]	0.60 [0.27, 0.72]	0.41 [0.00, 0.67]	0.68 [0.40, 0.92]	0.29 [-0.20, 0.65]	0.74 [0.45, 0.90]	0.63 [0.00, 1.00]	0.31 [0.00, 0.60]	0.92 [0.80, 1.00]
lr0.0003_wd0.0001_h512_d3	0.79 [0.68, 0.90]	0.57 [0.30, 0.68]	0.36 [0.00, 0.67]	0.65 [0.50, 0.81]	0.26 [0.00, 0.61]	0.77 [0.42, 1.00]	0.52 [0.00, 0.75]	0.30 [0.00, 0.67]	0.90 [0.83, 0.95]
lr0.0003_wd0.0001_h256_d4	0.83 [0.78, 0.93]	0.59 [0.37, 0.70]	0.30 [0.00, 0.75]	0.61 [0.43, 0.95]	0.18 [-0.17, 0.73]	0.72 [0.00, 1.00]	0.50 [0.00, 1.00]	0.23 [0.00, 0.60]	0.71 [0.00, 1.00]
lr0.0003_wd0.0001_h400_d4	0.82 [0.72, 0.94]	0.58 [0.35, 0.75]	0.43 [0.33, 0.57]	0.69 [0.60, 0.78]	0.33 [0.25, 0.50]	0.72 [0.30, 0.95]	0.67 [0.25, 1.00]	0.37 [0.22, 0.50]	0.93 [0.86, 1.00]
lr0.0003_wd0.0001_h512_d4	0.77 [0.65, 0.95]	0.57 [0.35, 0.71]	0.43 [0.36, 0.55]	0.70 [0.63, 0.87]	0.32 [0.20, 0.53]	0.73 [0.55, 0.84]	0.68 [0.50, 1.00]	0.32 [0.25, 0.40]	0.92 [0.88, 1.00]
lr0.0003_wd0.001_h256_d3	0.81 [0.74, 0.95]	0.60 [0.35, 0.72]	0.39 [0.24, 0.60]	0.66 [0.50, 0.80]	0.27 [0.00, 0.52]	0.48 [0.00, 0.85]	0.85 [0.50, 1.00]	0.28 [0.14, 0.50]	0.77 [0.00, 1.00]
lr0.0003_wd0.001_h400_d3	0.81 [0.74, 0.95]	0.62 [0.33, 0.76]	0.41 [0.24, 0.67]	0.67 [0.50, 0.90]	0.29 [0.00, 0.63]	0.53 [0.00, 0.95]	0.82 [0.33, 1.00]	0.32 [0.14, 0.50]	0.76 [0.00, 1.00]
lr0.0003_wd0.001_h512_d3	0.80 [0.68, 0.91]	0.58 [0.34, 0.73]	0.41 [0.33, 0.57]	0.68 [0.65, 0.73]	0.32 [0.21, 0.51]	0.63 [0.35, 0.95]	0.73 [0.50, 1.00]	0.34 [0.20, 0.67]	0.94 [0.89, 1.00]
lr0.0003_wd0.001_h256_d4	0.78 [0.70, 0.95]	0.56 [0.34, 0.72]	0.37 [0.00, 0.75]	0.66 [0.43, 0.95]	0.27 [-0.17, 0.73]	0.84 [0.68, 0.95]	0.48 [0.00, 1.00]	0.34 [0.00, 0.60]	0.90 [0.81, 1.00]
lr0.0003_wd0.001_h400_d4	0.84 [0.79, 0.93]	0.55 [0.39, 0.69]	0.48 [0.24, 0.75]	0.72 [0.50, 0.95]	0.38 [0.00, 0.73]	0.54 [0.00, 0.95]	0.90 [0.50, 1.00]	0.39 [0.14, 0.67]	0.78 [0.00, 1.00]
lr0.0003_wd0.001_h512_d4	0.81 [0.75, 0.96]	0.60 [0.37, 0.81]	0.36 [0.29, 0.44]	0.64 [0.50, 0.75]	0.22 [0.00, 0.38]	0.44 [0.00, 0.85]	0.83 [0.50, 1.00]	0.25 [0.17, 0.40]	0.76 [0.00, 1.00]
lr0.001_wd0.0_h256_d3	0.80 [0.72, 0.91]	0.58 [0.38, 0.72]	0.40 [0.29, 0.60]	0.66 [0.50, 0.89]	0.25 [0.00, 0.58]	0.53 [0.00, 0.90]	0.78 [0.50, 1.00]	0.30 [0.17, 0.50]	0.74 [0.00, 1.00]
lr0.001_wd0.0_h400_d3	0.84 [0.71, 1.00]	0.66 [0.43, 1.00]	0.50 [0.32, 0.60]	0.76 [0.58, 0.87]	0.41 [0.12, 0.53]	0.70 [0.40, 0.85]	0.83 [0.67, 1.00]	0.37 [0.20, 0.50]	0.95 [0.89, 1.00]
lr0.001_wd0.0_h512_d3	0.82 [0.74, 0.91]	0.59 [0.35, 0.72]	0.51 [0.29, 0.67]	0.78 [0.61, 0.92]	0.44 [0.19, 0.65]	0.65 [0.21, 0.84]	0.90 [0.75, 1.00]	0.37 [0.17, 0.50]	0.98 [0.94, 1.00]
lr0.001_wd0.0_h256_d4	0.82 [0.74, 0.96]	0.53 [0.29, 0.81]	0.28 [0.00, 0.60]	0.64 [0.50, 0.84]	0.22 [0.00, 0.52]	0.79 [0.40, 1.00]	0.50 [0.00, 1.00]	0.21 [0.00, 0.50]	0.91 [0.83, 1.00]
lr0.001_wd0.0_h400_d4	0.80 [0.68, 1.00]	0.65 [0.32, 1.00]	0.40 [0.27, 0.60]	0.67 [0.50, 0.84]	0.26 [0.00, 0.52]	0.45 [0.00, 0.85]	0.88 [0.67, 1.00]	0.28 [0.17, 0.50]	0.77 [0.00, 1.00]
lr0.001_wd0.0_h512_d4	0.81 [0.73, 0.95]	0.53 [0.29, 0.76]	0.38 [0.00, 0.67]	0.68 [0.50, 0.92]	0.28 [0.00, 0.65]	0.66 [0.00, 1.00]	0.70 [0.00, 1.00]	0.28 [0.00, 0.50]	0.75 [0.00, 1.00]
lr0.001_wd0.0001_h256_d3	0.79 [0.67, 0.89]	0.59 [0.33, 0.82]	0.41 [0.32, 0.60]	0.68 [0.63, 0.80]	0.34 [0.21, 0.52]	0.63 [0.32, 1.00]	0.73 [0.25, 1.00]	0.43 [0.19, 1.00]	0.95 [0.87, 1.00]
lr0.001_wd0.0001_h400_d3	0.81 [0.73, 0.93]	0.53 [0.35, 0.62]	0.40 [0.27, 0.55]	0.68 [0.58, 0.78]	0.29 [0.16, 0.45]	0.52 [0.16, 0.80]	0.83 [0.67, 1.00]	0.28 [0.16, 0.43]	0.96 [0.93, 1.00]
lr0.001_wd0.0001_h512_d3	0.79 [0.70, 0.91]	0.59 [0.36, 0.79]	0.51 [0.42, 0.67]	0.75 [0.70, 0.83]	0.42 [0.35, 0.60]	0.78 [0.45, 0.90]	0.72 [0.50, 1.00]	0.42 [0.27, 0.60]	0.95 [0.90, 1.00]
lr0.001_wd0.0001_h256_d4	0.82 [0.72, 0.96]	0.63 [0.36, 0.87]	0.48 [0.29, 0.67]	0.73 [0.50, 0.84]	0.37 [0.00, 0.61]	0.58 [0.00, 0.95]	0.88 [0.67, 1.00]	0.37 [0.17, 0.67]	0.78 [0.00, 1.00]
lr0.001_wd0.0001_h400_d4	0.82 [0.74, 0.95]	0.58 [0.31, 0.76]	0.32 [0.29, 0.40]	0.59 [0.50, 0.76]	0.15 [0.00, 0.36]	0.31 [0.00, 0.89]	0.87 [0.33, 1.00]	0.22 [0.17, 0.33]	0.58 [0.00, 1.00]
lr0.001_wd0.0001_h512_d4	0.81 [0.72, 0.96]	0.61 [0.36, 0.81]	0.32 [0.00, 0.46]	0.64 [0.50, 0.79]	0.21 [0.00, 0.40]	0.54 [0.00, 1.00]	0.75 [0.00, 1.00]	0.20 [0.00, 0.33]	0.76 [0.00, 1.00]
lr0.001_wd0.001_h256_d3	0.81 [0.70, 0.95]	0.57 [0.36, 0.76]	0.44 [0.35, 0.60]	0.72 [0.63, 0.89]	0.34 [0.19, 0.58]	0.70 [0.50, 0.85]	0.73 [0.50, 1.00]	0.33 [0.23, 0.43]	0.93 [0.89, 1.00]
lr0.001_wd0.001_h400_d3	0.77 [0.70, 0.91]	0.50 [0.34, 0.66]	0.40 [0.24, 0.55]	0.67 [0.50, 0.82]	0.28 [0.00, 0.45]	0.64 [0.00, 0.95]	0.70 [0.25, 1.00]	0.34 [0.14, 0.50]	0.74 [0.00, 1.00]
lr0.001_wd0.001_h512_d3	0.83 [0.68, 0.95]	0.55 [0.27, 0.68]	0.53 [0.30, 0.75]	0.78 [0.63, 0.95]	0.46 [0.20, 0.73]	0.70 [0.26, 0.90]	0.85 [0.50, 1.00]	0.41 [0.18, 0.60]	0.97 [0.88, 1.00]
lr0.001_wd0.001_h256_d4	0.77 [0.70, 0.89]	0.50 [0.38, 0.74]	0.34 [0.00, 0.57]	0.64 [0.50, 0.78]	0.23 [0.00, 0.50]	0.74 [0.35, 1.00]	0.53 [0.00, 0.75]	0.26 [0.00, 0.50]	0.90 [0.86, 0.94]
lr0.001_wd0.001_h400_d4	0.82 [0.72, 0.93]	0.55 [0.32, 0.78]	0.46 [0.35, 0.57]	0.71 [0.63, 0.78]	0.35 [0.22, 0.50]	0.66 [0.25, 0.89]	0.77 [0.67, 1.00]	0.34 [0.21, 0.50]	0.95 [0.92, 1.00]
lr0.001_wd0.001_h512_d4	0.81 [0.71, 0.95]	0.52 [0.30, 0.83]	0.38 [0.00, 0.57]	0.71 [0.50, 0.85]	0.33 [0.00, 0.53]	0.68 [0.25, 1.00]	0.75 [0.00, 1.00]	0.26 [0.00, 0.40]	0.96 [0.86, 1.00]

Supplementary Table 13: Grid search results on the classification task on GDHCM prospective dataset (choosing 50% as the threshold for clinical variables selection). Reported metrics are AUC, AUPRC, F1, Balanced Accuracy, MCC, Specificity, Sensitivity, PPV, and NPV.

Config	AUC	AUPRC	F1	Balanced_Accuracy	MCC	Specificity	Sensitivity	PPV	NPV
lr0.0001_wd0.0_h256_d1	0.65 [0.63, 0.68]	0.37 [0.31, 0.47]	0.42 [0.30, 0.53]	0.63 [0.55, 0.71]	0.25 [0.09, 0.38]	0.64 [0.24, 0.89]	0.63 [0.33, 0.92]	**0.36 [0.26, 0.47]**	0.88 [0.81, 0.96]
lr0.0001_wd0.0_h400_d1	0.69 [0.66, 0.71]	0.39 [0.34, 0.42]	0.45 [0.41, 0.49]	**0.65 [0.60, 0.70]**	0.27 [0.23, 0.33]	0.52 [0.20, 0.80]	0.78 [0.46, 1.00]	0.33 [0.26, 0.40]	**0.92 [0.84, 1.00]**
lr0.0001_wd0.0_h512_d1	0.65 [0.63, 0.68]	0.40 [0.32, 0.51]	0.41 [0.37, 0.43]	0.61 [0.59, 0.63]	0.20 [0.16, 0.27]	0.49 [0.26, 0.72]	0.73 [0.46, 1.00]	0.29 [0.26, 0.32]	0.89 [0.82, 1.00]
lr0.0001_wd0.0_h256_d2	0.65 [0.63, 0.68]	0.40 [0.33, 0.46]	0.34 [0.22, 0.41]	0.55 [0.51, 0.60]	0.11 [0.02, 0.20]	0.44 [0.20, 0.93]	0.67 [0.15, 0.92]	0.27 [0.23, 0.40]	0.85 [0.79, 0.93]
lr0.0001_wd0.0_h400_d2	0.67 [0.64, 0.72]	0.38 [0.33, 0.48]	0.42 [0.29, 0.56]	0.62 [0.53, 0.76]	0.23 [0.11, 0.44]	0.54 [0.07, 0.89]	0.70 [0.23, 1.00]	0.33 [0.22, 0.45]	0.90 [0.80, 1.00]
lr0.0001_wd0.0_h512_d2	0.65 [0.60, 0.74]	0.37 [0.30, 0.43]	0.37 [0.26, 0.41]	0.59 [0.54, 0.62]	0.17 [0.09, 0.23]	0.53 [0.26, 0.85]	0.65 [0.23, 0.92]	0.29 [0.26, 0.38]	0.87 [0.80, 0.93]
lr0.0001_wd0.0001_h256_d1	0.68 [0.63, 0.78]	**0.45 [0.40, 0.55]**	0.41 [0.38, 0.48]	0.61 [0.55, 0.68]	0.19 [0.11, 0.30]	0.38 [0.17, 0.59]	**0.84 [0.75, 0.92]**	0.28 [0.24, 0.34]	0.90 [0.87, 0.94]
lr0.0001_wd0.0001_h400_d1	0.66 [0.61, 0.69]	0.40 [0.31, 0.46]	0.39 [0.31, 0.45]	0.60 [0.55, 0.66]	0.18 [0.11, 0.27]	0.52 [0.17, 0.80]	0.68 [0.31, 0.92]	0.29 [0.24, 0.32]	0.87 [0.80, 0.91]
lr0.0001_wd0.0001_h512_d1	0.67 [0.63, 0.72]	0.37 [0.30, 0.41]	0.38 [0.34, 0.41]	0.57 [0.49, 0.63]	0.12 [-0.01, 0.21]	0.42 [0.15, 0.67]	0.71 [0.58, 0.92]	0.27 [0.22, 0.32]	0.84 [0.77, 0.88]
lr0.0001_wd0.0001_h256_d2	0.66 [0.64, 0.72]	0.37 [0.33, 0.42]	0.37 [0.25, 0.47]	0.58 [0.52, 0.67]	0.14 [0.03, 0.29]	0.50 [0.15, 0.83]	0.65 [0.23, 0.92]	0.27 [0.22, 0.36]	0.85 [0.79, 0.90]
lr0.0001_wd0.0001_h400_d2	0.64 [0.63, 0.66]	0.38 [0.31, 0.49]	0.38 [0.36, 0.44]	0.57 [0.52, 0.66]	0.14 [0.04, 0.26]	0.38 [0.04, 0.78]	0.76 [0.38, 1.00]	0.27 [0.22, 0.33]	0.88 [0.82, 1.00]
lr0.0001_wd0.0001_h512_d2	0.66 [0.62, 0.69]	0.40 [0.36, 0.50]	0.39 [0.25, 0.46]	0.61 [0.53, 0.66]	0.19 [0.06, 0.27]	0.56 [0.40, 0.83]	0.67 [0.23, 0.92]	0.29 [0.27, 0.31]	0.88 [0.79, 0.95]
lr0.0001_wd0.001_h256_d1	0.68 [0.65, 0.72]	0.44 [0.37, 0.48]	0.37 [0.11, 0.47]	0.61 [0.49, 0.68]	0.18 [-0.01, 0.30]	0.55 [0.35, 0.91]	0.67 [0.08, 0.85]	0.27 [0.20, 0.32]	0.88 [0.78, 0.92]
lr0.0001_wd0.001_h400_d1	0.65 [0.63, 0.67]	0.37 [0.34, 0.43]	0.42 [0.40, 0.47]	0.62 [0.60, 0.66]	0.21 [0.16, 0.29]	0.59 [0.35, 0.78]	0.65 [0.54, 0.85]	0.32 [0.27, 0.41]	0.86 [0.85, 0.89]
lr0.0001_wd0.001_h512_d1	0.67 [0.63, 0.71]	0.41 [0.34, 0.51]	0.37 [0.28, 0.41]	0.58 [0.52, 0.62]	0.15 [0.04, 0.24]	0.55 [0.28, 0.83]	0.62 [0.31, 0.92]	0.28 [0.25, 0.38]	**0.85 [0.79, 0.93]**
lr0.0001_wd0.001_h256_d2	0.65 [0.63, 0.70]	0.43 [0.30, 0.57]	0.36 [0.30, 0.41]	0.56 [0.50, 0.60]	0.11 [0.00, 0.17]	0.35 [0.00, 0.78]	0.77 [0.31, 1.00]	0.25 [0.21, 0.29]	0.69 [0.00, 0.90]
lr0.0001_wd0.001_h400_d2	0.67 [0.64, 0.70]	0.36 [0.31, 0.47]	0.39 [0.36, 0.44]	0.59 [0.54, 0.64]	0.17 [0.12, 0.25]	0.51 [0.09, 0.78]	0.67 [0.46, 1.00]	0.30 [0.23, 0.38]	0.87 [0.84, 1.00]
lr0.0001_wd0.001_h512_d2	0.66 [0.61, 0.73]	0.41 [0.31, 0.52]	0.37 [0.17, 0.46]	0.59 [0.49, 0.67]	0.16 [-0.02, 0.32]	0.52 [0.13, 0.83]	0.67 [0.15, 1.00]	0.27 [0.20, 0.35]	0.87 [0.78, 1.00]
lr0.0003_wd0.0_h256_d1	0.66 [0.64, 0.71]	0.38 [0.31, 0.44]	0.39 [0.34, 0.44]	0.59 [0.54, 0.65]	0.17 [0.07, 0.26]	0.46 [0.13, 0.65]	0.73 [0.54, 1.00]	0.28 [0.24, 0.30]	0.89 [0.81, 1.00]
lr0.0003_wd0.0_h400_d1	0.67 [0.65, 0.69]	0.42 [0.35, 0.57]	0.40 [0.37, 0.47]	0.61 [0.56, 0.68]	**0.19 [0.11, 0.30]**	0.53 [0.26, 0.83]	0.68 [0.38, 0.92]	0.31 [0.24, 0.38]	0.87 [0.83, 0.92]
lr0.0003_wd0.0_h512_d1	0.67 [0.64, 0.72]	0.42 [0.30, 0.51]	0.36 [0.24, 0.40]	0.58 [0.52, 0.60]	0.14 [0.04, 0.19]	0.55 [0.29, 0.80]	0.61 [0.23, 0.92]	0.28 [0.25, 0.33]	0.85 [0.79, 0.93]
lr0.0003_wd0.0_h256_d2	0.67 [0.62, 0.71]	0.44 [0.34, 0.53]	0.38 [0.25, 0.44]	0.60 [0.51, 0.66]	0.19 [0.02, 0.26]	**0.64 [0.41, 0.96]**	0.57 [0.17, 0.77]	0.34 [0.23, 0.50]	0.85 [0.79, 0.88]
lr0.0003_wd0.0_h400_d2	0.66 [0.63, 0.69]	0.39 [0.29, 0.50]	0.42 [0.35, 0.48]	0.61 [0.49, 0.69]	0.19 [-0.02, 0.33]	0.41 [0.07, 0.67]	0.82 [0.67, 0.92]	0.29 [0.22, 0.35]	0.87 [0.75, 0.95]
lr0.0003_wd0.0_h512_d2	0.67 [0.63, 0.69]	0.39 [0.35, 0.48]	0.42 [0.36, 0.54]	0.61 [0.55, 0.72]	0.22 [0.08, 0.41]	0.56 [0.24, 0.85]	0.67 [0.38, 1.00]	0.33 [0.25, 0.50]	0.88 [0.81, 1.00]
lr0.0003_wd0.0001_h256_d1	0.69 [0.65, 0.79]	0.41 [0.32, 0.61]	0.43 [0.41, 0.47]	0.63 [0.60, 0.67]	0.24 [0.20, 0.28]	0.45 [0.24, 0.85]	0.81 [0.42, 1.00]	0.31 [0.27, 0.42]	**0.92 [0.85, 1.00]**
lr0.0003_wd0.0001_h400_d1	0.65 [0.61, 0.71]	0.33 [0.30, 0.34]	0.33 [0.10, 0.43]	0.55 [0.46, 0.65]	0.09 [-0.09, 0.24]	0.43 [0.02, 0.85]	0.67 [0.08, 1.00]	0.23 [0.12, 0.30]	0.87 [0.76, 1.00]
lr0.0003_wd0.0001_h512_d1	0.67 [0.65, 0.69]	0.44 [0.38, 0.49]	0.43 [0.40, 0.45]	0.63 [0.61, 0.66]	0.25 [0.19, 0.29]	0.65 [0.37, 0.87]	0.62 [0.38, 0.85]	**0.36 [0.28, 0.45]**	0.87 [0.83, 0.90]
lr0.0003_wd0.0001_h256_d2	0.66 [0.62, 0.70]	0.38 [0.31, 0.45]	0.42 [0.35, 0.50]	0.60 [0.49, 0.70]	0.18 [-0.02, 0.35]	0.38 [0.07, 0.74]	0.82 [0.67, 0.92]	0.29 [0.22, 0.40]	0.87 [0.75, 0.92]
lr0.0003_wd0.0001_h400_d2	0.66 [0.65, 0.68]	0.37 [0.31, 0.42]	0.39 [0.35, 0.44]	0.59 [0.50, 0.65]	0.16 [0.00, 0.24]	0.43 [0.00, 0.83]	0.75 [0.38, 1.00]	0.29 [0.21, 0.38]	0.69 [0.00, 0.91]
lr0.0003_wd0.0001_h512_d2	0.66 [0.62, 0.68]	0.37 [0.32, 0.42]	0.40 [0.32, 0.47]	0.60 [0.50, 0.67]	0.18 [0.00, 0.29]	0.58 [0.33, 0.80]	0.62 [0.38, 0.77]	0.31 [0.21, 0.36]	0.85 [0.79, 0.88]
lr0.0003_wd0.001_h256_d1	0.69 [0.66, 0.73]	0.40 [0.33, 0.48]	0.43 [0.35, 0.48]	0.63 [0.55, 0.68]	0.23 [0.09, 0.31]	0.58 [0.51, 0.74]	0.68 [0.54, 0.77]	0.32 [0.26, 0.40]	0.87 [0.81, 0.90]
lr0.0003_wd0.001_h400_d1	0.67 [0.63, 0.69]	0.41 [0.31, 0.56]	0.41 [0.39, 0.44]	0.62 [0.61, 0.64]	0.21 [0.17, 0.25]	0.57 [0.33, 0.74]	0.66 [0.50, 0.92]	0.31 [0.28, 0.37]	0.87 [0.84, 0.94]
lr0.0003_wd0.001_h512_d1	0.66 [0.63, 0.70]	0.38 [0.34, 0.42]	0.44 [0.36, 0.50]	0.63 [0.57, 0.69]	0.23 [0.11, 0.35]	0.54 [0.35, 0.80]	0.73 [0.58, 0.85]	0.32 [0.25, 0.44]	0.88 [0.84, 0.90]
lr0.0003_wd0.001_h256_d2	0.66 [0.62, 0.70]	0.35 [0.33, 0.36]	0.41 [0.34, 0.47]	0.61 [0.47, 0.68]	0.17 [-0.13, 0.34]	0.42 [0.02, 0.78]	0.79 [0.38, 1.00]	**0.29 [0.21, 0.33]**	0.83 [0.50, 1.00]
lr0.0003_wd0.001_h400_d2	0.67 [0.63, 0.69]	0.38 [0.34, 0.48]	0.42 [0.35, 0.48]	0.62 [0.57, 0.68]	0.23 [0.12, 0.31]	0.55 [0.20, 0.83]	0.69 [0.42, 1.00]	0.32 [0.26, 0.40]	0.90 [0.83, 1.00]
lr0.0003_wd0.001_h512_d2	0.65 [0.61, 0.67]	0.36 [0.33, 0.42]	0.41 [0.37, 0.48]	0.61 [0.56, 0.69]	0.20 [0.13, 0.32]	0.59 [0.20, 0.72]	0.63 [0.46, 0.92]	0.32 [0.24, 0.38]	0.86 [0.82, 0.90]
lr0.001_wd0.0_h400_d1	0.67 [0.61, 0.71]	0.40 [0.35, 0.52]	0.36 [0.20, 0.53]	0.58 [0.52, 0.72]	0.13 [0.04, 0.37]	0.55 [0.33, 0.87]	0.60 [0.17, 0.85]	0.28 [0.22, 0.43]	0.84 [0.80, 0.89]
lr0.001_wd0.0_h512_d1	0.65 [0.64, 0.67]	0.42 [0.30, 0.52]	0.38 [0.33, 0.44]	0.59 [0.51, 0.64]	0.15 [0.01, 0.24]	0.52 [0.26, 0.76]	0.65 [0.38, 0.85]	0.28 [0.21, 0.31]	0.85 [0.80, 0.91]
lr0.001_wd0.0_h256_d2	0.67 [0.62, 0.76]	0.40 [0.33, 0.51]	0.38 [0.30, 0.46]	0.58 [0.55, 0.66]	0.16 [0.10, 0.27]	0.50 [0.11, 0.89]	0.66 [0.25, 1.00]	0.29 [0.24, 0.38]	0.87 [0.82, 1.00]
lr0.001_wd0.0_h400_d2	0.68 [0.63, 0.76]	0.41 [0.38, 0.45]	0.40 [0.37, 0.42]	0.59 [0.55, 0.62]	0.18 [0.11, 0.24]	**0.47 [0.18, 0.85]**	0.72 [0.38, 0.92]	**0.30 [0.23, 0.42]**	0.87 [0.83, 0.91]
lr0.001_wd0.0_h512_d2	0.69 [0.65, 0.74]	0.40 [0.32, 0.49]	0.42 [0.37, 0.47]	0.61 [0.55, 0.68]	0.20 [0.09, 0.30]	0.53 [0.22, 0.70]	0.70 [0.54, 0.92]	0.30 [0.24, 0.36]	0.87 [0.83, 0.91]
lr0.001_wd0.0001_h256_d1	**0.67 [0.62, 0.74]**	0.42 [0.29, 0.49]	0.38 [0.35, 0.43]	0.58 [0.54, 0.63]	0.14 [0.07, 0.21]	0.45 [0.15, 0.69]	**0.71 [0.50, 0.92]**	0.27 [0.23, 0.31]	**0.85 [0.83, 0.88]**
lr0.001_wd0.0001_h400_d1	0.69 [0.65, 0.80]	0.39 [0.34, 0.48]	0.43 [0.38, 0.50]	0.62 [0.54, 0.70]	0.22 [0.09, 0.35]	0.41 [0.15, 0.74]	**0.84 [0.67, 1.00]**	0.30 [0.24, 0.40]	0.91 [0.87, 1.00]
lr0.001_wd0.0001_h512_d1	0.65 [0.61, 0.67]	0.38 [0.33, 0.42]	0.42 [0.39, 0.47]	**0.62 [0.58, 0.68]**	0.21 [0.14, 0.30]	0.54 [0.30, 0.76]	**0.71 [0.50, 0.85]**	0.31 [0.26, 0.36]	**0.87 [0.85, 0.89]**
lr0.001_wd0.0001_h256_d2	0.69 [0.60, 0.76]	**0.45 [0.34, 0.56]**	0.42 [0.36, 0.58]	0.61 [0.52, 0.75]	0.20 [0.04, 0.45]	0.51 [0.13, 0.80]	**0.71 [0.50, 0.92]**	0.32 [0.23, 0.50]	**0.86 [0.82, 0.90]**
lr0.001_wd0.0001_h400_d2	0.67 [0.62, 0.70]	0.36 [0.31, 0.40]	0.38 [0.17, 0.53]	0.60 [0.49, 0.72]	0.18 [-0.02, 0.39]	**0.68 [0.50, 0.83]**	0.53 [0.15, 0.69]	0.31 [0.20, 0.44]	0.84 [0.78, 0.90]
lr0.001_wd0.0001_h512_d2	**0.70 [0.66, 0.76]**	0.41 [0.36, 0.46]	0.40 [0.25, 0.53]	0.62 [0.53, 0.74]	0.21 [0.06, 0.41]	0.62 [0.33, 0.83]	0.62 [0.23, 0.92]	0.31 [0.26, 0.38]	**0.87 [0.79, 0.96]**
lr0.001_wd0.001_h256_d1	**0.70 [0.67, 0.76]**	0.38 [0.34, 0.44]	0.43 [0.36, 0.48]	0.64 [0.58, 0.69]	0.24 [0.14, 0.32]	0.53 [0.29, 0.72]	0.75 [0.46, 0.92]	0.31 [0.26, 0.38]	0.90 [0.82, 0.95]
lr0.001_wd0.001_h400_d1	0.67 [0.61, 0.75]	0.38 [0.33, 0.41]	0.41 [0.36, 0.53]	0.62 [0.54, 0.73]	0.21 [0.07, 0.39]	0.55 [0.30, 0.80]	0.68 [0.38, 0.92]	0.31 [0.24, 0.41]	0.87 [0.82, 0.93]
lr0.001_wd0.001_h512_d1	0.66 [0.61, 0.69]	0.38 [0.32, 0.47]	0.42 [0.40, 0.49]	0.63 [0.60, 0.68]	0.22 [0.17, 0.31]	0.58 [0.50, 0.67]	0.68 [0.62, 0.75]	0.31 [0.28, 0.38]	**0.87 [0.84, 0.89]**
lr0.001_wd0.001_h256_d2	0.69 [0.62, 0.75]	0.40 [0.33, 0.46]	0.44 [0.37, 0.50]	0.64 [0.58, 0.69]	0.24 [0.13, 0.35]	0.58 [0.41, 0.80]	0.70 [0.54, 0.85]	0.33 [0.26, 0.44]	0.88 [0.84, 0.92]
lr0.001_wd0.001_h400_d2	**0.69 [0.63, 0.78]**	0.40 [0.35, 0.45]	**0.43 [0.33, 0.56]**	0.64 [0.55, 0.74]	**0.24 [0.08, 0.41]**	0.65 [0.43, 0.85]	0.63 [0.33, 0.85]	0.34 [0.26, 0.43]	0.87 [0.81, 0.92]
lr0.001_wd0.001_h512_d2	0.67 [0.64, 0.71]	0.39 [0.32, 0.51]	0.40 [0.33, 0.46]	0.60 [0.48, 0.66]	0.16 [-0.07, 0.31]	0.40 [0.04, 0.61]	0.79 [0.58, 1.00]	0.28 [0.20, 0.32]	0.85 [0.67, 1.00]
**lr0.001_wd0.0_h256_d1**	0.69 [0.63, 0.76]	**0.38 [0.35, 0.45]**	**0.47 [0.37, 0.57]**	**0.65 [0.52, 0.75]**	**0.28 [0.10, 0.45]**	0.56 [0.04, 0.83]	0.74 [0.62, 1.00]	**0.36 [0.23, 0.50]**	0.91 [0.85, 1.00]
Rank	2	8	1	1	1	8	9	1	2

Supplementary Table 14: Grid search results on the classification task on SYSMH external validation dataset (choosing 50% as the threshold for clinical variables selection). Reported metrics are AUC, AUPRC, F1, Balanced Accuracy, MCC, Specificity, Sensitivity, PPV, and NPV. Bold values indicate the best performance across all settings, and underlined values denote the second-best performance. The second-to-last row corresponds to the hyperparameter configuration we selected. The row labeled *Rank* indicates the relative position of the selected hyperparameter configuration among all candidate settings based on its validation performance across each metric.

Config	AUC	AUPRC	F1	Balanced_Accuracy	MCC	Specificity	Sensitivity	PPV	NPV
lr1e-05_wd0.005_h1024_d3	0.66 [0.62, 0.74]	0.42 [0.30, 0.54]	0.34 [0.30, 0.38]	0.49 [0.43, 0.53]	-0.03 [-0.27, 0.12]	0.11 [0.00, 0.48]	0.87 [0.54, 1.00]	0.21 [0.18, 0.23]	0.42 [0.00, 1.00]
lr1e-05_wd0.005_h2048_d3	0.67 [0.63, 0.78]	0.43 [0.31, 0.57]	0.37 [0.32, 0.43]	0.53 [0.48, 0.63]	0.06 [-0.03, 0.22]	0.20 [0.00, 0.41]	0.87 [0.75, 1.00]	0.23 [0.20, 0.29]	0.50 [0.00, 0.90]
lr1e-05_wd0.005_h1024_d4	0.66 [0.62, 0.74]	0.36 [0.28, 0.40]	0.38 [0.34, 0.44]	0.55 [0.49, 0.66]	0.08 [-0.03, 0.26]	0.22 [0.00, 0.57]	0.89 [0.75, 1.00]	0.25 [0.21, 0.31]	0.50 [0.00, 0.90]
lr1e-05_wd0.005_h2048_d4	0.66 [0.63, 0.70]	0.39 [0.34, 0.46]	0.33 [0.00, 0.50]	0.57 [0.50, 0.71]	0.11 [0.00, 0.35]	0.41 [0.00, 1.00]	0.72 [0.00, 1.00]	0.22 [0.00, 0.38]	0.52 [0.00, 0.91]
lr1e-05_wd0.01_h1024_d3	0.65 [0.60, 0.71]	0.40 [0.27, 0.46]	0.40 [0.35, 0.49]	0.57 [0.50, 0.70]	0.12 [0.00, 0.33]	0.27 [0.00, 0.65]	0.87 [0.75, 1.00]	0.26 [0.21, 0.36]	0.53 [0.00, 0.91]
lr1e-05_wd0.01_h2048_d3	0.66 [0.64, 0.75]	0.37 [0.29, 0.40]	0.29 [0.00, 0.40]	0.52 [0.50, 0.61]	0.04 [0.00, 0.19]	0.30 [0.00, 1.00]	0.75 [0.00, 1.00]	0.18 [0.00, 0.27]	0.33 [0.00, 0.88]
lr1e-05_wd0.01_h1024_d4	0.65 [0.60, 0.71]	0.42 [0.30, 0.51]	0.36 [0.35, 0.39]	0.52 [0.50, 0.57]	0.04 [0.00, 0.15]	0.05 [0.00, 0.22]	0.98 [0.92, 1.00]	0.22 [0.21, 0.25]	0.38 [0.00, 1.00]
lr1e-05_wd0.01_h2048_d4	0.66 [0.62, 0.74]	0.41 [0.28, 0.48]	0.37 [0.35, 0.42]	0.53 [0.50, 0.64]	0.06 [0.00, 0.22]	0.20 [0.00, 0.52]	0.87 [0.62, 1.00]	0.24 [0.21, 0.29]	0.34 [0.00, 0.89]
lr3e-05_wd0.005_h1024_d3	0.67 [0.63, 0.70]	0.39 [0.35, 0.42]	0.35 [0.31, 0.40]	0.52 [0.46, 0.58]	0.05 [-0.11, 0.17]	0.23 [0.00, 0.85]	0.81 [0.31, 1.00]	0.25 [0.19, 0.36]	0.48 [0.00, 0.92]
lr3e-05_wd0.005_h2048_d3	0.66 [0.63, 0.75]	0.43 [0.30, 0.61]	0.38 [0.32, 0.44]	0.57 [0.50, 0.64]	0.14 [0.00, 0.25]	0.39 [0.00, 0.80]	0.74 [0.33, 1.00]	0.28 [0.21, 0.37]	0.71 [0.00, 1.00]
lr3e-05_wd0.005_h1024_d4	0.66 [0.60, 0.76]	0.40 [0.28, 0.57]	0.35 [0.35, 0.37]	0.52 [0.50, 0.53]	0.05 [0.00, 0.08]	0.18 [0.00, 0.37]	0.86 [0.69, 1.00]	0.22 [0.21, 0.24]	0.70 [0.00, 1.00]
lr3e-05_wd0.005_h2048_d4	0.67 [0.65, 0.70]	0.39 [0.33, 0.53]	0.41 [0.35, 0.44]	0.60 [0.50, 0.64]	0.18 [0.00, 0.28]	0.43 [0.00, 0.83]	0.76 [0.46, 1.00]	0.31 [0.21, 0.43]	0.71 [0.00, 0.94]
lr3e-05_wd0.01_h1024_d3	0.67 [0.61, 0.74]	0.40 [0.32, 0.56]	0.36 [0.31, 0.41]	0.51 [0.46, 0.60]	0.01 [-0.11, 0.17]	0.09 [0.00, 0.35]	0.94 [0.83, 1.00]	0.22 [0.19, 0.27]	0.31 [0.00, 0.89]
lr3e-05_wd0.01_h2048_d3	0.68 [0.64, 0.72]	0.42 [0.32, 0.55]	0.31 [0.00, 0.42]	0.54 [0.47, 0.64]	0.05 [-0.12, 0.22]	0.39 [0.00, 0.93]	0.69 [0.00, 1.00]	0.20 [0.00, 0.31]	0.50 [0.00, 0.88]
lr3e-05_wd0.01_h1024_d4	0.66 [0.64, 0.70]	0.40 [0.31, 0.45]	0.37 [0.35, 0.39]	0.53 [0.50, 0.60]	0.06 [0.00, 0.17]	0.18 [0.00, 0.46]	0.89 [0.69, 1.00]	0.23 [0.21, 0.26]	0.34 [0.00, 0.88]
lr3e-05_wd0.01_h2048_d4	0.67 [0.63, 0.72]	0.38 [0.34, 0.44]	0.36 [0.30, 0.39]	0.55 [0.50, 0.60]	0.10 [0.00, 0.18]	0.33 [0.00, 0.91]	0.77 [0.23, 1.00]	0.27 [0.21, 0.43]	0.51 [0.00, 0.88]
lr1e-05_wd0.0_h256_d1	0.66 [0.60, 0.77]	0.42 [0.32, 0.55]	0.36 [0.32, 0.40]	0.52 [0.47, 0.58]	0.03 [-0.07, 0.17]	0.17 [0.00, 0.48]	0.87 [0.62, 1.00]	0.23 [0.20, 0.26]	0.49 [0.00, 0.92]
lr1e-05_wd0.0_h400_d1	0.64 [0.58, 0.74]	0.37 [0.27, 0.45]	0.35 [0.33, 0.37]	0.51 [0.50, 0.51]	0.05 [0.00, 0.07]	0.10 [0.00, 0.41]	0.92 [0.62, 1.00]	0.22 [0.21, 0.23]	0.76 [0.00, 1.00]
lr1e-05_wd0.0_h512_d1	0.66 [0.60, 0.73]	0.43 [0.29, 0.56]	0.33 [0.29, 0.36]	0.49 [0.44, 0.51]	-0.03 [-0.20, 0.07]	0.11 [0.00, 0.50]	0.86 [0.46, 1.00]	0.21 [0.19, 0.22]	0.45 [0.00, 1.00]
lr1e-05_wd0.0_h256_d2	0.64 [0.61, 0.71]	0.40 [0.27, 0.49]	0.33 [0.28, 0.37]	0.49 [0.43, 0.52]	-0.03 [-0.27, 0.07]	0.16 [0.00, 0.74]	0.83 [0.31, 1.00]	0.22 [0.18, 0.25]	0.42 [0.00, 1.00]
lr1e-05_wd0.0_h400_d2	0.65 [0.60, 0.70]	0.41 [0.28, 0.51]	0.28 [0.00, 0.37]	0.50 [0.47, 0.51]	0.00 [-0.07, 0.07]	0.23 [0.00, 1.00]	0.77 [0.00, 1.00]	0.17 [0.00, 0.22]	0.50 [0.00, 1.00]
lr1e-05_wd0.0_h512_d2	0.64 [0.61, 0.75]	0.39 [0.28, 0.57]	0.28 [0.00, 0.36]	0.50 [0.48, 0.50]	-0.01 [-0.04, 0.00]	0.23 [0.00, 1.00]	0.77 [0.00, 1.00]	0.17 [0.00, 0.22]	0.31 [0.00, 0.78]
lr1e-05_wd0.0001_h256_d1	0.62 [0.57, 0.67]	0.36 [0.26, 0.43]	0.28 [0.00, 0.39]	0.51 [0.47, 0.55]	0.01 [-0.14, 0.16]	0.35 [0.02, 0.96]	0.67 [0.00, 1.00]	0.18 [0.00, 0.26]	0.78 [0.50, 1.00]
lr1e-05_wd0.0001_h400_d1	0.65 [0.59, 0.73]	0.44 [0.27, 0.51]	0.34 [0.29, 0.36]	0.49 [0.44, 0.53]	-0.01 [-0.20, 0.12]	0.14 [0.00, 0.52]	0.84 [0.46, 1.00]	0.21 [0.19, 0.22]	0.61 [0.00, 1.00]
lr1e-05_wd0.0001_h512_d1	0.65 [0.61, 0.71]	0.41 [0.30, 0.48]	0.33 [0.27, 0.37]	0.51 [0.49, 0.51]	0.03 [-0.02, 0.07]	0.18 [0.00, 0.72]	0.83 [0.31, 1.00]	0.22 [0.20, 0.24]	0.71 [0.00, 1.00]
lr1e-05_wd0.0001_h256_d2	0.65 [0.57, 0.73]	0.38 [0.30, 0.45]	0.28 [0.00, 0.36]	0.50 [0.48, 0.50]	-0.01 [-0.07, 0.00]	0.21 [0.00, 1.00]	0.78 [0.00, 1.00]	0.17 [0.00, 0.22]	0.29 [0.00, 0.78]
lr1e-05_wd0.0001_h400_d2	0.64 [0.60, 0.76]	0.38 [0.29, 0.43]	0.29 [0.00, 0.37]	0.51 [0.49, 0.54]	0.03 [-0.07, 0.14]	0.28 [0.00, 0.98]	0.75 [0.00, 1.00]	0.18 [0.00, 0.23]	0.69 [0.00, 1.00]
lr1e-05_wd0.0001_h512_d2	0.64 [0.61, 0.68]	0.38 [0.30, 0.47]	0.28 [0.00, 0.39]	0.49 [0.41, 0.55]	-0.03 [-0.25, 0.16]	0.23 [0.00, 0.98]	0.75 [0.00, 1.00]	0.17 [0.00, 0.24]	0.46 [0.00, 1.00]
lr1e-05_wd0.001_h256_d1	0.65 [0.58, 0.78]	0.41 [0.30, 0.46]	0.34 [0.32, 0.36]	0.49 [0.48, 0.51]	-0.02 [-0.07, 0.01]	0.11 [0.00, 0.48]	0.88 [0.54, 1.00]	0.21 [0.20, 0.23]	0.42 [0.00, 0.79]
lr1e-05_wd0.001_h400_d1	0.65 [0.54, 0.77]	0.39 [0.25, 0.50]	0.24 [0.00, 0.40]	0.51 [0.47, 0.58]	0.00 [-0.13, 0.20]	0.42 [0.00, 0.98]	0.60 [0.00, 1.00]	0.18 [0.00, 0.25]	0.61 [0.00, 1.00]
lr1e-05_wd0.001_h512_d1	0.64 [0.59, 0.68]	0.39 [0.33, 0.44]	0.35 [0.31, 0.37]	0.50 [0.46, 0.56]	-0.01 [-0.11, 0.11]	0.13 [0.00, 0.43]	0.87 [0.69, 1.00]	0.22 [0.19, 0.26]	0.45 [0.00, 0.83]
lr1e-05_wd0.001_h256_d2	0.66 [0.60, 0.74]	0.40 [0.29, 0.54]	0.35 [0.32, 0.37]	0.52 [0.50, 0.57]	0.04 [0.00, 0.14]	0.17 [0.00, 0.83]	0.86 [0.31, 1.00]	0.24 [0.21, 0.33]	0.36 [0.00, 1.00]
lr1e-05_wd0.001_h400_d2	0.64 [0.60, 0.73]	0.40 [0.31, 0.50]	0.35 [0.34, 0.36]	0.51 [0.50, 0.56]	0.02 [0.00, 0.10]	0.12 [0.00, 0.59]	0.91 [0.54, 1.00]	0.23 [0.21, 0.27]	0.16 [0.00, 0.82]
lr1e-05_wd0.001_h512_d2	0.67 [0.59, 0.76]	0.43 [0.34, 0.55]	0.30 [0.13, 0.36]	0.51 [0.49, 0.55]	0.02 [-0.03, 0.08]	0.30 [0.00, 0.96]	0.72 [0.08, 1.00]	0.24 [0.20, 0.33]	0.47 [0.00, 0.81]
lr3e-05_wd0.0_h256_d1	0.67 [0.63, 0.72]	0.42 [0.31, 0.48]	0.37 [0.32, 0.43]	0.55 [0.46, 0.65]	0.05 [-0.26, 0.24]	0.27 [0.00, 0.54]	0.83 [0.69, 0.92]	0.25 [0.20, 0.30]	0.69 [0.00, 0.91]
lr3e-05_wd0.0_h400_d1	0.69 [0.65, 0.75]	0.46 [0.32, 0.61]	0.38 [0.35, 0.43]	0.56 [0.50, 0.62]	0.12 [0.00, 0.23]	0.24 [0.00, 0.39]	0.89 [0.75, 1.00]	0.25 [0.22, 0.28]	0.71 [0.00, 0.94]
lr3e-05_wd0.0_h512_d1	0.66 [0.64, 0.70]	0.39 [0.32, 0.46]	0.39 [0.37, 0.43]	0.57 [0.53, 0.63]	0.14 [0.09, 0.22]	0.23 [0.07, 0.41]	0.90 [0.83, 1.00]	0.25 [0.23, 0.29]	0.91 [0.88, 1.00]
lr3e-05_wd0.0_h256_d2	0.67 [0.62, 0.74]	0.43 [0.31, 0.53]	0.35 [0.34, 0.36]	0.51 [0.50, 0.54]	0.03 [0.00, 0.06]	0.17 [0.00, 0.46]	0.86 [0.62, 1.00]	0.22 [0.21, 0.24]	0.49 [0.00, 0.82]
lr3e-05_wd0.0_h400_d2	0.67 [0.65, 0.75]	0.42 [0.31, 0.54]	0.39 [0.35, 0.47]	0.56 [0.50, 0.68]	0.11 [0.00, 0.31]	0.20 [0.00, 0.43]	0.92 [0.75, 1.00]	0.25 [0.21, 0.32]	0.54 [0.00, 0.95]
lr3e-05_wd0.0_h512_d2	0.67 [0.63, 0.73]	0.39 [0.34, 0.43]	0.40 [0.37, 0.45]	0.59 [0.51, 0.67]	0.18 [0.07, 0.27]	0.32 [0.02, 0.63]	0.86 [0.62, 1.00]	0.27 [0.22, 0.32]	0.93 [0.85, 1.00]
lr3e-05_wd0.0001_h256_d1	0.66 [0.64, 0.71]	0.41 [0.35, 0.46]	0.36 [0.32, 0.38]	0.52 [0.46, 0.55]	0.02 [-0.26, 0.10]	0.13 [0.00, 0.41]	0.91 [0.69, 1.00]	0.22 [0.19, 0.25]	0.74 [0.00, 1.00]
lr3e-05_wd0.0001_h400_d1	0.67 [0.62, 0.72]	0.44 [0.31, 0.56]	0.39 [0.34, 0.44]	0.58 [0.50, 0.65]	0.14 [0.00, 0.26]	0.33 [0.00, 0.52]	0.83 [0.67, 1.00]	0.26 [0.21, 0.29]	0.70 [0.00, 0.94]
lr3e-05_wd0.0001_h512_d1	0.65 [0.62, 0.68]	0.37 [0.32, 0.43]	0.36 [0.33, 0.41]	0.53 [0.47, 0.60]	0.06 [-0.09, 0.17]	0.24 [0.02, 0.37]	0.83 [0.69, 1.00]	0.23 [0.21, 0.27]	0.84 [0.67, 1.00]
lr3e-05_wd0.0001_h256_d2	0.67 [0.62, 0.72]	0.39 [0.30, 0.52]	0.38 [0.34, 0.43]	0.55 [0.50, 0.62]	0.10 [0.00, 0.23]	0.18 [0.00, 0.41]	0.92 [0.77, 1.00]	0.24 [0.21, 0.28]	0.54 [0.00, 0.94]
lr3e-05_wd0.0001_h400_d2	0.66 [0.61, 0.71]	0.39 [0.32, 0.44]	0.37 [0.36, 0.40]	0.54 [0.50, 0.61]	0.09 [0.00, 0.19]	0.20 [0.00, 0.48]	0.89 [0.69, 1.00]	0.24 [0.22, 0.27]	0.74 [0.00, 1.00]
lr3e-05_wd0.0001_h512_d2	0.66 [0.64, 0.72]	0.43 [0.34, 0.50]	0.38 [0.33, 0.44]	0.56 [0.50, 0.64]	0.11 [0.00, 0.23]	0.33 [0.00, 0.59]	0.79 [0.62, 1.00]	0.26 [0.22, 0.32]	0.71 [0.00, 1.00]
lr3e-05_wd0.001_h256_d1	0.67 [0.62, 0.70]	0.43 [0.31, 0.50]	0.37 [0.33, 0.43]	0.54 [0.48, 0.63]	0.06 [-0.07, 0.21]	0.24 [0.02, 0.57]	0.83 [0.69, 1.00]	0.24 [0.20, 0.31]	0.83 [0.67, 1.00]
lr3e-05_wd0.001_h400_d1	0.69 [0.62, 0.80]	0.43 [0.31, 0.50]	0.37 [0.35, 0.40]	0.54 [0.50, 0.62]	0.08 [0.00, 0.19]	0.23 [0.00, 0.57]	0.86 [0.67, 1.00]	0.24 [0.21, 0.29]	0.51 [0.00, 0.87]
lr3e-05_wd0.001_h512_d1	0.66 [0.63, 0.68]	0.41 [0.34, 0.47]	0.39 [0.32, 0.47]	0.57 [0.46, 0.68]	0.08 [-0.26, 0.30]	0.37 [0.00, 0.69]	0.78 [0.54, 1.00]	0.28 [0.19, 0.36]	0.52 [0.00, 0.89]
lr3e-05_wd0.001_h256_d2	0.65 [0.63, 0.68]	0.39 [0.31, 0.45]	0.36 [0.33, 0.38]	0.52 [0.50, 0.57]	0.05 [0.00, 0.12]	0.16 [0.00, 0.37]	0.89 [0.75, 1.00]	0.23 [0.21, 0.26]	0.70 [0.00, 1.00]
lr3e-05_wd0.001_h400_d2	0.67 [0.63, 0.75]	0.42 [0.32, 0.51]	0.30 [0.00, 0.46]	0.54 [0.50, 0.66]	0.08 [0.00, 0.27]	0.36 [0.00, 1.00]	0.72 [0.00, 1.00]	0.19 [0.00, 0.31]	0.70 [0.00, 1.00]
lr3e-05_wd0.001_h512_d2	0.66 [0.61, 0.74]	0.39 [0.32, 0.45]	0.40 [0.35, 0.53]	0.57 [0.50, 0.72]	0.13 [0.00, 0.39]	0.23 [0.00, 0.78]	0.90 [0.67, 1.00]	0.27 [0.21, 0.44]	0.56 [0.00, 1.00]
lr0.0001_wd0.005_h256_d1	0.67 [0.64, 0.69]	0.41 [0.33, 0.49]	0.38 [0.32, 0.44]	0.57 [0.48, 0.64]	0.12 [-0.03, 0.24]	0.29 [0.20, 0.43]	0.84 [0.75, 0.92]	0.25 [0.20, 0.30]	0.86 [0.77, 0.91]
lr0.0001_wd0.005_h400_d1	0.67 [0.64, 0.73]	0.43 [0.34, 0.51]	0.40 [0.36, 0.45]	0.59 [0.50, 0.66]	0.15 [0.00, 0.27]	0.40 [0.00, 0.65]	0.78 [0.54, 1.00]	0.27 [0.22, 0.32]	0.70 [0.00, 0.90]
lr0.0001_wd0.005_h512_d1	0.68 [0.65, 0.71]	0.43 [0.33, 0.56]	0.40 [0.34, 0.50]	0.59 [0.50, 0.69]	0.17 [0.00, 0.33]	0.35 [0.00, 0.70]	0.83 [0.54, 1.00]	0.28 [0.21, 0.39]	0.73 [0.00, 1.00]
lr0.0001_wd0.005_h256_d2	0.65 [0.60, 0.72]	0.38 [0.32, 0.44]	0.37 [0.20, 0.52]	0.57 [0.50, 0.71]	0.12 [0.00, 0.37]	0.42 [0.00, 0.89]	0.72 [0.15, 1.00]	0.28 [0.21, 0.42]	0.51 [0.00, 0.90]
lr0.0001_wd0.005_h400_d2	0.66 [0.62, 0.70]	0.38 [0.33, 0.45]	0.37 [0.29, 0.45]	0.57 [0.47, 0.65]	0.12 [-0.13, 0.33]	0.50 [0.02, 0.89]	0.64 [0.23, 0.92]	0.32 [0.21, 0.50]	0.78 [0.50, 0.86]
lr0.0001_wd0.005_h512_d2	0.64 [0.60, 0.73]	0.39 [0.32, 0.44]	0.36 [0.30, 0.42]	0.55 [0.49, 0.62]	0.10 [-0.02, 0.21]	0.37 [0.00, 0.89]	0.72 [0.25, 1.00]	0.27 [0.20, 0.38]	0.67 [0.00, 0.90]
lr0.0001_wd0.01_h256_d1	0.66 [0.62, 0.68]	0.38 [0.33, 0.43]	0.42 [0.36, 0.52]	0.61 [0.54, 0.71]	0.20 [0.08, 0.37]	0.47 [0.09, 0.76]	0.75 [0.54, 1.00]	0.31 [0.24, 0.42]	0.90 [0.81, 1.00]
lr0.0001_wd0.01_h400_d1	0.67 [0.62, 0.72]	0.44 [0.35, 0.57]	0.38 [0.33, 0.44]	0.56 [0.47, 0.66]	0.10 [-0.09, 0.26]	0.26 [0.07, 0.57]	0.86 [0.75, 0.92]	0.25 [0.21, 0.31]	0.82 [0.67, 0.90]

Supplementary Table 15: Grid search results on the classification task on SYSMH external validation dataset (choosing 50% as the threshold for clinical variables selection). Reported metrics are AUC, AUPRC, F1, Balanced Accuracy, MCC, Specificity, Sensitivity, PPV, and NPV.

Config	AUC	AUPRC	F1	Balanced_Accuracy	MCC	Specificity	Sensitivity	PPV	NPV
lr0.0001_wd0.01_h512_d1	0.66 [0.63, 0.68]	0.40 [0.30, 0.54]	0.38 [0.33, 0.42]	0.58 [0.47, 0.64]	0.11 [-0.14, 0.22]	0.45 [0.02, 0.65]	0.70 [0.46, 0.92]	0.27 [0.20, 0.30]	0.78 [0.50, 0.89]
lr0.0001_wd0.01_h256_d2	0.67 [0.62, 0.71]	0.40 [0.35, 0.43]	0.37 [0.34, 0.42]	0.55 [0.50, 0.61]	0.10 [0.00, 0.22]	0.31 [0.00, 0.80]	0.79 [0.38, 1.00]	0.26 [0.22, 0.36]	0.68 [0.00, 0.93]
lr0.0001_wd0.01_h400_d2	0.67 [0.65, 0.76]	0.40 [0.31, 0.46]	0.38 [0.29, 0.47]	0.57 [0.50, 0.69]	0.14 [0.00, 0.31]	0.36 [0.00, 0.89]	0.78 [0.23, 1.00]	0.28 [0.22, 0.38]	0.73 [0.00, 1.00]
lr0.0001_wd0.01_h512_d2	0.65 [0.63, 0.69]	0.37 [0.30, 0.49]	0.38 [0.25, 0.47]	0.58 [0.50, 0.67]	0.15 [0.00, 0.30]	0.51 [0.00, 0.83]	0.66 [0.23, 1.00]	0.29 [0.21, 0.39]	0.69 [0.00, 0.94]
lr0.0003_wd0.005_h256_d1	0.65 [0.62, 0.68]	0.37 [0.29, 0.49]	0.41 [0.36, 0.44]	0.61 [0.57, 0.64]	0.19 [0.13, 0.25]	0.46 [0.31, 0.67]	0.76 [0.50, 0.92]	0.28 [0.24, 0.32]	0.89 [0.84, 0.94]
lr0.0003_wd0.005_h400_d1	0.67 [0.60, 0.71]	0.36 [0.32, 0.38]	0.43 [0.35, 0.51]	0.62 [0.53, 0.70]	0.22 [0.06, 0.35]	0.49 [0.13, 0.74]	0.76 [0.50, 0.92]	0.31 [0.22, 0.41]	0.88 [0.85, 0.91]
lr0.0003_wd0.005_h512_d1	0.67 [0.64, 0.72]	0.40 [0.36, 0.42]	0.41 [0.37, 0.46]	0.60 [0.55, 0.67]	0.18 [0.09, 0.28]	0.38 [0.13, 0.67]	0.82 [0.67, 1.00]	0.28 [0.24, 0.35]	0.90 [0.83, 1.00]
lr0.0003_wd0.005_h256_d2	0.66 [0.63, 0.72]	0.36 [0.30, 0.43]	0.32 [0.11, 0.43]	0.53 [0.48, 0.62]	0.05 [-0.04, 0.25]	0.29 [0.00, 0.89]	0.77 [0.08, 1.00]	0.22 [0.17, 0.27]	0.52 [0.00, 1.00]
lr0.0003_wd0.005_h400_d2	0.67 [0.60, 0.73]	0.39 [0.32, 0.50]	0.30 [0.00, 0.43]	0.53 [0.46, 0.63]	0.00 [-0.26, 0.22]	0.34 [0.00, 0.96]	0.72 [0.00, 0.92]	0.19 [0.00, 0.29]	0.67 [0.00, 0.92]
lr0.0003_wd0.005_h512_d2	0.67 [0.62, 0.76]	0.40 [0.31, 0.51]	0.37 [0.30, 0.43]	0.57 [0.50, 0.63]	0.15 [0.01, 0.22]	0.37 [0.11, 0.91]	0.77 [0.23, 1.00]	0.28 [0.21, 0.43]	0.88 [0.80, 1.00]
lr0.0003_wd0.01_h256_d1	0.67 [0.63, 0.72]	0.40 [0.37, 0.43]	0.41 [0.35, 0.48]	0.60 [0.52, 0.69]	0.19 [0.03, 0.32]	0.36 [0.17, 0.72]	0.84 [0.67, 1.00]	0.28 [0.23, 0.38]	0.90 [0.80, 1.00]
lr0.0003_wd0.01_h400_d1	0.69 [0.66, 0.79]	0.41 [0.34, 0.52]	0.39 [0.33, 0.43]	0.59 [0.55, 0.63]	0.16 [0.08, 0.23]	0.54 [0.24, 0.67]	0.65 [0.46, 0.92]	0.29 [0.24, 0.33]	0.86 [0.81, 0.92]
lr0.0003_wd0.01_h512_d1	0.67 [0.60, 0.76]	0.39 [0.35, 0.45]	0.41 [0.30, 0.58]	0.63 [0.54, 0.79]	0.22 [0.06, 0.49]	0.58 [0.43, 0.78]	0.68 [0.31, 1.00]	0.30 [0.24, 0.41]	0.88 [0.80, 1.00]
lr0.0003_wd0.01_h256_d2	0.69 [0.67, 0.73]	0.43 [0.34, 0.52]	0.33 [0.29, 0.37]	0.54 [0.50, 0.59]	0.09 [0.00, 0.20]	0.53 [0.00, 0.87]	0.56 [0.25, 1.00]	0.29 [0.21, 0.40]	0.65 [0.00, 0.83]
lr0.0003_wd0.01_h400_d2	0.70 [0.63, 0.76]	0.43 [0.38, 0.48]	0.40 [0.35, 0.47]	0.60 [0.53, 0.69]	0.19 [0.05, 0.31]	0.47 [0.20, 0.83]	0.73 [0.38, 1.00]	0.30 [0.24, 0.38]	0.88 [0.81, 1.00]
lr0.0003_wd0.01_h512_d2	0.69 [0.62, 0.76]	0.41 [0.30, 0.53]	0.30 [0.12, 0.41]	0.54 [0.50, 0.60]	0.09 [0.00, 0.20]	0.43 [0.00, 0.93]	0.65 [0.08, 1.00]	0.24 [0.21, 0.27]	0.70 [0.00, 1.00]
lr0.001_wd0.005_h256_d1	0.68 [0.64, 0.74]	0.40 [0.33, 0.51]	0.40 [0.30, 0.49]	0.60 [0.51, 0.68]	0.19 [0.04, 0.31]	0.51 [0.11, 0.89]	0.69 [0.25, 0.92]	0.31 [0.22, 0.38]	0.86 [0.82, 0.89]
lr0.001_wd0.005_h400_d1	0.67 [0.65, 0.74]	0.40 [0.31, 0.50]	0.39 [0.32, 0.46]	0.60 [0.51, 0.67]	0.17 [0.02, 0.27]	0.60 [0.36, 0.85]	0.59 [0.38, 0.75]	0.31 [0.22, 0.42]	0.84 [0.79, 0.90]
lr0.001_wd0.005_h512_d1	0.70 [0.64, 0.79]	0.42 [0.34, 0.59]	0.38 [0.33, 0.42]	0.58 [0.52, 0.64]	0.14 [0.05, 0.22]	0.48 [0.04, 0.70]	0.67 [0.46, 1.00]	0.27 [0.22, 0.31]	0.87 [0.80, 1.00]
lr0.001_wd0.005_h256_d2	0.69 [0.63, 0.74]	0.39 [0.36, 0.44]	0.38 [0.31, 0.41]	0.57 [0.50, 0.62]	0.13 [0.00, 0.22]	0.31 [0.00, 0.70]	0.83 [0.38, 1.00]	0.25 [0.22, 0.26]	0.72 [0.00, 0.94]
lr0.001_wd0.005_h400_d2	0.68 [0.65, 0.71]	0.40 [0.34, 0.52]	0.34 [0.17, 0.44]	0.57 [0.49, 0.65]	0.12 [-0.02, 0.28]	0.61 [0.28, 0.83]	0.53 [0.15, 0.92]	0.27 [0.20, 0.40]	0.84 [0.78, 0.93]
lr0.001_wd0.005_h512_d2	0.68 [0.68, 0.70]	0.46 [0.34, 0.56]	0.40 [0.33, 0.44]	0.59 [0.47, 0.65]	0.14 [-0.14, 0.28]	0.46 [0.02, 0.80]	0.71 [0.50, 0.92]	0.30 [0.20, 0.40]	0.80 [0.50, 0.93]
lr0.001_wd0.01_h256_d1	0.66 [0.63, 0.70]	0.39 [0.34, 0.49]	0.36 [0.15, 0.44]	0.59 [0.54, 0.66]	0.19 [0.09, 0.26]	0.57 [0.24, 1.00]	0.61 [0.08, 0.85]	0.43 [0.24, 1.00]	0.85 [0.80, 0.88]
lr0.001_wd0.01_h400_d1	0.67 [0.65, 0.70]	0.42 [0.35, 0.56]	0.39 [0.33, 0.45]	0.58 [0.48, 0.65]	0.15 [-0.04, 0.30]	0.46 [0.13, 0.83]	0.70 [0.38, 1.00]	0.29 [0.20, 0.38]	0.85 [0.75, 1.00]
lr0.001_wd0.01_h512_d1	0.67 [0.59, 0.71]	0.44 [0.39, 0.52]	0.43 [0.38, 0.48]	0.63 [0.61, 0.66]	0.25 [0.19, 0.37]	0.72 [0.37, 0.91]	0.54 [0.38, 0.85]	0.39 [0.28, 0.56]	0.86 [0.83, 0.89]
lr0.001_wd0.01_h256_d2	0.67 [0.63, 0.72]	0.36 [0.33, 0.39]	0.42 [0.35, 0.48]	0.61 [0.50, 0.67]	0.21 [0.00, 0.32]	0.49 [0.00, 0.85]	0.73 [0.42, 1.00]	0.33 [0.21, 0.44]	0.70 [0.00, 0.92]
lr0.001_wd0.01_h400_d2	0.67 [0.63, 0.74]	0.41 [0.31, 0.46]	0.34 [0.17, 0.47]	0.54 [0.48, 0.68]	0.09 [-0.04, 0.34]	0.34 [0.02, 0.80]	0.75 [0.15, 1.00]	0.23 [0.18, 0.31]	0.88 [0.77, 1.00]
lr0.001_wd0.01_h512_d2	0.68 [0.63, 0.75]	0.38 [0.32, 0.45]	0.36 [0.26, 0.44]	0.56 [0.51, 0.64]	0.13 [0.07, 0.24]	0.36 [0.02, 0.85]	0.77 [0.23, 1.00]	0.26 [0.21, 0.30]	0.89 [0.80, 1.00]
lr0.0001_wd0.0_h1024_d1	0.68 [0.66, 0.70]	0.42 [0.34, 0.46]	0.41 [0.38, 0.50]	0.60 [0.56, 0.68]	0.20 [0.13, 0.35]	0.40 [0.11, 0.83]	0.80 [0.54, 1.00]	0.30 [0.23, 0.47]	0.90 [0.86, 1.00]
lr0.0001_wd0.0_h2048_d1	0.70 [0.65, 0.72]	0.43 [0.36, 0.50]	0.40 [0.34, 0.50]	0.58 [0.50, 0.68]	0.15 [0.00, 0.35]	0.32 [0.00, 0.83]	0.84 [0.54, 1.00]	0.29 [0.21, 0.47]	0.70 [0.00, 0.93]
lr0.0001_wd0.0_h1024_d2	0.69 [0.65, 0.73]	0.39 [0.35, 0.48]	0.36 [0.31, 0.44]	0.53 [0.47, 0.64]	0.04 [-0.14, 0.24]	0.25 [0.00, 0.70]	0.81 [0.38, 1.00]	0.24 [0.20, 0.30]	0.60 [0.00, 0.91]
lr0.0001_wd0.0_h2048_d2	0.68 [0.63, 0.76]	0.43 [0.31, 0.49]	0.38 [0.35, 0.42]	0.56 [0.50, 0.62]	0.09 [0.00, 0.21]	0.30 [0.00, 0.63]	0.81 [0.62, 1.00]	0.25 [0.21, 0.32]	0.51 [0.00, 0.87]
lr0.0001_wd0.0001_h1024_d1	0.67 [0.65, 0.70]	0.40 [0.33, 0.50]	0.43 [0.33, 0.47]	0.63 [0.53, 0.69]	0.22 [0.05, 0.31]	0.57 [0.46, 0.72]	0.70 [0.54, 0.85]	0.31 [0.24, 0.38]	0.87 [0.80, 0.91]
lr0.0001_wd0.0001_h2048_d1	0.67 [0.65, 0.70]	0.40 [0.36, 0.46]	0.38 [0.22, 0.54]	0.59 [0.54, 0.73]	0.18 [0.08, 0.39]	0.51 [0.17, 0.91]	0.68 [0.17, 0.92]	0.30 [0.22, 0.42]	0.87 [0.80, 0.93]
lr0.0001_wd0.0001_h1024_d2	0.67 [0.65, 0.69]	0.37 [0.32, 0.43]	0.37 [0.20, 0.50]	0.58 [0.50, 0.72]	0.15 [0.00, 0.38]	0.43 [0.00, 0.89]	0.73 [0.15, 1.00]	0.28 [0.21, 0.33]	0.70 [0.00, 1.00]
lr0.0001_wd0.0001_h2048_d2	0.68 [0.66, 0.71]	0.43 [0.33, 0.49]	0.31 [0.25, 0.36]	0.50 [0.45, 0.53]	-0.01 [-0.15, 0.06]	0.29 [0.00, 0.83]	0.71 [0.23, 1.00]	0.22 [0.19, 0.27]	0.44 [0.00, 0.79]
lr0.0001_wd0.001_h1024_d1	0.68 [0.65, 0.74]	0.42 [0.35, 0.47]	0.43 [0.38, 0.48]	0.63 [0.56, 0.69]	0.23 [0.16, 0.32]	0.41 [0.11, 0.72]	0.84 [0.67, 1.00]	0.30 [0.23, 0.38]	0.92 [0.88, 1.00]
lr0.0001_wd0.001_h2048_d1	0.68 [0.64, 0.71]	0.42 [0.35, 0.48]	0.40 [0.35, 0.44]	0.59 [0.50, 0.64]	0.16 [0.00, 0.25]	0.45 [0.00, 0.72]	0.73 [0.54, 1.00]	0.29 [0.21, 0.35]	0.70 [0.00, 0.94]
lr0.0001_wd0.001_h1024_d2	0.67 [0.64, 0.73]	0.39 [0.32, 0.46]	0.42 [0.35, 0.47]	0.62 [0.50, 0.67]	0.21 [0.00, 0.28]	0.34 [0.00, 0.59]	0.89 [0.75, 1.00]	0.28 [0.21, 0.33]	0.75 [0.00, 1.00]
lr0.0001_wd0.001_h2048_d2	0.65 [0.61, 0.69]	0.42 [0.39, 0.50]	0.40 [0.36, 0.44]	0.60 [0.55, 0.64]	0.17 [0.09, 0.23]	0.53 [0.30, 0.78]	0.67 [0.46, 0.85]	0.29 [0.24, 0.38]	0.85 [0.84, 0.88]
lr0.0003_wd0.0_h1024_d1	0.67 [0.63, 0.70]	0.40 [0.32, 0.46]	0.42 [0.36, 0.49]	0.62 [0.54, 0.68]	0.21 [0.09, 0.31]	0.49 [0.09, 0.72]	0.74 [0.58, 1.00]	0.31 [0.23, 0.38]	0.89 [0.86, 1.00]
lr0.0003_wd0.0_h2048_d1	0.68 [0.63, 0.73]	0.39 [0.30, 0.43]	0.43 [0.34, 0.50]	0.62 [0.50, 0.69]	0.21 [0.01, 0.33]	0.43 [0.09, 0.76]	0.81 [0.62, 0.92]	0.30 [0.21, 0.42]	0.88 [0.80, 0.92]
lr0.0003_wd0.0_h1024_d2	0.69 [0.63, 0.74]	0.38 [0.30, 0.44]	0.39 [0.33, 0.42]	0.59 [0.57, 0.63]	0.19 [0.14, 0.27]	0.48 [0.13, 0.87]	0.70 [0.33, 1.00]	0.31 [0.25, 0.45]	0.90 [0.83, 1.00]
lr0.0003_wd0.0_h2048_d2	0.70 [0.65, 0.75]	0.39 [0.31, 0.49]	0.39 [0.35, 0.44]	0.59 [0.50, 0.64]	0.18 [0.00, 0.28]	0.47 [0.00, 0.89]	0.71 [0.31, 1.00]	0.31 [0.21, 0.44]	0.71 [0.00, 1.00]
lr0.0003_wd0.0001_h1024_d1	0.66 [0.62, 0.74]	0.40 [0.30, 0.61]	0.40 [0.33, 0.47]	0.60 [0.47, 0.67]	0.16 [-0.14, 0.28]	0.49 [0.02, 0.85]	0.71 [0.33, 0.92]	0.31 [0.20, 0.36]	0.80 [0.50, 0.93]
lr0.0003_wd0.0001_h2048_d1	0.67 [0.64, 0.71]	0.38 [0.31, 0.46]	0.40 [0.36, 0.46]	0.61 [0.57, 0.66]	0.19 [0.11, 0.27]	0.48 [0.22, 0.85]	0.73 [0.38, 0.92]	0.30 [0.23, 0.42]	0.88 [0.83, 0.92]
lr0.0003_wd0.0001_h1024_d2	0.69 [0.66, 0.73]	0.40 [0.35, 0.48]	0.36 [0.19, 0.47]	0.57 [0.50, 0.67]	0.12 [0.00, 0.29]	0.43 [0.00, 0.87]	0.70 [0.15, 1.00]	0.27 [0.21, 0.36]	0.69 [0.00, 0.91]
lr0.0003_wd0.0001_h2048_d2	0.66 [0.64, 0.69]	0.37 [0.33, 0.42]	0.34 [0.21, 0.42]	0.58 [0.53, 0.61]	0.16 [0.07, 0.24]	0.64 [0.22, 0.89]	0.52 [0.17, 1.00]	0.30 [0.27, 0.36]	0.86 [0.80, 1.00]
lr0.0003_wd0.001_h1024_d1	0.68 [0.64, 0.78]	0.44 [0.35, 0.50]	0.40 [0.38, 0.47]	0.60 [0.57, 0.68]	0.18 [0.12, 0.34]	0.40 [0.31, 0.54]	0.81 [0.67, 1.00]	0.27 [0.24, 0.31]	0.89 [0.85, 1.00]
lr0.0003_wd0.001_h2048_d1	0.67 [0.64, 0.70]	0.41 [0.33, 0.53]	0.38 [0.35, 0.46]	0.56 [0.50, 0.66]	0.11 [0.00, 0.27]	0.26 [0.00, 0.63]	0.86 [0.69, 1.00]	0.25 [0.22, 0.35]	0.72 [0.00, 1.00]
lr0.0003_wd0.001_h1024_d2	0.68 [0.65, 0.72]	0.39 [0.34, 0.42]	0.37 [0.18, 0.46]	0.59 [0.50, 0.66]	0.16 [-0.01, 0.27]	0.53 [0.13, 0.83]	0.65 [0.17, 1.00]	0.27 [0.20, 0.35]	0.88 [0.79, 1.00]
lr0.0003_wd0.001_h2048_d2	0.70 [0.67, 0.75]	0.43 [0.33, 0.57]	0.41 [0.35, 0.46]	0.60 [0.51, 0.66]	0.19 [0.07, 0.27]	0.46 [0.02, 0.78]	0.74 [0.42, 1.00]	0.30 [0.21, 0.37]	0.91 [0.84, 1.00]
lr0.001_wd0.0_h1024_d1	0.67 [0.64, 0.70]	0.39 [0.35, 0.48]	0.37 [0.34, 0.39]	0.58 [0.55, 0.61]	0.15 [0.09, 0.21]	0.55 [0.22, 0.83]	0.60 [0.38, 0.92]	0.30 [0.23, 0.38]	0.85 [0.83, 0.91]
lr0.001_wd0.0_h2048_d1	0.68 [0.66, 0.71]	0.38 [0.34, 0.46]	0.42 [0.34, 0.52]	0.62 [0.52, 0.71]	0.21 [0.03, 0.37]	0.57 [0.20, 0.76]	0.67 [0.38, 0.85]	0.32 [0.22, 0.42]	0.86 [0.81, 0.90]
lr0.001_wd0.0_h1024_d2	0.67 [0.64, 0.68]	0.42 [0.38, 0.55]	0.36 [0.22, 0.43]	0.59 [0.53, 0.64]	0.17 [0.05, 0.24]	0.69 [0.50, 0.93]	0.49 [0.15, 0.77]	0.33 [0.23, 0.40]	0.84 [0.80, 0.88]
lr0.001_wd0.0_h2048_d2	0.69 [0.63, 0.73]	0.40 [0.33, 0.45]	0.35 [0.26, 0.40]	0.56 [0.50, 0.61]	0.11 [0.00, 0.19]	0.51 [0.00, 0.85]	0.61 [0.23, 1.00]	0.27 [0.21, 0.30]	0.67 [0.00, 0.88]
lr0.001_wd0.0001_h1024_d1	0.69 [0.66, 0.73]	0.42 [0.32, 0.51]	0.45 [0.38, 0.51]	0.66 [0.57, 0.70]	0.26 [0.12, 0.35]	0.56 [0.42, 0.72]	0.75 [0.67, 0.92]	0.33 [0.26, 0.41]	0.89 [0.84, 0.95]
lr0.001_wd0.0001_h2048_d1	0.70 [0.65, 0.74]	0.44 [0.37, 0.53]	0.42 [0.34, 0.54]	0.61 [0.53, 0.70]	0.21 [0.05, 0.41]	0.41 [0.17, 0.87]	0.81 [0.54, 1.00]	0.31 [0.22, 0.54]	0.90 [0.83, 1.00]
lr0.001_wd0.0001_h1024_d2	0.71 [0.68, 0.76]	0.43 [0.36, 0.55]	0.41 [0.32, 0.47]	0.60 [0.49, 0.67]	0.18 [-0.01, 0.29]	0.47 [0.24, 0.74]	0.73 [0.54, 0.92]	0.29 [0.20, 0.37]	0.86 [0.79, 0.92]

Supplementary Table 16: Grid search results on the classification task on SYSMH external validation dataset (choosing 50% as the threshold for clinical variables selection). Reported metrics are AUC, AUPRC, F1, Balanced Accuracy, MCC, Specificity, Sensitivity, PPV, and NPV.

Config	AUC	AUPRC	F1	Balanced_Accuracy	MCC	Specificity	Sensitivity	PPV	NPV
lr0.001_wd0.0001_h2048_d2	0.71 [0.65, 0.75]	0.39 [0.33, 0.48]	0.43 [0.40, 0.50]	0.64 [0.60, 0.72]	0.26 [0.22, 0.38]	0.43 [0.20, 0.72]	0.84 [0.54, 1.00]	0.30 [0.25, 0.35]	0.94 [0.85, 1.00]
lr0.001_wd0.001_h1024_d1	0.66 [0.63, 0.69]	0.38 [0.30, 0.50]	0.37 [0.35, 0.41]	0.58 [0.54, 0.61]	0.14 [0.07, 0.18]	0.51 [0.26, 0.78]	0.64 [0.38, 0.92]	0.28 [0.24, 0.33]	0.85 [0.82, 0.92]
lr0.001_wd0.001_h2048_d1	0.67 [0.64, 0.75]	0.38 [0.34, 0.48]	0.40 [0.29, 0.54]	0.61 [0.53, 0.74]	0.19 [0.07, 0.40]	0.55 [0.24, 0.76]	0.67 [0.31, 0.92]	0.29 [0.26, 0.39]	0.87 [0.80, 0.94]
lr0.001_wd0.001_h1024_d2	0.68 [0.67, 0.69]	0.39 [0.33, 0.49]	0.36 [0.22, 0.45]	0.60 [0.54, 0.65]	0.20 [0.13, 0.26]	0.65 [0.29, 0.93]	0.55 [0.15, 0.92]	0.35 [0.26, 0.50]	0.86 [0.80, 0.93]
lr0.001_wd0.001_h2048_d2	0.67 [0.64, 0.69]	0.41 [0.32, 0.49]	0.33 [0.12, 0.50]	0.56 [0.46, 0.69]	0.08 [-0.26, 0.35]	0.56 [0.00, 0.93]	0.56 [0.08, 0.92]	0.29 [0.20, 0.44]	0.68 [0.00, 0.91]
lr0.0001_wd0.0_h256_d3	0.68 [0.65, 0.75]	0.44 [0.34, 0.52]	0.38 [0.27, 0.46]	0.58 [0.50, 0.67]	0.13 [0.00, 0.29]	0.39 [0.00, 0.87]	0.76 [0.23, 1.00]	0.28 [0.21, 0.33]	0.53 [0.00, 0.95]
lr0.0001_wd0.0_h400_d3	0.69 [0.64, 0.76]	0.41 [0.34, 0.48]	0.28 [0.00, 0.36]	0.53 [0.50, 0.59]	0.07 [0.00, 0.17]	0.52 [0.00, 1.00]	0.54 [0.00, 1.00]	0.22 [0.00, 0.36]	0.49 [0.00, 0.83]
lr0.0001_wd0.0_h512_d3	0.69 [0.64, 0.73]	0.42 [0.35, 0.52]	0.34 [0.19, 0.43]	0.54 [0.50, 0.65]	0.08 [0.00, 0.24]	0.32 [0.00, 0.87]	0.77 [0.15, 1.00]	0.25 [0.21, 0.30]	0.52 [0.00, 0.90]
lr0.0001_wd0.0_h256_d4	0.68 [0.62, 0.76]	0.42 [0.34, 0.61]	0.36 [0.30, 0.41]	0.56 [0.50, 0.63]	0.12 [0.00, 0.23]	0.38 [0.00, 0.91]	0.73 [0.23, 1.00]	0.29 [0.21, 0.43]	0.52 [0.00, 0.92]
lr0.0001_wd0.0_h400_d4	0.68 [0.66, 0.73]	0.38 [0.33, 0.41]	0.33 [0.11, 0.44]	0.54 [0.49, 0.66]	0.08 [-0.01, 0.26]	0.34 [0.00, 0.91]	0.75 [0.08, 1.00]	0.24 [0.20, 0.31]	0.52 [0.00, 0.91]
lr0.0001_wd0.0_h512_d4	0.69 [0.64, 0.75]	0.44 [0.37, 0.53]	0.41 [0.36, 0.50]	0.59 [0.50, 0.69]	0.16 [0.00, 0.33]	0.39 [0.00, 0.76]	0.79 [0.62, 1.00]	0.28 [0.22, 0.42]	0.70 [0.00, 0.92]
lr0.0001_wd0.0001_h256_d3	0.68 [0.64, 0.72]	0.42 [0.34, 0.53]	0.40 [0.35, 0.49]	0.58 [0.50, 0.69]	0.14 [0.00, 0.31]	0.30 [0.00, 0.70]	0.86 [0.58, 1.00]	0.27 [0.21, 0.36]	0.54 [0.00, 0.91]
lr0.0001_wd0.0001_h400_d3	0.68 [0.62, 0.73]	0.39 [0.34, 0.45]	0.35 [0.29, 0.40]	0.55 [0.50, 0.62]	0.09 [0.00, 0.21]	0.42 [0.00, 0.89]	0.67 [0.23, 1.00]	0.28 [0.21, 0.38]	0.66 [0.00, 0.88]
lr0.0001_wd0.0001_h512_d3	0.66 [0.63, 0.73]	0.41 [0.31, 0.54]	0.37 [0.33, 0.46]	0.56 [0.50, 0.68]	0.11 [0.00, 0.29]	0.32 [0.00, 0.85]	0.80 [0.31, 1.00]	0.27 [0.21, 0.36]	0.51 [0.00, 0.90]
lr0.0001_wd0.0001_h256_d4	0.67 [0.65, 0.70]	0.40 [0.32, 0.52]	0.31 [0.19, 0.42]	0.53 [0.50, 0.61]	0.05 [0.00, 0.22]	0.39 [0.00, 0.85]	0.66 [0.17, 1.00]	0.23 [0.21, 0.27]	0.50 [0.00, 0.93]
lr0.0001_wd0.0001_h400_d4	0.67 [0.64, 0.74]	0.39 [0.29, 0.45]	0.29 [0.00, 0.40]	0.51 [0.45, 0.58]	0.00 [-0.16, 0.17]	0.26 [0.00, 0.89]	0.75 [0.00, 1.00]	0.18 [0.00, 0.26]	0.50 [0.00, 0.92]
lr0.0001_wd0.0001_h512_d4	0.68 [0.61, 0.77]	0.41 [0.34, 0.50]	0.41 [0.35, 0.50]	0.58 [0.50, 0.70]	0.14 [0.00, 0.35]	0.33 [0.00, 0.74]	0.83 [0.54, 1.00]	0.29 [0.21, 0.40]	0.53 [0.00, 0.91]
lr0.0001_wd0.001_h256_d3	0.66 [0.62, 0.76]	0.39 [0.30, 0.61]	0.34 [0.24, 0.40]	0.52 [0.50, 0.58]	0.06 [0.00, 0.17]	0.21 [0.00, 0.78]	0.83 [0.25, 1.00]	0.23 [0.22, 0.26]	0.54 [0.00, 1.00]
lr0.0001_wd0.001_h400_d3	0.68 [0.64, 0.75]	0.39 [0.32, 0.47]	0.36 [0.29, 0.40]	0.54 [0.50, 0.59]	0.08 [0.00, 0.17]	0.29 [0.00, 0.76]	0.80 [0.31, 1.00]	0.24 [0.21, 0.27]	0.52 [0.00, 0.92]
lr0.0001_wd0.001_h512_d3	0.68 [0.64, 0.79]	0.44 [0.40, 0.48]	0.32 [0.20, 0.40]	0.51 [0.44, 0.59]	0.02 [-0.13, 0.16]	0.27 [0.00, 0.89]	0.75 [0.15, 1.00]	0.23 [0.18, 0.29]	0.47 [0.00, 0.88]
lr0.0001_wd0.001_h256_d4	0.67 [0.65, 0.71]	0.41 [0.33, 0.54]	0.38 [0.34, 0.42]	0.54 [0.50, 0.61]	0.08 [0.00, 0.22]	0.15 [0.00, 0.35]	0.94 [0.85, 1.00]	0.24 [0.21, 0.27]	0.53 [0.00, 0.93]
lr0.0001_wd0.001_h400_d4	0.68 [0.64, 0.75]	0.40 [0.34, 0.58]	0.40 [0.36, 0.45]	0.59 [0.50, 0.65]	0.17 [0.00, 0.25]	0.43 [0.00, 0.87]	0.76 [0.33, 1.00]	0.30 [0.22, 0.40]	0.71 [0.00, 0.93]
lr0.0001_wd0.001_h512_d4	0.67 [0.63, 0.75]	0.41 [0.32, 0.53]	0.36 [0.25, 0.45]	0.55 [0.50, 0.65]	0.08 [0.00, 0.25]	0.32 [0.00, 0.80]	0.77 [0.25, 1.00]	0.25 [0.21, 0.33]	0.51 [0.00, 0.89]
lr0.0003_wd0.0_h256_d3	0.69 [0.64, 0.73]	0.39 [0.36, 0.42]	0.41 [0.35, 0.50]	0.59 [0.50, 0.70]	0.15 [0.00, 0.33]	0.29 [0.00, 0.63]	0.89 [0.75, 1.00]	0.27 [0.21, 0.37]	0.54 [0.00, 0.94]
lr0.0003_wd0.0_h400_d3	0.68 [0.64, 0.70]	0.37 [0.35, 0.39]	0.37 [0.26, 0.43]	0.57 [0.50, 0.63]	0.14 [0.00, 0.23]	0.50 [0.00, 0.85]	0.65 [0.23, 1.00]	0.29 [0.21, 0.35]	0.68 [0.00, 0.91]
lr0.0003_wd0.0_h512_d3	0.69 [0.66, 0.73]	0.41 [0.33, 0.50]	0.42 [0.32, 0.52]	0.61 [0.50, 0.70]	0.21 [0.00, 0.38]	0.54 [0.00, 0.83]	0.69 [0.31, 1.00]	0.33 [0.21, 0.47]	0.70 [0.00, 0.95]
lr0.0003_wd0.0_h256_d4	0.69 [0.65, 0.75]	0.38 [0.37, 0.40]	0.39 [0.35, 0.44]	0.57 [0.50, 0.65]	0.12 [0.00, 0.26]	0.33 [0.00, 0.72]	0.81 [0.54, 1.00]	0.27 [0.21, 0.35]	0.52 [0.00, 0.91]
lr0.0003_wd0.0_h400_d4	0.69 [0.64, 0.74]	0.40 [0.35, 0.48]	0.39 [0.26, 0.51]	0.60 [0.50, 0.71]	0.19 [0.00, 0.35]	0.50 [0.00, 0.85]	0.70 [0.23, 1.00]	0.31 [0.21, 0.40]	0.71 [0.00, 1.00]
lr0.0003_wd0.0_h512_d4	0.69 [0.67, 0.71]	0.39 [0.35, 0.44]	0.36 [0.26, 0.47]	0.55 [0.50, 0.67]	0.09 [0.00, 0.30]	0.42 [0.00, 0.85]	0.69 [0.23, 1.00]	0.27 [0.21, 0.39]	0.50 [0.00, 0.88]
lr0.0003_wd0.0001_h256_d3	0.69 [0.67, 0.72]	0.45 [0.41, 0.46]	0.38 [0.32, 0.46]	0.54 [0.47, 0.66]	0.07 [-0.14, 0.27]	0.20 [0.00, 0.63]	0.89 [0.69, 1.00]	0.25 [0.20, 0.35]	0.63 [0.00, 0.89]
lr0.0003_wd0.0001_h400_d3	0.68 [0.66, 0.73]	0.36 [0.31, 0.41]	0.38 [0.31, 0.46]	0.56 [0.50, 0.67]	0.12 [0.00, 0.28]	0.35 [0.00, 0.80]	0.78 [0.31, 1.00]	0.27 [0.21, 0.35]	0.52 [0.00, 0.92]
lr0.0003_wd0.0001_h512_d3	0.66 [0.63, 0.69]	0.40 [0.35, 0.45]	0.35 [0.21, 0.43]	0.58 [0.50, 0.63]	0.15 [0.00, 0.25]	0.54 [0.00, 0.91]	0.61 [0.15, 1.00]	0.30 [0.21, 0.36]	0.69 [0.00, 1.00]
lr0.0003_wd0.0001_h256_d4	0.70 [0.66, 0.76]	0.40 [0.34, 0.44]	0.35 [0.12, 0.53]	0.59 [0.50, 0.75]	0.16 [0.00, 0.42]	0.50 [0.00, 0.93]	0.67 [0.08, 1.00]	0.29 [0.21, 0.36]	0.71 [0.00, 1.00]
lr0.0003_wd0.0001_h400_d4	0.68 [0.66, 0.73]	0.37 [0.33, 0.44]	0.39 [0.36, 0.45]	0.59 [0.50, 0.65]	0.15 [0.00, 0.26]	0.46 [0.00, 0.78]	0.71 [0.38, 1.00]	0.29 [0.22, 0.33]	0.69 [0.00, 0.91]
lr0.0003_wd0.0001_h512_d4	0.69 [0.64, 0.74]	0.40 [0.32, 0.47]	0.40 [0.33, 0.46]	0.60 [0.50, 0.67]	0.17 [0.00, 0.28]	0.50 [0.00, 0.85]	0.70 [0.31, 1.00]	0.31 [0.22, 0.36]	0.69 [0.00, 0.89]
lr0.0003_wd0.001_h256_d3	0.69 [0.65, 0.73]	0.43 [0.38, 0.53]	0.37 [0.24, 0.48]	0.58 [0.52, 0.67]	0.17 [0.04, 0.34]	0.56 [0.18, 0.87]	0.61 [0.23, 1.00]	0.31 [0.24, 0.50]	0.87 [0.79, 1.00]
lr0.0003_wd0.001_h400_d3	0.68 [0.63, 0.75]	0.41 [0.36, 0.46]	0.40 [0.35, 0.49]	0.59 [0.50, 0.69]	0.17 [0.00, 0.31]	0.47 [0.00, 0.87]	0.72 [0.38, 1.00]	0.31 [0.21, 0.45]	0.69 [0.00, 0.90]
lr0.0003_wd0.001_h512_d3	0.69 [0.67, 0.74]	0.40 [0.34, 0.44]	0.35 [0.10, 0.51]	0.58 [0.47, 0.72]	0.14 [-0.07, 0.37]	0.46 [0.00, 0.87]	0.70 [0.08, 1.00]	0.26 [0.14, 0.35]	0.72 [0.00, 1.00]
lr0.0003_wd0.001_h256_d4	0.68 [0.65, 0.72]	0.39 [0.36, 0.41]	0.32 [0.21, 0.44]	0.54 [0.50, 0.64]	0.09 [0.00, 0.25]	0.42 [0.00, 0.91]	0.67 [0.15, 1.00]	0.26 [0.21, 0.33]	0.71 [0.00, 1.00]
lr0.0003_wd0.001_h400_d4	0.68 [0.63, 0.76]	0.43 [0.33, 0.60]	0.42 [0.35, 0.54]	0.60 [0.50, 0.70]	0.19 [0.00, 0.41]	0.43 [0.00, 0.87]	0.78 [0.54, 1.00]	0.32 [0.21, 0.54]	0.70 [0.00, 0.92]
lr0.0003_wd0.001_h512_d4	0.69 [0.66, 0.71]	0.39 [0.34, 0.45]	0.38 [0.35, 0.41]	0.56 [0.50, 0.63]	0.12 [0.00, 0.21]	0.33 [0.00, 0.80]	0.79 [0.38, 1.00]	0.27 [0.21, 0.36]	0.54 [0.00, 1.00]
lr0.001_wd0.0_h256_d3	0.69 [0.66, 0.74]	0.42 [0.34, 0.53]	0.41 [0.35, 0.50]	0.60 [0.51, 0.70]	0.19 [0.04, 0.35]	0.46 [0.11, 0.83]	0.75 [0.38, 0.92]	0.31 [0.22, 0.40]	0.87 [0.83, 0.92]
lr0.001_wd0.0_h400_d3	0.71 [0.68, 0.77]	0.36 [0.33, 0.41]	0.39 [0.35, 0.44]	0.57 [0.50, 0.67]	0.13 [0.00, 0.27]	0.33 [0.00, 0.85]	0.81 [0.38, 1.00]	0.28 [0.21, 0.42]	0.52 [0.00, 0.92]
lr0.001_wd0.0_h512_d3	0.68 [0.67, 0.73]	0.39 [0.35, 0.42]	0.40 [0.36, 0.43]	0.59 [0.50, 0.64]	0.16 [0.00, 0.24]	0.47 [0.00, 0.78]	0.71 [0.46, 1.00]	0.29 [0.22, 0.38]	0.69 [0.00, 0.88]
lr0.001_wd0.0_h256_d4	0.70 [0.67, 0.78]	0.44 [0.31, 0.54]	0.39 [0.35, 0.46]	0.57 [0.50, 0.66]	0.12 [0.00, 0.27]	0.27 [0.00, 0.63]	0.87 [0.69, 1.00]	0.26 [0.21, 0.35]	0.54 [0.00, 0.92]
lr0.001_wd0.0_h400_d4	0.67 [0.63, 0.74]	0.42 [0.29, 0.51]	0.41 [0.36, 0.43]	0.60 [0.50, 0.65]	0.17 [0.00, 0.24]	0.45 [0.00, 0.70]	0.74 [0.54, 1.00]	0.29 [0.22, 0.33]	0.70 [0.00, 0.89]
lr0.001_wd0.0_h512_d4	0.70 [0.67, 0.74]	0.41 [0.32, 0.58]	0.41 [0.36, 0.46]	0.61 [0.52, 0.67]	0.19 [0.04, 0.28]	0.38 [0.11, 0.67]	0.84 [0.67, 0.92]	0.28 [0.23, 0.35]	0.89 [0.83, 0.93]
lr0.001_wd0.0001_h256_d3	0.70 [0.62, 0.80]	0.43 [0.37, 0.49]	0.39 [0.35, 0.44]	0.58 [0.50, 0.65]	0.17 [0.00, 0.27]	0.43 [0.00, 0.91]	0.73 [0.31, 1.00]	0.32 [0.21, 0.50]	0.71 [0.00, 1.00]
lr0.001_wd0.0001_h400_d3	0.70 [0.66, 0.73]	0.44 [0.33, 0.53]	0.31 [0.14, 0.46]	0.54 [0.47, 0.67]	0.11 [-0.14, 0.28]	0.51 [0.02, 1.00]	0.58 [0.08, 1.00]	0.41 [0.20, 1.00]	0.79 [0.50, 1.00]
lr0.001_wd0.0001_h512_d3	0.70 [0.66, 0.75]	0.40 [0.35, 0.51]	0.35 [0.20, 0.45]	0.56 [0.49, 0.66]	0.10 [-0.03, 0.28]	0.42 [0.00, 0.89]	0.70 [0.15, 1.00]	0.27 [0.21, 0.37]	0.66 [0.00, 0.90]
lr0.001_wd0.0001_h256_d4	0.71 [0.68, 0.73]	0.41 [0.35, 0.51]	0.45 [0.38, 0.52]	0.65 [0.59, 0.70]	0.27 [0.14, 0.36]	0.60 [0.42, 0.80]	0.71 [0.42, 0.92]	0.34 [0.26, 0.44]	0.89 [0.84, 0.95]
lr0.001_wd0.0001_h400_d4	0.70 [0.67, 0.73]	0.41 [0.36, 0.47]	0.38 [0.27, 0.50]	0.60 [0.55, 0.69]	0.18 [0.12, 0.33]	0.63 [0.20, 0.87]	0.58 [0.23, 0.92]	0.32 [0.23, 0.39]	0.85 [0.80, 0.90]
lr0.001_wd0.0001_h512_d4	0.68 [0.65, 0.73]	0.36 [0.32, 0.43]	0.38 [0.32, 0.44]	0.58 [0.50, 0.64]	0.14 [0.00, 0.28]	0.47 [0.00, 0.74]	0.68 [0.42, 1.00]	0.28 [0.21, 0.37]	0.70 [0.00, 1.00]
lr0.001_wd0.001_h256_d3	0.69 [0.64, 0.72]	0.40 [0.31, 0.47]	0.34 [0.10, 0.48]	0.59 [0.47, 0.68]	0.15 [-0.07, 0.32]	0.71 [0.39, 0.87]	0.46 [0.08, 0.85]	0.29 [0.14, 0.41]	0.84 [0.77, 0.90]
lr0.001_wd0.001_h400_d3	0.68 [0.63, 0.77]	0.43 [0.39, 0.53]	0.36 [0.35, 0.39]	0.54 [0.50, 0.59]	0.07 [0.00, 0.16]	0.27 [0.00, 0.76]	0.81 [0.42, 1.00]	0.25 [0.21, 0.31]	0.51 [0.00, 0.90]
lr0.001_wd0.001_h512_d3	0.71 [0.66, 0.76]	0.42 [0.39, 0.48]	0.43 [0.38, 0.49]	0.63 [0.57, 0.69]	0.23 [0.13, 0.31]	0.51 [0.31, 0.61]	0.76 [0.62, 0.83]	0.30 [0.24, 0.36]	0.89 [0.85, 0.91]
lr0.001_wd0.001_h256_d4	0.70 [0.67, 0.75]	0.40 [0.32, 0.44]	0.40 [0.26, 0.54]	0.61 [0.50, 0.74]	0.18 [0.00, 0.40]	0.52 [0.00, 0.83]	0.70 [0.25, 1.00]	0.30 [0.21, 0.39]	0.70 [0.00, 0.94]
lr0.001_wd0.001_h400_d4	0.69 [0.65, 0.72]	0.39 [0.34, 0.45]	0.39 [0.24, 0.50]	0.61 [0.50, 0.71]	0.18 [0.00, 0.36]	0.52 [0.00, 0.80]	0.69 [0.23, 1.00]	0.29 [0.21, 0.38]	0.70 [0.00, 0.96]
lr0.001_wd0.001_h512_d4	0.70 [0.65, 0.75]	0.40 [0.34, 0.44]	0.41 [0.36, 0.43]	0.62 [0.59, 0.63]	0.22 [0.16, 0.27]	0.50 [0.26, 0.76]	0.74 [0.42, 1.00]	0.30 [0.27, 0.33]	0.90 [0.83, 1.00]

Supplementary Table 17: Grid search results on the classification task on SYSMH external validation dataset (choosing 50% as the threshold for clinical variables selection). Reported metrics are AUC, AUPRC, F1, Balanced Accuracy, MCC, Specificity, Sensitivity, PPV, and NPV.

Config	AUC	AUPRC	F1	Balanced_Accuracy	MCC	Specificity	Sensitivity	PPV	NPV
lr0.0001_wd0.0_h256_d1	0.74 [0.64, 0.80]	0.43 [0.34, 0.54]	0.23 [0.17, 0.29]	0.56 [0.50, 0.59]	0.10 [0.00, 0.21]	0.50 [0.00, 0.93]	0.62 [0.25, 1.00]	0.17 [0.11, 0.33]	0.74 [0.00, 1.00]
lr0.0001_wd0.0_h400_d1	0.74 [0.67, 0.82]	0.53 [0.45, 0.60]	0.28 [0.18, 0.33]	0.61 [0.53, 0.72]	0.19 [0.04, 0.30]	0.61 [0.39, 0.97]	0.62 [0.25, 1.00]	0.23 [0.11, 0.50]	0.93 [0.90, 1.00]
lr0.0001_wd0.0_h512_d1	0.74 [0.64, 0.81]	0.42 [0.28, 0.60]	0.23 [0.17, 0.31]	0.55 [0.49, 0.63]	0.08 [-0.01, 0.21]	0.51 [0.00, 0.93]	0.58 [0.25, 1.00]	0.18 [0.10, 0.33]	0.72 [0.00, 0.92]
lr0.0001_wd0.0_h256_d2	0.73 [0.61, 0.80]	0.41 [0.21, 0.51]	0.27 [0.15, 0.33]	0.57 [0.46, 0.65]	0.14 [-0.06, 0.29]	0.59 [0.00, 0.97]	0.55 [0.25, 1.00]	0.26 [0.09, 0.50]	0.73 [0.00, 0.93]
lr0.0001_wd0.0_h400_d2	0.75 [0.67, 0.82]	0.37 [0.17, 0.62]	0.22 [0.00, 0.40]	0.56 [0.50, 0.68]	0.12 [0.00, 0.31]	0.64 [0.00, 1.00]	0.48 [0.00, 1.00]	0.21 [0.00, 0.50]	0.73 [0.00, 0.93]
lr0.0001_wd0.0_h512_d2	0.75 [0.65, 0.84]	0.44 [0.27, 0.57]	0.28 [0.19, 0.36]	0.61 [0.55, 0.66]	0.20 [0.11, 0.29]	0.57 [0.11, 0.97]	0.65 [0.25, 1.00]	0.25 [0.11, 0.50]	0.95 [0.90, 1.00]
lr0.0001_wd0.0001_h256_d1	0.75 [0.67, 0.82]	0.47 [0.26, 0.62]	0.30 [0.21, 0.44]	0.63 [0.57, 0.72]	0.20 [0.10, 0.36]	0.61 [0.14, 0.90]	0.65 [0.33, 1.00]	0.22 [0.12, 0.40]	0.95 [0.92, 1.00]
lr0.0001_wd0.0001_h400_d1	0.75 [0.64, 0.81]	0.39 [0.24, 0.55]	0.28 [0.22, 0.36]	0.60 [0.50, 0.66]	0.17 [0.00, 0.29]	0.65 [0.00, 0.97]	0.55 [0.25, 1.00]	0.25 [0.12, 0.50]	0.74 [0.00, 0.94]
lr0.0001_wd0.0001_h512_d1	0.74 [0.58, 0.83]	0.44 [0.22, 0.59]	0.32 [0.14, 0.50]	0.62 [0.42, 0.74]	0.19 [-0.12, 0.43]	0.68 [0.18, 0.93]	0.55 [0.33, 0.75]	0.25 [0.08, 0.50]	0.91 [0.83, 0.95]
lr0.0001_wd0.0001_h256_d2	0.76 [0.68, 0.82]	0.34 [0.20, 0.55]	0.23 [0.00, 0.36]	0.58 [0.48, 0.66]	0.14 [-0.03, 0.29]	0.77 [0.29, 1.00]	0.38 [0.00, 0.67]	0.22 [0.00, 0.50]	0.91 [0.89, 0.92]
lr0.0001_wd0.0001_h400_d2	0.77 [0.67, 0.83]	0.36 [0.23, 0.57]	0.18 [0.00, 0.40]	0.56 [0.48, 0.68]	0.09 [-0.07, 0.31]	0.80 [0.39, 1.00]	0.33 [0.00, 0.67]	0.13 [0.00, 0.33]	0.91 [0.88, 0.93]
lr0.0001_wd0.0001_h512_d2	0.76 [0.67, 0.83]	0.37 [0.18, 0.56]	0.18 [0.00, 0.36]	0.58 [0.48, 0.66]	0.10 [-0.07, 0.26]	0.82 [0.54, 1.00]	0.33 [0.00, 0.67]	0.13 [0.00, 0.29]	0.91 [0.88, 0.94]
lr0.0001_wd0.001_h256_d1	0.74 [0.63, 0.79]	0.45 [0.18, 0.54]	0.25 [0.18, 0.33]	0.57 [0.50, 0.63]	0.13 [0.00, 0.29]	0.59 [0.00, 0.97]	0.55 [0.25, 1.00]	0.22 [0.11, 0.50]	0.73 [0.00, 0.92]
lr0.0001_wd0.001_h400_d1	0.76 [0.67, 0.84]	0.48 [0.28, 0.54]	0.29 [0.16, 0.50]	0.60 [0.48, 0.72]	0.17 [-0.03, 0.43]	0.54 [0.29, 0.93]	0.67 [0.50, 1.00]	0.22 [0.09, 0.50]	0.93 [0.89, 1.00]
lr0.0001_wd0.001_h512_d1	0.76 [0.69, 0.81]	0.47 [0.27, 0.57]	0.27 [0.18, 0.40]	0.59 [0.54, 0.68]	0.17 [0.06, 0.31]	0.74 [0.43, 0.97]	0.45 [0.25, 0.67]	0.25 [0.11, 0.50]	0.91 [0.90, 0.93]
lr0.0001_wd0.001_h256_d2	0.76 [0.68, 0.82]	0.39 [0.27, 0.52]	0.27 [0.18, 0.36]	0.60 [0.54, 0.66]	0.17 [0.06, 0.29]	0.70 [0.46, 0.97]	0.50 [0.25, 0.75]	0.24 [0.12, 0.50]	0.92 [0.90, 0.94]
lr0.0001_wd0.001_h400_d2	0.75 [0.64, 0.82]	0.46 [0.32, 0.63]	0.21 [0.00, 0.44]	0.56 [0.48, 0.70]	0.09 [-0.07, 0.36]	0.62 [0.00, 0.97]	0.50 [0.00, 1.00]	0.16 [0.00, 0.40]	0.73 [0.00, 0.93]
lr0.0001_wd0.001_h512_d2	0.74 [0.67, 0.82]	0.34 [0.22, 0.55]	0.29 [0.15, 0.44]	0.60 [0.46, 0.70]	0.17 [-0.06, 0.36]	0.69 [0.25, 0.93]	0.52 [0.25, 0.67]	0.24 [0.09, 0.40]	0.91 [0.88, 0.94]
lr0.0003_wd0.0_h256_d1	0.76 [0.67, 0.83]	0.44 [0.23, 0.65]	0.34 [0.18, 0.50]	0.66 [0.53, 0.79]	0.24 [0.04, 0.44]	0.70 [0.39, 0.90]	0.62 [0.50, 0.75]	0.25 [0.11, 0.40]	0.93 [0.92, 0.96]
lr0.0003_wd0.0_h400_d1	0.76 [0.67, 0.84]	0.35 [0.20, 0.53]	0.29 [0.00, 0.46]	0.64 [0.46, 0.77]	0.21 [-0.09, 0.40]	0.80 [0.57, 0.93]	0.48 [0.00, 0.75]	0.23 [0.00, 0.40]	0.93 [0.90, 0.96]
lr0.0003_wd0.0_h512_d1	0.74 [0.62, 0.83]	0.44 [0.26, 0.65]	0.28 [0.13, 0.50]	0.60 [0.39, 0.79]	0.16 [-0.20, 0.44]	0.61 [0.11, 0.93]	0.60 [0.25, 1.00]	0.22 [0.07, 0.38]	0.90 [0.75, 1.00]
lr0.0003_wd0.0_h256_d2	0.75 [0.64, 0.82]	0.40 [0.26, 0.52]	0.29 [0.18, 0.40]	0.61 [0.53, 0.68]	0.19 [0.04, 0.31]	0.77 [0.39, 0.93]	0.45 [0.25, 0.67]	0.25 [0.11, 0.33]	0.92 [0.90, 0.93]
lr0.0003_wd0.0_h400_d2	0.76 [0.65, 0.84]	0.43 [0.24, 0.65]	0.22 [0.00, 0.40]	0.58 [0.48, 0.68]	0.11 [-0.07, 0.31]	0.70 [0.43, 0.97]	0.47 [0.00, 0.67]	0.16 [0.00, 0.33]	0.91 [0.88, 0.93]
lr0.0003_wd0.0_h512_d2	0.77 [0.64, 0.85]	0.41 [0.25, 0.53]	0.33 [0.21, 0.46]	0.65 [0.61, 0.77]	0.26 [0.16, 0.40]	0.74 [0.21, 0.97]	0.57 [0.25, 1.00]	0.30 [0.12, 0.50]	0.94 [0.90, 1.00]
lr0.0003_wd0.0001_h256_d1	0.76 [0.64, 0.83]	0.42 [0.29, 0.52]	0.27 [0.16, 0.40]	0.59 [0.48, 0.68]	0.16 [-0.03, 0.31]	0.66 [0.29, 0.97]	0.52 [0.25, 0.67]	0.25 [0.09, 0.50]	0.91 [0.89, 0.93]
lr0.0003_wd0.0001_h400_d1	0.74 [0.62, 0.84]	0.40 [0.21, 0.65]	0.30 [0.20, 0.43]	0.64 [0.56, 0.75]	0.22 [0.08, 0.36]	0.78 [0.64, 0.97]	0.50 [0.25, 0.75]	0.26 [0.14, 0.50]	0.93 [0.90, 0.96]
lr0.0003_wd0.0001_h512_d1	0.75 [0.61, 0.84]	0.40 [0.19, 0.58]	0.29 [0.15, 0.44]	0.60 [0.51, 0.70]	0.20 [0.01, 0.36]	0.72 [0.17, 0.97]	0.48 [0.25, 1.00]	0.28 [0.10, 0.50]	0.93 [0.90, 1.00]
lr0.0003_wd0.0001_h256_d2	0.76 [0.65, 0.84]	0.45 [0.31, 0.62]	0.31 [0.13, 0.50]	0.66 [0.47, 0.86]	0.22 [-0.04, 0.49]	0.67 [0.57, 0.76]	0.65 [0.33, 1.00]	0.21 [0.08, 0.33]	0.94 [0.89, 1.00]
lr0.0003_wd0.0001_h400_d2	0.75 [0.63, 0.83]	0.36 [0.21, 0.52]	0.30 [0.19, 0.44]	0.61 [0.55, 0.70]	0.21 [0.11, 0.36]	0.71 [0.11, 0.97]	0.52 [0.25, 1.00]	0.28 [0.11, 0.50]	0.93 [0.90, 1.00]
lr0.0003_wd0.0001_h512_d2	0.78 [0.69, 0.85]	0.45 [0.23, 0.63]	0.31 [0.14, 0.43]	0.63 [0.49, 0.75]	0.21 [-0.01, 0.36]	0.82 [0.64, 0.93]	0.43 [0.25, 0.75]	0.26 [0.09, 0.33]	0.92 [0.90, 0.96]
lr0.0003_wd0.001_h256_d1	0.74 [0.62, 0.82]	0.44 [0.26, 0.62]	0.27 [0.11, 0.40]	0.61 [0.40, 0.74]	0.16 [-0.12, 0.32]	0.61 [0.46, 0.86]	0.60 [0.33, 1.00]	0.19 [0.06, 0.33]	0.93 [0.87, 1.00]
lr0.0003_wd0.001_h400_d1	0.76 [0.62, 0.86]	0.42 [0.21, 0.64]	0.31 [0.25, 0.35]	0.65 [0.60, 0.74]	0.24 [0.15, 0.32]	0.75 [0.48, 0.97]	0.55 [0.25, 1.00]	0.27 [0.17, 0.50]	0.94 [0.90, 1.00]
lr0.0003_wd0.001_h512_d1	0.73 [0.61, 0.81]	0.38 [0.18, 0.55]	0.29 [0.21, 0.40]	0.64 [0.58, 0.79]	0.22 [0.10, 0.38]	0.73 [0.50, 0.97]	0.55 [0.25, 1.00]	0.25 [0.12, 0.50]	0.93 [0.90, 1.00]
lr0.0003_wd0.001_h256_d2	0.78 [0.67, 0.85]	0.45 [0.35, 0.70]	0.29 [0.22, 0.46]	0.65 [0.58, 0.77]	0.21 [0.12, 0.40]	0.54 [0.21, 0.82]	0.75 [0.33, 1.00]	0.19 [0.15, 0.33]	0.96 [0.92, 1.00]
lr0.0003_wd0.001_h400_d2	0.76 [0.63, 0.85]	0.41 [0.25, 0.57]	0.34 [0.18, 0.57]	0.63 [0.50, 0.75]	0.25 [0.00, 0.53]	0.70 [0.00, 0.97]	0.57 [0.25, 1.00]	0.34 [0.10, 0.67]	0.74 [0.00, 0.96]
lr0.0003_wd0.001_h512_d2	0.76 [0.65, 0.83]	0.38 [0.20, 0.64]	0.26 [0.00, 0.44]	0.62 [0.48, 0.75]	0.17 [-0.07, 0.36]	0.79 [0.54, 0.97]	0.45 [0.00, 0.75]	0.20 [0.00, 0.40]	0.92 [0.88, 0.96]
lr0.001_wd0.0_h256_d1	0.77 [0.63, 0.87]	0.42 [0.19, 0.52]	0.32 [0.18, 0.40]	0.66 [0.54, 0.74]	0.25 [0.06, 0.33]	0.75 [0.46, 0.97]	0.57 [0.25, 1.00]	0.28 [0.12, 0.50]	0.94 [0.90, 1.00]
lr0.001_wd0.0_h400_d1	0.75 [0.65, 0.84]	0.40 [0.32, 0.54]	0.24 [0.00, 0.40]	0.62 [0.48, 0.74]	0.16 [-0.07, 0.33]	0.75 [0.54, 0.97]	0.50 [0.00, 0.75]	0.16 [0.00, 0.27]	0.93 [0.88, 0.95]
lr0.001_wd0.0_h512_d1	0.75 [0.62, 0.83]	0.37 [0.27, 0.52]	0.24 [0.00, 0.50]	0.60 [0.43, 0.79]	0.14 [-0.14, 0.44]	0.80 [0.54, 0.90]	0.40 [0.00, 0.75]	0.19 [0.00, 0.38]	0.92 [0.86, 0.96]
lr0.001_wd0.0_h256_d2	0.77 [0.63, 0.85]	0.35 [0.22, 0.54]	0.29 [0.00, 0.50]	0.64 [0.47, 0.79]	0.21 [-0.09, 0.44]	0.84 [0.76, 0.93]	0.45 [0.00, 0.75]	0.22 [0.00, 0.38]	0.93 [0.87, 0.96]
lr0.001_wd0.0_h400_d2	0.74 [0.62, 0.81]	0.35 [0.17, 0.46]	0.24 [0.00, 0.40]	0.62 [0.48, 0.74]	0.17 [-0.07, 0.33]	0.66 [0.14, 0.97]	0.58 [0.00, 1.00]	0.16 [0.00, 0.27]	0.94 [0.88, 1.00]
lr0.001_wd0.0_h512_d2	0.79 [0.68, 0.86]	0.44 [0.27, 0.57]	0.39 [0.20, 0.57]	0.68 [0.56, 0.80]	0.30 [0.08, 0.52]	0.81 [0.66, 0.93]	0.55 [0.33, 0.75]	0.32 [0.14, 0.50]	0.94 [0.92, 0.96]
lr0.001_wd0.0001_h256_d1	0.74 [0.64, 0.82]	0.37 [0.26, 0.50]	0.23 [0.00, 0.40]	0.57 [0.46, 0.68]	0.14 [-0.06, 0.31]	0.76 [0.25, 1.00]	0.38 [0.00, 0.67]	0.22 [0.00, 0.50]	0.90 [0.88, 0.93]
lr0.001_wd0.0001_h400_d1	0.74 [0.62, 0.82]	0.43 [0.25, 0.56]	0.28 [0.18, 0.40]	0.60 [0.53, 0.68]	0.18 [0.04, 0.31]	0.75 [0.39, 0.97]	0.45 [0.25, 0.67]	0.26 [0.11, 0.50]	0.91 [0.90, 0.93]
lr0.001_wd0.0001_h512_d1	0.76 [0.64, 0.85]	0.38 [0.22, 0.60]	0.30 [0.19, 0.50]	0.62 [0.55, 0.72]	0.19 [0.06, 0.43]	0.68 [0.43, 0.93]	0.57 [0.50, 0.67]	0.24 [0.11, 0.50]	0.92 [0.91, 0.94]
lr0.001_wd0.0001_h256_d2	0.79 [0.68, 0.85]	0.46 [0.28, 0.55]	0.30 [0.19, 0.40]	0.63 [0.55, 0.68]	0.22 [0.06, 0.31]	0.67 [0.29, 0.97]	0.58 [0.25, 1.00]	0.27 [0.11, 0.50]	0.94 [0.90, 1.00]
lr0.001_wd0.0001_h400_d2	0.78 [0.69, 0.84]	0.46 [0.21, 0.63]	0.25 [0.00, 0.40]	0.61 [0.48, 0.68]	0.16 [-0.07, 0.31]	0.75 [0.50, 0.97]	0.47 [0.00, 0.67]	0.19 [0.00, 0.33]	0.92 [0.88, 0.94]
lr0.001_wd0.0001_h512_d2	0.78 [0.71, 0.83]	0.47 [0.26, 0.70]	0.35 [0.29, 0.44]	0.69 [0.59, 0.77]	0.28 [0.21, 0.36]	0.79 [0.54, 0.93]	0.58 [0.25, 1.00]	0.28 [0.19, 0.40]	0.94 [0.90, 1.00]
lr0.001_wd0.001_h256_d1	0.77 [0.68, 0.85]	0.37 [0.18, 0.55]	0.25 [0.00, 0.46]	0.63 [0.48, 0.77]	0.18 [-0.07, 0.40]	0.69 [0.21, 0.97]	0.57 [0.00, 1.00]	0.17 [0.00, 0.33]	0.94 [0.88, 1.00]
lr0.001_wd0.001_h400_d1	0.74 [0.62, 0.82]	0.40 [0.15, 0.53]	0.31 [0.21, 0.40]	0.64 [0.61, 0.68]	0.22 [0.14, 0.31]	0.61 [0.21, 0.86]	0.68 [0.50, 1.00]	0.22 [0.12, 0.33]	0.95 [0.93, 1.00]
lr0.001_wd0.001_h512_d1	0.73 [0.63, 0.82]	0.41 [0.20, 0.50]	0.30 [0.25, 0.40]	0.64 [0.58, 0.68]	0.19 [0.11, 0.31]	0.66 [0.48, 0.86]	0.62 [0.50, 0.75]	0.20 [0.17, 0.33]	0.93 [0.90, 0.95]
lr0.001_wd0.001_h256_d2	0.77 [0.68, 0.85]	0.49 [0.29, 0.63]	0.29 [0.22, 0.36]	0.61 [0.58, 0.66]	0.19 [0.12, 0.29]	0.83 [0.72, 0.97]	0.38 [0.25, 0.50]	0.26 [0.17, 0.50]	0.92 [0.90, 0.92]
lr0.001_wd0.001_h400_d2	0.78 [0.68, 0.88]	0.47 [0.32, 0.59]	0.28 [0.00, 0.50]	0.61 [0.47, 0.76]	0.19 [-0.09, 0.43]	0.73 [0.10, 0.93]	0.50 [0.00, 1.00]	0.23 [0.00, 0.50]	0.94 [0.87, 1.00]
lr0.001_wd0.001_h512_d2	0.76 [0.63, 0.84]	0.41 [0.17, 0.58]	0.31 [0.00, 0.57]	0.64 [0.50, 0.73]	0.25 [0.00, 0.53]	0.81 [0.39, 1.00]	0.47 [0.00, 0.67]	0.28 [0.00, 0.67]	0.92 [0.88, 0.96]

Supplementary Table 18: Grid search results on the classification task on GDHCM retrospective dataset (choosing 40% as the threshold for clinical variables selection). Reported metrics are AUC, AUPRC, F1, Balanced Accuracy, MCC, Specificity, Sensitivity, PPV, and NPV.

Config	AUC	AUPRC	F1	Balanced_Accuracy	MCC	Specificity	Sensitivity	PPV	NPV
lr1e-05_wd0.005_h1024_d3	0.73 [0.68, 0.78]	0.37 [0.19, 0.51]	0.17 [0.00, 0.33]	0.54 [0.50, 0.61]	0.09 [0.00, 0.29]	0.74 [0.00, 1.00]	0.35 [0.00, 1.00]	0.16 [0.00, 0.50]	0.72 [0.00, 0.91]
lr1e-05_wd0.005_h2048_d3	0.72 [0.62, 0.82]	0.37 [0.16, 0.52]	0.24 [0.00, 0.36]	0.60 [0.50, 0.66]	0.17 [0.00, 0.29]	0.81 [0.50, 1.00]	0.38 [0.00, 0.67]	0.23 [0.00, 0.50]	0.92 [0.90, 0.93]
lr1e-05_wd0.005_h1024_d4	0.75 [0.69, 0.80]	0.37 [0.20, 0.53]	0.22 [0.00, 0.33]	0.57 [0.50, 0.63]	0.13 [0.00, 0.29]	0.66 [0.00, 1.00]	0.48 [0.00, 1.00]	0.20 [0.00, 0.50]	0.73 [0.00, 0.94]
lr1e-05_wd0.005_h2048_d4	0.74 [0.67, 0.80]	0.39 [0.26, 0.53]	0.30 [0.18, 0.44]	0.61 [0.50, 0.70]	0.19 [0.00, 0.36]	0.63 [0.00, 0.97]	0.58 [0.25, 1.00]	0.27 [0.10, 0.50]	0.74 [0.00, 0.94]
lr1e-05_wd0.01_h1024_d3	0.74 [0.62, 0.83]	0.42 [0.17, 0.52]	0.24 [0.00, 0.44]	0.59 [0.50, 0.70]	0.14 [0.00, 0.36]	0.65 [0.00, 1.00]	0.53 [0.00, 1.00]	0.18 [0.00, 0.40]	0.74 [0.00, 0.95]
lr1e-05_wd0.01_h2048_d3	0.74 [0.62, 0.81]	0.37 [0.20, 0.53]	0.25 [0.00, 0.40]	0.60 [0.50, 0.68]	0.22 [0.00, 0.48]	0.64 [0.14, 1.00]	0.55 [0.00, 1.00]	0.32 [0.00, 1.00]	0.95 [0.90, 1.00]
lr1e-05_wd0.01_h1024_d4	0.74 [0.63, 0.81]	0.38 [0.19, 0.53]	0.25 [0.00, 0.57]	0.57 [0.50, 0.73]	0.15 [0.00, 0.53]	0.62 [0.00, 1.00]	0.53 [0.00, 1.00]	0.22 [0.00, 0.67]	0.73 [0.00, 0.93]
lr1e-05_wd0.01_h2048_d4	0.72 [0.61, 0.79]	0.47 [0.16, 0.61]	0.28 [0.17, 0.50]	0.59 [0.50, 0.72]	0.15 [0.00, 0.43]	0.64 [0.00, 0.93]	0.53 [0.33, 1.00]	0.22 [0.11, 0.50]	0.73 [0.00, 0.93]
lr3e-05_wd0.005_h1024_d3	0.72 [0.62, 0.80]	0.37 [0.16, 0.54]	0.26 [0.00, 0.50]	0.59 [0.50, 0.72]	0.19 [0.00, 0.43]	0.73 [0.11, 1.00]	0.45 [0.00, 1.00]	0.25 [0.00, 0.50]	0.93 [0.90, 1.00]
lr3e-05_wd0.005_h2048_d3	0.74 [0.65, 0.83]	0.41 [0.17, 0.63]	0.22 [0.00, 0.36]	0.57 [0.50, 0.66]	0.14 [0.00, 0.29]	0.65 [0.03, 1.00]	0.48 [0.00, 1.00]	0.20 [0.00, 0.50]	0.93 [0.90, 1.00]
lr3e-05_wd0.005_h1024_d4	0.74 [0.67, 0.79]	0.36 [0.21, 0.61]	0.16 [0.00, 0.33]	0.56 [0.44, 0.71]	0.07 [-0.08, 0.28]	0.68 [0.21, 1.00]	0.43 [0.00, 1.00]	0.10 [0.00, 0.25]	0.91 [0.86, 1.00]
lr3e-05_wd0.005_h2048_d4	0.76 [0.68, 0.86]	0.35 [0.18, 0.45]	0.18 [0.00, 0.44]	0.56 [0.48, 0.70]	0.09 [-0.07, 0.36]	0.69 [0.00, 1.00]	0.43 [0.00, 1.00]	0.14 [0.00, 0.40]	0.73 [0.00, 0.94]
lr3e-05_wd0.01_h1024_d3	0.74 [0.65, 0.79]	0.38 [0.27, 0.52]	0.22 [0.00, 0.33]	0.58 [0.48, 0.65]	0.15 [-0.06, 0.29]	0.61 [0.14, 0.97]	0.55 [0.00, 1.00]	0.20 [0.00, 0.50]	0.94 [0.90, 1.00]
lr3e-05_wd0.01_h2048_d3	0.74 [0.65, 0.81]	0.38 [0.18, 0.54]	0.24 [0.00, 0.44]	0.58 [0.50, 0.70]	0.15 [0.00, 0.36]	0.70 [0.00, 1.00]	0.45 [0.00, 1.00]	0.23 [0.00, 0.50]	0.73 [0.00, 0.93]
lr3e-05_wd0.01_h1024_d4	0.73 [0.64, 0.80]	0.43 [0.28, 0.63]	0.27 [0.15, 0.40]	0.61 [0.51, 0.68]	0.17 [0.01, 0.31]	0.54 [0.14, 0.86]	0.67 [0.33, 1.00]	0.19 [0.10, 0.33]	0.95 [0.90, 1.00]
lr3e-05_wd0.01_h2048_d4	0.76 [0.62, 0.85]	0.43 [0.18, 0.64]	0.23 [0.00, 0.44]	0.54 [0.40, 0.70]	0.10 [-0.15, 0.36]	0.60 [0.00, 1.00]	0.48 [0.00, 1.00]	0.22 [0.00, 0.50]	0.71 [0.00, 0.93]
lr1e-05_wd0.0_h256_d1	0.69 [0.63, 0.76]	0.31 [0.22, 0.46]	0.21 [0.15, 0.27]	0.54 [0.44, 0.62]	0.08 [-0.08, 0.19]	0.30 [0.07, 0.79]	0.78 [0.25, 1.00]	0.13 [0.08, 0.15]	0.95 [0.86, 1.00]
lr1e-05_wd0.0_h400_d1	0.72 [0.61, 0.79]	0.37 [0.15, 0.61]	0.21 [0.00, 0.33]	0.58 [0.46, 0.72]	0.11 [-0.06, 0.30]	0.48 [0.25, 0.96]	0.68 [0.00, 1.00]	0.12 [0.00, 0.20]	0.94 [0.88, 1.00]
lr1e-05_wd0.0_h512_d1	0.69 [0.62, 0.77]	0.33 [0.21, 0.59]	0.26 [0.16, 0.33]	0.59 [0.48, 0.71]	0.13 [-0.03, 0.28]	0.53 [0.29, 0.93]	0.65 [0.33, 1.00]	0.18 [0.09, 0.33]	0.93 [0.88, 1.00]
lr1e-05_wd0.0_h256_d2	0.73 [0.64, 0.80]	0.34 [0.16, 0.62]	0.25 [0.00, 0.35]	0.63 [0.48, 0.74]	0.17 [-0.06, 0.32]	0.62 [0.14, 0.96]	0.63 [0.00, 1.00]	0.16 [0.00, 0.25]	0.95 [0.90, 1.00]
lr1e-05_wd0.0_h400_d2	0.81 [0.74, 0.90]	0.45 [0.32, 0.60]	0.29 [0.22, 0.40]	0.58 [0.50, 0.65]	0.17 [0.00, 0.36]	0.43 [0.00, 0.96]	0.73 [0.33, 1.00]	0.25 [0.12, 0.50]	0.57 [0.00, 1.00]
lr1e-05_wd0.0_h512_d2	0.70 [0.62, 0.78]	0.35 [0.16, 0.59]	0.17 [0.00, 0.36]	0.58 [0.46, 0.76]	0.10 [-0.09, 0.34]	0.55 [0.00, 1.00]	0.60 [0.00, 1.00]	0.10 [0.00, 0.22]	0.76 [0.00, 1.00]
lr1e-05_wd0.0001_h256_d1	0.70 [0.62, 0.78]	0.40 [0.20, 0.63]	0.18 [0.00, 0.27]	0.54 [0.50, 0.62]	0.06 [0.00, 0.19]	0.40 [0.00, 1.00]	0.68 [0.00, 1.00]	0.10 [0.00, 0.15]	0.75 [0.00, 1.00]
lr1e-05_wd0.0001_h400_d1	0.74 [0.60, 0.89]	0.37 [0.17, 0.60]	0.20 [0.00, 0.29]	0.57 [0.48, 0.66]	0.10 [-0.06, 0.23]	0.34 [0.00, 0.96]	0.80 [0.00, 1.00]	0.11 [0.00, 0.17]	0.78 [0.00, 1.00]
lr1e-05_wd0.0001_h512_d1	0.71 [0.57, 0.84]	0.37 [0.16, 0.64]	0.25 [0.16, 0.33]	0.59 [0.48, 0.69]	0.13 [-0.03, 0.24]	0.48 [0.14, 0.82]	0.70 [0.33, 1.00]	0.16 [0.09, 0.21]	0.94 [0.89, 1.00]
lr1e-05_wd0.0001_h256_d2	0.74 [0.65, 0.90]	0.35 [0.17, 0.60]	0.15 [0.00, 0.27]	0.54 [0.50, 0.62]	0.07 [0.00, 0.19]	0.48 [0.00, 1.00]	0.60 [0.00, 1.00]	0.08 [0.00, 0.15]	0.76 [0.00, 1.00]
lr1e-05_wd0.0001_h400_d2	0.73 [0.68, 0.78]	0.32 [0.27, 0.46]	0.22 [0.00, 0.31]	0.59 [0.50, 0.67]	0.14 [0.00, 0.24]	0.61 [0.03, 1.00]	0.57 [0.00, 1.00]	0.15 [0.00, 0.25]	0.95 [0.90, 1.00]
lr1e-05_wd0.0001_h512_d2	0.70 [0.61, 0.78]	0.32 [0.16, 0.52]	0.18 [0.00, 0.27]	0.54 [0.50, 0.62]	0.07 [0.00, 0.15]	0.40 [0.03, 1.00]	0.68 [0.00, 1.00]	0.11 [0.00, 0.17]	0.95 [0.90, 1.00]
lr1e-05_wd0.001_h256_d1	0.70 [0.55, 0.78]	0.33 [0.25, 0.46]	0.22 [0.17, 0.27]	0.57 [0.49, 0.62]	0.11 [-0.01, 0.19]	0.27 [0.21, 0.32]	0.87 [0.67, 1.00]	0.13 [0.10, 0.15]	0.96 [0.90, 1.00]
lr1e-05_wd0.001_h400_d1	0.73 [0.62, 0.83]	0.35 [0.19, 0.61]	0.20 [0.00, 0.32]	0.56 [0.50, 0.67]	0.09 [0.00, 0.22]	0.42 [0.00, 1.00]	0.70 [0.00, 1.00]	0.12 [0.00, 0.20]	0.75 [0.00, 1.00]
lr1e-05_wd0.001_h512_d1	0.72 [0.62, 0.76]	0.39 [0.16, 0.54]	0.19 [0.00, 0.26]	0.57 [0.50, 0.64]	0.11 [0.00, 0.19]	0.44 [0.14, 1.00]	0.70 [0.00, 1.00]	0.11 [0.00, 0.15]	0.96 [0.89, 1.00]
lr1e-05_wd0.001_h256_d2	0.73 [0.70, 0.76]	0.29 [0.19, 0.43]	0.14 [0.00, 0.27]	0.52 [0.48, 0.62]	0.01 [-0.06, 0.19]	0.43 [0.00, 0.96]	0.60 [0.00, 1.00]	0.08 [0.00, 0.15]	0.56 [0.00, 1.00]
lr1e-05_wd0.001_h400_d2	0.77 [0.70, 0.82]	0.35 [0.26, 0.50]	0.24 [0.21, 0.30]	0.58 [0.50, 0.67]	0.12 [0.00, 0.24]	0.29 [0.00, 0.82]	0.87 [0.33, 1.00]	0.14 [0.12, 0.17]	0.78 [0.00, 1.00]
lr1e-05_wd0.001_h512_d2	0.75 [0.63, 0.81]	0.42 [0.23, 0.61]	0.24 [0.20, 0.33]	0.59 [0.50, 0.72]	0.13 [0.00, 0.30]	0.37 [0.00, 0.79]	0.82 [0.33, 1.00]	0.15 [0.11, 0.20]	0.77 [0.00, 1.00]
lr3e-05_wd0.0_h256_d1	0.73 [0.68, 0.81]	0.37 [0.19, 0.59]	0.23 [0.00, 0.36]	0.60 [0.50, 0.70]	0.15 [0.00, 0.27]	0.61 [0.07, 1.00]	0.58 [0.00, 1.00]	0.15 [0.00, 0.29]	0.94 [0.90, 1.00]
lr3e-05_wd0.0_h400_d1	0.71 [0.62, 0.78]	0.34 [0.17, 0.49]	0.19 [0.00, 0.31]	0.54 [0.46, 0.63]	0.06 [-0.09, 0.19]	0.70 [0.32, 0.93]	0.38 [0.00, 0.67]	0.14 [0.00, 0.25]	0.90 [0.89, 0.92]
lr3e-05_wd0.0_h512_d1	0.71 [0.62, 0.80]	0.43 [0.22, 0.60]	0.24 [0.12, 0.40]	0.57 [0.45, 0.68]	0.12 [-0.06, 0.31]	0.49 [0.14, 0.86]	0.65 [0.33, 1.00]	0.16 [0.08, 0.33]	0.93 [0.89, 1.00]
lr3e-05_wd0.0_h256_d2	0.77 [0.67, 0.81]	0.43 [0.26, 0.61]	0.21 [0.00, 0.40]	0.56 [0.50, 0.68]	0.09 [0.00, 0.31]	0.58 [0.00, 1.00]	0.53 [0.00, 1.00]	0.15 [0.00, 0.33]	0.73 [0.00, 0.93]
lr3e-05_wd0.0_h400_d2	0.74 [0.63, 0.80]	0.39 [0.18, 0.54]	0.29 [0.17, 0.57]	0.58 [0.49, 0.73]	0.16 [-0.01, 0.53]	0.53 [0.00, 0.97]	0.63 [0.50, 1.00]	0.24 [0.10, 0.67]	0.73 [0.00, 0.93]
lr3e-05_wd0.0_h512_d2	0.73 [0.60, 0.82]	0.39 [0.14, 0.63]	0.18 [0.00, 0.33]	0.55 [0.50, 0.65]	0.10 [0.00, 0.29]	0.75 [0.00, 1.00]	0.35 [0.00, 1.00]	0.17 [0.00, 0.50]	0.73 [0.00, 0.92]
lr3e-05_wd0.0001_h256_d1	0.76 [0.65, 0.83]	0.41 [0.26, 0.73]	0.23 [0.00, 0.42]	0.62 [0.48, 0.81]	0.17 [-0.06, 0.41]	0.45 [0.14, 0.96]	0.80 [0.00, 1.00]	0.14 [0.00, 0.27]	0.98 [0.90, 1.00]
lr3e-05_wd0.0001_h400_d1	0.73 [0.62, 0.84]	0.40 [0.25, 0.61]	0.25 [0.00, 0.40]	0.62 [0.48, 0.74]	0.16 [-0.06, 0.33]	0.65 [0.34, 0.96]	0.58 [0.00, 1.00]	0.17 [0.00, 0.29]	0.94 [0.90, 1.00]
lr3e-05_wd0.0001_h512_d1	0.77 [0.65, 0.91]	0.49 [0.19, 0.69]	0.23 [0.00, 0.40]	0.59 [0.50, 0.74]	0.15 [0.00, 0.33]	0.53 [0.04, 1.00]	0.65 [0.00, 1.00]	0.16 [0.00, 0.29]	0.96 [0.90, 1.00]
lr3e-05_wd0.0001_h256_d2	0.74 [0.64, 0.81]	0.40 [0.19, 0.62]	0.24 [0.00, 0.50]	0.58 [0.50, 0.72]	0.14 [0.00, 0.43]	0.56 [0.00, 1.00]	0.60 [0.00, 1.00]	0.19 [0.00, 0.50]	0.75 [0.00, 1.00]
lr3e-05_wd0.0001_h400_d2	0.71 [0.62, 0.78]	0.32 [0.17, 0.50]	0.21 [0.00, 0.29]	0.57 [0.50, 0.61]	0.11 [0.00, 0.21]	0.75 [0.39, 1.00]	0.38 [0.00, 0.67]	0.17 [0.00, 0.33]	0.91 [0.90, 0.92]
lr3e-05_wd0.0001_h512_d2	0.74 [0.63, 0.82]	0.37 [0.17, 0.55]	0.28 [0.00, 0.57]	0.61 [0.40, 0.73]	0.19 [-0.15, 0.53]	0.64 [0.14, 1.00]	0.58 [0.00, 1.00]	0.23 [0.00, 0.67]	0.92 [0.80, 1.00]
lr3e-05_wd0.001_h256_d1	0.75 [0.69, 0.80]	0.39 [0.19, 0.60]	0.21 [0.00, 0.31]	0.58 [0.50, 0.64]	0.12 [0.00, 0.21]	0.63 [0.28, 1.00]	0.53 [0.00, 1.00]	0.14 [0.00, 0.22]	0.93 [0.90, 1.00]
lr3e-05_wd0.001_h400_d1	0.73 [0.64, 0.80]	0.36 [0.19, 0.53]	0.25 [0.00, 0.50]	0.60 [0.48, 0.72]	0.16 [-0.06, 0.43]	0.65 [0.11, 0.96]	0.55 [0.00, 1.00]	0.20 [0.00, 0.50]	0.94 [0.89, 1.00]
lr3e-05_wd0.001_h512_d1	0.75 [0.68, 0.80]	0.41 [0.20, 0.52]	0.26 [0.00, 0.44]	0.61 [0.48, 0.74]	0.17 [-0.06, 0.36]	0.57 [0.07, 0.96]	0.65 [0.00, 1.00]	0.18 [0.00, 0.40]	0.96 [0.90, 1.00]
lr3e-05_wd0.001_h256_d2	0.74 [0.61, 0.80]	0.44 [0.27, 0.62]	0.25 [0.14, 0.44]	0.55 [0.42, 0.70]	0.08 [-0.12, 0.36]	0.43 [0.00, 0.90]	0.67 [0.50, 1.00]	0.18 [0.08, 0.40]	0.72 [0.00, 0.93]
lr3e-05_wd0.001_h400_d2	0.76 [0.67, 0.80]	0.41 [0.25, 0.54]	0.20 [0.00, 0.33]	0.53 [0.42, 0.63]	0.07 [-0.12, 0.29]	0.58 [0.00, 1.00]	0.48 [0.00, 1.00]	0.18 [0.00, 0.50]	0.71 [0.00, 0.92]
lr3e-05_wd0.001_h512_d2	0.73 [0.65, 0.78]	0.34 [0.18, 0.49]	0.22 [0.00, 0.31]	0.59 [0.50, 0.64]	0.13 [0.00, 0.21]	0.79 [0.61, 1.00]	0.38 [0.00, 0.67]	0.18 [0.00, 0.33]	0.91 [0.90, 0.94]
lr0.0001_wd0.005_h256_d1	0.75 [0.67, 0.82]	0.42 [0.26, 0.56]	0.25 [0.16, 0.33]	0.58 [0.48, 0.65]	0.13 [-0.03, 0.22]	0.61 [0.28, 0.93]	0.55 [0.25, 1.00]	0.19 [0.09, 0.33]	0.92 [0.89, 1.00]
lr0.0001_wd0.005_h400_d1	0.74 [0.64, 0.82]	0.40 [0.19, 0.63]	0.28 [0.18, 0.33]	0.61 [0.53, 0.72]	0.19 [0.04, 0.30]	0.68 [0.39, 0.97]	0.55 [0.25, 1.00]	0.24 [0.11, 0.50]	0.93 [0.90, 1.00]
lr0.0001_wd0.005_h512_d1	0.73 [0.61, 0.83]	0.37 [0.21, 0.53]	0.20 [0.00, 0.36]	0.58 [0.46, 0.69]	0.13 [-0.09, 0.26]	0.86 [0.62, 1.00]	0.30 [0.00, 0.75]	0.17 [0.00, 0.33]	0.91 [0.90, 0.95]
lr0.0001_wd0.005_h256_d2	0.75 [0.68, 0.81]	0.47 [0.22, 0.62]	0.28 [0.22, 0.35]	0.61 [0.50, 0.70]	0.19 [0.00, 0.29]	0.56 [0.00, 0.97]	0.67 [0.25, 1.00]	0.25 [0.12, 0.50]	0.76 [0.00, 1.00]
lr0.0001_wd0.005_h400_d2	0.76 [0.64, 0.84]	0.36 [0.17, 0.64]	0.20 [0.00, 0.40]	0.57 [0.50, 0.68]	0.14 [0.00, 0.31]	0.90 [0.66, 1.00]	0.25 [0.00, 0.50]	0.20 [0.00, 0.50]	0.91 [0.90, 0.93]
lr0.0001_wd0.005_h512_d2	0.76 [0.65, 0.82]	0.43 [0.25, 0.51]	0.30 [0.21, 0.44]	0.62 [0.58, 0.70]	0.21 [0.10, 0.36]	0.79 [0.50, 0.97]	0.45 [0.25, 0.67]	0.28 [0.12, 0.50]	0.92 [0.90, 0.93]
lr0.0001_wd0.01_h256_d1	0.72 [0.61, 0.83]	0.42 [0.21, 0.56]	0.28 [0.18, 0.33]	0.61 [0.53, 0.65]	0.19 [0.04, 0.26]	0.66 [0.28, 0.93]	0.55 [0.25, 1.00]	0.24 [0.11, 0.33]	0.93 [0.90, 1.00]
lr0.0001_wd0.01_h400_d1	0.73 [0.61, 0.82]	0.43 [0.20, 0.63]	0.24 [0.18, 0.31]	0.57 [0.52, 0.63]	0.11 [0.06, 0.21]	0.58 [0.03, 0.93]	0.55 [0.25, 1.00]	0.18 [0.11, 0.33]	0.93 [0.90, 1.00]

Supplementary Table 19: Grid search results on the classification task on GDHCM retrospective dataset (choosing 40% as the threshold for clinical variables selection). Reported metrics are AUC, AUPRC, F1, Balanced Accuracy, MCC, Specificity, Sensitivity, PPV, and NPV.

Config	AUC	AUPRC	F1	Balanced_Accuracy	MCC	Specificity	Sensitivity	PPV	NPV
lr0.0001_wd0.01_h512_d1	0.76 [0.65, 0.83]	0.47 [0.27, 0.55]	0.21 [0.00, 0.33]	0.55 [0.48, 0.63]	0.10 [-0.06, 0.29]	0.61 [0.03, 0.97]	0.48 [0.00, 1.00]	0.19 [0.00, 0.50]	0.92 [0.90, 1.00]
lr0.0001_wd0.01_h256_d2	0.76 [0.64, 0.84]	0.40 [0.17, 0.64]	0.26 [0.00, 0.44]	0.61 [0.50, 0.70]	0.21 [0.00, 0.36]	0.67 [0.11, 1.00]	0.55 [0.00, 1.00]	0.24 [0.00, 0.50]	0.95 [0.90, 1.00]
lr0.0001_wd0.01_h400_d2	0.75 [0.65, 0.83]	0.37 [0.18, 0.63]	0.27 [0.00, 0.57]	0.61 [0.50, 0.73]	0.19 [0.00, 0.53]	0.83 [0.54, 1.00]	0.38 [0.00, 0.67]	0.25 [0.00, 0.67]	0.92 [0.89, 0.94]
lr0.0001_wd0.01_h512_d2	0.74 [0.64, 0.83]	0.40 [0.21, 0.58]	0.24 [0.00, 0.40]	0.59 [0.50, 0.68]	0.16 [0.00, 0.31]	0.80 [0.43, 1.00]	0.38 [0.00, 0.67]	0.23 [0.00, 0.50]	0.91 [0.90, 0.93]
lr0.0003_wd0.005_h256_d1	0.77 [0.67, 0.84]	0.42 [0.21, 0.53]	0.27 [0.18, 0.40]	0.59 [0.50, 0.68]	0.17 [0.00, 0.31]	0.50 [0.00, 0.97]	0.68 [0.25, 1.00]	0.24 [0.11, 0.50]	0.75 [0.00, 1.00]
lr0.0003_wd0.005_h400_d1	0.77 [0.65, 0.87]	0.48 [0.26, 0.59]	0.29 [0.16, 0.44]	0.60 [0.48, 0.70]	0.17 [-0.03, 0.36]	0.53 [0.17, 0.90]	0.67 [0.50, 1.00]	0.22 [0.09, 0.40]	0.93 [0.89, 1.00]
lr0.0003_wd0.005_h512_d1	0.77 [0.68, 0.84]	0.41 [0.22, 0.64]	0.25 [0.17, 0.33]	0.58 [0.49, 0.65]	0.12 [-0.01, 0.22]	0.70 [0.32, 0.90]	0.45 [0.25, 0.67]	0.19 [0.10, 0.25]	0.91 [0.90, 0.92]
lr0.0003_wd0.005_h256_d2	0.74 [0.61, 0.84]	0.39 [0.16, 0.63]	0.16 [0.00, 0.35]	0.56 [0.48, 0.70]	0.07 [-0.07, 0.27]	0.67 [0.00, 1.00]	0.45 [0.00, 1.00]	0.11 [0.00, 0.23]	0.73 [0.00, 0.95]
lr0.0003_wd0.005_h400_d2	0.76 [0.63, 0.85]	0.42 [0.37, 0.48]	0.33 [0.15, 0.50]	0.61 [0.46, 0.72]	0.21 [-0.06, 0.43]	0.72 [0.25, 0.93]	0.50 [0.33, 0.67]	0.28 [0.09, 0.50]	0.91 [0.88, 0.93]
lr0.0003_wd0.005_h512_d2	0.78 [0.69, 0.85]	0.49 [0.32, 0.63]	0.30 [0.19, 0.40]	0.61 [0.54, 0.68]	0.21 [0.09, 0.31]	0.71 [0.07, 0.93]	0.52 [0.25, 1.00]	0.27 [0.10, 0.33]	0.93 [0.90, 1.00]
lr0.0003_wd0.01_h256_d1	0.75 [0.67, 0.84]	0.39 [0.28, 0.53]	0.25 [0.13, 0.36]	0.57 [0.47, 0.66]	0.12 [-0.04, 0.26]	0.68 [0.29, 0.93]	0.45 [0.25, 0.67]	0.20 [0.08, 0.33]	0.90 [0.89, 0.92]
lr0.0003_wd0.01_h400_d1	0.75 [0.64, 0.82]	0.46 [0.38, 0.53]	0.30 [0.15, 0.40]	0.60 [0.46, 0.68]	0.18 [-0.06, 0.31]	0.75 [0.25, 0.93]	0.45 [0.25, 0.67]	0.26 [0.09, 0.33]	0.91 [0.88, 0.93]
lr0.0003_wd0.01_h512_d1	0.75 [0.61, 0.81]	0.47 [0.24, 0.62]	0.32 [0.22, 0.46]	0.66 [0.58, 0.77]	0.23 [0.12, 0.40]	0.77 [0.68, 0.82]	0.55 [0.33, 0.75]	0.23 [0.17, 0.33]	0.93 [0.92, 0.96]
lr0.0003_wd0.01_h256_d2	0.77 [0.68, 0.83]	0.45 [0.31, 0.51]	0.24 [0.00, 0.33]	0.62 [0.48, 0.71]	0.15 [-0.07, 0.27]	0.77 [0.54, 0.97]	0.47 [0.00, 0.67]	0.17 [0.00, 0.25]	0.92 [0.88, 0.95]
lr0.0003_wd0.01_h400_d2	0.76 [0.62, 0.84]	0.39 [0.23, 0.64]	0.28 [0.00, 0.50]	0.61 [0.48, 0.78]	0.20 [-0.07, 0.45]	0.74 [0.07, 0.97]	0.48 [0.00, 1.00]	0.23 [0.00, 0.40]	0.93 [0.88, 1.00]
lr0.0003_wd0.01_h512_d2	0.76 [0.68, 0.84]	0.44 [0.34, 0.57]	0.25 [0.00, 0.44]	0.62 [0.48, 0.74]	0.18 [-0.07, 0.36]	0.68 [0.18, 0.97]	0.57 [0.00, 1.00]	0.19 [0.00, 0.40]	0.95 [0.88, 1.00]
lr0.001_wd0.005_h256_d1	0.82 [0.65, 0.90]	0.49 [0.36, 0.62]	0.32 [0.25, 0.40]	0.66 [0.60, 0.74]	0.26 [0.15, 0.32]	0.71 [0.39, 0.97]	0.62 [0.25, 1.00]	0.28 [0.15, 0.50]	0.95 [0.90, 1.00]
lr0.001_wd0.005_h400_d1	0.75 [0.62, 0.87]	0.43 [0.22, 0.57]	0.33 [0.20, 0.40]	0.66 [0.57, 0.78]	0.25 [0.08, 0.36]	0.68 [0.46, 0.93]	0.65 [0.33, 1.00]	0.24 [0.12, 0.33]	0.95 [0.93, 1.00]
lr0.001_wd0.005_h512_d1	0.75 [0.62, 0.84]	0.38 [0.16, 0.51]	0.22 [0.00, 0.50]	0.58 [0.42, 0.86]	0.10 [-0.12, 0.49]	0.60 [0.18, 0.97]	0.57 [0.00, 1.00]	0.14 [0.00, 0.33]	0.91 [0.83, 1.00]
lr0.001_wd0.005_h256_d2	0.80 [0.70, 0.84]	0.50 [0.35, 0.68]	0.37 [0.19, 0.46]	0.67 [0.55, 0.77]	0.28 [0.06, 0.40]	0.78 [0.43, 0.93]	0.57 [0.25, 0.75]	0.30 [0.11, 0.40]	0.93 [0.90, 0.96]
lr0.001_wd0.005_h400_d2	0.76 [0.64, 0.84]	0.49 [0.37, 0.61]	0.29 [0.00, 0.44]	0.63 [0.48, 0.74]	0.21 [-0.07, 0.36]	0.87 [0.76, 0.97]	0.40 [0.00, 0.67]	0.23 [0.00, 0.40]	0.92 [0.88, 0.96]
lr0.001_wd0.005_h512_d2	0.75 [0.61, 0.86]	0.31 [0.16, 0.45]	0.17 [0.00, 0.40]	0.55 [0.48, 0.68]	0.07 [-0.07, 0.31]	0.77 [0.29, 0.97]	0.33 [0.00, 0.67]	0.13 [0.00, 0.33]	0.90 [0.88, 0.93]
lr0.001_wd0.01_h256_d1	0.75 [0.62, 0.84]	0.42 [0.15, 0.59]	0.30 [0.17, 0.44]	0.62 [0.49, 0.79]	0.21 [-0.01, 0.38]	0.70 [0.32, 0.97]	0.55 [0.25, 1.00]	0.27 [0.10, 0.50]	0.93 [0.90, 1.00]
lr0.001_wd0.01_h400_d1	0.74 [0.61, 0.84]	0.37 [0.21, 0.59]	0.27 [0.15, 0.36]	0.58 [0.44, 0.66]	0.13 [-0.08, 0.26]	0.65 [0.21, 0.90]	0.52 [0.25, 0.67]	0.20 [0.08, 0.29]	0.91 [0.86, 0.93]
lr0.001_wd0.01_h512_d1	0.78 [0.70, 0.86]	0.42 [0.32, 0.48]	0.25 [0.00, 0.44]	0.61 [0.48, 0.70]	0.16 [-0.07, 0.36]	0.82 [0.71, 0.97]	0.40 [0.00, 0.67]	0.19 [0.00, 0.40]	0.92 [0.88, 0.95]
lr0.001_wd0.01_h256_d2	0.79 [0.70, 0.85]	0.51 [0.37, 0.60]	0.36 [0.25, 0.50]	0.68 [0.62, 0.79]	0.31 [0.16, 0.48]	0.80 [0.61, 1.00]	0.57 [0.25, 0.75]	0.40 [0.15, 1.00]	0.94 [0.91, 0.96]
lr0.001_wd0.01_h400_d2	0.80 [0.71, 0.84]	0.40 [0.33, 0.56]	0.27 [0.00, 0.44]	0.63 [0.48, 0.70]	0.19 [-0.07, 0.36]	0.79 [0.61, 0.97]	0.47 [0.00, 0.67]	0.21 [0.00, 0.40]	0.92 [0.88, 0.95]
lr0.001_wd0.01_h512_d2	0.77 [0.69, 0.85]	0.48 [0.26, 0.70]	0.42 [0.25, 0.55]	0.72 [0.64, 0.81]	0.35 [0.16, 0.49]	0.82 [0.61, 0.93]	0.62 [0.50, 0.75]	0.35 [0.15, 0.50]	0.94 [0.93, 0.96]
lr0.0001_wd0.0_h1024_d1	0.73 [0.61, 0.83]	0.42 [0.18, 0.55]	0.29 [0.20, 0.40]	0.61 [0.57, 0.68]	0.19 [0.11, 0.31]	0.66 [0.14, 0.89]	0.57 [0.33, 1.00]	0.22 [0.11, 0.33]	0.93 [0.90, 1.00]
lr0.0001_wd0.0_h2048_d1	0.76 [0.65, 0.81]	0.40 [0.30, 0.51]	0.28 [0.24, 0.36]	0.63 [0.59, 0.66]	0.20 [0.14, 0.26]	0.68 [0.32, 0.93]	0.58 [0.25, 1.00]	0.22 [0.14, 0.33]	0.94 [0.90, 1.00]
lr0.0001_wd0.0_h1024_d2	0.77 [0.71, 0.83]	0.46 [0.34, 0.59]	0.22 [0.16, 0.33]	0.53 [0.48, 0.61]	0.07 [-0.03, 0.29]	0.49 [0.00, 0.97]	0.57 [0.25, 1.00]	0.20 [0.09, 0.50]	0.72 [0.00, 0.90]
lr0.0001_wd0.0_h2048_d2	0.75 [0.61, 0.85]	0.45 [0.28, 0.65]	0.19 [0.00, 0.40]	0.54 [0.48, 0.68]	0.06 [-0.07, 0.31]	0.52 [0.00, 0.97]	0.57 [0.00, 1.00]	0.13 [0.00, 0.33]	0.73 [0.00, 0.93]
lr0.0001_wd0.0001_h1024_d1	0.72 [0.61, 0.84]	0.42 [0.21, 0.61]	0.23 [0.00, 0.40]	0.57 [0.46, 0.74]	0.12 [-0.09, 0.33]	0.71 [0.32, 0.97]	0.43 [0.00, 0.75]	0.20 [0.00, 0.50]	0.91 [0.90, 0.95]
lr0.0001_wd0.0001_h2048_d1	0.77 [0.62, 0.84]	0.45 [0.38, 0.55]	0.29 [0.22, 0.33]	0.63 [0.60, 0.72]	0.22 [0.12, 0.30]	0.72 [0.45, 0.97]	0.55 [0.25, 1.00]	0.26 [0.13, 0.50]	0.94 [0.90, 1.00]
lr0.0001_wd0.0001_h1024_d2	0.76 [0.65, 0.84]	0.47 [0.24, 0.65]	0.32 [0.25, 0.44]	0.63 [0.60, 0.70]	0.22 [0.15, 0.36]	0.83 [0.76, 0.90]	0.43 [0.33, 0.50]	0.26 [0.20, 0.40]	0.92 [0.92, 0.93]
lr0.0001_wd0.0001_h2048_d2	0.76 [0.68, 0.85]	0.47 [0.34, 0.62]	0.29 [0.00, 0.50]	0.61 [0.48, 0.70]	0.22 [-0.07, 0.56]	0.81 [0.46, 1.00]	0.40 [0.00, 0.67]	0.34 [0.00, 1.00]	0.92 [0.88, 0.93]
lr0.0001_wd0.001_h1024_d1	0.73 [0.62, 0.82]	0.42 [0.30, 0.55]	0.27 [0.12, 0.40]	0.57 [0.43, 0.68]	0.14 [-0.08, 0.31]	0.68 [0.25, 0.97]	0.45 [0.25, 0.67]	0.25 [0.07, 0.50]	0.90 [0.88, 0.93]
lr0.0001_wd0.001_h2048_d1	0.75 [0.65, 0.82]	0.44 [0.27, 0.55]	0.26 [0.00, 0.40]	0.61 [0.50, 0.70]	0.19 [0.00, 0.31]	0.80 [0.50, 1.00]	0.43 [0.00, 0.75]	0.24 [0.00, 0.50]	0.92 [0.90, 0.95]
lr0.0001_wd0.001_h1024_d2	0.75 [0.62, 0.84]	0.50 [0.42, 0.60]	0.28 [0.11, 0.44]	0.59 [0.42, 0.70]	0.18 [-0.10, 0.36]	0.67 [0.29, 0.97]	0.52 [0.25, 1.00]	0.26 [0.07, 0.50]	0.92 [0.88, 1.00]
lr0.0001_wd0.001_h2048_d2	0.76 [0.62, 0.84]	0.41 [0.30, 0.65]	0.20 [0.00, 0.40]	0.54 [0.39, 0.68]	0.05 [-0.20, 0.31]	0.51 [0.11, 0.97]	0.57 [0.00, 1.00]	0.13 [0.00, 0.33]	0.89 [0.75, 1.00]
lr0.0003_wd0.0_h1024_d1	0.74 [0.57, 0.82]	0.45 [0.14, 0.61]	0.27 [0.14, 0.44]	0.59 [0.49, 0.70]	0.16 [-0.01, 0.36]	0.80 [0.64, 0.93]	0.38 [0.25, 0.50]	0.23 [0.09, 0.40]	0.91 [0.90, 0.93]
lr0.0003_wd0.0_h2048_d1	0.72 [0.60, 0.81]	0.43 [0.26, 0.54]	0.23 [0.00, 0.44]	0.59 [0.48, 0.70]	0.13 [-0.07, 0.36]	0.72 [0.46, 0.97]	0.47 [0.00, 0.67]	0.17 [0.00, 0.40]	0.92 [0.88, 0.93]
lr0.0003_wd0.0_h1024_d2	0.77 [0.65, 0.84]	0.55 [0.43, 0.67]	0.30 [0.17, 0.40]	0.63 [0.49, 0.72]	0.19 [-0.01, 0.31]	0.66 [0.32, 0.86]	0.60 [0.33, 0.75]	0.21 [0.10, 0.33]	0.93 [0.90, 0.95]
lr0.0003_wd0.0_h2048_d2	0.78 [0.67, 0.89]	0.38 [0.30, 0.50]	0.32 [0.00, 0.50]	0.68 [0.47, 0.78]	0.26 [-0.09, 0.45]	0.78 [0.46, 0.93]	0.58 [0.00, 1.00]	0.24 [0.00, 0.40]	0.94 [0.87, 1.00]
lr0.0003_wd0.0001_h1024_d1	0.76 [0.67, 0.84]	0.40 [0.18, 0.65]	0.33 [0.17, 0.57]	0.65 [0.51, 0.73]	0.25 [0.01, 0.53]	0.63 [0.36, 0.97]	0.67 [0.50, 1.00]	0.28 [0.10, 0.67]	0.94 [0.91, 1.00]
lr0.0003_wd0.0001_h2048_d1	0.78 [0.70, 0.84]	0.49 [0.29, 0.57]	0.33 [0.24, 0.50]	0.65 [0.61, 0.72]	0.25 [0.14, 0.43]	0.73 [0.57, 0.97]	0.57 [0.25, 0.75]	0.30 [0.14, 0.50]	0.93 [0.90, 0.95]
lr0.0003_wd0.0001_h1024_d2	0.78 [0.68, 0.86]	0.47 [0.34, 0.70]	0.27 [0.00, 0.50]	0.62 [0.47, 0.79]	0.17 [-0.09, 0.44]	0.72 [0.43, 0.93]	0.52 [0.00, 0.75]	0.20 [0.00, 0.40]	0.92 [0.87, 0.96]
lr0.0003_wd0.0001_h2048_d2	0.79 [0.65, 0.85]	0.42 [0.26, 0.55]	0.24 [0.00, 0.33]	0.64 [0.50, 0.71]	0.18 [0.00, 0.28]	0.71 [0.41, 1.00]	0.57 [0.00, 1.00]	0.16 [0.00, 0.22]	0.94 [0.88, 1.00]
lr0.0003_wd0.001_h1024_d1	0.78 [0.69, 0.84]	0.43 [0.21, 0.55]	0.33 [0.27, 0.38]	0.69 [0.65, 0.74]	0.26 [0.19, 0.32]	0.66 [0.48, 0.83]	0.72 [0.50, 1.00]	0.22 [0.17, 0.29]	0.95 [0.92, 1.00]
lr0.0003_wd0.001_h2048_d1	0.77 [0.63, 0.84]	0.43 [0.21, 0.64]	0.29 [0.22, 0.35]	0.62 [0.56, 0.70]	0.19 [0.09, 0.29]	0.69 [0.38, 0.97]	0.55 [0.25, 0.75]	0.25 [0.13, 0.50]	0.93 [0.90, 0.95]
lr0.0003_wd0.001_h1024_d2	0.77 [0.65, 0.85]	0.50 [0.22, 0.66]	0.39 [0.33, 0.44]	0.73 [0.68, 0.76]	0.33 [0.30, 0.39]	0.74 [0.45, 0.86]	0.72 [0.50, 1.00]	0.28 [0.20, 0.33]	0.96 [0.93, 1.00]
lr0.0003_wd0.001_h2048_d2	0.79 [0.70, 0.87]	0.45 [0.32, 0.55]	0.36 [0.21, 0.50]	0.67 [0.58, 0.75]	0.28 [0.10, 0.43]	0.78 [0.50, 0.93]	0.55 [0.33, 0.75]	0.30 [0.12, 0.50]	0.93 [0.92, 0.96]
lr0.001_wd0.0_h1024_d1	0.77 [0.62, 0.93]	0.46 [0.36, 0.53]	0.23 [0.00, 0.44]	0.59 [0.47, 0.70]	0.13 [-0.09, 0.36]	0.67 [0.28, 0.93]	0.50 [0.00, 1.00]	0.17 [0.00, 0.40]	0.93 [0.87, 1.00]
lr0.001_wd0.0_h2048_d1	0.76 [0.64, 0.85]	0.35 [0.19, 0.51]	0.20 [0.00, 0.40]	0.56 [0.45, 0.68]	0.09 [-0.12, 0.31]	0.61 [0.21, 0.90]	0.50 [0.00, 1.00]	0.14 [0.00, 0.33]	0.92 [0.87, 1.00]
lr0.001_wd0.0_h1024_d2	0.77 [0.67, 0.86]	0.41 [0.19, 0.55]	0.26 [0.00, 0.60]	0.61 [0.48, 0.82]	0.16 [-0.07, 0.55]	0.64 [0.00, 0.97]	0.58 [0.00, 1.00]	0.19 [0.00, 0.50]	0.74 [0.00, 0.96]
lr0.001_wd0.0_h2048_d2	0.78 [0.69, 0.86]	0.42 [0.23, 0.62]	0.27 [0.00, 0.50]	0.61 [0.48, 0.72]	0.17 [-0.07, 0.43]	0.69 [0.00, 0.97]	0.53 [0.00, 1.00]	0.22 [0.00, 0.50]	0.74 [0.00, 0.95]
lr0.001_wd0.0001_h1024_d1	0.77 [0.60, 0.85]	0.47 [0.15, 0.71]	0.34 [0.18, 0.50]	0.67 [0.53, 0.78]	0.25 [0.04, 0.45]	0.66 [0.39, 0.89]	0.67 [0.50, 0.75]	0.25 [0.11, 0.40]	0.94 [0.92, 0.96]
lr0.001_wd0.0001_h2048_d1	0.75 [0.62, 0.85]	0.40 [0.35, 0.43]	0.31 [0.25, 0.40]	0.61 [0.57, 0.65]	0.23 [0.15, 0.36]	0.89 [0.72, 0.96]	0.33 [0.25, 0.50]	0.32 [0.20, 0.50]	0.91 [0.90, 0.93]
lr0.001_wd0.0001_h1024_d2	0.80 [0.65, 0.91]	0.49 [0.22, 0.82]	0.27 [0.00, 0.50]	0.62 [0.48, 0.74]	0.18 [-0.07, 0.43]	0.80 [0.57, 0.97]	0.45 [0.00, 0.75]	0.21 [0.00, 0.50]	0.92 [0.88, 0.95]

Supplementary Table 20: Grid search results on the classification task on GDHCM retrospective dataset (choosing 40% as the threshold for clinical variables selection). Reported metrics are AUC, AUPRC, F1, Balanced Accuracy, MCC, Specificity, Sensitivity, PPV, and NPV.

Config	AUC	AUPRC	F1	Balanced_Accuracy	MCC	Specificity	Sensitivity	PPV	NPV
lr0.001_wd0.0001_h2048_d2	0.76 [0.65, 0.85]	0.39 [0.23, 0.55]	0.24 [0.00, 0.36]	0.59 [0.45, 0.66]	0.14 [-0.11, 0.26]	0.80 [0.57, 0.93]	0.38 [0.00, 0.67]	0.20 [0.00, 0.33]	0.91 [0.89, 0.94]
lr0.001_wd0.001_h1024_d1	0.74 [0.62, 0.83]	0.37 [0.21, 0.52]	0.28 [0.00, 0.43]	0.64 [0.47, 0.81]	0.20 [-0.06, 0.41]	0.76 [0.61, 0.96]	0.52 [0.00, 1.00]	0.20 [0.00, 0.33]	0.94 [0.89, 1.00]
lr0.001_wd0.001_h2048_d1	0.74 [0.68, 0.83]	0.38 [0.29, 0.51]	0.31 [0.25, 0.44]	0.63 [0.58, 0.70]	0.21 [0.11, 0.36]	0.71 [0.41, 0.93]	0.55 [0.33, 0.75]	0.25 [0.15, 0.40]	0.93 [0.91, 0.95]
lr0.001_wd0.001_h1024_d2	0.82 [0.80, 0.84]	0.48 [0.38, 0.61]	0.21 [0.00, 0.36]	0.59 [0.48, 0.73]	0.11 [-0.07, 0.31]	0.76 [0.50, 0.97]	0.42 [0.00, 0.67]	0.15 [0.00, 0.25]	0.91 [0.88, 0.96]
lr0.001_wd0.001_h2048_d2	0.81 [0.69, 0.92]	0.48 [0.20, 0.59]	0.45 [0.31, 0.60]	0.74 [0.69, 0.82]	0.40 [0.24, 0.55]	0.80 [0.54, 0.97]	0.68 [0.50, 1.00]	0.39 [0.19, 0.67]	0.96 [0.93, 1.00]
lr0.0001_wd0.0_h256_d3	0.76 [0.67, 0.83]	0.40 [0.17, 0.61]	0.23 [0.00, 0.33]	0.59 [0.50, 0.65]	0.16 [0.00, 0.29]	0.80 [0.46, 1.00]	0.38 [0.00, 0.67]	0.22 [0.00, 0.50]	0.91 [0.90, 0.93]
lr0.0001_wd0.0_h400_d3	0.76 [0.65, 0.82]	0.34 [0.19, 0.50]	0.25 [0.00, 0.40]	0.60 [0.50, 0.68]	0.18 [0.00, 0.31]	0.82 [0.46, 1.00]	0.38 [0.00, 0.67]	0.24 [0.00, 0.50]	0.92 [0.90, 0.93]
lr0.0001_wd0.0_h512_d3	0.77 [0.67, 0.84]	0.40 [0.20, 0.63]	0.21 [0.00, 0.33]	0.56 [0.50, 0.61]	0.11 [0.00, 0.29]	0.63 [0.00, 1.00]	0.48 [0.00, 1.00]	0.19 [0.00, 0.50]	0.73 [0.00, 0.93]
lr0.0001_wd0.0_h256_d4	0.76 [0.68, 0.81]	0.48 [0.18, 0.62]	0.22 [0.00, 0.33]	0.58 [0.49, 0.65]	0.11 [-0.01, 0.22]	0.72 [0.32, 1.00]	0.43 [0.00, 0.67]	0.15 [0.00, 0.25]	0.91 [0.90, 0.92]
lr0.0001_wd0.0_h400_d4	0.77 [0.67, 0.84]	0.46 [0.36, 0.64]	0.26 [0.00, 0.40]	0.61 [0.50, 0.79]	0.17 [0.00, 0.38]	0.66 [0.00, 1.00]	0.57 [0.00, 1.00]	0.19 [0.00, 0.33]	0.75 [0.00, 1.00]
lr0.0001_wd0.0_h512_d4	0.76 [0.64, 0.84]	0.38 [0.18, 0.57]	0.26 [0.00, 0.44]	0.59 [0.50, 0.70]	0.17 [0.00, 0.36]	0.74 [0.00, 1.00]	0.45 [0.00, 1.00]	0.23 [0.00, 0.40]	0.73 [0.00, 0.93]
lr0.0001_wd0.0001_h256_d3	0.75 [0.67, 0.81]	0.42 [0.24, 0.55]	0.17 [0.00, 0.40]	0.56 [0.48, 0.68]	0.08 [-0.07, 0.31]	0.78 [0.43, 1.00]	0.33 [0.00, 0.67]	0.12 [0.00, 0.33]	0.91 [0.88, 0.93]
lr0.0001_wd0.0001_h400_d3	0.78 [0.70, 0.84]	0.40 [0.22, 0.64]	0.18 [0.00, 0.40]	0.57 [0.48, 0.68]	0.11 [-0.07, 0.31]	0.75 [0.14, 1.00]	0.40 [0.00, 1.00]	0.13 [0.00, 0.33]	0.92 [0.88, 1.00]
lr0.0001_wd0.0001_h512_d3	0.76 [0.67, 0.84]	0.37 [0.23, 0.53]	0.30 [0.22, 0.44]	0.61 [0.50, 0.70]	0.19 [0.00, 0.36]	0.67 [0.00, 0.93]	0.55 [0.25, 1.00]	0.25 [0.12, 0.40]	0.74 [0.00, 0.94]
lr0.0001_wd0.0001_h256_d4	0.75 [0.68, 0.84]	0.39 [0.25, 0.46]	0.28 [0.21, 0.33]	0.60 [0.58, 0.63]	0.18 [0.10, 0.29]	0.75 [0.50, 0.97]	0.45 [0.25, 0.67]	0.25 [0.13, 0.50]	0.92 [0.90, 0.93]
lr0.0001_wd0.0001_h400_d4	0.76 [0.65, 0.84]	0.36 [0.18, 0.54]	0.24 [0.00, 0.40]	0.58 [0.50, 0.68]	0.15 [0.00, 0.31]	0.68 [0.00, 1.00]	0.48 [0.00, 1.00]	0.22 [0.00, 0.50]	0.73 [0.00, 0.94]
lr0.0001_wd0.0001_h512_d4	0.75 [0.65, 0.84]	0.38 [0.19, 0.51]	0.39 [0.22, 0.50]	0.66 [0.50, 0.72]	0.30 [0.00, 0.43]	0.72 [0.00, 0.96]	0.60 [0.33, 1.00]	0.37 [0.12, 0.50]	0.75 [0.00, 0.95]
lr0.0001_wd0.001_h256_d3	0.74 [0.63, 0.81]	0.47 [0.22, 0.62]	0.29 [0.21, 0.40]	0.60 [0.50, 0.65]	0.18 [0.00, 0.36]	0.53 [0.00, 0.96]	0.67 [0.33, 1.00]	0.24 [0.12, 0.50]	0.75 [0.00, 1.00]
lr0.0001_wd0.001_h400_d3	0.75 [0.60, 0.84]	0.37 [0.17, 0.63]	0.17 [0.00, 0.36]	0.55 [0.48, 0.66]	0.08 [-0.07, 0.26]	0.77 [0.32, 1.00]	0.33 [0.00, 0.67]	0.12 [0.00, 0.29]	0.90 [0.88, 0.92]
lr0.0001_wd0.001_h512_d3	0.76 [0.68, 0.83]	0.37 [0.18, 0.55]	0.26 [0.00, 0.40]	0.61 [0.50, 0.68]	0.19 [0.00, 0.31]	0.84 [0.61, 1.00]	0.38 [0.00, 0.67]	0.24 [0.00, 0.50]	0.92 [0.90, 0.94]
lr0.0001_wd0.001_h256_d4	0.77 [0.68, 0.85]	0.36 [0.20, 0.56]	0.17 [0.00, 0.40]	0.55 [0.48, 0.68]	0.07 [-0.07, 0.31]	0.76 [0.29, 1.00]	0.33 [0.00, 0.67]	0.12 [0.00, 0.33]	0.90 [0.88, 0.93]
lr0.0001_wd0.001_h400_d4	0.76 [0.62, 0.85]	0.39 [0.17, 0.63]	0.27 [0.00, 0.44]	0.62 [0.50, 0.72]	0.21 [0.00, 0.36]	0.80 [0.46, 1.00]	0.43 [0.00, 0.75]	0.25 [0.00, 0.50]	0.92 [0.90, 0.95]
lr0.0001_wd0.001_h512_d4	0.77 [0.69, 0.82]	0.35 [0.18, 0.54]	0.20 [0.00, 0.44]	0.56 [0.45, 0.70]	0.13 [-0.11, 0.36]	0.92 [0.86, 1.00]	0.20 [0.00, 0.50]	0.22 [0.00, 0.50]	0.90 [0.89, 0.93]
lr0.0003_wd0.0_h256_d3	0.74 [0.62, 0.82]	0.36 [0.16, 0.53]	0.19 [0.00, 0.44]	0.54 [0.38, 0.70]	0.07 [-0.18, 0.36]	0.75 [0.29, 0.97]	0.33 [0.00, 0.67]	0.16 [0.00, 0.40]	0.90 [0.88, 0.93]
lr0.0003_wd0.0_h400_d3	0.76 [0.63, 0.84]	0.36 [0.23, 0.43]	0.29 [0.14, 0.44]	0.60 [0.49, 0.70]	0.18 [-0.01, 0.36]	0.75 [0.46, 0.93]	0.45 [0.25, 0.67]	0.25 [0.09, 0.40]	0.92 [0.90, 0.93]
lr0.0003_wd0.0_h512_d3	0.77 [0.64, 0.86]	0.38 [0.26, 0.44]	0.29 [0.18, 0.44]	0.60 [0.50, 0.70]	0.19 [0.00, 0.36]	0.62 [0.00, 0.97]	0.58 [0.25, 1.00]	0.27 [0.10, 0.50]	0.74 [0.00, 0.93]
lr0.0003_wd0.0_h256_d4	0.77 [0.65, 0.82]	0.31 [0.23, 0.40]	0.27 [0.00, 0.44]	0.64 [0.48, 0.71]	0.20 [-0.07, 0.36]	0.72 [0.38, 0.97]	0.57 [0.00, 1.00]	0.19 [0.00, 0.40]	0.94 [0.88, 1.00]
lr0.0003_wd0.0_h400_d4	0.78 [0.70, 0.86]	0.44 [0.19, 0.71]	0.30 [0.00, 0.44]	0.66 [0.48, 0.76]	0.24 [-0.07, 0.39]	0.75 [0.29, 0.97]	0.58 [0.00, 1.00]	0.23 [0.00, 0.40]	0.94 [0.88, 1.00]
lr0.0003_wd0.0_h512_d4	0.78 [0.62, 0.90]	0.41 [0.29, 0.55]	0.24 [0.00, 0.47]	0.59 [0.45, 0.84]	0.13 [-0.12, 0.46]	0.75 [0.57, 0.90]	0.43 [0.00, 1.00]	0.17 [0.00, 0.33]	0.92 [0.87, 1.00]
lr0.0003_wd0.0001_h256_d3	0.79 [0.71, 0.86]	0.45 [0.27, 0.60]	0.25 [0.00, 0.44]	0.62 [0.48, 0.70]	0.17 [-0.07, 0.36]	0.65 [0.21, 0.97]	0.58 [0.00, 1.00]	0.18 [0.00, 0.40]	0.94 [0.88, 1.00]
lr0.0003_wd0.0001_h400_d3	0.78 [0.70, 0.84]	0.38 [0.21, 0.63]	0.26 [0.00, 0.44]	0.62 [0.48, 0.70]	0.17 [-0.07, 0.36]	0.77 [0.57, 0.97]	0.47 [0.00, 0.67]	0.20 [0.00, 0.40]	0.92 [0.88, 0.94]
lr0.0003_wd0.0001_h512_d3	0.75 [0.63, 0.83]	0.37 [0.20, 0.55]	0.27 [0.17, 0.44]	0.59 [0.52, 0.70]	0.16 [0.03, 0.36]	0.85 [0.71, 0.93]	0.33 [0.25, 0.50]	0.24 [0.11, 0.40]	0.91 [0.89, 0.93]
lr0.0003_wd0.0001_h256_d4	0.78 [0.68, 0.85]	0.33 [0.22, 0.39]	0.26 [0.00, 0.55]	0.62 [0.48, 0.81]	0.20 [-0.07, 0.49]	0.87 [0.57, 1.00]	0.38 [0.00, 0.75]	0.21 [0.00, 0.50]	0.92 [0.88, 0.96]
lr0.0003_wd0.0001_h400_d4	0.80 [0.69, 0.89]	0.48 [0.35, 0.55]	0.32 [0.18, 0.50]	0.62 [0.50, 0.72]	0.23 [0.00, 0.43]	0.66 [0.00, 0.97]	0.58 [0.25, 1.00]	0.31 [0.10, 0.50]	0.74 [0.00, 0.94]
lr0.0003_wd0.0001_h512_d4	0.78 [0.70, 0.85]	0.46 [0.24, 0.62]	0.23 [0.00, 0.40]	0.60 [0.48, 0.68]	0.13 [-0.07, 0.31]	0.63 [0.00, 0.97]	0.57 [0.00, 1.00]	0.16 [0.00, 0.33]	0.74 [0.00, 0.95]
lr0.0003_wd0.001_h256_d3	0.76 [0.67, 0.81]	0.34 [0.18, 0.43]	0.31 [0.00, 0.44]	0.67 [0.48, 0.74]	0.24 [-0.07, 0.36]	0.82 [0.68, 0.97]	0.52 [0.00, 0.75]	0.23 [0.00, 0.40]	0.93 [0.88, 0.96]
lr0.0003_wd0.001_h400_d3	0.78 [0.67, 0.88]	0.40 [0.29, 0.55]	0.21 [0.00, 0.46]	0.57 [0.44, 0.77]	0.10 [-0.08, 0.40]	0.67 [0.21, 0.97]	0.47 [0.00, 0.75]	0.16 [0.00, 0.33]	0.90 [0.86, 0.96]
lr0.0003_wd0.001_h512_d3	0.76 [0.65, 0.83]	0.35 [0.26, 0.43]	0.31 [0.18, 0.50]	0.61 [0.50, 0.72]	0.20 [0.00, 0.43]	0.42 [0.00, 0.93]	0.80 [0.50, 1.00]	0.25 [0.10, 0.50]	0.57 [0.00, 1.00]
lr0.0003_wd0.001_h256_d4	0.76 [0.62, 0.84]	0.44 [0.37, 0.55]	0.27 [0.00, 0.50]	0.61 [0.48, 0.72]	0.19 [-0.07, 0.43]	0.85 [0.66, 0.97]	0.38 [0.00, 0.75]	0.24 [0.00, 0.50]	0.92 [0.88, 0.95]
lr0.0003_wd0.001_h400_d4	0.80 [0.69, 0.88]	0.47 [0.31, 0.64]	0.28 [0.00, 0.40]	0.68 [0.48, 0.79]	0.23 [-0.07, 0.34]	0.72 [0.41, 0.97]	0.63 [0.00, 1.00]	0.19 [0.00, 0.33]	0.95 [0.88, 1.00]
lr0.0003_wd0.001_h512_d4	0.81 [0.73, 0.88]	0.41 [0.24, 0.58]	0.25 [0.00, 0.40]	0.62 [0.48, 0.74]	0.16 [-0.07, 0.33]	0.79 [0.71, 0.97]	0.45 [0.00, 0.75]	0.17 [0.00, 0.29]	0.92 [0.88, 0.95]
lr0.001_wd0.0_h256_d3	0.76 [0.67, 0.80]	0.39 [0.17, 0.62]	0.23 [0.00, 0.40]	0.60 [0.48, 0.69]	0.14 [-0.07, 0.31]	0.76 [0.38, 0.97]	0.43 [0.00, 1.00]	0.17 [0.00, 0.33]	0.93 [0.88, 1.00]
lr0.001_wd0.0_h400_d3	0.79 [0.70, 0.86]	0.52 [0.35, 0.60]	0.25 [0.00, 0.36]	0.63 [0.48, 0.73]	0.18 [-0.07, 0.31]	0.70 [0.28, 0.97]	0.57 [0.00, 1.00]	0.17 [0.00, 0.29]	0.94 [0.88, 1.00]
lr0.001_wd0.0_h512_d3	0.79 [0.70, 0.88]	0.41 [0.19, 0.59]	0.25 [0.00, 0.60]	0.60 [0.48, 0.82]	0.16 [-0.07, 0.55]	0.60 [0.00, 0.97]	0.60 [0.00, 1.00]	0.19 [0.00, 0.50]	0.75 [0.00, 1.00]
lr0.001_wd0.0_h256_d4	0.80 [0.77, 0.82]	0.49 [0.36, 0.71]	0.33 [0.20, 0.44]	0.65 [0.57, 0.74]	0.23 [0.08, 0.36]	0.73 [0.46, 0.90]	0.57 [0.50, 0.67]	0.25 [0.12, 0.40]	0.93 [0.91, 0.96]
lr0.001_wd0.0_h400_d4	0.79 [0.71, 0.85]	0.55 [0.29, 0.74]	0.36 [0.29, 0.50]	0.70 [0.63, 0.79]	0.28 [0.17, 0.44]	0.73 [0.52, 0.83]	0.67 [0.50, 0.75]	0.25 [0.18, 0.38]	0.95 [0.92, 0.96]
lr0.001_wd0.0_h512_d4	0.79 [0.71, 0.84]	0.53 [0.34, 0.71]	0.22 [0.00, 0.38]	0.60 [0.48, 0.72]	0.13 [-0.07, 0.30]	0.69 [0.54, 0.97]	0.52 [0.00, 0.75]	0.14 [0.00, 0.25]	0.92 [0.88, 0.95]
lr0.001_wd0.0001_h256_d3	0.78 [0.71, 0.84]	0.40 [0.34, 0.51]	0.28 [0.00, 0.44]	0.65 [0.45, 0.76]	0.20 [-0.12, 0.39]	0.73 [0.34, 0.90]	0.57 [0.00, 1.00]	0.20 [0.00, 0.33]	0.94 [0.87, 1.00]
lr0.001_wd0.0001_h400_d3	0.80 [0.69, 0.91]	0.52 [0.40, 0.61]	0.24 [0.00, 0.44]	0.60 [0.48, 0.71]	0.16 [-0.07, 0.36]	0.64 [0.17, 0.97]	0.57 [0.00, 1.00]	0.18 [0.00, 0.40]	0.94 [0.88, 1.00]
lr0.001_wd0.0001_h512_d3	0.81 [0.73, 0.84]	0.49 [0.20, 0.73]	0.37 [0.23, 0.57]	0.75 [0.65, 0.84]	0.31 [0.22, 0.43]	0.83 [0.68, 0.96]	0.68 [0.50, 1.00]	0.39 [0.25, 0.67]	0.95 [0.92, 1.00]
lr0.001_wd0.0001_h256_d4	0.82 [0.70, 0.91]	0.43 [0.23, 0.57]	0.34 [0.24, 0.53]	0.70 [0.57, 0.88]	0.28 [0.14, 0.53]	0.57 [0.14, 0.83]	0.83 [0.50, 1.00]	0.23 [0.14, 0.36]	0.97 [0.92, 1.00]
lr0.001_wd0.0001_h400_d4	0.81 [0.80, 0.84]	0.44 [0.29, 0.56]	0.24 [0.18, 0.32]	0.58 [0.50, 0.67]	0.11 [0.00, 0.22]	0.42 [0.00, 0.90]	0.73 [0.25, 1.00]	0.16 [0.10, 0.25]	0.76 [0.00, 1.00]
lr0.001_wd0.0001_h512_d4	0.82 [0.76, 0.86]	0.42 [0.32, 0.56]	0.21 [0.00, 0.38]	0.59 [0.45, 0.78]	0.12 [-0.12, 0.36]	0.51 [0.04, 0.90]	0.67 [0.00, 1.00]	0.12 [0.00, 0.24]	0.96 [0.87, 1.00]
lr0.001_wd0.001_h256_d3	0.79 [0.71, 0.84]	0.47 [0.28, 0.70]	0.35 [0.22, 0.43]	0.68 [0.60, 0.75]	0.26 [0.12, 0.36]	0.75 [0.54, 0.86]	0.62 [0.50, 0.75]	0.25 [0.13, 0.33]	0.94 [0.92, 0.96]
lr0.001_wd0.001_h400_d3	0.78 [0.67, 0.88]	0.50 [0.22, 0.71]	0.32 [0.21, 0.40]	0.68 [0.58, 0.74]	0.24 [0.10, 0.33]	0.65 [0.50, 0.72]	0.72 [0.67, 0.75]	0.21 [0.13, 0.27]	0.95 [0.93, 0.95]
lr0.001_wd0.001_h512_d3	0.81 [0.69, 0.91]	0.47 [0.46, 0.49]	0.37 [0.20, 0.50]	0.70 [0.54, 0.81]	0.30 [0.07, 0.43]	0.78 [0.62, 0.93]	0.62 [0.25, 1.00]	0.28 [0.17, 0.50]	0.95 [0.89, 1.00]
lr0.001_wd0.001_h256_d4	0.78 [0.71, 0.82]	0.47 [0.32, 0.70]	0.25 [0.00, 0.40]	0.61 [0.48, 0.74]	0.15 [-0.07, 0.33]	0.64 [0.00, 0.97]	0.58 [0.00, 1.00]	0.17 [0.00, 0.33]	0.74 [0.00, 0.95]
lr0.001_wd0.001_h400_d4	0.80 [0.71, 0.91]	0.34 [0.16, 0.48]	0.20 [0.00, 0.33]	0.58 [0.45, 0.72]	0.11 [-0.12, 0.30]	0.58 [0.14, 0.90]	0.58 [0.00, 1.00]	0.13 [0.00, 0.20]	0.94 [0.87, 1.00]
lr0.001_wd0.001_h512_d4	0.78 [0.70, 0.84]	0.47 [0.37, 0.70]	0.25 [0.00, 0.40]	0.61 [0.47, 0.73]	0.16 [-0.09, 0.31]	0.81 [0.57, 0.93]	0.42 [0.00, 0.67]	0.20 [0.00, 0.33]	0.92 [0.87, 0.96]

Supplementary Table 21: Grid search results on the classification task on GDHCM retrospective dataset (choosing 40% as the threshold for clinical variables selection). Reported metrics are AUC, AUPRC, F1, Balanced Accuracy, MCC, Specificity, Sensitivity, PPV, and NPV.

Config	AUC	AUPRC	F1	Balanced_Accuracy	MCC	Specificity	Sensitivity	PPV	NPV
lr0.0001_wd0.0_h256_d1	0.72 [0.69, 0.77]	0.47 [0.29, 0.73]	0.31 [0.22, 0.36]	0.60 [0.54, 0.68]	0.16 [0.05, 0.25]	0.54 [0.20, 0.74]	0.65 [0.33, 1.00]	0.21 [0.17, 0.25]	0.91 [0.88, 1.00]
lr0.0001_wd0.0_h400_d1	0.72 [0.61, 0.86]	0.42 [0.23, 0.54]	0.30 [0.00, 0.46]	0.63 [0.47, 0.82]	0.18 [-0.09, 0.44]	0.67 [0.37, 0.95]	0.58 [0.00, 1.00]	0.22 [0.00, 0.40]	0.90 [0.83, 1.00]
lr0.0001_wd0.0_h512_d1	0.72 [0.57, 0.89]	0.56 [0.26, 0.72]	0.30 [0.25, 0.36]	0.56 [0.45, 0.68]	0.11 [-0.10, 0.25]	0.34 [0.05, 0.75]	0.78 [0.50, 1.00]	0.20 [0.14, 0.29]	0.91 [0.75, 1.00]
lr0.0001_wd0.0_h256_d2	0.74 [0.69, 0.86]	0.48 [0.32, 0.66]	0.24 [0.00, 0.40]	0.55 [0.50, 0.76]	0.07 [0.00, 0.36]	0.31 [0.00, 1.00]	0.80 [0.00, 1.00]	0.14 [0.00, 0.25]	0.37 [0.00, 1.00]
lr0.0001_wd0.0_h400_d2	0.74 [0.62, 0.82]	0.44 [0.24, 0.62]	0.32 [0.24, 0.43]	0.57 [0.42, 0.79]	0.10 [-0.17, 0.40]	0.30 [0.00, 0.80]	0.85 [0.50, 1.00]	0.21 [0.14, 0.33]	0.51 [0.00, 1.00]
lr0.0001_wd0.0_h512_d2	0.72 [0.57, 0.81]	0.44 [0.25, 0.61]	0.24 [0.00, 0.44]	0.53 [0.45, 0.68]	0.05 [-0.13, 0.32]	0.55 [0.00, 0.90]	0.52 [0.00, 1.00]	0.19 [0.00, 0.40]	0.67 [0.00, 0.89]
lr0.0001_wd0.0001_h256_d1	0.73 [0.60, 0.86]	0.49 [0.30, 0.64]	0.25 [0.17, 0.32]	0.52 [0.45, 0.57]	0.03 [-0.08, 0.12]	0.35 [0.00, 0.65]	0.68 [0.25, 1.00]	0.16 [0.12, 0.20]	0.69 [0.00, 0.89]
lr0.0001_wd0.0001_h400_d1	0.70 [0.64, 0.82]	0.47 [0.34, 0.65]	0.28 [0.22, 0.30]	0.53 [0.38, 0.60]	-0.00 [-0.47, 0.16]	0.46 [0.00, 0.84]	0.60 [0.33, 0.75]	0.19 [0.13, 0.25]	0.71 [0.00, 0.91]
lr0.0001_wd0.0001_h512_d1	0.70 [0.62, 0.82]	0.45 [0.26, 0.53]	0.33 [0.27, 0.43]	0.61 [0.53, 0.70]	0.17 [0.04, 0.30]	0.67 [0.55, 0.89]	0.55 [0.33, 0.75]	0.25 [0.18, 0.33]	0.89 [0.85, 0.93]
lr0.0001_wd0.0001_h256_d2	0.73 [0.57, 0.91]	0.52 [0.22, 0.73]	0.26 [0.24, 0.29]	0.50 [0.47, 0.53]	0.00 [-0.05, 0.04]	0.17 [0.00, 0.37]	0.83 [0.67, 1.00]	0.16 [0.14, 0.18]	0.51 [0.00, 0.88]
lr0.0001_wd0.0001_h400_d2	0.73 [0.62, 0.86]	0.51 [0.28, 0.73]	0.33 [0.26, 0.40]	0.60 [0.47, 0.70]	0.16 [-0.05, 0.30]	0.43 [0.20, 0.74]	0.77 [0.67, 1.00]	0.21 [0.16, 0.29]	0.91 [0.80, 1.00]
lr0.0001_wd0.0001_h512_d2	0.71 [0.64, 0.86]	0.47 [0.25, 0.56]	0.40 [0.33, 0.50]	0.65 [0.60, 0.70]	0.31 [0.23, 0.40]	0.76 [0.25, 0.95]	0.55 [0.25, 1.00]	0.40 [0.21, 0.50]	0.92 [0.86, 1.00]
lr0.0001_wd0.001_h256_d1	0.71 [0.56, 0.82]	0.48 [0.21, 0.73]	0.23 [0.00, 0.38]	0.56 [0.47, 0.74]	0.07 [-0.09, 0.33]	0.53 [0.37, 0.95]	0.58 [0.00, 1.00]	0.15 [0.00, 0.23]	0.88 [0.82, 1.00]
lr0.0001_wd0.001_h400_d1	0.72 [0.62, 0.82]	0.50 [0.25, 0.65]	0.35 [0.26, 0.50]	0.63 [0.47, 0.84]	0.19 [-0.05, 0.48]	0.41 [0.20, 0.68]	0.85 [0.75, 1.00]	0.22 [0.16, 0.33]	0.92 [0.80, 1.00]
lr0.0001_wd0.001_h512_d1	0.72 [0.64, 0.91]	0.43 [0.31, 0.56]	0.34 [0.25, 0.43]	0.62 [0.53, 0.79]	0.19 [0.06, 0.40]	0.50 [0.05, 0.80]	0.73 [0.50, 1.00]	0.23 [0.15, 0.33]	0.93 [0.87, 1.00]
lr0.0001_wd0.001_h256_d2	0.72 [0.59, 0.82]	0.44 [0.27, 0.58]	0.23 [0.00, 0.35]	0.55 [0.50, 0.71]	0.09 [0.00, 0.30]	0.35 [0.00, 1.00]	0.75 [0.00, 1.00]	0.14 [0.00, 0.21]	0.73 [0.00, 1.00]
lr0.0001_wd0.001_h400_d2	0.71 [0.56, 0.79]	0.47 [0.25, 0.73]	0.25 [0.24, 0.29]	0.48 [0.42, 0.52]	-0.05 [-0.17, 0.03]	0.12 [0.00, 0.37]	0.83 [0.67, 1.00]	0.15 [0.14, 0.17]	0.46 [0.00, 0.88]
lr0.0001_wd0.001_h512_d2	0.73 [0.60, 0.89]	0.53 [0.28, 0.64]	0.29 [0.25, 0.38]	0.55 [0.45, 0.74]	0.06 [-0.10, 0.33]	0.27 [0.00, 0.58]	0.83 [0.67, 1.00]	0.18 [0.15, 0.23]	0.68 [0.00, 1.00]
lr0.0003_wd0.0_h256_d1	0.71 [0.59, 0.79]	0.48 [0.29, 0.73]	0.31 [0.22, 0.44]	0.58 [0.50, 0.68]	0.14 [0.00, 0.32]	0.50 [0.10, 0.85]	0.65 [0.33, 1.00]	0.22 [0.17, 0.40]	0.90 [0.83, 1.00]
lr0.0003_wd0.0_h400_d1	0.69 [0.59, 0.81]	0.48 [0.30, 0.73]	0.37 [0.24, 0.67]	0.58 [0.42, 0.81]	0.15 [-0.17, 0.61]	0.57 [0.10, 0.95]	0.60 [0.33, 0.75]	0.31 [0.14, 0.67]	0.83 [0.67, 0.95]
lr0.0003_wd0.0_h512_d1	0.73 [0.65, 0.84]	0.47 [0.35, 0.56]	0.34 [0.29, 0.44]	0.61 [0.53, 0.68]	0.18 [0.04, 0.32]	0.67 [0.30, 0.85]	0.55 [0.33, 0.75]	0.26 [0.18, 0.40]	0.89 [0.86, 0.93]
lr0.0003_wd0.0_h256_d2	0.73 [0.64, 0.86]	0.47 [0.25, 0.74]	0.37 [0.30, 0.50]	0.64 [0.53, 0.70]	0.26 [0.09, 0.40]	0.51 [0.05, 0.90]	0.77 [0.33, 1.00]	0.29 [0.17, 0.50]	0.96 [0.89, 1.00]
lr0.0003_wd0.0_h400_d2	0.74 [0.69, 0.84]	0.54 [0.27, 0.73]	0.25 [0.00, 0.36]	0.56 [0.47, 0.62]	0.09 [-0.09, 0.20]	0.53 [0.10, 0.95]	0.58 [0.00, 1.00]	0.17 [0.00, 0.29]	0.90 [0.86, 1.00]
lr0.0003_wd0.0_h512_d2	0.74 [0.66, 0.89]	0.53 [0.31, 0.73]	0.33 [0.24, 0.40]	0.59 [0.52, 0.65]	0.18 [0.03, 0.34]	0.53 [0.15, 0.95]	0.65 [0.33, 1.00]	0.27 [0.14, 0.50]	0.91 [0.88, 1.00]
lr0.0003_wd0.0001_h256_d1	0.74 [0.68, 0.84]	0.51 [0.31, 0.62]	0.37 [0.33, 0.40]	0.65 [0.60, 0.70]	0.23 [0.16, 0.30]	0.70 [0.55, 0.89]	0.60 [0.33, 0.75]	0.28 [0.25, 0.33]	0.91 [0.88, 0.93]
lr0.0003_wd0.0001_h400_d1	0.73 [0.60, 0.84]	0.51 [0.31, 0.65]	0.33 [0.29, 0.40]	0.60 [0.53, 0.70]	0.16 [0.04, 0.30]	0.60 [0.30, 0.89]	0.60 [0.33, 0.75]	0.25 [0.18, 0.33]	0.88 [0.86, 0.93]
lr0.0003_wd0.0001_h512_d1	0.70 [0.60, 0.81]	0.44 [0.25, 0.69]	0.32 [0.18, 0.40]	0.61 [0.47, 0.68]	0.19 [-0.04, 0.27]	0.66 [0.37, 0.89]	0.57 [0.25, 1.00]	0.25 [0.14, 0.33]	0.90 [0.82, 1.00]
lr0.0003_wd0.0001_h256_d2	0.71 [0.60, 0.81]	0.50 [0.26, 0.73]	0.34 [0.29, 0.44]	0.62 [0.57, 0.68]	0.19 [0.12, 0.32]	0.63 [0.40, 0.85]	0.60 [0.33, 0.75]	0.26 [0.20, 0.40]	0.90 [0.89, 0.92]
lr0.0003_wd0.0001_h400_d2	0.74 [0.66, 0.86]	0.51 [0.29, 0.65]	0.38 [0.25, 0.44]	0.65 [0.54, 0.70]	0.24 [0.06, 0.32]	0.69 [0.42, 0.89]	0.60 [0.33, 0.75]	0.30 [0.15, 0.40]	0.91 [0.89, 0.93]
lr0.0003_wd0.0001_h512_d2	0.75 [0.66, 0.88]	0.49 [0.27, 0.62]	0.32 [0.25, 0.40]	0.58 [0.45, 0.66]	0.15 [-0.10, 0.26]	0.45 [0.10, 0.89]	0.72 [0.33, 1.00]	0.24 [0.15, 0.33]	0.91 [0.75, 1.00]
lr0.0003_wd0.001_h256_d1	0.74 [0.61, 0.86]	0.50 [0.27, 0.62]	0.43 [0.27, 0.60]	0.69 [0.50, 0.89]	0.30 [0.00, 0.58]	0.64 [0.25, 0.85]	0.73 [0.50, 1.00]	0.32 [0.17, 0.43]	0.92 [0.83, 1.00]
lr0.0003_wd0.001_h400_d1	0.73 [0.65, 0.84]	0.54 [0.29, 0.73]	0.37 [0.25, 0.50]	0.64 [0.55, 0.75]	0.24 [0.10, 0.42]	0.78 [0.45, 0.89]	0.50 [0.25, 0.75]	0.32 [0.21, 0.40]	0.90 [0.85, 0.94]
lr0.0003_wd0.001_h512_d1	0.73 [0.66, 0.84]	0.50 [0.29, 0.73]	0.36 [0.32, 0.40]	0.63 [0.57, 0.70]	0.22 [0.12, 0.30]	0.62 [0.30, 0.89]	0.65 [0.33, 1.00]	0.27 [0.20, 0.33]	0.92 [0.88, 1.00]
lr0.0003_wd0.001_h256_d2	0.75 [0.65, 0.88]	0.54 [0.28, 0.74]	0.27 [0.20, 0.35]	0.55 [0.50, 0.62]	0.08 [0.00, 0.19]	0.39 [0.00, 0.75]	0.70 [0.25, 1.00]	0.17 [0.14, 0.23]	0.72 [0.00, 1.00]
lr0.0003_wd0.001_h400_d2	0.73 [0.64, 0.84]	0.50 [0.25, 0.64]	0.35 [0.20, 0.50]	0.61 [0.50, 0.70]	0.21 [0.00, 0.40]	0.77 [0.47, 0.90]	0.45 [0.25, 0.67]	0.31 [0.17, 0.50]	0.88 [0.83, 0.90]
lr0.0003_wd0.001_h512_d2	0.79 [0.71, 0.88]	0.49 [0.29, 0.65]	0.34 [0.26, 0.50]	0.59 [0.53, 0.75]	0.19 [0.09, 0.42]	0.25 [0.05, 0.84]	0.93 [0.67, 1.00]	0.22 [0.15, 0.40]	0.99 [0.94, 1.00]
lr0.001_wd0.0_h256_d1	0.78 [0.72, 0.86]	0.56 [0.34, 0.75]	0.39 [0.29, 0.47]	0.68 [0.60, 0.78]	0.27 [0.13, 0.41]	0.59 [0.50, 0.70]	0.77 [0.67, 1.00]	0.26 [0.18, 0.33]	0.94 [0.91, 1.00]
lr0.001_wd0.0_h400_d1	0.79 [0.70, 0.93]	0.54 [0.42, 0.65]	0.40 [0.30, 0.57]	0.65 [0.55, 0.78]	0.27 [0.08, 0.50]	0.62 [0.26, 0.90]	0.68 [0.50, 1.00]	0.32 [0.18, 0.50]	0.92 [0.88, 1.00]
lr0.001_wd0.0_h512_d1	0.74 [0.61, 0.89]	0.47 [0.25, 0.60]	0.42 [0.29, 0.67]	0.66 [0.55, 0.81]	0.31 [0.08, 0.61]	0.54 [0.21, 0.95]	0.78 [0.50, 1.00]	0.35 [0.17, 0.67]	0.94 [0.88, 1.00]
lr0.001_wd0.0_h256_d2	0.72 [0.68, 0.86]	0.51 [0.31, 0.61]	0.36 [0.31, 0.40]	0.63 [0.57, 0.70]	0.21 [0.12, 0.30]	0.65 [0.40, 0.80]	0.62 [0.50, 0.75]	0.26 [0.20, 0.33]	0.90 [0.88, 0.93]
lr0.001_wd0.0_h400_d2	0.78 [0.66, 0.91]	0.55 [0.26, 0.75]	0.40 [0.29, 0.55]	0.69 [0.53, 0.87]	0.27 [0.04, 0.53]	0.54 [0.30, 0.74]	0.83 [0.67, 1.00]	0.26 [0.18, 0.38]	0.94 [0.86, 1.00]
lr0.001_wd0.0_h512_d2	0.75 [0.68, 0.89]	0.53 [0.29, 0.72]	0.50 [0.33, 0.80]	0.71 [0.61, 0.83]	0.41 [0.23, 0.80]	0.83 [0.65, 1.00]	0.60 [0.33, 0.75]	0.48 [0.30, 1.00]	0.92 [0.89, 0.95]
lr0.001_wd0.0001_h256_d1	0.74 [0.65, 0.88]	0.44 [0.28, 0.57]	0.35 [0.24, 0.57]	0.61 [0.50, 0.78]	0.19 [0.00, 0.50]	0.65 [0.37, 0.90]	0.57 [0.25, 0.75]	0.28 [0.14, 0.50]	0.89 [0.83, 0.94]
lr0.001_wd0.0001_h400_d1	0.75 [0.65, 0.88]	0.51 [0.33, 0.76]	0.38 [0.32, 0.50]	0.67 [0.60, 0.84]	0.26 [0.15, 0.48]	0.59 [0.32, 0.75]	0.75 [0.50, 1.00]	0.26 [0.19, 0.33]	0.93 [0.88, 1.00]
lr0.001_wd0.0001_h512_d1	0.77 [0.62, 0.88]	0.49 [0.24, 0.77]	0.35 [0.31, 0.46]	0.64 [0.57, 0.72]	0.22 [0.12, 0.35]	0.61 [0.32, 0.89]	0.67 [0.33, 1.00]	0.26 [0.19, 0.33]	0.92 [0.87, 1.00]
lr0.001_wd0.0001_h256_d2	0.79 [0.62, 0.91]	0.52 [0.24, 0.79]	0.47 [0.31, 0.57]	0.72 [0.57, 0.78]	0.35 [0.12, 0.50]	0.77 [0.65, 0.89]	0.67 [0.50, 0.75]	0.36 [0.22, 0.50]	0.92 [0.87, 0.94]
lr0.001_wd0.0001_h400_d2	0.75 [0.62, 0.91]	0.57 [0.33, 0.70]	0.45 [0.33, 0.57]	0.69 [0.62, 0.75]	0.40 [0.27, 0.51]	0.80 [0.37, 1.00]	0.58 [0.25, 1.00]	0.53 [0.20, 1.00]	0.92 [0.87, 1.00]
lr0.001_wd0.0001_h512_d2	0.73 [0.61, 0.81]	0.50 [0.32, 0.73]	0.40 [0.31, 0.67]	0.64 [0.55, 0.81]	0.27 [0.12, 0.61]	0.63 [0.10, 0.95]	0.65 [0.33, 1.00]	0.34 [0.18, 0.67]	0.92 [0.89, 1.00]
lr0.001_wd0.001_h256_d1	0.74 [0.61, 0.86]	0.49 [0.26, 0.63]	0.33 [0.15, 0.46]	0.62 [0.42, 0.79]	0.18 [-0.12, 0.40]	0.61 [0.47, 0.70]	0.63 [0.25, 1.00]	0.23 [0.11, 0.33]	0.90 [0.80, 1.00]
lr0.001_wd0.001_h400_d1	0.74 [0.62, 0.86]	0.48 [0.29, 0.76]	0.32 [0.20, 0.40]	0.61 [0.50, 0.68]	0.18 [0.00, 0.26]	0.67 [0.55, 0.89]	0.55 [0.25, 0.75]	0.24 [0.17, 0.33]	0.90 [0.83, 0.92]
lr0.001_wd0.001_h512_d1	0.72 [0.64, 0.84]	0.48 [0.36, 0.62]	0.34 [0.25, 0.50]	0.62 [0.55, 0.75]	0.19 [0.08, 0.42]	0.67 [0.50, 0.85]	0.57 [0.25, 0.75]	0.26 [0.20, 0.40]	0.89 [0.85, 0.94]
lr0.001_wd0.001_h256_d2	0.73 [0.65, 0.84]	0.47 [0.31, 0.62]	0.36 [0.25, 0.50]	0.61 [0.53, 0.70]	0.24 [0.09, 0.40]	0.65 [0.05, 0.95]	0.57 [0.25, 1.00]	0.37 [0.14, 0.50]	0.91 [0.86, 1.00]
lr0.001_wd0.001_h400_d2	0.77 [0.65, 0.93]	0.60 [0.26, 0.73]	0.38 [0.20, 0.57]	0.62 [0.50, 0.78]	0.22 [0.00, 0.50]	0.63 [0.25, 0.89]	0.62 [0.25, 0.75]	0.30 [0.17, 0.50]	0.89 [0.83, 0.94]
lr0.001_wd0.001_h512_d2	0.79 [0.69, 0.88]	0.59 [0.37, 0.73]	0.35 [0.27, 0.43]	0.61 [0.55, 0.70]	0.25 [0.10, 0.47]	0.62 [0.10, 1.00]	0.60 [0.25, 1.00]	0.40 [0.17, 1.00]	0.92 [0.87, 1.00]

Supplementary Table 22: Grid search results on the classification task on GDHCM prospective dataset (choosing 40% as the threshold for clinical variables selection). Reported metrics are AUC, AUPRC, F1, Balanced Accuracy, MCC, Specificity, Sensitivity, PPV, and NPV.

Config	AUC	AUPRC	F1	Balanced_Accuracy	MCC	Specificity	Sensitivity	PPV	NPV
lr1e-05_wd0.005_h1024_d3	0.70 [0.61, 0.84]	0.46 [0.30, 0.59]	0.33 [0.22, 0.55]	0.59 [0.49, 0.87]	0.13 [-0.01, 0.53]	0.29 [0.00, 0.74]	0.88 [0.67, 1.00]	0.21 [0.13, 0.38]	0.55 [0.00, 1.00]
lr1e-05_wd0.005_h2048_d3	0.71 [0.56, 0.89]	0.51 [0.21, 0.68]	0.25 [0.00, 0.38]	0.57 [0.43, 0.74]	0.09 [-0.17, 0.33]	0.57 [0.25, 0.85]	0.57 [0.00, 1.00]	0.17 [0.00, 0.25]	0.89 [0.81, 1.00]
lr1e-05_wd0.005_h1024_d4	0.77 [0.70, 0.84]	0.56 [0.42, 0.72]	0.33 [0.24, 0.57]	0.56 [0.50, 0.78]	0.10 [0.00, 0.50]	0.18 [0.00, 0.89]	0.93 [0.67, 1.00]	0.23 [0.14, 0.50]	0.19 [0.00, 0.94]
lr1e-05_wd0.005_h2048_d4	0.73 [0.65, 0.84]	0.44 [0.27, 0.60]	0.32 [0.24, 0.44]	0.56 [0.50, 0.68]	0.11 [0.00, 0.32]	0.35 [0.00, 0.89]	0.77 [0.33, 1.00]	0.24 [0.14, 0.40]	0.36 [0.00, 0.89]
lr1e-05_wd0.01_h1024_d3	0.71 [0.64, 0.81]	0.46 [0.28, 0.65]	0.17 [0.00, 0.29]	0.52 [0.50, 0.61]	0.04 [0.00, 0.19]	0.44 [0.00, 1.00]	0.60 [0.00, 1.00]	0.10 [0.00, 0.17]	0.54 [0.00, 1.00]
lr1e-05_wd0.01_h2048_d3	0.71 [0.64, 0.84]	0.45 [0.26, 0.57]	0.32 [0.29, 0.40]	0.59 [0.50, 0.71]	0.13 [0.00, 0.30]	0.29 [0.00, 0.74]	0.88 [0.67, 1.00]	0.20 [0.17, 0.29]	0.56 [0.00, 1.00]
lr1e-05_wd0.01_h1024_d4	0.71 [0.55, 0.89]	0.43 [0.21, 0.64]	0.21 [0.00, 0.29]	0.50 [0.50, 0.50]	0.00 [0.00, 0.00]	0.20 [0.00, 1.00]	0.80 [0.00, 1.00]	0.12 [0.00, 0.17]	0.17 [0.00, 0.83]
lr1e-05_wd0.01_h2048_d4	0.75 [0.66, 0.88]	0.45 [0.32, 0.59]	0.30 [0.24, 0.40]	0.53 [0.50, 0.64]	0.07 [0.00, 0.34]	0.19 [0.00, 0.95]	0.87 [0.33, 1.00]	0.23 [0.14, 0.50]	0.18 [0.00, 0.90]
lr3e-05_wd0.005_h1024_d3	0.72 [0.64, 0.86]	0.47 [0.24, 0.56]	0.26 [0.00, 0.36]	0.58 [0.45, 0.68]	0.11 [-0.13, 0.25]	0.63 [0.00, 0.90]	0.52 [0.00, 1.00]	0.20 [0.00, 0.33]	0.71 [0.00, 0.93]
lr3e-05_wd0.005_h2048_d3	0.74 [0.64, 0.91]	0.52 [0.24, 0.73]	0.29 [0.00, 0.50]	0.54 [0.35, 0.67]	0.14 [-0.26, 0.55]	0.74 [0.15, 1.00]	0.33 [0.00, 0.75]	0.40 [0.00, 1.00]	0.84 [0.75, 0.90]
lr3e-05_wd0.005_h1024_d4	0.72 [0.61, 0.86]	0.44 [0.25, 0.61]	0.34 [0.24, 0.50]	0.57 [0.50, 0.70]	0.17 [0.00, 0.40]	0.38 [0.00, 0.95]	0.77 [0.33, 1.00]	0.30 [0.14, 0.50]	0.56 [0.00, 1.00]
lr3e-05_wd0.005_h2048_d4	0.74 [0.68, 0.91]	0.51 [0.26, 0.61]	0.33 [0.25, 0.40]	0.57 [0.45, 0.64]	0.17 [-0.10, 0.34]	0.60 [0.00, 0.95]	0.53 [0.25, 1.00]	0.36 [0.15, 0.50]	0.68 [0.00, 0.90]
lr3e-05_wd0.01_h1024_d3	0.72 [0.60, 0.84]	0.51 [0.27, 0.73]	0.32 [0.24, 0.40]	0.56 [0.43, 0.65]	0.10 [-0.17, 0.34]	0.46 [0.00, 0.95]	0.65 [0.33, 1.00]	0.26 [0.14, 0.50]	0.67 [0.00, 0.90]
lr3e-05_wd0.01_h2048_d3	0.74 [0.58, 0.88]	0.42 [0.22, 0.53]	0.29 [0.24, 0.32]	0.54 [0.50, 0.59]	0.07 [0.00, 0.16]	0.32 [0.00, 0.84]	0.77 [0.33, 1.00]	0.19 [0.14, 0.25]	0.53 [0.00, 0.89]
lr3e-05_wd0.01_h1024_d4	0.72 [0.64, 0.86]	0.49 [0.30, 0.61]	0.30 [0.23, 0.40]	0.54 [0.40, 0.65]	0.05 [-0.27, 0.34]	0.43 [0.05, 0.95]	0.65 [0.33, 0.75]	0.24 [0.14, 0.50]	0.79 [0.50, 0.92]
lr3e-05_wd0.01_h2048_d4	0.73 [0.61, 0.88]	0.56 [0.42, 0.73]	0.41 [0.29, 0.57]	0.64 [0.50, 0.78]	0.28 [0.00, 0.50]	0.73 [0.00, 0.95]	0.55 [0.25, 1.00]	0.40 [0.17, 0.50]	0.72 [0.00, 0.94]
lr1e-05_wd0.0_h256_d1	0.77 [0.60, 0.95]	0.57 [0.35, 0.81]	0.29 [0.24, 0.36]	0.56 [0.50, 0.62]	0.11 [0.00, 0.20]	0.26 [0.00, 0.75]	0.85 [0.50, 1.00]	0.19 [0.14, 0.29]	0.75 [0.00, 1.00]
lr1e-05_wd0.0_h400_d1	0.69 [0.57, 0.79]	0.43 [0.23, 0.65]	0.29 [0.25, 0.32]	0.55 [0.45, 0.66]	0.09 [-0.10, 0.24]	0.21 [0.05, 0.32]	0.90 [0.75, 1.00]	0.17 [0.15, 0.19]	0.92 [0.75, 1.00]
lr1e-05_wd0.0_h512_d1	0.66 [0.56, 0.79]	0.44 [0.22, 0.61]	0.25 [0.00, 0.43]	0.57 [0.45, 0.79]	0.09 [-0.13, 0.40]	0.50 [0.10, 0.90]	0.63 [0.00, 1.00]	0.16 [0.00, 0.27]	0.91 [0.82, 1.00]
lr1e-05_wd0.0_h256_d2	0.77 [0.57, 0.88]	0.55 [0.24, 0.77]	0.30 [0.26, 0.33]	0.57 [0.47, 0.68]	0.12 [-0.05, 0.27]	0.20 [0.00, 0.37]	0.95 [0.75, 1.00]	0.18 [0.16, 0.20]	0.76 [0.00, 1.00]
lr1e-05_wd0.0_h400_d2	0.71 [0.61, 0.88]	0.51 [0.23, 0.63]	0.27 [0.24, 0.29]	0.52 [0.50, 0.58]	0.04 [0.00, 0.16]	0.16 [0.00, 0.37]	0.88 [0.67, 1.00]	0.16 [0.14, 0.17]	0.54 [0.00, 1.00]
lr1e-05_wd0.0_h512_d2	0.79 [0.66, 0.91]	0.46 [0.38, 0.55]	0.23 [0.00, 0.33]	0.53 [0.50, 0.61]	0.08 [0.00, 0.23]	0.40 [0.00, 1.00]	0.67 [0.00, 1.00]	0.16 [0.00, 0.33]	0.75 [0.00, 1.00]
lr1e-05_wd0.0001_h256_d1	0.70 [0.56, 0.88]	0.37 [0.21, 0.64]	0.29 [0.17, 0.50]	0.54 [0.46, 0.70]	0.09 [-0.06, 0.40]	0.36 [0.05, 0.90]	0.72 [0.33, 1.00]	0.22 [0.11, 0.50]	0.91 [0.80, 1.00]
lr1e-05_wd0.0001_h400_d1	0.71 [0.59, 0.88]	0.43 [0.23, 0.53]	0.16 [0.00, 0.27]	0.49 [0.38, 0.55]	-0.00 [-0.23, 0.13]	0.43 [0.05, 1.00]	0.55 [0.00, 1.00]	0.09 [0.00, 0.17]	0.89 [0.79, 1.00]
lr1e-05_wd0.0001_h512_d1	0.74 [0.64, 0.91]	0.42 [0.25, 0.56]	0.23 [0.00, 0.30]	0.53 [0.45, 0.58]	0.05 [-0.13, 0.16]	0.25 [0.00, 0.90]	0.80 [0.00, 1.00]	0.13 [0.00, 0.17]	0.76 [0.00, 1.00]
lr1e-05_wd0.0001_h256_d2	0.72 [0.60, 0.86]	0.44 [0.39, 0.55]	0.29 [0.24, 0.32]	0.55 [0.50, 0.63]	0.11 [0.00, 0.22]	0.10 [0.00, 0.26]	1.00 [1.00, 1.00]	0.17 [0.14, 0.19]	0.80 [0.00, 1.00]
lr1e-05_wd0.0001_h400_d2	0.80 [0.71, 0.93]	0.55 [0.35, 0.64]	0.21 [0.00, 0.29]	0.50 [0.50, 0.50]	0.00 [0.00, 0.00]	0.20 [0.00, 1.00]	0.80 [0.00, 1.00]	0.12 [0.00, 0.17]	0.17 [0.00, 0.83]
lr1e-05_wd0.0001_h512_d2	0.71 [0.57, 0.88]	0.42 [0.22, 0.61]	0.30 [0.25, 0.43]	0.58 [0.50, 0.79]	0.12 [0.00, 0.40]	0.43 [0.00, 0.85]	0.73 [0.25, 1.00]	0.20 [0.15, 0.27]	0.72 [0.00, 1.00]
lr1e-05_wd0.001_h256_d1	0.71 [0.61, 0.88]	0.42 [0.25, 0.61]	0.29 [0.21, 0.33]	0.55 [0.45, 0.68]	0.09 [-0.10, 0.27]	0.28 [0.15, 0.40]	0.83 [0.67, 1.00]	0.17 [0.12, 0.20]	0.89 [0.75, 1.00]
lr1e-05_wd0.001_h400_d1	0.72 [0.56, 0.88]	0.46 [0.28, 0.67]	0.27 [0.00, 0.42]	0.61 [0.50, 0.72]	0.18 [0.00, 0.35]	0.55 [0.05, 1.00]	0.67 [0.00, 1.00]	0.18 [0.00, 0.27]	0.94 [0.83, 1.00]
lr1e-05_wd0.001_h512_d1	0.72 [0.57, 0.86]	0.46 [0.26, 0.64]	0.31 [0.25, 0.35]	0.61 [0.54, 0.71]	0.20 [0.06, 0.30]	0.64 [0.42, 0.95]	0.58 [0.25, 1.00]	0.29 [0.15, 0.50]	0.90 [0.86, 1.00]
lr1e-05_wd0.001_h256_d2	0.73 [0.60, 0.88]	0.42 [0.23, 0.61]	0.28 [0.22, 0.35]	0.56 [0.52, 0.62]	0.12 [0.03, 0.19]	0.40 [0.10, 0.80]	0.73 [0.25, 1.00]	0.18 [0.14, 0.23]	0.93 [0.84, 1.00]
lr1e-05_wd0.001_h400_d2	0.78 [0.64, 0.89]	0.49 [0.31, 0.63]	0.33 [0.25, 0.46]	0.63 [0.55, 0.82]	0.21 [0.10, 0.44]	0.51 [0.10, 0.85]	0.75 [0.25, 1.00]	0.23 [0.18, 0.30]	0.94 [0.85, 1.00]
lr1e-05_wd0.001_h512_d2	0.75 [0.68, 0.86]	0.45 [0.32, 0.54]	0.27 [0.24, 0.31]	0.53 [0.50, 0.57]	0.05 [0.00, 0.12]	0.20 [0.00, 0.65]	0.85 [0.50, 1.00]	0.17 [0.14, 0.22]	0.54 [0.00, 1.00]
lr3e-05_wd0.0_h256_d1	0.74 [0.66, 0.84]	0.49 [0.33, 0.65]	0.33 [0.29, 0.40]	0.61 [0.53, 0.76]	0.18 [0.04, 0.36]	0.38 [0.05, 0.75]	0.85 [0.50, 1.00]	0.21 [0.17, 0.29]	0.95 [0.86, 1.00]
lr3e-05_wd0.0_h400_d1	0.75 [0.61, 0.88]	0.51 [0.24, 0.62]	0.31 [0.20, 0.50]	0.56 [0.50, 0.67]	0.17 [0.00, 0.55]	0.45 [0.05, 1.00]	0.67 [0.25, 1.00]	0.33 [0.17, 1.00]	0.91 [0.83, 1.00]
lr3e-05_wd0.0_h512_d1	0.73 [0.62, 0.84]	0.43 [0.32, 0.57]	0.25 [0.00, 0.36]	0.58 [0.50, 0.63]	0.14 [0.00, 0.22]	0.59 [0.10, 1.00]	0.57 [0.00, 1.00]	0.18 [0.00, 0.29]	0.92 [0.83, 1.00]
lr3e-05_wd0.0_h256_d2	0.70 [0.61, 0.88]	0.47 [0.23, 0.59]	0.30 [0.26, 0.33]	0.58 [0.50, 0.68]	0.13 [0.00, 0.27]	0.30 [0.00, 0.65]	0.85 [0.50, 1.00]	0.19 [0.15, 0.22]	0.75 [0.00, 1.00]
lr3e-05_wd0.0_h400_d2	0.71 [0.62, 0.89]	0.45 [0.25, 0.68]	0.27 [0.24, 0.30]	0.52 [0.50, 0.55]	0.02 [0.00, 0.08]	0.18 [0.00, 0.55]	0.85 [0.50, 1.00]	0.16 [0.14, 0.19]	0.34 [0.00, 0.88]
lr3e-05_wd0.0_h512_d2	0.68 [0.56, 0.82]	0.44 [0.21, 0.67]	0.31 [0.24, 0.36]	0.58 [0.50, 0.65]	0.13 [0.00, 0.26]	0.33 [0.00, 0.75]	0.83 [0.50, 1.00]	0.20 [0.14, 0.29]	0.56 [0.00, 1.00]
lr3e-05_wd0.0001_h256_d1	0.71 [0.65, 0.81]	0.50 [0.33, 0.58]	0.30 [0.26, 0.35]	0.58 [0.47, 0.71]	0.14 [-0.05, 0.30]	0.48 [0.20, 0.95]	0.68 [0.25, 1.00]	0.25 [0.16, 0.50]	0.89 [0.80, 1.00]
lr3e-05_wd0.0001_h400_d1	0.73 [0.62, 0.82]	0.57 [0.44, 0.72]	0.28 [0.27, 0.30]	0.55 [0.50, 0.61]	0.07 [0.00, 0.19]	0.21 [0.00, 0.47]	0.88 [0.67, 1.00]	0.17 [0.17, 0.19]	0.56 [0.00, 1.00]
lr3e-05_wd0.0001_h512_d1	0.68 [0.60, 0.77]	0.47 [0.36, 0.54]	0.26 [0.20, 0.32]	0.54 [0.51, 0.57]	0.06 [0.01, 0.12]	0.53 [0.40, 0.68]	0.55 [0.33, 0.75]	0.17 [0.14, 0.20]	0.87 [0.85, 0.90]
lr3e-05_wd0.0001_h256_d2	0.75 [0.69, 0.84]	0.42 [0.34, 0.58]	0.17 [0.00, 0.33]	0.53 [0.47, 0.60]	0.04 [-0.09, 0.15]	0.50 [0.00, 1.00]	0.55 [0.00, 1.00]	0.10 [0.00, 0.21]	0.71 [0.00, 1.00]
lr3e-05_wd0.0001_h400_d2	0.71 [0.60, 0.86]	0.42 [0.29, 0.59]	0.33 [0.25, 0.44]	0.60 [0.53, 0.68]	0.18 [0.09, 0.32]	0.34 [0.05, 0.85]	0.85 [0.50, 1.00]	0.22 [0.14, 0.40]	0.96 [0.89, 1.00]
lr3e-05_wd0.0001_h512_d2	0.79 [0.64, 0.93]	0.52 [0.35, 0.72]	0.34 [0.25, 0.55]	0.59 [0.50, 0.87]	0.15 [0.00, 0.53]	0.24 [0.00, 0.74]	0.95 [0.75, 1.00]	0.21 [0.14, 0.38]	0.58 [0.00, 1.00]
lr3e-05_wd0.001_h256_d1	0.72 [0.56, 0.88]	0.46 [0.22, 0.56]	0.21 [0.00, 0.33]	0.54 [0.50, 0.65]	0.06 [0.00, 0.21]	0.54 [0.05, 1.00]	0.53 [0.00, 1.00]	0.14 [0.00, 0.22]	0.88 [0.83, 1.00]
lr3e-05_wd0.001_h400_d1	0.71 [0.61, 0.79]	0.49 [0.33, 0.64]	0.34 [0.18, 0.50]	0.62 [0.47, 0.79]	0.21 [-0.04, 0.42]	0.66 [0.25, 0.95]	0.58 [0.25, 1.00]	0.30 [0.14, 0.50]	0.89 [0.82, 1.00]
lr3e-05_wd0.001_h512_d1	0.73 [0.59, 0.93]	0.52 [0.23, 0.68]	0.27 [0.25, 0.31]	0.52 [0.50, 0.55]	0.06 [0.00, 0.13]	0.21 [0.00, 0.50]	0.83 [0.50, 1.00]	0.16 [0.14, 0.18]	0.74 [0.00, 1.00]
lr3e-05_wd0.001_h256_d2	0.75 [0.65, 0.88]	0.54 [0.48, 0.61]	0.28 [0.24, 0.36]	0.52 [0.45, 0.62]	0.03 [-0.10, 0.20]	0.26 [0.00, 0.75]	0.78 [0.50, 1.00]	0.18 [0.14, 0.29]	0.50 [0.00, 0.89]
lr3e-05_wd0.001_h400_d2	0.72 [0.57, 0.88]	0.52 [0.28, 0.80]	0.30 [0.25, 0.35]	0.56 [0.50, 0.71]	0.10 [0.00, 0.30]	0.19 [0.00, 0.42]	0.93 [0.67, 1.00]	0.18 [0.15, 0.21]	0.58 [0.00, 1.00]
lr3e-05_wd0.001_h512_d2	0.78 [0.71, 0.88]	0.44 [0.30, 0.57]	0.21 [0.00, 0.31]	0.52 [0.45, 0.58]	0.04 [-0.10, 0.16]	0.28 [0.00, 1.00]	0.75 [0.00, 1.00]	0.13 [0.00, 0.18]	0.72 [0.00, 1.00]
lr0.0001_wd0.005_h256_d1	0.72 [0.57, 0.89]	0.49 [0.29, 0.73]	0.28 [0.18, 0.35]	0.56 [0.47, 0.62]	0.10 [-0.04, 0.19]	0.49 [0.05, 0.70]	0.63 [0.25, 1.00]	0.19 [0.14, 0.25]	0.90 [0.82, 1.00]
lr0.0001_wd0.005_h400_d1	0.72 [0.61, 0.86]	0.41 [0.23, 0.58]	0.29 [0.00, 0.50]	0.57 [0.35, 0.75]	0.11 [-0.26, 0.40]	0.57 [0.00, 0.75]	0.58 [0.00, 1.00]	0.20 [0.00, 0.38]	0.70 [0.00, 0.94]
lr0.0001_wd0.005_h512_d1	0.73 [0.60, 0.82]	0.55 [0.40, 0.73]	0.29 [0.15, 0.40]	0.56 [0.42, 0.68]	0.10 [-0.12, 0.26]	0.51 [0.00, 0.84]	0.62 [0.25, 1.00]	0.20 [0.11, 0.27]	0.70 [0.00, 0.92]
lr0.0001_wd0.005_h256_d2	0.72 [0.56, 0.82]	0.43 [0.21, 0.69]	0.29 [0.24, 0.35]	0.54 [0.50, 0.62]	0.06 [0.00, 0.19]	0.12 [0.00, 0.50]	0.95 [0.75, 1.00]	0.17 [0.14, 0.23]	0.38 [0.00, 1.00]
lr0.0001_wd0.005_h400_d2	0.73 [0.62, 0.82]	0.43 [0.24, 0.55]	0.26 [0.24, 0.29]	0.48 [0.42, 0.50]	-0.03 [-0.17, 0.00]	0.02 [0.00, 0.10]	0.95 [0.75, 1.00]	0.15 [0.14, 0.17]	0.13 [0.00, 0.67]
lr0.0001_wd0.005_h512_d2	0.72 [0.60, 0.88]	0.50 [0.27, 0.73]	0.31 [0.29, 0.35]	0.59 [0.50, 0.71]	0.14 [0.00, 0.30]	0.34 [0.00, 0.65]	0.83 [0.50, 1.00]	0.19 [0.17, 0.22]	0.76 [0.00, 1.00]
lr0.0001_wd0.01_h256_d1	0.70 [0.60, 0.86]	0.50 [0.35, 0.66]	0.31 [0.18, 0.50]	0.61 [0.47, 0.75]	0.16 [-0.04, 0.40]	0.58 [0.42, 0.75]	0.63 [0.25, 1.00]	0.22 [0.14, 0.38]	0.90 [0.82, 1.00]
lr0.0001_wd0.01_h400_d1	0.71 [0.60, 0.84]	0.39 [0.25, 0.51]	0.29 [0.25, 0.35]	0.57 [0.47, 0.68]	0.11 [-0.05, 0.27]	0.33 [0.05, 0.55]	0.80 [0.50, 1.00]	0.18 [0.14, 0.23]	0.91 [0.80, 1.00]

Supplementary Table 23: Grid search results on the classification task on GDHCM prospective dataset (choosing 40% as the threshold for clinical variables selection). Reported metrics are AUC, AUPRC, F1, Balanced Accuracy, MCC, Specificity, Sensitivity, PPV, and NPV.

Config	AUC	AUPRC	F1	Balanced_Accuracy	MCC	Specificity	Sensitivity	PPV	NPV
lr0.0001_wd0.01_h512_d1	0.74 [0.66, 0.91]	0.54 [0.26, 0.70]	0.34 [0.24, 0.46]	0.62 [0.52, 0.72]	0.19 [0.03, 0.35]	0.51 [0.21, 0.70]	0.73 [0.50, 1.00]	0.23 [0.14, 0.33]	0.92 [0.88, 1.00]
lr0.0001_wd0.01_h256_d2	0.74 [0.66, 0.86]	0.45 [0.31, 0.61]	0.29 [0.24, 0.33]	0.56 [0.47, 0.63]	0.10 [-0.05, 0.22]	0.26 [0.00, 0.65]	0.85 [0.50, 1.00]	0.18 [0.14, 0.22]	0.73 [0.00, 1.00]
lr0.0001_wd0.01_h400_d2	0.75 [0.66, 0.89]	0.56 [0.28, 0.68]	0.22 [0.00, 0.31]	0.52 [0.50, 0.62]	0.03 [0.00, 0.17]	0.32 [0.00, 1.00]	0.73 [0.00, 1.00]	0.13 [0.00, 0.20]	0.35 [0.00, 0.92]
lr0.0001_wd0.01_h512_d2	0.74 [0.61, 0.91]	0.50 [0.29, 0.73]	0.34 [0.25, 0.50]	0.61 [0.50, 0.70]	0.19 [0.00, 0.40]	0.54 [0.00, 0.90]	0.68 [0.25, 1.00]	0.26 [0.17, 0.50]	0.73 [0.00, 1.00]
lr0.0003_wd0.005_h256_d1	0.72 [0.64, 0.81]	0.53 [0.39, 0.73]	0.39 [0.25, 0.67]	0.64 [0.55, 0.81]	0.27 [0.10, 0.61]	0.73 [0.15, 0.95]	0.55 [0.25, 1.00]	0.35 [0.19, 0.67]	0.92 [0.85, 1.00]
lr0.0003_wd0.005_h400_d1	0.72 [0.60, 0.86]	0.48 [0.27, 0.76]	0.29 [0.25, 0.33]	0.56 [0.45, 0.63]	0.10 [-0.10, 0.22]	0.29 [0.15, 0.58]	0.83 [0.67, 1.00]	0.18 [0.15, 0.20]	0.90 [0.75, 1.00]
lr0.0003_wd0.005_h512_d1	0.73 [0.59, 0.82]	0.51 [0.37, 0.73]	0.37 [0.24, 0.46]	0.67 [0.52, 0.82]	0.25 [0.03, 0.44]	0.65 [0.37, 0.85]	0.68 [0.25, 1.00]	0.27 [0.14, 0.33]	0.92 [0.85, 1.00]
lr0.0003_wd0.005_h256_d2	0.73 [0.62, 0.89]	0.52 [0.34, 0.72]	0.32 [0.26, 0.40]	0.58 [0.47, 0.70]	0.13 [-0.05, 0.30]	0.56 [0.20, 0.89]	0.60 [0.33, 0.75]	0.24 [0.16, 0.33]	0.86 [0.80, 0.93]
lr0.0003_wd0.005_h400_d2	0.74 [0.64, 0.88]	0.53 [0.29, 0.73]	0.33 [0.22, 0.46]	0.63 [0.53, 0.82]	0.19 [0.04, 0.44]	0.63 [0.55, 0.80]	0.63 [0.25, 1.00]	0.23 [0.18, 0.30]	0.91 [0.84, 1.00]
lr0.0003_wd0.005_h512_d2	0.74 [0.62, 0.89]	0.54 [0.28, 0.73]	0.43 [0.27, 0.67]	0.65 [0.55, 0.78]	0.30 [0.10, 0.67]	0.57 [0.10, 1.00]	0.72 [0.50, 1.00]	0.41 [0.17, 1.00]	0.93 [0.89, 1.00]
lr0.0003_wd0.01_h256_d1	0.75 [0.68, 0.82]	0.57 [0.43, 0.67]	0.40 [0.29, 0.50]	0.67 [0.60, 0.75]	0.28 [0.13, 0.42]	0.62 [0.20, 0.84]	0.72 [0.50, 1.00]	0.30 [0.18, 0.40]	0.94 [0.89, 1.00]
lr0.0003_wd0.01_h400_d1	0.75 [0.65, 0.88]	0.54 [0.30, 0.67]	0.37 [0.27, 0.50]	0.65 [0.57, 0.75]	0.23 [0.10, 0.40]	0.64 [0.47, 0.89]	0.65 [0.33, 0.75]	0.28 [0.17, 0.38]	0.91 [0.89, 0.94]
lr0.0003_wd0.01_h512_d1	0.70 [0.61, 0.81]	0.52 [0.43, 0.63]	0.37 [0.29, 0.44]	0.64 [0.61, 0.68]	0.28 [0.19, 0.47]	0.65 [0.21, 1.00]	0.63 [0.25, 1.00]	0.41 [0.17, 1.00]	0.92 [0.87, 1.00]
lr0.0003_wd0.01_h256_d2	0.73 [0.68, 0.86]	0.49 [0.27, 0.61]	0.35 [0.25, 0.57]	0.60 [0.53, 0.72]	0.21 [0.06, 0.51]	0.36 [0.05, 0.95]	0.83 [0.50, 1.00]	0.27 [0.14, 0.67]	0.96 [0.89, 1.00]
lr0.0003_wd0.01_h400_d2	0.76 [0.64, 0.88]	0.50 [0.34, 0.65]	0.35 [0.22, 0.55]	0.64 [0.53, 0.87]	0.23 [0.05, 0.53]	0.48 [0.05, 0.80]	0.80 [0.25, 1.00]	0.24 [0.14, 0.38]	0.95 [0.84, 1.00]
lr0.0003_wd0.01_h512_d2	0.72 [0.66, 0.86]	0.53 [0.29, 0.72]	0.46 [0.29, 0.67]	0.68 [0.59, 0.81]	0.35 [0.16, 0.61]	0.77 [0.50, 0.95]	0.60 [0.33, 0.75]	0.42 [0.23, 0.67]	0.91 [0.89, 0.95]
lr0.001_wd0.005_h256_d1	0.75 [0.69, 0.86]	0.60 [0.53, 0.73]	0.36 [0.29, 0.43]	0.65 [0.57, 0.79]	0.23 [0.12, 0.40]	0.46 [0.21, 0.60]	0.85 [0.75, 1.00]	0.23 [0.17, 0.27]	0.94 [0.89, 1.00]
lr0.001_wd0.005_h400_d1	0.74 [0.65, 0.84]	0.54 [0.31, 0.74]	0.42 [0.31, 0.57]	0.69 [0.62, 0.82]	0.31 [0.17, 0.51]	0.74 [0.58, 0.95]	0.63 [0.50, 1.00]	0.36 [0.20, 0.67]	0.92 [0.88, 1.00]
lr0.001_wd0.005_h512_d1	0.76 [0.64, 0.84]	0.51 [0.24, 0.68]	0.29 [0.00, 0.44]	0.61 [0.45, 0.76]	0.15 [-0.13, 0.36]	0.58 [0.35, 0.90]	0.63 [0.00, 1.00]	0.19 [0.00, 0.33]	0.90 [0.82, 1.00]
lr0.001_wd0.005_h256_d2	0.77 [0.69, 0.89]	0.51 [0.27, 0.75]	0.38 [0.18, 0.60]	0.67 [0.47, 0.84]	0.26 [-0.04, 0.52]	0.59 [0.26, 0.85]	0.75 [0.25, 1.00]	0.27 [0.14, 0.50]	0.93 [0.82, 1.00]
lr0.001_wd0.005_h400_d2	0.74 [0.64, 0.88]	0.51 [0.26, 0.77]	0.42 [0.29, 0.80]	0.63 [0.53, 0.83]	0.29 [0.04, 0.80]	0.61 [0.20, 1.00]	0.65 [0.33, 1.00]	0.42 [0.18, 1.00]	0.91 [0.86, 1.00]
lr0.001_wd0.005_h512_d2	0.79 [0.68, 0.89]	0.54 [0.26, 0.78]	0.36 [0.00, 0.60]	0.66 [0.42, 0.87]	0.24 [-0.17, 0.53]	0.62 [0.11, 0.85]	0.70 [0.00, 1.00]	0.26 [0.00, 0.50]	0.93 [0.81, 1.00]
lr0.001_wd0.01_h256_d1	0.73 [0.61, 0.84]	0.49 [0.26, 0.64]	0.35 [0.29, 0.40]	0.62 [0.55, 0.68]	0.26 [0.08, 0.47]	0.73 [0.37, 1.00]	0.52 [0.25, 1.00]	0.41 [0.20, 1.00]	0.90 [0.86, 1.00]
lr0.001_wd0.01_h400_d1	0.73 [0.62, 0.84]	0.44 [0.28, 0.65]	0.32 [0.25, 0.40]	0.60 [0.53, 0.68]	0.17 [0.04, 0.26]	0.76 [0.55, 0.89]	0.45 [0.25, 0.67]	0.27 [0.18, 0.33]	0.88 [0.85, 0.93]
lr0.001_wd0.01_h512_d1	0.80 [0.68, 0.93]	0.60 [0.29, 0.76]	0.45 [0.31, 0.67]	0.72 [0.62, 0.92]	0.35 [0.17, 0.65]	0.61 [0.25, 0.84]	0.83 [0.67, 1.00]	0.32 [0.20, 0.50]	0.96 [0.92, 1.00]
lr0.001_wd0.01_h256_d2	0.75 [0.64, 0.88]	0.56 [0.28, 0.74]	0.41 [0.31, 0.67]	0.65 [0.55, 0.75]	0.32 [0.12, 0.67]	0.63 [0.10, 1.00]	0.67 [0.33, 1.00]	0.43 [0.18, 1.00]	0.94 [0.87, 1.00]
lr0.001_wd0.01_h400_d2	0.75 [0.65, 0.89]	0.53 [0.31, 0.73]	0.39 [0.27, 0.57]	0.64 [0.50, 0.78]	0.23 [0.00, 0.50]	0.62 [0.25, 0.89]	0.67 [0.50, 0.75]	0.29 [0.17, 0.50]	0.90 [0.83, 0.94]
lr0.001_wd0.01_h512_d2	0.73 [0.66, 0.82]	0.43 [0.31, 0.59]	0.34 [0.24, 0.44]	0.61 [0.42, 0.70]	0.17 [-0.17, 0.32]	0.43 [0.10, 0.85]	0.78 [0.50, 1.00]	0.24 [0.14, 0.40]	0.90 [0.67, 1.00]
lr0.0001_wd0.0_h1024_d1	0.72 [0.62, 0.84]	0.45 [0.26, 0.62]	0.32 [0.22, 0.50]	0.61 [0.47, 0.84]	0.15 [-0.05, 0.48]	0.58 [0.20, 0.80]	0.63 [0.25, 1.00]	0.22 [0.16, 0.33]	0.88 [0.80, 1.00]
lr0.0001_wd0.0_h2048_d1	0.71 [0.57, 0.82]	0.48 [0.22, 0.62]	0.37 [0.32, 0.44]	0.64 [0.57, 0.73]	0.24 [0.12, 0.35]	0.63 [0.20, 0.89]	0.65 [0.33, 1.00]	0.29 [0.20, 0.40]	0.92 [0.89, 1.00]
lr0.0001_wd0.0_h1024_d2	0.75 [0.68, 0.86]	0.55 [0.29, 0.73]	0.38 [0.31, 0.46]	0.66 [0.55, 0.82]	0.27 [0.13, 0.44]	0.49 [0.10, 0.85]	0.83 [0.50, 1.00]	0.27 [0.18, 0.40]	0.96 [0.89, 1.00]
lr0.0001_wd0.0_h2048_d2	0.74 [0.61, 0.89]	0.54 [0.23, 0.73]	0.41 [0.31, 0.50]	0.66 [0.55, 0.75]	0.28 [0.12, 0.42]	0.61 [0.10, 0.85]	0.72 [0.50, 1.00]	0.32 [0.18, 0.40]	0.93 [0.89, 1.00]
lr0.0001_wd0.0001_h1024_d1	0.72 [0.60, 0.82]	0.50 [0.31, 0.70]	0.31 [0.20, 0.40]	0.58 [0.50, 0.65]	0.15 [0.00, 0.26]	0.61 [0.05, 0.89]	0.55 [0.25, 1.00]	0.24 [0.17, 0.33]	0.91 [0.83, 1.00]
lr0.0001_wd0.0001_h2048_d1	0.72 [0.61, 0.84]	0.47 [0.29, 0.67]	0.35 [0.29, 0.40]	0.63 [0.59, 0.70]	0.21 [0.15, 0.30]	0.61 [0.25, 0.84]	0.65 [0.33, 1.00]	0.25 [0.21, 0.29]	0.92 [0.88, 1.00]
lr0.0001_wd0.0001_h1024_d2	0.73 [0.61, 0.88]	0.51 [0.32, 0.65]	0.29 [0.24, 0.35]	0.58 [0.52, 0.62]	0.14 [0.03, 0.23]	0.70 [0.37, 0.90]	0.45 [0.25, 0.75]	0.26 [0.14, 0.33]	0.88 [0.85, 0.91]
lr0.0001_wd0.0001_h2048_d2	0.72 [0.64, 0.84]	0.51 [0.33, 0.62]	0.39 [0.36, 0.44]	0.65 [0.62, 0.68]	0.27 [0.20, 0.34]	0.76 [0.55, 0.95]	0.55 [0.33, 0.75]	0.34 [0.25, 0.50]	0.90 [0.88, 0.93]
lr0.0001_wd0.001_h1024_d1	0.75 [0.66, 0.88]	0.51 [0.31, 0.63]	0.35 [0.00, 0.60]	0.65 [0.45, 0.89]	0.24 [-0.13, 0.58]	0.76 [0.58, 0.95]	0.53 [0.00, 1.00]	0.30 [0.00, 0.67]	0.90 [0.82, 1.00]
lr0.0001_wd0.001_h2048_d1	0.73 [0.68, 0.82]	0.48 [0.26, 0.65]	0.34 [0.30, 0.40]	0.61 [0.53, 0.66]	0.21 [0.09, 0.34]	0.55 [0.05, 0.95]	0.67 [0.33, 1.00]	0.28 [0.17, 0.50]	0.93 [0.87, 1.00]
lr0.0001_wd0.001_h1024_d2	0.72 [0.60, 0.84]	0.38 [0.23, 0.57]	0.32 [0.24, 0.40]	0.59 [0.50, 0.70]	0.13 [0.00, 0.30]	0.34 [0.00, 0.74]	0.83 [0.67, 1.00]	0.21 [0.14, 0.29]	0.55 [0.00, 0.93]
lr0.0001_wd0.001_h2048_d2	0.73 [0.64, 0.82]	0.53 [0.40, 0.62]	0.38 [0.29, 0.50]	0.64 [0.55, 0.75]	0.26 [0.08, 0.42]	0.78 [0.35, 0.90]	0.50 [0.25, 0.75]	0.35 [0.19, 0.50]	0.89 [0.86, 0.94]
lr0.0003_wd0.0_h1024_d1	0.74 [0.66, 0.89]	0.48 [0.26, 0.55]	0.40 [0.29, 0.55]	0.69 [0.61, 0.87]	0.30 [0.19, 0.53]	0.54 [0.21, 0.75]	0.85 [0.50, 1.00]	0.27 [0.17, 0.38]	0.96 [0.88, 1.00]
lr0.0003_wd0.0_h2048_d1	0.73 [0.62, 0.86]	0.52 [0.24, 0.74]	0.35 [0.29, 0.44]	0.64 [0.57, 0.73]	0.22 [0.16, 0.35]	0.65 [0.25, 0.90]	0.62 [0.25, 1.00]	0.27 [0.21, 0.33]	0.92 [0.86, 1.00]
lr0.0003_wd0.0_h1024_d2	0.73 [0.64, 0.81]	0.47 [0.28, 0.73]	0.37 [0.29, 0.50]	0.65 [0.59, 0.70]	0.26 [0.15, 0.40]	0.59 [0.37, 0.90]	0.72 [0.33, 1.00]	0.28 [0.20, 0.50]	0.94 [0.89, 1.00]
lr0.0003_wd0.0_h2048_d2	0.75 [0.69, 0.84]	0.51 [0.30, 0.65]	0.38 [0.20, 0.57]	0.65 [0.50, 0.78]	0.27 [0.00, 0.50]	0.72 [0.32, 0.95]	0.58 [0.25, 1.00]	0.34 [0.17, 0.50]	0.91 [0.83, 1.00]
lr0.0003_wd0.0001_h1024_d1	0.75 [0.68, 0.86]	0.50 [0.33, 0.74]	0.43 [0.31, 0.50]	0.72 [0.62, 0.84]	0.32 [0.17, 0.48]	0.65 [0.55, 0.75]	0.78 [0.67, 1.00]	0.30 [0.20, 0.38]	0.94 [0.92, 1.00]
lr0.0003_wd0.0001_h2048_d1	0.72 [0.61, 0.82]	0.53 [0.41, 0.74]	0.36 [0.25, 0.40]	0.62 [0.55, 0.68]	0.27 [0.10, 0.47]	0.85 [0.68, 1.00]	0.40 [0.25, 0.67]	0.46 [0.25, 1.00]	0.89 [0.85, 0.93]
lr0.0003_wd0.0001_h1024_d2	0.75 [0.61, 0.82]	0.53 [0.37, 0.73]	0.32 [0.17, 0.44]	0.58 [0.45, 0.70]	0.14 [-0.08, 0.32]	0.67 [0.20, 0.89]	0.50 [0.25, 0.75]	0.26 [0.12, 0.40]	0.87 [0.80, 0.93]
lr0.0003_wd0.0001_h2048_d2	0.76 [0.68, 0.88]	0.46 [0.32, 0.65]	0.43 [0.32, 0.67]	0.67 [0.57, 0.75]	0.38 [0.17, 0.67]	0.65 [0.15, 1.00]	0.68 [0.25, 1.00]	0.54 [0.19, 1.00]	0.94 [0.87, 1.00]
lr0.0003_wd0.001_h1024_d1	0.72 [0.68, 0.81]	0.49 [0.29, 0.61]	0.34 [0.25, 0.42]	0.62 [0.54, 0.72]	0.23 [0.06, 0.47]	0.57 [0.35, 1.00]	0.67 [0.25, 1.00]	0.37 [0.15, 1.00]	0.91 [0.87, 1.00]
lr0.0003_wd0.001_h2048_d1	0.73 [0.70, 0.81]	0.50 [0.35, 0.62]	0.47 [0.38, 0.67]	0.67 [0.62, 0.75]	0.45 [0.22, 0.67]	0.90 [0.55, 1.00]	0.43 [0.25, 0.75]	0.75 [0.25, 1.00]	0.90 [0.87, 0.92]
lr0.0003_wd0.001_h1024_d2	0.77 [0.61, 0.89]	0.51 [0.25, 0.62]	0.31 [0.22, 0.40]	0.59 [0.53, 0.64]	0.18 [0.05, 0.34]	0.51 [0.16, 0.95]	0.67 [0.25, 1.00]	0.25 [0.16, 0.50]	0.93 [0.84, 1.00]
lr0.0003_wd0.001_h2048_d2	0.73 [0.64, 0.91]	0.56 [0.31, 0.72]	0.49 [0.35, 0.67]	0.71 [0.62, 0.82]	0.40 [0.19, 0.60]	0.82 [0.50, 0.95]	0.60 [0.33, 0.75]	0.47 [0.23, 0.67]	0.92 [0.90, 0.95]
lr0.001_wd0.0_h1024_d1	0.72 [0.64, 0.81]	0.51 [0.36, 0.73]	0.40 [0.33, 0.44]	0.66 [0.60, 0.73]	0.30 [0.23, 0.35]	0.87 [0.79, 0.95]	0.45 [0.25, 0.67]	0.39 [0.33, 0.50]	0.90 [0.86, 0.94]
lr0.001_wd0.0_h2048_d1	0.73 [0.60, 0.82]	0.52 [0.24, 0.74]	0.40 [0.30, 0.57]	0.66 [0.57, 0.73]	0.28 [0.12, 0.51]	0.59 [0.26, 0.95]	0.73 [0.50, 1.00]	0.33 [0.18, 0.67]	0.93 [0.89, 1.00]
lr0.001_wd0.0_h1024_d2	0.77 [0.65, 0.93]	0.59 [0.32, 0.81]	0.47 [0.40, 0.57]	0.72 [0.65, 0.84]	0.38 [0.26, 0.51]	0.82 [0.68, 0.95]	0.63 [0.50, 1.00]	0.41 [0.33, 0.67]	0.92 [0.89, 1.00]
lr0.001_wd0.0_h2048_d2	0.79 [0.72, 0.91]	0.56 [0.34, 0.70]	0.37 [0.00, 0.67]	0.69 [0.50, 0.81]	0.31 [0.00, 0.61]	0.69 [0.42, 1.00]	0.68 [0.00, 1.00]	0.29 [0.00, 0.67]	0.94 [0.83, 1.00]
lr0.001_wd0.0001_h1024_d1	0.75 [0.62, 0.84]	0.49 [0.25, 0.73]	0.33 [0.00, 0.67]	0.62 [0.47, 0.81]	0.21 [-0.09, 0.61]	0.76 [0.45, 0.95]	0.48 [0.00, 0.75]	0.29 [0.00, 0.67]	0.90 [0.86, 0.95]
lr0.001_wd0.0001_h2048_d1	0.74 [0.61, 0.81]	0.55 [0.27, 0.73]	0.42 [0.32, 0.57]	0.68 [0.57, 0.76]	0.32 [0.17, 0.51]	0.63 [0.15, 0.95]	0.73 [0.50, 1.00]	0.35 [0.19, 0.67]	0.95 [0.89, 1.00]
lr0.001_wd0.0001_h1024_d2	0.80 [0.70, 0.86]	0.63 [0.37, 0.81]	0.42 [0.33, 0.75]	0.68 [0.60, 0.85]	0.31 [0.15, 0.70]	0.53 [0.20, 0.95]	0.83 [0.67, 1.00]	0.32 [0.20, 0.75]	0.96 [0.90, 1.00]

Supplementary Table 24: Grid search results on the classification task on GDHCM prospective dataset (choosing 40% as the threshold for clinical variables selection). Reported metrics are AUC, AUPRC, F1, Balanced Accuracy, MCC, Specificity, Sensitivity, PPV, and NPV.

Config	AUC	AUPRC	F1	Balanced_Accuracy	MCC	Specificity	Sensitivity	PPV	NPV
lr0.001_wd0.0001_h2048_d2	0.78 [0.70, 0.86]	0.57 [0.40, 0.68]	0.39 [0.27, 0.57]	0.65 [0.55, 0.75]	0.27 [0.08, 0.51]	0.54 [0.16, 0.95]	0.75 [0.50, 1.00]	0.32 [0.16, 0.67]	0.94 [0.86, 1.00]
lr0.001_wd0.001_h1024_d1	0.73 [0.61, 0.86]	0.47 [0.27, 0.63]	0.37 [0.33, 0.43]	0.65 [0.60, 0.70]	0.24 [0.15, 0.30]	0.59 [0.37, 0.89]	0.72 [0.33, 1.00]	0.26 [0.20, 0.33]	0.93 [0.89, 1.00]
lr0.001_wd0.001_h2048_d1	0.76 [0.64, 0.88]	0.58 [0.48, 0.75]	0.36 [0.31, 0.43]	0.63 [0.57, 0.70]	0.25 [0.12, 0.34]	0.77 [0.63, 0.95]	0.50 [0.25, 0.75]	0.35 [0.22, 0.50]	0.90 [0.86, 0.93]
lr0.001_wd0.001_h1024_d2	0.74 [0.60, 0.89]	0.51 [0.23, 0.68]	0.32 [0.17, 0.50]	0.57 [0.45, 0.70]	0.15 [-0.08, 0.40]	0.58 [0.00, 0.95]	0.57 [0.25, 1.00]	0.29 [0.12, 0.50]	0.70 [0.00, 0.90]
lr0.001_wd0.001_h2048_d2	0.73 [0.61, 0.86]	0.54 [0.43, 0.73]	0.31 [0.20, 0.40]	0.60 [0.50, 0.70]	0.17 [0.00, 0.30]	0.76 [0.55, 0.90]	0.45 [0.25, 0.75]	0.26 [0.17, 0.33]	0.89 [0.83, 0.93]
lr0.0001_wd0.0_h256_d3	0.75 [0.70, 0.84]	0.40 [0.27, 0.65]	0.21 [0.00, 0.30]	0.51 [0.50, 0.53]	0.02 [0.00, 0.09]	0.21 [0.00, 1.00]	0.80 [0.00, 1.00]	0.12 [0.00, 0.17]	0.37 [0.00, 1.00]
lr0.0001_wd0.0_h400_d3	0.71 [0.58, 0.81]	0.44 [0.23, 0.65]	0.31 [0.24, 0.40]	0.57 [0.50, 0.65]	0.13 [0.00, 0.26]	0.48 [0.00, 0.89]	0.67 [0.33, 1.00]	0.24 [0.14, 0.33]	0.71 [0.00, 0.89]
lr0.0001_wd0.0_h512_d3	0.71 [0.64, 0.82]	0.50 [0.28, 0.61]	0.30 [0.21, 0.38]	0.56 [0.46, 0.65]	0.09 [-0.05, 0.23]	0.46 [0.00, 0.89]	0.65 [0.33, 1.00]	0.22 [0.13, 0.33]	0.70 [0.00, 0.92]
lr0.0001_wd0.0_h256_d4	0.73 [0.64, 0.82]	0.45 [0.29, 0.56]	0.28 [0.25, 0.31]	0.54 [0.50, 0.61]	0.08 [0.00, 0.19]	0.15 [0.00, 0.42]	0.93 [0.67, 1.00]	0.17 [0.15, 0.18]	0.58 [0.00, 1.00]
lr0.0001_wd0.0_h400_d4	0.74 [0.65, 0.89]	0.45 [0.29, 0.62]	0.33 [0.24, 0.57]	0.57 [0.50, 0.73]	0.14 [0.00, 0.51]	0.23 [0.00, 0.95]	0.90 [0.50, 1.00]	0.26 [0.14, 0.67]	0.38 [0.00, 1.00]
lr0.0001_wd0.0_h512_d4	0.75 [0.61, 0.88]	0.53 [0.39, 0.74]	0.33 [0.24, 0.55]	0.59 [0.50, 0.87]	0.14 [0.00, 0.53]	0.33 [0.00, 0.90]	0.85 [0.25, 1.00]	0.24 [0.14, 0.38]	0.37 [0.00, 1.00]
lr0.0001_wd0.0001_h256_d3	0.71 [0.64, 0.84]	0.47 [0.30, 0.63]	0.31 [0.25, 0.44]	0.57 [0.45, 0.73]	0.11 [-0.10, 0.35]	0.36 [0.00, 0.79]	0.78 [0.50, 1.00]	0.21 [0.15, 0.33]	0.71 [0.00, 1.00]
lr0.0001_wd0.0001_h400_d3	0.72 [0.58, 0.89]	0.50 [0.41, 0.61]	0.22 [0.00, 0.33]	0.54 [0.50, 0.61]	0.08 [0.00, 0.23]	0.63 [0.00, 1.00]	0.45 [0.00, 1.00]	0.18 [0.00, 0.33]	0.69 [0.00, 0.89]
lr0.0001_wd0.0001_h512_d3	0.73 [0.65, 0.89]	0.56 [0.25, 0.73]	0.32 [0.00, 0.57]	0.58 [0.40, 0.73]	0.20 [-0.27, 0.55]	0.65 [0.05, 1.00]	0.52 [0.00, 1.00]	0.40 [0.00, 1.00]	0.83 [0.50, 1.00]
lr0.0001_wd0.0001_h256_d4	0.73 [0.65, 0.86]	0.50 [0.29, 0.61]	0.32 [0.24, 0.40]	0.60 [0.50, 0.70]	0.16 [0.00, 0.30]	0.57 [0.00, 0.85]	0.63 [0.25, 1.00]	0.23 [0.14, 0.29]	0.72 [0.00, 0.93]
lr0.0001_wd0.0001_h400_d4	0.71 [0.60, 0.84]	0.42 [0.26, 0.61]	0.25 [0.00, 0.44]	0.55 [0.50, 0.73]	0.08 [0.00, 0.35]	0.47 [0.00, 1.00]	0.63 [0.00, 1.00]	0.16 [0.00, 0.33]	0.52 [0.00, 0.94]
lr0.0001_wd0.0001_h512_d4	0.72 [0.58, 0.88]	0.50 [0.22, 0.72]	0.25 [0.00, 0.44]	0.56 [0.50, 0.68]	0.12 [0.00, 0.32]	0.76 [0.25, 1.00]	0.37 [0.00, 0.75]	0.22 [0.00, 0.40]	0.87 [0.83, 0.89]
lr0.0001_wd0.001_h256_d3	0.73 [0.65, 0.84]	0.37 [0.27, 0.57]	0.23 [0.00, 0.44]	0.53 [0.48, 0.68]	0.06 [-0.04, 0.32]	0.51 [0.00, 1.00]	0.55 [0.00, 1.00]	0.17 [0.00, 0.40]	0.52 [0.00, 0.89]
lr0.0001_wd0.001_h400_d3	0.74 [0.69, 0.88]	0.51 [0.28, 0.72]	0.32 [0.29, 0.40]	0.59 [0.50, 0.68]	0.15 [0.00, 0.26]	0.42 [0.00, 0.89]	0.75 [0.33, 1.00]	0.23 [0.17, 0.33]	0.75 [0.00, 1.00]
lr0.0001_wd0.001_h512_d3	0.73 [0.63, 0.82]	0.48 [0.30, 0.73]	0.24 [0.00, 0.57]	0.57 [0.45, 0.78]	0.12 [-0.13, 0.50]	0.74 [0.00, 1.00]	0.40 [0.00, 1.00]	0.20 [0.00, 0.50]	0.70 [0.00, 0.94]
lr0.0001_wd0.001_h256_d4	0.71 [0.60, 0.89]	0.52 [0.26, 0.72]	0.37 [0.24, 0.57]	0.59 [0.43, 0.78]	0.16 [-0.17, 0.50]	0.53 [0.00, 0.95]	0.65 [0.33, 1.00]	0.31 [0.14, 0.50]	0.68 [0.00, 0.94]
lr0.0001_wd0.001_h400_d4	0.71 [0.61, 0.91]	0.41 [0.30, 0.55]	0.25 [0.00, 0.40]	0.54 [0.50, 0.64]	0.10 [0.00, 0.34]	0.42 [0.00, 1.00]	0.67 [0.00, 1.00]	0.20 [0.00, 0.50]	0.55 [0.00, 1.00]
lr0.0001_wd0.001_h512_d4	0.72 [0.59, 0.81]	0.42 [0.22, 0.65]	0.29 [0.24, 0.40]	0.53 [0.50, 0.63]	0.09 [0.00, 0.47]	0.20 [0.00, 1.00]	0.85 [0.25, 1.00]	0.32 [0.14, 1.00]	0.17 [0.00, 0.87]
lr0.0003_wd0.0_h256_d3	0.77 [0.61, 0.89]	0.52 [0.24, 0.70]	0.27 [0.24, 0.30]	0.51 [0.50, 0.53]	0.03 [0.00, 0.09]	0.07 [0.00, 0.30]	0.95 [0.75, 1.00]	0.16 [0.14, 0.18]	0.37 [0.00, 1.00]
lr0.0003_wd0.0_h400_d3	0.73 [0.66, 0.86]	0.51 [0.26, 0.64]	0.40 [0.18, 0.67]	0.61 [0.46, 0.75]	0.28 [-0.05, 0.67]	0.76 [0.26, 1.00]	0.45 [0.25, 0.67]	0.50 [0.13, 1.00]	0.87 [0.82, 0.91]
lr0.0003_wd0.0_h512_d3	0.76 [0.66, 0.88]	0.51 [0.28, 0.64]	0.40 [0.27, 0.57]	0.63 [0.50, 0.78]	0.26 [0.00, 0.50]	0.62 [0.15, 0.95]	0.65 [0.33, 1.00]	0.35 [0.17, 0.50]	0.91 [0.83, 1.00]
lr0.0003_wd0.0_h256_d4	0.75 [0.68, 0.91]	0.45 [0.30, 0.56]	0.39 [0.27, 0.67]	0.63 [0.50, 0.81]	0.23 [0.00, 0.61]	0.58 [0.00, 0.95]	0.67 [0.50, 1.00]	0.32 [0.17, 0.67]	0.72 [0.00, 0.95]
lr0.0003_wd0.0_h400_d4	0.75 [0.65, 0.89]	0.53 [0.27, 0.73]	0.32 [0.24, 0.44]	0.58 [0.50, 0.73]	0.13 [0.00, 0.35]	0.48 [0.00, 0.85]	0.68 [0.25, 1.00]	0.23 [0.14, 0.33]	0.53 [0.00, 0.94]
lr0.0003_wd0.0_h512_d4	0.76 [0.66, 0.93]	0.56 [0.28, 0.73]	0.40 [0.29, 0.57]	0.65 [0.50, 0.78]	0.26 [0.00, 0.50]	0.52 [0.00, 0.90]	0.77 [0.50, 1.00]	0.31 [0.17, 0.50]	0.75 [0.00, 1.00]
lr0.0003_wd0.0001_h256_d3	0.75 [0.63, 0.86]	0.48 [0.26, 0.64]	0.26 [0.24, 0.29]	0.50 [0.50, 0.50]	0.00 [0.00, 0.00]	0.05 [0.00, 0.25]	0.95 [0.75, 1.00]	0.15 [0.14, 0.17]	0.17 [0.00, 0.83]
lr0.0003_wd0.0001_h400_d3	0.75 [0.63, 0.84]	0.54 [0.27, 0.73]	0.33 [0.25, 0.50]	0.58 [0.45, 0.75]	0.15 [-0.10, 0.42]	0.56 [0.05, 0.89]	0.60 [0.25, 1.00]	0.26 [0.15, 0.40]	0.89 [0.75, 1.00]
lr0.0003_wd0.0001_h512_d3	0.74 [0.61, 0.84]	0.49 [0.30, 0.64]	0.39 [0.24, 0.57]	0.62 [0.50, 0.75]	0.23 [0.00, 0.51]	0.51 [0.00, 0.95]	0.73 [0.50, 1.00]	0.33 [0.14, 0.67]	0.55 [0.00, 0.94]
lr0.0003_wd0.0001_h256_d4	0.74 [0.61, 0.82]	0.52 [0.24, 0.73]	0.32 [0.00, 0.67]	0.60 [0.50, 0.81]	0.18 [0.00, 0.61]	0.51 [0.00, 1.00]	0.68 [0.00, 1.00]	0.25 [0.00, 0.67]	0.54 [0.00, 0.95]
lr0.0003_wd0.0001_h400_d4	0.77 [0.69, 0.86]	0.54 [0.34, 0.73]	0.45 [0.29, 0.67]	0.66 [0.50, 0.81]	0.30 [0.00, 0.61]	0.55 [0.00, 0.95]	0.77 [0.50, 1.00]	0.37 [0.17, 0.67]	0.76 [0.00, 1.00]
lr0.0003_wd0.0001_h512_d4	0.74 [0.66, 0.89]	0.43 [0.30, 0.53]	0.32 [0.24, 0.40]	0.57 [0.50, 0.65]	0.14 [0.00, 0.34]	0.52 [0.00, 0.95]	0.62 [0.25, 1.00]	0.28 [0.14, 0.50]	0.53 [0.00, 0.90]
lr0.0003_wd0.001_h256_d3	0.73 [0.63, 0.84]	0.50 [0.27, 0.62]	0.35 [0.30, 0.50]	0.61 [0.53, 0.75]	0.20 [0.09, 0.42]	0.28 [0.05, 0.84]	0.93 [0.67, 1.00]	0.23 [0.17, 0.40]	0.99 [0.94, 1.00]
lr0.0003_wd0.001_h400_d3	0.74 [0.63, 0.88]	0.51 [0.35, 0.61]	0.34 [0.24, 0.55]	0.61 [0.50, 0.87]	0.16 [0.00, 0.53]	0.42 [0.00, 0.75]	0.80 [0.50, 1.00]	0.23 [0.14, 0.38]	0.55 [0.00, 1.00]
lr0.0003_wd0.001_h512_d3	0.75 [0.69, 0.91]	0.52 [0.29, 0.73]	0.41 [0.27, 0.60]	0.66 [0.53, 0.89]	0.31 [0.04, 0.58]	0.65 [0.10, 1.00]	0.67 [0.33, 1.00]	0.43 [0.18, 1.00]	0.93 [0.85, 1.00]
lr0.0003_wd0.001_h256_d4	0.73 [0.68, 0.82]	0.46 [0.26, 0.73]	0.40 [0.29, 0.57]	0.65 [0.50, 0.78]	0.26 [0.00, 0.50]	0.54 [0.00, 0.89]	0.77 [0.50, 1.00]	0.30 [0.17, 0.50]	0.75 [0.00, 1.00]
lr0.0003_wd0.001_h400_d4	0.77 [0.70, 0.89]	0.55 [0.29, 0.74]	0.43 [0.24, 0.67]	0.67 [0.50, 0.81]	0.34 [0.00, 0.61]	0.61 [0.00, 1.00]	0.73 [0.25, 1.00]	0.47 [0.14, 1.00]	0.75 [0.00, 1.00]
lr0.0003_wd0.001_h512_d4	0.76 [0.69, 0.86]	0.57 [0.34, 0.74]	0.39 [0.29, 0.50]	0.63 [0.50, 0.73]	0.28 [0.00, 0.55]	0.62 [0.00, 1.00]	0.63 [0.33, 1.00]	0.44 [0.17, 1.00]	0.74 [0.00, 1.00]
lr0.001_wd0.0_h256_d3	0.78 [0.71, 0.89]	0.59 [0.35, 0.69]	0.41 [0.30, 0.67]	0.65 [0.53, 0.81]	0.28 [0.09, 0.61]	0.46 [0.05, 0.95]	0.83 [0.50, 1.00]	0.33 [0.17, 0.67]	0.97 [0.89, 1.00]
lr0.001_wd0.0_h400_d3	0.77 [0.66, 0.88]	0.54 [0.43, 0.70]	0.35 [0.30, 0.40]	0.62 [0.55, 0.66]	0.22 [0.08, 0.34]	0.58 [0.32, 0.95]	0.67 [0.33, 1.00]	0.29 [0.19, 0.50]	0.91 [0.88, 1.00]
lr0.001_wd0.0_h512_d3	0.78 [0.65, 0.89]	0.58 [0.30, 0.74]	0.42 [0.33, 0.55]	0.67 [0.60, 0.78]	0.30 [0.15, 0.45]	0.75 [0.45, 0.95]	0.60 [0.33, 0.75]	0.35 [0.21, 0.50]	0.91 [0.88, 0.94]
lr0.001_wd0.0_h256_d4	0.76 [0.69, 0.86]	0.55 [0.44, 0.74]	0.35 [0.29, 0.50]	0.64 [0.58, 0.75]	0.23 [0.15, 0.42]	0.76 [0.45, 0.90]	0.52 [0.25, 0.75]	0.31 [0.21, 0.40]	0.90 [0.86, 0.94]
lr0.001_wd0.0_h400_d4	0.77 [0.66, 0.88]	0.51 [0.28, 0.74]	0.47 [0.32, 0.67]	0.71 [0.58, 0.81]	0.38 [0.12, 0.61]	0.72 [0.40, 0.95]	0.70 [0.33, 1.00]	0.42 [0.20, 0.67]	0.94 [0.89, 1.00]
lr0.001_wd0.0_h512_d4	0.81 [0.74, 0.89]	0.53 [0.32, 0.66]	0.39 [0.29, 0.57]	0.63 [0.50, 0.73]	0.26 [0.00, 0.51]	0.57 [0.00, 0.95]	0.70 [0.33, 1.00]	0.34 [0.17, 0.67]	0.75 [0.00, 1.00]
lr0.001_wd0.0001_h256_d3	0.78 [0.69, 0.91]	0.56 [0.32, 0.70]	0.32 [0.24, 0.40]	0.58 [0.52, 0.64]	0.16 [0.03, 0.34]	0.40 [0.15, 0.95]	0.75 [0.33, 1.00]	0.24 [0.14, 0.50]	0.93 [0.86, 1.00]
lr0.001_wd0.0001_h400_d3	0.75 [0.63, 0.88]	0.51 [0.26, 0.70]	0.43 [0.29, 0.57]	0.67 [0.59, 0.78]	0.31 [0.16, 0.50]	0.84 [0.70, 0.90]	0.50 [0.33, 0.67]	0.38 [0.25, 0.50]	0.90 [0.88, 0.94]
lr0.001_wd0.0001_h512_d3	0.77 [0.58, 0.89]	0.52 [0.22, 0.68]	0.37 [0.00, 0.50]	0.64 [0.35, 0.75]	0.23 [-0.26, 0.42]	0.77 [0.65, 0.84]	0.52 [0.00, 0.75]	0.29 [0.00, 0.40]	0.90 [0.78, 0.94]
lr0.001_wd0.0001_h256_d4	0.80 [0.73, 0.91]	0.54 [0.32, 0.67]	0.36 [0.29, 0.43]	0.64 [0.50, 0.70]	0.22 [0.00, 0.32]	0.46 [0.00, 0.89]	0.82 [0.33, 1.00]	0.25 [0.17, 0.33]	0.76 [0.00, 1.00]
lr0.001_wd0.0001_h400_d4	0.81 [0.68, 0.89]	0.64 [0.33, 0.80]	0.54 [0.40, 0.80]	0.74 [0.64, 0.83]	0.47 [0.26, 0.80]	0.78 [0.45, 1.00]	0.70 [0.33, 1.00]	0.53 [0.27, 1.00]	0.94 [0.90, 1.00]
lr0.001_wd0.0001_h512_d4	0.80 [0.68, 0.93]	0.57 [0.30, 0.79]	0.33 [0.29, 0.40]	0.61 [0.50, 0.68]	0.18 [0.00, 0.26]	0.50 [0.00, 0.89]	0.72 [0.33, 1.00]	0.24 [0.17, 0.33]	0.74 [0.00, 1.00]
lr0.001_wd0.001_h256_d3	0.78 [0.67, 0.89]	0.56 [0.35, 0.76]	0.36 [0.25, 0.67]	0.59 [0.45, 0.81]	0.17 [-0.10, 0.61]	0.29 [0.00, 0.95]	0.88 [0.67, 1.00]	0.27 [0.15, 0.67]	0.74 [0.00, 1.00]
lr0.001_wd0.001_h400_d3	0.75 [0.64, 0.84]	0.55 [0.26, 0.75]	0.38 [0.17, 0.57]	0.61 [0.45, 0.78]	0.22 [-0.08, 0.51]	0.59 [0.00, 0.95]	0.63 [0.25, 1.00]	0.33 [0.13, 0.67]	0.71 [0.00, 0.94]
lr0.001_wd0.001_h512_d3	0.80 [0.72, 0.92]	0.60 [0.25, 0.71]	0.54 [0.36, 0.76]	0.73 [0.63, 0.87]	0.39 [0.15, 0.64]	0.72 [0.50, 1.00]	0.83 [0.67, 1.00]	0.52 [0.33, 1.00]	0.97 [0.93, 1.00]
lr0.001_wd0.001_h256_d4	0.76 [0.70, 0.91]	0.57 [0.30, 0.79]	0.39 [0.31, 0.57]	0.66 [0.55, 0.78]	0.26 [0.13, 0.50]	0.55 [0.10, 0.89]	0.77 [0.67, 1.00]	0.28 [0.18, 0.50]	0.94 [0.90, 1.00]
lr0.001_wd0.001_h400_d4	0.77 [0.71, 0.88]	0.59 [0.30, 0.73]	0.49 [0.33, 0.67]	0.71 [0.60, 0.79]	0.44 [0.20, 0.67]	0.71 [0.20, 1.00]	0.72 [0.33, 1.00]	0.57 [0.20, 1.00]	0.95 [0.90, 1.00]
lr0.001_wd0.001_h512_d4	0.77 [0.71, 0.86]	0.56 [0.36, 0.65]	0.42 [0.30, 0.50]	0.70 [0.55, 0.84]	0.31 [0.08, 0.48]	0.57 [0.30, 0.84]	0.83 [0.67, 1.00]	0.29 [0.19, 0.40]	0.95 [0.88, 1.00]

Supplementary Table 25: Grid search results on the classification task on GDHCM prospective dataset (choosing 40% as the threshold for clinical variables selection). Reported metrics are AUC, AUPRC, F1, Balanced Accuracy, MCC, Specificity, Sensitivity, PPV, and NPV.

Config	AUC	AUPRC	F1	Balanced_Accuracy	MCC	Specificity	Sensitivity	PPV	NPV
lr0.0001_wd0.0_h256_d1	0.65 [0.60, 0.72]	0.35 [0.29, 0.38]	0.36 [0.31, 0.40]	0.55 [0.46, 0.61]	0.10 [-0.11, 0.19]	0.38 [0.09, 0.83]	0.72 [0.31, 1.00]	0.26 [0.19, 0.33]	0.84 [0.67, 1.00]
lr0.0001_wd0.0_h400_d1	0.63 [0.56, 0.68]	0.35 [0.29, 0.42]	0.36 [0.22, 0.45]	0.56 [0.50, 0.66]	0.11 [-0.01, 0.27]	0.40 [0.04, 0.76]	0.72 [0.23, 1.00]	0.25 [0.21, 0.32]	0.87 [0.78, 1.00]
lr0.0001_wd0.0_h512_d1	0.66 [0.60, 0.70]	0.37 [0.31, 0.40]	0.36 [0.24, 0.50]	0.58 [0.50, 0.71]	0.14 [0.00, 0.36]	0.45 [0.00, 0.80]	0.71 [0.23, 1.00]	0.27 [0.21, 0.34]	0.71 [0.00, 1.00]
lr0.0001_wd0.0_h256_d2	0.68 [0.61, 0.71]	0.37 [0.33, 0.40]	0.41 [0.34, 0.52]	0.59 [0.50, 0.73]	0.16 [0.00, 0.38]	0.25 [0.00, 0.61]	0.92 [0.77, 1.00]	0.27 [0.21, 0.38]	0.56 [0.00, 1.00]
lr0.0001_wd0.0_h400_d2	0.65 [0.58, 0.71]	0.38 [0.34, 0.43]	0.38 [0.34, 0.40]	0.56 [0.50, 0.62]	0.12 [0.00, 0.31]	0.34 [0.00, 0.93]	0.78 [0.31, 1.00]	0.30 [0.21, 0.57]	0.51 [0.00, 0.88]
lr0.0001_wd0.0_h512_d2	0.68 [0.59, 0.76]	0.37 [0.30, 0.45]	0.29 [0.18, 0.36]	0.52 [0.50, 0.55]	0.06 [0.00, 0.12]	0.42 [0.00, 0.91]	0.63 [0.15, 1.00]	0.25 [0.22, 0.33]	0.69 [0.00, 1.00]
lr0.0001_wd0.0001_h256_d1	0.64 [0.58, 0.72]	0.37 [0.30, 0.41]	0.37 [0.28, 0.43]	0.54 [0.40, 0.62]	0.07 [-0.30, 0.23]	0.34 [0.02, 0.65]	0.75 [0.54, 1.00]	0.25 [0.17, 0.30]	0.80 [0.40, 1.00]
lr0.0001_wd0.0001_h400_d1	0.65 [0.60, 0.75]	0.37 [0.28, 0.47]	0.35 [0.21, 0.43]	0.56 [0.50, 0.63]	0.12 [0.00, 0.23]	0.41 [0.00, 0.91]	0.71 [0.15, 1.00]	0.27 [0.21, 0.33]	0.68 [0.00, 0.89]
lr0.0001_wd0.0001_h512_d1	0.66 [0.56, 0.76]	0.36 [0.30, 0.40]	0.38 [0.26, 0.51]	0.61 [0.54, 0.74]	0.18 [0.09, 0.39]	0.61 [0.41, 0.85]	0.61 [0.23, 0.92]	0.30 [0.26, 0.35]	0.86 [0.80, 0.96]
lr0.0001_wd0.0001_h256_d2	0.65 [0.60, 0.74]	0.40 [0.35, 0.47]	0.36 [0.34, 0.38]	0.54 [0.50, 0.61]	0.10 [0.00, 0.21]	0.35 [0.00, 0.87]	0.74 [0.31, 1.00]	0.29 [0.21, 0.40]	0.53 [0.00, 1.00]
lr0.0001_wd0.0001_h400_d2	0.67 [0.59, 0.74]	0.35 [0.31, 0.40]	0.35 [0.32, 0.37]	0.53 [0.50, 0.56]	0.05 [0.00, 0.13]	0.26 [0.00, 0.72]	0.79 [0.38, 1.00]	0.23 [0.22, 0.28]	0.50 [0.00, 0.90]
lr0.0001_wd0.0001_h512_d2	0.68 [0.59, 0.79]	0.42 [0.36, 0.51]	0.38 [0.30, 0.47]	0.55 [0.45, 0.67]	0.09 [-0.10, 0.29]	0.24 [0.00, 0.65]	0.86 [0.69, 1.00]	0.25 [0.19, 0.36]	0.70 [0.00, 1.00]
lr0.0001_wd0.001_h256_d1	0.66 [0.59, 0.78]	0.41 [0.29, 0.51]	0.36 [0.34, 0.37]	0.53 [0.50, 0.56]	0.07 [0.00, 0.11]	0.27 [0.00, 0.57]	0.80 [0.54, 1.00]	0.23 [0.21, 0.26]	0.69 [0.00, 1.00]
lr0.0001_wd0.001_h400_d1	0.68 [0.62, 0.80]	0.43 [0.32, 0.56]	0.36 [0.30, 0.44]	0.54 [0.46, 0.66]	0.03 [-0.26, 0.27]	0.28 [0.00, 0.67]	0.80 [0.38, 1.00]	0.24 [0.19, 0.30]	0.51 [0.00, 0.92]
lr0.0001_wd0.001_h512_d1	0.66 [0.58, 0.71]	0.36 [0.32, 0.42]	0.35 [0.30, 0.42]	0.54 [0.45, 0.62]	0.09 [-0.10, 0.23]	0.31 [0.00, 0.93]	0.77 [0.23, 1.00]	0.28 [0.19, 0.50]	0.68 [0.00, 1.00]
lr0.0001_wd0.001_h256_d2	0.63 [0.56, 0.66]	0.36 [0.34, 0.38]	0.35 [0.32, 0.38]	0.52 [0.50, 0.56]	0.04 [0.00, 0.10]	0.21 [0.00, 0.72]	0.83 [0.38, 1.00]	0.23 [0.21, 0.28]	0.33 [0.00, 0.84]
lr0.0001_wd0.001_h400_d2	0.65 [0.54, 0.73]	0.38 [0.31, 0.45]	0.36 [0.30, 0.41]	0.52 [0.43, 0.59]	0.03 [-0.27, 0.21]	0.09 [0.00, 0.26]	0.95 [0.83, 1.00]	0.23 [0.18, 0.26]	0.45 [0.00, 1.00]
lr0.0001_wd0.001_h512_d2	0.67 [0.60, 0.72]	0.40 [0.31, 0.53]	0.37 [0.34, 0.42]	0.53 [0.50, 0.62]	0.07 [0.00, 0.21]	0.13 [0.00, 0.39]	0.94 [0.85, 1.00]	0.23 [0.21, 0.28]	0.55 [0.00, 1.00]
lr0.0003_wd0.0_h256_d1	0.70 [0.63, 0.80]	0.40 [0.32, 0.54]	0.39 [0.34, 0.45]	0.57 [0.50, 0.67]	0.13 [0.01, 0.28]	0.31 [0.02, 0.63]	0.83 [0.54, 1.00]	0.26 [0.21, 0.31]	0.88 [0.80, 1.00]
lr0.0003_wd0.0_h400_d1	0.65 [0.54, 0.71]	0.39 [0.35, 0.46]	0.35 [0.23, 0.45]	0.56 [0.50, 0.67]	0.11 [0.00, 0.28]	0.50 [0.00, 0.76]	0.63 [0.25, 1.00]	0.26 [0.21, 0.31]	0.67 [0.00, 0.92]
lr0.0003_wd0.0_h512_d1	0.65 [0.56, 0.74]	0.37 [0.32, 0.48]	0.39 [0.33, 0.43]	0.58 [0.48, 0.62]	0.13 [-0.07, 0.21]	0.35 [0.04, 0.52]	0.81 [0.69, 0.92]	0.26 [0.20, 0.29]	0.84 [0.67, 0.90]
lr0.0003_wd0.0_h256_d2	0.66 [0.61, 0.74]	0.37 [0.32, 0.50]	0.38 [0.34, 0.42]	0.56 [0.50, 0.63]	0.13 [0.00, 0.24]	0.33 [0.00, 0.72]	0.80 [0.46, 1.00]	0.26 [0.21, 0.35]	0.73 [0.00, 1.00]
lr0.0003_wd0.0_h400_d2	0.65 [0.55, 0.75]	0.38 [0.29, 0.49]	0.32 [0.18, 0.42]	0.52 [0.50, 0.61]	0.04 [0.00, 0.19]	0.34 [0.00, 0.85]	0.70 [0.15, 1.00]	0.23 [0.21, 0.28]	0.49 [0.00, 0.89]
lr0.0003_wd0.0_h512_d2	0.66 [0.59, 0.69]	0.39 [0.31, 0.48]	0.39 [0.31, 0.48]	0.58 [0.50, 0.68]	0.15 [0.00, 0.32]	0.44 [0.00, 0.80]	0.72 [0.31, 1.00]	0.30 [0.21, 0.41]	0.71 [0.00, 1.00]
lr0.0003_wd0.0001_h256_d1	0.64 [0.55, 0.71]	0.34 [0.28, 0.41]	0.38 [0.27, 0.44]	0.60 [0.51, 0.66]	0.17 [0.02, 0.26]	0.56 [0.30, 0.78]	0.63 [0.31, 0.92]	0.29 [0.24, 0.33]	0.86 [0.79, 0.94]
lr0.0003_wd0.0001_h400_d1	0.66 [0.57, 0.81]	0.38 [0.30, 0.46]	0.38 [0.29, 0.45]	0.57 [0.45, 0.68]	0.13 [-0.09, 0.32]	0.31 [0.00, 0.72]	0.83 [0.46, 1.00]	0.26 [0.19, 0.32]	0.71 [0.00, 1.00]
lr0.0003_wd0.0001_h512_d1	0.66 [0.61, 0.75]	0.38 [0.30, 0.44]	0.37 [0.27, 0.51]	0.58 [0.50, 0.73]	0.14 [0.00, 0.37]	0.54 [0.00, 0.78]	0.63 [0.31, 1.00]	0.28 [0.21, 0.37]	0.68 [0.00, 0.93]
lr0.0003_wd0.0001_h256_d2	0.67 [0.59, 0.73]	0.39 [0.34, 0.46]	0.36 [0.32, 0.41]	0.53 [0.50, 0.60]	0.06 [0.00, 0.17]	0.25 [0.00, 0.83]	0.82 [0.31, 1.00]	0.25 [0.21, 0.33]	0.34 [0.00, 0.87]
lr0.0003_wd0.0001_h400_d2	0.67 [0.60, 0.79]	0.39 [0.32, 0.54]	0.40 [0.34, 0.47]	0.58 [0.50, 0.67]	0.14 [0.00, 0.29]	0.25 [0.00, 0.59]	0.91 [0.69, 1.00]	0.26 [0.21, 0.32]	0.56 [0.00, 1.00]
lr0.0003_wd0.0001_h512_d2	0.65 [0.54, 0.77]	0.35 [0.30, 0.46]	0.31 [0.17, 0.38]	0.53 [0.48, 0.58]	0.07 [-0.04, 0.16]	0.47 [0.00, 0.80]	0.59 [0.15, 1.00]	0.24 [0.18, 0.29]	0.68 [0.00, 1.00]
lr0.0003_wd0.001_h256_d1	0.67 [0.60, 0.79]	0.37 [0.31, 0.51]	0.39 [0.34, 0.44]	0.58 [0.51, 0.64]	0.16 [0.03, 0.23]	0.35 [0.11, 0.59]	0.81 [0.62, 1.00]	0.26 [0.21, 0.32]	0.89 [0.83, 1.00]
lr0.0003_wd0.001_h400_d1	0.66 [0.57, 0.75]	0.39 [0.28, 0.51]	0.37 [0.29, 0.46]	0.58 [0.48, 0.66]	0.13 [-0.03, 0.28]	0.53 [0.22, 0.71]	0.62 [0.38, 0.92]	0.28 [0.20, 0.36]	0.84 [0.77, 0.95]
lr0.0003_wd0.001_h512_d1	0.67 [0.55, 0.80]	0.38 [0.33, 0.51]	0.41 [0.30, 0.51]	0.60 [0.46, 0.70]	0.18 [-0.07, 0.35]	0.35 [0.11, 0.72]	0.84 [0.67, 1.00]	0.28 [0.19, 0.41]	0.90 [0.75, 1.00]
lr0.0003_wd0.001_h256_d2	0.65 [0.59, 0.75]	0.34 [0.27, 0.53]	0.32 [0.17, 0.36]	0.51 [0.48, 0.57]	0.03 [-0.04, 0.12]	0.30 [0.00, 0.80]	0.72 [0.15, 1.00]	0.22 [0.18, 0.29]	0.52 [0.00, 1.00]
lr0.0003_wd0.001_h400_d2	0.67 [0.56, 0.75]	0.37 [0.30, 0.44]	0.38 [0.34, 0.48]	0.56 [0.50, 0.68]	0.11 [0.00, 0.31]	0.37 [0.00, 0.67]	0.76 [0.46, 1.00]	0.26 [0.21, 0.33]	0.51 [0.00, 0.92]
lr0.0003_wd0.001_h512_d2	0.64 [0.58, 0.71]	0.36 [0.30, 0.44]	0.34 [0.29, 0.38]	0.50 [0.46, 0.57]	-0.00 [-0.13, 0.14]	0.16 [0.00, 0.52]	0.84 [0.46, 1.00]	0.22 [0.20, 0.24]	0.46 [0.00, 0.91]
lr0.001_wd0.0_h256_d1	0.67 [0.60, 0.73]	0.39 [0.30, 0.49]	0.40 [0.34, 0.45]	0.58 [0.50, 0.65]	0.14 [0.00, 0.25]	0.33 [0.00, 0.61]	0.83 [0.69, 1.00]	0.27 [0.21, 0.33]	0.69 [0.00, 0.91]
lr0.001_wd0.0_h400_d1	0.66 [0.58, 0.70]	0.35 [0.31, 0.43]	0.39 [0.33, 0.47]	0.59 [0.50, 0.68]	0.16 [0.01, 0.30]	0.39 [0.17, 0.76]	0.78 [0.38, 0.92]	0.27 [0.21, 0.32]	0.87 [0.80, 0.93]
lr0.001_wd0.0_h512_d1	0.67 [0.59, 0.78]	0.38 [0.33, 0.51]	0.37 [0.33, 0.46]	0.56 [0.50, 0.66]	0.13 [0.00, 0.27]	0.31 [0.00, 0.85]	0.80 [0.31, 1.00]	0.27 [0.21, 0.36]	0.74 [0.00, 1.00]
lr0.001_wd0.0_h256_d2	0.68 [0.64, 0.73]	0.42 [0.31, 0.52]	0.32 [0.11, 0.43]	0.53 [0.48, 0.63]	0.06 [-0.04, 0.27]	0.23 [0.00, 0.89]	0.82 [0.08, 1.00]	0.22 [0.17, 0.28]	0.55 [0.00, 1.00]
lr0.001_wd0.0_h400_d2	0.68 [0.63, 0.81]	0.38 [0.29, 0.47]	0.36 [0.10, 0.53]	0.59 [0.46, 0.77]	0.15 [-0.09, 0.44]	0.45 [0.00, 0.85]	0.73 [0.08, 1.00]	0.25 [0.12, 0.36]	0.71 [0.00, 1.00]
lr0.001_wd0.0_h512_d2	0.68 [0.65, 0.76]	0.37 [0.30, 0.45]	0.36 [0.27, 0.44]	0.57 [0.50, 0.65]	0.12 [0.00, 0.24]	0.47 [0.00, 0.83]	0.67 [0.31, 1.00]	0.27 [0.22, 0.33]	0.68 [0.00, 0.92]
lr0.001_wd0.0001_h256_d1	0.66 [0.56, 0.74]	0.38 [0.31, 0.47]	0.33 [0.13, 0.42]	0.57 [0.50, 0.62]	0.13 [0.00, 0.20]	0.53 [0.00, 0.98]	0.60 [0.08, 1.00]	0.32 [0.22, 0.50]	0.67 [0.00, 0.88]
lr0.001_wd0.0001_h400_d1	0.65 [0.58, 0.72]	0.40 [0.33, 0.53]	0.36 [0.31, 0.44]	0.55 [0.51, 0.64]	0.11 [0.02, 0.25]	0.44 [0.04, 0.74]	0.67 [0.46, 1.00]	0.26 [0.23, 0.37]	0.86 [0.79, 1.00]
lr0.001_wd0.0001_h512_d1	0.66 [0.59, 0.70]	0.37 [0.33, 0.41]	0.40 [0.34, 0.50]	0.58 [0.50, 0.70]	0.15 [0.00, 0.33]	0.29 [0.00, 0.63]	0.87 [0.75, 1.00]	0.27 [0.21, 0.37]	0.73 [0.00, 1.00]
lr0.001_wd0.0001_h256_d2	0.67 [0.60, 0.76]	0.39 [0.29, 0.54]	0.39 [0.34, 0.47]	0.57 [0.50, 0.67]	0.12 [0.00, 0.29]	0.27 [0.00, 0.65]	0.86 [0.62, 1.00]	0.27 [0.21, 0.36]	0.55 [0.00, 1.00]
lr0.001_wd0.0001_h400_d2	0.66 [0.57, 0.75]	0.36 [0.31, 0.43]	0.36 [0.33, 0.39]	0.53 [0.49, 0.59]	0.05 [-0.03, 0.14]	0.23 [0.00, 0.48]	0.83 [0.69, 1.00]	0.23 [0.20, 0.27]	0.65 [0.00, 0.87]
lr0.001_wd0.0001_h512_d2	0.69 [0.59, 0.82]	0.46 [0.32, 0.68]	0.36 [0.31, 0.41]	0.53 [0.46, 0.61]	0.04 [-0.11, 0.19]	0.22 [0.00, 0.61]	0.84 [0.62, 1.00]	0.24 [0.19, 0.31]	0.47 [0.00, 0.86]
lr0.001_wd0.001_h256_d1	0.69 [0.58, 0.78]	0.39 [0.31, 0.47]	0.38 [0.35, 0.43]	0.57 [0.52, 0.63]	0.14 [0.07, 0.23]	0.34 [0.04, 0.65]	0.79 [0.50, 1.00]	0.26 [0.22, 0.33]	0.90 [0.83, 1.00]
lr0.001_wd0.001_h400_d1	0.66 [0.60, 0.73]	0.36 [0.31, 0.41]	0.36 [0.32, 0.39]	0.52 [0.47, 0.58]	0.05 [-0.07, 0.14]	0.10 [0.00, 0.30]	0.94 [0.83, 1.00]	0.22 [0.20, 0.26]	0.72 [0.00, 1.00]
lr0.001_wd0.001_h512_d1	0.66 [0.58, 0.73]	0.35 [0.29, 0.44]	0.43 [0.31, 0.51]	0.63 [0.52, 0.71]	0.22 [0.04, 0.35]	0.65 [0.54, 0.76]	0.62 [0.46, 0.77]	0.33 [0.22, 0.38]	0.86 [0.81, 0.91]
lr0.001_wd0.001_h256_d2	0.66 [0.59, 0.79]	0.36 [0.29, 0.48]	0.40 [0.29, 0.49]	0.61 [0.53, 0.69]	0.20 [0.07, 0.31]	0.45 [0.20, 0.76]	0.77 [0.31, 1.00]	0.28 [0.26, 0.36]	0.91 [0.80, 1.00]
lr0.001_wd0.001_h400_d2	0.68 [0.61, 0.77]	0.38 [0.33, 0.44]	0.23 [0.00, 0.36]	0.49 [0.46, 0.51]	-0.01 [-0.11, 0.07]	0.40 [0.00, 1.00]	0.58 [0.00, 1.00]	0.17 [0.00, 0.22]	0.64 [0.00, 1.00]
lr0.001_wd0.001_h512_d2	0.69 [0.62, 0.77]	0.42 [0.32, 0.52]	0.37 [0.34, 0.39]	0.56 [0.50, 0.60]	0.12 [0.00, 0.23]	0.36 [0.00, 0.89]	0.75 [0.31, 1.00]	0.29 [0.21, 0.44]	0.53 [0.00, 1.00]

Supplementary Table 26: Grid search results on the classification task on SYSMH external validation dataset (choosing 40% as the threshold for clinical variables selection). Reported metrics are AUC, AUPRC, F1, Balanced Accuracy, MCC, Specificity, Sensitivity, PPV, and NPV.

Config	AUC	AUPRC	F1	Balanced_Accuracy	MCC	Specificity	Sensitivity	PPV	NPV
lr1e-05_wd0.005_h1024_d3	0.66 [0.57, 0.74]	0.38 [0.29, 0.45]	0.28 [0.00, 0.36]	0.52 [0.50, 0.58]	0.05 [0.00, 0.16]	0.36 [0.00, 1.00]	0.68 [0.00, 1.00]	0.19 [0.00, 0.33]	0.52 [0.00, 1.00]
lr1e-05_wd0.005_h2048_d3	0.66 [0.60, 0.73]	0.37 [0.31, 0.40]	0.38 [0.35, 0.45]	0.56 [0.50, 0.66]	0.10 [0.00, 0.26]	0.34 [0.00, 0.70]	0.78 [0.46, 1.00]	0.26 [0.21, 0.32]	0.51 [0.00, 0.89]
lr1e-05_wd0.005_h1024_d4	0.67 [0.61, 0.73]	0.38 [0.32, 0.46]	0.29 [0.00, 0.39]	0.54 [0.50, 0.60]	0.11 [0.00, 0.34]	0.43 [0.00, 1.00]	0.65 [0.00, 1.00]	0.28 [0.00, 0.75]	0.52 [0.00, 1.00]
lr1e-05_wd0.005_h2048_d4	0.67 [0.60, 0.75]	0.38 [0.35, 0.43]	0.36 [0.33, 0.39]	0.55 [0.50, 0.59]	0.13 [0.00, 0.28]	0.37 [0.00, 0.96]	0.74 [0.23, 1.00]	0.32 [0.21, 0.60]	0.53 [0.00, 1.00]
lr1e-05_wd0.01_h1024_d3	0.65 [0.57, 0.70]	0.36 [0.30, 0.48]	0.29 [0.00, 0.39]	0.52 [0.50, 0.58]	0.03 [0.00, 0.16]	0.30 [0.00, 1.00]	0.73 [0.00, 1.00]	0.18 [0.00, 0.24]	0.50 [0.00, 0.92]
lr1e-05_wd0.01_h2048_d3	0.65 [0.58, 0.70]	0.41 [0.38, 0.46]	0.21 [0.00, 0.39]	0.51 [0.46, 0.58]	0.01 [-0.11, 0.14]	0.48 [0.00, 1.00]	0.54 [0.00, 1.00]	0.13 [0.00, 0.26]	0.62 [0.00, 0.88]
lr1e-05_wd0.01_h1024_d4	0.65 [0.60, 0.72]	0.37 [0.30, 0.45]	0.40 [0.34, 0.47]	0.58 [0.50, 0.68]	0.13 [0.00, 0.30]	0.30 [0.00, 0.53]	0.86 [0.69, 1.00]	0.27 [0.21, 0.32]	0.53 [0.00, 0.92]
lr1e-05_wd0.01_h2048_d4	0.65 [0.57, 0.74]	0.41 [0.30, 0.50]	0.30 [0.00, 0.43]	0.55 [0.50, 0.63]	0.11 [0.00, 0.27]	0.47 [0.00, 1.00]	0.63 [0.00, 1.00]	0.22 [0.00, 0.40]	0.69 [0.00, 1.00]
lr3e-05_wd0.005_h1024_d3	0.65 [0.62, 0.70]	0.36 [0.32, 0.46]	0.36 [0.29, 0.43]	0.55 [0.50, 0.63]	0.10 [0.00, 0.23]	0.32 [0.00, 0.76]	0.78 [0.31, 1.00]	0.25 [0.21, 0.33]	0.53 [0.00, 1.00]
lr3e-05_wd0.005_h2048_d3	0.66 [0.56, 0.73]	0.40 [0.34, 0.50]	0.34 [0.27, 0.43]	0.52 [0.44, 0.63]	0.00 [-0.26, 0.27]	0.22 [0.00, 0.59]	0.81 [0.38, 1.00]	0.22 [0.19, 0.28]	0.68 [0.00, 1.00]
lr3e-05_wd0.005_h1024_d4	0.67 [0.61, 0.72]	0.36 [0.32, 0.41]	0.27 [0.00, 0.36]	0.49 [0.47, 0.50]	-0.03 [-0.12, 0.00]	0.31 [0.00, 0.93]	0.68 [0.00, 1.00]	0.17 [0.00, 0.22]	0.31 [0.00, 0.78]
lr3e-05_wd0.005_h2048_d4	0.67 [0.59, 0.75]	0.45 [0.33, 0.56]	0.28 [0.00, 0.36]	0.50 [0.50, 0.51]	0.00 [0.00, 0.01]	0.22 [0.00, 1.00]	0.78 [0.00, 1.00]	0.17 [0.00, 0.22]	0.32 [0.00, 0.80]
lr3e-05_wd0.01_h1024_d3	0.63 [0.56, 0.67]	0.36 [0.30, 0.40]	0.28 [0.00, 0.36]	0.51 [0.47, 0.58]	0.01 [-0.12, 0.17]	0.36 [0.00, 0.93]	0.66 [0.00, 1.00]	0.20 [0.00, 0.36]	0.32 [0.00, 0.81]
lr3e-05_wd0.01_h2048_d3	0.68 [0.60, 0.76]	0.42 [0.35, 0.48]	0.37 [0.34, 0.43]	0.54 [0.50, 0.63]	0.08 [0.00, 0.25]	0.25 [0.00, 0.80]	0.83 [0.46, 1.00]	0.26 [0.21, 0.40]	0.34 [0.00, 0.84]
lr3e-05_wd0.01_h1024_d4	0.66 [0.59, 0.73]	0.38 [0.31, 0.49]	0.30 [0.00, 0.41]	0.54 [0.50, 0.62]	0.08 [0.00, 0.24]	0.44 [0.00, 1.00]	0.65 [0.00, 1.00]	0.22 [0.00, 0.42]	0.50 [0.00, 0.89]
lr3e-05_wd0.01_h2048_d4	0.64 [0.59, 0.69]	0.36 [0.33, 0.43]	0.31 [0.21, 0.36]	0.51 [0.50, 0.53]	0.03 [0.00, 0.09]	0.33 [0.00, 0.91]	0.69 [0.15, 1.00]	0.25 [0.21, 0.33]	0.32 [0.00, 0.80]
lr1e-05_wd0.0_h256_d1	0.66 [0.58, 0.80]	0.40 [0.33, 0.56]	0.28 [0.00, 0.37]	0.51 [0.50, 0.57]	0.02 [0.00, 0.11]	0.30 [0.00, 1.00]	0.72 [0.00, 1.00]	0.18 [0.00, 0.27]	0.32 [0.00, 0.83]
lr1e-05_wd0.0_h400_d1	0.66 [0.59, 0.73]	0.39 [0.37, 0.44]	0.28 [0.00, 0.36]	0.51 [0.50, 0.52]	0.03 [0.00, 0.10]	0.33 [0.00, 1.00]	0.69 [0.00, 1.00]	0.18 [0.00, 0.24]	0.51 [0.00, 1.00]
lr1e-05_wd0.0_h512_d1	0.68 [0.64, 0.74]	0.43 [0.34, 0.56]	0.29 [0.00, 0.37]	0.50 [0.50, 0.51]	0.01 [0.00, 0.07]	0.20 [0.00, 1.00]	0.80 [0.00, 1.00]	0.17 [0.00, 0.22]	0.36 [0.00, 1.00]
lr1e-05_wd0.0_h256_d2	0.65 [0.59, 0.71]	0.38 [0.29, 0.54]	0.21 [0.00, 0.37]	0.51 [0.50, 0.55]	0.04 [0.00, 0.10]	0.57 [0.00, 1.00]	0.46 [0.00, 1.00]	0.15 [0.00, 0.29]	0.68 [0.00, 1.00]
lr1e-05_wd0.0_h400_d2	0.64 [0.57, 0.68]	0.41 [0.34, 0.50]	0.27 [0.00, 0.36]	0.51 [0.50, 0.55]	0.02 [0.00, 0.09]	0.34 [0.00, 1.00]	0.68 [0.00, 1.00]	0.18 [0.00, 0.28]	0.32 [0.00, 0.80]
lr1e-05_wd0.0_h512_d2	0.67 [0.58, 0.74]	0.43 [0.30, 0.55]	0.21 [0.00, 0.36]	0.50 [0.50, 0.50]	0.00 [0.00, 0.00]	0.40 [0.00, 1.00]	0.60 [0.00, 1.00]	0.13 [0.00, 0.22]	0.31 [0.00, 0.79]
lr1e-05_wd0.0001_h256_d1	0.62 [0.55, 0.67]	0.39 [0.28, 0.46]	0.20 [0.00, 0.37]	0.51 [0.46, 0.57]	-0.01 [-0.26, 0.14]	0.57 [0.00, 1.00]	0.44 [0.00, 1.00]	0.15 [0.00, 0.33]	0.68 [0.00, 1.00]
lr1e-05_wd0.0001_h400_d1	0.65 [0.58, 0.73]	0.42 [0.31, 0.53]	0.27 [0.00, 0.37]	0.51 [0.50, 0.53]	0.03 [0.00, 0.10]	0.36 [0.00, 1.00]	0.66 [0.00, 1.00]	0.19 [0.00, 0.27]	0.52 [0.00, 1.00]
lr1e-05_wd0.0001_h512_d1	0.63 [0.57, 0.68]	0.36 [0.30, 0.41]	0.34 [0.29, 0.36]	0.51 [0.48, 0.56]	0.04 [-0.04, 0.15]	0.22 [0.00, 0.89]	0.80 [0.23, 1.00]	0.25 [0.21, 0.38]	0.51 [0.00, 1.00]
lr1e-05_wd0.0001_h256_d2	0.67 [0.62, 0.75]	0.41 [0.29, 0.51]	0.28 [0.00, 0.36]	0.52 [0.50, 0.58]	0.05 [0.00, 0.23]	0.39 [0.00, 1.00]	0.65 [0.00, 1.00]	0.23 [0.00, 0.50]	0.32 [0.00, 0.81]
lr1e-05_wd0.0001_h400_d2	0.64 [0.57, 0.73]	0.37 [0.30, 0.47]	0.27 [0.00, 0.36]	0.51 [0.50, 0.55]	0.02 [0.00, 0.09]	0.36 [0.00, 1.00]	0.66 [0.00, 1.00]	0.18 [0.00, 0.29]	0.32 [0.00, 0.80]
lr1e-05_wd0.0001_h512_d2	0.61 [0.58, 0.65]	0.34 [0.30, 0.39]	0.28 [0.00, 0.36]	0.50 [0.49, 0.50]	-0.01 [-0.07, 0.00]	0.20 [0.00, 0.98]	0.80 [0.00, 1.00]	0.17 [0.00, 0.22]	0.16 [0.00, 0.78]
lr1e-05_wd0.001_h256_d1	0.68 [0.58, 0.76]	0.41 [0.33, 0.47]	0.29 [0.00, 0.36]	0.51 [0.50, 0.53]	0.02 [0.00, 0.12]	0.21 [0.00, 1.00]	0.80 [0.00, 1.00]	0.17 [0.00, 0.22]	0.36 [0.00, 1.00]
lr1e-05_wd0.001_h400_d1	0.70 [0.58, 0.83]	0.40 [0.31, 0.58]	0.28 [0.00, 0.36]	0.48 [0.43, 0.50]	-0.09 [-0.26, 0.00]	0.17 [0.00, 0.87]	0.78 [0.00, 1.00]	0.17 [0.00, 0.22]	0.15 [0.00, 0.75]
lr1e-05_wd0.001_h512_d1	0.64 [0.55, 0.77]	0.35 [0.30, 0.41]	0.34 [0.30, 0.36]	0.51 [0.50, 0.52]	0.02 [0.00, 0.07]	0.13 [0.00, 0.54]	0.88 [0.46, 1.00]	0.22 [0.21, 0.23]	0.52 [0.00, 1.00]
lr1e-05_wd0.001_h256_d2	0.67 [0.63, 0.74]	0.39 [0.33, 0.48]	0.20 [0.00, 0.36]	0.51 [0.50, 0.53]	0.01 [0.00, 0.07]	0.55 [0.00, 1.00]	0.46 [0.00, 1.00]	0.14 [0.00, 0.27]	0.47 [0.00, 0.80]
lr1e-05_wd0.001_h400_d2	0.66 [0.59, 0.72]	0.39 [0.29, 0.46]	0.35 [0.34, 0.36]	0.52 [0.50, 0.59]	0.06 [0.00, 0.20]	0.18 [0.00, 0.87]	0.86 [0.31, 1.00]	0.25 [0.21, 0.40]	0.36 [0.00, 1.00]
lr1e-05_wd0.001_h512_d2	0.67 [0.60, 0.71]	0.38 [0.36, 0.45]	0.29 [0.00, 0.41]	0.52 [0.50, 0.61]	0.05 [0.00, 0.24]	0.24 [0.00, 1.00]	0.80 [0.00, 1.00]	0.18 [0.00, 0.26]	0.36 [0.00, 1.00]
lr3e-05_wd0.0_h256_d1	0.64 [0.59, 0.73]	0.40 [0.29, 0.46]	0.33 [0.27, 0.39]	0.50 [0.39, 0.56]	-0.03 [-0.36, 0.12]	0.20 [0.00, 0.65]	0.80 [0.38, 1.00]	0.22 [0.17, 0.25]	0.58 [0.00, 1.00]
lr3e-05_wd0.0_h400_d1	0.67 [0.58, 0.77]	0.39 [0.32, 0.45]	0.36 [0.30, 0.42]	0.56 [0.51, 0.62]	0.13 [0.07, 0.21]	0.38 [0.02, 0.78]	0.73 [0.31, 1.00]	0.26 [0.22, 0.32]	0.90 [0.80, 1.00]
lr3e-05_wd0.0_h512_d1	0.64 [0.59, 0.69]	0.40 [0.33, 0.45]	0.37 [0.34, 0.39]	0.55 [0.50, 0.59]	0.10 [0.00, 0.16]	0.28 [0.00, 0.57]	0.81 [0.54, 1.00]	0.24 [0.21, 0.29]	0.71 [0.00, 1.00]
lr3e-05_wd0.0_h256_d2	0.66 [0.60, 0.72]	0.41 [0.31, 0.51]	0.38 [0.34, 0.44]	0.55 [0.50, 0.64]	0.11 [0.00, 0.31]	0.27 [0.00, 0.93]	0.83 [0.31, 1.00]	0.30 [0.21, 0.57]	0.35 [0.00, 0.91]
lr3e-05_wd0.0_h400_d2	0.66 [0.57, 0.73]	0.41 [0.34, 0.51]	0.29 [0.00, 0.39]	0.52 [0.50, 0.59]	0.04 [0.00, 0.15]	0.32 [0.00, 1.00]	0.72 [0.00, 1.00]	0.19 [0.00, 0.29]	0.52 [0.00, 1.00]
lr3e-05_wd0.0_h512_d2	0.66 [0.56, 0.76]	0.37 [0.33, 0.40]	0.38 [0.30, 0.46]	0.58 [0.50, 0.69]	0.13 [0.00, 0.34]	0.44 [0.00, 0.67]	0.72 [0.38, 1.00]	0.26 [0.22, 0.30]	0.70 [0.00, 1.00]
lr3e-05_wd0.0001_h256_d1	0.65 [0.61, 0.70]	0.37 [0.31, 0.43]	0.34 [0.25, 0.38]	0.52 [0.49, 0.58]	0.04 [-0.02, 0.15]	0.26 [0.00, 0.67]	0.78 [0.31, 1.00]	0.23 [0.21, 0.25]	0.50 [0.00, 0.88]
lr3e-05_wd0.0001_h400_d1	0.65 [0.60, 0.70]	0.41 [0.30, 0.48]	0.35 [0.32, 0.38]	0.52 [0.50, 0.55]	0.03 [0.00, 0.10]	0.17 [0.00, 0.61]	0.86 [0.46, 1.00]	0.23 [0.21, 0.25]	0.33 [0.00, 0.86]
lr3e-05_wd0.0001_h512_d1	0.64 [0.59, 0.70]	0.35 [0.31, 0.38]	0.35 [0.28, 0.37]	0.52 [0.43, 0.58]	0.05 [-0.13, 0.16]	0.29 [0.00, 0.78]	0.75 [0.38, 1.00]	0.24 [0.18, 0.33]	0.67 [0.00, 1.00]
lr3e-05_wd0.0001_h256_d2	0.64 [0.60, 0.68]	0.40 [0.32, 0.55]	0.27 [0.00, 0.37]	0.51 [0.50, 0.52]	0.03 [-0.01, 0.10]	0.37 [0.00, 1.00]	0.65 [0.00, 1.00]	0.18 [0.00, 0.23]	0.68 [0.00, 1.00]
lr3e-05_wd0.0001_h400_d2	0.65 [0.61, 0.71]	0.38 [0.35, 0.43]	0.36 [0.31, 0.43]	0.53 [0.50, 0.63]	0.06 [0.00, 0.21]	0.25 [0.00, 0.70]	0.82 [0.38, 1.00]	0.24 [0.21, 0.31]	0.33 [0.00, 0.87]
lr3e-05_wd0.0001_h512_d2	0.65 [0.56, 0.73]	0.37 [0.32, 0.40]	0.36 [0.28, 0.50]	0.53 [0.40, 0.69]	0.04 [-0.30, 0.33]	0.30 [0.00, 0.72]	0.77 [0.38, 1.00]	0.26 [0.17, 0.39]	0.62 [0.00, 1.00]
lr3e-05_wd0.001_h256_d1	0.66 [0.63, 0.71]	0.37 [0.34, 0.44]	0.29 [0.00, 0.36]	0.51 [0.49, 0.54]	0.02 [-0.07, 0.12]	0.27 [0.00, 0.98]	0.75 [0.00, 1.00]	0.18 [0.00, 0.24]	0.52 [0.00, 1.00]
lr3e-05_wd0.001_h400_d1	0.67 [0.61, 0.75]	0.36 [0.31, 0.42]	0.33 [0.26, 0.36]	0.50 [0.50, 0.50]	0.00 [0.00, 0.00]	0.14 [0.00, 0.70]	0.86 [0.31, 1.00]	0.22 [0.21, 0.22]	0.16 [0.00, 0.78]
lr3e-05_wd0.001_h512_d1	0.65 [0.62, 0.69]	0.38 [0.33, 0.43]	0.35 [0.17, 0.48]	0.55 [0.48, 0.69]	0.10 [-0.04, 0.32]	0.32 [0.00, 0.80]	0.78 [0.15, 1.00]	0.24 [0.18, 0.33]	0.72 [0.00, 1.00]
lr3e-05_wd0.001_h256_d2	0.64 [0.61, 0.66]	0.34 [0.30, 0.42]	0.36 [0.34, 0.40]	0.55 [0.50, 0.61]	0.09 [0.00, 0.18]	0.36 [0.00, 0.76]	0.74 [0.38, 1.00]	0.26 [0.21, 0.31]	0.50 [0.00, 0.89]
lr3e-05_wd0.001_h400_d2	0.68 [0.63, 0.77]	0.40 [0.32, 0.47]	0.37 [0.34, 0.40]	0.55 [0.50, 0.60]	0.10 [0.00, 0.18]	0.25 [0.00, 0.59]	0.85 [0.62, 1.00]	0.25 [0.21, 0.30]	0.54 [0.00, 1.00]
lr3e-05_wd0.001_h512_d2	0.65 [0.59, 0.71]	0.38 [0.31, 0.46]	0.36 [0.30, 0.42]	0.54 [0.50, 0.63]	0.07 [0.00, 0.23]	0.27 [0.00, 0.67]	0.81 [0.38, 1.00]	0.24 [0.21, 0.27]	0.51 [0.00, 0.94]
lr0.0001_wd0.005_h256_d1	0.64 [0.60, 0.72]	0.35 [0.29, 0.43]	0.34 [0.27, 0.39]	0.52 [0.50, 0.58]	0.05 [0.00, 0.16]	0.27 [0.00, 0.72]	0.78 [0.31, 1.00]	0.23 [0.21, 0.24]	0.66 [0.00, 0.92]
lr0.0001_wd0.005_h400_d1	0.67 [0.58, 0.75]	0.40 [0.32, 0.47]	0.37 [0.27, 0.48]	0.54 [0.41, 0.69]	0.06 [-0.19, 0.32]	0.22 [0.02, 0.56]	0.85 [0.67, 1.00]	0.24 [0.17, 0.33]	0.82 [0.60, 1.00]
lr0.0001_wd0.005_h512_d1	0.66 [0.61, 0.72]	0.37 [0.29, 0.42]	0.32 [0.15, 0.43]	0.55 [0.46, 0.64]	0.07 [-0.09, 0.23]	0.51 [0.11, 0.76]	0.58 [0.15, 0.83]	0.24 [0.15, 0.30]	0.81 [0.71, 0.91]
lr0.0001_wd0.005_h256_d2	0.66 [0.60, 0.72]	0.37 [0.30, 0.44]	0.37 [0.34, 0.43]	0.54 [0.50, 0.62]	0.07 [0.00, 0.21]	0.25 [0.00, 0.78]	0.83 [0.38, 1.00]	0.25 [0.21, 0.33]	0.34 [0.00, 0.88]
lr0.0001_wd0.005_h400_d2	0.65 [0.60, 0.70]	0.37 [0.31, 0.47]	0.31 [0.20, 0.36]	0.51 [0.45, 0.57]	0.03 [-0.10, 0.14]	0.41 [0.00, 0.89]	0.62 [0.15, 1.00]	0.25 [0.19, 0.31]	0.63 [0.00, 0.83]
lr0.0001_wd0.005_h512_d2	0.64 [0.61, 0.68]	0.35 [0.31, 0.41]	0.37 [0.34, 0.41]	0.53 [0.49, 0.60]	0.06 [-0.03, 0.17]	0.14 [0.00, 0.43]	0.92 [0.77, 1.00]	0.23 [0.21, 0.28]	0.50 [0.00, 0.90]
lr0.0001_wd0.01_h256_d1	0.65 [0.60, 0.70]	0.35 [0.31, 0.44]	0.37 [0.30, 0.44]	0.54 [0.44, 0.64]	0.06 [-0.20, 0.23]	0.20 [0.00, 0.59]	0.87 [0.69, 1.00]	0.24 [0.19, 0.32]	0.63 [0.00, 1.00]
lr0.0001_wd0.01_h400_d1	0.68 [0.57, 0.75]	0.43 [0.33, 0.48]	0.40 [0.30, 0.44]	0.59 [0.45, 0.66]	0.18 [-0.10, 0.27]	0.44 [0.15, 0.91]	0.75 [0.31, 1.00]	0.31 [0.19, 0.50]	0.87 [0.70, 1.00]

Supplementary Table 27: Grid search results on the classification task on SYSMH external validation dataset (choosing 40% as the threshold for clinical variables selection). Reported metrics are AUC, AUPRC, F1, Balanced Accuracy, MCC, Specificity, Sensitivity, PPV, and NPV.

Config	AUC	AUPRC	F1	Balanced_Accuracy	MCC	Specificity	Sensitivity	PPV	NPV
lr0.0001_wd0.01_h512_d1	0.66 [0.59, 0.70]	0.39 [0.29, 0.51]	0.39 [0.34, 0.46]	0.57 [0.50, 0.66]	0.13 [0.00, 0.27]	0.24 [0.00, 0.70]	0.89 [0.62, 1.00]	0.26 [0.21, 0.36]	0.74 [0.00, 1.00]
lr0.0001_wd0.01_h256_d2	0.66 [0.59, 0.70]	0.33 [0.31, 0.34]	0.34 [0.21, 0.43]	0.54 [0.50, 0.63]	0.09 [0.00, 0.23]	0.33 [0.00, 0.91]	0.75 [0.15, 1.00]	0.26 [0.21, 0.33]	0.53 [0.00, 1.00]
lr0.0001_wd0.01_h400_d2	0.66 [0.57, 0.74]	0.36 [0.33, 0.40]	0.38 [0.34, 0.44]	0.55 [0.50, 0.64]	0.10 [0.00, 0.25]	0.25 [0.00, 0.67]	0.86 [0.62, 1.00]	0.26 [0.21, 0.35]	0.54 [0.00, 1.00]
lr0.0001_wd0.01_h512_d2	0.65 [0.56, 0.74]	0.41 [0.30, 0.46]	0.29 [0.00, 0.39]	0.51 [0.48, 0.57]	0.03 [-0.07, 0.18]	0.24 [0.00, 1.00]	0.78 [0.00, 1.00]	0.18 [0.00, 0.25]	0.69 [0.00, 1.00]
lr0.0003_wd0.005_h256_d1	0.65 [0.55, 0.79]	0.36 [0.30, 0.58]	0.38 [0.27, 0.47]	0.59 [0.52, 0.67]	0.17 [0.04, 0.29]	0.42 [0.13, 0.85]	0.76 [0.25, 1.00]	0.28 [0.23, 0.32]	0.89 [0.81, 1.00]
lr0.0003_wd0.005_h400_d1	0.67 [0.55, 0.76]	0.40 [0.35, 0.44]	0.40 [0.28, 0.52]	0.58 [0.43, 0.76]	0.15 [-0.13, 0.42]	0.42 [0.02, 0.76]	0.75 [0.54, 1.00]	0.28 [0.18, 0.39]	0.87 [0.69, 1.00]
lr0.0003_wd0.005_h512_d1	0.66 [0.56, 0.75]	0.38 [0.30, 0.51]	0.33 [0.00, 0.52]	0.59 [0.47, 0.71]	0.16 [-0.12, 0.37]	0.63 [0.13, 0.93]	0.55 [0.00, 1.00]	0.26 [0.00, 0.42]	0.87 [0.77, 1.00]
lr0.0003_wd0.005_h256_d2	0.66 [0.61, 0.71]	0.36 [0.34, 0.37]	0.36 [0.32, 0.44]	0.54 [0.47, 0.64]	0.07 [-0.07, 0.24]	0.28 [0.00, 0.87]	0.80 [0.31, 1.00]	0.26 [0.20, 0.40]	0.49 [0.00, 0.91]
lr0.0003_wd0.005_h400_d2	0.68 [0.65, 0.72]	0.38 [0.31, 0.50]	0.39 [0.34, 0.45]	0.56 [0.50, 0.65]	0.12 [0.00, 0.25]	0.20 [0.00, 0.61]	0.92 [0.69, 1.00]	0.25 [0.21, 0.33]	0.56 [0.00, 1.00]
lr0.0003_wd0.005_h512_d2	0.68 [0.59, 0.74]	0.39 [0.32, 0.47]	0.37 [0.27, 0.49]	0.57 [0.50, 0.71]	0.13 [0.00, 0.35]	0.41 [0.00, 0.87]	0.74 [0.23, 1.00]	0.28 [0.21, 0.33]	0.52 [0.00, 0.96]
lr0.0003_wd0.01_h256_d1	0.67 [0.59, 0.74]	0.37 [0.31, 0.46]	0.37 [0.32, 0.42]	0.55 [0.47, 0.62]	0.09 [-0.14, 0.21]	0.29 [0.02, 0.70]	0.81 [0.46, 1.00]	0.25 [0.20, 0.30]	0.82 [0.50, 1.00]
lr0.0003_wd0.01_h400_d1	0.68 [0.57, 0.77]	0.38 [0.33, 0.50]	0.37 [0.33, 0.41]	0.54 [0.50, 0.61]	0.09 [0.00, 0.18]	0.24 [0.00, 0.52]	0.84 [0.58, 1.00]	0.24 [0.22, 0.29]	0.71 [0.00, 1.00]
lr0.0003_wd0.01_h512_d1	0.64 [0.59, 0.68]	0.34 [0.31, 0.39]	0.38 [0.34, 0.43]	0.56 [0.50, 0.62]	0.10 [0.00, 0.21]	0.33 [0.00, 0.76]	0.78 [0.46, 1.00]	0.26 [0.21, 0.35]	0.51 [0.00, 0.88]
lr0.0003_wd0.01_h256_d2	0.67 [0.56, 0.78]	0.43 [0.29, 0.58]	0.33 [0.29, 0.36]	0.50 [0.47, 0.53]	0.00 [-0.05, 0.07]	0.21 [0.00, 0.76]	0.79 [0.31, 1.00]	0.22 [0.20, 0.27]	0.31 [0.00, 0.80]
lr0.0003_wd0.01_h400_d2	0.68 [0.60, 0.75]	0.40 [0.33, 0.49]	0.40 [0.33, 0.55]	0.60 [0.50, 0.73]	0.22 [0.01, 0.40]	0.52 [0.09, 0.98]	0.67 [0.23, 1.00]	0.39 [0.21, 0.75]	0.87 [0.80, 1.00]
lr0.0003_wd0.01_h512_d2	0.66 [0.54, 0.79]	0.40 [0.29, 0.52]	0.35 [0.18, 0.46]	0.55 [0.50, 0.68]	0.09 [0.00, 0.29]	0.39 [0.00, 0.85]	0.72 [0.15, 1.00]	0.25 [0.21, 0.33]	0.51 [0.00, 0.90]
lr0.001_wd0.005_h256_d1	0.66 [0.60, 0.72]	0.39 [0.34, 0.48]	0.36 [0.34, 0.39]	0.52 [0.48, 0.59]	0.04 [-0.06, 0.14]	0.16 [0.00, 0.48]	0.89 [0.69, 1.00]	0.23 [0.21, 0.27]	0.47 [0.00, 0.86]
lr0.001_wd0.005_h400_d1	0.65 [0.60, 0.74]	0.40 [0.28, 0.52]	0.38 [0.30, 0.41]	0.57 [0.50, 0.61]	0.13 [-0.00, 0.19]	0.40 [0.22, 0.61]	0.75 [0.58, 0.92]	0.26 [0.21, 0.31]	0.86 [0.79, 0.92]
lr0.001_wd0.005_h512_d1	0.67 [0.56, 0.77]	0.42 [0.33, 0.51]	0.37 [0.35, 0.41]	0.55 [0.50, 0.62]	0.10 [0.00, 0.23]	0.31 [0.00, 0.78]	0.79 [0.46, 1.00]	0.27 [0.21, 0.38]	0.54 [0.00, 1.00]
lr0.001_wd0.005_h256_d2	0.69 [0.59, 0.73]	0.40 [0.28, 0.58]	0.36 [0.21, 0.44]	0.56 [0.50, 0.65]	0.12 [0.00, 0.28]	0.43 [0.00, 0.91]	0.70 [0.15, 1.00]	0.29 [0.21, 0.40]	0.51 [0.00, 0.90]
lr0.001_wd0.005_h400_d2	0.66 [0.58, 0.75]	0.39 [0.32, 0.48]	0.36 [0.32, 0.41]	0.56 [0.50, 0.61]	0.12 [0.00, 0.21]	0.49 [0.00, 0.83]	0.64 [0.31, 1.00]	0.27 [0.22, 0.33]	0.68 [0.00, 0.93]
lr0.001_wd0.005_h512_d2	0.67 [0.58, 0.75]	0.39 [0.31, 0.54]	0.32 [0.00, 0.50]	0.56 [0.50, 0.69]	0.10 [0.00, 0.33]	0.43 [0.00, 1.00]	0.69 [0.00, 1.00]	0.22 [0.00, 0.39]	0.51 [0.00, 0.89]
lr0.001_wd0.01_h256_d1	0.67 [0.63, 0.72]	0.45 [0.39, 0.50]	0.40 [0.34, 0.44]	0.58 [0.50, 0.65]	0.16 [0.00, 0.25]	0.29 [0.00, 0.71]	0.87 [0.58, 1.00]	0.27 [0.21, 0.35]	0.74 [0.00, 1.00]
lr0.001_wd0.01_h400_d1	0.66 [0.54, 0.79]	0.41 [0.31, 0.64]	0.39 [0.24, 0.53]	0.59 [0.50, 0.72]	0.16 [0.00, 0.37]	0.47 [0.00, 0.80]	0.72 [0.23, 1.00]	0.29 [0.21, 0.43]	0.70 [0.00, 0.95]
lr0.001_wd0.01_h512_d1	0.68 [0.62, 0.77]	0.37 [0.30, 0.48]	0.42 [0.34, 0.48]	0.61 [0.50, 0.68]	0.19 [0.00, 0.32]	0.43 [0.00, 0.80]	0.78 [0.54, 1.00]	0.30 [0.21, 0.44]	0.71 [0.00, 0.95]
lr0.001_wd0.01_h256_d2	0.68 [0.59, 0.76]	0.39 [0.32, 0.50]	0.35 [0.21, 0.44]	0.56 [0.50, 0.66]	0.12 [0.00, 0.29]	0.35 [0.00, 0.91]	0.77 [0.15, 1.00]	0.27 [0.21, 0.33]	0.53 [0.00, 1.00]
lr0.001_wd0.01_h400_d2	0.67 [0.60, 0.74]	0.41 [0.35, 0.49]	0.38 [0.35, 0.41]	0.55 [0.50, 0.61]	0.11 [0.00, 0.24]	0.18 [0.00, 0.43]	0.92 [0.77, 1.00]	0.24 [0.21, 0.28]	0.74 [0.00, 1.00]
lr0.001_wd0.01_h512_d2	0.67 [0.64, 0.72]	0.38 [0.30, 0.48]	0.36 [0.21, 0.44]	0.60 [0.53, 0.66]	0.18 [0.09, 0.28]	0.68 [0.39, 0.91]	0.51 [0.15, 0.83]	0.32 [0.26, 0.40]	0.85 [0.79, 0.92]
lr0.0001_wd0.0_h1024_d1	0.66 [0.59, 0.70]	0.38 [0.32, 0.45]	0.40 [0.34, 0.44]	0.58 [0.53, 0.65]	0.18 [0.07, 0.28]	0.38 [0.07, 0.83]	0.79 [0.46, 1.00]	0.28 [0.23, 0.43]	0.92 [0.83, 1.00]
lr0.0001_wd0.0_h2048_d1	0.69 [0.63, 0.78]	0.39 [0.31, 0.47]	0.36 [0.30, 0.43]	0.56 [0.50, 0.63]	0.13 [0.00, 0.27]	0.33 [0.00, 0.91]	0.78 [0.23, 1.00]	0.28 [0.21, 0.43]	0.73 [0.00, 1.00]
lr0.0001_wd0.0_h1024_d2	0.68 [0.61, 0.71]	0.42 [0.35, 0.51]	0.37 [0.31, 0.50]	0.55 [0.50, 0.70]	0.08 [0.00, 0.33]	0.28 [0.00, 0.70]	0.81 [0.38, 1.00]	0.25 [0.21, 0.37]	0.50 [0.00, 0.91]
lr0.0001_wd0.0_h2048_d2	0.66 [0.63, 0.72]	0.39 [0.32, 0.61]	0.36 [0.33, 0.39]	0.56 [0.50, 0.61]	0.10 [0.00, 0.21]	0.44 [0.00, 0.80]	0.67 [0.38, 1.00]	0.27 [0.21, 0.36]	0.67 [0.00, 0.88]
lr0.0001_wd0.0001_h1024_d1	0.66 [0.60, 0.75]	0.37 [0.30, 0.48]	0.34 [0.09, 0.47]	0.57 [0.45, 0.68]	0.10 [-0.11, 0.30]	0.54 [0.17, 0.83]	0.59 [0.08, 0.85]	0.25 [0.11, 0.32]	0.83 [0.73, 0.92]
lr0.0001_wd0.0001_h2048_d1	0.65 [0.58, 0.72]	0.40 [0.31, 0.46]	0.36 [0.30, 0.41]	0.53 [0.44, 0.59]	0.04 [-0.20, 0.18]	0.16 [0.00, 0.48]	0.89 [0.69, 1.00]	0.23 [0.19, 0.27]	0.65 [0.00, 1.00]
lr0.0001_wd0.0001_h1024_d2	0.66 [0.59, 0.72]	0.34 [0.27, 0.39]	0.33 [0.11, 0.41]	0.55 [0.49, 0.62]	0.10 [-0.01, 0.21]	0.40 [0.00, 0.91]	0.70 [0.08, 1.00]	0.24 [0.20, 0.30]	0.70 [0.00, 1.00]
lr0.0001_wd0.0001_h2048_d2	0.68 [0.64, 0.76]	0.41 [0.33, 0.61]	0.36 [0.34, 0.37]	0.51 [0.50, 0.57]	0.02 [0.00, 0.12]	0.12 [0.00, 0.61]	0.91 [0.54, 1.00]	0.23 [0.21, 0.28]	0.16 [0.00, 0.82]
lr0.0001_wd0.001_h1024_d1	0.66 [0.60, 0.75]	0.40 [0.34, 0.54]	0.38 [0.34, 0.44]	0.56 [0.50, 0.65]	0.11 [0.00, 0.24]	0.27 [0.00, 0.74]	0.85 [0.46, 1.00]	0.26 [0.21, 0.33]	0.54 [0.00, 1.00]
lr0.0001_wd0.001_h2048_d1	0.66 [0.60, 0.71]	0.36 [0.28, 0.46]	0.43 [0.36, 0.50]	0.63 [0.54, 0.71]	0.21 [0.07, 0.34]	0.47 [0.30, 0.72]	0.78 [0.62, 0.92]	0.30 [0.24, 0.38]	0.88 [0.82, 0.95]
lr0.0001_wd0.001_h1024_d2	0.64 [0.60, 0.68]	0.36 [0.31, 0.50]	0.37 [0.34, 0.40]	0.55 [0.50, 0.62]	0.16 [0.00, 0.44]	0.29 [0.00, 1.00]	0.82 [0.23, 1.00]	0.38 [0.21, 1.00]	0.74 [0.00, 1.00]
lr0.0001_wd0.001_h2048_d2	0.66 [0.57, 0.70]	0.36 [0.29, 0.38]	0.42 [0.39, 0.45]	0.63 [0.60, 0.66]	0.23 [0.17, 0.27]	0.56 [0.41, 0.76]	0.70 [0.46, 0.85]	0.32 [0.26, 0.37]	0.88 [0.83, 0.91]
lr0.0003_wd0.0_h1024_d1	0.66 [0.59, 0.71]	0.41 [0.33, 0.54]	0.37 [0.30, 0.43]	0.56 [0.46, 0.64]	0.11 [-0.07, 0.27]	0.36 [0.22, 0.53]	0.76 [0.54, 1.00]	0.25 [0.19, 0.30]	0.85 [0.75, 1.00]
lr0.0003_wd0.0_h2048_d1	0.66 [0.59, 0.71]	0.36 [0.33, 0.39]	0.37 [0.30, 0.44]	0.58 [0.50, 0.65]	0.14 [0.00, 0.24]	0.51 [0.13, 0.85]	0.64 [0.31, 1.00]	0.28 [0.22, 0.36]	0.87 [0.78, 1.00]
lr0.0003_wd0.0_h1024_d2	0.68 [0.59, 0.72]	0.42 [0.30, 0.50]	0.40 [0.36, 0.45]	0.60 [0.50, 0.66]	0.18 [0.00, 0.27]	0.52 [0.00, 0.78]	0.68 [0.42, 1.00]	0.31 [0.22, 0.38]	0.69 [0.00, 0.90]
lr0.0003_wd0.0_h2048_d2	0.68 [0.63, 0.76]	0.39 [0.34, 0.49]	0.32 [0.11, 0.42]	0.54 [0.49, 0.61]	0.08 [-0.01, 0.22]	0.34 [0.00, 0.91]	0.74 [0.08, 1.00]	0.23 [0.20, 0.27]	0.71 [0.00, 1.00]
lr0.0003_wd0.0001_h1024_d1	0.65 [0.57, 0.72]	0.39 [0.31, 0.54]	0.39 [0.32, 0.48]	0.60 [0.55, 0.68]	0.20 [0.10, 0.31]	0.45 [0.17, 0.91]	0.76 [0.25, 1.00]	0.30 [0.24, 0.43]	0.90 [0.82, 1.00]
lr0.0003_wd0.0001_h2048_d1	0.63 [0.55, 0.68]	0.33 [0.28, 0.43]	0.40 [0.35, 0.43]	0.60 [0.53, 0.63]	0.17 [0.06, 0.23]	0.44 [0.22, 0.65]	0.75 [0.62, 0.92]	0.28 [0.22, 0.33]	0.87 [0.83, 0.93]
lr0.0003_wd0.0001_h1024_d2	0.68 [0.56, 0.83]	0.38 [0.31, 0.56]	0.34 [0.27, 0.43]	0.53 [0.43, 0.63]	0.06 [-0.12, 0.21]	0.35 [0.00, 0.91]	0.70 [0.23, 1.00]	0.27 [0.18, 0.43]	0.48 [0.00, 0.87]
lr0.0003_wd0.0001_h2048_d2	0.66 [0.56, 0.76]	0.36 [0.29, 0.50]	0.38 [0.32, 0.44]	0.57 [0.48, 0.64]	0.14 [-0.07, 0.28]	0.43 [0.04, 0.83]	0.72 [0.38, 1.00]	0.28 [0.20, 0.43]	0.84 [0.67, 1.00]
lr0.0003_wd0.001_h1024_d1	0.65 [0.55, 0.71]	0.40 [0.32, 0.50]	0.41 [0.34, 0.44]	0.60 [0.50, 0.64]	0.18 [0.00, 0.25]	0.35 [0.00, 0.61]	0.86 [0.62, 1.00]	0.27 [0.21, 0.31]	0.73 [0.00, 0.94]
lr0.0003_wd0.001_h2048_d1	0.64 [0.54, 0.71]	0.37 [0.27, 0.48]	0.35 [0.32, 0.39]	0.57 [0.53, 0.59]	0.12 [0.05, 0.16]	0.59 [0.22, 0.83]	0.54 [0.31, 0.92]	0.28 [0.24, 0.33]	0.84 [0.80, 0.91]
lr0.0003_wd0.001_h1024_d2	0.67 [0.59, 0.77]	0.36 [0.29, 0.47]	0.37 [0.32, 0.42]	0.55 [0.47, 0.62]	0.08 [-0.14, 0.22]	0.27 [0.00, 0.74]	0.83 [0.38, 1.00]	0.25 [0.20, 0.29]	0.64 [0.00, 1.00]
lr0.0003_wd0.001_h2048_d2	0.69 [0.64, 0.76]	0.39 [0.30, 0.54]	0.33 [0.18, 0.43]	0.54 [0.50, 0.64]	0.07 [0.00, 0.23]	0.36 [0.00, 0.85]	0.72 [0.15, 1.00]	0.24 [0.21, 0.29]	0.50 [0.00, 0.91]
lr0.001_wd0.0_h1024_d1	0.66 [0.59, 0.72]	0.36 [0.28, 0.48]	0.38 [0.34, 0.42]	0.57 [0.50, 0.61]	0.12 [0.00, 0.19]	0.38 [0.00, 0.80]	0.75 [0.38, 1.00]	0.27 [0.21, 0.36]	0.69 [0.00, 0.90]
lr0.001_wd0.0_h2048_d1	0.69 [0.60, 0.73]	0.38 [0.31, 0.51]	0.29 [0.00, 0.39]	0.51 [0.48, 0.55]	0.03 [-0.07, 0.16]	0.31 [0.11, 0.98]	0.72 [0.00, 1.00]	0.18 [0.00, 0.24]	0.84 [0.75, 1.00]
lr0.001_wd0.0_h1024_d2	0.68 [0.59, 0.75]	0.43 [0.32, 0.67]	0.45 [0.36, 0.58]	0.64 [0.53, 0.74]	0.27 [0.12, 0.47]	0.58 [0.07, 0.89]	0.69 [0.50, 1.00]	0.37 [0.23, 0.58]	0.90 [0.84, 1.00]
lr0.001_wd0.0_h2048_d2	0.69 [0.65, 0.78]	0.40 [0.33, 0.52]	0.40 [0.34, 0.47]	0.58 [0.50, 0.67]	0.16 [0.00, 0.28]	0.33 [0.00, 0.80]	0.83 [0.46, 1.00]	0.28 [0.21, 0.40]	0.73 [0.00, 1.00]
lr0.001_wd0.0001_h1024_d1	0.68 [0.61, 0.75]	0.40 [0.36, 0.49]	0.41 [0.34, 0.51]	0.59 [0.50, 0.73]	0.16 [0.00, 0.37]	0.36 [0.00, 0.67]	0.83 [0.54, 1.00]	0.28 [0.21, 0.37]	0.70 [0.00, 0.93]
lr0.001_wd0.0001_h2048_d1	0.65 [0.61, 0.72]	0.34 [0.30, 0.39]	0.42 [0.40, 0.46]	0.63 [0.61, 0.67]	0.23 [0.19, 0.29]	0.53 [0.31, 0.80]	0.73 [0.46, 0.92]	0.31 [0.26, 0.40]	0.89 [0.84, 0.95]
lr0.001_wd0.0001_h1024_d2	0.70 [0.55, 0.80]	0.39 [0.32, 0.50]	0.29 [0.00, 0.40]	0.50 [0.46, 0.59]	-0.05 [-0.26, 0.16]	0.27 [0.00, 0.93]	0.74 [0.00, 1.00]	0.18 [0.00, 0.27]	0.33 [0.00, 0.86]

Supplementary Table 28: Grid search results on the classification task on SYSMH external validation dataset (choosing 40% as the threshold for clinical variables selection). Reported metrics are AUC, AUPRC, F1, Balanced Accuracy, MCC, Specificity, Sensitivity, PPV, and NPV.

Config	AUC	AUPRC	F1	Balanced_Accuracy	MCC	Specificity	Sensitivity	PPV	NPV
lr0.001_wd0.0001_h2048_d2	0.68 [0.59, 0.79]	0.41 [0.28, 0.57]	0.37 [0.31, 0.43]	0.56 [0.50, 0.63]	0.10 [0.00, 0.22]	0.33 [0.00, 0.61]	0.78 [0.50, 1.00]	0.25 [0.21, 0.31]	0.71 [0.00, 1.00]
lr0.001_wd0.001_h1024_d1	0.64 [0.55, 0.70]	0.37 [0.29, 0.42]	0.28 [0.00, 0.40]	0.51 [0.42, 0.58]	0.02 [-0.13, 0.17]	0.39 [0.00, 0.98]	0.64 [0.00, 1.00]	0.18 [0.00, 0.26]	0.66 [0.00, 0.92]
lr0.001_wd0.001_h2048_d1	0.67 [0.65, 0.73]	0.40 [0.30, 0.55]	0.31 [0.12, 0.38]	0.54 [0.50, 0.61]	0.08 [0.00, 0.21]	0.50 [0.00, 0.93]	0.58 [0.08, 1.00]	0.27 [0.21, 0.38]	0.65 [0.00, 0.83]
lr0.001_wd0.001_h1024_d2	0.67 [0.60, 0.74]	0.41 [0.32, 0.58]	0.38 [0.35, 0.44]	0.56 [0.50, 0.64]	0.11 [0.00, 0.24]	0.27 [0.00, 0.61]	0.86 [0.58, 1.00]	0.25 [0.21, 0.30]	0.53 [0.00, 0.91]
lr0.001_wd0.001_h2048_d2	0.67 [0.61, 0.72]	0.40 [0.34, 0.48]	0.41 [0.29, 0.57]	0.61 [0.50, 0.75]	0.20 [0.00, 0.44]	0.61 [0.00, 0.78]	0.62 [0.31, 1.00]	0.33 [0.22, 0.45]	0.68 [0.00, 0.92]
lr0.0001_wd0.0_h256_d3	0.67 [0.62, 0.73]	0.36 [0.30, 0.47]	0.36 [0.34, 0.40]	0.52 [0.50, 0.60]	0.03 [0.00, 0.17]	0.12 [0.00, 0.59]	0.92 [0.62, 1.00]	0.23 [0.21, 0.30]	0.17 [0.00, 0.84]
lr0.0001_wd0.0_h400_d3	0.67 [0.60, 0.76]	0.42 [0.34, 0.48]	0.38 [0.35, 0.43]	0.55 [0.50, 0.63]	0.08 [0.00, 0.21]	0.22 [0.00, 0.57]	0.87 [0.67, 1.00]	0.25 [0.21, 0.31]	0.35 [0.00, 0.87]
lr0.0001_wd0.0_h512_d3	0.68 [0.57, 0.76]	0.45 [0.37, 0.54]	0.36 [0.30, 0.44]	0.54 [0.49, 0.64]	0.07 [-0.03, 0.25]	0.29 [0.00, 0.67]	0.78 [0.38, 1.00]	0.25 [0.20, 0.35]	0.68 [0.00, 1.00]
lr0.0001_wd0.0_h256_d4	0.67 [0.57, 0.76]	0.42 [0.33, 0.48]	0.38 [0.34, 0.45]	0.56 [0.50, 0.66]	0.12 [0.00, 0.27]	0.39 [0.00, 0.89]	0.73 [0.31, 1.00]	0.29 [0.21, 0.44]	0.51 [0.00, 0.90]
lr0.0001_wd0.0_h400_d4	0.68 [0.62, 0.75]	0.39 [0.33, 0.48]	0.38 [0.34, 0.43]	0.55 [0.50, 0.63]	0.09 [0.00, 0.23]	0.21 [0.00, 0.65]	0.88 [0.62, 1.00]	0.25 [0.21, 0.33]	0.54 [0.00, 1.00]
lr0.0001_wd0.0_h512_d4	0.66 [0.58, 0.72]	0.35 [0.30, 0.40]	0.29 [0.00, 0.41]	0.53 [0.45, 0.61]	0.04 [-0.16, 0.21]	0.40 [0.00, 0.89]	0.66 [0.00, 1.00]	0.20 [0.00, 0.33]	0.50 [0.00, 0.93]
lr0.0001_wd0.0001_h256_d3	0.68 [0.63, 0.76]	0.36 [0.33, 0.43]	0.37 [0.34, 0.41]	0.54 [0.50, 0.60]	0.09 [0.00, 0.18]	0.14 [0.00, 0.35]	0.94 [0.85, 1.00]	0.23 [0.21, 0.27]	0.55 [0.00, 1.00]
lr0.0001_wd0.0001_h400_d3	0.65 [0.56, 0.73]	0.38 [0.31, 0.46]	0.28 [0.00, 0.36]	0.50 [0.50, 0.50]	0.00 [0.00, 0.00]	0.20 [0.00, 1.00]	0.80 [0.00, 1.00]	0.17 [0.00, 0.22]	0.16 [0.00, 0.78]
lr0.0001_wd0.0001_h512_d3	0.66 [0.61, 0.77]	0.38 [0.32, 0.44]	0.35 [0.22, 0.45]	0.55 [0.50, 0.67]	0.11 [0.00, 0.29]	0.34 [0.00, 0.93]	0.77 [0.15, 1.00]	0.27 [0.21, 0.40]	0.52 [0.00, 0.95]
lr0.0001_wd0.0001_h256_d4	0.66 [0.61, 0.71]	0.40 [0.34, 0.49]	0.28 [0.00, 0.38]	0.53 [0.50, 0.59]	0.08 [0.00, 0.28]	0.41 [0.00, 1.00]	0.65 [0.00, 1.00]	0.25 [0.00, 0.60]	0.52 [0.00, 1.00]
lr0.0001_wd0.0001_h400_d4	0.67 [0.62, 0.73]	0.39 [0.31, 0.48]	0.34 [0.19, 0.43]	0.54 [0.50, 0.65]	0.07 [0.00, 0.26]	0.31 [0.00, 0.87]	0.77 [0.15, 1.00]	0.24 [0.21, 0.28]	0.51 [0.00, 0.94]
lr0.0001_wd0.0001_h512_d4	0.68 [0.59, 0.74]	0.37 [0.33, 0.39]	0.40 [0.34, 0.51]	0.56 [0.50, 0.70]	0.11 [0.00, 0.35]	0.27 [0.00, 0.72]	0.86 [0.62, 1.00]	0.27 [0.21, 0.41]	0.35 [0.00, 0.89]
lr0.0001_wd0.001_h256_d3	0.67 [0.61, 0.72]	0.38 [0.36, 0.40]	0.34 [0.30, 0.36]	0.51 [0.50, 0.57]	0.04 [0.00, 0.18]	0.18 [0.00, 0.91]	0.85 [0.23, 1.00]	0.26 [0.21, 0.43]	0.16 [0.00, 0.81]
lr0.0001_wd0.001_h400_d3	0.66 [0.59, 0.74]	0.40 [0.34, 0.50]	0.37 [0.24, 0.51]	0.57 [0.50, 0.74]	0.15 [0.00, 0.39]	0.46 [0.00, 0.93]	0.69 [0.23, 1.00]	0.32 [0.21, 0.57]	0.52 [0.00, 0.96]
lr0.0001_wd0.001_h512_d3	0.67 [0.60, 0.75]	0.39 [0.32, 0.45]	0.36 [0.31, 0.42]	0.54 [0.50, 0.64]	0.08 [0.00, 0.24]	0.24 [0.00, 0.57]	0.84 [0.46, 1.00]	0.24 [0.21, 0.28]	0.52 [0.00, 0.94]
lr0.0001_wd0.001_h256_d4	0.66 [0.62, 0.69]	0.36 [0.34, 0.39]	0.40 [0.34, 0.49]	0.58 [0.50, 0.68]	0.15 [0.00, 0.31]	0.26 [0.00, 0.67]	0.91 [0.69, 1.00]	0.27 [0.21, 0.38]	0.56 [0.00, 1.00]
lr0.0001_wd0.001_h400_d4	0.67 [0.58, 0.78]	0.38 [0.31, 0.50]	0.36 [0.34, 0.40]	0.53 [0.50, 0.60]	0.06 [0.00, 0.17]	0.27 [0.00, 0.76]	0.80 [0.38, 1.00]	0.25 [0.21, 0.31]	0.33 [0.00, 0.84]
lr0.0001_wd0.001_h512_d4	0.68 [0.60, 0.74]	0.40 [0.28, 0.53]	0.35 [0.30, 0.37]	0.53 [0.50, 0.59]	0.06 [0.00, 0.15]	0.30 [0.00, 0.78]	0.76 [0.31, 1.00]	0.25 [0.21, 0.29]	0.53 [0.00, 1.00]
lr0.0003_wd0.0_h256_d3	0.67 [0.61, 0.71]	0.35 [0.32, 0.39]	0.39 [0.34, 0.44]	0.57 [0.50, 0.66]	0.13 [0.00, 0.26]	0.41 [0.00, 0.80]	0.73 [0.38, 1.00]	0.29 [0.21, 0.36]	0.51 [0.00, 0.88]
lr0.0003_wd0.0_h400_d3	0.69 [0.64, 0.74]	0.40 [0.34, 0.50]	0.37 [0.34, 0.44]	0.54 [0.50, 0.64]	0.07 [0.00, 0.25]	0.24 [0.00, 0.74]	0.85 [0.54, 1.00]	0.25 [0.21, 0.37]	0.34 [0.00, 0.85]
lr0.0003_wd0.0_h512_d3	0.70 [0.65, 0.79]	0.42 [0.33, 0.57]	0.39 [0.34, 0.47]	0.56 [0.50, 0.67]	0.10 [0.00, 0.29]	0.23 [0.00, 0.65]	0.89 [0.69, 1.00]	0.26 [0.21, 0.36]	0.35 [0.00, 0.88]
lr0.0003_wd0.0_h256_d4	0.69 [0.60, 0.82]	0.39 [0.31, 0.54]	0.40 [0.36, 0.49]	0.58 [0.50, 0.71]	0.13 [0.00, 0.35]	0.28 [0.00, 0.54]	0.87 [0.69, 1.00]	0.26 [0.22, 0.33]	0.53 [0.00, 0.96]
lr0.0003_wd0.0_h400_d4	0.69 [0.63, 0.77]	0.39 [0.33, 0.45]	0.37 [0.22, 0.52]	0.58 [0.50, 0.73]	0.15 [0.00, 0.38]	0.44 [0.00, 0.93]	0.72 [0.15, 1.00]	0.31 [0.21, 0.40]	0.52 [0.00, 0.93]
lr0.0003_wd0.0_h512_d4	0.71 [0.65, 0.78]	0.42 [0.34, 0.54]	0.39 [0.33, 0.54]	0.56 [0.50, 0.76]	0.11 [0.00, 0.42]	0.27 [0.00, 0.74]	0.86 [0.38, 1.00]	0.26 [0.21, 0.38]	0.35 [0.00, 0.96]
lr0.0003_wd0.0001_h256_d3	0.69 [0.58, 0.75]	0.42 [0.35, 0.46]	0.33 [0.00, 0.56]	0.56 [0.50, 0.74]	0.11 [0.00, 0.41]	0.36 [0.00, 1.00]	0.75 [0.00, 1.00]	0.22 [0.00, 0.43]	0.54 [0.00, 1.00]
lr0.0003_wd0.0001_h400_d3	0.65 [0.61, 0.71]	0.41 [0.33, 0.48]	0.34 [0.29, 0.37]	0.53 [0.50, 0.59]	0.05 [0.00, 0.18]	0.31 [0.00, 0.80]	0.74 [0.31, 1.00]	0.25 [0.21, 0.36]	0.32 [0.00, 0.82]
lr0.0003_wd0.0001_h512_d3	0.67 [0.59, 0.72]	0.39 [0.32, 0.47]	0.32 [0.10, 0.44]	0.53 [0.46, 0.64]	0.05 [-0.09, 0.25]	0.43 [0.00, 0.85]	0.64 [0.08, 1.00]	0.23 [0.13, 0.35]	0.49 [0.00, 0.86]
lr0.0003_wd0.0001_h256_d4	0.68 [0.59, 0.74]	0.38 [0.35, 0.44]	0.30 [0.00, 0.41]	0.53 [0.47, 0.61]	0.05 [-0.12, 0.18]	0.35 [0.00, 0.93]	0.72 [0.00, 1.00]	0.19 [0.00, 0.29]	0.51 [0.00, 0.92]
lr0.0003_wd0.0001_h400_d4	0.69 [0.62, 0.78]	0.41 [0.34, 0.52]	0.39 [0.34, 0.45]	0.56 [0.50, 0.66]	0.10 [0.00, 0.27]	0.18 [0.00, 0.58]	0.93 [0.75, 1.00]	0.25 [0.21, 0.32]	0.37 [0.00, 0.94]
lr0.0003_wd0.0001_h512_d4	0.68 [0.60, 0.71]	0.36 [0.30, 0.44]	0.32 [0.10, 0.41]	0.55 [0.46, 0.61]	0.08 [-0.09, 0.18]	0.46 [0.00, 0.85]	0.63 [0.08, 1.00]	0.23 [0.13, 0.32]	0.67 [0.00, 0.89]
lr0.0003_wd0.001_h256_d3	0.70 [0.64, 0.77]	0.41 [0.30, 0.52]	0.41 [0.34, 0.49]	0.61 [0.50, 0.69]	0.20 [0.00, 0.31]	0.59 [0.00, 0.87]	0.64 [0.31, 1.00]	0.34 [0.21, 0.43]	0.69 [0.00, 0.90]
lr0.0003_wd0.001_h400_d3	0.67 [0.59, 0.75]	0.38 [0.33, 0.53]	0.30 [0.00, 0.44]	0.57 [0.50, 0.65]	0.13 [0.00, 0.27]	0.64 [0.00, 1.00]	0.49 [0.00, 1.00]	0.26 [0.00, 0.50]	0.66 [0.00, 0.89]
lr0.0003_wd0.001_h512_d3	0.69 [0.58, 0.75]	0.38 [0.34, 0.46]	0.35 [0.32, 0.41]	0.56 [0.50, 0.62]	0.11 [0.00, 0.23]	0.45 [0.00, 0.93]	0.66 [0.23, 1.00]	0.31 [0.21, 0.50]	0.50 [0.00, 0.87]
lr0.0003_wd0.001_h256_d4	0.68 [0.61, 0.75]	0.40 [0.33, 0.52]	0.40 [0.34, 0.48]	0.59 [0.50, 0.68]	0.15 [0.00, 0.31]	0.34 [0.00, 0.74]	0.83 [0.62, 1.00]	0.28 [0.21, 0.40]	0.53 [0.00, 0.95]
lr0.0003_wd0.001_h400_d4	0.68 [0.60, 0.74]	0.42 [0.34, 0.52]	0.30 [0.00, 0.44]	0.53 [0.50, 0.65]	0.06 [0.00, 0.28]	0.36 [0.00, 1.00]	0.70 [0.00, 1.00]	0.21 [0.00, 0.40]	0.33 [0.00, 0.86]
lr0.0003_wd0.001_h512_d4	0.68 [0.64, 0.74]	0.40 [0.32, 0.49]	0.28 [0.00, 0.38]	0.52 [0.50, 0.57]	0.05 [0.00, 0.18]	0.37 [0.00, 1.00]	0.68 [0.00, 1.00]	0.19 [0.00, 0.28]	0.52 [0.00, 1.00]
lr0.001_wd0.0_h256_d3	0.70 [0.65, 0.75]	0.41 [0.33, 0.50]	0.37 [0.34, 0.44]	0.54 [0.50, 0.64]	0.08 [0.00, 0.28]	0.29 [0.00, 0.83]	0.80 [0.46, 1.00]	0.27 [0.21, 0.43]	0.33 [0.00, 0.84]
lr0.001_wd0.0_h400_d3	0.68 [0.64, 0.76]	0.37 [0.31, 0.45]	0.28 [0.00, 0.39]	0.53 [0.50, 0.59]	0.06 [0.00, 0.15]	0.47 [0.00, 1.00]	0.59 [0.00, 1.00]	0.20 [0.00, 0.31]	0.49 [0.00, 0.84]
lr0.001_wd0.0_h512_d3	0.69 [0.63, 0.76]	0.42 [0.29, 0.59]	0.40 [0.33, 0.46]	0.60 [0.50, 0.67]	0.17 [0.00, 0.32]	0.47 [0.00, 0.74]	0.73 [0.46, 1.00]	0.29 [0.22, 0.33]	0.71 [0.00, 1.00]
lr0.001_wd0.0_h256_d4	0.69 [0.63, 0.76]	0.40 [0.30, 0.51]	0.33 [0.22, 0.36]	0.52 [0.50, 0.54]	0.04 [0.00, 0.13]	0.22 [0.00, 0.93]	0.81 [0.15, 1.00]	0.25 [0.21, 0.40]	0.33 [0.00, 0.88]
lr0.001_wd0.0_h400_d4	0.68 [0.61, 0.74]	0.42 [0.32, 0.51]	0.37 [0.33, 0.44]	0.54 [0.50, 0.64]	0.07 [0.00, 0.25]	0.25 [0.00, 0.74]	0.82 [0.54, 1.00]	0.25 [0.21, 0.37]	0.53 [0.00, 1.00]
lr0.001_wd0.0_h512_d4	0.69 [0.63, 0.75]	0.37 [0.32, 0.41]	0.31 [0.00, 0.47]	0.54 [0.50, 0.68]	0.08 [0.00, 0.30]	0.37 [0.00, 1.00]	0.72 [0.00, 1.00]	0.21 [0.00, 0.36]	0.51 [0.00, 0.89]
lr0.001_wd0.0001_h256_d3	0.67 [0.60, 0.77]	0.39 [0.29, 0.59]	0.38 [0.34, 0.44]	0.55 [0.50, 0.66]	0.09 [0.00, 0.27]	0.16 [0.00, 0.49]	0.94 [0.83, 1.00]	0.24 [0.21, 0.30]	0.53 [0.00, 0.92]
lr0.001_wd0.0001_h400_d3	0.68 [0.58, 0.78]	0.40 [0.34, 0.48]	0.39 [0.27, 0.55]	0.60 [0.46, 0.77]	0.17 [-0.26, 0.44]	0.44 [0.00, 1.00]	0.75 [0.15, 1.00]	0.43 [0.19, 1.00]	0.53 [0.00, 0.97]
lr0.001_wd0.0001_h512_d3	0.66 [0.57, 0.75]	0.39 [0.32, 0.49]	0.36 [0.17, 0.44]	0.58 [0.49, 0.67]	0.15 [-0.02, 0.31]	0.46 [0.28, 0.83]	0.70 [0.15, 1.00]	0.25 [0.20, 0.29]	0.89 [0.78, 1.00]
lr0.001_wd0.0001_h256_d4	0.68 [0.61, 0.77]	0.37 [0.31, 0.49]	0.38 [0.35, 0.46]	0.55 [0.50, 0.65]	0.11 [0.00, 0.31]	0.30 [0.00, 0.85]	0.81 [0.46, 1.00]	0.28 [0.21, 0.46]	0.54 [0.00, 1.00]
lr0.001_wd0.0001_h400_d4	0.69 [0.61, 0.77]	0.41 [0.33, 0.50]	0.39 [0.35, 0.45]	0.57 [0.50, 0.65]	0.14 [0.00, 0.25]	0.37 [0.00, 0.72]	0.78 [0.54, 1.00]	0.27 [0.22, 0.35]	0.71 [0.00, 1.00]
lr0.001_wd0.0001_h512_d4	0.70 [0.59, 0.83]	0.38 [0.30, 0.58]	0.40 [0.34, 0.48]	0.57 [0.50, 0.68]	0.12 [0.00, 0.30]	0.27 [0.00, 0.65]	0.88 [0.69, 1.00]	0.27 [0.21, 0.36]	0.52 [0.00, 0.90]
lr0.001_wd0.001_h256_d3	0.68 [0.62, 0.81]	0.43 [0.34, 0.65]	0.39 [0.34, 0.50]	0.57 [0.50, 0.73]	0.12 [0.00, 0.37]	0.31 [0.00, 0.78]	0.83 [0.38, 1.00]	0.27 [0.21, 0.34]	0.52 [0.00, 0.96]
lr0.001_wd0.001_h400_d3	0.68 [0.61, 0.75]	0.41 [0.32, 0.50]	0.31 [0.00, 0.47]	0.55 [0.50, 0.67]	0.08 [0.00, 0.29]	0.35 [0.00, 1.00]	0.74 [0.00, 1.00]	0.20 [0.00, 0.32]	0.51 [0.00, 0.92]
lr0.001_wd0.001_h512_d3	0.68 [0.60, 0.75]	0.39 [0.33, 0.54]	0.38 [0.33, 0.47]	0.58 [0.50, 0.66]	0.15 [0.00, 0.29]	0.55 [0.00, 0.85]	0.61 [0.31, 1.00]	0.31 [0.21, 0.41]	0.67 [0.00, 0.86]
lr0.001_wd0.001_h256_d4	0.69 [0.62, 0.79]	0.42 [0.29, 0.54]	0.38 [0.26, 0.47]	0.59 [0.50, 0.69]	0.18 [0.00, 0.36]	0.50 [0.00, 0.96]	0.67 [0.23, 1.00]	0.35 [0.22, 0.67]	0.70 [0.00, 0.95]
lr0.001_wd0.001_h400_d4	0.68 [0.59, 0.80]	0.40 [0.30, 0.65]	0.34 [0.30, 0.46]	0.55 [0.46, 0.66]	0.11 [-0.07, 0.31]	0.46 [0.00, 0.93]	0.64 [0.23, 1.00]	0.30 [0.19, 0.50]	0.67 [0.00, 1.00]
lr0.001_wd0.001_h512_d4	0.71 [0.63, 0.87]	0.38 [0.30, 0.64]	0.35 [0.00, 0.65]	0.58 [0.50, 0.82]	0.14 [0.00, 0.55]	0.43 [0.00, 1.00]	0.74 [0.00, 1.00]	0.24 [0.00, 0.53]	0.52 [0.00, 0.95]

Supplementary Table 29: Grid search results on the classification task on SYSMH external validation dataset (choosing 40% as the threshold for clinical variables selection). Reported metrics are AUC, AUPRC, F1, Balanced Accuracy, MCC, Specificity, Sensitivity, PPV, and NPV.

Config	AUC	AUPRC	F1	Balanced_Accuracy	MCC	Specificity	Sensitivity	PPV	NPV
lr0.0001_wd0.0_h256_d1	0.72[0.62, 0.83]	0.34[0.17, 0.51]	0.23[0.17, 0.30]	0.59[0.51, 0.67]	0.12[0.01, 0.24]	0.44[0.25, 0.66]	0.73[0.50, 1.00]	0.14[0.10, 0.17]	0.94[0.89, 1.00]
lr0.0001_wd0.0_h400_d1	0.74[0.60, 0.84]	0.36[0.15, 0.60]	0.27[0.20, 0.36]	0.62[0.52, 0.72]	0.17[0.07, 0.30]	0.40[0.03, 0.83]	0.83[0.50, 1.00]	0.17[0.12, 0.29]	0.97[0.92, 1.00]
lr0.0001_wd0.0_h512_d1	0.72[0.62, 0.78]	0.35[0.19, 0.61]	0.25[0.00, 0.36]	0.63[0.48, 0.74]	0.18[-0.06, 0.32]	0.66[0.21, 0.96]	0.60[0.00, 1.00]	0.17[0.00, 0.29]	0.95[0.90, 1.00]
lr0.0001_wd0.0_h256_d2	0.69[0.58, 0.79]	0.37[0.16, 0.52]	0.21[0.12, 0.29]	0.53[0.45, 0.61]	0.04[-0.06, 0.16]	0.36[0.00, 0.72]	0.70[0.33, 1.00]	0.13[0.08, 0.20]	0.55[0.00, 0.93]
lr0.0001_wd0.0_h400_d2	0.74[0.68, 0.79]	0.43[0.20, 0.52]	0.19[0.00, 0.27]	0.55[0.50, 0.61]	0.09[0.00, 0.16]	0.41[0.00, 1.00]	0.70[0.00, 1.00]	0.11[0.00, 0.18]	0.76[0.00, 1.00]
lr0.0001_wd0.0_h512_d2	0.73[0.60, 0.86]	0.32[0.21, 0.41]	0.21[0.00, 0.40]	0.55[0.50, 0.68]	0.10[0.00, 0.31]	0.41[0.00, 1.00]	0.70[0.00, 1.00]	0.14[0.00, 0.33]	0.77[0.00, 1.00]
lr0.0001_wd0.0001_h256_d1	0.73[0.63, 0.84]	0.34[0.16, 0.60]	0.26[0.21, 0.36]	0.61[0.53, 0.66]	0.17[0.09, 0.26]	0.39[0.07, 0.83]	0.83[0.50, 1.00]	0.17[0.12, 0.29]	0.97[0.92, 1.00]
lr0.0001_wd0.0001_h400_d1	0.72[0.65, 0.77]	0.32[0.18, 0.43]	0.23[0.17, 0.31]	0.56[0.50, 0.64]	0.09[0.00, 0.19]	0.29[0.00, 0.76]	0.83[0.50, 1.00]	0.14[0.10, 0.22]	0.77[0.00, 1.00]
lr0.0001_wd0.0001_h512_d1	0.74[0.63, 0.88]	0.37[0.16, 0.56]	0.23[0.22, 0.27]	0.57[0.50, 0.62]	0.11[0.00, 0.18]	0.24[0.00, 0.69]	0.90[0.50, 1.00]	0.14[0.12, 0.18]	0.78[0.00, 1.00]
lr0.0001_wd0.0001_h256_d2	0.72[0.64, 0.78]	0.36[0.16, 0.61]	0.26[0.00, 0.44]	0.63[0.50, 0.70]	0.21[0.00, 0.36]	0.66[0.28, 1.00]	0.60[0.00, 1.00]	0.20[0.00, 0.40]	0.95[0.90, 1.00]
lr0.0001_wd0.0001_h400_d2	0.73[0.68, 0.78]	0.39[0.17, 0.61]	0.23[0.00, 0.44]	0.58[0.50, 0.70]	0.13[0.00, 0.36]	0.56[0.00, 1.00]	0.60[0.00, 1.00]	0.16[0.00, 0.40]	0.75[0.00, 1.00]
lr0.0001_wd0.0001_h512_d2	0.75[0.60, 0.91]	0.34[0.16, 0.56]	0.24[0.17, 0.40]	0.55[0.49, 0.68]	0.09[-0.01, 0.31]	0.27[0.00, 0.86]	0.83[0.50, 1.00]	0.16[0.10, 0.33]	0.57[0.00, 1.00]
lr0.0001_wd0.001_h256_d1	0.73[0.63, 0.85]	0.31[0.22, 0.40]	0.18[0.00, 0.29]	0.54[0.50, 0.61]	0.07[0.00, 0.16]	0.44[0.07, 1.00]	0.63[0.00, 1.00]	0.11[0.00, 0.20]	0.95[0.90, 1.00]
lr0.0001_wd0.001_h400_d1	0.73[0.63, 0.84]	0.42[0.18, 0.61]	0.26[0.17, 0.40]	0.59[0.50, 0.68]	0.14[0.00, 0.31]	0.40[0.00, 0.86]	0.78[0.50, 1.00]	0.17[0.10, 0.33]	0.76[0.00, 1.00]
lr0.0001_wd0.001_h512_d1	0.73[0.60, 0.87]	0.41[0.17, 0.61]	0.24[0.15, 0.29]	0.57[0.44, 0.64]	0.10[-0.08, 0.21]	0.54[0.21, 0.75]	0.60[0.33, 1.00]	0.15[0.08, 0.20]	0.92[0.86, 1.00]
lr0.0001_wd0.001_h256_d2	0.74[0.62, 0.89]	0.35[0.15, 0.55]	0.21[0.00, 0.40]	0.56[0.50, 0.68]	0.09[0.00, 0.31]	0.42[0.00, 1.00]	0.70[0.00, 1.00]	0.14[0.00, 0.33]	0.57[0.00, 1.00]
lr0.0001_wd0.001_h400_d2	0.72[0.61, 0.84]	0.36[0.17, 0.56]	0.21[0.00, 0.40]	0.56[0.50, 0.68]	0.09[0.00, 0.31]	0.42[0.00, 1.00]	0.70[0.00, 1.00]	0.14[0.00, 0.33]	0.57[0.00, 1.00]
lr0.0001_wd0.001_h512_d2	0.70[0.60, 0.78]	0.37[0.17, 0.53]	0.24[0.00, 0.44]	0.59[0.50, 0.70]	0.18[0.00, 0.36]	0.63[0.11, 1.00]	0.55[0.00, 1.00]	0.23[0.00, 0.50]	0.95[0.90, 1.00]
lr0.0003_wd0.0_h256_d1	0.72[0.61, 0.78]	0.34[0.15, 0.51]	0.26[0.20, 0.33]	0.61[0.57, 0.65]	0.17[0.13, 0.22]	0.42[0.14, 0.79]	0.80[0.50, 1.00]	0.16[0.11, 0.25]	0.97[0.91, 1.00]
lr0.0003_wd0.0_h400_d1	0.74[0.62, 0.79]	0.36[0.19, 0.61]	0.27[0.18, 0.40]	0.61[0.53, 0.68]	0.18[0.04, 0.31]	0.49[0.21, 0.86]	0.73[0.50, 1.00]	0.19[0.11, 0.33]	0.95[0.92, 1.00]
lr0.0003_wd0.0_h512_d1	0.74[0.63, 0.80]	0.37[0.18, 0.61]	0.31[0.18, 0.50]	0.63[0.53, 0.72]	0.22[0.04, 0.43]	0.63[0.25, 0.93]	0.63[0.50, 1.00]	0.25[0.11, 0.50]	0.94[0.91, 1.00]
lr0.0003_wd0.0_h256_d2	0.72[0.57, 0.78]	0.35[0.16, 0.61]	0.24[0.17, 0.38]	0.56[0.49, 0.72]	0.10[-0.01, 0.30]	0.24[0.00, 0.69]	0.88[0.67, 1.00]	0.14[0.10, 0.25]	0.77[0.00, 1.00]
lr0.0003_wd0.0_h400_d2	0.70[0.60, 0.76]	0.27[0.15, 0.39]	0.27[0.22, 0.36]	0.61[0.50, 0.66]	0.16[0.00, 0.26]	0.42[0.00, 0.83]	0.80[0.50, 1.00]	0.17[0.12, 0.29]	0.77[0.00, 1.00]
lr0.0003_wd0.0_h512_d2	0.72[0.62, 0.78]	0.32[0.18, 0.39]	0.24[0.18, 0.29]	0.57[0.52, 0.59]	0.13[0.06, 0.21]	0.50[0.04, 0.93]	0.65[0.25, 1.00]	0.18[0.10, 0.33]	0.94[0.90, 1.00]
lr0.0003_wd0.0001_h256_d1	0.74[0.69, 0.77]	0.37[0.23, 0.60]	0.28[0.22, 0.40]	0.62[0.59, 0.68]	0.19[0.14, 0.31]	0.52[0.17, 0.86]	0.73[0.50, 1.00]	0.19[0.12, 0.33]	0.96[0.91, 1.00]
lr0.0003_wd0.0001_h400_d1	0.74[0.67, 0.81]	0.31[0.19, 0.52]	0.28[0.20, 0.44]	0.62[0.54, 0.70]	0.19[0.06, 0.36]	0.54[0.14, 0.90]	0.70[0.50, 1.00]	0.20[0.11, 0.40]	0.95[0.89, 1.00]
lr0.0003_wd0.0001_h512_d1	0.76[0.64, 0.79]	0.33[0.21, 0.46]	0.26[0.21, 0.35]	0.62[0.55, 0.70]	0.18[0.12, 0.27]	0.40[0.10, 0.69]	0.85[0.50, 1.00]	0.16[0.12, 0.23]	0.97[0.91, 1.00]
lr0.0003_wd0.0001_h256_d2	0.71[0.61, 0.80]	0.37[0.17, 0.73]	0.28[0.15, 0.55]	0.59[0.44, 0.81]	0.14[-0.08, 0.49]	0.47[0.00, 0.86]	0.72[0.50, 1.00]	0.19[0.08, 0.43]	0.73[0.00, 0.96]
lr0.0003_wd0.0001_h400_d2	0.70[0.58, 0.78]	0.31[0.17, 0.51]	0.26[0.18, 0.40]	0.59[0.50, 0.68]	0.14[0.00, 0.31]	0.40[0.00, 0.86]	0.78[0.50, 1.00]	0.17[0.11, 0.33]	0.75[0.00, 1.00]
lr0.0003_wd0.0001_h512_d2	0.73[0.61, 0.79]	0.38[0.22, 0.55]	0.25[0.18, 0.33]	0.57[0.50, 0.61]	0.13[0.00, 0.29]	0.53[0.00, 0.97]	0.62[0.25, 1.00]	0.21[0.12, 0.50]	0.75[0.00, 1.00]
lr0.0003_wd0.001_h256_d1	0.74[0.62, 0.83]	0.35[0.17, 0.53]	0.32[0.17, 0.46]	0.64[0.51, 0.77]	0.22[0.01, 0.40]	0.60[0.21, 0.86]	0.68[0.50, 1.00]	0.23[0.10, 0.33]	0.94[0.91, 1.00]
lr0.0003_wd0.001_h400_d1	0.74[0.68, 0.77]	0.37[0.22, 0.52]	0.27[0.21, 0.33]	0.62[0.59, 0.67]	0.20[0.14, 0.29]	0.45[0.17, 0.97]	0.78[0.25, 1.00]	0.22[0.12, 0.50]	0.97[0.90, 1.00]
lr0.0003_wd0.001_h512_d1	0.73[0.63, 0.79]	0.32[0.18, 0.52]	0.31[0.23, 0.40]	0.65[0.61, 0.68]	0.25[0.19, 0.31]	0.65[0.29, 0.97]	0.65[0.25, 1.00]	0.27[0.13, 0.50]	0.95[0.90, 1.00]
lr0.0003_wd0.001_h256_d2	0.73[0.58, 0.79]	0.37[0.20, 0.61]	0.25[0.18, 0.40]	0.57[0.50, 0.68]	0.11[0.00, 0.31]	0.37[0.00, 0.86]	0.77[0.33, 1.00]	0.17[0.12, 0.33]	0.57[0.00, 1.00]
lr0.0003_wd0.001_h400_d2	0.73[0.60, 0.81]	0.35[0.20, 0.52]	0.21[0.20, 0.24]	0.54[0.50, 0.57]	0.07[0.00, 0.13]	0.32[0.00, 0.79]	0.77[0.33, 1.00]	0.13[0.11, 0.15]	0.76[0.00, 1.00]
lr0.0003_wd0.001_h512_d2	0.71[0.63, 0.77]	0.31[0.18, 0.44]	0.25[0.17, 0.44]	0.56[0.50, 0.70]	0.12[0.00, 0.36]	0.29[0.00, 0.90]	0.83[0.50, 1.00]	0.17[0.10, 0.40]	0.77[0.00, 1.00]
lr0.001_wd0.0_h256_d1	0.74[0.64, 0.80]	0.33[0.21, 0.49]	0.29[0.21, 0.40]	0.65[0.59, 0.71]	0.23[0.14, 0.31]	0.40[0.18, 0.86]	0.90[0.50, 1.00]	0.19[0.12, 0.33]	0.99[0.93, 1.00]
lr0.001_wd0.0_h400_d1	0.76[0.65, 0.86]	0.38[0.21, 0.52]	0.32[0.21, 0.50]	0.65[0.55, 0.72]	0.24[0.10, 0.43]	0.57[0.10, 0.93]	0.73[0.50, 1.00]	0.25[0.12, 0.50]	0.96[0.93, 1.00]
lr0.001_wd0.0_h512_d1	0.73[0.61, 0.78]	0.36[0.16, 0.53]	0.25[0.17, 0.31]	0.59[0.51, 0.67]	0.15[0.01, 0.24]	0.50[0.11, 0.93]	0.68[0.25, 1.00]	0.19[0.10, 0.33]	0.95[0.90, 1.00]
lr0.001_wd0.0_h256_d2	0.70[0.64, 0.77]	0.32[0.16, 0.56]	0.28[0.20, 0.40]	0.61[0.56, 0.68]	0.19[0.08, 0.31]	0.70[0.25, 0.97]	0.52[0.25, 1.00]	0.25[0.12, 0.50]	0.93[0.90, 1.00]
lr0.001_wd0.0_h400_d2	0.75[0.65, 0.89]	0.32[0.18, 0.45]	0.30[0.00, 0.46]	0.66[0.43, 0.78]	0.24[-0.13, 0.40]	0.68[0.29, 0.90]	0.65[0.00, 1.00]	0.22[0.00, 0.40]	0.96[0.89, 1.00]
lr0.001_wd0.0_h512_d2	0.73[0.62, 0.81]	0.35[0.20, 0.56]	0.29[0.18, 0.44]	0.61[0.53, 0.70]	0.18[0.04, 0.36]	0.67[0.39, 0.90]	0.55[0.33, 0.75]	0.22[0.11, 0.40]	0.92[0.91, 0.93]
lr0.001_wd0.0001_h256_d1	0.76[0.65, 0.84]	0.37[0.18, 0.56]	0.31[0.20, 0.44]	0.65[0.57, 0.72]	0.23[0.08, 0.36]	0.57[0.21, 0.90]	0.73[0.50, 1.00]	0.22[0.12, 0.40]	0.96[0.92, 1.00]
lr0.001_wd0.0001_h400_d1	0.75[0.63, 0.81]	0.37[0.27, 0.44]	0.31[0.15, 0.40]	0.63[0.46, 0.72]	0.20[-0.06, 0.31]	0.58[0.21, 0.86]	0.68[0.50, 1.00]	0.22[0.09, 0.33]	0.94[0.88, 1.00]
lr0.001_wd0.0001_h512_d1	0.75[0.62, 0.84]	0.34[0.16, 0.52]	0.31[0.21, 0.42]	0.67[0.58, 0.81]	0.25[0.12, 0.41]	0.57[0.21, 0.86]	0.77[0.33, 1.00]	0.21[0.12, 0.33]	0.97[0.92, 1.00]
lr0.001_wd0.0001_h256_d2	0.73[0.60, 0.84]	0.34[0.16, 0.66]	0.24[0.19, 0.31]	0.58[0.50, 0.65]	0.11[0.00, 0.19]	0.47[0.00, 0.86]	0.68[0.25, 1.00]	0.16[0.10, 0.22]	0.75[0.00, 1.00]
lr0.001_wd0.0001_h400_d2	0.71[0.63, 0.77]	0.34[0.16, 0.61]	0.28[0.00, 0.50]	0.60[0.41, 0.72]	0.18[-0.14, 0.43]	0.70[0.21, 0.93]	0.50[0.00, 1.00]	0.23[0.00, 0.50]	0.93[0.88, 1.00]
lr0.001_wd0.0001_h512_d2	0.73[0.63, 0.86]	0.45[0.26, 0.54]	0.25[0.17, 0.36]	0.57[0.50, 0.66]	0.10[0.00, 0.26]	0.48[0.00, 0.83]	0.67[0.50, 1.00]	0.16[0.10, 0.29]	0.74[0.00, 0.94]
lr0.001_wd0.001_h256_d1	0.73[0.61, 0.78]	0.38[0.18, 0.60]	0.31[0.17, 0.42]	0.65[0.49, 0.81]	0.23[-0.01, 0.41]	0.57[0.21, 0.86]	0.73[0.50, 1.00]	0.22[0.10, 0.33]	0.95[0.90, 1.00]
lr0.001_wd0.001_h400_d1	0.74[0.63, 0.80]	0.29[0.16, 0.41]	0.33[0.22, 0.46]	0.67[0.62, 0.77]	0.26[0.18, 0.40]	0.60[0.25, 0.86]	0.75[0.50, 1.00]	0.24[0.12, 0.33]	0.96[0.92, 1.00]
lr0.001_wd0.001_h512_d1	0.76[0.74, 0.79]	0.38[0.25, 0.61]	0.33[0.21, 0.40]	0.67[0.60, 0.74]	0.25[0.13, 0.35]	0.61[0.21, 0.83]	0.73[0.50, 1.00]	0.22[0.12, 0.29]	0.95[0.92, 1.00]
lr0.001_wd0.001_h256_d2	0.74[0.62, 0.86]	0.30[0.16, 0.45]	0.24[0.19, 0.31]	0.56[0.50, 0.64]	0.09[0.00, 0.19]	0.29[0.00, 0.76]	0.83[0.50, 1.00]	0.15[0.11, 0.22]	0.57[0.00, 1.00]
lr0.001_wd0.001_h400_d2	0.70[0.61, 0.78]	0.27[0.15, 0.40]	0.27[0.19, 0.38]	0.63[0.55, 0.72]	0.18[0.06, 0.30]	0.47[0.25, 0.69]	0.78[0.50, 1.00]	0.17[0.11, 0.25]	0.96[0.91, 1.00]
lr0.001_wd0.001_h512_d2	0.74[0.60, 0.84]	0.35[0.17, 0.65]	0.33[0.17, 0.57]	0.63[0.49, 0.73]	0.24[-0.01, 0.53]	0.54[0.24, 0.97]	0.73[0.50, 1.00]	0.28[0.10, 0.67]	0.95[0.90, 1.00]

Supplementary Table 30: Grid search results on the classification task on GDHCM retrospective dataset (choosing 60% as the threshold for clinical variables selection). Reported metrics are AUC, AUPRC, F1, Balanced Accuracy, MCC, Specificity, Sensitivity, PPV, and NPV.

Config	AUC	AUPRC	F1	Balanced_Accuracy	MCC	Specificity	Sensitivity	PPV	NPV
lr1e-05_wd0.005_h1024_d3	0.71 [0.62, 0.79]	0.34 [0.26, 0.44]	0.14 [0.00, 0.27]	0.52 [0.50, 0.59]	0.03 [0.00, 0.13]	0.54 [0.00, 1.00]	0.50 [0.00, 1.00]	0.08 [0.00, 0.18]	0.54 [0.00, 0.91]
lr1e-05_wd0.005_h2048_d3	0.71 [0.62, 0.78]	0.29 [0.15, 0.53]	0.16 [0.00, 0.36]	0.53 [0.50, 0.66]	0.05 [0.00, 0.26]	0.57 [0.00, 1.00]	0.50 [0.00, 1.00]	0.11 [0.00, 0.29]	0.55 [0.00, 0.92]
lr1e-05_wd0.005_h1024_d4	0.74 [0.57, 0.86]	0.34 [0.15, 0.52]	0.18 [0.00, 0.31]	0.53 [0.50, 0.63]	0.04 [0.00, 0.19]	0.42 [0.00, 1.00]	0.63 [0.00, 1.00]	0.11 [0.00, 0.22]	0.55 [0.00, 0.92]
lr1e-05_wd0.005_h2048_d4	0.70 [0.62, 0.79]	0.36 [0.15, 0.66]	0.20 [0.00, 0.33]	0.55 [0.50, 0.65]	0.08 [0.00, 0.22]	0.40 [0.00, 1.00]	0.70 [0.00, 1.00]	0.12 [0.00, 0.25]	0.56 [0.00, 1.00]
lr1e-05_wd0.01_h1024_d3	0.72 [0.58, 0.79]	0.31 [0.15, 0.42]	0.16 [0.00, 0.35]	0.55 [0.50, 0.74]	0.06 [0.00, 0.32]	0.50 [0.00, 1.00]	0.60 [0.00, 1.00]	0.09 [0.00, 0.21]	0.56 [0.00, 1.00]
lr1e-05_wd0.01_h2048_d3	0.76 [0.64, 0.84]	0.33 [0.21, 0.44]	0.14 [0.00, 0.29]	0.52 [0.50, 0.61]	0.03 [0.00, 0.16]	0.54 [0.00, 1.00]	0.50 [0.00, 1.00]	0.09 [0.00, 0.20]	0.54 [0.00, 0.91]
lr1e-05_wd0.01_h1024_d4	0.74 [0.60, 0.82]	0.38 [0.15, 0.73]	0.14 [0.00, 0.29]	0.51 [0.43, 0.61]	0.01 [-0.13, 0.16]	0.52 [0.00, 1.00]	0.50 [0.00, 1.00]	0.09 [0.00, 0.20]	0.54 [0.00, 0.91]
lr1e-05_wd0.01_h2048_d4	0.72 [0.60, 0.82]	0.36 [0.15, 0.53]	0.22 [0.00, 0.44]	0.56 [0.50, 0.70]	0.10 [0.00, 0.36]	0.42 [0.00, 1.00]	0.70 [0.00, 1.00]	0.15 [0.00, 0.40]	0.57 [0.00, 1.00]
lr3e-05_wd0.005_h1024_d3	0.72 [0.65, 0.82]	0.43 [0.17, 0.63]	0.21 [0.00, 0.40]	0.55 [0.50, 0.68]	0.09 [0.00, 0.31]	0.41 [0.00, 1.00]	0.70 [0.00, 1.00]	0.14 [0.00, 0.33]	0.57 [0.00, 1.00]
lr3e-05_wd0.005_h2048_d3	0.73 [0.63, 0.78]	0.33 [0.18, 0.41]	0.21 [0.00, 0.40]	0.55 [0.50, 0.68]	0.10 [0.00, 0.31]	0.40 [0.00, 1.00]	0.70 [0.00, 1.00]	0.14 [0.00, 0.33]	0.77 [0.00, 1.00]
lr3e-05_wd0.005_h1024_d4	0.76 [0.68, 0.85]	0.33 [0.18, 0.46]	0.20 [0.00, 0.36]	0.55 [0.50, 0.66]	0.08 [0.00, 0.26]	0.41 [0.00, 1.00]	0.70 [0.00, 1.00]	0.13 [0.00, 0.29]	0.57 [0.00, 1.00]
lr3e-05_wd0.005_h2048_d4	0.75 [0.64, 0.79]	0.40 [0.16, 0.57]	0.17 [0.00, 0.44]	0.55 [0.50, 0.70]	0.09 [0.00, 0.36]	0.60 [0.00, 1.00]	0.50 [0.00, 1.00]	0.13 [0.00, 0.40]	0.74 [0.00, 1.00]
lr3e-05_wd0.01_h1024_d3	0.72 [0.62, 0.81]	0.33 [0.16, 0.51]	0.20 [0.00, 0.36]	0.55 [0.50, 0.66]	0.08 [0.00, 0.26]	0.41 [0.00, 1.00]	0.70 [0.00, 1.00]	0.13 [0.00, 0.29]	0.57 [0.00, 1.00]
lr3e-05_wd0.01_h2048_d3	0.71 [0.63, 0.78]	0.39 [0.18, 0.64]	0.19 [0.00, 0.33]	0.54 [0.50, 0.65]	0.07 [0.00, 0.22]	0.37 [0.00, 1.00]	0.70 [0.00, 1.00]	0.12 [0.00, 0.25]	0.76 [0.00, 1.00]
lr3e-05_wd0.01_h1024_d4	0.71 [0.60, 0.79]	0.30 [0.16, 0.39]	0.21 [0.00, 0.40]	0.55 [0.50, 0.68]	0.08 [0.00, 0.31]	0.39 [0.00, 1.00]	0.70 [0.00, 1.00]	0.14 [0.00, 0.33]	0.57 [0.00, 1.00]
lr3e-05_wd0.01_h2048_d4	0.76 [0.69, 0.81]	0.38 [0.20, 0.63]	0.21 [0.00, 0.36]	0.57 [0.50, 0.68]	0.10 [0.00, 0.26]	0.44 [0.00, 1.00]	0.70 [0.00, 1.00]	0.13 [0.00, 0.29]	0.57 [0.00, 1.00]
lr1e-05_wd0.0_h256_d1	0.69 [0.60, 0.80]	0.44 [0.35, 0.53]	0.21 [0.00, 0.40]	0.55 [0.50, 0.62]	0.14 [0.00, 0.48]	0.45 [0.00, 1.00]	0.65 [0.00, 1.00]	0.27 [0.00, 1.00]	0.76 [0.00, 1.00]
lr1e-05_wd0.0_h400_d1	0.71 [0.58, 0.85]	0.30 [0.16, 0.57]	0.15 [0.00, 0.31]	0.55 [0.50, 0.69]	0.07 [0.00, 0.26]	0.56 [0.00, 1.00]	0.55 [0.00, 1.00]	0.09 [0.00, 0.18]	0.75 [0.00, 1.00]
lr1e-05_wd0.0_h512_d1	0.67 [0.56, 0.84]	0.38 [0.15, 0.65]	0.15 [0.00, 0.26]	0.52 [0.41, 0.60]	0.03 [-0.14, 0.18]	0.50 [0.00, 1.00]	0.55 [0.00, 1.00]	0.09 [0.00, 0.16]	0.74 [0.00, 1.00]
lr1e-05_wd0.0_h256_d2	0.71 [0.60, 0.85]	0.33 [0.18, 0.61]	0.19 [0.00, 0.40]	0.57 [0.41, 0.74]	0.09 [-0.14, 0.33]	0.59 [0.00, 1.00]	0.55 [0.00, 1.00]	0.12 [0.00, 0.27]	0.75 [0.00, 1.00]
lr1e-05_wd0.0_h400_d2	0.78 [0.61, 0.94]	0.39 [0.15, 0.56]	0.21 [0.00, 0.40]	0.54 [0.50, 0.65]	0.10 [0.00, 0.36]	0.41 [0.00, 1.00]	0.67 [0.00, 1.00]	0.18 [0.00, 0.50]	0.57 [0.00, 1.00]
lr1e-05_wd0.0_h512_d2	0.72 [0.67, 0.77]	0.34 [0.23, 0.61]	0.17 [0.00, 0.22]	0.51 [0.50, 0.56]	0.02 [0.00, 0.10]	0.37 [0.00, 1.00]	0.65 [0.00, 1.00]	0.11 [0.00, 0.20]	0.36 [0.00, 0.90]
lr1e-05_wd0.0001_h256_d1	0.70 [0.61, 0.78]	0.37 [0.26, 0.62]	0.11 [0.00, 0.22]	0.49 [0.47, 0.50]	-0.03 [-0.09, 0.00]	0.51 [0.00, 1.00]	0.47 [0.00, 1.00]	0.07 [0.00, 0.12]	0.53 [0.00, 0.90]
lr1e-05_wd0.0001_h400_d1	0.71 [0.60, 0.86]	0.38 [0.23, 0.70]	0.19 [0.00, 0.30]	0.56 [0.50, 0.67]	0.08 [0.00, 0.24]	0.31 [0.00, 1.00]	0.80 [0.00, 1.00]	0.11 [0.00, 0.17]	0.58 [0.00, 1.00]
lr1e-05_wd0.0001_h512_d1	0.71 [0.62, 0.83]	0.40 [0.25, 0.51]	0.24 [0.18, 0.29]	0.58 [0.50, 0.66]	0.13 [0.00, 0.23]	0.21 [0.00, 0.52]	0.95 [0.75, 1.00]	0.14 [0.10, 0.18]	0.79 [0.00, 1.00]
lr1e-05_wd0.0001_h256_d2	0.74 [0.64, 0.78]	0.28 [0.16, 0.41]	0.13 [0.00, 0.23]	0.50 [0.46, 0.53]	0.00 [-0.09, 0.09]	0.40 [0.00, 1.00]	0.60 [0.00, 1.00]	0.07 [0.00, 0.13]	0.56 [0.00, 1.00]
lr1e-05_wd0.0001_h400_d2	0.73 [0.65, 0.79]	0.40 [0.24, 0.61]	0.21 [0.00, 0.33]	0.56 [0.50, 0.67]	0.10 [0.00, 0.26]	0.45 [0.00, 1.00]	0.67 [0.00, 1.00]	0.15 [0.00, 0.33]	0.57 [0.00, 1.00]
lr1e-05_wd0.0001_h512_d2	0.72 [0.60, 0.90]	0.34 [0.16, 0.65]	0.13 [0.00, 0.24]	0.51 [0.50, 0.56]	0.02 [0.00, 0.09]	0.48 [0.00, 1.00]	0.55 [0.00, 1.00]	0.08 [0.00, 0.14]	0.54 [0.00, 0.92]
lr1e-05_wd0.001_h256_d1	0.67 [0.54, 0.78]	0.33 [0.20, 0.41]	0.20 [0.00, 0.27]	0.59 [0.50, 0.64]	0.15 [0.00, 0.19]	0.39 [0.21, 1.00]	0.80 [0.00, 1.00]	0.12 [0.00, 0.15]	0.98 [0.90, 1.00]
lr1e-05_wd0.001_h400_d1	0.74 [0.56, 0.93]	0.38 [0.21, 0.68]	0.17 [0.00, 0.43]	0.56 [0.50, 0.75]	0.09 [0.00, 0.36]	0.57 [0.00, 1.00]	0.55 [0.00, 1.00]	0.11 [0.00, 0.30]	0.75 [0.00, 1.00]
lr1e-05_wd0.001_h512_d1	0.71 [0.64, 0.81]	0.32 [0.17, 0.51]	0.17 [0.00, 0.22]	0.51 [0.50, 0.53]	0.02 [0.00, 0.07]	0.32 [0.00, 1.00]	0.70 [0.00, 1.00]	0.10 [0.00, 0.13]	0.56 [0.00, 1.00]
lr1e-05_wd0.001_h256_d2	0.73 [0.63, 0.81]	0.40 [0.22, 0.57]	0.13 [0.00, 0.22]	0.50 [0.50, 0.50]	0.00 [0.00, 0.00]	0.40 [0.00, 1.00]	0.60 [0.00, 1.00]	0.07 [0.00, 0.12]	0.36 [0.00, 0.90]
lr1e-05_wd0.001_h400_d2	0.75 [0.65, 0.87]	0.40 [0.20, 0.58]	0.09 [0.00, 0.22]	0.50 [0.50, 0.52]	0.01 [0.00, 0.07]	0.61 [0.00, 1.00]	0.40 [0.00, 1.00]	0.05 [0.00, 0.12]	0.74 [0.00, 1.00]
lr1e-05_wd0.001_h512_d2	0.67 [0.56, 0.79]	0.33 [0.14, 0.62]	0.15 [0.00, 0.31]	0.54 [0.50, 0.69]	0.05 [0.00, 0.26]	0.48 [0.00, 1.00]	0.60 [0.00, 1.00]	0.08 [0.00, 0.18]	0.56 [0.00, 1.00]
lr3e-05_wd0.0_h256_d1	0.70 [0.58, 0.81]	0.40 [0.20, 0.60]	0.17 [0.00, 0.22]	0.51 [0.50, 0.54]	0.02 [0.00, 0.07]	0.36 [0.00, 1.00]	0.67 [0.00, 1.00]	0.10 [0.00, 0.12]	0.56 [0.00, 1.00]
lr3e-05_wd0.0_h400_d1	0.71 [0.61, 0.77]	0.27 [0.15, 0.34]	0.19 [0.00, 0.30]	0.55 [0.48, 0.67]	0.06 [-0.06, 0.24]	0.39 [0.00, 0.96]	0.70 [0.00, 1.00]	0.11 [0.00, 0.17]	0.56 [0.00, 1.00]
lr3e-05_wd0.0_h512_d1	0.71 [0.61, 0.78]	0.30 [0.17, 0.45]	0.19 [0.00, 0.31]	0.55 [0.50, 0.69]	0.07 [0.00, 0.26]	0.47 [0.00, 1.00]	0.63 [0.00, 1.00]	0.11 [0.00, 0.18]	0.74 [0.00, 1.00]
lr3e-05_wd0.0_h256_d2	0.77 [0.69, 0.86]	0.35 [0.21, 0.63]	0.14 [0.00, 0.25]	0.51 [0.48, 0.58]	0.01 [-0.06, 0.11]	0.52 [0.00, 1.00]	0.50 [0.00, 1.00]	0.08 [0.00, 0.17]	0.54 [0.00, 0.90]
lr3e-05_wd0.0_h400_d2	0.69 [0.57, 0.82]	0.34 [0.15, 0.52]	0.22 [0.18, 0.29]	0.54 [0.50, 0.61]	0.05 [0.00, 0.16]	0.31 [0.00, 0.72]	0.77 [0.50, 1.00]	0.13 [0.11, 0.20]	0.55 [0.00, 0.92]
lr3e-05_wd0.0_h512_d2	0.73 [0.63, 0.81]	0.34 [0.16, 0.53]	0.19 [0.00, 0.31]	0.55 [0.50, 0.64]	0.08 [0.00, 0.19]	0.41 [0.00, 1.00]	0.70 [0.00, 1.00]	0.12 [0.00, 0.22]	0.56 [0.00, 1.00]
lr3e-05_wd0.0001_h256_d1	0.73 [0.58, 0.91]	0.37 [0.22, 0.59]	0.14 [0.00, 0.25]	0.52 [0.50, 0.58]	0.03 [0.00, 0.11]	0.54 [0.00, 1.00]	0.50 [0.00, 1.00]	0.08 [0.00, 0.17]	0.74 [0.00, 1.00]
lr3e-05_wd0.0001_h400_d1	0.77 [0.67, 0.85]	0.35 [0.24, 0.50]	0.21 [0.00, 0.44]	0.57 [0.50, 0.83]	0.09 [0.00, 0.43]	0.40 [0.00, 1.00]	0.73 [0.00, 1.00]	0.13 [0.00, 0.29]	0.56 [0.00, 1.00]
lr3e-05_wd0.0001_h512_d1	0.73 [0.58, 0.86]	0.34 [0.19, 0.54]	0.21 [0.17, 0.25]	0.52 [0.50, 0.58]	0.02 [0.00, 0.11]	0.20 [0.00, 0.66]	0.83 [0.50, 1.00]	0.12 [0.10, 0.17]	0.36 [0.00, 0.91]
lr3e-05_wd0.0001_h256_d2	0.70 [0.60, 0.84]	0.36 [0.17, 0.53]	0.19 [0.00, 0.29]	0.54 [0.50, 0.61]	0.06 [0.00, 0.16]	0.38 [0.00, 1.00]	0.70 [0.00, 1.00]	0.11 [0.00, 0.20]	0.56 [0.00, 1.00]
lr3e-05_wd0.0001_h400_d2	0.70 [0.60, 0.83]	0.31 [0.14, 0.41]	0.18 [0.00, 0.27]	0.54 [0.50, 0.62]	0.06 [0.00, 0.18]	0.39 [0.00, 1.00]	0.70 [0.00, 1.00]	0.11 [0.00, 0.18]	0.56 [0.00, 1.00]
lr3e-05_wd0.0001_h512_d2	0.71 [0.61, 0.78]	0.31 [0.16, 0.44]	0.21 [0.17, 0.22]	0.53 [0.50, 0.62]	0.04 [0.00, 0.18]	0.12 [0.00, 0.36]	0.93 [0.67, 1.00]	0.12 [0.10, 0.12]	0.38 [0.00, 1.00]
lr3e-05_wd0.001_h256_d1	0.73 [0.61, 0.80]	0.34 [0.16, 0.52]	0.15 [0.00, 0.30]	0.53 [0.45, 0.65]	0.05 [-0.11, 0.20]	0.52 [0.00, 1.00]	0.55 [0.00, 1.00]	0.09 [0.00, 0.19]	0.75 [0.00, 1.00]
lr3e-05_wd0.001_h400_d1	0.69 [0.57, 0.75]	0.42 [0.14, 0.59]	0.18 [0.00, 0.31]	0.54 [0.50, 0.69]	0.06 [0.00, 0.26]	0.35 [0.00, 1.00]	0.73 [0.00, 1.00]	0.10 [0.00, 0.18]	0.56 [0.00, 1.00]
lr3e-05_wd0.001_h512_d1	0.70 [0.62, 0.78]	0.34 [0.22, 0.60]	0.17 [0.00, 0.29]	0.51 [0.42, 0.61]	0.01 [-0.12, 0.16]	0.38 [0.00, 1.00]	0.63 [0.00, 1.00]	0.10 [0.00, 0.20]	0.53 [0.00, 0.91]
lr3e-05_wd0.001_h256_d2	0.70 [0.57, 0.81]	0.38 [0.16, 0.47]	0.18 [0.00, 0.27]	0.53 [0.50, 0.62]	0.05 [0.00, 0.19]	0.33 [0.00, 1.00]	0.73 [0.00, 1.00]	0.10 [0.00, 0.15]	0.56 [0.00, 1.00]
lr3e-05_wd0.001_h400_d2	0.78 [0.60, 0.94]	0.44 [0.21, 0.67]	0.19 [0.00, 0.31]	0.54 [0.50, 0.63]	0.06 [0.00, 0.19]	0.37 [0.00, 1.00]	0.70 [0.00, 1.00]	0.11 [0.00, 0.22]	0.56 [0.00, 1.00]
lr3e-05_wd0.001_h512_d2	0.71 [0.58, 0.85]	0.34 [0.16, 0.52]	0.19 [0.00, 0.33]	0.53 [0.49, 0.65]	0.04 [-0.01, 0.22]	0.42 [0.00, 1.00]	0.63 [0.00, 1.00]	0.12 [0.00, 0.25]	0.54 [0.00, 0.92]
lr0.0001_wd0.005_h256_d1	0.72 [0.62, 0.83]	0.34 [0.17, 0.51]	0.25 [0.17, 0.31]	0.60 [0.51, 0.67]	0.15 [0.01, 0.24]	0.47 [0.25, 0.76]	0.73 [0.50, 1.00]	0.16 [0.10, 0.22]	0.95 [0.90, 1.00]
lr0.0001_wd0.005_h400_d1	0.74 [0.60, 0.84]	0.36 [0.15, 0.60]	0.26 [0.20, 0.33]	0.61 [0.52, 0.72]	0.17 [0.07, 0.30]	0.39 [0.03, 0.79]	0.83 [0.50, 1.00]	0.16 [0.12, 0.25]	0.97 [0.92, 1.00]
lr0.0001_wd0.005_h512_d1	0.72 [0.62, 0.79]	0.36 [0.20, 0.61]	0.25 [0.00, 0.36]	0.63 [0.48, 0.74]	0.18 [-0.06, 0.32]	0.66 [0.21, 0.96]	0.60 [0.00, 1.00]	0.17 [0.00, 0.29]	0.95 [0.90, 1.00]
lr0.0001_wd0.005_h256_d2	0.69 [0.58, 0.79]	0.37 [0.16, 0.52]	0.22 [0.12, 0.31]	0.53 [0.45, 0.63]	0.05 [-0.06, 0.19]	0.37 [0.00, 0.76]	0.70 [0.33, 1.00]	0.13 [0.08, 0.22]	0.55 [0.00, 0.93]
lr0.0001_wd0.005_h400_d2	0.75 [0.68, 0.79]	0.43 [0.21, 0.61]	0.25 [0.00, 0.57]	0.58 [0.50, 0.73]	0.16 [0.00, 0.53]	0.57 [0.00, 1.00]	0.60 [0.00, 1.00]	0.22 [0.00, 0.67]	0.75 [0.00, 1.00]
lr0.0001_wd0.005_h512_d2	0.73 [0.60, 0.86]	0.33 [0.19, 0.41]	0.21 [0.00, 0.40]	0.56 [0.50, 0.68]	0.11 [0.00, 0.31]	0.42 [0.00, 1.00]	0.70 [0.00, 1.00]	0.14 [0.00, 0.33]	0.77 [0.00, 1.00]
lr0.0001_wd0.01_h256_d1	0.73 [0.65, 0.84]	0.34 [0.17, 0.60]	0.25 [0.21, 0.31]	0.60 [0.53, 0.65]	0.16 [0.09, 0.19]	0.37 [0.07, 0.76]	0.83 [0.50, 1.00]	0.16 [0.12, 0.22]	0.97 [0.92, 1.00]
lr0.0001_wd0.01_h400_d1	0.72 [0.65, 0.77]	0.32 [0.18, 0.43]	0.23 [0.17, 0.31]	0.56 [0.50, 0.64]	0.09 [0.00, 0.19]	0.29 [0.00, 0.76]	0.83 [0.50, 1.00]	0.14 [0.10, 0.22]	0.77 [0.00, 1.00]

Supplementary Table 31: Grid search results on the classification task on GDHCM retrospective dataset (choosing 60% as the threshold for clinical variables selection). Reported metrics are AUC, AUPRC, F1, Balanced Accuracy, MCC, Specificity, Sensitivity, PPV, and NPV.

Config	AUC	AUPRC	F1	Balanced_Accuracy	MCC	Specificity	Sensitivity	PPV	NPV
lr0.0001_wd0.01_h512_d1	0.74 [0.63, 0.88]	0.37 [0.16, 0.56]	0.23 [0.22, 0.27]	0.57 [0.50, 0.62]	0.11 [0.00, 0.18]	0.24 [0.00, 0.69]	0.90 [0.50, 1.00]	0.14 [0.12, 0.18]	0.78 [0.00, 1.00]
lr0.0001_wd0.01_h256_d2	0.72 [0.64, 0.78]	0.36 [0.16, 0.61]	0.26 [0.00, 0.44]	0.61 [0.50, 0.70]	0.20 [0.00, 0.36]	0.63 [0.14, 1.00]	0.60 [0.00, 1.00]	0.20 [0.00, 0.40]	0.95 [0.90, 1.00]
lr0.0001_wd0.01_h400_d2	0.73 [0.69, 0.78]	0.40 [0.18, 0.61]	0.23 [0.00, 0.40]	0.59 [0.50, 0.68]	0.14 [0.00, 0.31]	0.57 [0.00, 1.00]	0.60 [0.00, 1.00]	0.16 [0.00, 0.33]	0.75 [0.00, 1.00]
lr0.0001_wd0.01_h512_d2	0.74 [0.60, 0.91]	0.33 [0.16, 0.56]	0.24 [0.17, 0.40]	0.56 [0.49, 0.68]	0.09 [-0.01, 0.31]	0.28 [0.00, 0.86]	0.83 [0.50, 1.00]	0.16 [0.10, 0.33]	0.57 [0.00, 1.00]
lr0.0003_wd0.005_h256_d1	0.73 [0.62, 0.90]	0.30 [0.16, 0.46]	0.25 [0.15, 0.36]	0.57 [0.46, 0.66]	0.11 [-0.06, 0.26]	0.41 [0.00, 0.83]	0.73 [0.50, 1.00]	0.17 [0.09, 0.29]	0.74 [0.00, 1.00]
lr0.0003_wd0.005_h400_d1	0.74 [0.64, 0.87]	0.37 [0.19, 0.61]	0.29 [0.21, 0.40]	0.66 [0.58, 0.74]	0.21 [0.10, 0.33]	0.53 [0.25, 0.76]	0.78 [0.50, 1.00]	0.19 [0.12, 0.27]	0.96 [0.92, 1.00]
lr0.0003_wd0.005_h512_d1	0.74 [0.67, 0.79]	0.35 [0.18, 0.50]	0.26 [0.17, 0.36]	0.61 [0.49, 0.76]	0.15 [-0.01, 0.34]	0.41 [0.03, 0.59]	0.82 [0.67, 1.00]	0.16 [0.10, 0.22]	0.96 [0.90, 1.00]
lr0.0003_wd0.005_h256_d2	0.74 [0.61, 0.88]	0.29 [0.16, 0.40]	0.24 [0.00, 0.57]	0.58 [0.50, 0.73]	0.16 [0.00, 0.53]	0.54 [0.00, 1.00]	0.63 [0.00, 1.00]	0.21 [0.00, 0.67]	0.75 [0.00, 1.00]
lr0.0003_wd0.005_h400_d2	0.72 [0.55, 0.84]	0.40 [0.17, 0.58]	0.25 [0.17, 0.36]	0.59 [0.50, 0.66]	0.14 [0.00, 0.26]	0.41 [0.00, 0.83]	0.77 [0.33, 1.00]	0.16 [0.11, 0.29]	0.77 [0.00, 1.00]
lr0.0003_wd0.005_h512_d2	0.72 [0.57, 0.82]	0.32 [0.19, 0.41]	0.31 [0.19, 0.50]	0.62 [0.55, 0.72]	0.22 [0.08, 0.43]	0.55 [0.11, 0.93]	0.70 [0.50, 1.00]	0.24 [0.11, 0.50]	0.95 [0.90, 1.00]
lr0.0003_wd0.01_h256_d1	0.72 [0.62, 0.78]	0.34 [0.15, 0.51]	0.27 [0.21, 0.36]	0.63 [0.59, 0.68]	0.19 [0.13, 0.26]	0.45 [0.17, 0.83]	0.80 [0.50, 1.00]	0.17 [0.12, 0.29]	0.97 [0.91, 1.00]
lr0.0003_wd0.01_h400_d1	0.74 [0.63, 0.80]	0.36 [0.20, 0.61]	0.27 [0.18, 0.40]	0.61 [0.53, 0.68]	0.17 [0.04, 0.31]	0.49 [0.18, 0.86]	0.73 [0.50, 1.00]	0.19 [0.11, 0.33]	0.95 [0.92, 1.00]
lr0.0003_wd0.01_h512_d1	0.75 [0.63, 0.82]	0.37 [0.18, 0.61]	0.34 [0.19, 0.57]	0.64 [0.55, 0.73]	0.25 [0.06, 0.53]	0.65 [0.21, 0.97]	0.63 [0.50, 1.00]	0.29 [0.11, 0.67]	0.94 [0.92, 1.00]
lr0.0003_wd0.01_h256_d2	0.71 [0.57, 0.78]	0.33 [0.15, 0.61]	0.25 [0.17, 0.44]	0.56 [0.49, 0.70]	0.12 [-0.01, 0.36]	0.29 [0.00, 0.90]	0.83 [0.50, 1.00]	0.17 [0.10, 0.40]	0.77 [0.00, 1.00]
lr0.0003_wd0.01_h400_d2	0.71 [0.61, 0.77]	0.33 [0.15, 0.52]	0.34 [0.21, 0.44]	0.65 [0.59, 0.70]	0.30 [0.14, 0.48]	0.65 [0.18, 1.00]	0.65 [0.25, 1.00]	0.40 [0.12, 1.00]	0.95 [0.91, 1.00]
lr0.0003_wd0.01_h512_d2	0.73 [0.62, 0.78]	0.32 [0.19, 0.44]	0.26 [0.18, 0.33]	0.60 [0.52, 0.67]	0.17 [0.06, 0.29]	0.44 [0.04, 0.97]	0.75 [0.25, 1.00]	0.21 [0.10, 0.50]	0.96 [0.90, 1.00]
lr0.001_wd0.005_h256_d1	0.76 [0.69, 0.88]	0.37 [0.20, 0.60]	0.28 [0.22, 0.44]	0.63 [0.57, 0.70]	0.21 [0.13, 0.36]	0.42 [0.14, 0.90]	0.85 [0.50, 1.00]	0.19 [0.12, 0.40]	0.97 [0.93, 1.00]
lr0.001_wd0.005_h400_d1	0.75 [0.68, 0.84]	0.37 [0.22, 0.52]	0.31 [0.19, 0.50]	0.62 [0.54, 0.72]	0.22 [0.08, 0.43]	0.51 [0.07, 0.93]	0.73 [0.50, 1.00]	0.24 [0.10, 0.50]	0.96 [0.93, 1.00]
lr0.001_wd0.005_h512_d1	0.74 [0.67, 0.78]	0.33 [0.22, 0.49]	0.31 [0.22, 0.44]	0.66 [0.62, 0.70]	0.24 [0.18, 0.36]	0.51 [0.25, 0.90]	0.80 [0.50, 1.00]	0.22 [0.12, 0.40]	0.97 [0.92, 1.00]
lr0.001_wd0.005_h256_d2	0.71 [0.63, 0.78]	0.33 [0.17, 0.47]	0.28 [0.21, 0.40]	0.63 [0.59, 0.68]	0.20 [0.14, 0.31]	0.45 [0.18, 0.86]	0.80 [0.50, 1.00]	0.19 [0.12, 0.33]	0.97 [0.91, 1.00]
lr0.001_wd0.005_h400_d2	0.74 [0.61, 0.80]	0.34 [0.16, 0.53]	0.35 [0.17, 0.57]	0.64 [0.49, 0.73]	0.26 [-0.01, 0.53]	0.60 [0.29, 0.97]	0.68 [0.50, 1.00]	0.31 [0.10, 0.67]	0.94 [0.90, 1.00]
lr0.001_wd0.005_h512_d2	0.74 [0.68, 0.84]	0.25 [0.19, 0.38]	0.29 [0.22, 0.36]	0.65 [0.52, 0.73]	0.22 [0.07, 0.31]	0.37 [0.03, 0.79]	0.93 [0.67, 1.00]	0.17 [0.12, 0.25]	0.99 [0.96, 1.00]
lr0.001_wd0.01_h256_d1	0.71 [0.61, 0.78]	0.33 [0.16, 0.49]	0.25 [0.14, 0.40]	0.59 [0.49, 0.68]	0.13 [-0.01, 0.31]	0.56 [0.21, 0.86]	0.62 [0.33, 1.00]	0.17 [0.09, 0.33]	0.93 [0.90, 1.00]
lr0.001_wd0.01_h400_d1	0.73 [0.67, 0.79]	0.30 [0.19, 0.39]	0.32 [0.20, 0.44]	0.65 [0.57, 0.70]	0.24 [0.13, 0.36]	0.54 [0.14, 0.90]	0.75 [0.50, 1.00]	0.25 [0.11, 0.40]	0.96 [0.93, 1.00]
lr0.001_wd0.01_h512_d1	0.74 [0.70, 0.78]	0.34 [0.21, 0.52]	0.31 [0.21, 0.44]	0.64 [0.58, 0.70]	0.21 [0.10, 0.36]	0.64 [0.21, 0.90]	0.63 [0.50, 1.00]	0.23 [0.12, 0.40]	0.94 [0.92, 1.00]
lr0.001_wd0.01_h256_d2	0.72 [0.62, 0.77]	0.32 [0.21, 0.40]	0.32 [0.19, 0.40]	0.66 [0.54, 0.71]	0.24 [0.09, 0.31]	0.61 [0.07, 0.86]	0.70 [0.50, 1.00]	0.24 [0.10, 0.33]	0.95 [0.92, 1.00]
lr0.001_wd0.01_h400_d2	0.72 [0.63, 0.84]	0.32 [0.18, 0.53]	0.28 [0.21, 0.40]	0.61 [0.50, 0.68]	0.17 [0.00, 0.31]	0.51 [0.00, 0.89]	0.70 [0.33, 1.00]	0.20 [0.12, 0.33]	0.76 [0.00, 1.00]
lr0.001_wd0.01_h512_d2	0.74 [0.60, 0.84]	0.31 [0.16, 0.41]	0.25 [0.20, 0.36]	0.59 [0.50, 0.66]	0.13 [0.00, 0.26]	0.44 [0.00, 0.83]	0.73 [0.50, 1.00]	0.17 [0.12, 0.29]	0.75 [0.00, 1.00]
lr0.0001_wd0.0_h1024_d1	0.74 [0.64, 0.78]	0.34 [0.21, 0.52]	0.28 [0.21, 0.36]	0.64 [0.61, 0.67]	0.21 [0.16, 0.26]	0.49 [0.21, 0.83]	0.80 [0.50, 1.00]	0.19 [0.12, 0.29]	0.97 [0.92, 1.00]
lr0.0001_wd0.0_h2048_d1	0.76 [0.67, 0.82]	0.30 [0.21, 0.36]	0.20 [0.00, 0.36]	0.58 [0.48, 0.66]	0.11 [-0.07, 0.26]	0.46 [0.00, 0.97]	0.70 [0.00, 1.00]	0.13 [0.00, 0.29]	0.76 [0.00, 1.00]
lr0.0001_wd0.0_h1024_d2	0.73 [0.62, 0.83]	0.38 [0.17, 0.61]	0.29 [0.18, 0.44]	0.60 [0.50, 0.70]	0.17 [0.00, 0.36]	0.47 [0.00, 0.90]	0.73 [0.50, 1.00]	0.22 [0.11, 0.40]	0.75 [0.00, 1.00]
lr0.0001_wd0.0_h2048_d2	0.75 [0.69, 0.83]	0.40 [0.21, 0.52]	0.27 [0.17, 0.40]	0.58 [0.49, 0.66]	0.19 [-0.01, 0.48]	0.48 [0.07, 1.00]	0.68 [0.25, 1.00]	0.33 [0.10, 1.00]	0.95 [0.90, 1.00]
lr0.0001_wd0.0001_h1024_d1	0.75 [0.60, 0.81]	0.34 [0.17, 0.52]	0.29 [0.22, 0.40]	0.63 [0.50, 0.68]	0.20 [0.00, 0.31]	0.45 [0.00, 0.86]	0.80 [0.50, 1.00]	0.21 [0.12, 0.33]	0.77 [0.00, 1.00]
lr0.0001_wd0.0001_h2048_d1	0.73 [0.65, 0.78]	0.32 [0.20, 0.45]	0.27 [0.17, 0.50]	0.58 [0.49, 0.72]	0.13 [-0.01, 0.43]	0.42 [0.00, 0.93]	0.73 [0.50, 1.00]	0.20 [0.10, 0.50]	0.75 [0.00, 1.00]
lr0.0001_wd0.0001_h1024_d2	0.73 [0.60, 0.79]	0.39 [0.20, 0.55]	0.26 [0.16, 0.44]	0.58 [0.48, 0.70]	0.12 [-0.03, 0.36]	0.42 [0.00, 0.90]	0.73 [0.50, 1.00]	0.18 [0.09, 0.40]	0.75 [0.00, 1.00]
lr0.0001_wd0.0001_h2048_d2	0.75 [0.63, 0.81]	0.38 [0.19, 0.61]	0.30 [0.17, 0.50]	0.60 [0.49, 0.72]	0.17 [-0.01, 0.43]	0.46 [0.00, 0.93]	0.73 [0.50, 1.00]	0.23 [0.10, 0.50]	0.75 [0.00, 1.00]
lr0.0001_wd0.001_h1024_d1	0.73 [0.64, 0.78]	0.34 [0.19, 0.59]	0.27 [0.18, 0.40]	0.61 [0.53, 0.68]	0.16 [0.04, 0.31]	0.54 [0.21, 0.86]	0.68 [0.50, 1.00]	0.19 [0.11, 0.33]	0.94 [0.91, 1.00]
lr0.0001_wd0.001_h2048_d1	0.73 [0.62, 0.78]	0.35 [0.15, 0.61]	0.32 [0.21, 0.44]	0.64 [0.61, 0.70]	0.26 [0.16, 0.36]	0.64 [0.21, 0.97]	0.65 [0.25, 1.00]	0.30 [0.12, 0.50]	0.95 [0.90, 1.00]
lr0.0001_wd0.001_h1024_d2	0.71 [0.62, 0.77]	0.33 [0.18, 0.52]	0.27 [0.17, 0.40]	0.60 [0.49, 0.68]	0.16 [-0.01, 0.31]	0.46 [0.10, 0.86]	0.73 [0.50, 1.00]	0.19 [0.10, 0.33]	0.95 [0.90, 1.00]
lr0.0001_wd0.001_h2048_d2	0.75 [0.67, 0.80]	0.42 [0.23, 0.60]	0.26 [0.18, 0.33]	0.59 [0.53, 0.65]	0.16 [0.04, 0.22]	0.60 [0.14, 0.93]	0.58 [0.25, 1.00]	0.20 [0.11, 0.33]	0.93 [0.90, 1.00]
lr0.0003_wd0.0_h1024_d1	0.75 [0.61, 0.84]	0.37 [0.19, 0.65]	0.25 [0.16, 0.40]	0.58 [0.48, 0.68]	0.14 [-0.03, 0.31]	0.32 [0.10, 0.86]	0.83 [0.50, 1.00]	0.16 [0.09, 0.33]	0.96 [0.89, 1.00]
lr0.0003_wd0.0_h2048_d1	0.76 [0.62, 0.87]	0.38 [0.22, 0.63]	0.32 [0.20, 0.44]	0.64 [0.57, 0.70]	0.23 [0.13, 0.36]	0.61 [0.14, 0.90]	0.68 [0.50, 1.00]	0.24 [0.11, 0.40]	0.95 [0.93, 1.00]
lr0.0003_wd0.0_h1024_d2	0.72 [0.65, 0.77]	0.35 [0.18, 0.61]	0.34 [0.20, 0.50]	0.64 [0.57, 0.72]	0.25 [0.10, 0.43]	0.65 [0.14, 0.93]	0.63 [0.50, 1.00]	0.29 [0.11, 0.50]	0.94 [0.91, 1.00]
lr0.0003_wd0.0_h2048_d2	0.75 [0.70, 0.82]	0.32 [0.19, 0.41]	0.23 [0.00, 0.40]	0.60 [0.50, 0.68]	0.15 [0.00, 0.31]	0.65 [0.14, 1.00]	0.55 [0.00, 1.00]	0.16 [0.00, 0.33]	0.94 [0.90, 1.00]
lr0.0003_wd0.0001_h1024_d1	0.74 [0.64, 0.79]	0.36 [0.18, 0.61]	0.36 [0.21, 0.50]	0.67 [0.61, 0.72]	0.29 [0.16, 0.43]	0.65 [0.21, 0.93]	0.70 [0.50, 1.00]	0.30 [0.12, 0.50]	0.96 [0.93, 1.00]
lr0.0003_wd0.0001_h2048_d1	0.76 [0.67, 0.81]	0.32 [0.21, 0.40]	0.32 [0.21, 0.40]	0.67 [0.58, 0.78]	0.25 [0.10, 0.36]	0.61 [0.29, 0.86]	0.73 [0.50, 1.00]	0.23 [0.12, 0.33]	0.96 [0.93, 1.00]
lr0.0003_wd0.0001_h1024_d2	0.71 [0.61, 0.78]	0.37 [0.16, 0.62]	0.25 [0.18, 0.33]	0.60 [0.54, 0.65]	0.14 [0.06, 0.22]	0.63 [0.25, 0.79]	0.57 [0.33, 1.00]	0.17 [0.12, 0.25]	0.93 [0.90, 1.00]
lr0.0003_wd0.0001_h2048_d2	0.71 [0.61, 0.84]	0.26 [0.17, 0.39]	0.27 [0.19, 0.44]	0.61 [0.55, 0.70]	0.19 [0.10, 0.36]	0.47 [0.11, 0.90]	0.75 [0.25, 1.00]	0.20 [0.11, 0.40]	0.96 [0.89, 1.00]
lr0.0003_wd0.001_h1024_d1	0.76 [0.68, 0.82]	0.35 [0.18, 0.50]	0.34 [0.21, 0.46]	0.70 [0.58, 0.77]	0.28 [0.10, 0.40]	0.61 [0.41, 0.86]	0.78 [0.50, 1.00]	0.23 [0.12, 0.33]	0.96 [0.93, 1.00]
lr0.0003_wd0.001_h2048_d1	0.76 [0.65, 0.86]	0.32 [0.19, 0.43]	0.27 [0.17, 0.33]	0.64 [0.51, 0.72]	0.19 [0.01, 0.30]	0.39 [0.18, 0.62]	0.88 [0.67, 1.00]	0.16 [0.10, 0.21]	0.97 [0.91, 1.00]
lr0.0003_wd0.001_h1024_d2	0.71 [0.57, 0.78]	0.40 [0.20, 0.61]	0.24 [0.12, 0.33]	0.57 [0.45, 0.62]	0.13 [-0.06, 0.29]	0.58 [0.25, 0.97]	0.57 [0.25, 1.00]	0.20 [0.08, 0.50]	0.93 [0.89, 1.00]
lr0.0003_wd0.001_h2048_d2	0.74 [0.60, 0.84]	0.36 [0.17, 0.59]	0.27 [0.21, 0.36]	0.63 [0.58, 0.76]	0.19 [0.10, 0.34]	0.58 [0.21, 0.93]	0.68 [0.25, 1.00]	0.20 [0.12, 0.33]	0.95 [0.90, 1.00]
lr0.001_wd0.0_h1024_d1	0.76 [0.65, 0.85]	0.31 [0.23, 0.40]	0.30 [0.23, 0.44]	0.65 [0.62, 0.70]	0.23 [0.14, 0.36]	0.56 [0.28, 0.90]	0.73 [0.50, 1.00]	0.22 [0.13, 0.40]	0.96 [0.92, 1.00]
lr0.001_wd0.0_h2048_d1	0.77 [0.63, 0.90]	0.33 [0.16, 0.62]	0.19 [0.00, 0.25]	0.57 [0.48, 0.66]	0.10 [-0.07, 0.21]	0.34 [0.00, 0.97]	0.80 [0.00, 1.00]	0.11 [0.00, 0.14]	0.78 [0.00, 1.00]
lr0.001_wd0.0_h1024_d2	0.74 [0.61, 0.86]	0.36 [0.19, 0.66]	0.29 [0.18, 0.50]	0.65 [0.53, 0.79]	0.21 [0.04, 0.44]	0.51 [0.28, 0.83]	0.78 [0.50, 1.00]	0.19 [0.11, 0.38]	0.96 [0.90, 1.00]
lr0.001_wd0.0_h2048_d2	0.81 [0.74, 0.89]	0.41 [0.22, 0.65]	0.33 [0.00, 0.57]	0.64 [0.46, 0.75]	0.25 [-0.09, 0.53]	0.73 [0.11, 0.97]	0.55 [0.00, 1.00]	0.29 [0.00, 0.67]	0.94 [0.90, 1.00]
lr0.001_wd0.0001_h1024_d1	0.76 [0.60, 0.86]	0.33 [0.20, 0.43]	0.30 [0.13, 0.50]	0.62 [0.47, 0.74]	0.20 [-0.04, 0.43]	0.52 [0.14, 0.93]	0.72 [0.33, 1.00]	0.22 [0.08, 0.50]	0.96 [0.89, 1.00]
lr0.001_wd0.0001_h2048_d1	0.76 [0.67, 0.85]	0.36 [0.23, 0.53]	0.35 [0.18, 0.67]	0.65 [0.53, 0.84]	0.26 [0.04, 0.62]	0.57 [0.21, 0.93]	0.73 [0.50, 1.00]	0.28 [0.11, 0.60]	0.95 [0.92, 1.00]
lr0.001_wd0.0001_h1024_d2	0.74 [0.69, 0.79]	0.33 [0.24, 0.49]	0.22 [0.00, 0.40]	0.59 [0.50, 0.68]	0.14 [0.00, 0.31]	0.48 [0.00, 1.00]	0.70 [0.00, 1.00]	0.15 [0.00, 0.33]	0.77 [0.00, 1.00]

Supplementary Table 32: Grid search results on the classification task on GDHCM retrospective dataset (choosing 60% as the threshold for clinical variables selection). Reported metrics are AUC, AUPRC, F1, Balanced Accuracy, MCC, Specificity, Sensitivity, PPV, and NPV.

Config	AUC	AUPRC	F1	Balanced_Accuracy	MCC	Specificity	Sensitivity	PPV	NPV
lr0.001_wd0.0001_h2048_d2	0.76 [0.71, 0.84]	0.33 [0.23, 0.42]	0.35 [0.23, 0.50]	0.67 [0.59, 0.79]	0.26 [0.13, 0.44]	0.72 [0.29, 0.90]	0.62 [0.33, 1.00]	0.27 [0.13, 0.40]	0.94 [0.91, 1.00]
lr0.001_wd0.001_h1024_d1	0.75 [0.67, 0.83]	0.30 [0.19, 0.39]	0.28 [0.23, 0.33]	0.63 [0.59, 0.66]	0.20 [0.19, 0.22]	0.62 [0.29, 0.93]	0.65 [0.25, 1.00]	0.21 [0.13, 0.33]	0.95 [0.90, 1.00]
lr0.001_wd0.001_h2048_d1	0.75 [0.65, 0.85]	0.30 [0.19, 0.40]	0.29 [0.21, 0.36]	0.65 [0.59, 0.70]	0.22 [0.14, 0.27]	0.46 [0.18, 0.83]	0.85 [0.50, 1.00]	0.19 [0.12, 0.29]	0.97 [0.92, 1.00]
lr0.001_wd0.001_h1024_d2	0.73 [0.67, 0.81]	0.39 [0.20, 0.61]	0.27 [0.17, 0.40]	0.60 [0.52, 0.69]	0.20 [0.03, 0.48]	0.59 [0.18, 1.00]	0.62 [0.25, 1.00]	0.31 [0.11, 1.00]	0.94 [0.90, 1.00]
lr0.001_wd0.001_h2048_d2	0.75 [0.64, 0.84]	0.38 [0.19, 0.59]	0.34 [0.15, 0.60]	0.67 [0.46, 0.82]	0.27 [-0.06, 0.55]	0.61 [0.25, 0.97]	0.73 [0.25, 1.00]	0.29 [0.09, 0.50]	0.95 [0.88, 1.00]
lr0.0001_wd0.0_h256_d3	0.75 [0.62, 0.88]	0.35 [0.17, 0.49]	0.22 [0.00, 0.44]	0.56 [0.50, 0.70]	0.10 [0.00, 0.36]	0.42 [0.00, 1.00]	0.70 [0.00, 1.00]	0.15 [0.00, 0.40]	0.57 [0.00, 1.00]
lr0.0001_wd0.0_h400_d3	0.73 [0.65, 0.82]	0.33 [0.18, 0.47]	0.21 [0.00, 0.33]	0.57 [0.50, 0.65]	0.11 [0.00, 0.22]	0.58 [0.00, 1.00]	0.55 [0.00, 1.00]	0.16 [0.00, 0.33]	0.74 [0.00, 1.00]
lr0.0001_wd0.0_h512_d3	0.73 [0.68, 0.81]	0.40 [0.17, 0.61]	0.21 [0.00, 0.38]	0.58 [0.50, 0.72]	0.11 [0.00, 0.30]	0.50 [0.00, 1.00]	0.65 [0.00, 1.00]	0.13 [0.00, 0.25]	0.75 [0.00, 1.00]
lr0.0001_wd0.0_h256_d4	0.72 [0.60, 0.85]	0.35 [0.15, 0.53]	0.21 [0.00, 0.40]	0.56 [0.50, 0.68]	0.09 [0.00, 0.31]	0.42 [0.00, 1.00]	0.70 [0.00, 1.00]	0.14 [0.00, 0.33]	0.57 [0.00, 1.00]
lr0.0001_wd0.0_h400_d4	0.75 [0.69, 0.84]	0.30 [0.19, 0.39]	0.21 [0.00, 0.40]	0.56 [0.50, 0.68]	0.10 [0.00, 0.31]	0.42 [0.00, 1.00]	0.70 [0.00, 1.00]	0.14 [0.00, 0.33]	0.57 [0.00, 1.00]
lr0.0001_wd0.0_h512_d4	0.75 [0.62, 0.81]	0.35 [0.16, 0.55]	0.20 [0.00, 0.36]	0.55 [0.50, 0.66]	0.08 [0.00, 0.26]	0.41 [0.00, 1.00]	0.70 [0.00, 1.00]	0.13 [0.00, 0.29]	0.57 [0.00, 1.00]
lr0.0001_wd0.0001_h256_d3	0.71 [0.63, 0.80]	0.33 [0.16, 0.47]	0.22 [0.00, 0.40]	0.59 [0.50, 0.68]	0.14 [0.00, 0.31]	0.48 [0.00, 1.00]	0.70 [0.00, 1.00]	0.15 [0.00, 0.33]	0.77 [0.00, 1.00]
lr0.0001_wd0.0001_h400_d3	0.76 [0.68, 0.81]	0.34 [0.18, 0.52]	0.23 [0.00, 0.36]	0.58 [0.50, 0.66]	0.13 [0.00, 0.26]	0.57 [0.00, 1.00]	0.60 [0.00, 1.00]	0.16 [0.00, 0.29]	0.75 [0.00, 1.00]
lr0.0001_wd0.0001_h512_d3	0.73 [0.61, 0.85]	0.37 [0.16, 0.57]	0.20 [0.00, 0.33]	0.55 [0.50, 0.65]	0.08 [0.00, 0.22]	0.40 [0.00, 1.00]	0.70 [0.00, 1.00]	0.12 [0.00, 0.25]	0.56 [0.00, 1.00]
lr0.0001_wd0.0001_h256_d4	0.71 [0.61, 0.84]	0.40 [0.22, 0.63]	0.19 [0.00, 0.36]	0.53 [0.50, 0.66]	0.05 [0.00, 0.26]	0.37 [0.00, 1.00]	0.70 [0.00, 1.00]	0.12 [0.00, 0.29]	0.37 [0.00, 0.92]
lr0.0001_wd0.0001_h400_d4	0.74 [0.65, 0.85]	0.46 [0.18, 0.73]	0.15 [0.00, 0.33]	0.53 [0.50, 0.65]	0.04 [0.00, 0.22]	0.56 [0.00, 1.00]	0.50 [0.00, 1.00]	0.10 [0.00, 0.25]	0.55 [0.00, 0.92]
lr0.0001_wd0.0001_h512_d4	0.73 [0.62, 0.84]	0.39 [0.19, 0.53]	0.24 [0.18, 0.40]	0.56 [0.50, 0.68]	0.09 [0.00, 0.31]	0.34 [0.00, 0.86]	0.77 [0.33, 1.00]	0.16 [0.11, 0.33]	0.57 [0.00, 1.00]
lr0.0001_wd0.001_h256_d3	0.73 [0.65, 0.84]	0.40 [0.17, 0.61]	0.22 [0.00, 0.40]	0.59 [0.50, 0.68]	0.14 [0.00, 0.31]	0.47 [0.00, 1.00]	0.70 [0.00, 1.00]	0.15 [0.00, 0.33]	0.77 [0.00, 1.00]
lr0.0001_wd0.001_h400_d3	0.74 [0.62, 0.82]	0.39 [0.15, 0.63]	0.22 [0.00, 0.40]	0.57 [0.50, 0.68]	0.11 [0.00, 0.31]	0.44 [0.00, 1.00]	0.70 [0.00, 1.00]	0.14 [0.00, 0.33]	0.57 [0.00, 1.00]
lr0.0001_wd0.001_h512_d3	0.74 [0.67, 0.82]	0.37 [0.19, 0.61]	0.21 [0.00, 0.40]	0.55 [0.50, 0.68]	0.08 [0.00, 0.31]	0.39 [0.00, 1.00]	0.70 [0.00, 1.00]	0.14 [0.00, 0.33]	0.57 [0.00, 1.00]
lr0.0001_wd0.001_h256_d4	0.74 [0.62, 0.82]	0.40 [0.15, 0.62]	0.13 [0.00, 0.22]	0.51 [0.50, 0.54]	0.01 [0.00, 0.06]	0.52 [0.00, 1.00]	0.50 [0.00, 1.00]	0.08 [0.00, 0.14]	0.54 [0.00, 0.90]
lr0.0001_wd0.001_h400_d4	0.72 [0.63, 0.84]	0.39 [0.16, 0.52]	0.21 [0.00, 0.36]	0.56 [0.50, 0.66]	0.09 [0.00, 0.26]	0.43 [0.00, 1.00]	0.70 [0.00, 1.00]	0.13 [0.00, 0.29]	0.57 [0.00, 1.00]
lr0.0001_wd0.001_h512_d4	0.74 [0.63, 0.82]	0.34 [0.22, 0.39]	0.25 [0.00, 0.43]	0.60 [0.50, 0.75]	0.15 [0.00, 0.36]	0.55 [0.00, 1.00]	0.65 [0.00, 1.00]	0.17 [0.00, 0.33]	0.76 [0.00, 1.00]
lr0.0003_wd0.0_h256_d3	0.72 [0.63, 0.80]	0.36 [0.20, 0.59]	0.27 [0.21, 0.40]	0.62 [0.50, 0.70]	0.18 [0.00, 0.31]	0.33 [0.00, 0.86]	0.90 [0.50, 1.00]	0.17 [0.12, 0.33]	0.79 [0.00, 1.00]
lr0.0003_wd0.0_h400_d3	0.73 [0.65, 0.81]	0.30 [0.17, 0.40]	0.24 [0.22, 0.31]	0.57 [0.50, 0.63]	0.09 [0.00, 0.19]	0.37 [0.00, 0.76]	0.77 [0.50, 1.00]	0.15 [0.12, 0.22]	0.56 [0.00, 0.94]
lr0.0003_wd0.0_h512_d3	0.74 [0.63, 0.80]	0.31 [0.18, 0.40]	0.23 [0.18, 0.31]	0.56 [0.50, 0.64]	0.08 [0.00, 0.19]	0.28 [0.00, 0.76]	0.83 [0.50, 1.00]	0.14 [0.10, 0.22]	0.57 [0.00, 1.00]
lr0.0003_wd0.0_h256_d4	0.72 [0.65, 0.78]	0.35 [0.17, 0.49]	0.25 [0.18, 0.40]	0.56 [0.50, 0.68]	0.09 [0.00, 0.31]	0.29 [0.00, 0.86]	0.83 [0.50, 1.00]	0.17 [0.10, 0.33]	0.37 [0.00, 0.94]
lr0.0003_wd0.0_h400_d4	0.70 [0.58, 0.80]	0.32 [0.15, 0.56]	0.30 [0.18, 0.55]	0.63 [0.50, 0.81]	0.19 [0.00, 0.49]	0.47 [0.00, 0.86]	0.78 [0.50, 1.00]	0.20 [0.11, 0.43]	0.76 [0.00, 1.00]
lr0.0003_wd0.0_h512_d4	0.72 [0.61, 0.82]	0.32 [0.16, 0.44]	0.24 [0.17, 0.36]	0.55 [0.50, 0.66]	0.10 [0.00, 0.26]	0.42 [0.00, 0.90]	0.68 [0.25, 1.00]	0.17 [0.10, 0.29]	0.75 [0.00, 1.00]
lr0.0003_wd0.0001_h256_d3	0.73 [0.57, 0.84]	0.38 [0.20, 0.53]	0.24 [0.16, 0.40]	0.54 [0.48, 0.68]	0.07 [-0.03, 0.31]	0.32 [0.00, 0.86]	0.77 [0.50, 1.00]	0.16 [0.09, 0.33]	0.55 [0.00, 0.93]
lr0.0003_wd0.0001_h400_d3	0.75 [0.61, 0.87]	0.29 [0.17, 0.39]	0.24 [0.14, 0.40]	0.57 [0.49, 0.74]	0.09 [-0.01, 0.33]	0.39 [0.00, 0.72]	0.75 [0.33, 1.00]	0.15 [0.09, 0.27]	0.56 [0.00, 0.95]
lr0.0003_wd0.0001_h512_d3	0.74 [0.61, 0.84]	0.41 [0.20, 0.56]	0.19 [0.00, 0.36]	0.54 [0.50, 0.66]	0.08 [0.00, 0.26]	0.52 [0.00, 1.00]	0.57 [0.00, 1.00]	0.12 [0.00, 0.29]	0.74 [0.00, 1.00]
lr0.0003_wd0.0001_h256_d4	0.74 [0.64, 0.86]	0.38 [0.20, 0.46]	0.24 [0.14, 0.40]	0.56 [0.49, 0.68]	0.09 [-0.01, 0.31]	0.42 [0.00, 0.86]	0.70 [0.33, 1.00]	0.16 [0.09, 0.33]	0.55 [0.00, 0.94]
lr0.0003_wd0.0001_h400_d4	0.74 [0.65, 0.84]	0.34 [0.17, 0.53]	0.32 [0.20, 0.40]	0.66 [0.57, 0.78]	0.24 [0.08, 0.36]	0.66 [0.46, 0.86]	0.67 [0.50, 1.00]	0.23 [0.12, 0.33]	0.94 [0.93, 1.00]
lr0.0003_wd0.0001_h512_d4	0.74 [0.60, 0.84]	0.29 [0.17, 0.39]	0.27 [0.20, 0.40]	0.60 [0.50, 0.68]	0.15 [0.00, 0.31]	0.60 [0.00, 0.86]	0.60 [0.33, 1.00]	0.19 [0.12, 0.33]	0.74 [0.00, 0.94]
lr0.0003_wd0.001_h256_d3	0.72 [0.60, 0.80]	0.30 [0.15, 0.53]	0.23 [0.21, 0.25]	0.58 [0.50, 0.68]	0.12 [0.00, 0.23]	0.42 [0.00, 0.90]	0.75 [0.25, 1.00]	0.16 [0.12, 0.25]	0.76 [0.00, 1.00]
lr0.0003_wd0.001_h400_d3	0.73 [0.61, 0.84]	0.31 [0.16, 0.45]	0.34 [0.21, 0.44]	0.67 [0.60, 0.75]	0.26 [0.12, 0.36]	0.65 [0.21, 0.90]	0.68 [0.50, 1.00]	0.26 [0.12, 0.40]	0.95 [0.93, 1.00]
lr0.0003_wd0.001_h512_d3	0.73 [0.61, 0.85]	0.35 [0.16, 0.57]	0.20 [0.00, 0.29]	0.55 [0.39, 0.63]	0.06 [-0.16, 0.17]	0.39 [0.00, 0.79]	0.70 [0.00, 1.00]	0.12 [0.00, 0.18]	0.75 [0.00, 1.00]
lr0.0003_wd0.001_h256_d4	0.70 [0.60, 0.76]	0.37 [0.18, 0.48]	0.21 [0.00, 0.33]	0.56 [0.50, 0.63]	0.12 [0.00, 0.29]	0.64 [0.00, 1.00]	0.48 [0.00, 1.00]	0.19 [0.00, 0.50]	0.73 [0.00, 0.93]
lr0.0003_wd0.001_h400_d4	0.73 [0.60, 0.83]	0.33 [0.19, 0.44]	0.25 [0.15, 0.31]	0.57 [0.46, 0.69]	0.11 [-0.06, 0.24]	0.52 [0.00, 0.93]	0.62 [0.25, 1.00]	0.19 [0.09, 0.33]	0.73 [0.00, 0.95]
lr0.0003_wd0.001_h512_d4	0.74 [0.62, 0.84]	0.36 [0.23, 0.45]	0.24 [0.17, 0.33]	0.55 [0.50, 0.61]	0.10 [0.00, 0.29]	0.48 [0.00, 0.97]	0.62 [0.25, 1.00]	0.21 [0.10, 0.50]	0.55 [0.00, 0.91]
lr0.001_wd0.0_h256_d3	0.72 [0.62, 0.85]	0.33 [0.17, 0.41]	0.22 [0.00, 0.40]	0.56 [0.49, 0.68]	0.10 [-0.01, 0.31]	0.66 [0.00, 1.00]	0.47 [0.00, 1.00]	0.16 [0.00, 0.33]	0.73 [0.00, 0.93]
lr0.001_wd0.0_h400_d3	0.73 [0.67, 0.83]	0.37 [0.18, 0.65]	0.31 [0.21, 0.40]	0.66 [0.56, 0.73]	0.22 [0.08, 0.31]	0.63 [0.21, 0.86]	0.68 [0.50, 1.00]	0.22 [0.12, 0.33]	0.95 [0.90, 1.00]
lr0.001_wd0.0_h512_d3	0.75 [0.61, 0.88]	0.28 [0.20, 0.42]	0.25 [0.18, 0.38]	0.59 [0.50, 0.72]	0.13 [0.00, 0.30]	0.43 [0.00, 0.75]	0.75 [0.33, 1.00]	0.16 [0.12, 0.25]	0.76 [0.00, 1.00]
lr0.001_wd0.0_h256_d4	0.72 [0.63, 0.81]	0.34 [0.20, 0.51]	0.32 [0.22, 0.50]	0.64 [0.50, 0.72]	0.21 [0.00, 0.43]	0.61 [0.00, 0.93]	0.67 [0.50, 1.00]	0.25 [0.12, 0.50]	0.75 [0.00, 0.95]
lr0.001_wd0.0_h400_d4	0.75 [0.61, 0.86]	0.36 [0.18, 0.43]	0.31 [0.17, 0.44]	0.65 [0.52, 0.72]	0.22 [0.03, 0.36]	0.66 [0.45, 0.90]	0.65 [0.33, 1.00]	0.22 [0.11, 0.40]	0.95 [0.91, 1.00]
lr0.001_wd0.0_h512_d4	0.76 [0.69, 0.87]	0.38 [0.23, 0.52]	0.33 [0.24, 0.47]	0.67 [0.60, 0.84]	0.24 [0.14, 0.46]	0.74 [0.57, 0.86]	0.60 [0.33, 1.00]	0.23 [0.14, 0.31]	0.94 [0.92, 1.00]
lr0.001_wd0.0001_h256_d3	0.74 [0.63, 0.91]	0.34 [0.18, 0.52]	0.31 [0.20, 0.57]	0.66 [0.50, 0.90]	0.21 [0.00, 0.56]	0.49 [0.00, 0.79]	0.83 [0.50, 1.00]	0.20 [0.12, 0.40]	0.77 [0.00, 1.00]
lr0.001_wd0.0001_h400_d3	0.75 [0.65, 0.84]	0.35 [0.19, 0.43]	0.29 [0.13, 0.55]	0.62 [0.47, 0.81]	0.19 [-0.04, 0.49]	0.53 [0.03, 0.86]	0.72 [0.33, 1.00]	0.20 [0.08, 0.43]	0.95 [0.89, 1.00]
lr0.001_wd0.0001_h512_d3	0.76 [0.68, 0.84]	0.35 [0.19, 0.64]	0.34 [0.21, 0.50]	0.68 [0.58, 0.79]	0.25 [0.10, 0.44]	0.68 [0.50, 0.83]	0.67 [0.50, 0.75]	0.23 [0.12, 0.38]	0.94 [0.92, 0.96]
lr0.001_wd0.0001_h256_d4	0.73 [0.63, 0.84]	0.36 [0.16, 0.61]	0.30 [0.15, 0.40]	0.63 [0.51, 0.68]	0.20 [0.01, 0.31]	0.66 [0.28, 0.86]	0.60 [0.33, 1.00]	0.22 [0.10, 0.33]	0.94 [0.90, 1.00]
lr0.001_wd0.0001_h400_d4	0.76 [0.71, 0.82]	0.32 [0.22, 0.43]	0.25 [0.00, 0.46]	0.59 [0.46, 0.77]	0.13 [-0.09, 0.40]	0.49 [0.00, 0.93]	0.70 [0.00, 1.00]	0.16 [0.00, 0.33]	0.56 [0.00, 0.96]
lr0.001_wd0.0001_h512_d4	0.77 [0.65, 0.83]	0.33 [0.16, 0.41]	0.28 [0.00, 0.50]	0.61 [0.48, 0.72]	0.18 [-0.06, 0.43]	0.73 [0.34, 0.96]	0.48 [0.00, 0.75]	0.23 [0.00, 0.50]	0.92 [0.90, 0.93]
lr0.001_wd0.001_h256_d3	0.72 [0.64, 0.79]	0.28 [0.18, 0.42]	0.31 [0.21, 0.44]	0.67 [0.58, 0.70]	0.24 [0.10, 0.36]	0.56 [0.38, 0.90]	0.78 [0.50, 1.00]	0.21 [0.12, 0.40]	0.96 [0.93, 1.00]
lr0.001_wd0.001_h400_d3	0.77 [0.63, 0.84]	0.34 [0.21, 0.45]	0.26 [0.16, 0.33]	0.59 [0.48, 0.69]	0.16 [-0.03, 0.29]	0.45 [0.04, 0.97]	0.73 [0.25, 1.00]	0.22 [0.09, 0.50]	0.95 [0.89, 1.00]
lr0.001_wd0.001_h512_d3	0.72 [0.62, 0.84]	0.35 [0.19, 0.42]	0.25 [0.17, 0.36]	0.57 [0.50, 0.66]	0.13 [0.00, 0.29]	0.59 [0.00, 0.97]	0.55 [0.25, 1.00]	0.23 [0.11, 0.50]	0.73 [0.00, 0.92]
lr0.001_wd0.001_h256_d4	0.75 [0.62, 0.88]	0.34 [0.18, 0.56]	0.26 [0.18, 0.40]	0.59 [0.50, 0.68]	0.13 [0.00, 0.31]	0.33 [0.00, 0.86]	0.85 [0.50, 1.00]	0.17 [0.10, 0.33]	0.57 [0.00, 1.00]
lr0.001_wd0.001_h400_d4	0.73 [0.69, 0.78]	0.28 [0.16, 0.38]	0.28 [0.20, 0.44]	0.61 [0.50, 0.71]	0.18 [0.00, 0.36]	0.46 [0.00, 0.90]	0.77 [0.33, 1.00]	0.19 [0.12, 0.40]	0.77 [0.00, 1.00]
lr0.001_wd0.001_h512_d4	0.76 [0.68, 0.88]	0.35 [0.26, 0.48]	0.31 [0.22, 0.60]	0.62 [0.50, 0.82]	0.19 [0.00, 0.55]	0.37 [0.00, 0.90]	0.88 [0.67, 1.00]	0.21 [0.12, 0.50]	0.58 [0.00, 1.00]

Supplementary Table 33: Grid search results on the classification task on GDHCM retrospective dataset (choosing 60% as the threshold for clinical variables selection). Reported metrics are AUC, AUPRC, F1, Balanced Accuracy, MCC, Specificity, Sensitivity, PPV, and NPV.

Config	AUC	AUPRC	F1	Balanced_Accuracy	MCC	Specificity	Sensitivity	PPV	NPV
lr0.0001_wd0.0_h256_d1	0.75[0.59, 0.96]	0.48[0.31, 0.87]	0.31[0.00, 0.43]	0.64[0.50, 0.79]	0.22[0.00, 0.40]	0.69[0.30, 1.00]	0.60[0.00, 1.00]	0.22[0.00, 0.33]	0.93[0.86, 1.00]
lr0.0001_wd0.0_h400_d1	0.72[0.65, 0.88]	0.36[0.22, 0.67]	0.32[0.25, 0.40]	0.59[0.50, 0.68]	0.15[0.00, 0.27]	0.41[0.00, 0.80]	0.77[0.33, 1.00]	0.22[0.17, 0.33]	0.75[0.00, 1.00]
lr0.0001_wd0.0_h512_d1	0.74[0.68, 0.88]	0.45[0.29, 0.54]	0.41[0.31, 0.50]	0.67[0.55, 0.75]	0.28[0.13, 0.42]	0.62[0.10, 0.84]	0.72[0.50, 1.00]	0.30[0.18, 0.40]	0.94[0.89, 1.00]
lr0.0001_wd0.0_h256_d2	0.73[0.66, 0.82]	0.43[0.26, 0.63]	0.26[0.00, 0.38]	0.58[0.50, 0.74]	0.12[0.00, 0.33]	0.45[0.00, 1.00]	0.70[0.00, 1.00]	0.16[0.00, 0.23]	0.75[0.00, 1.00]
lr0.0001_wd0.0_h400_d2	0.76[0.66, 0.91]	0.47[0.31, 0.70]	0.27[0.00, 0.43]	0.59[0.50, 0.79]	0.13[0.00, 0.40]	0.38[0.00, 1.00]	0.80[0.00, 1.00]	0.17[0.00, 0.27]	0.57[0.00, 1.00]
lr0.0001_wd0.0_h512_d2	0.71[0.65, 0.88]	0.45[0.26, 0.67]	0.38[0.29, 0.50]	0.62[0.50, 0.76]	0.29[0.00, 0.55]	0.52[0.00, 1.00]	0.72[0.25, 1.00]	0.52[0.17, 1.00]	0.75[0.00, 1.00]
lr0.0001_wd0.0001_h256_d1	0.73[0.60, 0.91]	0.45[0.25, 0.70]	0.22[0.00, 0.46]	0.57[0.45, 0.82]	0.09[-0.13, 0.44]	0.65[0.20, 0.90]	0.50[0.00, 1.00]	0.14[0.00, 0.30]	0.91[0.82, 1.00]
lr0.0001_wd0.0001_h400_d1	0.74[0.60, 0.91]	0.43[0.30, 0.64]	0.36[0.30, 0.50]	0.64[0.55, 0.84]	0.22[0.08, 0.48]	0.51[0.10, 0.89]	0.77[0.33, 1.00]	0.25[0.18, 0.33]	0.94[0.88, 1.00]
lr0.0001_wd0.0001_h512_d1	0.71[0.64, 0.86]	0.33[0.26, 0.41]	0.30[0.00, 0.50]	0.60[0.47, 0.75]	0.16[-0.09, 0.42]	0.62[0.10, 0.95]	0.58[0.00, 1.00]	0.22[0.00, 0.40]	0.92[0.86, 1.00]
lr0.0001_wd0.0001_h256_d2	0.78[0.62, 0.96]	0.55[0.27, 0.87]	0.18[0.00, 0.40]	0.51[0.40, 0.70]	0.01[-0.20, 0.32]	0.47[0.05, 1.00]	0.55[0.00, 1.00]	0.11[0.00, 0.25]	0.87[0.67, 1.00]
lr0.0001_wd0.0001_h400_d2	0.74[0.64, 0.93]	0.47[0.28, 0.70]	0.17[0.00, 0.33]	0.51[0.45, 0.60]	0.00[-0.13, 0.16]	0.52[0.00, 1.00]	0.50[0.00, 1.00]	0.11[0.00, 0.25]	0.51[0.00, 0.88]
lr0.0001_wd0.0001_h512_d2	0.75[0.64, 0.82]	0.44[0.27, 0.71]	0.22[0.00, 0.30]	0.51[0.50, 0.53]	0.04[0.00, 0.09]	0.22[0.00, 1.00]	0.80[0.00, 1.00]	0.13[0.00, 0.17]	0.57[0.00, 1.00]
lr0.0001_wd0.001_h256_d1	0.73[0.62, 0.95]	0.45[0.26, 0.76]	0.30[0.24, 0.33]	0.57[0.50, 0.61]	0.14[0.00, 0.23]	0.53[0.00, 0.89]	0.62[0.25, 1.00]	0.23[0.14, 0.33]	0.72[0.00, 1.00]
lr0.0001_wd0.001_h400_d1	0.69[0.59, 0.88]	0.36[0.22, 0.48]	0.39[0.29, 0.60]	0.67[0.55, 0.89]	0.26[0.08, 0.58]	0.55[0.15, 0.79]	0.78[0.50, 1.00]	0.26[0.19, 0.43]	0.94[0.86, 1.00]
lr0.0001_wd0.001_h512_d1	0.76[0.65, 0.89]	0.47[0.36, 0.61]	0.38[0.22, 0.60]	0.65[0.53, 0.89]	0.26[0.05, 0.58]	0.63[0.05, 0.95]	0.67[0.25, 1.00]	0.31[0.17, 0.50]	0.93[0.84, 1.00]
lr0.0001_wd0.001_h256_d2	0.79[0.65, 0.95]	0.50[0.28, 0.83]	0.28[0.00, 0.50]	0.59[0.50, 0.84]	0.17[0.00, 0.48]	0.39[0.05, 1.00]	0.80[0.00, 1.00]	0.17[0.00, 0.33]	0.97[0.86, 1.00]
lr0.0001_wd0.001_h400_d2	0.77[0.67, 0.91]	0.48[0.31, 0.70]	0.28[0.00, 0.43]	0.59[0.50, 0.79]	0.12[0.00, 0.40]	0.43[0.00, 1.00]	0.75[0.00, 1.00]	0.17[0.00, 0.27]	0.56[0.00, 1.00]
lr0.0001_wd0.001_h512_d2	0.79[0.68, 1.00]	0.53[0.29, 1.00]	0.23[0.00, 0.35]	0.53[0.50, 0.62]	0.04[0.00, 0.19]	0.30[0.00, 1.00]	0.75[0.00, 1.00]	0.14[0.00, 0.23]	0.35[0.00, 0.91]
lr0.0003_wd0.0_h256_d1	0.71[0.60, 0.89]	0.40[0.27, 0.78]	0.30[0.00, 0.46]	0.62[0.45, 0.82]	0.19[-0.13, 0.44]	0.80[0.63, 0.89]	0.45[0.00, 1.00]	0.24[0.00, 0.33]	0.90[0.85, 1.00]
lr0.0003_wd0.0_h400_d1	0.69[0.62, 0.84]	0.44[0.26, 0.60]	0.26[0.00, 0.40]	0.58[0.47, 0.66]	0.14[-0.09, 0.34]	0.64[0.32, 0.95]	0.52[0.00, 1.00]	0.22[0.00, 0.50]	0.89[0.83, 1.00]
lr0.0003_wd0.0_h512_d1	0.72[0.63, 0.89]	0.42[0.31, 0.51]	0.33[0.22, 0.40]	0.60[0.54, 0.65]	0.18[0.05, 0.26]	0.68[0.45, 0.89]	0.53[0.33, 0.75]	0.26[0.17, 0.33]	0.89[0.88, 0.91]
lr0.0003_wd0.0_h256_d2	0.73[0.65, 0.91]	0.47[0.28, 0.70]	0.40[0.33, 0.57]	0.67[0.60, 0.78]	0.31[0.20, 0.50]	0.66[0.25, 0.95]	0.68[0.25, 1.00]	0.35[0.21, 0.50]	0.94[0.86, 1.00]
lr0.0003_wd0.0_h400_d2	0.74[0.68, 0.89]	0.47[0.29, 0.71]	0.42[0.30, 0.57]	0.64[0.53, 0.78]	0.32[0.09, 0.55]	0.58[0.05, 1.00]	0.70[0.33, 1.00]	0.45[0.17, 1.00]	0.95[0.89, 1.00]
lr0.0003_wd0.0_h512_d2	0.69[0.62, 0.84]	0.39[0.25, 0.55]	0.31[0.00, 0.46]	0.62[0.45, 0.73]	0.18[-0.13, 0.35]	0.80[0.70, 0.89]	0.43[0.00, 0.75]	0.24[0.00, 0.33]	0.89[0.84, 0.94]
lr0.0003_wd0.0001_h256_d1	0.73[0.66, 0.95]	0.49[0.30, 0.83]	0.27[0.00, 0.40]	0.60[0.47, 0.70]	0.15[-0.09, 0.30]	0.67[0.26, 0.95]	0.53[0.00, 1.00]	0.19[0.00, 0.29]	0.91[0.83, 1.00]
lr0.0003_wd0.0001_h400_d1	0.74[0.64, 0.96]	0.45[0.26, 0.87]	0.34[0.29, 0.38]	0.63[0.55, 0.74]	0.21[0.08, 0.33]	0.63[0.47, 0.95]	0.63[0.25, 1.00]	0.28[0.20, 0.50]	0.91[0.86, 1.00]
lr0.0003_wd0.0001_h512_d1	0.72[0.63, 0.98]	0.48[0.27, 0.92]	0.38[0.25, 0.55]	0.65[0.50, 0.87]	0.25[0.00, 0.53]	0.69[0.50, 0.95]	0.62[0.33, 1.00]	0.31[0.17, 0.50]	0.91[0.83, 1.00]
lr0.0003_wd0.0001_h256_d2	0.74[0.64, 0.93]	0.54[0.30, 0.81]	0.28[0.00, 0.50]	0.56[0.45, 0.67]	0.15[-0.13, 0.55]	0.71[0.00, 1.00]	0.42[0.00, 1.00]	0.34[0.00, 1.00]	0.69[0.00, 0.90]
lr0.0003_wd0.0001_h400_d2	0.71[0.62, 0.88]	0.51[0.28, 0.77]	0.26[0.00, 0.38]	0.58[0.45, 0.68]	0.12[-0.13, 0.29]	0.61[0.35, 0.90]	0.55[0.00, 1.00]	0.18[0.00, 0.25]	0.90[0.82, 1.00]
lr0.0003_wd0.0001_h512_d2	0.73[0.64, 0.88]	0.48[0.28, 0.71]	0.39[0.29, 0.55]	0.64[0.50, 0.87]	0.27[0.00, 0.55]	0.51[0.00, 1.00]	0.77[0.33, 1.00]	0.39[0.17, 1.00]	0.76[0.00, 1.00]
lr0.0003_wd0.001_h256_d1	0.70[0.59, 0.89]	0.38[0.27, 0.50]	0.37[0.22, 0.43]	0.66[0.53, 0.79]	0.25[0.05, 0.40]	0.74[0.58, 0.80]	0.58[0.25, 1.00]	0.29[0.20, 0.33]	0.91[0.84, 1.00]
lr0.0003_wd0.001_h400_d1	0.71[0.62, 0.84]	0.50[0.24, 0.75]	0.35[0.29, 0.44]	0.63[0.53, 0.73]	0.21[0.04, 0.35]	0.47[0.21, 0.80]	0.78[0.50, 1.00]	0.24[0.17, 0.33]	0.94[0.86, 1.00]
lr0.0003_wd0.001_h512_d1	0.71[0.61, 0.88]	0.37[0.28, 0.48]	0.27[0.00, 0.50]	0.59[0.50, 0.75]	0.15[0.00, 0.42]	0.84[0.74, 1.00]	0.35[0.00, 0.67]	0.22[0.00, 0.40]	0.88[0.83, 0.94]
lr0.0003_wd0.001_h256_d2	0.72[0.62, 0.88]	0.43[0.24, 0.65]	0.30[0.00, 0.50]	0.61[0.50, 0.84]	0.16[0.00, 0.48]	0.58[0.00, 1.00]	0.65[0.00, 1.00]	0.20[0.00, 0.33]	0.73[0.00, 1.00]
lr0.0003_wd0.001_h400_d2	0.73[0.64, 0.91]	0.46[0.30, 0.62]	0.34[0.00, 0.67]	0.62[0.45, 0.92]	0.25[-0.13, 0.65]	0.73[0.05, 1.00]	0.52[0.00, 1.00]	0.38[0.00, 1.00]	0.91[0.82, 1.00]
lr0.0003_wd0.001_h512_d2	0.72[0.67, 0.93]	0.48[0.32, 0.59]	0.24[0.00, 0.46]	0.56[0.45, 0.72]	0.09[-0.08, 0.35]	0.61[0.05, 1.00]	0.50[0.00, 1.00]	0.16[0.00, 0.33]	0.90[0.81, 1.00]
lr0.001_wd0.0_h256_d1	0.76[0.64, 1.00]	0.48[0.28, 1.00]	0.36[0.29, 0.55]	0.64[0.53, 0.87]	0.21[0.04, 0.53]	0.66[0.30, 0.84]	0.62[0.33, 1.00]	0.26[0.18, 0.38]	0.90[0.86, 1.00]
lr0.001_wd0.0_h400_d1	0.81[0.69, 0.98]	0.62[0.47, 0.92]	0.41[0.36, 0.50]	0.71[0.62, 0.79]	0.32[0.20, 0.40]	0.67[0.47, 0.80]	0.75[0.50, 1.00]	0.30[0.23, 0.38]	0.94[0.88, 1.00]
lr0.001_wd0.0_h512_d1	0.76[0.66, 0.88]	0.48[0.34, 0.66]	0.39[0.36, 0.43]	0.69[0.65, 0.79]	0.28[0.22, 0.40]	0.60[0.55, 0.68]	0.78[0.67, 1.00]	0.26[0.25, 0.27]	0.94[0.92, 1.00]
lr0.001_wd0.0_h256_d2	0.73[0.61, 0.86]	0.49[0.33, 0.59]	0.38[0.22, 0.55]	0.65[0.54, 0.87]	0.24[0.05, 0.53]	0.68[0.35, 0.90]	0.62[0.33, 1.00]	0.30[0.17, 0.50]	0.91[0.88, 1.00]
lr0.001_wd0.0_h400_d2	0.75[0.65, 0.89]	0.51[0.42, 0.64]	0.39[0.00, 0.75]	0.69[0.47, 0.95]	0.29[-0.09, 0.73]	0.80[0.63, 0.95]	0.58[0.00, 1.00]	0.30[0.00, 0.60]	0.91[0.83, 1.00]
lr0.001_wd0.0_h512_d2	0.74[0.69, 0.91]	0.56[0.45, 0.79]	0.45[0.25, 0.67]	0.67[0.50, 0.81]	0.32[0.00, 0.61]	0.77[0.50, 0.95]	0.57[0.25, 0.75]	0.42[0.17, 0.67]	0.90[0.83, 0.95]
lr0.001_wd0.0001_h256_d1	0.76[0.68, 0.89]	0.52[0.32, 0.73]	0.47[0.29, 0.67]	0.71[0.53, 0.92]	0.35[0.04, 0.65]	0.64[0.30, 0.95]	0.78[0.67, 1.00]	0.37[0.18, 0.67]	0.93[0.86, 1.00]
lr0.001_wd0.0001_h400_d1	0.72[0.59, 0.93]	0.50[0.25, 0.72]	0.44[0.33, 0.50]	0.70[0.60, 0.82]	0.38[0.16, 0.55]	0.74[0.45, 1.00]	0.67[0.33, 1.00]	0.46[0.25, 1.00]	0.94[0.88, 1.00]
lr0.001_wd0.0001_h512_d1	0.76[0.69, 0.95]	0.51[0.35, 0.64]	0.39[0.25, 0.60]	0.67[0.56, 0.89]	0.28[0.10, 0.58]	0.73[0.50, 0.95]	0.62[0.25, 1.00]	0.33[0.20, 0.50]	0.92[0.86, 1.00]
lr0.001_wd0.0001_h256_d2	0.72[0.58, 0.89]	0.42[0.30, 0.53]	0.35[0.00, 0.60]	0.65[0.47, 0.89]	0.30[-0.09, 0.58]	0.84[0.50, 1.00]	0.47[0.00, 1.00]	0.43[0.00, 1.00]	0.90[0.83, 1.00]
lr0.001_wd0.0001_h400_d2	0.73[0.58, 0.86]	0.40[0.23, 0.51]	0.25[0.00, 0.33]	0.56[0.45, 0.62]	0.11[-0.13, 0.27]	0.73[0.35, 0.95]	0.38[0.00, 0.75]	0.24[0.00, 0.50]	0.87[0.85, 0.92]
lr0.001_wd0.0001_h512_d2	0.72[0.66, 0.91]	0.47[0.31, 0.71]	0.23[0.00, 0.60]	0.60[0.47, 0.89]	0.15[-0.09, 0.58]	0.90[0.79, 1.00]	0.30[0.00, 1.00]	0.20[0.00, 0.43]	0.88[0.83, 1.00]
lr0.001_wd0.001_h256_d1	0.73[0.65, 0.88]	0.49[0.35, 0.77]	0.36[0.33, 0.40]	0.65[0.60, 0.71]	0.24[0.15, 0.34]	0.57[0.42, 0.95]	0.72[0.33, 1.00]	0.28[0.21, 0.50]	0.92[0.90, 1.00]
lr0.001_wd0.001_h400_d1	0.72[0.62, 0.91]	0.42[0.26, 0.56]	0.25[0.00, 0.40]	0.58[0.47, 0.71]	0.13[-0.09, 0.34]	0.73[0.42, 0.95]	0.42[0.00, 1.00]	0.23[0.00, 0.50]	0.88[0.83, 1.00]
lr0.001_wd0.001_h512_d1	0.76[0.69, 0.86]	0.44[0.34, 0.64]	0.51[0.36, 0.67]	0.74[0.62, 0.82]	0.41[0.20, 0.60]	0.82[0.70, 0.90]	0.67[0.50, 0.75]	0.42[0.29, 0.60]	0.93[0.88, 0.95]
lr0.001_wd0.001_h256_d2	0.71[0.63, 0.84]	0.42[0.28, 0.65]	0.30[0.00, 0.44]	0.60[0.40, 0.72]	0.17[-0.20, 0.35]	0.77[0.45, 0.89]	0.43[0.00, 1.00]	0.25[0.00, 0.40]	0.90[0.80, 1.00]
lr0.001_wd0.001_h400_d2	0.74[0.64, 0.93]	0.44[0.29, 0.59]	0.33[0.00, 0.44]	0.63[0.50, 0.70]	0.24[0.00, 0.34]	0.87[0.74, 1.00]	0.40[0.00, 0.67]	0.30[0.00, 0.50]	0.89[0.83, 0.93]
lr0.001_wd0.001_h512_d2	0.74[0.64, 0.93]	0.48[0.28, 0.72]	0.39[0.25, 0.50]	0.68[0.50, 0.84]	0.26[0.00, 0.48]	0.57[0.35, 0.74]	0.78[0.50, 1.00]	0.26[0.17, 0.33]	0.94[0.83, 1.00]

Supplementary Table 34: Grid search results on the classification task on GDHCM prospective dataset (choosing 60% as the threshold for clinical variables selection). Reported metrics are AUC, AUPRC, F1, Balanced Accuracy, MCC, Specificity, Sensitivity, PPV, and NPV.

Config	AUC	AUPRC	F1	Balanced_Accuracy	MCC	Specificity	Sensitivity	PPV	NPV
lr1e-05_wd0.005_h1024_d3	0.75 [0.66, 0.89]	0.44 [0.28, 0.64]	0.22 [0.00, 0.29]	0.50 [0.50, 0.50]	0.00 [0.00, 0.00]	0.20 [0.00, 1.00]	0.80 [0.00, 1.00]	0.13 [0.00, 0.17]	0.17 [0.00, 0.86]
lr1e-05_wd0.005_h2048_d3	0.82 [0.58, 0.95]	0.52 [0.22, 0.82]	0.18 [0.00, 0.32]	0.52 [0.45, 0.66]	0.02 [-0.13, 0.24]	0.44 [0.00, 1.00]	0.60 [0.00, 1.00]	0.10 [0.00, 0.19]	0.54 [0.00, 1.00]
lr1e-05_wd0.005_h1024_d4	0.82 [0.65, 0.92]	0.53 [0.34, 0.77]	0.22 [0.00, 0.29]	0.50 [0.50, 0.50]	0.00 [0.00, 0.00]	0.20 [0.00, 1.00]	0.80 [0.00, 1.00]	0.13 [0.00, 0.17]	0.17 [0.00, 0.86]
lr1e-05_wd0.005_h2048_d4	0.78 [0.65, 0.89]	0.46 [0.28, 0.63]	0.25 [0.00, 0.40]	0.55 [0.50, 0.76]	0.07 [0.00, 0.36]	0.31 [0.00, 1.00]	0.80 [0.00, 1.00]	0.15 [0.00, 0.25]	0.37 [0.00, 1.00]
lr1e-05_wd0.01_h1024_d3	0.78 [0.70, 0.89]	0.47 [0.33, 0.64]	0.23 [0.00, 0.33]	0.53 [0.50, 0.60]	0.07 [0.00, 0.20]	0.26 [0.00, 1.00]	0.80 [0.00, 1.00]	0.14 [0.00, 0.20]	0.57 [0.00, 1.00]
lr1e-05_wd0.01_h2048_d3	0.73 [0.63, 0.89]	0.42 [0.25, 0.64]	0.17 [0.00, 0.33]	0.51 [0.43, 0.60]	0.02 [-0.17, 0.20]	0.42 [0.00, 1.00]	0.60 [0.00, 1.00]	0.10 [0.00, 0.20]	0.73 [0.00, 1.00]
lr1e-05_wd0.01_h1024_d4	0.79 [0.65, 0.98]	0.59 [0.35, 0.92]	0.22 [0.00, 0.29]	0.50 [0.50, 0.50]	0.00 [0.00, 0.00]	0.20 [0.00, 1.00]	0.80 [0.00, 1.00]	0.13 [0.00, 0.17]	0.17 [0.00, 0.86]
lr1e-05_wd0.01_h2048_d4	0.80 [0.69, 0.93]	0.54 [0.30, 0.70]	0.22 [0.00, 0.30]	0.51 [0.50, 0.53]	0.02 [0.00, 0.09]	0.21 [0.00, 1.00]	0.80 [0.00, 1.00]	0.13 [0.00, 0.17]	0.37 [0.00, 1.00]
lr3e-05_wd0.005_h1024_d3	0.78 [0.61, 0.91]	0.53 [0.25, 0.79]	0.16 [0.00, 0.29]	0.52 [0.45, 0.61]	0.03 [-0.13, 0.19]	0.59 [0.00, 1.00]	0.45 [0.00, 1.00]	0.12 [0.00, 0.25]	0.71 [0.00, 1.00]
lr3e-05_wd0.005_h2048_d3	0.76 [0.60, 0.95]	0.46 [0.23, 0.83]	0.25 [0.00, 0.36]	0.56 [0.50, 0.65]	0.11 [0.00, 0.26]	0.31 [0.00, 1.00]	0.80 [0.00, 1.00]	0.15 [0.00, 0.22]	0.77 [0.00, 1.00]
lr3e-05_wd0.005_h1024_d4	0.76 [0.60, 0.89]	0.46 [0.26, 0.68]	0.27 [0.00, 0.38]	0.60 [0.50, 0.74]	0.16 [0.00, 0.33]	0.40 [0.00, 1.00]	0.80 [0.00, 1.00]	0.17 [0.00, 0.23]	0.77 [0.00, 1.00]
lr3e-05_wd0.005_h2048_d4	0.77 [0.65, 0.88]	0.46 [0.25, 0.67]	0.17 [0.00, 0.29]	0.49 [0.45, 0.50]	-0.03 [-0.13, 0.00]	0.38 [0.00, 1.00]	0.60 [0.00, 1.00]	0.10 [0.00, 0.17]	0.34 [0.00, 0.86]
lr3e-05_wd0.01_h1024_d3	0.72 [0.56, 0.88]	0.41 [0.22, 0.63]	0.18 [0.00, 0.33]	0.53 [0.50, 0.60]	0.06 [0.00, 0.15]	0.51 [0.00, 1.00]	0.55 [0.00, 1.00]	0.11 [0.00, 0.21]	0.72 [0.00, 1.00]
lr3e-05_wd0.01_h2048_d3	0.73 [0.61, 0.89]	0.40 [0.26, 0.58]	0.26 [0.00, 0.43]	0.56 [0.50, 0.79]	0.08 [0.00, 0.40]	0.32 [0.00, 1.00]	0.80 [0.00, 1.00]	0.15 [0.00, 0.27]	0.37 [0.00, 1.00]
lr3e-05_wd0.01_h1024_d4	0.79 [0.68, 0.93]	0.52 [0.31, 0.70]	0.22 [0.00, 0.29]	0.50 [0.50, 0.50]	0.00 [0.00, 0.00]	0.25 [0.00, 1.00]	0.75 [0.00, 1.00]	0.13 [0.00, 0.17]	0.34 [0.00, 0.86]
lr3e-05_wd0.01_h2048_d4	0.80 [0.65, 0.98]	0.59 [0.29, 0.92]	0.17 [0.00, 0.32]	0.53 [0.43, 0.66]	0.03 [-0.17, 0.24]	0.60 [0.00, 1.00]	0.45 [0.00, 1.00]	0.12 [0.00, 0.25]	0.70 [0.00, 1.00]
lr1e-05_wd0.0_h256_d1	0.72 [0.61, 0.86]	0.34 [0.21, 0.49]	0.25 [0.00, 0.33]	0.57 [0.50, 0.61]	0.13 [0.00, 0.20]	0.39 [0.10, 1.00]	0.75 [0.00, 1.00]	0.15 [0.00, 0.21]	0.95 [0.86, 1.00]
lr1e-05_wd0.0_h400_d1	0.74 [0.60, 0.89]	0.44 [0.24, 0.68]	0.26 [0.00, 0.35]	0.57 [0.47, 0.71]	0.13 [-0.09, 0.30]	0.34 [0.05, 0.95]	0.80 [0.00, 1.00]	0.15 [0.00, 0.21]	0.97 [0.86, 1.00]
lr1e-05_wd0.0_h512_d1	0.73 [0.62, 0.93]	0.40 [0.26, 0.81]	0.40 [0.32, 0.67]	0.66 [0.57, 0.81]	0.29 [0.17, 0.61]	0.43 [0.15, 0.95]	0.88 [0.67, 1.00]	0.30 [0.19, 0.67]	0.97 [0.92, 1.00]
lr1e-05_wd0.0_h256_d2	0.82 [0.60, 0.98]	0.62 [0.26, 0.92]	0.33 [0.27, 0.46]	0.61 [0.50, 0.82]	0.19 [0.00, 0.44]	0.28 [0.05, 0.63]	0.95 [0.75, 1.00]	0.20 [0.17, 0.30]	0.97 [0.83, 1.00]
lr1e-05_wd0.0_h400_d2	0.81 [0.64, 0.96]	0.57 [0.25, 0.81]	0.37 [0.29, 0.57]	0.63 [0.50, 0.78]	0.23 [0.00, 0.50]	0.33 [0.00, 0.89]	0.93 [0.67, 1.00]	0.26 [0.17, 0.50]	0.79 [0.00, 1.00]
lr1e-05_wd0.0_h512_d2	0.78 [0.59, 0.91]	0.49 [0.29, 0.79]	0.32 [0.29, 0.38]	0.61 [0.55, 0.74]	0.20 [0.13, 0.33]	0.49 [0.10, 0.90]	0.72 [0.25, 1.00]	0.25 [0.18, 0.33]	0.95 [0.86, 1.00]
lr1e-05_wd0.0001_h256_d1	0.71 [0.64, 0.89]	0.37 [0.26, 0.53]	0.29 [0.26, 0.35]	0.56 [0.47, 0.62]	0.12 [-0.05, 0.23]	0.36 [0.16, 0.84]	0.77 [0.33, 1.00]	0.19 [0.16, 0.25]	0.91 [0.80, 1.00]
lr1e-05_wd0.0001_h400_d1	0.71 [0.59, 1.00]	0.58 [0.39, 1.00]	0.28 [0.17, 0.40]	0.55 [0.45, 0.64]	0.12 [-0.08, 0.34]	0.58 [0.05, 0.95]	0.52 [0.25, 1.00]	0.26 [0.12, 0.50]	0.89 [0.81, 1.00]
lr1e-05_wd0.0001_h512_d1	0.78 [0.68, 0.88]	0.43 [0.29, 0.62]	0.18 [0.00, 0.32]	0.54 [0.45, 0.63]	0.07 [-0.13, 0.22]	0.47 [0.05, 1.00]	0.60 [0.00, 1.00]	0.11 [0.00, 0.19]	0.94 [0.82, 1.00]
lr1e-05_wd0.0001_h256_d2	0.74 [0.61, 1.00]	0.46 [0.22, 1.00]	0.27 [0.24, 0.32]	0.52 [0.45, 0.57]	0.04 [-0.10, 0.17]	0.08 [0.00, 0.15]	0.95 [0.75, 1.00]	0.16 [0.14, 0.19]	0.55 [0.00, 1.00]
lr1e-05_wd0.0001_h400_d2	0.75 [0.66, 0.89]	0.42 [0.25, 0.68]	0.23 [0.00, 0.30]	0.53 [0.50, 0.63]	0.04 [0.00, 0.22]	0.25 [0.00, 1.00]	0.80 [0.00, 1.00]	0.14 [0.00, 0.18]	0.37 [0.00, 1.00]
lr1e-05_wd0.0001_h512_d2	0.72 [0.55, 0.88]	0.36 [0.22, 0.51]	0.31 [0.00, 0.53]	0.63 [0.50, 0.82]	0.20 [0.00, 0.49]	0.51 [0.05, 1.00]	0.75 [0.00, 1.00]	0.20 [0.00, 0.36]	0.94 [0.83, 1.00]
lr1e-05_wd0.001_h256_d1	0.74 [0.61, 0.96]	0.49 [0.25, 0.87]	0.16 [0.00, 0.32]	0.49 [0.40, 0.57]	-0.02 [-0.20, 0.13]	0.53 [0.11, 0.95]	0.45 [0.00, 1.00]	0.10 [0.00, 0.20]	0.87 [0.80, 1.00]
lr1e-05_wd0.001_h400_d1	0.69 [0.59, 0.88]	0.45 [0.24, 0.77]	0.32 [0.29, 0.33]	0.58 [0.50, 0.68]	0.15 [0.00, 0.27]	0.35 [0.00, 0.89]	0.82 [0.33, 1.00]	0.22 [0.17, 0.33]	0.76 [0.00, 1.00]
lr1e-05_wd0.001_h512_d1	0.77 [0.60, 0.90]	0.51 [0.30, 0.83]	0.36 [0.30, 0.43]	0.65 [0.53, 0.79]	0.25 [0.09, 0.40]	0.42 [0.05, 0.89]	0.87 [0.33, 1.00]	0.25 [0.17, 0.33]	0.98 [0.89, 1.00]
lr1e-05_wd0.001_h256_d2	0.72 [0.56, 0.93]	0.45 [0.22, 0.72]	0.25 [0.00, 0.36]	0.57 [0.50, 0.65]	0.13 [0.00, 0.26]	0.33 [0.05, 1.00]	0.80 [0.00, 1.00]	0.15 [0.00, 0.22]	0.97 [0.86, 1.00]
lr1e-05_wd0.001_h400_d2	0.77 [0.68, 0.91]	0.46 [0.26, 0.67]	0.29 [0.00, 0.57]	0.57 [0.42, 0.78]	0.11 [-0.17, 0.50]	0.40 [0.00, 0.89]	0.73 [0.00, 1.00]	0.20 [0.00, 0.50]	0.55 [0.00, 1.00]
lr1e-05_wd0.001_h512_d2	0.76 [0.62, 0.88]	0.39 [0.24, 0.62]	0.24 [0.00, 0.36]	0.56 [0.50, 0.62]	0.10 [0.00, 0.20]	0.41 [0.00, 1.00]	0.70 [0.00, 1.00]	0.15 [0.00, 0.29]	0.74 [0.00, 1.00]
lr3e-05_wd0.0_h256_d1	0.80 [0.66, 1.00]	0.56 [0.29, 1.00]	0.23 [0.00, 0.32]	0.53 [0.50, 0.57]	0.07 [0.00, 0.17]	0.31 [0.00, 1.00]	0.75 [0.00, 1.00]	0.14 [0.00, 0.19]	0.74 [0.00, 1.00]
lr3e-05_wd0.0_h400_d1	0.81 [0.59, 0.97]	0.58 [0.23, 0.92]	0.32 [0.17, 0.40]	0.60 [0.45, 0.76]	0.19 [-0.08, 0.36]	0.48 [0.10, 0.95]	0.72 [0.25, 1.00]	0.25 [0.12, 0.50]	0.94 [0.81, 1.00]
lr3e-05_wd0.0_h512_d1	0.80 [0.72, 0.89]	0.59 [0.34, 0.78]	0.22 [0.00, 0.46]	0.59 [0.47, 0.82]	0.12 [-0.09, 0.44]	0.63 [0.25, 1.00]	0.55 [0.00, 1.00]	0.14 [0.00, 0.30]	0.90 [0.83, 1.00]
lr3e-05_wd0.0_h256_d2	0.76 [0.66, 0.88]	0.47 [0.30, 0.62]	0.23 [0.00, 0.32]	0.54 [0.50, 0.58]	0.07 [0.00, 0.16]	0.32 [0.00, 1.00]	0.75 [0.00, 1.00]	0.14 [0.00, 0.20]	0.75 [0.00, 1.00]
lr3e-05_wd0.0_h400_d2	0.79 [0.70, 0.95]	0.54 [0.34, 0.83]	0.16 [0.00, 0.30]	0.51 [0.50, 0.53]	0.02 [0.00, 0.09]	0.41 [0.00, 1.00]	0.60 [0.00, 1.00]	0.10 [0.00, 0.17]	0.54 [0.00, 1.00]
lr3e-05_wd0.0_h512_d2	0.74 [0.60, 0.89]	0.53 [0.23, 0.78]	0.21 [0.00, 0.43]	0.57 [0.42, 0.79]	0.09 [-0.17, 0.40]	0.54 [0.00, 1.00]	0.60 [0.00, 1.00]	0.13 [0.00, 0.27]	0.73 [0.00, 1.00]
lr3e-05_wd0.0001_h256_d1	0.78 [0.62, 0.96]	0.49 [0.27, 0.87]	0.29 [0.00, 0.46]	0.64 [0.50, 0.72]	0.21 [0.00, 0.35]	0.67 [0.42, 1.00]	0.60 [0.00, 1.00]	0.20 [0.00, 0.33]	0.93 [0.84, 1.00]
lr3e-05_wd0.0001_h400_d1	0.75 [0.68, 0.86]	0.50 [0.29, 0.65]	0.37 [0.30, 0.50]	0.61 [0.53, 0.73]	0.26 [0.09, 0.55]	0.42 [0.05, 1.00]	0.80 [0.33, 1.00]	0.38 [0.17, 1.00]	0.97 [0.90, 1.00]
lr3e-05_wd0.0001_h512_d1	0.74 [0.60, 0.82]	0.44 [0.26, 0.61]	0.28 [0.00, 0.44]	0.58 [0.47, 0.73]	0.12 [-0.09, 0.35]	0.57 [0.05, 0.95]	0.58 [0.00, 1.00]	0.19 [0.00, 0.33]	0.91 [0.86, 1.00]
lr3e-05_wd0.0001_h256_d2	0.72 [0.64, 0.89]	0.47 [0.30, 0.78]	0.34 [0.31, 0.40]	0.63 [0.55, 0.70]	0.23 [0.13, 0.30]	0.33 [0.10, 0.74]	0.93 [0.67, 1.00]	0.22 [0.18, 0.29]	0.99 [0.93, 1.00]
lr3e-05_wd0.0001_h400_d2	0.74 [0.64, 0.91]	0.45 [0.28, 0.70]	0.25 [0.00, 0.35]	0.57 [0.47, 0.68]	0.12 [-0.09, 0.27]	0.33 [0.05, 0.95]	0.80 [0.00, 1.00]	0.15 [0.00, 0.21]	0.97 [0.86, 1.00]
lr3e-05_wd0.0001_h512_d2	0.77 [0.71, 0.91]	0.57 [0.33, 0.79]	0.30 [0.26, 0.35]	0.57 [0.50, 0.71]	0.12 [0.00, 0.30]	0.14 [0.00, 0.42]	1.00 [1.00, 1.00]	0.18 [0.15, 0.21]	0.60 [0.00, 1.00]
lr3e-05_wd0.001_h256_d1	0.76 [0.65, 0.91]	0.48 [0.25, 0.79]	0.24 [0.00, 0.43]	0.56 [0.45, 0.79]	0.09 [-0.08, 0.40]	0.47 [0.00, 1.00]	0.65 [0.00, 1.00]	0.15 [0.00, 0.27]	0.74 [0.00, 1.00]
lr3e-05_wd0.001_h400_d1	0.69 [0.62, 0.86]	0.32 [0.24, 0.42]	0.36 [0.26, 0.46]	0.62 [0.55, 0.72]	0.26 [0.13, 0.35]	0.58 [0.11, 0.95]	0.67 [0.25, 1.00]	0.34 [0.15, 0.50]	0.94 [0.86, 1.00]
lr3e-05_wd0.001_h512_d1	0.76 [0.65, 0.86]	0.44 [0.27, 0.53]	0.32 [0.29, 0.35]	0.60 [0.50, 0.71]	0.17 [0.00, 0.30]	0.43 [0.00, 0.89]	0.77 [0.33, 1.00]	0.23 [0.17, 0.33]	0.75 [0.00, 1.00]
lr3e-05_wd0.001_h256_d2	0.74 [0.66, 0.91]	0.50 [0.28, 0.79]	0.26 [0.00, 0.40]	0.57 [0.47, 0.76]	0.10 [-0.09, 0.36]	0.39 [0.00, 0.95]	0.75 [0.00, 1.00]	0.16 [0.00, 0.25]	0.75 [0.00, 1.00]
lr3e-05_wd0.001_h400_d2	0.79 [0.64, 0.93]	0.49 [0.27, 0.59]	0.40 [0.27, 0.57]	0.66 [0.50, 0.78]	0.25 [0.00, 0.50]	0.44 [0.00, 0.89]	0.88 [0.67, 1.00]	0.28 [0.17, 0.50]	0.76 [0.00, 1.00]
lr3e-05_wd0.001_h512_d2	0.76 [0.68, 0.89]	0.49 [0.28, 0.64]	0.29 [0.00, 0.43]	0.62 [0.50, 0.79]	0.18 [0.00, 0.40]	0.44 [0.00, 1.00]	0.80 [0.00, 1.00]	0.18 [0.00, 0.27]	0.77 [0.00, 1.00]
lr0.0001_wd0.005_h256_d1	0.74 [0.65, 0.88]	0.39 [0.26, 0.63]	0.31 [0.00, 0.50]	0.63 [0.47, 0.84]	0.20 [-0.09, 0.48]	0.56 [0.15, 0.95]	0.70 [0.00, 1.00]	0.21 [0.00, 0.33]	0.95 [0.86, 1.00]
lr0.0001_wd0.005_h400_d1	0.72 [0.61, 0.88]	0.40 [0.26, 0.51]	0.27 [0.00, 0.46]	0.59 [0.50, 0.82]	0.15 [0.00, 0.44]	0.43 [0.05, 1.00]	0.75 [0.00, 1.00]	0.17 [0.00, 0.30]	0.95 [0.86, 1.00]
lr0.0001_wd0.005_h512_d1	0.71 [0.62, 0.89]	0.47 [0.25, 0.78]	0.36 [0.29, 0.46]	0.63 [0.55, 0.72]	0.24 [0.13, 0.35]	0.54 [0.10, 0.95]	0.72 [0.33, 1.00]	0.29 [0.17, 0.50]	0.94 [0.88, 1.00]
lr0.0001_wd0.005_h256_d2	0.76 [0.61, 0.93]	0.46 [0.25, 0.73]	0.21 [0.00, 0.29]	0.51 [0.50, 0.53]	0.02 [0.00, 0.05]	0.42 [0.00, 1.00]	0.60 [0.00, 1.00]	0.14 [0.00, 0.20]	0.51 [0.00, 0.86]
lr0.0001_wd0.005_h400_d2	0.78 [0.64, 0.91]	0.54 [0.28, 0.67]	0.16 [0.00, 0.30]	0.48 [0.40, 0.53]	-0.02 [-0.20, 0.09]	0.37 [0.00, 1.00]	0.60 [0.00, 1.00]	0.10 [0.00, 0.17]	0.53 [0.00, 1.00]
lr0.0001_wd0.005_h512_d2	0.78 [0.62, 0.91]	0.49 [0.28, 0.79]	0.16 [0.00, 0.30]	0.49 [0.45, 0.53]	-0.01 [-0.13, 0.09]	0.39 [0.00, 1.00]	0.60 [0.00, 1.00]	0.10 [0.00, 0.17]	0.54 [0.00, 1.00]
lr0.0001_wd0.01_h256_d1	0.72 [0.64, 0.89]	0.40 [0.27, 0.68]	0.28 [0.00, 0.40]	0.60 [0.45, 0.70]	0.16 [-0.13, 0.32]	0.45 [0.20, 0.89]	0.75 [0.00, 1.00]	0.17 [0.00, 0.25]	0.95 [0.85, 1.00]
lr0.0001_wd0.01_h400_d1	0.69 [0.61, 0.88]	0.36 [0.25, 0.62]	0.30 [0.00, 0.55]	0.61 [0.47, 0.87]	0.17 [-0.09, 0.53]	0.58 [0.25, 0.95]	0.65 [0.00, 1.00]	0.20 [0.00, 0.38]	0.91 [0.83, 1.00]

Supplementary Table 35: Grid search results on the classification task on GDHCM prospective dataset (choosing 60% as the threshold for clinical variables selection). Reported metrics are AUC, AUPRC, F1, Balanced Accuracy, MCC, Specificity, Sensitivity, PPV, and NPV.

Config	AUC	AUPRC	F1	Balanced_Accuracy	MCC	Specificity	Sensitivity	PPV	NPV
lr0.0001_wd0.01_h512_d1	0.69 [0.64, 0.86]	0.40 [0.24, 0.66]	0.39 [0.29, 0.50]	0.64 [0.55, 0.80]	0.29 [0.13, 0.55]	0.47 [0.10, 1.00]	0.82 [0.33, 1.00]	0.38 [0.17, 1.00]	0.96 [0.90, 1.00]
lr0.0001_wd0.01_h256_d2	0.73 [0.61, 0.91]	0.45 [0.23, 0.79]	0.19 [0.00, 0.35]	0.55 [0.42, 0.71]	0.06 [-0.17, 0.30]	0.54 [0.15, 0.95]	0.55 [0.00, 1.00]	0.12 [0.00, 0.21]	0.91 [0.81, 1.00]
lr0.0001_wd0.01_h400_d2	0.76 [0.69, 0.95]	0.49 [0.35, 0.83]	0.22 [0.00, 0.30]	0.53 [0.50, 0.63]	0.05 [0.00, 0.22]	0.41 [0.00, 1.00]	0.65 [0.00, 1.00]	0.14 [0.00, 0.20]	0.54 [0.00, 1.00]
lr0.0001_wd0.01_h512_d2	0.71 [0.64, 0.84]	0.31 [0.27, 0.40]	0.29 [0.00, 0.50]	0.60 [0.50, 0.75]	0.16 [0.00, 0.40]	0.55 [0.00, 1.00]	0.65 [0.00, 1.00]	0.20 [0.00, 0.38]	0.74 [0.00, 1.00]
lr0.0003_wd0.005_h256_d1	0.74 [0.66, 0.96]	0.52 [0.30, 0.81]	0.31 [0.25, 0.40]	0.57 [0.53, 0.64]	0.16 [0.04, 0.34]	0.38 [0.05, 0.95]	0.77 [0.33, 1.00]	0.24 [0.14, 0.50]	0.95 [0.85, 1.00]
lr0.0003_wd0.005_h400_d1	0.73 [0.62, 0.93]	0.48 [0.24, 0.81]	0.31 [0.18, 0.40]	0.59 [0.47, 0.68]	0.18 [-0.04, 0.34]	0.52 [0.26, 0.95]	0.67 [0.25, 1.00]	0.25 [0.14, 0.50]	0.92 [0.82, 1.00]
lr0.0003_wd0.005_h512_d1	0.73 [0.66, 0.89]	0.45 [0.30, 0.63]	0.37 [0.29, 0.46]	0.67 [0.57, 0.82]	0.26 [0.17, 0.44]	0.65 [0.35, 0.90]	0.68 [0.25, 1.00]	0.28 [0.22, 0.33]	0.93 [0.86, 1.00]
lr0.0003_wd0.005_h256_d2	0.76 [0.65, 0.95]	0.53 [0.29, 0.83]	0.32 [0.24, 0.50]	0.55 [0.50, 0.67]	0.15 [0.00, 0.55]	0.28 [0.00, 1.00]	0.82 [0.33, 1.00]	0.33 [0.14, 1.00]	0.55 [0.00, 1.00]
lr0.0003_wd0.005_h400_d2	0.71 [0.64, 0.84]	0.38 [0.27, 0.60]	0.29 [0.00, 0.55]	0.60 [0.45, 0.87]	0.14 [-0.13, 0.53]	0.60 [0.00, 0.89]	0.60 [0.00, 1.00]	0.20 [0.00, 0.38]	0.72 [0.00, 1.00]
lr0.0003_wd0.005_h512_d2	0.74 [0.62, 0.95]	0.52 [0.35, 0.83]	0.33 [0.25, 0.40]	0.59 [0.54, 0.62]	0.26 [0.06, 0.47]	0.66 [0.11, 1.00]	0.53 [0.25, 1.00]	0.52 [0.15, 1.00]	0.90 [0.87, 1.00]
lr0.0003_wd0.01_h256_d1	0.77 [0.68, 0.91]	0.54 [0.36, 0.70]	0.35 [0.22, 0.50]	0.61 [0.42, 0.79]	0.21 [-0.17, 0.55]	0.61 [0.10, 1.00]	0.62 [0.25, 1.00]	0.37 [0.14, 1.00]	0.87 [0.67, 1.00]
lr0.0003_wd0.01_h400_d1	0.73 [0.60, 0.91]	0.44 [0.25, 0.60]	0.36 [0.00, 0.50]	0.65 [0.40, 0.82]	0.28 [-0.20, 0.55]	0.69 [0.30, 1.00]	0.62 [0.00, 1.00]	0.37 [0.00, 1.00]	0.93 [0.80, 1.00]
lr0.0003_wd0.01_h512_d1	0.70 [0.63, 0.86]	0.40 [0.27, 0.51]	0.39 [0.32, 0.50]	0.64 [0.57, 0.70]	0.29 [0.17, 0.55]	0.68 [0.15, 1.00]	0.60 [0.33, 1.00]	0.41 [0.19, 1.00]	0.92 [0.88, 1.00]
lr0.0003_wd0.01_h256_d2	0.75 [0.64, 0.91]	0.47 [0.30, 0.70]	0.25 [0.00, 0.33]	0.57 [0.50, 0.60]	0.13 [0.00, 0.20]	0.43 [0.11, 1.00]	0.70 [0.00, 1.00]	0.16 [0.00, 0.25]	0.95 [0.86, 1.00]
lr0.0003_wd0.01_h400_d2	0.73 [0.60, 0.95]	0.49 [0.23, 0.83]	0.30 [0.24, 0.36]	0.56 [0.50, 0.65]	0.12 [0.00, 0.26]	0.25 [0.00, 0.89]	0.87 [0.33, 1.00]	0.21 [0.14, 0.33]	0.58 [0.00, 1.00]
lr0.0003_wd0.01_h512_d2	0.72 [0.65, 0.93]	0.47 [0.28, 0.81]	0.31 [0.26, 0.33]	0.58 [0.55, 0.61]	0.16 [0.08, 0.27]	0.59 [0.11, 0.95]	0.57 [0.25, 1.00]	0.28 [0.15, 0.50]	0.90 [0.86, 1.00]
lr0.001_wd0.005_h256_d1	0.68 [0.57, 0.88]	0.44 [0.26, 0.71]	0.34 [0.00, 0.50]	0.64 [0.42, 0.82]	0.25 [-0.17, 0.55]	0.76 [0.55, 1.00]	0.52 [0.00, 1.00]	0.37 [0.00, 1.00]	0.90 [0.81, 1.00]
lr0.001_wd0.005_h400_d1	0.72 [0.66, 0.86]	0.46 [0.27, 0.71]	0.28 [0.00, 0.44]	0.59 [0.45, 0.73]	0.14 [-0.13, 0.35]	0.70 [0.25, 0.89]	0.48 [0.00, 1.00]	0.21 [0.00, 0.33]	0.90 [0.84, 1.00]
lr0.001_wd0.005_h512_d1	0.75 [0.64, 0.93]	0.48 [0.30, 0.81]	0.37 [0.29, 0.55]	0.67 [0.57, 0.87]	0.29 [0.17, 0.53]	0.71 [0.40, 0.95]	0.63 [0.25, 1.00]	0.33 [0.20, 0.50]	0.93 [0.86, 1.00]
lr0.001_wd0.005_h256_d2	0.73 [0.61, 0.86]	0.48 [0.23, 0.63]	0.44 [0.36, 0.55]	0.70 [0.62, 0.87]	0.40 [0.26, 0.53]	0.78 [0.30, 1.00]	0.62 [0.25, 1.00]	0.52 [0.22, 1.00]	0.93 [0.87, 1.00]
lr0.001_wd0.005_h400_d2	0.78 [0.71, 0.91]	0.53 [0.36, 0.72]	0.46 [0.30, 0.57]	0.68 [0.55, 0.78]	0.37 [0.08, 0.55]	0.77 [0.35, 1.00]	0.60 [0.33, 0.75]	0.49 [0.19, 1.00]	0.91 [0.88, 0.94]
lr0.001_wd0.005_h512_d2	0.74 [0.69, 0.82]	0.46 [0.29, 0.72]	0.50 [0.29, 0.80]	0.72 [0.57, 0.89]	0.41 [0.17, 0.80]	0.81 [0.50, 1.00]	0.63 [0.25, 1.00]	0.48 [0.23, 1.00]	0.92 [0.86, 1.00]
lr0.001_wd0.01_h256_d1	0.72 [0.65, 0.86]	0.43 [0.28, 0.71]	0.32 [0.00, 0.50]	0.61 [0.45, 0.68]	0.25 [-0.13, 0.55]	0.76 [0.35, 1.00]	0.47 [0.00, 1.00]	0.40 [0.00, 1.00]	0.91 [0.85, 1.00]
lr0.001_wd0.01_h400_d1	0.74 [0.62, 0.86]	0.38 [0.27, 0.50]	0.27 [0.18, 0.38]	0.57 [0.47, 0.68]	0.13 [-0.04, 0.29]	0.58 [0.26, 0.90]	0.57 [0.25, 1.00]	0.21 [0.14, 0.33]	0.91 [0.82, 1.00]
lr0.001_wd0.01_h512_d1	0.75 [0.65, 0.89]	0.55 [0.33, 0.71]	0.40 [0.25, 0.60]	0.66 [0.55, 0.89]	0.31 [0.10, 0.58]	0.76 [0.45, 1.00]	0.57 [0.25, 1.00]	0.43 [0.21, 1.00]	0.91 [0.85, 1.00]
lr0.001_wd0.01_h256_d2	0.73 [0.66, 0.88]	0.51 [0.33, 0.77]	0.36 [0.00, 0.60]	0.68 [0.50, 0.80]	0.30 [0.00, 0.52]	0.78 [0.47, 1.00]	0.57 [0.00, 1.00]	0.31 [0.00, 0.50]	0.92 [0.83, 1.00]
lr0.001_wd0.01_h400_d2	0.75 [0.67, 0.95]	0.46 [0.31, 0.64]	0.46 [0.35, 0.67]	0.71 [0.62, 0.81]	0.35 [0.19, 0.61]	0.70 [0.50, 0.95]	0.72 [0.67, 0.75]	0.37 [0.23, 0.67]	0.93 [0.91, 0.95]
lr0.001_wd0.01_h512_d2	0.71 [0.64, 0.89]	0.43 [0.28, 0.60]	0.22 [0.00, 0.57]	0.57 [0.47, 0.78]	0.10 [-0.09, 0.50]	0.76 [0.45, 1.00]	0.37 [0.00, 0.67]	0.17 [0.00, 0.50]	0.87 [0.82, 0.94]
lr0.0001_wd0.0_h1024_d1	0.73 [0.64, 0.93]	0.43 [0.28, 0.72]	0.28 [0.00, 0.46]	0.60 [0.47, 0.82]	0.15 [-0.09, 0.44]	0.61 [0.10, 0.95]	0.60 [0.00, 1.00]	0.19 [0.00, 0.30]	0.92 [0.86, 1.00]
lr0.0001_wd0.0_h2048_d1	0.71 [0.65, 0.88]	0.43 [0.28, 0.61]	0.19 [0.00, 0.40]	0.53 [0.42, 0.64]	0.05 [-0.17, 0.34]	0.84 [0.70, 0.95]	0.22 [0.00, 0.50]	0.19 [0.00, 0.50]	0.86 [0.81, 0.90]
lr0.0001_wd0.0_h1024_d2	0.75 [0.66, 0.91]	0.39 [0.26, 0.70]	0.23 [0.00, 0.32]	0.52 [0.50, 0.57]	0.05 [0.00, 0.17]	0.24 [0.00, 1.00]	0.80 [0.00, 1.00]	0.13 [0.00, 0.19]	0.57 [0.00, 1.00]
lr0.0001_wd0.0_h2048_d2	0.73 [0.61, 0.91]	0.45 [0.28, 0.67]	0.23 [0.00, 0.32]	0.54 [0.50, 0.57]	0.09 [0.00, 0.17]	0.48 [0.05, 1.00]	0.60 [0.00, 1.00]	0.17 [0.00, 0.33]	0.92 [0.86, 1.00]
lr0.0001_wd0.0001_h1024_d1	0.71 [0.62, 0.88]	0.40 [0.26, 0.49]	0.37 [0.20, 0.60]	0.66 [0.50, 0.89]	0.25 [0.00, 0.58]	0.63 [0.20, 0.79]	0.68 [0.25, 1.00]	0.27 [0.17, 0.43]	0.93 [0.83, 1.00]
lr0.0001_wd0.0001_h2048_d1	0.74 [0.60, 0.95]	0.43 [0.34, 0.64]	0.26 [0.00, 0.43]	0.59 [0.47, 0.70]	0.13 [-0.04, 0.30]	0.62 [0.32, 1.00]	0.55 [0.00, 1.00]	0.18 [0.00, 0.30]	0.90 [0.82, 1.00]
lr0.0001_wd0.0001_h1024_d2	0.78 [0.70, 0.98]	0.56 [0.33, 0.92]	0.31 [0.24, 0.36]	0.58 [0.50, 0.68]	0.15 [0.00, 0.27]	0.38 [0.00, 0.95]	0.78 [0.25, 1.00]	0.25 [0.14, 0.50]	0.56 [0.00, 1.00]
lr0.0001_wd0.0001_h2048_d2	0.73 [0.65, 0.91]	0.48 [0.29, 0.79]	0.27 [0.00, 0.55]	0.63 [0.45, 0.82]	0.20 [-0.13, 0.45]	0.86 [0.63, 1.00]	0.40 [0.00, 1.00]	0.25 [0.00, 0.50]	0.90 [0.82, 1.00]
lr0.0001_wd0.001_h1024_d1	0.73 [0.62, 0.93]	0.39 [0.24, 0.72]	0.23 [0.00, 0.43]	0.59 [0.42, 0.79]	0.10 [-0.17, 0.40]	0.72 [0.58, 0.89]	0.45 [0.00, 1.00]	0.16 [0.00, 0.27]	0.89 [0.81, 1.00]
lr0.0001_wd0.001_h2048_d1	0.70 [0.65, 0.86]	0.39 [0.26, 0.49]	0.35 [0.20, 0.50]	0.61 [0.50, 0.67]	0.24 [0.00, 0.55]	0.60 [0.15, 1.00]	0.62 [0.25, 1.00]	0.38 [0.17, 1.00]	0.93 [0.83, 1.00]
lr0.0001_wd0.001_h1024_d2	0.74 [0.65, 0.88]	0.36 [0.30, 0.47]	0.28 [0.00, 0.43]	0.60 [0.50, 0.79]	0.15 [0.00, 0.40]	0.51 [0.00, 1.00]	0.70 [0.00, 1.00]	0.18 [0.00, 0.27]	0.75 [0.00, 1.00]
lr0.0001_wd0.001_h2048_d2	0.74 [0.66, 0.88]	0.42 [0.31, 0.57]	0.30 [0.25, 0.40]	0.56 [0.50, 0.64]	0.13 [0.00, 0.34]	0.51 [0.00, 0.95]	0.62 [0.25, 1.00]	0.25 [0.17, 0.50]	0.72 [0.00, 1.00]
lr0.0003_wd0.0_h1024_d1	0.72 [0.67, 0.89]	0.47 [0.31, 0.61]	0.41 [0.29, 0.60]	0.67 [0.53, 0.89]	0.27 [0.04, 0.58]	0.67 [0.30, 0.85]	0.68 [0.50, 1.00]	0.30 [0.18, 0.43]	0.91 [0.86, 1.00]
lr0.0003_wd0.0_h2048_d1	0.72 [0.65, 0.88]	0.41 [0.32, 0.45]	0.34 [0.29, 0.43]	0.64 [0.57, 0.79]	0.24 [0.17, 0.40]	0.80 [0.58, 0.90]	0.48 [0.25, 1.00]	0.31 [0.27, 0.33]	0.90 [0.86, 1.00]
lr0.0003_wd0.0_h1024_d2	0.73 [0.67, 0.88]	0.50 [0.32, 0.77]	0.35 [0.29, 0.50]	0.62 [0.57, 0.68]	0.26 [0.12, 0.55]	0.62 [0.20, 1.00]	0.62 [0.25, 1.00]	0.39 [0.20, 1.00]	0.93 [0.86, 1.00]
lr0.0003_wd0.0_h2048_d2	0.74 [0.66, 0.86]	0.43 [0.31, 0.57]	0.39 [0.25, 0.57]	0.66 [0.55, 0.78]	0.28 [0.10, 0.50]	0.85 [0.79, 0.90]	0.47 [0.25, 0.67]	0.35 [0.25, 0.50]	0.90 [0.85, 0.94]
lr0.0003_wd0.0001_h1024_d1	0.76 [0.70, 0.84]	0.52 [0.35, 0.63]	0.40 [0.35, 0.44]	0.67 [0.62, 0.73]	0.29 [0.19, 0.35]	0.65 [0.35, 0.95]	0.70 [0.33, 1.00]	0.32 [0.23, 0.50]	0.94 [0.90, 1.00]
lr0.0003_wd0.0001_h2048_d1	0.75 [0.64, 0.95]	0.47 [0.29, 0.83]	0.33 [0.22, 0.50]	0.61 [0.54, 0.75]	0.18 [0.05, 0.42]	0.73 [0.55, 0.85]	0.50 [0.25, 0.75]	0.26 [0.17, 0.40]	0.89 [0.85, 0.94]
lr0.0003_wd0.0001_h1024_d2	0.73 [0.60, 0.91]	0.46 [0.25, 0.62]	0.32 [0.00, 0.75]	0.64 [0.45, 0.95]	0.26 [-0.13, 0.73]	0.81 [0.30, 1.00]	0.47 [0.00, 1.00]	0.36 [0.00, 1.00]	0.91 [0.82, 1.00]
lr0.0003_wd0.0001_h2048_d2	0.73 [0.62, 0.89]	0.43 [0.25, 0.55]	0.21 [0.00, 0.50]	0.54 [0.45, 0.67]	0.12 [-0.13, 0.55]	0.91 [0.75, 1.00]	0.17 [0.00, 0.33]	0.33 [0.00, 1.00]	0.86 [0.83, 0.90]
lr0.0003_wd0.001_h1024_d1	0.71 [0.62, 0.84]	0.44 [0.33, 0.55]	0.22 [0.00, 0.50]	0.55 [0.45, 0.67]	0.15 [-0.13, 0.55]	0.93 [0.89, 1.00]	0.17 [0.00, 0.33]	0.37 [0.00, 1.00]	0.86 [0.82, 0.90]
lr0.0003_wd0.001_h2048_d1	0.70 [0.62, 0.82]	0.42 [0.31, 0.50]	0.29 [0.00, 0.44]	0.59 [0.45, 0.68]	0.17 [-0.13, 0.32]	0.86 [0.75, 0.95]	0.32 [0.00, 0.50]	0.29 [0.00, 0.50]	0.88 [0.85, 0.89]
lr0.0003_wd0.001_h1024_d2	0.72 [0.64, 0.89]	0.47 [0.26, 0.64]	0.33 [0.00, 0.55]	0.62 [0.42, 0.87]	0.24 [-0.17, 0.55]	0.82 [0.55, 1.00]	0.42 [0.00, 1.00]	0.41 [0.00, 1.00]	0.88 [0.81, 1.00]
lr0.0003_wd0.001_h2048_d2	0.74 [0.65, 0.89]	0.47 [0.32, 0.60]	0.37 [0.00, 0.60]	0.66 [0.47, 0.89]	0.31 [-0.09, 0.58]	0.90 [0.79, 1.00]	0.42 [0.00, 1.00]	0.43 [0.00, 1.00]	0.90 [0.83, 1.00]
lr0.001_wd0.0_h1024_d1	0.73 [0.64, 0.81]	0.45 [0.30, 0.73]	0.34 [0.00, 0.55]	0.63 [0.42, 0.87]	0.27 [-0.17, 0.55]	0.80 [0.45, 1.00]	0.47 [0.00, 1.00]	0.42 [0.00, 1.00]	0.90 [0.81, 1.00]
lr0.001_wd0.0_h2048_d1	0.72 [0.61, 0.93]	0.45 [0.30, 0.59]	0.25 [0.00, 0.38]	0.59 [0.47, 0.74]	0.13 [-0.09, 0.33]	0.75 [0.47, 0.95]	0.42 [0.00, 1.00]	0.20 [0.00, 0.33]	0.89 [0.83, 1.00]
lr0.001_wd0.0_h1024_d2	0.76 [0.68, 0.89]	0.51 [0.37, 0.67]	0.44 [0.25, 0.75]	0.70 [0.55, 0.95]	0.34 [0.10, 0.73]	0.86 [0.79, 0.90]	0.53 [0.25, 1.00]	0.39 [0.25, 0.60]	0.91 [0.85, 1.00]
lr0.001_wd0.0_h2048_d2	0.75 [0.68, 0.88]	0.42 [0.32, 0.55]	0.26 [0.00, 0.40]	0.58 [0.47, 0.70]	0.13 [-0.09, 0.32]	0.69 [0.30, 0.95]	0.47 [0.00, 1.00]	0.22 [0.00, 0.33]	0.89 [0.86, 1.00]
lr0.001_wd0.0001_h1024_d1	0.75 [0.66, 0.89]	0.51 [0.26, 0.72]	0.37 [0.00, 0.67]	0.65 [0.40, 0.81]	0.25 [-0.20, 0.61]	0.71 [0.45, 0.95]	0.58 [0.00, 1.00]	0.31 [0.00, 0.67]	0.91 [0.80, 1.00]
lr0.001_wd0.0001_h2048_d1	0.77 [0.68, 0.93]	0.46 [0.34, 0.59]	0.40 [0.25, 0.50]	0.67 [0.55, 0.84]	0.32 [0.10, 0.55]	0.72 [0.30, 1.00]	0.62 [0.25, 1.00]	0.42 [0.22, 1.00]	0.93 [0.85, 1.00]
lr0.001_wd0.0001_h1024_d2	0.75 [0.61, 0.86]	0.48 [0.23, 0.70]	0.48 [0.35, 0.67]	0.72 [0.62, 0.92]	0.39 [0.19, 0.65]	0.77 [0.50, 0.95]	0.67 [0.33, 1.00]	0.43 [0.23, 0.67]	0.93 [0.90, 1.00]

Supplementary Table 36: Grid search results on the classification task on GDHCM prospective dataset (choosing 60% as the threshold for clinical variables selection). Reported metrics are AUC, AUPRC, F1, Balanced Accuracy, MCC, Specificity, Sensitivity, PPV, and NPV.

Config	AUC	AUPRC	F1	Balanced_Accuracy	MCC	Specificity	Sensitivity	PPV	NPV
lr0.001_wd0.0001_h2048_d2	0.75 [0.68, 0.84]	0.48 [0.34, 0.72]	0.49 [0.36, 0.67]	0.73 [0.62, 0.82]	0.39 [0.20, 0.60]	0.84 [0.75, 0.90]	0.62 [0.50, 0.75]	0.42 [0.29, 0.60]	0.92 [0.88, 0.95]
lr0.001_wd0.001_h1024_d1	0.71 [0.62, 0.91]	0.52 [0.27, 0.79]	0.30 [0.00, 0.44]	0.61 [0.45, 0.73]	0.18 [-0.13, 0.35]	0.82 [0.65, 0.95]	0.40 [0.00, 0.75]	0.27 [0.00, 0.50]	0.89 [0.82, 0.94]
lr0.001_wd0.001_h2048_d1	0.75 [0.67, 0.89]	0.51 [0.30, 0.64]	0.46 [0.25, 0.75]	0.71 [0.55, 0.95]	0.39 [0.10, 0.73]	0.75 [0.40, 1.00]	0.67 [0.25, 1.00]	0.47 [0.25, 1.00]	0.94 [0.85, 1.00]
lr0.001_wd0.001_h1024_d2	0.74 [0.60, 0.89]	0.43 [0.25, 0.55]	0.39 [0.00, 0.75]	0.67 [0.42, 0.95]	0.32 [-0.17, 0.73]	0.82 [0.60, 1.00]	0.53 [0.00, 1.00]	0.43 [0.00, 1.00]	0.91 [0.81, 1.00]
lr0.001_wd0.001_h2048_d2	0.72 [0.62, 0.89]	0.42 [0.30, 0.53]	0.37 [0.22, 0.57]	0.64 [0.53, 0.78]	0.24 [0.05, 0.50]	0.82 [0.74, 0.89]	0.47 [0.25, 0.67]	0.31 [0.20, 0.50]	0.89 [0.84, 0.94]
lr0.0001_wd0.0_h256_d3	0.78 [0.65, 0.93]	0.60 [0.30, 0.81]	0.22 [0.00, 0.29]	0.50 [0.50, 0.50]	0.00 [0.00, 0.00]	0.20 [0.00, 1.00]	0.80 [0.00, 1.00]	0.13 [0.00, 0.17]	0.17 [0.00, 0.86]
lr0.0001_wd0.0_h400_d3	0.75 [0.63, 0.89]	0.53 [0.33, 0.78]	0.23 [0.00, 0.35]	0.53 [0.50, 0.63]	0.05 [0.00, 0.23]	0.25 [0.00, 1.00]	0.80 [0.00, 1.00]	0.14 [0.00, 0.21]	0.37 [0.00, 1.00]
lr0.0001_wd0.0_h512_d3	0.80 [0.66, 0.89]	0.54 [0.30, 0.78]	0.28 [0.00, 0.50]	0.59 [0.50, 0.84]	0.13 [0.00, 0.48]	0.44 [0.00, 1.00]	0.75 [0.00, 1.00]	0.18 [0.00, 0.33]	0.55 [0.00, 1.00]
lr0.0001_wd0.0_h256_d4	0.78 [0.66, 0.91]	0.54 [0.39, 0.70]	0.25 [0.00, 0.38]	0.56 [0.50, 0.74]	0.10 [0.00, 0.33]	0.47 [0.00, 1.00]	0.65 [0.00, 1.00]	0.18 [0.00, 0.33]	0.54 [0.00, 1.00]
lr0.0001_wd0.0_h400_d4	0.75 [0.63, 0.93]	0.50 [0.24, 0.81]	0.25 [0.00, 0.35]	0.58 [0.50, 0.71]	0.13 [0.00, 0.30]	0.50 [0.00, 1.00]	0.65 [0.00, 1.00]	0.18 [0.00, 0.33]	0.74 [0.00, 1.00]
lr0.0001_wd0.0_h512_d4	0.80 [0.69, 0.98]	0.52 [0.29, 0.92]	0.22 [0.00, 0.29]	0.50 [0.50, 0.50]	0.00 [0.00, 0.00]	0.20 [0.00, 1.00]	0.80 [0.00, 1.00]	0.13 [0.00, 0.17]	0.17 [0.00, 0.86]
lr0.0001_wd0.0001_h256_d3	0.75 [0.65, 0.91]	0.52 [0.30, 0.79]	0.23 [0.00, 0.35]	0.53 [0.50, 0.63]	0.05 [0.00, 0.23]	0.25 [0.00, 1.00]	0.80 [0.00, 1.00]	0.14 [0.00, 0.21]	0.37 [0.00, 1.00]
lr0.0001_wd0.0001_h400_d3	0.76 [0.66, 0.89]	0.48 [0.40, 0.64]	0.24 [0.00, 0.38]	0.53 [0.50, 0.65]	0.04 [0.00, 0.22]	0.31 [0.00, 1.00]	0.75 [0.00, 1.00]	0.14 [0.00, 0.25]	0.36 [0.00, 0.92]
lr0.0001_wd0.0001_h512_d3	0.76 [0.60, 0.93]	0.45 [0.25, 0.81]	0.17 [0.00, 0.35]	0.52 [0.45, 0.63]	0.02 [-0.13, 0.23]	0.43 [0.00, 1.00]	0.60 [0.00, 1.00]	0.10 [0.00, 0.21]	0.54 [0.00, 1.00]
lr0.0001_wd0.0001_h256_d4	0.78 [0.68, 0.93]	0.52 [0.32, 0.82]	0.23 [0.00, 0.33]	0.52 [0.50, 0.60]	0.03 [0.00, 0.15]	0.29 [0.00, 1.00]	0.75 [0.00, 1.00]	0.14 [0.00, 0.21]	0.35 [0.00, 0.90]
lr0.0001_wd0.0001_h400_d4	0.76 [0.64, 0.89]	0.42 [0.26, 0.64]	0.22 [0.00, 0.55]	0.57 [0.50, 0.87]	0.11 [0.00, 0.53]	0.55 [0.00, 1.00]	0.60 [0.00, 1.00]	0.14 [0.00, 0.38]	0.54 [0.00, 1.00]
lr0.0001_wd0.0001_h512_d4	0.81 [0.63, 0.98]	0.51 [0.29, 0.92]	0.27 [0.00, 0.46]	0.57 [0.50, 0.82]	0.11 [0.00, 0.44]	0.34 [0.00, 1.00]	0.80 [0.00, 1.00]	0.16 [0.00, 0.30]	0.57 [0.00, 1.00]
lr0.0001_wd0.001_h256_d3	0.76 [0.61, 0.89]	0.41 [0.26, 0.57]	0.26 [0.00, 0.40]	0.58 [0.50, 0.76]	0.13 [0.00, 0.36]	0.36 [0.00, 1.00]	0.80 [0.00, 1.00]	0.16 [0.00, 0.25]	0.77 [0.00, 1.00]
lr0.0001_wd0.001_h400_d3	0.77 [0.64, 0.91]	0.51 [0.24, 0.79]	0.27 [0.00, 0.46]	0.59 [0.50, 0.82]	0.14 [0.00, 0.44]	0.48 [0.00, 1.00]	0.70 [0.00, 1.00]	0.17 [0.00, 0.30]	0.74 [0.00, 1.00]
lr0.0001_wd0.001_h512_d3	0.80 [0.70, 0.93]	0.56 [0.34, 0.81]	0.22 [0.00, 0.29]	0.50 [0.50, 0.50]	0.00 [0.00, 0.00]	0.20 [0.00, 1.00]	0.80 [0.00, 1.00]	0.13 [0.00, 0.17]	0.17 [0.00, 0.86]
lr0.0001_wd0.001_h256_d4	0.75 [0.63, 0.91]	0.50 [0.24, 0.79]	0.27 [0.00, 0.50]	0.60 [0.50, 0.84]	0.16 [0.00, 0.48]	0.56 [0.00, 1.00]	0.65 [0.00, 1.00]	0.18 [0.00, 0.33]	0.74 [0.00, 1.00]
lr0.0001_wd0.001_h400_d4	0.76 [0.63, 0.93]	0.49 [0.24, 0.81]	0.30 [0.00, 0.50]	0.58 [0.45, 0.78]	0.17 [-0.13, 0.55]	0.49 [0.00, 1.00]	0.67 [0.00, 1.00]	0.32 [0.00, 1.00]	0.54 [0.00, 1.00]
lr0.0001_wd0.001_h512_d4	0.78 [0.64, 0.95]	0.54 [0.33, 0.83]	0.22 [0.00, 0.29]	0.50 [0.50, 0.50]	0.00 [0.00, 0.00]	0.20 [0.00, 1.00]	0.80 [0.00, 1.00]	0.13 [0.00, 0.17]	0.17 [0.00, 0.86]
lr0.0003_wd0.0_h256_d3	0.75 [0.64, 0.91]	0.48 [0.33, 0.79]	0.27 [0.00, 0.40]	0.58 [0.50, 0.76]	0.12 [0.00, 0.36]	0.37 [0.00, 1.00]	0.80 [0.00, 1.00]	0.16 [0.00, 0.25]	0.57 [0.00, 1.00]
lr0.0003_wd0.0_h400_d3	0.75 [0.66, 0.89]	0.40 [0.32, 0.49]	0.19 [0.00, 0.38]	0.53 [0.45, 0.65]	0.05 [-0.13, 0.22]	0.67 [0.00, 1.00]	0.40 [0.00, 1.00]	0.15 [0.00, 0.33]	0.70 [0.00, 0.92]
lr0.0003_wd0.0_h512_d3	0.74 [0.65, 0.89]	0.39 [0.27, 0.53]	0.29 [0.00, 0.55]	0.60 [0.50, 0.87]	0.17 [0.00, 0.53]	0.41 [0.00, 1.00]	0.80 [0.00, 1.00]	0.18 [0.00, 0.38]	0.77 [0.00, 1.00]
lr0.0003_wd0.0_h256_d4	0.75 [0.66, 0.91]	0.39 [0.32, 0.56]	0.32 [0.00, 0.55]	0.61 [0.50, 0.87]	0.19 [0.00, 0.53]	0.53 [0.00, 1.00]	0.70 [0.00, 1.00]	0.24 [0.00, 0.50]	0.55 [0.00, 1.00]
lr0.0003_wd0.0_h400_d4	0.79 [0.68, 1.00]	0.59 [0.27, 1.00]	0.24 [0.00, 0.60]	0.56 [0.40, 0.89]	0.09 [-0.20, 0.58]	0.53 [0.00, 1.00]	0.60 [0.00, 1.00]	0.15 [0.00, 0.43]	0.73 [0.00, 1.00]
lr0.0003_wd0.0_h512_d4	0.78 [0.70, 0.82]	0.49 [0.32, 0.74]	0.23 [0.00, 0.35]	0.53 [0.50, 0.63]	0.04 [0.00, 0.19]	0.30 [0.00, 1.00]	0.75 [0.00, 1.00]	0.14 [0.00, 0.23]	0.35 [0.00, 0.91]
lr0.0003_wd0.0001_h256_d3	0.75 [0.68, 0.88]	0.42 [0.33, 0.57]	0.23 [0.00, 0.43]	0.59 [0.45, 0.79]	0.11 [-0.13, 0.40]	0.63 [0.00, 1.00]	0.55 [0.00, 1.00]	0.15 [0.00, 0.30]	0.72 [0.00, 1.00]
lr0.0003_wd0.0001_h400_d3	0.78 [0.70, 0.86]	0.51 [0.32, 0.65]	0.28 [0.00, 0.38]	0.60 [0.50, 0.74]	0.16 [0.00, 0.33]	0.50 [0.00, 1.00]	0.70 [0.00, 1.00]	0.18 [0.00, 0.29]	0.75 [0.00, 1.00]
lr0.0003_wd0.0001_h512_d3	0.73 [0.56, 0.96]	0.52 [0.25, 0.87]	0.34 [0.24, 0.50]	0.57 [0.50, 0.70]	0.17 [0.00, 0.55]	0.28 [0.00, 1.00]	0.87 [0.33, 1.00]	0.34 [0.14, 1.00]	0.38 [0.00, 1.00]
lr0.0003_wd0.0001_h256_d4	0.80 [0.68, 0.93]	0.53 [0.26, 0.81]	0.18 [0.00, 0.32]	0.53 [0.45, 0.61]	0.04 [-0.13, 0.19]	0.45 [0.00, 1.00]	0.60 [0.00, 1.00]	0.10 [0.00, 0.19]	0.74 [0.00, 1.00]
lr0.0003_wd0.0001_h400_d4	0.76 [0.68, 0.88]	0.51 [0.31, 0.68]	0.27 [0.00, 0.40]	0.55 [0.50, 0.65]	0.14 [0.00, 0.47]	0.56 [0.00, 1.00]	0.55 [0.00, 1.00]	0.33 [0.00, 1.00]	0.52 [0.00, 0.89]
lr0.0003_wd0.0001_h512_d4	0.78 [0.65, 0.89]	0.56 [0.26, 0.78]	0.32 [0.00, 0.57]	0.61 [0.50, 0.73]	0.21 [0.00, 0.51]	0.73 [0.00, 1.00]	0.48 [0.00, 1.00]	0.30 [0.00, 0.67]	0.71 [0.00, 0.94]
lr0.0003_wd0.001_h256_d3	0.77 [0.63, 0.96]	0.49 [0.28, 0.81]	0.24 [0.00, 0.46]	0.55 [0.50, 0.73]	0.08 [0.00, 0.35]	0.50 [0.00, 1.00]	0.60 [0.00, 1.00]	0.17 [0.00, 0.33]	0.53 [0.00, 0.93]
lr0.0003_wd0.001_h400_d3	0.74 [0.67, 0.88]	0.50 [0.34, 0.62]	0.36 [0.29, 0.50]	0.61 [0.50, 0.68]	0.23 [0.00, 0.55]	0.50 [0.00, 1.00]	0.72 [0.33, 1.00]	0.38 [0.17, 1.00]	0.73 [0.00, 1.00]
lr0.0003_wd0.001_h512_d3	0.75 [0.65, 0.95]	0.42 [0.28, 0.64]	0.31 [0.00, 0.75]	0.60 [0.50, 0.95]	0.17 [0.00, 0.73]	0.55 [0.00, 1.00]	0.65 [0.00, 1.00]	0.24 [0.00, 0.60]	0.54 [0.00, 1.00]
lr0.0003_wd0.001_h256_d4	0.73 [0.59, 1.00]	0.46 [0.25, 1.00]	0.34 [0.00, 0.86]	0.59 [0.50, 0.97]	0.17 [0.00, 0.84]	0.39 [0.00, 1.00]	0.80 [0.00, 1.00]	0.25 [0.00, 0.75]	0.37 [0.00, 1.00]
lr0.0003_wd0.001_h400_d4	0.75 [0.67, 0.88]	0.48 [0.28, 0.71]	0.22 [0.00, 0.32]	0.54 [0.50, 0.59]	0.07 [0.00, 0.17]	0.56 [0.00, 1.00]	0.52 [0.00, 1.00]	0.16 [0.00, 0.25]	0.72 [0.00, 1.00]
lr0.0003_wd0.001_h512_d4	0.80 [0.71, 0.96]	0.51 [0.33, 0.87]	0.19 [0.00, 0.38]	0.54 [0.45, 0.74]	0.04 [-0.13, 0.33]	0.47 [0.00, 1.00]	0.60 [0.00, 1.00]	0.11 [0.00, 0.23]	0.54 [0.00, 1.00]
lr0.001_wd0.0_h256_d3	0.75 [0.71, 0.86]	0.54 [0.35, 0.61]	0.36 [0.00, 0.50]	0.66 [0.50, 0.79]	0.31 [0.00, 0.55]	0.81 [0.55, 1.00]	0.52 [0.00, 1.00]	0.40 [0.00, 1.00]	0.91 [0.83, 1.00]
lr0.001_wd0.0_h400_d3	0.76 [0.68, 0.86]	0.64 [0.52, 0.76]	0.38 [0.32, 0.50]	0.64 [0.60, 0.67]	0.30 [0.20, 0.55]	0.52 [0.20, 1.00]	0.77 [0.33, 1.00]	0.39 [0.19, 1.00]	0.96 [0.89, 1.00]
lr0.001_wd0.0_h512_d3	0.75 [0.67, 0.96]	0.55 [0.37, 0.87]	0.42 [0.25, 0.60]	0.69 [0.55, 0.89]	0.31 [0.10, 0.58]	0.81 [0.70, 0.95]	0.57 [0.25, 1.00]	0.36 [0.25, 0.50]	0.91 [0.85, 1.00]
lr0.001_wd0.0_h256_d4	0.77 [0.68, 0.93]	0.57 [0.31, 0.81]	0.25 [0.00, 0.43]	0.55 [0.50, 0.70]	0.09 [0.00, 0.30]	0.36 [0.00, 1.00]	0.75 [0.00, 1.00]	0.16 [0.00, 0.30]	0.56 [0.00, 1.00]
lr0.001_wd0.0_h400_d4	0.77 [0.69, 0.98]	0.61 [0.40, 0.92]	0.43 [0.25, 0.67]	0.70 [0.56, 0.92]	0.33 [0.10, 0.65]	0.83 [0.75, 0.90]	0.57 [0.25, 1.00]	0.36 [0.20, 0.50]	0.91 [0.86, 1.00]
lr0.001_wd0.0_h512_d4	0.79 [0.71, 0.98]	0.52 [0.29, 0.92]	0.36 [0.00, 0.75]	0.64 [0.47, 0.95]	0.25 [-0.09, 0.73]	0.73 [0.00, 0.95]	0.55 [0.00, 1.00]	0.33 [0.00, 0.60]	0.72 [0.00, 1.00]
lr0.001_wd0.0001_h256_d3	0.77 [0.67, 0.95]	0.47 [0.31, 0.64]	0.44 [0.30, 0.67]	0.68 [0.53, 0.92]	0.33 [0.09, 0.65]	0.70 [0.05, 0.90]	0.67 [0.33, 1.00]	0.37 [0.17, 0.50]	0.94 [0.89, 1.00]
lr0.001_wd0.0001_h400_d3	0.76 [0.68, 0.91]	0.52 [0.43, 0.66]	0.39 [0.22, 0.57]	0.66 [0.53, 0.79]	0.28 [0.05, 0.51]	0.74 [0.58, 0.95]	0.58 [0.25, 1.00]	0.33 [0.20, 0.67]	0.91 [0.84, 1.00]
lr0.001_wd0.0001_h512_d3	0.77 [0.71, 0.95]	0.55 [0.29, 0.72]	0.41 [0.25, 0.67]	0.67 [0.53, 0.92]	0.31 [0.09, 0.65]	0.71 [0.05, 0.95]	0.63 [0.25, 1.00]	0.36 [0.17, 0.50]	0.93 [0.85, 1.00]
lr0.001_wd0.0001_h256_d4	0.75 [0.63, 0.96]	0.50 [0.33, 0.87]	0.24 [0.00, 0.44]	0.54 [0.48, 0.68]	0.06 [-0.09, 0.32]	0.54 [0.00, 0.95]	0.53 [0.00, 1.00]	0.17 [0.00, 0.40]	0.69 [0.00, 0.89]
lr0.001_wd0.0001_h400_d4	0.78 [0.68, 0.98]	0.56 [0.38, 0.92]	0.45 [0.29, 0.55]	0.70 [0.50, 0.82]	0.36 [0.00, 0.55]	0.63 [0.00, 1.00]	0.77 [0.33, 1.00]	0.45 [0.17, 1.00]	0.76 [0.00, 1.00]
lr0.001_wd0.0001_h512_d4	0.79 [0.65, 1.00]	0.53 [0.35, 1.00]	0.36 [0.25, 0.60]	0.63 [0.50, 0.89]	0.24 [0.00, 0.58]	0.70 [0.00, 0.95]	0.57 [0.25, 1.00]	0.34 [0.17, 0.50]	0.72 [0.00, 1.00]
lr0.001_wd0.001_h256_d3	0.75 [0.68, 0.93]	0.50 [0.29, 0.64]	0.40 [0.29, 0.55]	0.67 [0.50, 0.87]	0.26 [0.00, 0.53]	0.53 [0.00, 0.85]	0.80 [0.50, 1.00]	0.29 [0.17, 0.40]	0.76 [0.00, 1.00]
lr0.001_wd0.001_h400_d3	0.75 [0.68, 0.93]	0.55 [0.33, 0.72]	0.45 [0.30, 0.67]	0.68 [0.55, 0.81]	0.32 [0.08, 0.61]	0.59 [0.10, 0.95]	0.77 [0.67, 1.00]	0.35 [0.18, 0.67]	0.94 [0.88, 1.00]
lr0.001_wd0.001_h512_d3	0.80 [0.70, 0.95]	0.51 [0.32, 0.64]	0.30 [0.00, 0.50]	0.61 [0.47, 0.73]	0.18 [-0.09, 0.40]	0.75 [0.45, 0.95]	0.47 [0.00, 1.00]	0.24 [0.00, 0.50]	0.90 [0.86, 1.00]
lr0.001_wd0.001_h256_d4	0.81 [0.75, 0.88]	0.52 [0.44, 0.66]	0.26 [0.00, 0.50]	0.59 [0.48, 0.75]	0.14 [-0.09, 0.42]	0.74 [0.00, 1.00]	0.43 [0.00, 1.00]	0.21 [0.00, 0.50]	0.71 [0.00, 0.94]
lr0.001_wd0.001_h400_d4	0.81 [0.74, 0.95]	0.49 [0.41, 0.64]	0.52 [0.40, 0.75]	0.76 [0.64, 0.95]	0.45 [0.30, 0.73]	0.76 [0.45, 0.95]	0.77 [0.33, 1.00]	0.43 [0.27, 0.60]	0.95 [0.90, 1.00]
lr0.001_wd0.001_h512_d4	0.80 [0.66, 0.93]	0.60 [0.41, 0.81]	0.28 [0.00, 0.46]	0.59 [0.50, 0.73]	0.13 [0.00, 0.35]	0.47 [0.00, 1.00]	0.70 [0.00, 1.00]	0.19 [0.00, 0.33]	0.55 [0.00, 0.93]

Supplementary Table 37: Grid search results on the classification task on GDHCM prospective dataset (choosing 60% as the threshold for clinical variables selection). Reported metrics are AUC, AUPRC, F1, Balanced Accuracy, MCC, Specificity, Sensitivity, PPV, and NPV.

Config	AUC	AUPRC	F1	Balanced_Accuracy	MCC	Specificity	Sensitivity	PPV	NPV
lr0.0001_wd0.0_h256_d1	0.64[0.57, 0.70]	0.40[0.34, 0.48]	0.36[0.32, 0.37]	0.54[0.51, 0.60]	0.10[0.01, 0.23]	0.39[0.13, 0.89]	0.70[0.31, 0.92]	0.27[0.21, 0.44]	0.84[0.80, 0.89]
lr0.0001_wd0.0_h400_d1	0.65[0.56, 0.74]	0.39[0.32, 0.51]	0.40[0.30, 0.53]	0.57[0.44, 0.72]	0.13[-0.13, 0.37]	0.32[0.04, 0.74]	0.82[0.69, 1.00]	0.27[0.18, 0.43]	0.87[0.67, 1.00]
lr0.0001_wd0.0_h512_d1	0.66[0.55, 0.77]	0.41[0.30, 0.48]	0.32[0.26, 0.38]	0.50[0.48, 0.53]	0.02[-0.04, 0.12]	0.24[0.00, 0.70]	0.76[0.31, 1.00]	0.22[0.19, 0.23]	0.51[0.00, 1.00]
lr0.0001_wd0.0_h256_d2	0.68[0.60, 0.75]	0.39[0.31, 0.44]	0.35[0.20, 0.48]	0.56[0.50, 0.69]	0.10[0.00, 0.32]	0.35[0.00, 0.89]	0.77[0.15, 1.00]	0.26[0.21, 0.33]	0.52[0.00, 0.93]
lr0.0001_wd0.0_h400_d2	0.66[0.62, 0.71]	0.42[0.35, 0.48]	0.39[0.34, 0.47]	0.57[0.50, 0.67]	0.13[0.00, 0.28]	0.30[0.00, 0.71]	0.84[0.50, 1.00]	0.27[0.21, 0.33]	0.53[0.00, 0.92]
lr0.0001_wd0.0_h512_d2	0.66[0.57, 0.71]	0.40[0.32, 0.52]	0.34[0.19, 0.44]	0.54[0.46, 0.65]	0.03[-0.26, 0.25]	0.37[0.00, 0.87]	0.70[0.15, 1.00]	0.25[0.19, 0.31]	0.50[0.00, 0.89]
lr0.0001_wd0.0001_h256_d1	0.65[0.62, 0.71]	0.40[0.33, 0.48]	0.37[0.29, 0.41]	0.56[0.49, 0.62]	0.10[-0.01, 0.23]	0.39[0.00, 0.78]	0.72[0.46, 1.00]	0.26[0.21, 0.38]	0.67[0.00, 0.89]
lr0.0001_wd0.0001_h400_d1	0.67[0.60, 0.75]	0.40[0.33, 0.58]	0.41[0.35, 0.50]	0.61[0.52, 0.70]	0.22[0.10, 0.33]	0.43[0.04, 0.93]	0.78[0.31, 1.00]	0.34[0.21, 0.57]	0.91[0.83, 1.00]
lr0.0001_wd0.0001_h512_d1	0.68[0.64, 0.72]	0.45[0.34, 0.54]	0.38[0.34, 0.43]	0.57[0.51, 0.64]	0.13[0.03, 0.23]	0.35[0.04, 0.59]	0.79[0.54, 1.00]	0.26[0.21, 0.30]	0.88[0.82, 1.00]
lr0.0001_wd0.0001_h256_d2	0.67[0.63, 0.72]	0.39[0.34, 0.47]	0.35[0.32, 0.39]	0.53[0.50, 0.59]	0.06[0.00, 0.15]	0.32[0.00, 0.83]	0.75[0.31, 1.00]	0.25[0.21, 0.33]	0.49[0.00, 0.84]
lr0.0001_wd0.0001_h400_d2	0.66[0.57, 0.71]	0.40[0.37, 0.47]	0.38[0.29, 0.50]	0.54[0.42, 0.69]	0.06[-0.37, 0.33]	0.26[0.00, 0.76]	0.83[0.62, 1.00]	0.26[0.18, 0.42]	0.74[0.00, 1.00]
lr0.0001_wd0.0001_h512_d2	0.65[0.58, 0.72]	0.38[0.30, 0.48]	0.35[0.27, 0.42]	0.54[0.49, 0.63]	0.08[-0.02, 0.22]	0.45[0.00, 0.80]	0.64[0.38, 1.00]	0.26[0.21, 0.36]	0.66[0.00, 0.90]
lr0.0001_wd0.001_h256_d1	0.66[0.60, 0.72]	0.36[0.30, 0.44]	0.33[0.26, 0.37]	0.53[0.49, 0.57]	0.10[-0.01, 0.18]	0.42[0.04, 0.91]	0.65[0.23, 1.00]	0.27[0.22, 0.43]	0.88[0.77, 1.00]
lr0.0001_wd0.001_h400_d1	0.66[0.62, 0.70]	0.37[0.33, 0.42]	0.38[0.33, 0.45]	0.55[0.49, 0.66]	0.10[-0.02, 0.27]	0.27[0.00, 0.73]	0.84[0.58, 1.00]	0.26[0.20, 0.37]	0.68[0.00, 0.92]
lr0.0001_wd0.001_h512_d1	0.66[0.58, 0.72]	0.40[0.34, 0.52]	0.39[0.35, 0.48]	0.57[0.51, 0.69]	0.14[0.07, 0.32]	0.30[0.02, 0.56]	0.84[0.62, 1.00]	0.26[0.21, 0.33]	0.92[0.83, 1.00]
lr0.0001_wd0.001_h256_d2	0.67[0.61, 0.76]	0.45[0.37, 0.54]	0.36[0.31, 0.40]	0.54[0.50, 0.61]	0.06[0.00, 0.18]	0.28[0.00, 0.57]	0.80[0.46, 1.00]	0.24[0.21, 0.27]	0.50[0.00, 0.88]
lr0.0001_wd0.001_h400_d2	0.67[0.62, 0.75]	0.43[0.33, 0.53]	0.40[0.34, 0.55]	0.56[0.50, 0.73]	0.12[0.00, 0.42]	0.27[0.00, 0.80]	0.86[0.67, 1.00]	0.28[0.21, 0.47]	0.52[0.00, 0.90]
lr0.0001_wd0.001_h512_d2	0.67[0.60, 0.71]	0.42[0.33, 0.50]	0.35[0.26, 0.47]	0.53[0.47, 0.69]	0.04[-0.13, 0.32]	0.25[0.00, 0.70]	0.81[0.31, 1.00]	0.23[0.21, 0.31]	0.60[0.00, 0.95]
lr0.0003_wd0.0_h256_d1	0.67[0.62, 0.72]	0.43[0.32, 0.52]	0.38[0.27, 0.50]	0.57[0.44, 0.71]	0.10[-0.20, 0.34]	0.44[0.04, 0.72]	0.70[0.31, 0.85]	0.28[0.19, 0.39]	0.78[0.50, 0.93]
lr0.0003_wd0.0_h400_d1	0.67[0.62, 0.72]	0.36[0.30, 0.46]	0.27[0.00, 0.41]	0.55[0.48, 0.62]	0.08[-0.10, 0.20]	0.60[0.13, 0.96]	0.49[0.00, 0.92]	0.22[0.00, 0.33]	0.83[0.79, 0.88]
lr0.0003_wd0.0_h512_d1	0.68[0.56, 0.77]	0.43[0.33, 0.53]	0.41[0.23, 0.53]	0.63[0.51, 0.72]	0.23[0.01, 0.37]	0.59[0.15, 0.83]	0.66[0.25, 1.00]	0.32[0.21, 0.43]	0.89[0.80, 1.00]
lr0.0003_wd0.0_h256_d2	0.66[0.62, 0.70]	0.39[0.33, 0.52]	0.41[0.34, 0.47]	0.59[0.50, 0.67]	0.17[0.00, 0.29]	0.33[0.00, 0.76]	0.85[0.54, 1.00]	0.29[0.21, 0.39]	0.55[0.00, 1.00]
lr0.0003_wd0.0_h400_d2	0.66[0.58, 0.72]	0.39[0.33, 0.44]	0.38[0.32, 0.44]	0.57[0.46, 0.66]	0.10[-0.26, 0.26]	0.38[0.00, 0.85]	0.76[0.31, 1.00]	0.28[0.19, 0.36]	0.72[0.00, 1.00]
lr0.0003_wd0.0_h512_d2	0.69[0.59, 0.78]	0.37[0.31, 0.46]	0.38[0.13, 0.48]	0.62[0.53, 0.67]	0.25[0.13, 0.33]	0.71[0.20, 0.98]	0.52[0.08, 1.00]	0.40[0.26, 0.50]	0.87[0.79, 1.00]
lr0.0003_wd0.0001_h256_d1	0.67[0.60, 0.73]	0.36[0.30, 0.46]	0.42[0.36, 0.54]	0.62[0.56, 0.76]	0.23[0.10, 0.42]	0.49[0.13, 0.80]	0.76[0.38, 1.00]	0.31[0.24, 0.38]	0.91[0.82, 1.00]
lr0.0003_wd0.0001_h400_d1	0.67[0.60, 0.72]	0.42[0.31, 0.49]	0.41[0.32, 0.56]	0.61[0.54, 0.74]	0.19[0.06, 0.41]	0.50[0.09, 0.72]	0.71[0.46, 1.00]	0.29[0.24, 0.43]	0.89[0.80, 1.00]
lr0.0003_wd0.0001_h512_d1	0.66[0.58, 0.74]	0.38[0.28, 0.53]	0.36[0.26, 0.41]	0.58[0.54, 0.62]	0.17[0.09, 0.27]	0.49[0.11, 0.91]	0.66[0.23, 1.00]	0.31[0.23, 0.50]	0.88[0.80, 1.00]
lr0.0003_wd0.0001_h256_d2	0.68[0.60, 0.79]	0.38[0.29, 0.54]	0.44[0.39, 0.51]	0.65[0.61, 0.71]	0.26[0.17, 0.35]	0.55[0.31, 0.83]	0.75[0.46, 1.00]	0.33[0.28, 0.43]	0.90[0.84, 1.00]
lr0.0003_wd0.0001_h400_d2	0.68[0.62, 0.76]	0.43[0.34, 0.59]	0.36[0.34, 0.38]	0.55[0.50, 0.59]	0.09[0.00, 0.20]	0.38[0.00, 0.87]	0.72[0.31, 1.00]	0.27[0.22, 0.40]	0.67[0.00, 0.88]
lr0.0003_wd0.0001_h512_d2	0.66[0.57, 0.77]	0.41[0.33, 0.51]	0.41[0.33, 0.52]	0.59[0.50, 0.71]	0.16[0.00, 0.37]	0.43[0.00, 0.76]	0.75[0.38, 1.00]	0.30[0.21, 0.42]	0.52[0.00, 0.89]
lr0.0003_wd0.001_h256_d1	0.67[0.60, 0.77]	0.42[0.33, 0.56]	0.38[0.31, 0.44]	0.57[0.51, 0.64]	0.15[0.01, 0.29]	0.45[0.09, 0.87]	0.70[0.31, 1.00]	0.30[0.21, 0.45]	0.85[0.80, 1.00]
lr0.0003_wd0.001_h400_d1	0.67[0.56, 0.78]	0.39[0.28, 0.52]	0.39[0.32, 0.41]	0.58[0.48, 0.61]	0.14[-0.03, 0.22]	0.39[0.20, 0.61]	0.77[0.62, 1.00]	0.26[0.20, 0.31]	0.87[0.77, 1.00]
lr0.0003_wd0.001_h512_d1	0.66[0.57, 0.71]	0.40[0.33, 0.47]	0.39[0.38, 0.43]	0.59[0.56, 0.64]	0.15[0.10, 0.23]	0.45[0.35, 0.57]	0.73[0.62, 0.77]	0.27[0.25, 0.30]	0.86[0.84, 0.89]
lr0.0003_wd0.001_h256_d2	0.67[0.64, 0.70]	0.42[0.29, 0.50]	0.38[0.34, 0.42]	0.56[0.50, 0.64]	0.11[0.00, 0.23]	0.26[0.00, 0.69]	0.86[0.58, 1.00]	0.26[0.21, 0.33]	0.54[0.00, 1.00]
lr0.0003_wd0.001_h400_d2	0.68[0.62, 0.75]	0.38[0.31, 0.48]	0.44[0.37, 0.51]	0.65[0.59, 0.72]	0.25[0.18, 0.36]	0.63[0.50, 0.80]	0.67[0.38, 0.85]	0.34[0.28, 0.39]	0.88[0.82, 0.93]
lr0.0003_wd0.001_h512_d2	0.68[0.59, 0.76]	0.41[0.30, 0.52]	0.42[0.34, 0.47]	0.61[0.50, 0.67]	0.18[0.00, 0.29]	0.47[0.00, 0.72]	0.75[0.62, 1.00]	0.30[0.21, 0.38]	0.70[0.00, 0.88]
lr0.001_wd0.0_h256_d1	0.66[0.58, 0.71]	0.41[0.29, 0.51]	0.41[0.36, 0.50]	0.60[0.53, 0.69]	0.17[0.05, 0.33]	0.43[0.13, 0.70]	0.76[0.67, 0.92]	0.28[0.23, 0.39]	0.86[0.81, 0.89]
lr0.001_wd0.0_h400_d1	0.65[0.60, 0.71]	0.38[0.31, 0.49]	0.38[0.25, 0.43]	0.59[0.53, 0.64]	0.16[0.06, 0.23]	0.48[0.28, 0.83]	0.70[0.23, 0.92]	0.27[0.23, 0.30]	0.87[0.79, 0.93]
lr0.001_wd0.0_h512_d1	0.67[0.58, 0.78]	0.42[0.32, 0.57]	0.43[0.34, 0.56]	0.61[0.50, 0.73]	0.21[0.00, 0.44]	0.40[0.00, 0.87]	0.82[0.58, 1.00]	0.32[0.21, 0.54]	0.72[0.00, 0.92]
lr0.001_wd0.0_h256_d2	0.68[0.60, 0.73]	0.38[0.29, 0.50]	0.38[0.26, 0.49]	0.60[0.54, 0.70]	0.18[0.09, 0.32]	0.55[0.24, 0.85]	0.65[0.23, 1.00]	0.30[0.24, 0.36]	0.88[0.80, 1.00]
lr0.001_wd0.0_h400_d2	0.68[0.64, 0.78]	0.38[0.28, 0.61]	0.39[0.32, 0.52]	0.59[0.50, 0.69]	0.16[0.00, 0.40]	0.57[0.00, 0.89]	0.60[0.38, 1.00]	0.32[0.22, 0.55]	0.67[0.00, 0.88]
lr0.001_wd0.0_h512_d2	0.69[0.63, 0.78]	0.41[0.34, 0.54]	0.39[0.21, 0.48]	0.61[0.53, 0.68]	0.20[0.09, 0.32]	0.56[0.15, 0.91]	0.65[0.15, 1.00]	0.32[0.25, 0.41]	0.89[0.79, 1.00]
lr0.001_wd0.0001_h256_d1	0.69[0.60, 0.80]	0.43[0.34, 0.60]	0.41[0.35, 0.50]	0.60[0.51, 0.69]	0.19[0.07, 0.33]	0.43[0.02, 0.76]	0.78[0.58, 1.00]	0.29[0.22, 0.42]	0.90[0.83, 1.00]
lr0.001_wd0.0001_h400_d1	0.69[0.58, 0.80]	0.43[0.34, 0.58]	0.41[0.35, 0.50]	0.61[0.56, 0.72]	0.20[0.10, 0.35]	0.58[0.28, 0.76]	0.65[0.46, 0.85]	0.31[0.25, 0.39]	0.86[0.82, 0.93]
lr0.001_wd0.0001_h512_d1	0.68[0.62, 0.78]	0.40[0.32, 0.54]	0.35[0.21, 0.48]	0.57[0.46, 0.69]	0.10[-0.26, 0.32]	0.48[0.00, 0.91]	0.66[0.15, 1.00]	0.30[0.19, 0.38]	0.71[0.00, 1.00]
lr0.001_wd0.0001_h256_d2	0.69[0.62, 0.78]	0.42[0.30, 0.58]	0.41[0.38, 0.43]	0.61[0.53, 0.64]	0.23[0.12, 0.33]	0.56[0.07, 0.93]	0.66[0.33, 1.00]	0.36[0.23, 0.57]	0.91[0.83, 1.00]
lr0.001_wd0.0001_h400_d2	0.70[0.63, 0.82]	0.40[0.33, 0.58]	0.39[0.22, 0.48]	0.62[0.54, 0.69]	0.21[0.07, 0.34]	0.55[0.38, 0.93]	0.69[0.15, 1.00]	0.31[0.23, 0.40]	0.89[0.80, 1.00]
lr0.001_wd0.0001_h512_d2	0.69[0.62, 0.78]	0.45[0.33, 0.67]	0.35[0.18, 0.42]	0.56[0.50, 0.64]	0.12[0.00, 0.22]	0.41[0.02, 0.85]	0.72[0.15, 1.00]	0.25[0.22, 0.31]	0.89[0.78, 1.00]
lr0.001_wd0.001_h256_d1	0.66[0.62, 0.72]	0.37[0.29, 0.55]	0.42[0.36, 0.48]	0.63[0.58, 0.69]	0.23[0.15, 0.32]	0.65[0.50, 0.78]	0.62[0.38, 0.85]	0.33[0.29, 0.38]	0.87[0.82, 0.92]
lr0.001_wd0.001_h400_d1	0.65[0.58, 0.73]	0.40[0.29, 0.48]	0.35[0.27, 0.47]	0.56[0.47, 0.67]	0.10[-0.09, 0.30]	0.58[0.09, 0.87]	0.54[0.23, 0.85]	0.29[0.21, 0.39]	0.80[0.67, 0.87]
lr0.001_wd0.001_h512_d1	0.69[0.60, 0.74]	0.39[0.36, 0.41]	0.43[0.35, 0.50]	0.62[0.54, 0.70]	0.20[0.08, 0.33]	0.46[0.24, 0.69]	0.78[0.67, 0.85]	0.30[0.23, 0.37]	0.88[0.84, 0.91]
lr0.001_wd0.001_h256_d2	0.69[0.60, 0.74]	0.36[0.28, 0.41]	0.35[0.21, 0.46]	0.56[0.50, 0.67]	0.13[0.00, 0.29]	0.41[0.00, 0.91]	0.72[0.15, 1.00]	0.28[0.21, 0.33]	0.72[0.00, 1.00]
lr0.001_wd0.001_h400_d2	0.69[0.60, 0.79]	0.42[0.30, 0.60]	0.33[0.00, 0.48]	0.59[0.47, 0.69]	0.16[-0.12, 0.32]	0.57[0.17, 0.93]	0.62[0.00, 1.00]	0.23[0.00, 0.33]	0.89[0.77, 1.00]
lr0.001_wd0.001_h512_d2	0.68[0.58, 0.74]	0.39[0.33, 0.53]	0.37[0.13, 0.49]	0.60[0.50, 0.70]	0.19[0.00, 0.32]	0.56[0.00, 0.96]	0.64[0.08, 1.00]	0.32[0.22, 0.40]	0.70[0.00, 0.95]

Supplementary Table 38: Grid search results on the classification task on SYSMH external validation dataset (choosing 60% as the threshold for clinical variables selection). Reported metrics are AUC, AUPRC, F1, Balanced Accuracy, MCC, Specificity, Sensitivity, PPV, and NPV.

Config	AUC	AUPRC	F1	Balanced_Accuracy	MCC	Specificity	Sensitivity	PPV	NPV
lr1e-05_wd0.005_h1024_d3	0.65 [0.61, 0.72]	0.40 [0.32, 0.49]	0.39 [0.30, 0.50]	0.57 [0.50, 0.70]	0.11 [0.00, 0.33]	0.35 [0.00, 0.63]	0.78 [0.46, 1.00]	0.27 [0.21, 0.37]	0.51 [0.00, 0.91]
lr1e-05_wd0.005_h2048_d3	0.67 [0.61, 0.75]	0.40 [0.32, 0.48]	0.36 [0.33, 0.41]	0.52 [0.47, 0.62]	0.03 [-0.09, 0.20]	0.20 [0.00, 0.52]	0.84 [0.54, 1.00]	0.23 [0.21, 0.27]	0.47 [0.00, 0.90]
lr1e-05_wd0.005_h1024_d4	0.66 [0.61, 0.73]	0.36 [0.31, 0.46]	0.37 [0.33, 0.44]	0.54 [0.50, 0.64]	0.07 [0.00, 0.25]	0.28 [0.00, 0.74]	0.80 [0.54, 1.00]	0.25 [0.21, 0.37]	0.50 [0.00, 0.88]
lr1e-05_wd0.005_h2048_d4	0.68 [0.63, 0.75]	0.38 [0.32, 0.47]	0.28 [0.00, 0.44]	0.52 [0.45, 0.66]	0.03 [-0.16, 0.29]	0.36 [0.00, 0.89]	0.68 [0.00, 1.00]	0.18 [0.00, 0.28]	0.51 [0.00, 1.00]
lr1e-05_wd0.01_h1024_d3	0.65 [0.59, 0.73]	0.38 [0.31, 0.47]	0.29 [0.00, 0.40]	0.52 [0.50, 0.61]	0.04 [0.00, 0.18]	0.28 [0.00, 1.00]	0.77 [0.00, 1.00]	0.18 [0.00, 0.26]	0.33 [0.00, 0.89]
lr1e-05_wd0.01_h2048_d3	0.64 [0.57, 0.70]	0.36 [0.32, 0.46]	0.29 [0.00, 0.43]	0.54 [0.50, 0.64]	0.08 [0.00, 0.24]	0.49 [0.00, 1.00]	0.59 [0.00, 1.00]	0.22 [0.00, 0.33]	0.49 [0.00, 0.88]
lr1e-05_wd0.01_h1024_d4	0.65 [0.59, 0.72]	0.39 [0.31, 0.45]	0.28 [0.00, 0.44]	0.54 [0.50, 0.66]	0.06 [0.00, 0.26]	0.48 [0.00, 1.00]	0.59 [0.00, 1.00]	0.20 [0.00, 0.33]	0.49 [0.00, 0.88]
lr1e-05_wd0.01_h2048_d4	0.67 [0.65, 0.71]	0.38 [0.31, 0.47]	0.31 [0.10, 0.42]	0.53 [0.46, 0.63]	0.04 [-0.09, 0.22]	0.38 [0.00, 0.85]	0.67 [0.08, 1.00]	0.22 [0.13, 0.31]	0.49 [0.00, 0.87]
lr3e-05_wd0.005_h1024_d3	0.66 [0.62, 0.70]	0.39 [0.33, 0.47]	0.40 [0.34, 0.46]	0.58 [0.50, 0.65]	0.15 [0.00, 0.31]	0.30 [0.00, 0.85]	0.86 [0.46, 1.00]	0.29 [0.21, 0.46]	0.54 [0.00, 0.94]
lr3e-05_wd0.005_h2048_d3	0.66 [0.61, 0.73]	0.43 [0.30, 0.51]	0.32 [0.25, 0.36]	0.51 [0.50, 0.55]	0.03 [0.00, 0.09]	0.32 [0.00, 0.83]	0.71 [0.23, 1.00]	0.24 [0.21, 0.29]	0.32 [0.00, 0.80]
lr3e-05_wd0.005_h1024_d4	0.67 [0.60, 0.73]	0.37 [0.33, 0.43]	0.33 [0.22, 0.39]	0.52 [0.45, 0.59]	0.05 [-0.09, 0.17]	0.36 [0.00, 0.85]	0.69 [0.31, 1.00]	0.24 [0.17, 0.36]	0.49 [0.00, 0.89]
lr3e-05_wd0.005_h2048_d4	0.66 [0.61, 0.73]	0.39 [0.32, 0.46]	0.32 [0.00, 0.47]	0.56 [0.50, 0.67]	0.10 [0.00, 0.30]	0.51 [0.00, 1.00]	0.61 [0.00, 1.00]	0.24 [0.00, 0.39]	0.50 [0.00, 0.87]
lr3e-05_wd0.01_h1024_d3	0.66 [0.62, 0.74]	0.39 [0.31, 0.46]	0.33 [0.27, 0.36]	0.52 [0.50, 0.57]	0.05 [0.00, 0.14]	0.34 [0.00, 0.87]	0.71 [0.23, 1.00]	0.26 [0.21, 0.33]	0.32 [0.00, 0.81]
lr3e-05_wd0.01_h2048_d3	0.68 [0.63, 0.73]	0.43 [0.32, 0.49]	0.32 [0.10, 0.41]	0.52 [0.46, 0.60]	0.04 [-0.09, 0.17]	0.30 [0.00, 0.85]	0.75 [0.08, 1.00]	0.21 [0.13, 0.28]	0.51 [0.00, 0.90]
lr3e-05_wd0.01_h1024_d4	0.66 [0.63, 0.69]	0.41 [0.32, 0.46]	0.34 [0.24, 0.41]	0.52 [0.48, 0.62]	0.04 [-0.04, 0.23]	0.29 [0.00, 0.78]	0.75 [0.31, 1.00]	0.24 [0.20, 0.38]	0.32 [0.00, 0.84]
lr3e-05_wd0.01_h2048_d4	0.68 [0.63, 0.74]	0.40 [0.30, 0.53]	0.37 [0.32, 0.43]	0.56 [0.50, 0.64]	0.11 [0.00, 0.25]	0.36 [0.00, 0.83]	0.75 [0.31, 1.00]	0.28 [0.21, 0.38]	0.51 [0.00, 0.91]
lr1e-05_wd0.0_h256_d1	0.65 [0.59, 0.75]	0.40 [0.31, 0.57]	0.32 [0.25, 0.36]	0.48 [0.44, 0.50]	-0.07 [-0.18, 0.00]	0.15 [0.00, 0.67]	0.81 [0.31, 1.00]	0.21 [0.20, 0.22]	0.35 [0.00, 0.78]
lr1e-05_wd0.0_h400_d1	0.64 [0.59, 0.71]	0.34 [0.30, 0.37]	0.33 [0.29, 0.36]	0.49 [0.47, 0.50]	-0.03 [-0.14, 0.01]	0.16 [0.00, 0.52]	0.83 [0.46, 1.00]	0.21 [0.20, 0.22]	0.41 [0.00, 0.79]
lr1e-05_wd0.0_h512_d1	0.67 [0.61, 0.72]	0.39 [0.33, 0.45]	0.34 [0.29, 0.36]	0.51 [0.50, 0.53]	0.03 [0.00, 0.07]	0.16 [0.00, 0.76]	0.86 [0.31, 1.00]	0.23 [0.21, 0.27]	0.36 [0.00, 1.00]
lr1e-05_wd0.0_h256_d2	0.63 [0.61, 0.69]	0.35 [0.28, 0.46]	0.35 [0.32, 0.37]	0.52 [0.50, 0.58]	0.07 [0.00, 0.23]	0.20 [0.00, 0.93]	0.85 [0.23, 1.00]	0.27 [0.21, 0.50]	0.56 [0.00, 1.00]
lr1e-05_wd0.0_h400_d2	0.65 [0.61, 0.72]	0.39 [0.30, 0.47]	0.28 [0.00, 0.36]	0.50 [0.50, 0.51]	0.01 [0.00, 0.04]	0.26 [0.00, 1.00]	0.75 [0.00, 1.00]	0.17 [0.00, 0.22]	0.48 [0.00, 0.83]
lr1e-05_wd0.0_h512_d2	0.65 [0.60, 0.70]	0.41 [0.39, 0.45]	0.26 [0.00, 0.36]	0.50 [0.49, 0.50]	-0.00 [-0.02, 0.00]	0.32 [0.00, 1.00]	0.68 [0.00, 1.00]	0.17 [0.00, 0.22]	0.31 [0.00, 0.78]
lr1e-05_wd0.0001_h256_d1	0.61 [0.56, 0.67]	0.37 [0.29, 0.46]	0.34 [0.32, 0.37]	0.50 [0.46, 0.57]	-0.02 [-0.26, 0.14]	0.20 [0.00, 0.83]	0.81 [0.31, 1.00]	0.24 [0.19, 0.33]	0.51 [0.00, 1.00]
lr1e-05_wd0.0001_h400_d1	0.65 [0.59, 0.72]	0.42 [0.31, 0.47]	0.32 [0.23, 0.36]	0.48 [0.46, 0.50]	-0.08 [-0.26, 0.00]	0.16 [0.00, 0.61]	0.80 [0.31, 1.00]	0.20 [0.18, 0.22]	0.30 [0.00, 0.76]
lr1e-05_wd0.0001_h512_d1	0.65 [0.58, 0.70]	0.41 [0.33, 0.47]	0.34 [0.27, 0.38]	0.51 [0.50, 0.53]	0.03 [0.00, 0.12]	0.16 [0.00, 0.72]	0.86 [0.31, 1.00]	0.22 [0.21, 0.24]	0.36 [0.00, 1.00]
lr1e-05_wd0.0001_h256_d2	0.65 [0.60, 0.74]	0.36 [0.32, 0.43]	0.33 [0.25, 0.36]	0.49 [0.46, 0.50]	-0.06 [-0.26, 0.00]	0.13 [0.00, 0.67]	0.84 [0.31, 1.00]	0.21 [0.20, 0.22]	0.15 [0.00, 0.78]
lr1e-05_wd0.0001_h400_d2	0.65 [0.59, 0.74]	0.37 [0.31, 0.42]	0.29 [0.00, 0.38]	0.52 [0.50, 0.57]	0.03 [0.00, 0.12]	0.30 [0.00, 1.00]	0.74 [0.00, 1.00]	0.18 [0.00, 0.26]	0.49 [0.00, 0.85]
lr1e-05_wd0.0001_h512_d2	0.64 [0.61, 0.68]	0.39 [0.31, 0.50]	0.35 [0.33, 0.38]	0.51 [0.47, 0.58]	0.01 [-0.06, 0.13]	0.14 [0.00, 0.54]	0.88 [0.62, 1.00]	0.22 [0.21, 0.28]	0.31 [0.00, 0.83]
lr1e-05_wd0.001_h256_d1	0.66 [0.58, 0.78]	0.42 [0.31, 0.53]	0.33 [0.31, 0.36]	0.48 [0.43, 0.56]	-0.08 [-0.26, 0.11]	0.19 [0.00, 0.80]	0.78 [0.31, 1.00]	0.22 [0.19, 0.31]	0.41 [0.00, 0.80]
lr1e-05_wd0.001_h400_d1	0.66 [0.59, 0.79]	0.42 [0.29, 0.57]	0.26 [0.00, 0.37]	0.50 [0.49, 0.53]	0.01 [-0.02, 0.07]	0.38 [0.00, 1.00]	0.63 [0.00, 1.00]	0.17 [0.00, 0.23]	0.65 [0.00, 0.86]
lr1e-05_wd0.001_h512_d1	0.65 [0.60, 0.72]	0.37 [0.27, 0.48]	0.34 [0.28, 0.37]	0.52 [0.50, 0.55]	0.06 [0.00, 0.11]	0.25 [0.00, 0.74]	0.80 [0.31, 1.00]	0.23 [0.21, 0.25]	0.70 [0.00, 1.00]
lr1e-05_wd0.001_h256_d2	0.65 [0.61, 0.71]	0.34 [0.29, 0.39]	0.29 [0.00, 0.38]	0.51 [0.50, 0.53]	0.02 [0.00, 0.12]	0.21 [0.00, 1.00]	0.80 [0.00, 1.00]	0.17 [0.00, 0.23]	0.36 [0.00, 1.00]
lr1e-05_wd0.001_h400_d2	0.66 [0.63, 0.72]	0.37 [0.33, 0.42]	0.29 [0.00, 0.38]	0.51 [0.50, 0.54]	0.03 [0.00, 0.14]	0.22 [0.00, 1.00]	0.80 [0.00, 1.00]	0.17 [0.00, 0.24]	0.36 [0.00, 1.00]
lr1e-05_wd0.001_h512_d2	0.69 [0.59, 0.77]	0.45 [0.37, 0.54]	0.26 [0.00, 0.36]	0.49 [0.47, 0.50]	-0.01 [-0.04, 0.00]	0.31 [0.00, 1.00]	0.68 [0.00, 1.00]	0.17 [0.00, 0.22]	0.31 [0.00, 0.78]
lr3e-05_wd0.0_h256_d1	0.66 [0.60, 0.71]	0.39 [0.33, 0.52]	0.35 [0.30, 0.42]	0.52 [0.48, 0.61]	0.03 [-0.07, 0.19]	0.23 [0.00, 0.67]	0.81 [0.38, 1.00]	0.23 [0.20, 0.29]	0.47 [0.00, 0.88]
lr3e-05_wd0.0_h400_d1	0.66 [0.59, 0.74]	0.42 [0.33, 0.51]	0.36 [0.34, 0.38]	0.53 [0.50, 0.61]	0.06 [0.00, 0.27]	0.23 [0.00, 0.91]	0.82 [0.31, 1.00]	0.27 [0.21, 0.50]	0.32 [0.00, 0.82]
lr3e-05_wd0.0_h512_d1	0.66 [0.63, 0.71]	0.41 [0.34, 0.47]	0.35 [0.29, 0.38]	0.53 [0.51, 0.58]	0.06 [0.01, 0.15]	0.29 [0.02, 0.63]	0.77 [0.38, 1.00]	0.23 [0.21, 0.25]	0.86 [0.78, 1.00]
lr3e-05_wd0.0_h256_d2	0.67 [0.63, 0.73]	0.41 [0.31, 0.49]	0.40 [0.34, 0.54]	0.57 [0.50, 0.73]	0.12 [0.00, 0.39]	0.24 [0.00, 0.70]	0.89 [0.77, 1.00]	0.26 [0.21, 0.42]	0.53 [0.00, 0.91]
lr3e-05_wd0.0_h400_d2	0.66 [0.58, 0.74]	0.40 [0.32, 0.51]	0.37 [0.33, 0.41]	0.54 [0.50, 0.62]	0.07 [0.00, 0.20]	0.29 [0.00, 0.58]	0.79 [0.54, 1.00]	0.24 [0.21, 0.30]	0.50 [0.00, 0.87]
lr3e-05_wd0.0_h512_d2	0.66 [0.59, 0.74]	0.40 [0.32, 0.48]	0.38 [0.34, 0.43]	0.56 [0.50, 0.62]	0.12 [0.00, 0.21]	0.33 [0.00, 0.87]	0.80 [0.31, 1.00]	0.28 [0.21, 0.40]	0.52 [0.00, 0.93]
lr3e-05_wd0.0001_h256_d1	0.66 [0.60, 0.71]	0.39 [0.35, 0.48]	0.34 [0.31, 0.37]	0.52 [0.46, 0.56]	0.03 [-0.09, 0.13]	0.23 [0.00, 0.80]	0.80 [0.31, 1.00]	0.23 [0.20, 0.31]	0.48 [0.00, 0.90]
lr3e-05_wd0.0001_h400_d1	0.66 [0.61, 0.69]	0.43 [0.31, 0.51]	0.34 [0.32, 0.37]	0.50 [0.47, 0.57]	-0.01 [-0.14, 0.14]	0.21 [0.02, 0.76]	0.80 [0.38, 1.00]	0.23 [0.20, 0.31]	0.76 [0.50, 1.00]
lr3e-05_wd0.0001_h512_d1	0.64 [0.60, 0.70]	0.39 [0.35, 0.43]	0.32 [0.00, 0.51]	0.57 [0.50, 0.71]	0.12 [0.00, 0.35]	0.52 [0.20, 1.00]	0.61 [0.00, 0.85]	0.22 [0.00, 0.38]	0.84 [0.79, 0.91]
lr3e-05_wd0.0001_h256_d2	0.66 [0.64, 0.70]	0.38 [0.31, 0.46]	0.37 [0.28, 0.49]	0.55 [0.50, 0.71]	0.08 [0.00, 0.34]	0.26 [0.00, 0.74]	0.83 [0.31, 1.00]	0.25 [0.21, 0.34]	0.34 [0.00, 0.93]
lr3e-05_wd0.0001_h400_d2	0.66 [0.62, 0.70]	0.41 [0.36, 0.46]	0.28 [0.00, 0.40]	0.51 [0.44, 0.61]	0.01 [-0.15, 0.18]	0.30 [0.00, 1.00]	0.72 [0.00, 1.00]	0.18 [0.00, 0.26]	0.46 [0.00, 0.89]
lr3e-05_wd0.0001_h512_d2	0.66 [0.62, 0.71]	0.40 [0.34, 0.47]	0.36 [0.32, 0.41]	0.53 [0.50, 0.60]	0.04 [0.00, 0.17]	0.20 [0.00, 0.59]	0.85 [0.46, 1.00]	0.23 [0.21, 0.28]	0.33 [0.00, 0.87]
lr3e-05_wd0.001_h256_d1	0.66 [0.60, 0.70]	0.39 [0.34, 0.50]	0.31 [0.14, 0.38]	0.51 [0.46, 0.56]	0.02 [-0.26, 0.25]	0.27 [0.00, 1.00]	0.75 [0.08, 1.00]	0.37 [0.20, 1.00]	0.33 [0.00, 0.84]
lr3e-05_wd0.001_h400_d1	0.67 [0.61, 0.73]	0.42 [0.36, 0.51]	0.34 [0.31, 0.39]	0.51 [0.46, 0.58]	0.02 [-0.09, 0.16]	0.19 [0.00, 0.57]	0.83 [0.46, 1.00]	0.22 [0.20, 0.24]	0.48 [0.00, 0.92]
lr3e-05_wd0.001_h512_d1	0.65 [0.61, 0.71]	0.42 [0.32, 0.49]	0.38 [0.30, 0.45]	0.56 [0.43, 0.67]	0.08 [-0.27, 0.29]	0.32 [0.02, 0.59]	0.80 [0.46, 1.00]	0.25 [0.18, 0.32]	0.79 [0.33, 1.00]
lr3e-05_wd0.001_h256_d2	0.65 [0.60, 0.72]	0.36 [0.31, 0.48]	0.33 [0.25, 0.36]	0.50 [0.48, 0.51]	-0.00 [-0.04, 0.04]	0.20 [0.00, 0.67]	0.80 [0.31, 1.00]	0.21 [0.21, 0.22]	0.47 [0.00, 0.83]
lr3e-05_wd0.001_h400_d2	0.68 [0.64, 0.74]	0.40 [0.34, 0.48]	0.28 [0.00, 0.38]	0.51 [0.46, 0.57]	0.01 [-0.09, 0.13]	0.29 [0.00, 1.00]	0.72 [0.00, 1.00]	0.18 [0.00, 0.24]	0.47 [0.00, 0.88]
lr3e-05_wd0.001_h512_d2	0.66 [0.62, 0.72]	0.40 [0.33, 0.48]	0.35 [0.28, 0.38]	0.52 [0.50, 0.57]	0.04 [-0.01, 0.13]	0.19 [0.00, 0.61]	0.84 [0.38, 1.00]	0.22 [0.21, 0.24]	0.53 [0.00, 1.00]
lr0.0001_wd0.005_h256_d1	0.66 [0.62, 0.71]	0.39 [0.30, 0.51]	0.36 [0.27, 0.42]	0.56 [0.49, 0.61]	0.12 [-0.02, 0.19]	0.33 [0.04, 0.59]	0.78 [0.38, 1.00]	0.24 [0.21, 0.29]	0.89 [0.77, 1.00]
lr0.0001_wd0.005_h400_d1	0.66 [0.59, 0.73]	0.39 [0.33, 0.46]	0.37 [0.30, 0.42]	0.55 [0.44, 0.62]	0.09 [-0.20, 0.22]	0.29 [0.02, 0.78]	0.81 [0.38, 1.00]	0.25 [0.19, 0.33]	0.84 [0.50, 1.00]
lr0.0001_wd0.005_h512_d1	0.68 [0.61, 0.77]	0.42 [0.33, 0.50]	0.37 [0.30, 0.44]	0.57 [0.46, 0.66]	0.15 [-0.07, 0.29]	0.46 [0.26, 0.83]	0.69 [0.31, 1.00]	0.27 [0.19, 0.33]	0.87 [0.75, 1.00]
lr0.0001_wd0.005_h256_d2	0.67 [0.61, 0.71]	0.41 [0.32, 0.52]	0.35 [0.33, 0.36]	0.53 [0.50, 0.58]	0.07 [0.00, 0.17]	0.33 [0.00, 0.85]	0.74 [0.31, 1.00]	0.25 [0.21, 0.36]	0.50 [0.00, 0.86]
lr0.0001_wd0.005_h400_d2	0.68 [0.61, 0.73]	0.43 [0.34, 0.52]	0.39 [0.34, 0.48]	0.58 [0.50, 0.69]	0.15 [0.00, 0.32]	0.34 [0.00, 0.85]	0.81 [0.38, 1.00]	0.29 [0.21, 0.42]	0.72 [0.00, 1.00]
lr0.0001_wd0.005_h512_d2	0.63 [0.59, 0.67]	0.37 [0.30, 0.44]	0.37 [0.33, 0.40]	0.57 [0.50, 0.61]	0.12 [0.00, 0.19]	0.40 [0.00, 0.85]	0.73 [0.31, 1.00]	0.28 [0.21, 0.36]	0.69 [0.00, 0.89]
lr0.0001_wd0.01_h256_d1	0.65 [0.56, 0.72]	0.39 [0.32, 0.44]	0.40 [0.36, 0.45]	0.60 [0.54, 0.65]	0.19 [0.08, 0.27]	0.42 [0.16, 0.76]	0.78 [0.54, 1.00]	0.29 [0.22, 0.39]	0.90 [0.85, 1.00]
lr0.0001_wd0.01_h400_d1	0.68 [0.59, 0.75]	0.43 [0.29, 0.54]	0.40 [0.33, 0.49]	0.61 [0.53, 0.68]	0.20 [0.11, 0.31]	0.55 [0.07, 0.85]	0.67 [0.31, 1.00]	0.31 [0.23, 0.38]	0.89 [0.81, 1.00]

Supplementary Table 39: Grid search results on the classification task on SYSMH external validation dataset (choosing 60% as the threshold for clinical variables selection). Reported metrics are AUC, AUPRC, F1, Balanced Accuracy, MCC, Specificity, Sensitivity, PPV, and NPV.

Config	AUC	AUPRC	F1	Balanced_Accuracy	MCC	Specificity	Sensitivity	PPV	NPV
lr0.0001_wd0.01_h512_d1	0.65 [0.57, 0.71]	0.37 [0.31, 0.51]	0.35 [0.31, 0.39]	0.56 [0.55, 0.57]	0.13 [0.09, 0.18]	0.48 [0.13, 0.80]	0.64 [0.31, 1.00]	0.27 [0.23, 0.31]	0.87 [0.80, 1.00]
lr0.0001_wd0.01_h256_d2	0.66 [0.58, 0.76]	0.39 [0.34, 0.44]	0.36 [0.26, 0.45]	0.55 [0.47, 0.66]	0.09 [-0.05, 0.27]	0.45 [0.00, 0.80]	0.65 [0.38, 1.00]	0.27 [0.20, 0.40]	0.65 [0.00, 0.90]
lr0.0001_wd0.01_h400_d2	0.65 [0.58, 0.70]	0.36 [0.30, 0.42]	0.36 [0.32, 0.43]	0.56 [0.50, 0.64]	0.11 [0.00, 0.23]	0.45 [0.00, 0.83]	0.68 [0.31, 1.00]	0.27 [0.22, 0.33]	0.68 [0.00, 0.91]
lr0.0001_wd0.01_h512_d2	0.66 [0.61, 0.72]	0.39 [0.32, 0.55]	0.29 [0.00, 0.43]	0.55 [0.50, 0.64]	0.09 [0.00, 0.24]	0.54 [0.00, 1.00]	0.56 [0.00, 1.00]	0.22 [0.00, 0.32]	0.67 [0.00, 0.91]
lr0.0003_wd0.005_h256_d1	0.68 [0.62, 0.74]	0.40 [0.35, 0.49]	0.33 [0.00, 0.47]	0.59 [0.50, 0.68]	0.18 [0.00, 0.30]	0.61 [0.20, 1.00]	0.58 [0.00, 1.00]	0.25 [0.00, 0.36]	0.88 [0.79, 1.00]
lr0.0003_wd0.005_h400_d1	0.67 [0.55, 0.77]	0.40 [0.34, 0.49]	0.38 [0.24, 0.53]	0.60 [0.55, 0.72]	0.19 [0.10, 0.37]	0.48 [0.20, 0.93]	0.71 [0.17, 1.00]	0.31 [0.24, 0.40]	0.88 [0.81, 1.00]
lr0.0003_wd0.005_h512_d1	0.68 [0.61, 0.75]	0.39 [0.31, 0.51]	0.29 [0.00, 0.39]	0.55 [0.50, 0.60]	0.11 [0.00, 0.17]	0.60 [0.02, 1.00]	0.50 [0.00, 1.00]	0.23 [0.00, 0.36]	0.86 [0.79, 1.00]
lr0.0003_wd0.005_h256_d2	0.67 [0.57, 0.75]	0.42 [0.36, 0.55]	0.40 [0.32, 0.48]	0.58 [0.47, 0.68]	0.15 [-0.07, 0.34]	0.33 [0.00, 0.87]	0.83 [0.46, 1.00]	0.29 [0.20, 0.50]	0.69 [0.00, 1.00]
lr0.0003_wd0.005_h400_d2	0.67 [0.64, 0.71]	0.42 [0.34, 0.51]	0.38 [0.33, 0.49]	0.55 [0.47, 0.68]	0.09 [-0.06, 0.31]	0.29 [0.00, 0.67]	0.81 [0.69, 1.00]	0.25 [0.21, 0.38]	0.66 [0.00, 0.89]
lr0.0003_wd0.005_h512_d2	0.71 [0.62, 0.78]	0.40 [0.30, 0.46]	0.37 [0.32, 0.43]	0.55 [0.49, 0.62]	0.11 [-0.03, 0.25]	0.26 [0.00, 0.83]	0.84 [0.31, 1.00]	0.25 [0.20, 0.33]	0.71 [0.00, 1.00]
lr0.0003_wd0.01_h256_d1	0.67 [0.59, 0.74]	0.37 [0.30, 0.53]	0.42 [0.38, 0.49]	0.62 [0.58, 0.71]	0.20 [0.14, 0.34]	0.45 [0.33, 0.58]	0.79 [0.69, 0.85]	0.29 [0.24, 0.34]	0.89 [0.85, 0.93]
lr0.0003_wd0.01_h400_d1	0.66 [0.57, 0.75]	0.38 [0.32, 0.51]	0.37 [0.33, 0.40]	0.55 [0.52, 0.60]	0.09 [0.04, 0.17]	0.33 [0.11, 0.59]	0.76 [0.62, 0.92]	0.24 [0.22, 0.30]	0.84 [0.82, 0.86]
lr0.0003_wd0.01_h512_d1	0.66 [0.65, 0.71]	0.42 [0.29, 0.61]	0.35 [0.19, 0.42]	0.57 [0.51, 0.63]	0.13 [0.02, 0.21]	0.54 [0.20, 0.85]	0.60 [0.17, 0.92]	0.27 [0.22, 0.35]	0.85 [0.80, 0.90]
lr0.0003_wd0.01_h256_d2	0.68 [0.58, 0.79]	0.39 [0.29, 0.48]	0.35 [0.19, 0.44]	0.55 [0.50, 0.65]	0.10 [0.00, 0.28]	0.44 [0.00, 0.87]	0.67 [0.15, 1.00]	0.27 [0.22, 0.40]	0.68 [0.00, 0.91]
lr0.0003_wd0.01_h400_d2	0.68 [0.60, 0.77]	0.40 [0.34, 0.52]	0.36 [0.32, 0.38]	0.55 [0.47, 0.61]	0.08 [-0.14, 0.21]	0.45 [0.02, 0.80]	0.65 [0.38, 0.92]	0.28 [0.20, 0.36]	0.76 [0.50, 0.84]
lr0.0003_wd0.01_h512_d2	0.67 [0.57, 0.75]	0.40 [0.28, 0.52]	0.34 [0.11, 0.43]	0.57 [0.48, 0.63]	0.12 [-0.04, 0.23]	0.55 [0.20, 0.89]	0.59 [0.08, 0.92]	0.26 [0.17, 0.33]	0.85 [0.77, 0.90]
lr0.001_wd0.005_h256_d1	0.67 [0.58, 0.75]	0.40 [0.34, 0.50]	0.37 [0.26, 0.46]	0.57 [0.48, 0.68]	0.13 [-0.06, 0.31]	0.38 [0.11, 0.85]	0.77 [0.23, 1.00]	0.27 [0.21, 0.31]	0.86 [0.71, 1.00]
lr0.001_wd0.005_h400_d1	0.66 [0.58, 0.74]	0.38 [0.32, 0.53]	0.43 [0.38, 0.48]	0.64 [0.59, 0.69]	0.25 [0.16, 0.32]	0.57 [0.30, 0.76]	0.71 [0.46, 1.00]	0.32 [0.29, 0.35]	0.90 [0.82, 1.00]
lr0.001_wd0.005_h512_d1	0.68 [0.63, 0.75]	0.39 [0.33, 0.51]	0.42 [0.35, 0.44]	0.61 [0.50, 0.65]	0.20 [0.00, 0.30]	0.46 [0.00, 0.87]	0.76 [0.42, 1.00]	0.31 [0.21, 0.45]	0.71 [0.00, 0.94]
lr0.001_wd0.005_h256_d2	0.71 [0.65, 0.78]	0.41 [0.33, 0.51]	0.39 [0.26, 0.54]	0.58 [0.50, 0.71]	0.16 [0.00, 0.41]	0.46 [0.00, 0.85]	0.71 [0.23, 1.00]	0.30 [0.22, 0.50]	0.70 [0.00, 1.00]
lr0.001_wd0.005_h400_d2	0.68 [0.65, 0.77]	0.37 [0.31, 0.47]	0.39 [0.24, 0.52]	0.59 [0.47, 0.75]	0.16 [-0.05, 0.40]	0.50 [0.00, 0.78]	0.69 [0.31, 1.00]	0.29 [0.19, 0.38]	0.69 [0.00, 0.96]
lr0.001_wd0.005_h512_d2	0.67 [0.58, 0.75]	0.43 [0.33, 0.53]	0.36 [0.31, 0.39]	0.55 [0.50, 0.59]	0.09 [0.00, 0.15]	0.34 [0.00, 0.67]	0.76 [0.42, 1.00]	0.25 [0.22, 0.29]	0.68 [0.00, 0.88]
lr0.001_wd0.01_h256_d1	0.67 [0.59, 0.76]	0.42 [0.31, 0.55]	0.40 [0.34, 0.50]	0.58 [0.50, 0.72]	0.14 [0.00, 0.37]	0.32 [0.00, 0.59]	0.84 [0.54, 1.00]	0.26 [0.21, 0.34]	0.73 [0.00, 1.00]
lr0.001_wd0.01_h400_d1	0.69 [0.59, 0.76]	0.42 [0.32, 0.59]	0.40 [0.12, 0.49]	0.65 [0.51, 0.71]	0.26 [0.03, 0.35]	0.56 [0.43, 0.93]	0.74 [0.08, 0.92]	0.31 [0.25, 0.33]	0.92 [0.80, 0.96]
lr0.001_wd0.01_h512_d1	0.66 [0.60, 0.77]	0.40 [0.29, 0.49]	0.37 [0.26, 0.49]	0.56 [0.43, 0.71]	0.09 [-0.27, 0.34]	0.37 [0.02, 0.85]	0.75 [0.23, 1.00]	0.26 [0.18, 0.34]	0.79 [0.33, 1.00]
lr0.001_wd0.01_h256_d2	0.68 [0.59, 0.72]	0.41 [0.34, 0.53]	0.38 [0.32, 0.45]	0.57 [0.50, 0.65]	0.14 [0.00, 0.30]	0.44 [0.00, 0.85]	0.71 [0.38, 1.00]	0.28 [0.22, 0.42]	0.70 [0.00, 1.00]
lr0.001_wd0.01_h400_d2	0.70 [0.62, 0.76]	0.42 [0.33, 0.54]	0.42 [0.36, 0.46]	0.63 [0.56, 0.67]	0.23 [0.10, 0.30]	0.53 [0.41, 0.89]	0.73 [0.38, 0.92]	0.33 [0.24, 0.50]	0.89 [0.84, 0.95]
lr0.001_wd0.01_h512_d2	0.69 [0.64, 0.76]	0.43 [0.34, 0.51]	0.39 [0.26, 0.50]	0.60 [0.50, 0.72]	0.18 [0.00, 0.37]	0.52 [0.00, 0.85]	0.69 [0.23, 1.00]	0.30 [0.21, 0.39]	0.70 [0.00, 0.96]
lr0.0001_wd0.0_h1024_d1	0.65 [0.55, 0.71]	0.38 [0.33, 0.47]	0.39 [0.36, 0.48]	0.60 [0.54, 0.69]	0.17 [0.09, 0.32]	0.53 [0.15, 0.80]	0.67 [0.38, 0.92]	0.30 [0.24, 0.36]	0.86 [0.82, 0.93]
lr0.0001_wd0.0_h2048_d1	0.66 [0.60, 0.72]	0.42 [0.34, 0.50]	0.40 [0.34, 0.49]	0.58 [0.50, 0.68]	0.16 [0.00, 0.31]	0.28 [0.00, 0.67]	0.89 [0.69, 1.00]	0.27 [0.21, 0.38]	0.74 [0.00, 1.00]
lr0.0001_wd0.0_h1024_d2	0.66 [0.61, 0.70]	0.38 [0.33, 0.45]	0.32 [0.22, 0.36]	0.49 [0.43, 0.55]	-0.04 [-0.27, 0.09]	0.21 [0.00, 0.76]	0.78 [0.23, 1.00]	0.21 [0.18, 0.23]	0.39 [0.00, 0.86]
lr0.0001_wd0.0_h2048_d2	0.66 [0.57, 0.74]	0.42 [0.34, 0.55]	0.37 [0.32, 0.41]	0.55 [0.50, 0.62]	0.09 [0.00, 0.23]	0.37 [0.00, 0.82]	0.73 [0.42, 1.00]	0.27 [0.21, 0.38]	0.50 [0.00, 0.87]
lr0.0001_wd0.0001_h1024_d1	0.65 [0.57, 0.72]	0.40 [0.31, 0.50]	0.39 [0.28, 0.49]	0.60 [0.52, 0.68]	0.17 [0.04, 0.31]	0.56 [0.41, 0.74]	0.64 [0.31, 0.85]	0.29 [0.25, 0.38]	0.86 [0.79, 0.90]
lr0.0001_wd0.0001_h2048_d1	0.65 [0.56, 0.70]	0.40 [0.33, 0.52]	0.34 [0.27, 0.39]	0.54 [0.44, 0.61]	0.05 [-0.20, 0.18]	0.47 [0.04, 0.87]	0.61 [0.23, 0.92]	0.27 [0.19, 0.33]	0.76 [0.50, 0.84]
lr0.0001_wd0.0001_h1024_d2	0.67 [0.57, 0.72]	0.39 [0.32, 0.53]	0.36 [0.18, 0.46]	0.59 [0.50, 0.67]	0.16 [0.00, 0.32]	0.60 [0.35, 0.85]	0.59 [0.15, 1.00]	0.27 [0.22, 0.30]	0.87 [0.78, 1.00]
lr0.0001_wd0.0001_h2048_d2	0.67 [0.62, 0.72]	0.40 [0.32, 0.47]	0.38 [0.34, 0.44]	0.55 [0.50, 0.66]	0.11 [0.00, 0.27]	0.24 [0.00, 0.54]	0.87 [0.62, 1.00]	0.25 [0.21, 0.30]	0.71 [0.00, 1.00]
lr0.0001_wd0.001_h1024_d1	0.64 [0.54, 0.70]	0.36 [0.29, 0.47]	0.38 [0.31, 0.43]	0.56 [0.50, 0.64]	0.11 [0.00, 0.23]	0.36 [0.00, 0.78]	0.77 [0.33, 1.00]	0.26 [0.22, 0.30]	0.52 [0.00, 0.89]
lr0.0001_wd0.001_h2048_d1	0.65 [0.58, 0.70]	0.36 [0.32, 0.44]	0.30 [0.00, 0.43]	0.57 [0.47, 0.62]	0.11 [-0.12, 0.21]	0.72 [0.48, 0.93]	0.42 [0.00, 0.77]	0.25 [0.00, 0.33]	0.83 [0.78, 0.88]
lr0.0001_wd0.001_h1024_d2	0.67 [0.61, 0.72]	0.38 [0.31, 0.45]	0.38 [0.34, 0.43]	0.57 [0.50, 0.63]	0.16 [0.00, 0.31]	0.49 [0.00, 0.93]	0.65 [0.31, 1.00]	0.34 [0.21, 0.57]	0.70 [0.00, 1.00]
lr0.0001_wd0.001_h2048_d2	0.66 [0.59, 0.70]	0.41 [0.34, 0.49]	0.40 [0.25, 0.51]	0.61 [0.51, 0.71]	0.23 [0.07, 0.35]	0.46 [0.02, 0.96]	0.77 [0.17, 1.00]	0.33 [0.22, 0.50]	0.93 [0.81, 1.00]
lr0.0003_wd0.0_h1024_d1	0.66 [0.59, 0.73]	0.40 [0.32, 0.49]	0.37 [0.28, 0.41]	0.55 [0.41, 0.61]	0.09 [-0.25, 0.21]	0.34 [0.07, 0.83]	0.76 [0.38, 0.92]	0.26 [0.17, 0.38]	0.81 [0.50, 0.92]
lr0.0003_wd0.0_h2048_d1	0.66 [0.60, 0.71]	0.42 [0.32, 0.56]	0.37 [0.26, 0.44]	0.59 [0.54, 0.66]	0.16 [0.07, 0.26]	0.54 [0.28, 0.85]	0.63 [0.23, 0.92]	0.29 [0.24, 0.33]	0.86 [0.80, 0.93]
lr0.0003_wd0.0_h1024_d2	0.68 [0.59, 0.77]	0.39 [0.29, 0.49]	0.41 [0.34, 0.55]	0.60 [0.50, 0.73]	0.16 [0.00, 0.40]	0.49 [0.00, 0.76]	0.70 [0.54, 1.00]	0.30 [0.22, 0.45]	0.68 [0.00, 0.90]
lr0.0003_wd0.0_h2048_d2	0.69 [0.59, 0.76]	0.44 [0.35, 0.54]	0.37 [0.20, 0.45]	0.59 [0.52, 0.66]	0.17 [0.06, 0.26]	0.48 [0.24, 0.89]	0.70 [0.15, 1.00]	0.28 [0.23, 0.32]	0.88 [0.79, 1.00]
lr0.0003_wd0.0001_h1024_d1	0.68 [0.63, 0.74]	0.42 [0.31, 0.57]	0.34 [0.00, 0.43]	0.60 [0.50, 0.64]	0.19 [0.00, 0.27]	0.48 [0.22, 1.00]	0.72 [0.00, 1.00]	0.23 [0.00, 0.30]	0.92 [0.79, 1.00]
lr0.0003_wd0.0001_h2048_d1	0.69 [0.63, 0.74]	0.35 [0.27, 0.42]	0.32 [0.00, 0.46]	0.57 [0.50, 0.67]	0.13 [0.00, 0.29]	0.47 [0.00, 1.00]	0.66 [0.00, 1.00]	0.23 [0.00, 0.36]	0.71 [0.00, 1.00]
lr0.0003_wd0.0001_h1024_d2	0.66 [0.58, 0.77]	0.40 [0.34, 0.49]	0.36 [0.31, 0.41]	0.55 [0.45, 0.60]	0.11 [-0.15, 0.23]	0.27 [0.04, 0.85]	0.83 [0.31, 1.00]	0.26 [0.19, 0.36]	0.88 [0.60, 1.00]
lr0.0003_wd0.0001_h2048_d2	0.67 [0.62, 0.75]	0.39 [0.31, 0.53]	0.40 [0.33, 0.47]	0.61 [0.57, 0.68]	0.20 [0.12, 0.30]	0.54 [0.26, 0.85]	0.68 [0.31, 0.92]	0.31 [0.26, 0.36]	0.87 [0.81, 0.92]
lr0.0003_wd0.001_h1024_d1	0.66 [0.59, 0.74]	0.40 [0.29, 0.65]	0.36 [0.26, 0.44]	0.56 [0.54, 0.65]	0.12 [0.07, 0.26]	0.41 [0.16, 0.85]	0.72 [0.23, 0.92]	0.26 [0.22, 0.30]	0.86 [0.80, 0.94]
lr0.0003_wd0.001_h2048_d1	0.68 [0.62, 0.75]	0.40 [0.30, 0.54]	0.29 [0.11, 0.53]	0.57 [0.49, 0.74]	0.11 [-0.01, 0.39]	0.71 [0.33, 0.91]	0.42 [0.08, 0.83]	0.27 [0.20, 0.38]	0.83 [0.78, 0.94]
lr0.0003_wd0.001_h1024_d2	0.69 [0.61, 0.74]	0.38 [0.27, 0.51]	0.36 [0.27, 0.47]	0.57 [0.51, 0.68]	0.14 [0.03, 0.30]	0.48 [0.02, 0.87]	0.66 [0.23, 1.00]	0.27 [0.22, 0.33]	0.89 [0.80, 1.00]
lr0.0003_wd0.001_h2048_d2	0.70 [0.61, 0.86]	0.43 [0.32, 0.64]	0.41 [0.38, 0.50]	0.62 [0.57, 0.72]	0.20 [0.12, 0.37]	0.50 [0.30, 0.78]	0.73 [0.46, 0.92]	0.30 [0.26, 0.38]	0.88 [0.84, 0.96]
lr0.001_wd0.0_h1024_d1	0.68 [0.60, 0.75]	0.42 [0.33, 0.50]	0.39 [0.30, 0.43]	0.59 [0.45, 0.63]	0.15 [-0.10, 0.23]	0.48 [0.15, 0.76]	0.70 [0.46, 0.92]	0.29 [0.19, 0.35]	0.84 [0.70, 0.93]
lr0.001_wd0.0_h2048_d1	0.69 [0.58, 0.75]	0.38 [0.31, 0.45]	0.40 [0.36, 0.46]	0.58 [0.53, 0.66]	0.17 [0.12, 0.27]	0.37 [0.07, 0.70]	0.80 [0.54, 1.00]	0.28 [0.22, 0.35]	0.92 [0.84, 1.00]
lr0.001_wd0.0_h1024_d2	0.70 [0.60, 0.79]	0.39 [0.32, 0.48]	0.37 [0.30, 0.42]	0.59 [0.55, 0.64]	0.17 [0.09, 0.24]	0.56 [0.33, 0.87]	0.62 [0.31, 0.92]	0.30 [0.26, 0.40]	0.86 [0.81, 0.94]
lr0.001_wd0.0_h2048_d2	0.71 [0.62, 0.83]	0.39 [0.28, 0.61]	0.38 [0.20, 0.56]	0.61 [0.52, 0.78]	0.20 [0.06, 0.46]	0.59 [0.11, 0.89]	0.62 [0.15, 1.00]	0.30 [0.24, 0.41]	0.89 [0.79, 1.00]
lr0.001_wd0.0001_h1024_d1	0.69 [0.57, 0.75]	0.37 [0.29, 0.48]	0.31 [0.00, 0.43]	0.56 [0.49, 0.64]	0.11 [-0.07, 0.23]	0.57 [0.04, 0.98]	0.56 [0.00, 1.00]	0.23 [0.00, 0.36]	0.87 [0.78, 1.00]
lr0.001_wd0.0001_h2048_d1	0.67 [0.58, 0.76]	0.39 [0.32, 0.45]	0.37 [0.33, 0.42]	0.56 [0.50, 0.62]	0.13 [0.01, 0.20]	0.37 [0.13, 0.87]	0.75 [0.31, 1.00]	0.28 [0.21, 0.40]	0.87 [0.80, 1.00]
lr0.001_wd0.0001_h1024_d2	0.69 [0.60, 0.76]	0.41 [0.33, 0.58]	0.38 [0.30, 0.48]	0.58 [0.48, 0.69]	0.13 [-0.07, 0.33]	0.47 [0.04, 0.78]	0.70 [0.31, 0.92]	0.27 [0.20, 0.32]	0.83 [0.67, 0.95]

Supplementary Table 40: Grid search results on the classification task on SYSMH external validation dataset (choosing 60% as the threshold for clinical variables selection). Reported metrics are AUC, AUPRC, F1, Balanced Accuracy, MCC, Specificity, Sensitivity, PPV, and NPV.

Config	AUC	AUPRC	F1	Balanced_Accuracy	MCC	Specificity	Sensitivity	PPV	NPV
lr0.001_wd0.0001_h2048_d2	0.69 [0.62, 0.75]	0.37 [0.32, 0.46]	0.39 [0.31, 0.47]	0.60 [0.56, 0.67]	0.20 [0.11, 0.29]	0.52 [0.11, 0.78]	0.68 [0.33, 1.00]	0.30 [0.23, 0.38]	0.90 [0.82, 1.00]
lr0.001_wd0.001_h1024_d1	0.67 [0.58, 0.71]	0.39 [0.30, 0.52]	0.36 [0.29, 0.50]	0.57 [0.48, 0.70]	0.12 [-0.03, 0.34]	0.55 [0.22, 0.78]	0.59 [0.31, 0.85]	0.28 [0.20, 0.40]	0.83 [0.77, 0.89]
lr0.001_wd0.001_h2048_d1	0.70 [0.58, 0.75]	0.41 [0.37, 0.51]	0.44 [0.37, 0.53]	0.63 [0.51, 0.72]	0.25 [0.07, 0.37]	0.62 [0.02, 0.87]	0.65 [0.42, 1.00]	0.37 [0.22, 0.45]	0.89 [0.85, 1.00]
lr0.001_wd0.001_h1024_d2	0.67 [0.60, 0.76]	0.37 [0.33, 0.39]	0.31 [0.00, 0.46]	0.58 [0.47, 0.68]	0.13 [-0.09, 0.31]	0.64 [0.09, 1.00]	0.51 [0.00, 0.92]	0.26 [0.00, 0.40]	0.81 [0.67, 0.95]
lr0.001_wd0.001_h2048_d2	0.67 [0.59, 0.75]	0.42 [0.32, 0.52]	0.44 [0.34, 0.56]	0.63 [0.50, 0.75]	0.23 [0.00, 0.43]	0.53 [0.00, 0.76]	0.73 [0.46, 1.00]	0.33 [0.21, 0.45]	0.70 [0.00, 0.92]
lr0.0001_wd0.0_h256_d3	0.67 [0.63, 0.76]	0.39 [0.35, 0.42]	0.37 [0.29, 0.53]	0.57 [0.50, 0.71]	0.14 [0.00, 0.38]	0.51 [0.00, 0.89]	0.64 [0.23, 1.00]	0.33 [0.21, 0.47]	0.50 [0.00, 0.88]
lr0.0001_wd0.0_h400_d3	0.68 [0.60, 0.75]	0.39 [0.30, 0.48]	0.41 [0.34, 0.48]	0.59 [0.50, 0.70]	0.18 [0.00, 0.34]	0.31 [0.00, 0.85]	0.88 [0.46, 1.00]	0.30 [0.21, 0.46]	0.56 [0.00, 1.00]
lr0.0001_wd0.0_h512_d3	0.68 [0.61, 0.74]	0.40 [0.35, 0.46]	0.39 [0.34, 0.48]	0.58 [0.50, 0.68]	0.13 [0.00, 0.30]	0.33 [0.00, 0.73]	0.82 [0.42, 1.00]	0.27 [0.21, 0.34]	0.53 [0.00, 0.94]
lr0.0001_wd0.0_h256_d4	0.69 [0.64, 0.74]	0.41 [0.36, 0.48]	0.40 [0.34, 0.48]	0.57 [0.50, 0.70]	0.14 [0.00, 0.34]	0.29 [0.00, 0.78]	0.86 [0.46, 1.00]	0.27 [0.21, 0.38]	0.54 [0.00, 0.96]
lr0.0001_wd0.0_h400_d4	0.70 [0.64, 0.75]	0.40 [0.35, 0.49]	0.40 [0.33, 0.48]	0.58 [0.50, 0.69]	0.15 [0.00, 0.32]	0.43 [0.00, 0.85]	0.74 [0.38, 1.00]	0.30 [0.21, 0.46]	0.52 [0.00, 0.93]
lr0.0001_wd0.0_h512_d4	0.69 [0.60, 0.76]	0.38 [0.31, 0.46]	0.39 [0.34, 0.47]	0.56 [0.50, 0.69]	0.11 [0.00, 0.32]	0.13 [0.00, 0.47]	0.98 [0.92, 1.00]	0.24 [0.21, 0.31]	0.39 [0.00, 1.00]
lr0.0001_wd0.0001_h256_d3	0.68 [0.63, 0.74]	0.42 [0.31, 0.54]	0.40 [0.34, 0.50]	0.58 [0.50, 0.71]	0.15 [0.00, 0.34]	0.37 [0.00, 0.85]	0.80 [0.38, 1.00]	0.30 [0.21, 0.42]	0.52 [0.00, 0.91]
lr0.0001_wd0.0001_h400_d3	0.68 [0.61, 0.73]	0.39 [0.33, 0.50]	0.35 [0.24, 0.47]	0.55 [0.50, 0.68]	0.09 [0.00, 0.30]	0.43 [0.00, 0.83]	0.67 [0.23, 1.00]	0.27 [0.21, 0.33]	0.50 [0.00, 0.92]
lr0.0001_wd0.0001_h512_d3	0.67 [0.62, 0.75]	0.41 [0.35, 0.53]	0.41 [0.34, 0.51]	0.59 [0.50, 0.71]	0.15 [0.00, 0.35]	0.37 [0.00, 0.78]	0.81 [0.58, 1.00]	0.29 [0.21, 0.41]	0.52 [0.00, 0.91]
lr0.0001_wd0.0001_h256_d4	0.68 [0.64, 0.74]	0.39 [0.32, 0.51]	0.31 [0.00, 0.44]	0.55 [0.50, 0.65]	0.09 [0.00, 0.24]	0.36 [0.00, 1.00]	0.74 [0.00, 1.00]	0.20 [0.00, 0.31]	0.52 [0.00, 0.93]
lr0.0001_wd0.0001_h400_d4	0.68 [0.62, 0.72]	0.40 [0.36, 0.45]	0.43 [0.34, 0.59]	0.60 [0.50, 0.77]	0.18 [0.00, 0.46]	0.32 [0.00, 0.76]	0.89 [0.77, 1.00]	0.29 [0.21, 0.48]	0.54 [0.00, 0.92]
lr0.0001_wd0.0001_h512_d4	0.67 [0.63, 0.76]	0.42 [0.38, 0.47]	0.40 [0.35, 0.50]	0.58 [0.50, 0.68]	0.15 [0.00, 0.35]	0.40 [0.00, 0.83]	0.76 [0.54, 1.00]	0.30 [0.22, 0.47]	0.69 [0.00, 0.92]
lr0.0001_wd0.001_h256_d3	0.67 [0.63, 0.73]	0.41 [0.34, 0.47]	0.36 [0.34, 0.38]	0.54 [0.50, 0.59]	0.08 [0.00, 0.18]	0.34 [0.00, 0.80]	0.75 [0.38, 1.00]	0.26 [0.21, 0.36]	0.50 [0.00, 0.88]
lr0.0001_wd0.001_h400_d3	0.68 [0.63, 0.75]	0.41 [0.32, 0.46]	0.35 [0.32, 0.39]	0.53 [0.50, 0.57]	0.06 [0.00, 0.15]	0.21 [0.00, 0.83]	0.85 [0.31, 1.00]	0.24 [0.21, 0.33]	0.34 [0.00, 0.91]
lr0.0001_wd0.001_h512_d3	0.69 [0.62, 0.76]	0.42 [0.37, 0.49]	0.40 [0.34, 0.46]	0.58 [0.50, 0.67]	0.14 [0.00, 0.28]	0.37 [0.00, 0.67]	0.79 [0.62, 1.00]	0.28 [0.21, 0.35]	0.52 [0.00, 0.88]
lr0.0001_wd0.001_h256_d4	0.66 [0.61, 0.71]	0.38 [0.33, 0.45]	0.38 [0.34, 0.42]	0.56 [0.50, 0.63]	0.11 [0.00, 0.27]	0.34 [0.00, 0.87]	0.78 [0.38, 1.00]	0.28 [0.21, 0.45]	0.51 [0.00, 0.88]
lr0.0001_wd0.001_h400_d4	0.68 [0.61, 0.76]	0.39 [0.32, 0.54]	0.36 [0.27, 0.43]	0.56 [0.50, 0.63]	0.13 [0.00, 0.27]	0.32 [0.00, 0.87]	0.80 [0.23, 1.00]	0.26 [0.22, 0.33]	0.73 [0.00, 1.00]
lr0.0001_wd0.001_h512_d4	0.68 [0.64, 0.73]	0.38 [0.34, 0.44]	0.35 [0.20, 0.43]	0.55 [0.50, 0.65]	0.09 [0.00, 0.26]	0.35 [0.00, 0.89]	0.75 [0.15, 1.00]	0.25 [0.21, 0.29]	0.52 [0.00, 0.94]
lr0.0003_wd0.0_h256_d3	0.69 [0.61, 0.74]	0.38 [0.32, 0.44]	0.36 [0.20, 0.49]	0.57 [0.50, 0.71]	0.12 [0.00, 0.34]	0.35 [0.00, 0.89]	0.78 [0.15, 1.00]	0.26 [0.21, 0.34]	0.53 [0.00, 0.93]
lr0.0003_wd0.0_h400_d3	0.69 [0.64, 0.73]	0.41 [0.34, 0.56]	0.38 [0.34, 0.43]	0.57 [0.50, 0.63]	0.13 [0.00, 0.27]	0.25 [0.00, 0.78]	0.88 [0.38, 1.00]	0.26 [0.21, 0.33]	0.56 [0.00, 1.00]
lr0.0003_wd0.0_h512_d3	0.70 [0.62, 0.78]	0.43 [0.34, 0.54]	0.40 [0.34, 0.47]	0.58 [0.50, 0.68]	0.15 [0.00, 0.30]	0.43 [0.00, 0.80]	0.73 [0.38, 1.00]	0.30 [0.21, 0.36]	0.51 [0.00, 0.89]
lr0.0003_wd0.0_h256_d4	0.69 [0.63, 0.75]	0.39 [0.36, 0.47]	0.43 [0.34, 0.53]	0.61 [0.50, 0.72]	0.21 [0.00, 0.37]	0.32 [0.00, 0.67]	0.91 [0.77, 1.00]	0.29 [0.21, 0.40]	0.75 [0.00, 1.00]
lr0.0003_wd0.0_h400_d4	0.68 [0.61, 0.76]	0.43 [0.37, 0.50]	0.39 [0.34, 0.43]	0.57 [0.50, 0.65]	0.12 [0.00, 0.27]	0.34 [0.00, 0.87]	0.80 [0.38, 1.00]	0.29 [0.21, 0.45]	0.52 [0.00, 0.91]
lr0.0003_wd0.0_h512_d4	0.69 [0.60, 0.80]	0.42 [0.32, 0.53]	0.43 [0.34, 0.51]	0.61 [0.50, 0.72]	0.19 [0.00, 0.37]	0.37 [0.00, 0.74]	0.86 [0.62, 1.00]	0.30 [0.21, 0.40]	0.55 [0.00, 0.96]
lr0.0003_wd0.0001_h256_d3	0.70 [0.62, 0.74]	0.40 [0.35, 0.46]	0.37 [0.34, 0.43]	0.53 [0.50, 0.62]	0.08 [0.00, 0.25]	0.08 [0.00, 0.24]	0.98 [0.92, 1.00]	0.23 [0.21, 0.27]	0.58 [0.00, 1.00]
lr0.0003_wd0.0001_h400_d3	0.68 [0.60, 0.76]	0.43 [0.35, 0.50]	0.39 [0.34, 0.44]	0.57 [0.50, 0.64]	0.12 [0.00, 0.25]	0.29 [0.00, 0.74]	0.84 [0.54, 1.00]	0.27 [0.21, 0.37]	0.52 [0.00, 0.91]
lr0.0003_wd0.0001_h512_d3	0.67 [0.57, 0.73]	0.40 [0.35, 0.48]	0.37 [0.27, 0.45]	0.57 [0.50, 0.67]	0.14 [0.00, 0.28]	0.38 [0.00, 0.87]	0.76 [0.23, 1.00]	0.27 [0.22, 0.33]	0.71 [0.00, 1.00]
lr0.0003_wd0.0001_h256_d4	0.70 [0.58, 0.76]	0.42 [0.38, 0.49]	0.35 [0.11, 0.47]	0.56 [0.49, 0.68]	0.11 [-0.01, 0.30]	0.38 [0.00, 0.91]	0.75 [0.08, 1.00]	0.25 [0.20, 0.36]	0.53 [0.00, 1.00]
lr0.0003_wd0.0001_h400_d4	0.67 [0.58, 0.74]	0.39 [0.33, 0.50]	0.42 [0.34, 0.53]	0.60 [0.50, 0.72]	0.17 [0.00, 0.37]	0.30 [0.00, 0.74]	0.89 [0.69, 1.00]	0.29 [0.21, 0.43]	0.55 [0.00, 0.93]
lr0.0003_wd0.0001_h512_d4	0.69 [0.62, 0.76]	0.41 [0.32, 0.50]	0.34 [0.10, 0.46]	0.56 [0.47, 0.68]	0.09 [-0.07, 0.31]	0.33 [0.00, 0.87]	0.78 [0.08, 1.00]	0.23 [0.14, 0.31]	0.53 [0.00, 0.95]
lr0.0003_wd0.001_h256_d3	0.68 [0.61, 0.77]	0.35 [0.29, 0.52]	0.26 [0.00, 0.40]	0.55 [0.50, 0.60]	0.11 [0.00, 0.22]	0.73 [0.20, 1.00]	0.37 [0.00, 1.00]	0.23 [0.00, 0.36]	0.84 [0.78, 1.00]
lr0.0003_wd0.001_h400_d3	0.66 [0.56, 0.72]	0.38 [0.31, 0.48]	0.36 [0.27, 0.43]	0.57 [0.50, 0.64]	0.14 [0.00, 0.29]	0.63 [0.00, 0.87]	0.51 [0.31, 1.00]	0.32 [0.22, 0.45]	0.66 [0.00, 0.85]
lr0.0003_wd0.001_h512_d3	0.70 [0.60, 0.76]	0.42 [0.35, 0.54]	0.39 [0.32, 0.53]	0.58 [0.50, 0.72]	0.15 [0.00, 0.39]	0.38 [0.00, 0.83]	0.78 [0.31, 1.00]	0.29 [0.21, 0.44]	0.53 [0.00, 0.93]
lr0.0003_wd0.001_h256_d4	0.69 [0.64, 0.74]	0.39 [0.31, 0.45]	0.38 [0.32, 0.44]	0.58 [0.50, 0.66]	0.15 [0.00, 0.26]	0.48 [0.00, 0.87]	0.68 [0.31, 1.00]	0.29 [0.22, 0.40]	0.69 [0.00, 0.94]
lr0.0003_wd0.001_h400_d4	0.69 [0.59, 0.74]	0.42 [0.36, 0.48]	0.39 [0.34, 0.47]	0.57 [0.50, 0.67]	0.13 [0.00, 0.29]	0.23 [0.00, 0.72]	0.91 [0.62, 1.00]	0.26 [0.21, 0.38]	0.56 [0.00, 1.00]
lr0.0003_wd0.001_h512_d4	0.70 [0.59, 0.78]	0.41 [0.34, 0.53]	0.38 [0.29, 0.45]	0.58 [0.50, 0.68]	0.15 [0.00, 0.32]	0.31 [0.00, 0.89]	0.85 [0.23, 1.00]	0.28 [0.21, 0.38]	0.56 [0.00, 1.00]
lr0.001_wd0.0_h256_d3	0.70 [0.61, 0.74]	0.40 [0.30, 0.51]	0.38 [0.27, 0.48]	0.61 [0.54, 0.69]	0.19 [0.07, 0.32]	0.57 [0.26, 0.87]	0.64 [0.23, 0.92]	0.30 [0.24, 0.33]	0.87 [0.80, 0.93]
lr0.001_wd0.0_h400_d3	0.71 [0.66, 0.77]	0.40 [0.33, 0.46]	0.43 [0.36, 0.52]	0.64 [0.57, 0.74]	0.24 [0.12, 0.41]	0.52 [0.37, 0.67]	0.76 [0.50, 1.00]	0.31 [0.26, 0.38]	0.90 [0.84, 1.00]
lr0.001_wd0.0_h512_d3	0.69 [0.62, 0.78]	0.39 [0.31, 0.46]	0.42 [0.34, 0.47]	0.62 [0.50, 0.69]	0.21 [0.00, 0.31]	0.41 [0.00, 0.76]	0.83 [0.46, 1.00]	0.30 [0.21, 0.35]	0.73 [0.00, 1.00]
lr0.001_wd0.0_h256_d4	0.70 [0.59, 0.79]	0.42 [0.35, 0.52]	0.45 [0.36, 0.57]	0.64 [0.50, 0.75]	0.25 [0.00, 0.44]	0.38 [0.00, 0.74]	0.90 [0.75, 1.00]	0.31 [0.22, 0.45]	0.76 [0.00, 1.00]
lr0.001_wd0.0_h400_d4	0.70 [0.62, 0.83]	0.39 [0.32, 0.56]	0.43 [0.33, 0.62]	0.62 [0.49, 0.83]	0.20 [-0.03, 0.54]	0.43 [0.07, 0.78]	0.80 [0.46, 1.00]	0.31 [0.20, 0.44]	0.86 [0.75, 1.00]
lr0.001_wd0.0_h512_d4	0.71 [0.62, 0.81]	0.43 [0.34, 0.57]	0.42 [0.34, 0.55]	0.60 [0.50, 0.77]	0.17 [0.00, 0.44]	0.33 [0.00, 0.62]	0.88 [0.69, 1.00]	0.29 [0.21, 0.39]	0.54 [0.00, 0.97]
lr0.001_wd0.0001_h256_d3	0.70 [0.59, 0.78]	0.39 [0.36, 0.47]	0.41 [0.30, 0.48]	0.63 [0.55, 0.70]	0.23 [0.09, 0.35]	0.55 [0.39, 0.78]	0.71 [0.31, 1.00]	0.30 [0.28, 0.33]	0.90 [0.80, 1.00]
lr0.001_wd0.0001_h400_d3	0.68 [0.62, 0.76]	0.40 [0.31, 0.55]	0.40 [0.35, 0.47]	0.57 [0.48, 0.68]	0.11 [-0.06, 0.30]	0.35 [0.00, 0.76]	0.79 [0.58, 1.00]	0.28 [0.21, 0.39]	0.65 [0.00, 0.89]
lr0.001_wd0.0001_h512_d3	0.69 [0.63, 0.79]	0.39 [0.32, 0.48]	0.40 [0.15, 0.50]	0.63 [0.54, 0.73]	0.28 [0.14, 0.37]	0.55 [0.09, 1.00]	0.72 [0.08, 1.00]	0.45 [0.24, 1.00]	0.92 [0.81, 1.00]
lr0.001_wd0.0001_h256_d4	0.68 [0.57, 0.78]	0.39 [0.32, 0.53]	0.41 [0.32, 0.50]	0.61 [0.54, 0.71]	0.20 [0.07, 0.34]	0.47 [0.15, 0.72]	0.74 [0.50, 1.00]	0.29 [0.24, 0.38]	0.90 [0.82, 1.00]
lr0.001_wd0.0001_h400_d4	0.67 [0.60, 0.78]	0.37 [0.29, 0.53]	0.40 [0.37, 0.46]	0.59 [0.55, 0.67]	0.17 [0.10, 0.29]	0.35 [0.11, 0.70]	0.82 [0.50, 1.00]	0.27 [0.23, 0.31]	0.90 [0.84, 1.00]
lr0.001_wd0.0001_h512_d4	0.69 [0.59, 0.76]	0.41 [0.35, 0.48]	0.41 [0.36, 0.48]	0.61 [0.50, 0.68]	0.18 [0.00, 0.31]	0.50 [0.00, 0.82]	0.71 [0.50, 1.00]	0.31 [0.22, 0.43]	0.69 [0.00, 0.90]
lr0.001_wd0.001_h256_d3	0.68 [0.63, 0.75]	0.41 [0.31, 0.57]	0.41 [0.34, 0.49]	0.59 [0.50, 0.69]	0.15 [0.00, 0.31]	0.30 [0.00, 0.61]	0.87 [0.75, 1.00]	0.27 [0.21, 0.36]	0.73 [0.00, 1.00]
lr0.001_wd0.001_h400_d3	0.71 [0.63, 0.85]	0.43 [0.33, 0.60]	0.39 [0.34, 0.44]	0.56 [0.50, 0.65]	0.11 [0.00, 0.25]	0.30 [0.00, 0.71]	0.82 [0.54, 1.00]	0.26 [0.21, 0.35]	0.52 [0.00, 0.88]
lr0.001_wd0.001_h512_d3	0.69 [0.61, 0.75]	0.38 [0.34, 0.47]	0.42 [0.35, 0.48]	0.63 [0.59, 0.68]	0.23 [0.18, 0.30]	0.53 [0.28, 0.85]	0.72 [0.33, 0.92]	0.31 [0.27, 0.36]	0.89 [0.83, 0.95]
lr0.001_wd0.001_h256_d4	0.71 [0.64, 0.79]	0.41 [0.33, 0.49]	0.43 [0.34, 0.47]	0.63 [0.50, 0.70]	0.21 [0.00, 0.35]	0.44 [0.00, 0.70]	0.82 [0.54, 1.00]	0.30 [0.21, 0.33]	0.73 [0.00, 1.00]
lr0.001_wd0.001_h400_d4	0.70 [0.63, 0.77]	0.38 [0.26, 0.57]	0.41 [0.24, 0.51]	0.62 [0.48, 0.72]	0.20 [-0.03, 0.36]	0.58 [0.48, 0.63]	0.66 [0.33, 0.85]	0.30 [0.19, 0.37]	0.87 [0.78, 0.93]
lr0.001_wd0.001_h512_d4	0.68 [0.62, 0.74]	0.40 [0.28, 0.52]	0.40 [0.31, 0.48]	0.61 [0.50, 0.72]	0.19 [0.00, 0.36]	0.35 [0.00, 0.60]	0.87 [0.69, 1.00]	0.29 [0.21, 0.37]	0.55 [0.00, 0.93]

Supplementary Table 41: Grid search results on the classification task on SYSMH external validation dataset (choosing 60% as the threshold for clinical variables selection). Reported metrics are AUC, AUPRC, F1, Balanced Accuracy, MCC, Specificity, Sensitivity, PPV, and NPV.

References

[1] MacQueen, J.: Some methods for classification and analysis of multivariate observations. In: Proceedings of the Fifth Berkeley Symposium on Mathematical Statistics and Probability, vol. 1, pp. 281–297 (1967). University of California Press

[2] Ward Jr, J.H.: Hierarchical grouping to optimize an objective function. Journal of the American Statistical Association **58**(301), 236–244 (1963)

[3] Ng, A.Y., Jordan, M.I., Weiss, Y.: On spectral clustering: Analysis and an algorithm. In: Advances in Neural Information Processing Systems (NeurIPS), vol. 14, pp. 849–856 (2002)

[4] Reynolds, D.A.: Gaussian mixture models. In: Encyclopedia of Biometrics, pp. 659–663. Springer, New York, NY (2009)

[5] Ester, M., Kriegel, H.-P., Sander, J., Xu, X.: A density-based algorithm for discovering clusters in large spatial databases with noise. In: Proceedings of the Second International Conference on Knowledge Discovery and Data Mining (KDD), pp. 226–231 (1996). AAAI Press

[6] Rousseeuw, P.J.: Silhouettes: A graphical aid to the interpretation and validation of cluster analysis. Journal of Computational and Applied Mathematics **20**, 53–65 (1987)

[7] Caliński, T., Harabasz, J.: A dendrite method for cluster analysis. Communications in Statistics-theory and Methods **3**(1), 1–27 (1974)

[8] Davies, D.L., Bouldin, D.W.: A cluster separation measure. IEEE transactions on pattern analysis and machine intelligence (2), 224–227 (2009)

[9] Lin, T.-Y., Goyal, P., Girshick, R., He, K., Dollár, P.: Focal loss for dense object detection. In: Proceedings of the IEEE International Conference on Computer Vision (ICCV), pp. 2980–2988 (2017)

[10] Hosmer Jr, D.W., Lemeshow, S., Sturdivant, R.X.: Applied Logistic Regression. John Wiley & Sons, Hoboken, NJ (2013)

[11] Gorishniy, Y., Rubachev, I., Khrulkov, V., Babenko, A.: Revisiting deep learning models for tabular data. Advances in Neural Information Processing Systems **34**, 18932–18943 (2021)

[12] Salton, G., Buckley, C.: Term-weighting approaches in automatic text retrieval. In: Information Processing & Management vol. 24, pp. 513–523. Elsevier, Amsterdam, The Netherlands (1988)

[13] Devlin, J., Chang, M.-W., Lee, K., Toutanova, K.: Bert: Pre-training of

deep bidirectional transformers for language understanding. In: Proceedings of NAACL-HLT, pp. 4171–4186 (2019)

[14] Liu, Y., Ott, M., Goyal, N., Du, J., Joshi, M., Chen, D., Levy, O., Lewis, M., Zettlemoyer, L., Stoyanov, V.: Roberta: A robustly optimized bert pretraining approach. In: arXiv Preprint arXiv:1907.11692 (2019)

[15] Cui, Y., Che, W., Liu, T., Qin, B., Yang, Z., Wang, S., Hu, G.: Revisiting pre-trained models for chinese natural language processing. In: Proceedings of the 2020 Conference on Empirical Methods in Natural Language Processing: Findings, pp. 657–668 (2020)

[16] Beltagy, I., Peters, M.E., Cohan, A.: Longformer: The long-document transformer. In: arXiv Preprint arXiv:2004.05150 (2020)

[17] Zaheer, M., Guruganesh, G., Dubey, A., Ainslie, J., Alberti, C., Ontanon, S., Pham, P., Ravula, A., Wang, Q., Yang, L., *et al.*: Big bird: Transformers for longer sequences. In: Advances in Neural Information Processing Systems, vol. 33, pp. 17283–17297 (2020)

[18] Ngiam, J., Khosla, A., Kim, M., Nam, J., Lee, H., Ng, A.Y.: Multimodal deep learning. In: Proceedings of the 28th International Conference on Machine Learning (ICML), pp. 689–696 (2011)

[19] Baltrušaitis, T., Ahuja, C., Morency, L.-P.: Multimodal machine learning: A survey and taxonomy. IEEE Transactions on Pattern Analysis and Machine Intelligence **41**(2), 423–443 (2019)

[20] Tsai, Y.-H.H., Bai, S., Liang, P.P., Kolter, J.Z., Morency, L.-P., Salakhutdinov, R.: Multimodal transformer for unaligned multimodal language sequences. In: Proceedings of ACL, pp. 6558–6569 (2019)

[21] Lu, J., Batra, D., Parikh, D., Lee, S.: Vilbert: Pretraining task-agnostic visiolinguistic representations for vision-and-language tasks. In: Advances in Neural Information Processing Systems, vol. 32 (2019)

[22] Alberti, C., Andor, D., Bogatyy, I., Pitler, E., Devlin, J., Collins, M.: Fusion of detected objects in text for visual question answering. In: Proceedings of EMNLP-IJCNLP, pp. 2131–2140 (2019)

[23] Loshchilov, I., Hutter, F.: Decoupled weight decay regularization. In: International Conference on Learning Representations (ICLR) (2019)
